# Heterogeneous photocatalytic aldehyde alkylative amination for multicomponent synthesis of α-branched amines

**DOI:** 10.1093/nsr/nwag020

**Published:** 2026-01-19

**Authors:** Wei Ou, Qingzhu Xu, Qiyuan Wang, Ying Tao, Jie Wang, Zhenyuan Teng, Fuhua Li, Jie Ding, Wei Liu, Hao Hou, Akira Yamakata, Teruhisa Ohno, Bin Liu, Chenliang Su

**Affiliations:** International Collaborative Laboratory of 2D Materials for Optoelectronics Science and Technology of Ministry of Education, Institute of Microscale Optoelectronics, Shenzhen University, Shenzhen 518060, China; International Collaborative Laboratory of 2D Materials for Optoelectronics Science and Technology of Ministry of Education, Institute of Microscale Optoelectronics, Shenzhen University, Shenzhen 518060, China; International Collaborative Laboratory of 2D Materials for Optoelectronics Science and Technology of Ministry of Education, Institute of Microscale Optoelectronics, Shenzhen University, Shenzhen 518060, China; International Collaborative Laboratory of 2D Materials for Optoelectronics Science and Technology of Ministry of Education, Institute of Microscale Optoelectronics, Shenzhen University, Shenzhen 518060, China; International Collaborative Laboratory of 2D Materials for Optoelectronics Science and Technology of Ministry of Education, Institute of Microscale Optoelectronics, Shenzhen University, Shenzhen 518060, China; Department of Chemistry, Hong Kong Institute of Clean Energy (HKICE) & Center of Super-Diamond and Advanced Films (COSDAF), City University of Hong Kong, Hong Kong 999077, China; Department of Chemistry, Hong Kong Institute of Clean Energy (HKICE) & Center of Super-Diamond and Advanced Films (COSDAF), City University of Hong Kong, Hong Kong 999077, China; Department of Chemistry, Hong Kong Institute of Clean Energy (HKICE) & Center of Super-Diamond and Advanced Films (COSDAF), City University of Hong Kong, Hong Kong 999077, China; International Collaborative Laboratory of 2D Materials for Optoelectronics Science and Technology of Ministry of Education, Institute of Microscale Optoelectronics, Shenzhen University, Shenzhen 518060, China; International Collaborative Laboratory of 2D Materials for Optoelectronics Science and Technology of Ministry of Education, Institute of Microscale Optoelectronics, Shenzhen University, Shenzhen 518060, China; Graduate School of Natural Science and Technology, Okayama University, Okayama-shi 700-8530, Japan; Department of Applied Chemistry, Faculty of Engineering, Kyushu Institute of Technology, Kitakyushu-shi 804-8550, Japan; Department of Chemistry, Hong Kong Institute of Clean Energy (HKICE) & Center of Super-Diamond and Advanced Films (COSDAF), City University of Hong Kong, Hong Kong 999077, China; International Collaborative Laboratory of 2D Materials for Optoelectronics Science and Technology of Ministry of Education, Institute of Microscale Optoelectronics, Shenzhen University, Shenzhen 518060, China

**Keywords:** semiconductor photocatalysis, multicomponent reaction, TiO_2_, decarboxylation, α-branched amines

## Abstract

α-Branched amines and aza-heterocycles are crucial motifs commonly found in natural products and pharmaceutical compounds, and development of mild and sustainable methods to synthesize them from abundant feedstocks is urgently needed. Herein, a powerful and straightforward TiO_2_ (P25) photoredox catalytic decarboxylative alkylation strategy is designed for universal aldehyde alkylative amination (AAA) using readily available aldehydes, amines and carboxylic acids. This AAA approach facilitates rapid access to a wide range of complex α-branched amines, amino acids and aza-heterocycles (>80 examples). Notably, this strategy can also be extended to the reductive alkylation of inert amides, which is of significant practical utility. To showcase its effectiveness, this AAA protocol was employed to streamline the synthetic routes of numerous pharmaceutical-related molecules, which can be easily scaled up using a recirculating-flow system. Mechanistic studies suggest that the photogenerated holes on TiO_2_ oxidized the chemisorbed carboxylic acid to release CO_2_ and R• radicals. Simultaneously, the photogenerated electrons reduced Ti(IV) to Ti(III), enabling the retrieval of an electron from reactive intermediates to complete the catalytic cycle. Additionally, transient absorption decay and theoretical calculations revealed that mixed-phase TiO_2_ (P25) exhibited enhanced charge transfer dynamics and thermodynamics, resulting in superior photocatalytic activity. This study provides guidance for future materials design in oxidative decarboxylation.

## Teaser

This work develops an efficient and straightforward three-component a-branched amine synthesis strategy from accessible aldehydes, amines and carboxylic acids, and uncovers the activity trend of different TiO2 crystal phases in photocatalytic decarboxylative addition.

## INTRODUCTION

**Figure 1.**
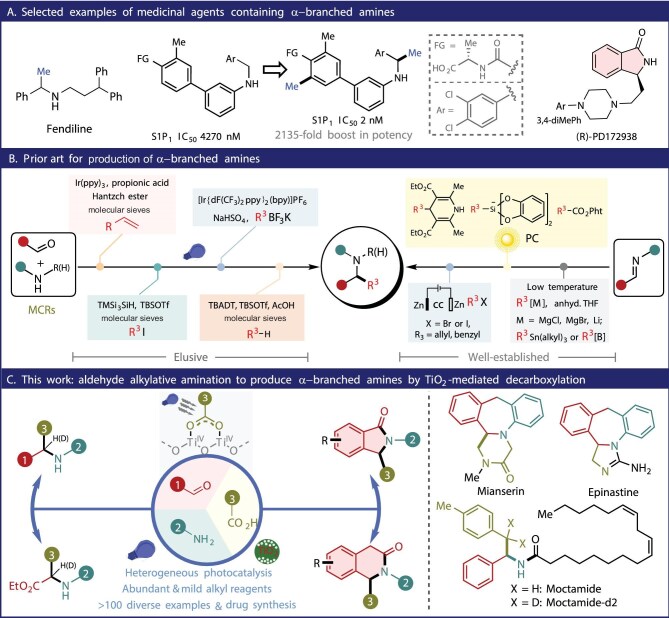
Bioactive α-branched amines and their main synthetic strategies.

The α-branched amines and aza-heterocycles, as essential units, are widely found in biological and pharmaceutical molecules, gaining intense interest from the broad scientific community [1–3]. Impressively, a seemingly simple conversion of an α-C–H bond in amines to a C–Me bond has the potential to greatly enhance the IC_50_ value of a drug candidate, giving rise to the ‘magic methyl effect’ [4–6]. This effect has been observed in many compounds, such as sphingosine-1-phosphate inhibitor (S1P_1_), where the IC_50_ value can increase by up to 2135-fold with the introduction of methyl groups [7], especially at the α-position of amines (Fig. 1A), highlighting the impact of α-branched amine units on pharmacological properties, which can bring huge potential for drug discovery and development.

The direct addition of alkyl fragments to aldehyde-derived imines/iminium ions would be an ideal and step-economic approach for multicomponent synthesis of α-branched amines [8–15]. Organometallic reagents are powerful for generation of reactive carbon-based nucleophiles for imine addition, but this approach is incompatible with *in situ* imine formation from carbonyls and amines, and the high reactivity of organometallic reagents often necessitates meticulous preparation under anhydrous conditions by skilled operators. In 2018, Gaunt’s group developed a groundbreaking strategy, named carbonyl alkylative amination (CAA), for single-step, multicomponent synthesis of α-branched amines via direct addition of α-amino radicals to simple alkenes [16,17]. Soon after, the same group proposed a different visible light-driven CAA method via addition of alkyl radicals from reduction of alkyl iodides that have poor natural and commercial accessibility to *in situ*-generated iminium ions for rapid synthesis of α-branched tertiary amines [18].

Carboxylic acids are low-cost and industrially abundant feedstocks, which act as ideal alkylating reagents instead of alkyl iodides [19–21]. Thus, development of a general and direct aldehyde alkylative amination (AAA) from carboxylic acids, aldehydes and amines for multicomponent synthesis of important α*-*branched amines with robust and reusable heterogeneous catalysts are highly attractive but very challenging. This is due to the need for a strong oxidant to activate the typically inert carboxylic acids while simultaneously tolerating oxidant-sensitive aldehydes. Therefore, identifying efficient photocatalysts that provide proper adsorption and strong oxidation capability for the selective and effective oxidative decarboxylation of carboxylic acids, including acetic acid [*E*_(AcO_^•/−^_)_ = 2.6 V vs. normal hydrogen electrode (NHE)], is crucial [22].

TiO_2_ is an exceptionally safe, inexpensive and environmentally friendly semiconductor photocatalyst with strong oxidative ability (valence band at 2.7 V vs. NHE) [23–29], capable of oxidative decarboxylation of simple carboxylic acids, including acetic acid. For example, Manley and co-workers demonstrated that TiO_2_-mediated decarboxylation of carboxylic acids could generate alkyl radicals for alkylations and annulations of electron-deficient alkenes with very limited reaction scope [27]. In continuation of our interests on the use of semiconductor photocatalysts for constructing value-added organic molecules [30–33], we herein designed a general TiO_2_ photoredox catalytic oxidative decarboxylation of carboxylic acids for universal AAA to greatly simplify the synthesis process of α-branched secondary amines (Fig. 1C). Our strategy takes advantage of carboxylic acids as alkylating agents, which are readily available from both natural and commercial sources and provide an acidic environment to promote *in situ* formation of imines from aldehydes and amines. Benefitting from the mild reaction conditions with simple operation, this protocol can be applied to synthesize a wide range of α-branched amines, amino acids and aza-heterocycles (>80 examples, up to 93% isolated yields) with excellent functionality tolerance, which is easily scalable in a recirculating-flow system, opening a powerful AAA approach using carboxylic acids, aldehydes and amines to produce bioactive α-branched amines (e.g. mianserin and epinastine). We further developed a novel thermo-photorelay catalytic system enabling direct deoxygenative alkylation of secondary amides to α-branched amines using carboxylic acids as the alkylating reagents. This strategy not only broadens the synthetic utility of amides, but also showcases the potential of integrating different catalytic modes in this mild TiO_2_-photocatalytic decarboxylative alkylation system.

## RESULTS AND DISCUSSION

The photocatalytic decarboxylative AAA reaction was first studied using model substrates, as listed in Table S5 (see Supplementary data for more details). Condition screening experiments found that the highest yield (76%) of the desired products could be achieved under the following conditions: aldehydes (1.0 equiv.), amines (1.0 equiv.), carboxylic acids (2.0 equiv.), magnesium sulfate (2.0 equiv.) and TiO_2_ (100 mg/0.5 mmol) in acetonitrile under 390 nm light irradiation. Almost no products were obtained when changing TiO_2_ to other semiconductors or homogeneous photocatalysts (Entries 2–7). Other solvents such as dichloromethane (DCM), dimethyl sulfoxide (DMSO) and tetrahydrofuran (THF) gave decreased yields (Entries 8–11). Finally, control experiments demonstrated that TiO_2_, light and MgSO_4_ are all essential for the AAA reaction (Entries 12–14).

**Figure 2.**
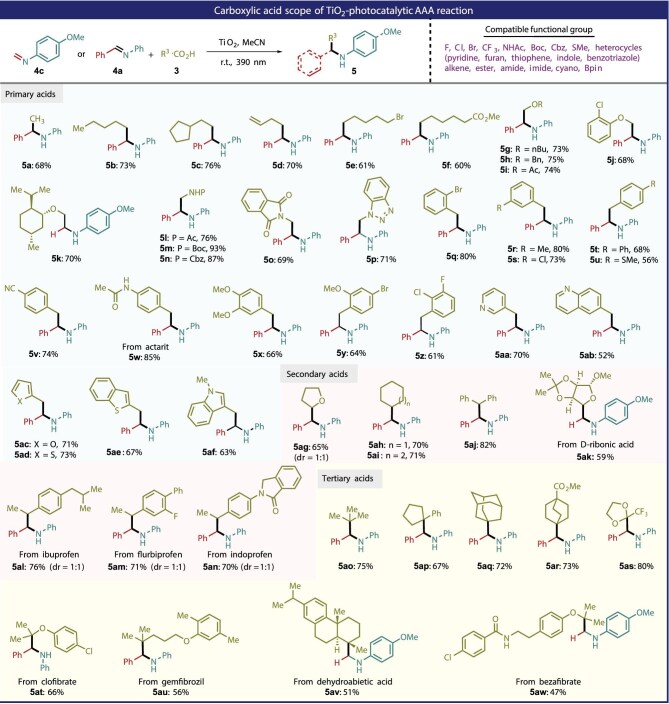
The scope of carboxylic acids in TiO_2_ photocatalytic decarboxylative alkylation.

Under the optimal reaction conditions, the scope of carboxylic acids was investigated (Fig. 2). Our strategy showed a broad reaction scope with excellent functionalities tolerance. A wide range of natural/commercial primary (**5a**–**5af**), secondary (**5ag**–**5an**) and tertiary (**5ao**–**5aw**) carboxylic acids were compatible. For primary carboxylic acids, sensitive functional groups ranging from olefins, halogens, ester, *tert*-butoxycarbonyl and benzyloxycarbonyl groups were tolerable, which gave the corresponding products (**5e**–**5p**) in 61%–93% yields. The α-heteroatom-substituted carboxylic acids could readily undergo decarboxylation–alkylation. Some of the products are key building blocks for the synthesis of ethylene glycol amine and ethylenediamine-related natural compounds and pharmaceutical products. For example, the use of (−)-menthoxyacetic acid as an alkylating reagent smoothly produced ethylene glycol amine derivative **5k** with multiple chiral centers in 70% yield. Benzyl, pyridyl, quinolyl, furyl, thienyl and indolyl acetic acids were all suitable for this TiO_2_ photocatalytic decarboxylative alkylation. For secondary carboxylic acids, both cyclic and acyclic substrates could be effectively transformed into the corresponding products **5ag**–**5ak** in 59%–82% yields.

Impressively, commercial drugs such as ibuprofen, flurbiprofen and indoprofen could be directly served as the alkylating reagents, furnishing the desired drug-related products in acceptable yields. Finally, for tertiary carboxylic acids, to our delight, not only chain and cyclic tertiary substrates but also commercial drugs containing such units reacted well to give **5ao**–**5aw** in 47%–80% yields. Installation of trifluoromethylacyl in bioactive molecules has gained intense research interest in promoting pharmaceutical activities [34]. Motivated by this, the masked acyl CF_3_-acid was applied as the alkylating reagent, which furnished the corresponding protected carbonyls product **5as** in 80% yield.

**Figure 3.**
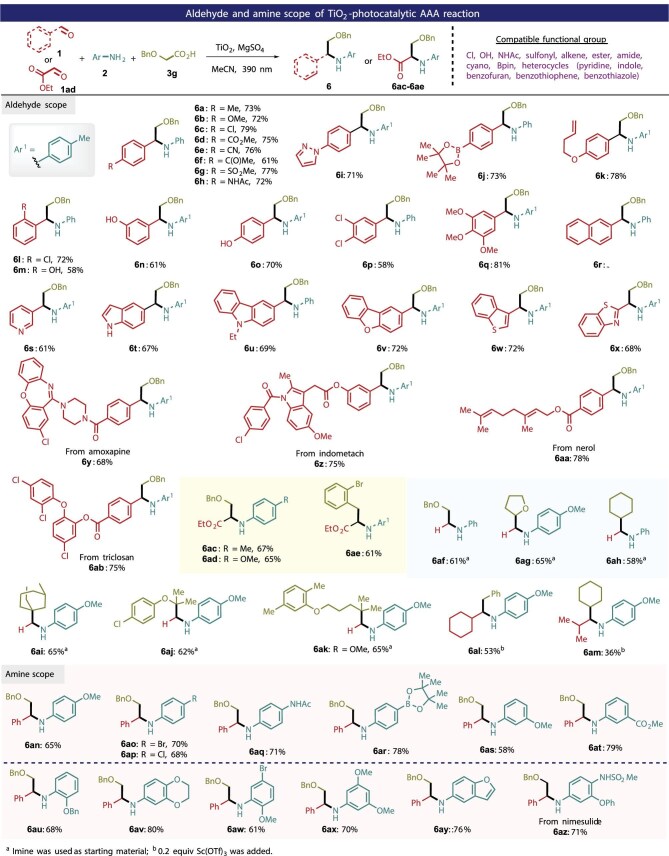
Scope of aldehyde and amine substrates.

The use of carboxylic acids could promote *in situ* generation of imines from aldehydes and amines, enabling decarboxylative alkylation for AAA. Subsequently, the generality of aldehydes and amines to access α-branched secondary amines was evaluated. As shown in Fig. 3, a wide range of functionalized benzaldehydes could be successfully used in the AAA reaction, affording the desired products **6a**–**6q** with 58%–81% yields. This protocol features a broad functionalities tolerance, compatible with substrates bearing ester, cyan, keto, hydroxy, amide, sulfonyl, boronic ester and allyl groups. Heterocyclic aromatic aldehydes including pyridine, indole, carbazole, benzo[*b*]thiophene and benzo[*b*]thiazole furnished the corresponding α-branched secondary amines **6r**–**6x** in 61%–79% yields.

Late-stage functionalization of drugs and natural products is significant. We performed alkylative amination of several natural/pharmaceutical molecule-derived aldehydes, producing natural/pharmaceutical-related complex amine products **6y**–**6ab** in impressive yields. Interestingly, this protocol allowed us to prepare valuable α*-*substituted glycine derivatives **6ac**–**6ae** in synthetically useful yields using acetaldehyde ester, aniline and carboxylic acids. When the corresponding formaldehyde imines were employed as substrates, the reaction proceeded smoothly to afford the secondary amines **6af**–**6ak** in 58%–65% yields. Furthermore, we investigated AAA with alkyl aldehydes and found that the addition of the Lewis acid Sc(OTf)_3_ could markedly accelerate the reaction, delivering the secondary amines **6al** and **6am** in 53% and 36% yield, respectively.

Exploration of the scope of anilines indicated that the electronic properties on the aromatic ring of anilines had little impact on their reactivities. Anilines bearing secondary amide, borate ester, ester group and halogen substituents could be effectively aminated, giving **6aq**–**6ax** in 58%–80% yields. A reaction using benzofuranamine afforded the desired product **6ay** in 76% yield. This method could also be applied to pharmaceutical-related anilines such as nimesulide derivative **6az**.

**Figure 4.**
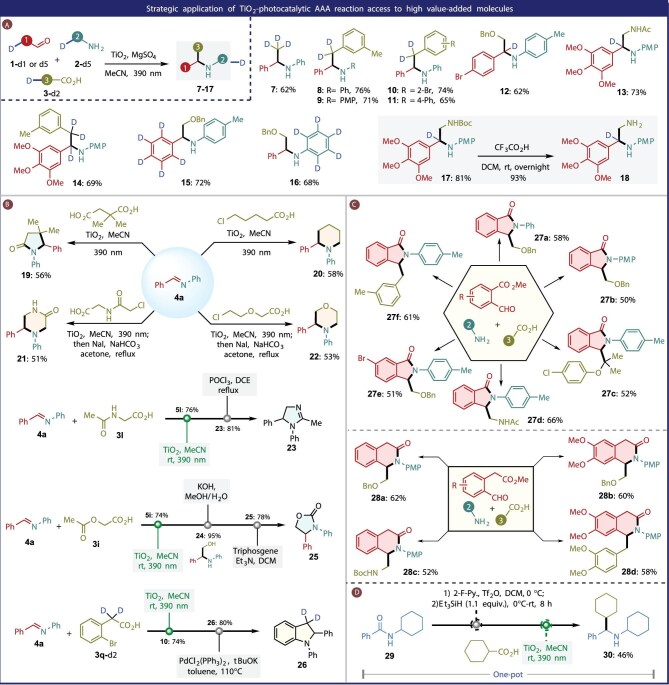
Reaction extension and product diversification.

With an understanding of the scope of this AAA strategy, rapid synthesis of heterocyclic and pharmaceutical-related α*-*branched amines was designed (Fig. 4). Since ‘deuterium switch’ at the metabolic site of pharmaceutical molecules could exert a significant influence on their efficacy and safety [35–43], we tried to apply this AAA protocol for the synthesis of deuterated pharmaceutical amines and their related building blocks. Regioselective synthesis of β-deuterated amines is useful but very challenging due to the poor reactivity of β-amino C–H bonds [44,45]. Using readily available or easily preparable deuterated carboxylic acids, this AAA protocol could be used to synthesize a variety of β-deuterated amines (**7**–**11**) that could not be accessed easily by other methods. Installing CD_3_ on the α-position of pharmaceutical amines could potentially have both deuterium isotope and magic methyl effects. To our delight, commercially available deuterated acetic acid could be directly used in the AAA reaction to install the CD_3_ unit. Moreover, using easily obtainable deuterated benzaldehydes individually or in combination with deuterated carboxylic acids, α-deuterated amines **12, 13, 17** and α,β-deuterated amine **14** could be synthesized, respectively. When commercial deuterated aniline (d5) was used in the AAA reaction, the corresponding product **16** could be produced smoothly. Valuable deuterated ethylenediamine derivative **18** could be synthesized by removing the Boc group of compound **17** (Fig. 4A). To evaluate the potential of the AAA reaction in α-branched cyclic amine or amide synthesis, multiple types of domino reactions were designed (Fig. 4B). For example, carbon radical addition of imine using 2,2-dimethylsuccinic acid followed by lactamization afforded lactam **19** in 56% yield with high selectivity, which probably originated from the higher stability of the tertiary radical compared to the primary radical. Starting with 5-chloroalkyl carboxylic acid, carbon radical addition of imine followed by intramolecular alkylation produced cyclic amine **20** in 58% yield. Carboxylic acids with oxo or secondary amide appearing in chains were compatible, requiring additional iodation using NaI to give piperazin-2-one derivative **21** and morpholine derivative **22** in acceptable yields. The AAA reaction products ethylene glycol amine derivative **5l** and ethylenediamine derivative **5i** can be readily converted to imidazole **23** and oxazolidinone **25**, respectively, in one and two known steps [46,47]. Taking the obtained β-deuterated secondary amine **10** as the reactant, deuterated indoline **26** was efficiently synthesized in 80% yield by a sequential Pd-catalyzed intramolecular amination.

Isoindolinones are widely present in natural alkaloids and pharmaceutical compounds, displaying impressive biological activities [48]. Based on our established AAA strategy, and combined with the intramolecular lactam cyclization of secondary amine, we proposed a straightforward approach to synthesize 3-substituted isoindolinones using methyl 2-formylbenzoates (Fig. 4C), affording the corresponding 3-substituted isoindolinones **27a**–**27f** in acceptable yields. Impressively, isoquinolinones **28a**–**28d**, the key building blocks of many pharmaceutical alkaloids, could be well produced based on similar principles, by using methyl 2-formylphenylacetate as the starting material [49].

Amides are ideal precursors for amine synthesis, yet their inherent chemical inertness poses significant challenges for their direct transformations. Herein, we proposed a Tf_2_O-mediated amide activation coupled with TiO_2_-photocatalytic decarboxylative alkylation, enabling a streamlined deoxygenative photochemical alkylation of secondary amides (Fig. 4D). Guided by this approach, *sec-*amide **29** was first semi-reduced to imine intermediate by the Tf_2_O/Et_3_SiH system [50,51]. To the residue from this step were then added cyclohexanecarboxylic acid, TiO_2_ and MeCN, and the mixture was irradiated at 390 nm to afford the desired amine **30** in 46% yield.

**Figure 5.**
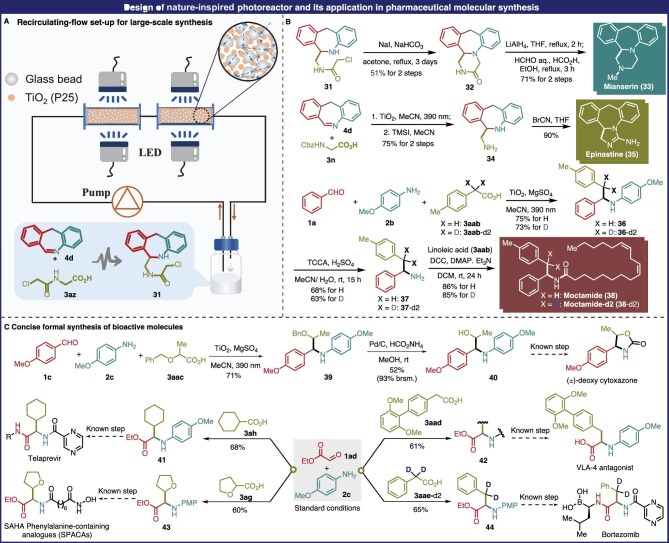
Design of a flow heterogeneous photocatalytic reactor for APIs synthesis.

To demonstrate the practical application potential of the AAA protocol, gram-scale synthesis towards making active pharmaceutical intermediates (APIs) and alkaloids was developed (Fig. 5). Flow chemistry offers an effective approach for large-scale synthesis, especially in homogeneous photocatalytic systems. However, for heterogeneous photocatalytic systems, the poor penetration depth of incident light will heavily limit its efficiency. Inspired by cloud reflection and refraction [52], we designed a flow heterogeneous photocatalytic system using glass beads to mimic cloud reflection and refraction co-packed with TiO_2_ (P25), as shown in Fig. 5A. Mianserin is an oral antidepressant drug, which has been approved for use as a racemic mixture [53]. Large-scale production of mianserin is thus of great importance, and we used our designed flow photocatalytic reactor to synthesize the key mianserin intermediate **31** in gram scale. A mixture of 6 mmol (∼1.16 g) 11*H*-dibenzo[*b,e*]azepine **4d** and 12 mmol (∼1.81 g) glycine derivative **3az** was recirculated through the photoreactor co-packed with glass beads and P25 nanoparticles (refer to Supplementary data for details) in 48 h, successfully affording the corresponding crude product **31** that was subsequently treated with NaI, producing the pure piperazin-2-one skeleton **32** in 51% yield. Reduction of **32** using LiAlH_4_, followed by the Eschweiler–Clarke methylation reaction [54], produced the desired mianserin **33** in 71% yield. Epinastine **35**, an effective antihistamine [55], could be also synthesized with 67.5% yield in a similar way over three steps. Photocatalytic multicomponent synthesis of linearly α-substituted pharmaceutical amines was also demonstrated to make moctamide **38**, an antihyperlipoproteinemic agent [56,57]. Deuterated moctamide **38-d2** that cannot be easily obtained by other methods, was well synthesized using the corresponding deuterated carboxylic acids (Fig. 5B).

The 2-amino alcohol **40** was a key intermediate for the preparation of deoxy cytoxazone [58], a potential drug for immunotherapy and allergen immune response. With the help of the AAA reaction, a two-step synthesis of 2-amino alcohol **40** is illustrated in Fig. 5C. Since bortezomib, the first α*-*alkyl-substituted amino acid drug, was approved by the Food and Drug Administration (FDA), the compounds containing α-alkyl-substituted amino acids have been garnering increasing attention. Numerous synthetic strategies to make α-alkyl-substituted amino acid drugs widely used in clinical treatment are being developed [59–66]. Unfortunately, the existing methods require use of non-commercial alkyl reagents, such as *N*-hydroxyphthalimide (NHP) esters [59], Katritzky salts [60] or metal catalysts [65,66]. Herein, we provided a more concise route to synthesis of α‑substituted glycine derivatives by our developed method. Similarly, the key intermediates **41, 42** and **43** for the synthesis of telaprevir [62], the novel-pattern histone deacetylase (HDAC) inhibitors, VLA-4 antagonists [63,64] and suberoylanilide hydroxamic acid (SAHA) phenylalanine-containing analogs (SPACAs) [65] could be readily achieved from aldehyde **1ad**, amine **2c** and the corresponding carboxylic acids by one step, which highlighted the efficiency practicality of this tactic in the α-alkylation of glycine drugs. Finally, deuterated bortezomib precursor **44** could be conveniently synthesized from aldehyde **1ad**, amine **2c** and deuterated carboxylic acid **3aae-d2** [66].

**Figure 6.**
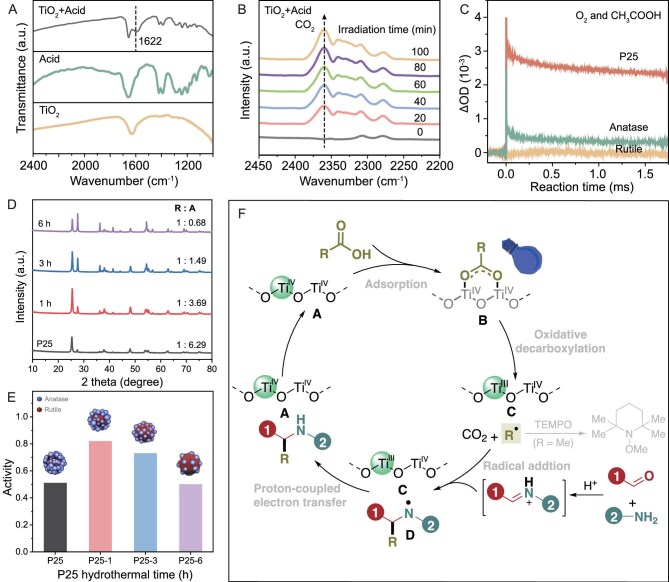
Mechanism studies. (A) DRIFT spectra of TiO_2_ (P25), TiO_2_ (P25) after adsorption of hexahydrobenzoic acid and free hexahydrobenzoic acid; (B) DRIFT spectra of TiO_2_ (P25)-bound hexahydrobenzoic acid during irradiation with a 390 nm light; (C) comparison of transient absorption decay among P25, anatase and rutile at 2000 cm^−1^ under O_2_ and CH_3_CO_2_H atmosphere; (D) XRD patterns of TiO_2_ (P25) after hydrothermal treatment at different times; (E) reactivity of TiO_2_ (P25) after hydrothermal treatment at different times; (F) a probable mechanism of the TiO_2_-photocatalytic decarboxylative AAA reactions from carboxylic acids, aldehydes and amines.

To probe the photocatalytic AAA reaction mechanism, the interaction between carboxylic acid and TiO_2_ (P25) was first studied by diffuse reflectance infrared Fourier transform spectroscopy (DRIFTS) [67]. Several new vibrational features appeared in the wavenumber region from 1000 to 1700 cm^−1^ after adsorbing hexahydrobenzoic acid onto the TiO_2_ (P25) surface (Fig. 6A), which could be assigned to the aliphatic C–H and C=O bond stretching [68]. To further study the light-induced reaction, *in situ* DRIFTS measurements were conducted over the hexahydrobenzoic acid-adsorbed TiO_2_ (P25) surface under light illumination, and the results are presented in Fig. 6A. Prior to light irradiation, no infrared absorption peak of CO_2_ was observed on the hexahydrobenzoic acid-adsorbed TiO_2_ (P25). The intensity of the CO_2_ absorption peak continuously increased with increasing illumination time (Fig. 6B), attesting to the photocatalytic decarboxylation process. During this process, carbon radicals would be generated along with the release of CO_2_. To capture carbon radicals, 2.0 equiv. of 2,2,6,6-tetramethyl-1-piperidinyloxy (TEMPO) was added into the photoreaction system, and TEMPO-Me was detected by gas chromatography–mass spectrometry (GC–MS).

Compared to pure anatase or rutile-phase TiO_2_, mixed-phase P25 exhibited significantly better photocatalytic AAA activity (Fig. S4). To reveal the underlying reasons, transient absorption spectroscopy measurements were first performed to probe the charge transfer dynamics from photogenerated holes to chemisorbed carboxylic acid on different TiO_2_ (Fig. 6B). A larger intensity was observed on P25 compared to pure anatase and pure rutile TiO_2_ in a CH_3_COOH environment at 2000 cm^−1^, indicating faster hole transfer to CH_3_COOH over the P25 surface [69–72]. Density functional theory (DFT) calculations demonstrated that the oxygen vacancy (corresponding to the formation of Ti^3+^) [73] formation energy at the interface of P25 is significantly lower compared to that on pure anatase and the rutile TiO_2_ surface (Fig. S6). This suggests that Ti^3+^ formation is easier at the P25 interface, which is consistent with our observation of a deeper violet color (indicating Ti^3+^) in the reaction solution when P25 was used as a photocatalyst.

To elucidate the influence of interfacial interactions between distinct TiO_2_ phases on photocatalytic activity, a series of TiO_2_ samples with controlled rutile/anatase phase ratios were prepared based on our previously reported method through hydrothermal treatment of commercial P25 in 6 M HCl solution at 200°C for 1–6 h [74]. Accordingly, the samples were designated as P25-*x*, where *x* denotes the hydrothermal treatment duration in hours. X-ray diffraction (XRD) patterns (Fig. 6D) verify gradual phase transition, with increasing rutile content corresponding to prolonged treatment time. The AAA reaction performance of as-prepared TiO_2_ photocatalysts was then investigated under standard conditions. As shown in Fig. 6E, the results demonstrate a clear phase-dependent activity trend, with P25-1 and P25-3 exhibiting superior activity compared to commercial P25. These findings underscore the critical role of TiO_2_ phase junctions in optimizing the photocatalytic efficiency. Notably, the optimal rutile/anatase ratio (∼1:3.7), identified through this systematic investigation, corresponds to peak activity for the AAA reaction.

With all these results, a probable mechanism is proposed (Fig. 6F): first, the chemisorbed carboxylic acid **B** on P25 was cleaved to free carbon radicals R• and CO_2_ by photogenerated holes. Photogenerated electrons helped to reduce Ti (IV) to Ti (III)-state **C**. The sequential addition of carbon radicals R• to the iminium ion intermediate that was generated *in situ* from acid-promoted condensation of aldehyde and amine released *N*-centered radical **D**. Proton-coupled electron transfer between **C** and **D** furnished product and closed the catalytic cycle.

## CONCLUSION

In conclusion, we have established a general TiO_2_-mediated photocatalytic decarboxylative AAA reaction for multicomponent synthesis of high value α-branched amines and aza-heterocycles. Notably, a wide range of natural or commercial primary, secondary and tertiary carboxylic acids can be directly utilized as modular alkylating reagents to install desired alkyl units at the α-position of amines and aza-heterocycles. As demonstrated herein, this multi-component synthetic procedure, utilizing readily accessible starting materials (aldehyde, amine and carboxylic acid, as well as inert amide) and offering broad reaction scopes, significantly simplifies the synthesis of numerous valuable (deuterated) building blocks and pharmaceuticals. Furthermore, the scalability and industrial availability of the starting materials and catalysts make this approach promising for the large-scale synthesis of bioactive α-branched secondary amines.

## Supplementary Material

nwag020_Supplemental_File
