# Supplementary material for: Heterogeneous photocatalytic aldehyde alkylative amination for multicomponent synthesis of α-branched amines
Source: Natl Sci Rev. 2026 Jan 19;13(5):nwag020. doi: 10.1093/nsr/nwag020 (PMC12976600; doi:10.1093/nsr/nwag020)

Supporting Information

Heterogeneous Photocatalytic Aldehyde Alkylative Amination for Multicomponent Synthesis of α-Branched Amines

Wei Ou,[a] Qingzhu Xu,[a] Qiyuan Wang,[a] Ying Tao,[a] Jie Wang,[a] Zhenyuan Teng,[b] Fuhua Li,[b] Jie Ding,[b] Wei Liu,[a] Hao Hou,[a] Akira Yamakata,[c] Teruhisa Ohno,[d] Bin Liu,[b]* Chenliang Su[a]*

[a]International Collaborative Laboratory of 2D Materials for Optoelectronics Science and Technology of Ministry of Education, Institute of Microscale Optoelectronics, Shenzhen University, Shenzhen 518060, P. R. China

[b]Department of Chemistry, Hong Kong Institute of Clean Energy (HKICE) & Center of Super-Diamond and Advanced Films (COSDAF), City University of Hong Kong, Hong Kong SAR 999077, P. R. China

[c]Graduate School of Natural Science and Technology, Okayama University, Okayama-shi, Japan

[d]Department of Applied Chemistry, Faculty of Engineering, Kyushu Institute of Technology, Kitakyushu-shi, Japan

Table of Contents

1. General information S2

2. Experimental procedures S7

3. Characterization of products S9

4. Mechanistic studies S71

5. References S74

6. ^{1}H NMR and ^{13}C NMR spectra...S76

1. General information.

NMR Spectra were recorded on a Bruker AC 600 spectrometer at 25 ℃ in the solvents indicated. Chemical shifts (*δ*) are reported in ppm and respectively referenced to internal standard Me_4Si and solvent signals (Me_4Si, 0 ppm for ^1H NMR and $CDCl_3$, 77.0 ppm for ^{13}C NMR). Mass spectra were recorded on a Bruker Dalton Esquire 3000 plus LC-MS apparatus (ESI direct injection). HRMS spectra were recorded on a 7.0T FT-MS apparatus. Silica gel (300-400 mesh) was used for flash column chromatography, eluting (unless otherwise stated) with EtOAc/ *n*-hexane mixture. All other commercially available compounds were used as received. The AEROXIDE TiO_2 P25 was purchased from Guangzhou Heqian Trading Co., Ltd. Dry dichloromethane was distilled over calcium hydride under N_2.

Table S1. The structure of carboxylic used

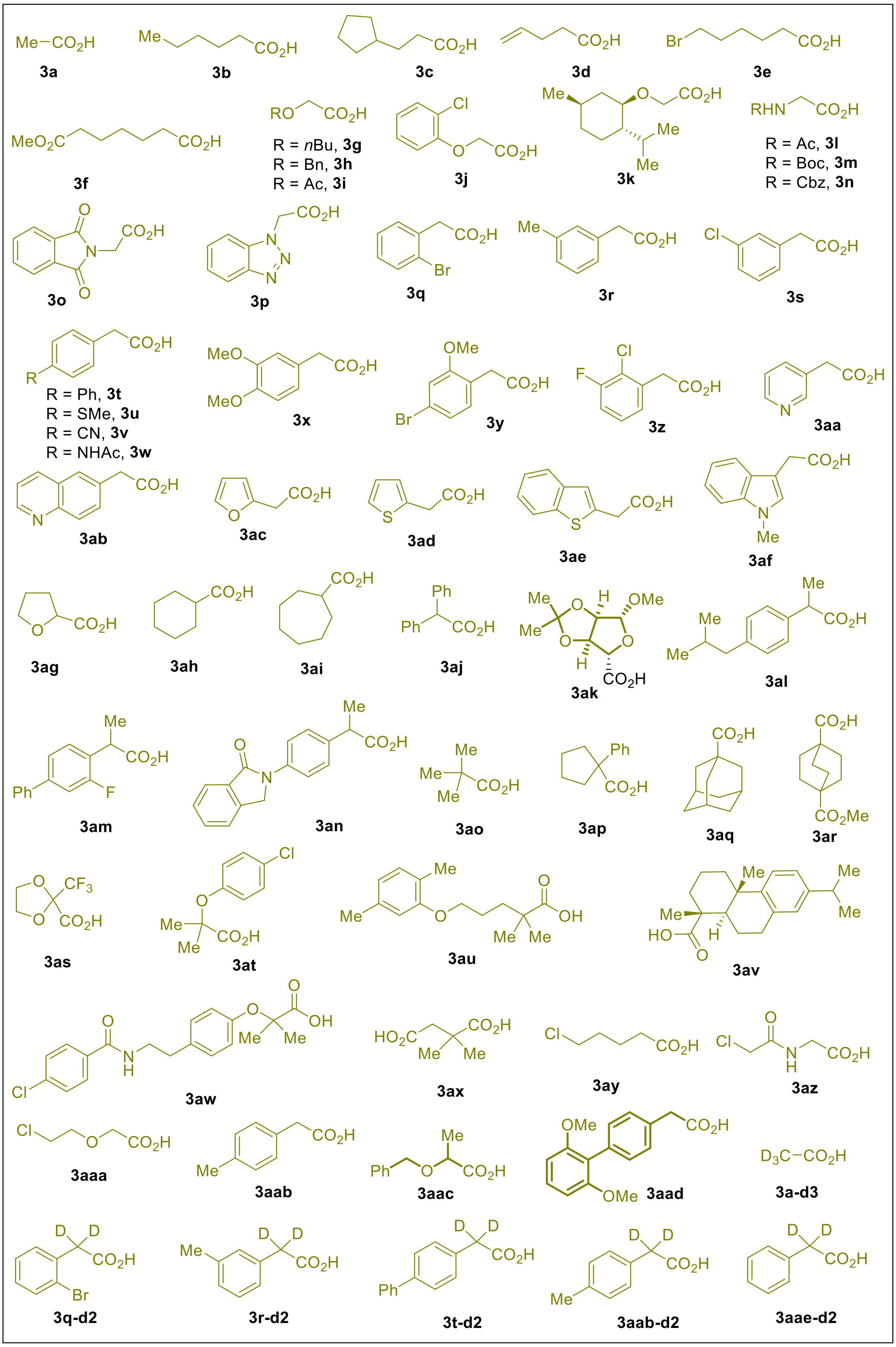

Table S2. The structure of aldehyde used

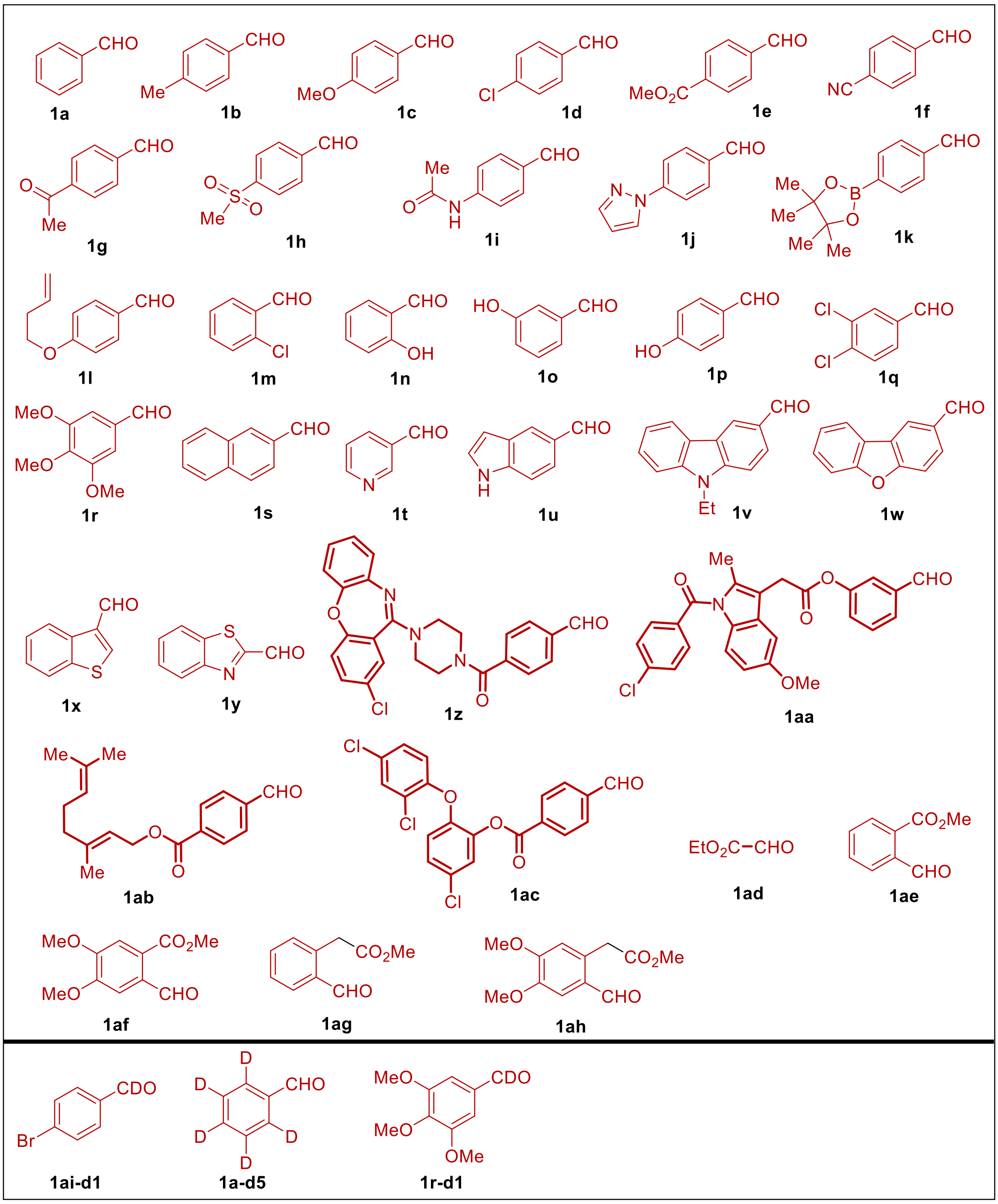

Table S3. The structure of amine used

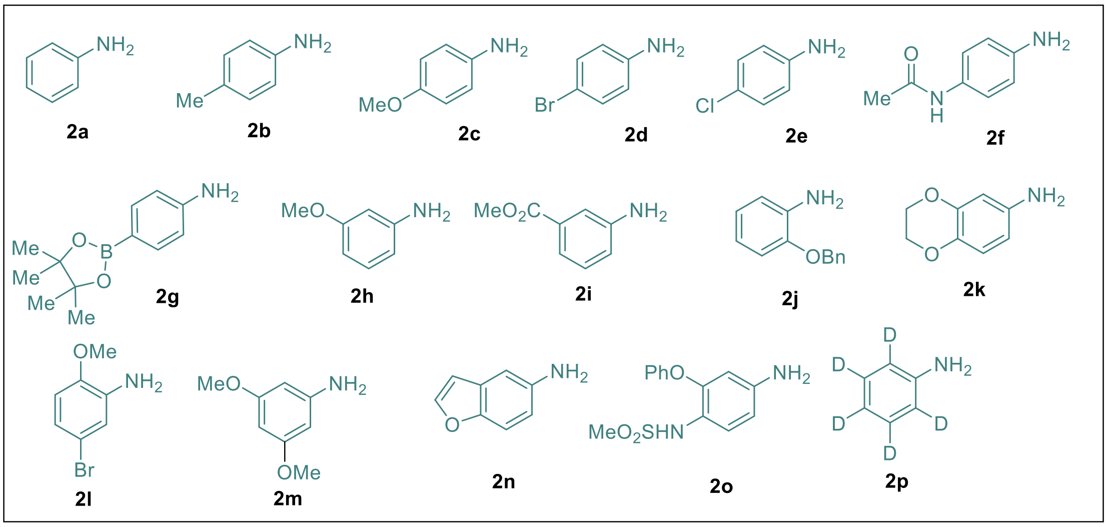

Table S4. The structure of imine used

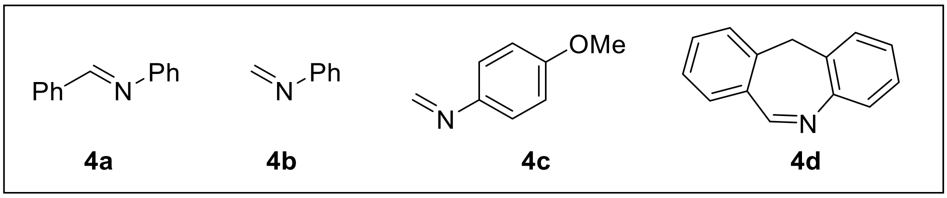

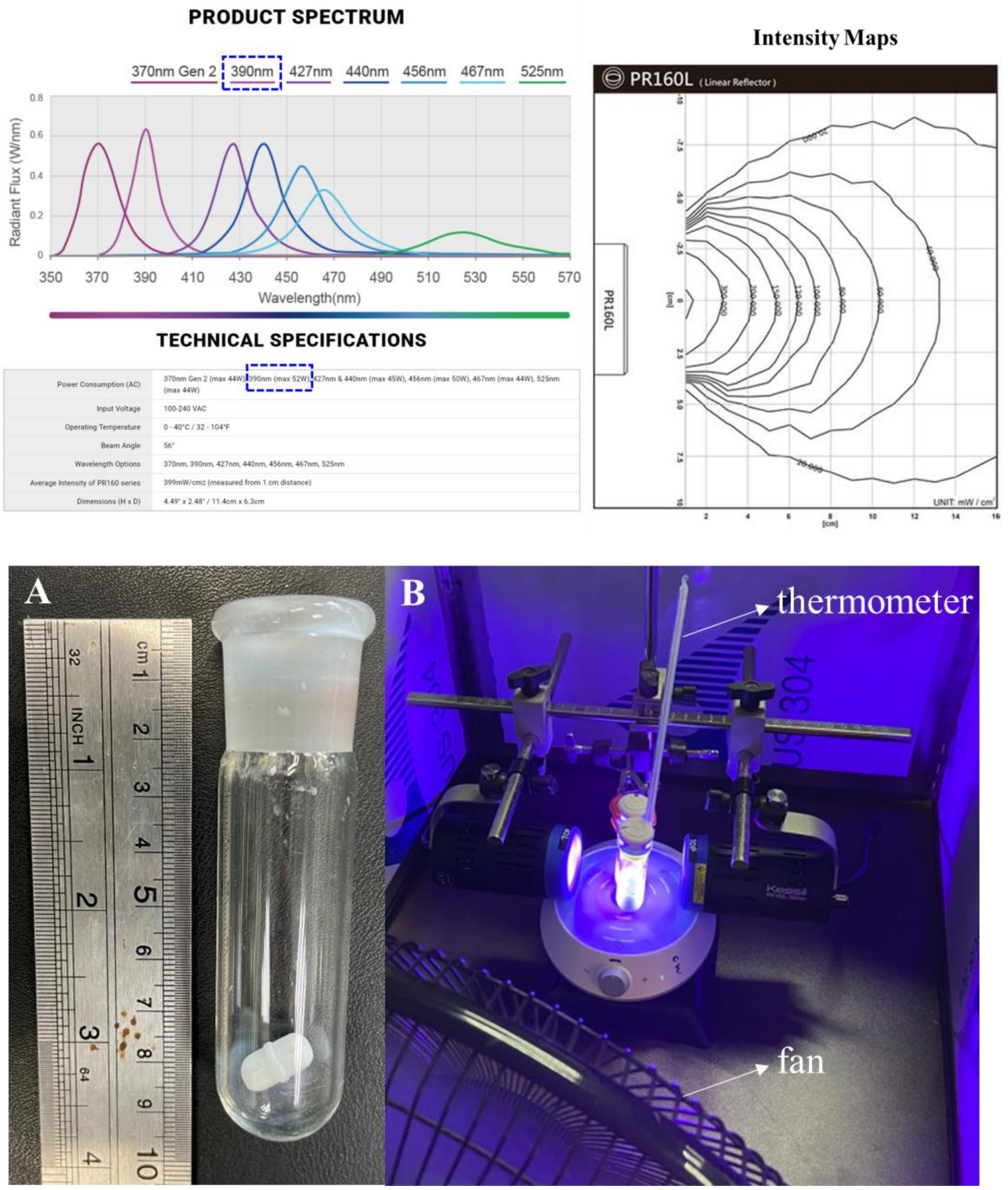

Figure S1. Standard setup and the detailed information about the light source (Kessil LED PR160L) for the photocatalytic reactor. *Note: The reaction tube is made of borosilicate glass and no filters are used.*

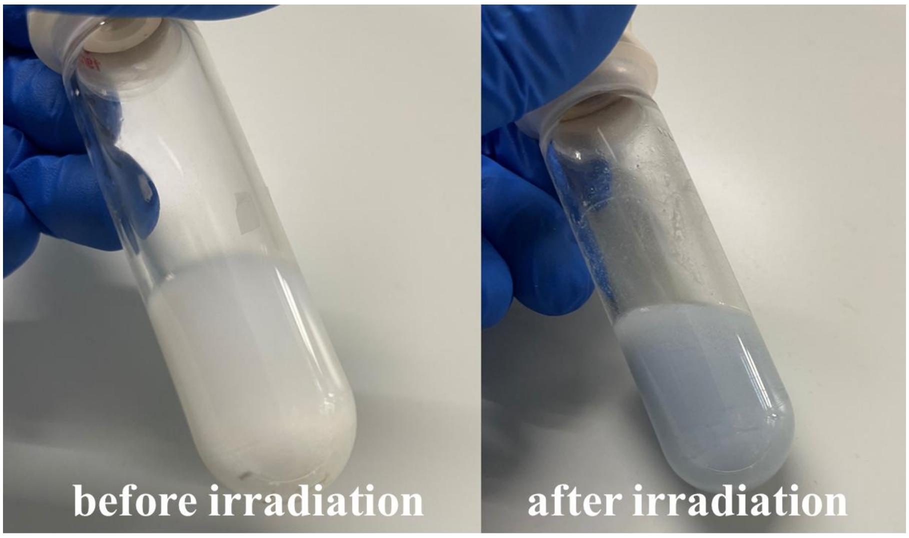

Figure S2. The picture of before irradiation and after irradiation.

2. Experimental Procedures

Characterization of the TiO_2 catalyst

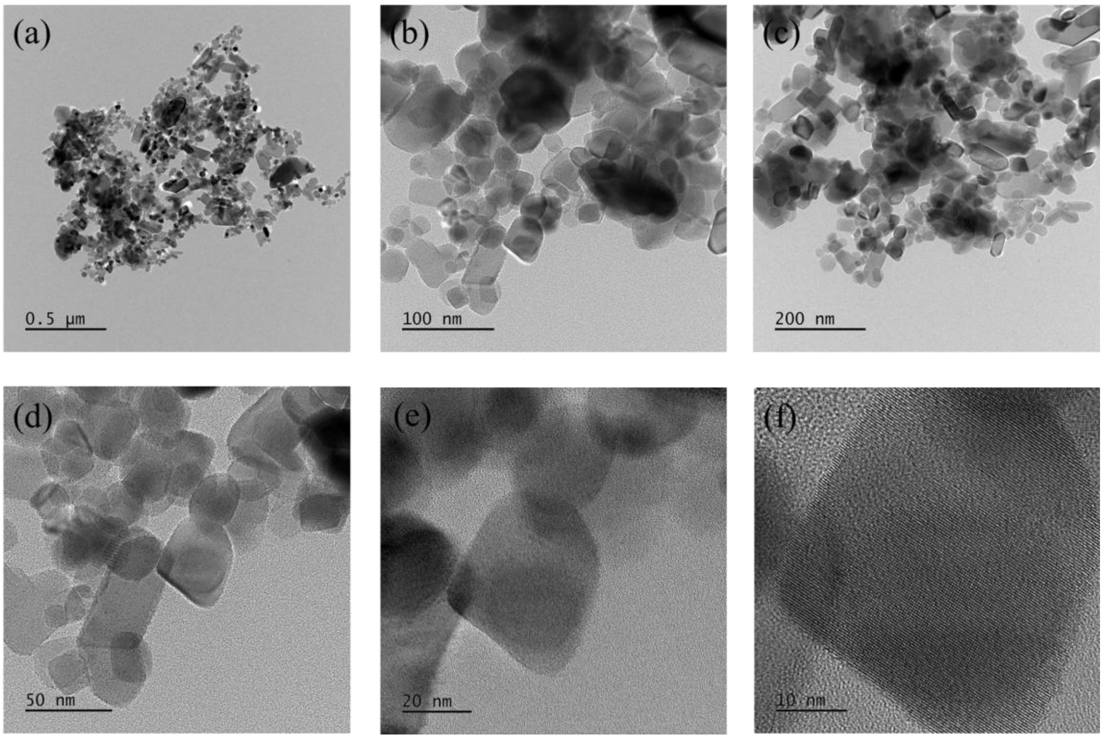

Figure S3. TEM images of TiO_2 (P25).

General procedure for the reaction condition screening

Benzaldehyde (0.2 mmol, 1.0 equiv.), aniline (0.2 mmol, 1.0 equiv.), benzyloxyacetic acid (0.4 mmol, 2.0 equiv.), semiconductor photocatalyst (40 mg) or homogeneous photocatalyst (10 mg) and $MgSO_4$ (0.4 mmol, 2.0 equiv.) were added to an oven dried 10 mL quartz reaction tube. The reaction tube was then evacuated and backfilled with N_2 three times, and 3 mL of degassed anhydrous solvent was added. The reaction mixture was irradiated using Kessil lamp (PR-160, 390 nm) and cooled with a fan for 24 h.

Table S5. Reaction condition screening

Optimization of the AAC reaction conditions

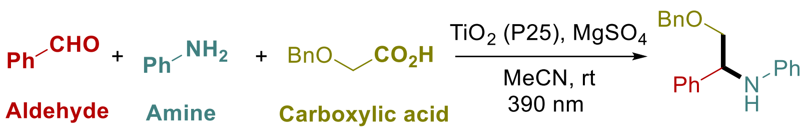

Entry	Deviation from the standard conditions	Yield (%)[b]
1	none	76
2	ZnO (390 nm) instead of TiO_2	0
3	$BiVO_4$ (420 nm) instead of TiO_2	0
4	*g*-C_3N_4 (420 nm) instead of TiO_2	trace
5	Mes-Acr-Ph (420 nm) instead of TiO_2	0
6	4CzIPN (420 nm) instead of TiO_2	0
7	$Ir(ppy)_3$ (420 nm) instead of TiO_2	0
8	DMSO instead of MeCN	35
9	THF instead of MeCN	42
10	CH_2Cl_2 instead of MeCN	32
11	MeOH instead of MeCN	0
12	no TiO_2	0
13	dark	0
14	no $MgSO_4$	66
15	TiO_2 (Antase)	51
16	TiO_2 (Rutile)	25

General procedure (A) for photocatalytic synthesis of *α*-substituted amines 5b-5aw, 6af-6al:

Imine **4** (0.5 mmol), carboxylic acid **3** (1.0 mmol, 2.0 equiv.), TiO_2 P25 (100 mg) were added to an oven dried 10 mL quartz reaction tube. The reaction tube was then evacuated and backfilled with N_2 three times, and 6 mL of degassed anhydrous MeCN was added. The reaction mixture was irradiated using Kessil lamp (PR-160, 390 nm) and cooled with a fan for 24-48 h. After reaction, the mixture was filtered through

diatomite and a small amount of silica gel. The filtrate was concentrated under reduced pressure. The sat. $NaHCO_3$ (10 mL) was added to residue and the mixture was extracted by CH_2Cl_2 (10 mL×3). Combined organic layers were dried over Na_2SO_4 and concentrated. The residue was purified by column chromatography on silica gel to furnish the corresponding products.

General procedure (B) for photocatalytic synthesis of *α*-substituted amines 6a-6ab, 6am-6ay:

Aldehyde **1** (0.2 mmol), amine **2** (0.2 mmol), benzyloxyacetic acid **3g** (66 mg, 0.4 mmol, 2.0 equiv.), TiO_2 P25 (40 mg), $MgSO_4$ (48 mg, 0.4 mmol, 2.0 equiv.) were added to an oven dried 10 mL quartz reaction tube. The reaction tube was then evacuated and backfilled with N_2 three times, and 4 mL of degassed anhydrous MeCN was added. The reaction mixture was irradiated using Kessil lamp (PR-160, 390 nm) and cooled with a fan for 24-48 h. After reaction, the mixture was filtered through diatomite and a small amount of silica gel. The filtrate was concentrated under reduced pressure. The sat. $NaHCO_3$ (10 mL) was added to residue and the mixture was extracted by CH_2Cl_2 (10 mL×3). Combined organic layers were dried over Na_2SO_4 and concentrated. The residue was purified by column chromatography on silica gel to furnish the corresponding products.

General procedure (C) for photocatalytic synthesis of α-substituted of glycine derivatives 6ac-6ae, 39-42:

Ethyl glyoxylate **1ad** (0.2 mL, ~50wt% in toluene), *p*-toluidine **2b** (22 mg, 0.2 mmol) or *p*-anisidine **2c** (25 mg, 0.2 mmol), acid **3** (0.4 mmol, 2.0 equiv.), TiO_2 P25 (40 mg), $MgSO_4$ (48 mg, 0.4 mmol, 2.0 equiv.) were added to an oven dried 10 mL quartz reaction tube. The reaction tube was then evacuated and backfilled with N_2 three times, and 4 mL of degassed anhydrous MeCN was added. The reaction mixture was irradiated using Kessil lamp (PR-160, 390 nm) and cooled with a fan for 48 h. After reaction, the mixture was filtered through diatomite and a small amount of silica gel. The filtrate was concentrated under reduced pressure. The sat. $NaHCO_3$ (10 mL) was added to residue and the mixture was extracted by CH_2Cl_2 (10 mL×3). Combined organic layers were dried over Na_2SO_4 and concentrated. The residue was purified by column chromatography on silica gel to furnish the corresponding products.

3. Characterization of products

***N*-(1-phenylethyl)aniline 5a**

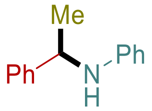

Imine **4a** (18 mg, 0.1 mmol), AcOH (0.2 mL), $Sc(OTf)_2$ (0.12 mmol, 59 mg, 1.2 eq.), TiO_2 P25 (40 mg) were added to an oven dried 10 mL quartz reaction tube. The reaction tube was then evacuated and backfilled with N_2 three times, and 3 mL of degassed anhydrous MeCN was added. The reaction mixture was irradiated using Kessil lamp (PR-160, 390 nm) and cooled with a fan for 48 h. The two parallel reactions were performed. After reaction, the two reaction mixtures were combined and filtered through diatomite and a small amount of silica gel. The filtrate was concentrated under reduced pressure. The sat. $NaHCO_3$ (10 mL) was added to residue and the mixture was extracted by CH_2Cl_2 (10 mL $\times$3). Combined organic layers were dried over Na_2SO_4 and concentrated. The residue was purified by column chromatography on silica gel to furnish the product **5a**[1] (27 mg, 68%), as a pale yellow oil. ^{1}H NMR (600 MHz, $CDCl_3$) δ 0.93 (t, J = 7.3 Hz, 3H), 1.31-1.37 (m, 1H), 1.40-1.46 (m, 1H), 1.70-1.81 (m, 2H), 4.30 (t, J = 6.8 Hz, 1H), 6.49-6.51 (m, 2H), 6.60-6.63 (m, 1H), 7.05-7.08 (m, 2H), 7.19-7.24 (m, 1H), 7.29-7.34 (m, 4H) ppm; ^{13}C NMR (150 MHz, $CDCl_3$) δ 13.9, 19.5, 41.1, 57.9, 113.2, 117.1, 126.3, 126.8, 128.5, 129.0, 144.3, 147.5 ppm.

***N*-(1-phenylhexyl)aniline 5b**

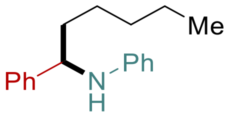

Following the **general procedure A**, 92 mg of **5b**[2] as a colorless oil was obtained (yield: 73%); ^{1}H NMR (600 MHz, $CDCl_3$) δ 0.86 (t, J = 7.1 Hz, 3H), 1.25-1.32 (m, 5H), 1.37-1.44 (m, 1H), 1.72-1.81 (m, 2H), 4.04 (br. s, 1H), 4.28 (t, J = 6.8 Hz, 1H), 6.49-6.51 (m, 2H), 6.60-6.63 (m, 1H), 7.05-7.08 (m, 2H), 7.19-7.23 (m, 1H), 7.28-7.34 (m, 4H) ppm; ^{13}C NMR (150 MHz, $CDCl_3$) δ 14.0, 22.5, 26.0, 31.7, 39.0, 58.2, 113.2, 117.0, 126.3, 126.8, 128.5, 129.1, 144.3, 147.5 ppm.

***N*-(3-cyclopentyl-1-phenylpropyl)aniline 5c**

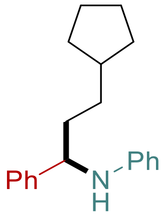

Following the **general procedure A**, 106 mg of **5c**[3] as a colorless oil was obtained (yield: 76%); ^{1}H NMR (600 MHz, $CDCl_3$) δ 1.00-1.06 (m, 2H), 1.29-1.35 (m, 1H), 1.39-1.44 (m, 1H), 1.46-1.51 (m, 2H), 1.55-1.59 (m, 2H), 1.71-1.81 (m, 5H), 4.04 (br. s, 1H), 4.27 (t, *J* = 6.8 Hz, 1H), 6.49-6.51 (m, 2H), 6.60-6.62 (m, 1H), 7.05-7.08 (m, 2H), 7.19-7.22 (m, 1H), 7.28-7.33 (m, 4H) ppm; ^{13}C NMR (150 MHz, $CDCl_3$) δ 25.1, 32.6, 32.7, 32.8, 38.1, 40.0, 58.4, 113.2, 117.0, 126.3, 126.8, 128.5, 129.0, 144.3, 147.5 ppm.

_N_-(1-phenylpent-4-en-1-yl)aniline 5d

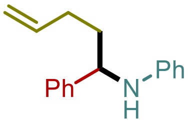

Following the **general procedure A**, 83 mg of **5d**[4] as a colorless oil was obtained (yield: 70%); ^{1}H NMR (600 MHz, $CDCl_3$) δ 1.76-1.87 (m, 2H), 2.01-2.12 (m, 2H), 4.01 (br. s, 1H), 4.25-4.28 (m, 1H), 4.92-4.97 (m, 2H), 5.72-5.79 (m, 1H), 6.43-6.44 (m, 2H), 6.54-6.56 (m, 1H), 6.99-7.01 (m, 2H), 7.13-7.17 (m, 1H), 7.22-7.27 (m, 4H) ppm; ^{13}C NMR (150 MHz, $CDCl_3$) δ 30.4, 37.8, 57.6, 113.2, 115.3, 117.2, 126.4, 26.9, 128.6, 129.1, 137.8, 143.9, 147.3 ppm.

_N_-(6-bromo-1-phenylhexyl)aniline 5e

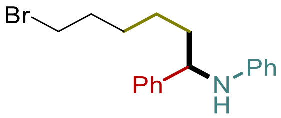

Following the **general procedure A**, 101 mg of **5e** as a colorless oil was obtained (yield: 61%); ^{1}H NMR (600 MHz, $CDCl_3$) δ 1.31-1.36 (m, 1H), 1.42-1.49 (m, 3H), 1.75-1.85 (m, 4H), 3.37 (t, *J* = 6.7 Hz, 2H), 4.30 (t, *J* = 6.8 Hz, 2H), 6.50-6.51 (m, 2H), 6.61-6.64 (m, 1H), 7.06-7.09 (m, 2H), 7.29-7.23 (m, 1H), 7.29-7.33 (m, 4H) ppm; ^{13}C NMR (150 MHz, $CDCl_3$) δ 25.5, 28.0, 32.6, 33.7, 38.6, 58.1, 113.2, 117.2, 126.3, 126.9, 128.5, 129.1, 143.9, 147.3 ppm; HRMS (ESI) *m/z* calcd for $[C_{18}H_{23}NBr]^+$ (M + H^+): 332.1008; found:332.1004.

Methyl-6-phenyl-6-(phenylamino)hexanoate 5f

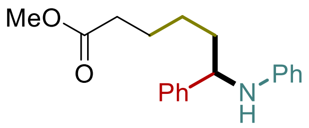

Following the **general procedure A**, 89 mg of **5f**[5] as a yellow oil was obtained (yield: 60%); ^{1}H NMR (600 MHz, $CDCl_3$) δ 1.31-1.38 (m, 1H), 1.41-1.49 (m, 1H), 1.61-1.67 (m, 2H), 1.74-1.85 (m, 2H), 2.29 (t, *J* = 7.4 Hz, 2H), 3.64 (s, 3H), 4.29 (t, *J* = 6.7 Hz, 2H), 4.06 (br. s, 1H), 6.49-6.51 (m, 2H), 6.61-6.63 (m, 1H), 7.06-7.08 (m, 2H), 7.19-7.22 (m, 1H), 7.29-7.33 (m, 4H) ppm; ^{13}C NMR (150 MHz, $CDCl_3$) δ 24.7, 25.8, 33.8, 51.5, 58.0, 113.2, 117.2, 126.3, 126.9, 128.5, 129.1, 143.9, 147.3, 174.0 ppm.

***N*-(2-butoxy-1-phenylethyl)aniline 5g**

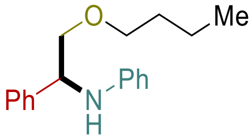

Following the **general procedure A**, 98 mg of **5g** as a colorless oil was obtained (yield: 73%); ^{1}H NMR (600 MHz, $CDCl_3$) δ 0.91 (t, *J* = 7.4 Hz, 3H), 1.34-1.40 (m, 2H), 1.54-1.58 (m, 2H), 3.40-3.43 (m, 1H), 3.49-3.53 (m, 2H), 3.65 (dd, *J* = 4.0, 10.2 Hz, 1H), 4.49 (dd, *J* = 4.0, 8.8 Hz, 1H), 4.62 (br. s, 1H), 6.52 (d, *J* = 7.8 Hz, 2H), 6.64-6.67 (m, 1H), 7.05-7.08 (m, 2H), 7.24-7.26 (m, 1H), 7.31-7.33 (m, 2H), 7.40-7.41 (m, 2H) ppm; ^{13}C NMR (150 MHz, $CDCl_3$) δ 13.9, 19.3, 31.6, 58.1, 70.7, 75.1, 114.0, 117.6, 126.7, 127.3, 128.6, 128.9, 140.9, 147.8 ppm; HRMS (ESI) *m/z* calcd for $[C_{18}H_{24}NO]^+$ (M + H^+): 270.1852; found: 270.1856.

***N*-(2-(Benzyloxy)-1-phenylethyl)aniline 5h**

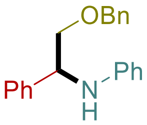

Following the **general procedure A**, 113 mg of **5h**[6] as a colorless oil was obtained (yield: 75%); ^{1}H NMR (600 MHz, $CDCl_3$) δ 3.58 (dd, *J* = 8.5, 9.9 Hz, 1H), 3.73 (dd, *J* = 4.1, 9.9 Hz, 1H), 4.51-4.58 (m, 3H), 4.62 (br. s, 1H), 6.51-6.52 (m, 2H), 6.64-6.67 (m, 1H), 7.05-7.08 (m, 2H), 7.22-7.35 (m, 8H), 7.39-7.40 (m, 2H) ppm; ^{13}C NMR (150 MHz, $CDCl_3$) δ 58.1, 72.9, 74.4, 113.9, 117.6, 126.8, 127.4, 127.7, 127.8, 128.5, 128.6, 129.0, 137.7, 140.6, 147.6 ppm.

2-Phenyl-2-(phenylamino)ethyl acetate 5i

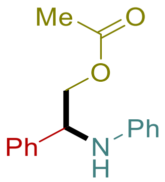

Following the **general procedure A**, 94 mg of **5i** as a colorless oil was obtained (yield: 74%); ^{1}H NMR (600 MHz, $CDCl_3$) δ 2.05 (s, 3H), 4.26-4.34 (m, 2H), 4.44 (br. s, 1H), 4.63 (dd, J = 4.9, 7.5 Hz, 1H), 6.52-6.54 (m, 2H), 6.65-6.69 (m, 1H), 7.07-7.11 (m, 2H), 7.23-7.28 (m, 1H), 7.32-7.35 (m, 2H), 7.39-7.41 (m, 2H) ppm; ^{13}C NMR (150 MHz, $CDCl_3$) δ 20.9, 57.8, 67.8, 113.5, 117.8, 126.7, 127.8, 128.8, 129.1, 139.5, 146.9, 171.2 ppm; HRMS (ESI) *m/z* calcd for $[C_{16}H_{18}NO_2]^+$ (M + H$^+$): 256.1332; found: 256.1346.

***N*-(2-(2-chlorophenoxy)-1-phenylethyl)aniline 5j**

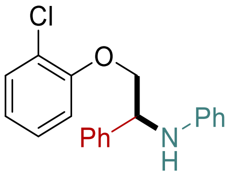

Following the **general procedure A**, 110 mg of **5j** as a colorless oil was obtained (yield: 68%); ^{1}H NMR (600 MHz, $CDCl_3$) δ 4.09 (dd, J = 8.0, 9.5 Hz, 1H), 4.30 (dd, J = 4.0, 9.5 Hz, 1H), 4.75 (dd, J = 4.0, 8.0 Hz, 1H), 4.85 (br. s, 1H), 6.58-6.59 (m, 2H), 6.68-6.70 (m, 1H), 6.87-6.92 (m, 2H), 7.08-7.11 (m, 2H), 7.15-7.18 (m, 1H), 7.27-7.30 (m, 1H), 7.35-7.37 (m, 3H), 7.50-7.51 (m, 2H) ppm; ^{13}C NMR (150 MHz, $CDCl_3$) δ 57.9, 73.2, 114.1, 114.2, 118.0, 122.2, 123.4, 127.0, 127.7, 127.8, 128.8, 129.0, 130.4, 139.9, 147.4, 153.8 ppm; HRMS (ESI) *m/z* calcd for $[C_{20}H_{19}ClNO]^+$ (M + H$^+$):324.1150; found: 324.1152.

***N*-(2-(((1R,2S,5R)-2-isopropyl-5-methylcyclohexyl)oxy)ethyl)-4-methoxyaniline 5k**

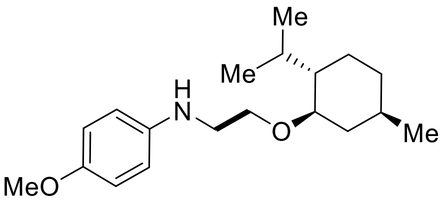

Following the **general procedure A**, 107 mg of **5k** as a white solid was obtained (yield: 70%); ^{1}H NMR (600 MHz, $CDCl_3$) δ 0.78 (d, J = 7.0 Hz, 3H), 0.82-1.00 (m, 9H), 1.12-1.27 (m, 1H), 1.31-1.37 (m, 1H), 1.60-1.67 (m, 2H), 2.07-2.10 (m, 1H), 2.18-2.23 (m, 1H), 3.04-3.08 (m, 1H), 3.18-3.26 (m, 2H), 3.48-3.52 (m, 1H), 3.74 (s, 3H), 3.79-3.82 (m, 1H), 6.59-6.61 (m, 2H), 6.76-6.79 (m, 2H) ppm; ^{13}C NMR (150 MHz, $CDCl_3$) δ 16.2, 20.9, 22.3, 23.3, 25.7, 31.5, 34.5, 40.4, 45.2, 48.3, 55.8, 66.7, 79.4, 114.6, 114.8, 142.5, 152.2 ppm; HRMS (ESI) m/z calcd for $[C_{19}H_{31}NO_2Na]^+$ (M + Na^+): 328.2247; found: 328.2244.

_N_-(2-phenyl-2-(phenylamino)ethyl)acetamide 5l

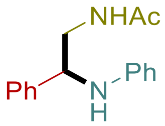

Following the **general procedure A**, 97 mg of **5l**[7] as a white solid was obtained (yield: 76%); ^{1}H NMR (600 MHz, $CDCl_3$) δ 1.96 (s, 3H), 3.54-3.61 (m, 2H), 4.45 (dd, J = 4.6, 7.7 Hz, 1H), 5.9 (br.s, 1H), 6.50 (d, J = 7.8 Hz, 2H), 6.62-6.64 (m, 1H), 7.06-7.08 (m, 2H), 7.25-7.27 (m, 1H), 7.32-7.37 (m, 4H) ppm; ^{13}C NMR (150 MHz, $CDCl_3$) δ 23.2, 46.2, 59.5, 113.2, 117.3, 126.5, 127.5, 128.8, 129.1, 140.9, 147.2, 171.6 ppm.

***tert*-Butyl (2-phenyl-2-(phenylamino)ethyl)carbamate 5m**

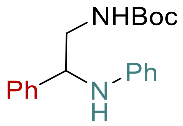

Following the **general procedure A**, 145 mg of **5m**[8] as a white solid was obtained (yield: 93%); ^{1}H NMR (600 MHz, $CDCl_3$) δ 1.46 (s, 9H), 3.40-3.44 (m, 1H), 3.46-3.51 (m, 1H), 4.41-4.43 (m, 1H), 4.81 (s, 1H), 4.95 (s, 1H), 6.48-6.50 (m, 2H), 6.62-6.64 (m, 1H), 7.06-7.09 (m, 2H), 7.24-7.27 (m 1H), 7.32-7.38 (m, 4H) ppm; ^{13}C NMR (150 MHz, $CDCl_3$) δ 28.3, 47.2, 60.0, 79.9, 113.2, 117.2, 126.5, 127.5, 128.8, 129.1, 141.1, 147.4, 157.1 ppm.

Benzyl (2-phenyl-2-(phenylamino)ethyl)carbamate 5n

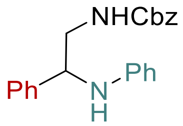

Following the **general procedure A**, 151 mg of **5n**[8] as a white solid was obtained (yield: 87%); ^{1}H NMR (600 MHz, $CDCl_3$) δ 3.52-3.54 (m, 2H), 4.46-4.48 (m, 1H), 4.73 (s, 1H), 5.01 (s, 1H), 5.10-5.15 (m, 2H), 6.49-6.50 (m, 2H), 6.63-6.66 (m, 1H), 7.06-7.09 (m, 2H), 7.24-7.26 (m 1H), 7.31-7.34 (m, 9H) ppm; ^{13}C NMR (150 MHz, $CDCl_3$) δ 47.5, 59.3, 67.0, 113.3, 117.4, 126.5, 127.6, 128.1, 128.2, 128.5, 128.8, 129.1, 136.2, 140.8, 147.1, 157.4 ppm.

2-(2-phenyl-2-(phenylamino)ethyl)isoindoline-1,3-dione 5o

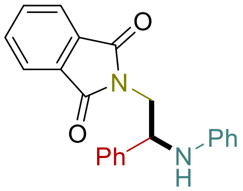

Following the **general procedure A**, 118 mg of **5o** as a white solid was obtained (yield: 69%); ^{1}H NMR (600 MHz, $CDCl_3$) δ 3.97 (dd, J = 9.7, 14.4 Hz, 1H), 4.01 (dd, J = 4.1, 14.4 Hz, 1H), 4.72-4.74 (m, 1H), 4.88 (s, 1H), 6.48 (d, J = 7.7 Hz, 2H), 6.57-6.59 (m, 1H), 7.01-7.04 (m, 2H), 7.27-7.29 (m, 1H), 7.35-7.37 (m, 2H), 7.49-7.50 (m, 2H), 7.71-7.72 (m, 2H), 7.84-7.85 (m, 2H) ppm; ^{13}C NMR (150 MHz, $CDCl_3$) δ 44.4, 58.2, 113.1, 117.3, 123.5, 126.5, 127.8, 128.9, 129.0, 131.8, 134.2, 140.3, 146.7, 168.8 ppm; HRMS (ESI) *m/z* calcd for $[C_{22}H_{19}N_2O_2]^+$ (M + H^+): 343.1441; found: 343.1443.

***N*-(2-(1-methyl-1H-indol-3-yl)-1-phenylethyl)aniline 5p**

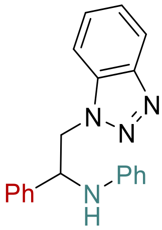

Following the **general procedure A**, 115 mg of **5p**[9] as a colorless oil was obtained (yield: 71%); ^{1}H NMR (600 MHz, $CDCl_3$) δ 4.82 (br. s, 1H), 4.90-4.92 (m, 1H), 4.95-4.96 (m, 2H), 6.50-6.52 (m, 2H), 6.63-6.65 (m, 1H), 7.03-7.06 (m, 2H), 7.10-7.11 (m, 1H), 7.23-7.28 (m, 3H), 7.29-7.36 (m, 4H), 8.01-8.03 (m, 1H) ppm; ^{13}C NMR (150 MHz, $CDCl_3$) δ 54.0, 58.2, 108.8, 113.6, 118.1, 120.0, 123.9, 126.4, 127.5, 128.1, 129.1, 133.3, 139.7, 145.7, 146.2 ppm.

***N*-(2-(2-bromophenyl)-1-phenylethyl)aniline 5q**

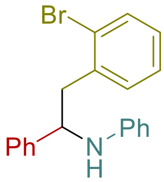

Following the **general procedure A**, 140 mg of **5q**[10] as a colorless oil was obtained (yield: 80%); ^{1}H NMR (600 MHz, $CDCl_3$) δ 3.15-3.21 (m, 2H), 4.70 (dd, *J* = 6.1, 8.2 Hz, 1H), 6.46-6.47 (m, 2H), 6.59-6.62 (m, 1H), 7.02-7.04 (m, 2H), 7.05-7.09 (m, 2H), 7.16-7.18 (m, 1H), 7.22-7.24 (m, 1H), 7.29-7.31 (m, 2H), 7.37-7.38 (m, 2H), 7.54-7.56 (m, 1H) ppm; ^{13}C NMR (150 MHz, $CDCl_3$) δ 45.2, 58.1, 113.5, 117.4, 124.9, 126.3, 127.1, 127.4, 128.4, 128.6, 129.0, 131.3, 133.0, 137.6, 143.3, 147.1 ppm.

***N*-(1-phenyl-2-(m-tolyl)ethyl)aniline 5r**

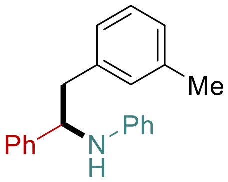

Following the **general procedure A**, 115 mg of **5r**[11] as a colorless oil was obtained (yield: 80%); ^{1}H NMR (600 MHz, $CDCl_3$) δ 2.30 (s, 3H), 2.93 (dd, *J* = 8.5, 14.0 Hz, 1H), 3.10 (dd, *J* = 5.5, 14.0 Hz, 1H), 4.11 (br. s, 1H), 4.55 (dd, *J* = 5.5, 8.5 Hz, 1H), 6.43-6.45 (m, 2H), 6.60-6.62 (m, 1H), 6.92-6.94 (m, 2H), 7.02-7.05 (m, 3H), 7.14-7.17 (m, 1H), 7.22-7.24 (m, 1H), 7.29-7.34 (m, 4H) ppm; ^{13}C NMR (150 MHz, $CDCl_3$) δ 21.4, 45.2, 59.2, 113.6, 117.4, 126.1, 126.4, 127.0, 127.5, 128.4, 128.5, 128.9, 130.0, 137.6, 138.1, 143.6, 147.3 ppm.

***N*-(2-(3-chlorophenyl)-1-phenylethyl)aniline 5s**

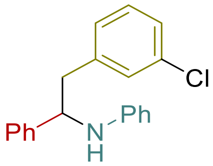

Following the **general procedure A**, 112 mg of **5s**[11] as a colorless oil was obtained (yield: 73%); ^{1}H NMR (600 MHz, $CDCl_3$) δ 3.00 (dd, *J* = 8.0, 14.0 Hz, 1H), 3.08 (dd, *J* = 6.0, 14.0 Hz, 1H), 4.06 (br.s, 1H), 4.57 (dd, *J* = 6.0, 8.0 Hz, 1H), 6.46-6.48 (m, 2H), 6.62-6.65 (m, 1H), 6.96-6.98 (m, 1H), 7.04-7.07 (m, 2H), 7.10 (s, 1H), 7.16-7.19 (m, 2H), 7.22-7.25 (m, 1H), 7.28-7.31 (m, 3H) ppm; ^{13}C NMR (150 MHz, $CDCl_3$) δ

44.6, 59.1, 113.6, 117.6, 126.4, 126.9, 127.2, 127.3, 128.6, 129.0, 129.1, 129.3, 129.7, 134.2, 139.7, 142.8, 146.9 ppm.

_N_-(2-([1,1'-biphenyl]-4-yl)-1-phenylethyl)aniline 5t

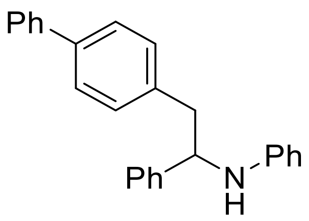

Following the **general procedure A**, 119 mg of **5t**[11] as a white solid was obtained (yield: 68%); ^{1}H NMR (600 MHz, $CDCl_3$) δ 3.05 (dd, J = 8.3, 14.0 Hz, 1H), 3.16 (dd, J = 5.7, 14.0 Hz, 1H), 4.14 (br. s, 1H), 4.62 (dd, J = 5.7, 8.3 Hz, 1H), 6.47 (d, J = 8.0 Hz, 2H), 6.61-6.64 (m, 1H), 7.04-7.06 (m, 2H), 7.17-7.19 (m, 2H), 7.22-7.25 (m, 1H), 7.29-7.35 (m, 5H), 7.41-7.43 (m, 2H), 7.49-7.50 (m, 2H), 7.56-7.57 (m, 2H) ppm; ^{13}C NMR (150 MHz, $CDCl_3$) δ 44.7, 59.1, 113.6, 117.5, 126.4, 127.0, 127.1, 127.2, 128.6, 128.7, 129.0, 129.6, 136.7, 139.6, 140.7, 143.4, 147.2 ppm.

_N_-(2-(4-(methylthio)phenyl)-1-phenylethyl)aniline 5u

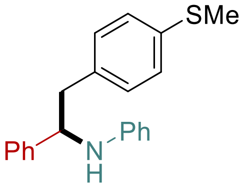

Following the **general procedure A**, 89 mg of **5u**[12] as a colorless oil was obtained (yield: 56%); ^{1}H NMR (600 MHz, $CDCl_3$) δ 2.45 (s, 3H), 2.98 (dd, J = 8.0, 14.1 Hz, 1H), 3.08 (dd, J = 5.8, 14.1 Hz, 1H), 4.55 (dd, J = 5.8, 8.0 Hz, 1H), 6.45-6.47 (m, 2H), 6.61-6.64 (m, 1H), 7.01-7.06 (m, 4H), 7.15-7.16 (m, 2H), 7.21-7.24 (m, 1H), 7.28-7.31 (m, 3H) ppm; ^{13}C NMR (150 MHz, $CDCl_3$) δ 15.9, 44.5, 59.1, 113.6, 117.5, 126.4, 126.8, 17.1, 128.6, 129.0, 129.7, 134.5, 136.5, 143.2, 147.1 ppm.

4-(2-phenyl-2-(phenylamino)ethyl)benzonitrile 5v

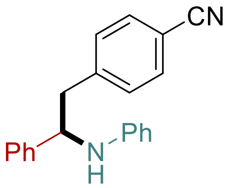

Following the **general procedure A**, 110 mg of **5v**[10] as a colorless oil was obtained (yield: 74%); ^{1}H NMR (600 MHz, $CDCl_3$) δ 3.04 (m, 2H), 3.98 (br. s, 1H), 4.52-4.55

(m, 1H), 6.42 (d, *J* = 7.8 Hz, 2H), 6.57-6.59 (m, 1H), 6.99-7.01 (m, 2H), 7.08 (d, *J* = 7.8 Hz, 2H), 7.15-7.17 (m, 3H), 7.20-7.22 (m, 2H), 7.43-7.45 (m, 2H) ppm; ^{13}C NMR (150 MHz, $CDCl_3$) δ 44.7, 58.9, 110.5, 113.5, 117.9, 118.8, 126.4, 127.4, 128.7, 129.1, 130.0, 132.1, 142.2, 143.3, 146.7 ppm.

_N_-(4-(2-phenyl-2-(phenylamino)ethyl)phenyl)acetamide 5w

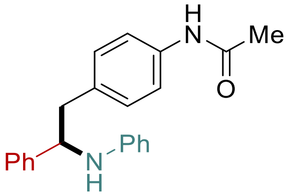

Following the **general procedure A**, 140 mg of **5w** as a white solid was obtained (yield: 85%); ^{1}H NMR (600 MHz, $CDCl_3$) δ 2.13 (s, 3H), 2.98 (dd, *J* = 8.0, 14.1 Hz, 1H), 3.07 (dd, *J* = 5.8, 14.0 Hz, 1H), 4.54 (dd, *J* = 5.8, 8.0 Hz, 1H), 6.45 (d, *J* = 7.8 Hz, 1H), 6.60-6.63 (m, 1H), 7.03-7.05 (m, 4H), 7.20-7.23 (m, 1H), 7.27-7.30 (m, 4H), 7.38-7.39 (m, 3H, Ar*H* and N*H*) ppm; ^{13}C NMR (150 MHz, $CDCl_3$) δ 24.5, 44.4, 59.1, 113.6, 117.4, 119.9, 126.4, 127.0, 128.5, 129.0, 129.7, 133.5, 136.5, 143.2, 147.1, 168.4 ppm; HRMS (ESI) *m/z* calcd for $[C_{22}H_2N_2ONa]^+$ (M + Na^+): 353.1624; found: 353.1628.

_N_-(2-(3,4-dimethoxyphenyl)-1-phenylethyl)aniline 5x

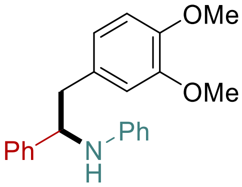

Following the **general procedure A**, 110 mg of **5x** as a white solid was obtained (yield: 66%); ^{1}H NMR (600 MHz, $CDCl_3$) δ 2.98 (dd, *J* = 7.6, 13.9 Hz, 1H), 3.07 (dd, *J* = 5.9, 13.9 Hz, 1H), 3.71 (s, 3H), 3.85 (s, 3H), 4.55 (br. t, *J* = 6.8 Hz, 1H), 6.44 (s, 1H), 6.46-6.47 (m, 2H), 6.61-6.64 (m, 1H), 6.68-6.70 (m, 1H), 6.77-6.78 (m, 1H), 7.04-7.06 (m, 2H), 7.21-7.24 (m, 1H), 7.29-7.30 (m, 4H) ppm; ^{13}C NMR (150 MHz, $CDCl_3$) δ 44.6, 55.7, 55.8, 59.1, 111.0, 112.4, 113.6, 117.5, 121.3, 126.6, 127.0, 128.5, 129.0, 129.9, 143.3, 147.2, 147.8, 148.7 ppm; HRMS (ESI) *m/z* calcd for $[C_{22}H_{24}NO_2]^+$ (M + H^+): 334.1802; found: 334.1804.

_N_-(2-(4-bromo-2-methoxyphenyl)-1-phenylethyl)aniline 5y

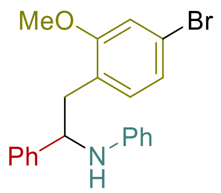

Following the **general procedure A**, 122 mg of **5y** as a colorless oil was obtained (yield: 64%); ^{1}H NMR (600 MHz, $CDCl_3$) δ 2.87 (dd, J = 5.0, 13.8 Hz, 1H), 2.98 (dd, J = 8.6, 13.8 Hz, 1H), 3.78 (s, 3H), 4.45 (dd, J = 5.0, 8.6 Hz, 1H), 6.34-6.35 (m, 2H), 6.50-6.53 (m, 1H), 6.74-6.75 (m, 1H), 6.88-6.89 (m, 1H), 6.91 (s, 1H), 6.94-6.97 (m, 2H), 7.13-7.16 (m, 1H), 7.20-7.23 (m, 2H), 7.24-7.26 (m, 2H) ppm; ^{13}C NMR (150 MHz, $CDCl_3$) δ 39.3, 55.6, 58.9, 113.2, 114.1, 117.0, 120.9, 123.6, 125.8, 126.3, 127.0, 128.5, 129.0, 32.1, 143.7, 147.4, 158.2 ppm; HRMS (ESI) *m/z* calcd for $[C_{21}H_{21}BrN_2]^+$ (M + H^+): 382.0801; found: 382.0802.

_N_- (2-(2-chloro-3-fluorophenyl)-1-phenylethyl)aniline 5z

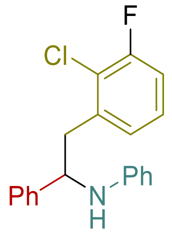

Following the **general procedure A**, 99 mg of **5z** as a colorless oil was obtained (yield: 61%); ^{1}H NMR (600 MHz, $CDCl_3$) δ 3.10-3.16 (m, 2H), 4.60-4.63 (m, 1H), 4.15 (br. s, 1H), 6.39-6.40 (m, 2H), 6.53-6.56 (m, 1H), 6.80-6.81 (m, 1H), 6.92-7.03 (m, 4H), 7.14-7.17 (m, 1H), 7.21-7.24 (m, 2H), 7.27-7.28 (m, 2H) ppm; ^{13}C NMR (150 MHz, $CDCl_3$) δ 42.4 (2C), 57.9, 113.5, 114.8, 114.9, 117.6, 121.3, 121.4, 126.2, 126.333, 127.2, 127.3, 128.7, 129.0, 138.4, 143.0, 147.0, 157.6, 159.2 ppm; HRMS (ESI) *m/z* calcd for $[C_{20}H_{18}ClFN]^+$ (M + H^+): 326.1106; found: 326.1109.

_N_-(1-phenyl-2-(pyridin-3-yl)ethyl)aniline 5aa

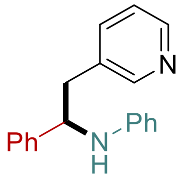

Following the **general procedure A**, 96 mg of **5aa**[10] as a colorless oil was obtained (yield: 70%); ^{1}H NMR (600 MHz, $CDCl_3$) δ 3.06-3.02 (m, 2H), 4.12 (br. s, 1H), 4.60 (br. t, J = 6.9 Hz, 1H), 6.49-6.50 (m, 2H), 6.32-6.66 (m, 1H), 7.06-7.08 (m, 2H), 7.14-7.16 (m, 1H), 7.22-7.26 (m, 3H), 7.28-7.30 (m, 2H), 7.32-7.34 (m, 1H), 8.34 (d,

J = 1.9 Hz, 1H), 8.45 (dd, J = 1.9, 4.7 Hz, 1H) ppm; ^{13}C NMR (150 MHz, $CDCl_3$) δ 41.8, 58.9, 113.5, 117.7, 123.3, 126.4, 127.4, 128.7, 129.1, 133.2, 136.6, 142.3, 146.7, 148.1, 150.5 ppm.

***N*-(1-phenyl-2-(quinolin-6-yl)ethyl)aniline 5ab**

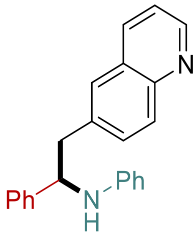

Following the **general procedure A**, 84 mg of **5ab** as a white solid was obtained (yield: 52%); ^{1}H NMR (600 MHz, $CDCl_3$) δ 3.24 (dd, J = 7.8, 14.0 Hz, 1H), 3.30 (dd, J = 6.2, 14.0 Hz, 1H), 4.18 (br. s, 1H), 4.72 (dd, J = 6.2, 7.8 Hz, 1H), 6.47-6.49 (m, 2H), 6.62-6.64 (m, 1H), 7.04-7.06 (m, 2H), 7.22-7.25 (m, 1H), 7.28-7.32 (m, 4H), 7.37 (dd, J = 4.2, 8.3 Hz, 1H), 7.45 (dd, J = 1.9, 8.6 Hz, 1H), 7.52 (s, 1H), 8.01 (d, J = 8.6 Hz, 1H), 8.05 (d, J = 8.3 Hz, 1H), 8.87 (dd, J = 1.6, 4.2 Hz, 1H) ppm; ^{13}C NMR (150 MHz, $CDCl_3$) δ 44.9, 59.0, 113.6, 117.6, 121.3, 126.4, 127.2, 127.6, 128.2, 128.6, 129.0, 129.5, 131.1, 135.7, 136.2, 142.9, 147.0, 147.3, 150.0, 150.1 ppm; HRMS (ESI) *m/z* calcd for $[C_{23}H_{21}N_2]^+$ ($M + H^+$): 325.1699; found: 325.1704.

***N*-(2-(furan-2-yl)-1-phenylethyl)aniline 5ac**

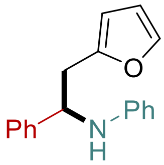

Following the **general procedure A**, 93 mg of **5ac**[10] as a colorless oil was obtained (yield: 71%); ^{1}H NMR (600 MHz, $CDCl_3$) δ 2.95 (dd, J = 8.5, 15.2 Hz, 1H), 3.04 (dd, J = 5.0, 15.2 Hz, 1H), 4.20 (br. s, 1H), 4.55 (dd, J = 5.0, 8.5 Hz, 1H), 5.93-5.94 (m, 1H), 6.18-6.19 (m, 1H), 6.41 (d, J = 8.3 Hz, 2H), 6.55-6.57 (m, 1H), 6.97-7.00 (m, 2H), 7.14-7.16 (m, 1H), 7.21-7.27 (m, 5H) ppm; ^{13}C NMR (150 MHz, $CDCl_3$) δ 37.3, 57.5, 107.4, 110.3, 113.6, 117.5, 126.2, 127.2, 128.6, 129.0, 141.8, 143.1, 147.2, 152.0 ppm.

***N*-(1-phenyl-2-(thiophen-2-yl)ethyl)aniline 5ad**

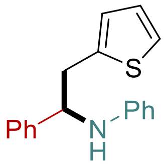

Following the **general procedure A**, 102 mg of **5ad** as a colorless oil was obtained (yield: 73%); ^{1}H NMR (600 MHz, $CDCl_3$) δ 3.24 (dd, J = 8.2, 15.1 Hz, 1H), 3.32 (dd, J = 5.4, 15.1 Hz, 1H), 4.16 (br. s, 1H), 4.59 (dd, J = 5.4, 8.2 Hz, 1H), 6.48-6.49 (m, 2H), 6.63-6.65 (m, 1H), 6.77-6.78 (m, 1H), 6.90-6.91 (m, 1H), 7.05-7.08 (m, 2H), 7.13-7.14 (m, 1H), 7.22-7.26 (m, 1H), 7.30-7.33 (m, 2H), 7.35-7.36 (m, 2H) ppm; ^{13}C NMR (150 MHz, $CDCl_3$) δ 38.9, 59.3, 113.6, 117.6, 124.4, 126.2, 126.4, 126.8, 127.3, 128.6, 129.0, 140.0, 142.9, 147.1 ppm; HRMS (ESI) *m/z* calcd for $[C_{18}H_{18}NS]^+$ (M + H^+): 280.1154; found: 280.1156.

***N*- (2-(benzo[b]thiophen-2-yl)-1-phenylethyl)aniline 5ae**

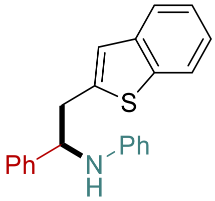

Following the **general procedure A**, 110 mg of **5ae** as a colorless oil was obtained (yield: 67%); ^{1}H NMR (600 MHz, $CDCl_3$) δ 3.32 (dd, J = 8.2, 15.1 Hz, 1H), 3.40 (dd, J = 5.2, 15.1 Hz, 1H), 4.21 (br. s, 1H), 4.70 (dd, J = 5.2, 8.2 Hz, 1H), 6.50-6.51 (m, 2H), 6.64-6.66 (m, 1H), 7.01 (s, 1H), 7.05-7.08 (m, 2H), 7.24-7.27 (m, 2H), 7.29-7.36 (m, 3H), 7.38-7.39 (m, 2H), 7.65 (d, J = 7.8 Hz, 1H), 7.73 (d, J = 8.0 Hz, 1H) ppm; ^{13}C NMR (150 MHz, $CDCl_3$) δ 39.8, 58.8, 113.7, 117.8, 122.2, 122.8, 123.0, 123.9, 124.2, 126.4, 127.4, 128.7, 129.1, 139.7, 141.2, 142.7, 146.9 ppm; HRMS (ESI) *m/z* calcd for $[C_{22}H_{20}NS]^+$ (M + H^+): 330.1311; found: 330.1308.

***N*-(2-(1-methyl-1H-indol-3-yl)-1-phenylethyl)aniline 5af**

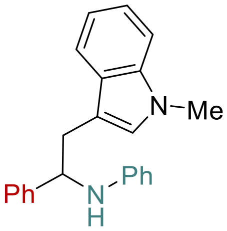

Following the **general procedure A**, 103 mg of **5af** as a colorless oil was obtained (yield: 63%); ^{1}H NMR (600 MHz, $CDCl_3$) δ 3.13 (dd, J = 8.5, 14.9 Hz, 1H), 3.27 (dd,

J = 4.9, 14.9 Hz, 1H), 4.25 (br. s, 1H), 4.66 (dd, J = 4.9, 8.5 Hz, 1H), 6.42-6.44 (m, 2H), 6.58-6.60 (m, 1H), 6.75 (s, 1H), 7.00-7.03 (m, 2H), 7.08-7.11 (m, 1H), 7.21-7.25 (m, 2H), 7.27-7.32 (m, 3H), 7.38-7.39 (m, 2H), 7.52-7.53 (m, 1H) ppm; ^{13}C NMR (150 MHz, $CDCl_3$) δ 32.6, 35.0, 58.3, 109.3, 110.4, 113.6, 117.2, 118.7, 119.0, 121.7, 126.4, 126.9, 127.3, 128.1, 128.5, 128.9, 137.0, 144.2, 147.5 ppm; HRMS (ESI) *m/z* calcd for $[C_{23}H_{22}N_2Na]^+$ (M + Na^+): 349.1675; found: 349.1673.

***N*-phenyl(-tetrahydrofuran-2-yl)methyl)aniline 5ag**

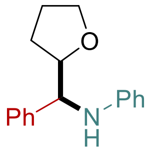

Following the **general procedure A**, 82 mg of **5ag**[13] as a colorless oil was obtained (yield: 65%); a separable diastereomeric amine (*dr* = 1:1); A: ^{1}H NMR (600 MHz, $CDCl_3$) δ 1.76-1.94 (m, 4H), 3.78-3.82 (m, 1H), 3.90-3.94 (m, 1H), 4.02-4.05 (m, 1H), 4.21 (d, J = 6.4 Hz, 1H), 4.76 (br. s, 1H), 6.51-6.53 (m, 2H), 6.61-6.64 (m, 1H), 7.04-7.06 (m, 2H), 7.22-7.25 (m, 1H), 7.30-7.32 (m, 2H), 7.40-7.41 (m, 2H) ppm; ^{13}C NMR (150 MHz, $CDCl_3$) δ 25.7, 28.8, 62.0, 68.6, 83.0, 113.8, 117.4, 127.2, 127.3, 128.6, 129.0, 141.6, 147.8 ppm.

B: ^{1}H NMR (600 MHz, $CDCl_3$) δ 1.56-1.60 (m, 1H), 1.72-1.80 (m, 3H), 3.73-3.81 (m, 2H), 4.23-4.26 (m, 1H), 4.42 (d, J = 4.3 Hz, 1H), 4.57 (br. s, 1H), 6.51-6.53 (m, 2H), 6.61-6.63 (m, 1H), 7.04-7.06 (m, 2H), 7.21-7.24 (m, 1H), 7.28-7.30 (m, 2H), 7.37-7.38 (m, 2H) ppm; ^{13}C NMR (150 MHz, $CDCl_3$) δ 25.5, 27.2, 60.7, 68.7, 82.0, 113.8, 117.4, 127.2, 127.7, 128.2, 128.9, 140.0, 147.3 ppm.

***N*-(cyclohexyl(phenyl)methyl)aniline 5ah**

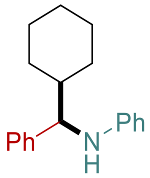

Following the **general procedure A**, 93 mg of **5ah**[14] as a colorless oil was obtained (yield: 70%); ^{1}H NMR (600 MHz, $CDCl_3$) δ 1.01-1.23 (m, 5H), 1.51-1.54 (m, 1H), 1.62-1.67 (m, 2H), 1.69-1.76 (m, 2H), 1.87-1.89 (m, 1H), 4.10 (d, J = 6.3 Hz, 1H), 4.13 (br. s, 1H), 6.48-6.49 (m, 2H), 6.58-6.60 (m, 1H), 7.03-7.06 (m, 2H), 7.18-7.21

(m, 1H), 7.27-7.28 (m, 4H) ppm; ^{13}C NMR (150 MHz, CDCl$_3$) δ 26.3 (2C), 26.4, 29.4, 30.2, 44.9, 63.3, 113.1, 116.8, 126.7, 127.2, 128.1, 129.0, 142.6, 147.7 ppm.

_N_ -(cycloheptyl(phenyl)methyl)aniline 5ai

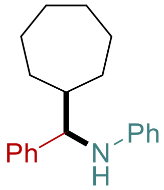

Following the **general procedure A**, 99 mg of **5ai**[5] as a colorless oil was obtained (yield: 71%); ^{1}H NMR (600 MHz, CDCl$_3$) δ 1.32-1.37 (m, 3H), 1.42-1.48 (m, 3H), 1.52-1.55 (m, 2H), 1.67-1.68 (m, 3H), 1.75-1.77 (m, 1H), 1.88-1.90 (m, 1H), 4.08 (br. s, 1H), 4.22 (d, J = 5.5 Hz, 1H), 6.47-6.48 (m, 2H), 6.60-6.61 (m, 1H), 7.04-7.07 (m, 2H), 7.18-7.20 (m, 1H), 7.26-7.31 (m, 4H) ppm; ^{13}C NMR (150 MHz, CDCl$_3$) δ 26.9, 27.0, 28.0, 28.2, 29.4, 32.2, 46.4, 63.7, 113.1, 116.9, 126.6, 127.1, 128.2, 129.0, 142.8, 147.8 ppm.

_N_-(1,2,2-triphenylethyl)aniline 5aj

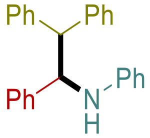

Following the **general procedure A**, 143 mg of **5aj**[10] as a colorless oil was obtained (yield: 82%); ^{1}H NMR (600 MHz, CDCl$_3$) δ 4.16 (br.s, 1H), 4.23 (d, J = 8.6 Hz, 1H), 4.98 (d, J = 8.6 Hz, 1H), 6.43 (d, J = 7.7 Hz, 2H), 6.61-6.64 (m, 1H), 7.02-7.05 (m, 4H), 7.08-7.15 (m, 8H), 7.22-7.24 (m, 3H), 7.28-7.31 (m, 2H) ppm; ^{13}C NMR (150 MHz, CDCl$_3$) δ 59.5, 61.3, 113.6, 117.5, 125.8, 126.4, 126.9, 127.4, 128.0, 128.1, 128.2, 128.5, 128.7, 129.0, 140.5, 141.9, 142.3, 147.1 ppm.

4-Methoxy-_N_-(((3aR,4R,6R,6aR)-6-methoxy-2,2-dimethyltetrahydrofuro[3,4-d][1,3]dioxol-4-yl)methyl)aniline 5ak

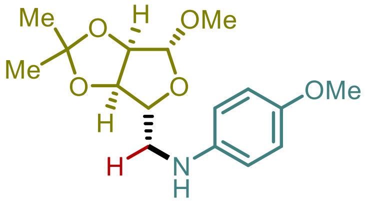

Following the **general procedure A**, 91 mg of **5ak** as a colorless oil was obtained (yield: 59%); ^{1}H NMR (600 MHz, CDCl$_3$) δ 1.32 (s, 3H), 1.49 (s, 3H), 3.16 (dd, J =

7.1, 12.4 Hz, 1H), 3.22 (dd, J = 5.5, 12.4 Hz, 1H), 3.36 (s, 3H), 3.74 (s, 3H), 4.45 (br. t, J = 6.2 Hz, 1H), 4.63 (d, J = 6.0 Hz, 1H), 4.71 (d, J = 6.0 Hz, 1H), 5.01 (s, 1H), 6.60 (d, J = 8.9 Hz, 2H), 6.78 (d, J = 8.9 Hz, 2H) ppm; ^{13}C NMR (150 MHz, $CDCl_3$) δ 24.9, 26.4, 48.1, 55.2, 55.7, 82.5, 85.5, 85.8, 109.8, 1112.4, 114.4, 114.9, 142.0, 152.4 ppm. HRMS (ESI) *m/z* calcd for $[C_{16}H_{24}NO_5]^+$ (M + H^+): 310.1649; found: 310.1651.

***N*-(2-(4-isobutylphenyl)-1-phenylpropyl)aniline 5al**

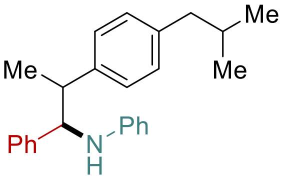

Following the **general procedure A**, 130 mg of **5al,** as an inseparable diastereomeric mixture (*dr* = 1:1), a colorless oil was obtained (yield: 76%); ^{1}H NMR (600 MHz, $CDCl_3$) δ 0.87-0.90 (m, 12H), 1.15 (d, J = 6.9 Hz, 3H), 1.31 (d, J = 6.9 Hz, 3H), 1.80-1.87 (m, 2H), 2.41 (d, J = 7.1, 2H), 2.44 (d, J = 7.1, 2H), 2.94-3.00 (m, 1H), 3.17-3.22 (m, 1H), 4.02 (br. s, 1H), 4.17 (br. s, 1H), 4.30 (d, J = 8.3 Hz, 1H), 4.49 (d, J = 5.3 Hz, 1H), 6.34-6.36 (m, 2H), 6.44-6.45 (m, 2H), 6.55-6.60 (m, 2H), 6.96-7.04 (m, 9H), 7.06-7.12 (m, 4H), 7.15-7.24 (m, 5H), 7.29-7.34 (m, 4H) ppm; ^{13}C NMR (150 MHz, $CDCl_3$) δ 16.1, 19.5, 22.3 (2C), 22.4 (2C), 30.2, 45.0 (2C), 45.4, 47.0, 63.2, 64.0, 113.5, 117.2 (2C), 126.7, 127.0, 127.4 (2C), 127.5, 127.7, 127.9, 128.3, 128.8, 128.9, 129.0, 129.4, 139.9, 140.0, 140.3, 141.6, 142.9, 147.3, 147.7 ppm; HRMS (ESI) *m/z* calcd for $[C_{25}H_{30}N]^+$ (M + H^+): 343.2373; found: 343.2381.

***N*-((1R,2S)-2-(2-fluoro-[1,1'-biphenyl]-4-yl)-1-phenylpropyl)aniline 5am**

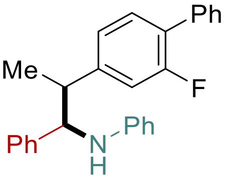

Following the **general procedure A**, 135 mg of **5am,** as an inseparable diastereomeric mixture (*dr* = 1:1), a white solid was obtained (yield: 71%); ^{1}H NMR (600 MHz, $CDCl_3$) δ 1.19 (d, J = 7.0 Hz, 3H), 1.35 (d, J = 7.1 Hz, 3H), 3.03-3.08 (m, 1H), 3.25-3.29 (m, 1H), 4.03 (br. s, 1H), 4.16 (br. s, 1H), 4.39 (d, J = 8.2 Hz, 1H), 4.56 (d, J = 5.5 Hz, 1H), 6.41-6.43 (m, 2H), 6.49-6.50 (m, 2H), 6.58-6.64 (m, 2H), 6.88-6.90 (m, 1H), 6.95-7.07 (m, 7H), 7.16-7.20 (m, 3H), 7.23-7.27 (m, 3H), 7.27-7.38 (m, 8H), 7.41-7.45 (m, 4H), 7.52-7.55 (m, 4H) ppm; ^{13}C NMR (150 MHz,

CDCl$_3$) δ 16.1, 19.5, 45.3, 46.9, 62.9, 63.6, 113.6, 113.7, 115.2, 115.3, 115.6, 115.7, 117.5, 123.7, 124.0, 124.1, 127.1, 127.2, 127.3, 128.1, 128.2, 128.4, 128.6, 128.9, 129.1, 129.2, 130.3, 130.8, 135.5, 135.6, 141.1, 142.2, 144.5, 144.6, 145.0, 147.0, 147.2, 158.7, 158.9, 160.3, 160.6 ppm; HRMS (ESI) *m/z* calcd for $[C_{27}H_{25}FN]^+$ (M + H^+): 382.1966; found: 382.1965.

2-(4-(1-Phenyl-1-(phenylamino)propan-2-yl)phenyl)isoindolin-1-one 5an

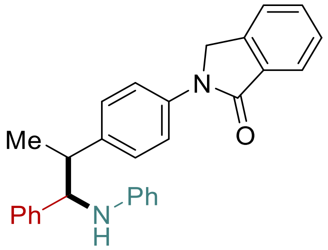

Following the **general procedure A**, 146 mg of **5an,** as an inseparable diastereomeric mixture (*dr* = 1:1.3), a white solid was obtained (yield: 70%); ^{1}H NMR (600 MHz, CDCl$_3$) δ 1.19 (d, *J* = 7.0 Hz, 3H), 1.34 (d, *J* = 7.1 Hz, 3.9H), 3.03-3.07 (m, 1.2H), 3.25-3.29 (m, 1.5H), 4.38 (d, *J* = 8.1 Hz, 1.1H), 4.55 (d, *J* = 5.5 Hz, 1.4H), 4.78-4.84 (m, 4.7H), 6.39-6.41 (m, 2H), 6.47-6.49 (m, 2.6H), 6.56-6.62 (m, 2.4H), 6.98-7.00 (m, 2H), 7.03-7.06 (m, 2.6H), 7.13-7.19 (m, 6.3H), 7.21-7.26 (m, 6.9H), 7.30-7.36 (m, 3.9H), 7.48-7.52 (m, 4.6H), 7.57-7.60 (m, 2.4H), 7.75-7.76 (m, 2.7H), 7.81-7.83 (m, 1.9H), 7.91-7.93 (m, 2.4H) ppm; ^{13}C NMR (150 MHz, CDCl$_3$) δ, 16.5, 19.6, 45.1, 46.8, 50.7, 63.0, 63.7, 113.6, 117.3, 119.1, 119.5, 122.6 (2C), 124.1 (2C), 126.9, 127.1, 127.5 (2C), 128.0, 128.3, 128.4 (3C), 128.8, 128.9, 129.0, 132.0, 132.1, 133.2 (2C), 138.0, 138.2, 138.8, 139.2, 140.0, 141.3, 142.6, 147.1, 147.4, 167.4, 167.5ppm; HRMS (ESI) *m/z* calcd for $[C_{29}H_{27}N_2O]^+$ (M + H^+): 419.2118; found: 419.2126.

***N*-(2,2-dimethyl-1-phenylpropyl)aniline 5ao**

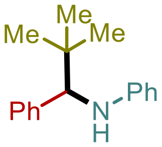

Following the **general procedure A**, 90 mg of **5ao**[15] as a pale yellow oil was obtained (yield: 75%); ^{1}H NMR (600 MHz, CDCl$_3$) δ 0.99 (s, 9H), 4.04 (s, 1H), 4.25 (s, 1H), 6.47-6.48 (m, 2H), 6.56-6.59 (m, 1H), 7.02-7.05 (m, 2H), 7.18-7.21 (m, 2H), 7.25-7.30 (m, 4H) ppm; ^{13}C NMR (150 MHz, CDCl$_3$) δ 27.0, 34.9, 67.1, 113.1, 116.9, 126.7, 127.7, 128.5, 129.0, 141.1, 147.7 ppm.

N-(phenyl(1-phenylcyclopentyl)methyl)aniline 5ap

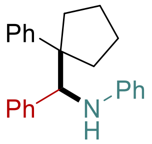

Following the **general procedure A**, 110 mg of **5ap** as a colorless oil was obtained (yield: 67%); ^{1}H NMR (600 MHz, $CDCl_3$) δ 1.61-1.68 (m, 2H), 1.75-1.89 (m, 3H), 2.00-2.04 (m, 1H), 2.09-2.14 (m, 1H), 2.26-2.30 (m, 1H), 4.18 (br.s, 1H), 4.46 (s, 1H), 6.40-6.41 (m, 2H), 6.53-6.55 (m, 1H), 6.88-6.89 (m, 2H), 6.98-7.01 (m, 2H), 7.05-7.07 (m, 2H), 7.15-7.17 (m, 2H), 7.23-7.29 (m, 3H) ppm; ^{13}C NMR (150 MHz, $CDCl_3$) δ 22.6, 22.7, 35.4, 36.4, 56.1, 62.8, 113.3, 116.9, 126.5, 126.9, 127.3, 127.7, 128.6, 128.9, 140.6, 143.1, 147.3 ppm.

N- (adamantan-1-yl)(phenyl)methyl)aniline 5aq

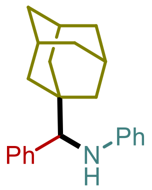

Following the **general procedure A**, 114 mg of **5aq**[5] as a white solid was obtained (yield: 72%); ^{1}H NMR (600 MHz, $CDCl_3$) δ 1.49-1.53 (m, 3H), 1.58-1.60 (m, 3H), 1.67-1.72 (m, 6H), 1.98 (br.s, 3H), 3.87 (s, 1H), 4.33 (br.s, 1H), 6.47-6.48 (m, 2H), 6.55-6.58 (m, 1H), 7.02-7.04 (m, 2H), 7.18-7.21 (m, 1H), 7.24-7.28 (m, 4H) ppm; ^{13}C NMR (150 MHz, $CDCl_3$) δ 28.4, 36.5, 36.9, 39.2, 67.9, 113.1, 116.7, 126.7, 127.6, 128.6, 129.0, 140.2, 147.9 ppm.

Methyl-4-(phenyl(phenylamino)methyl)bicyclo[2.2.2]octane-1-carboxylate 5ar

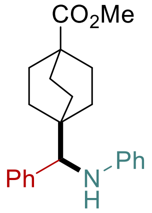

Following the **general procedure A**, 127 mg of **5ar** as a white solid was obtained (yield: 73%); ^{1}H NMR (600 MHz, $CDCl_3$) δ 1.44-1.49 (m, 3H), 1.1.63-1.68 (m, 3H), 1.76 (t, J = 8.0 Hz, 6H), 3.62 (s, 3H), 4.01 (s, 1H), 4.22 (br. s, 1H), 6.46-6.47 (m, 2H), 6.57-6.59 (m, 1H), 7.02-7.04 (m, 2H), 7.19-7.24 (m, 3H), 7.26-7.29 (m, 2H) ppm; ^{13}C NMR (150 MHz, $CDCl_3$) δ 27.5, 28.1, 35.5, 39.0, 51.7, 65.7, 113.2, 117.0, 126.9, 127.9, 128.5, 129.0, 140.5, 147.4, 178.1 ppm; HRMS (ESI) *m/z* calcd for

$[C_{23}H_{28}NO_2]^+$ (M + H$^+$): 350.2115; found: 350.2115.

***N*-(phenyl(2-(trifluoromethyl)-1,3-dioxolan-2-yl)methyl)aniline 5as**

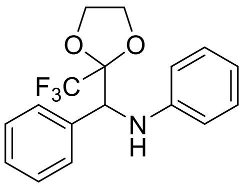

Following the **general procedure A**, 162 mg of **5as** as a white solid was obtained (yield: 80%); ^{1}H NMR (600 MHz, $CDCl_3$) δ 3.33-3.36 (m, 1H), 3.68-3.72 (m, 1H), 4.01-4.09 (m, 2H), 4.68 (br. s, 1H), 4.93 (s, 1H), 6.62-6.63 (m, 2H), 6.70-6.73 (m, 1H), 7.13-7.16 (m, 2H), 7.30-7.38 (m, 3H), 7.48-7.49 (m, 2H) ppm; ^{13}C NMR (150 MHz, $CDCl_3$) δ 55.0, 66.9, 67.3, 105.7 (q, *J* = 29.6 Hz), 113.4, 117.8, 123.3 (q, *J* = 289.5 Hz), 127.9, 128.0, 128.8, 129.1, 136.7, 146.0 ppm; HRMS (ESI) *m/z* calcd for $[C_{17}H_{17}F_3NO_2]^+$ (M + H$^+$): 324.1206; found: 324.1209.

***N*-(2-(4-chlorophenoxy)-2-methyl-1-phenylpropyl)aniline5at**

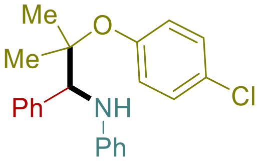

Following the **general procedure A**, 116 mg of **5at** as a white solid was obtained (yield: 66%); ^{1}H NMR (600 MHz, $CDCl_3$) δ 1.15 (s, 3H), 1.37 (s, 3H), 4.31 (s, 1H), 5.09 (br.s, 1H), 6.54 (d, *J* = 7.8, 2H), 6.62-6.64 (m, 1H), 6.83-6.86 (m, 2H), 7.05-7.08 (m, 2H), 7.23-7.26 (m, 1H), 7.29-7.31 (m, 2H), 7.42 (d, *J* = 7.4 Hz, 2H) ppm; ^{13}C NMR (150 MHz, $CDCl_3$) δ 23.7, 25.2, 66.1, 82.9, 113.7, 117.4, 125.6, 127.4, 128.0, 128.8, 129.0, 129.1, 129.3, 140.1, 147.4, 152.9 ppm; HRMS (ESI) *m/z* calcd for $[C_{22}H_{23}ClNO]^+$ (M + H$^+$): 352.1463; found: 352.1464.

***N*-(5-(2,5-dimethylphenoxy)-2,2-dimethyl-1-phenylpentyl)aniline 5au**

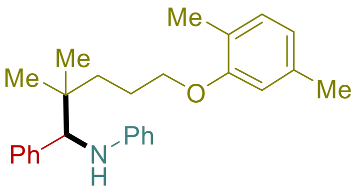

Following the **general procedure A**, 108 mg of **5au** as a colorless oil was obtained (yield: 56%); ^{1}H NMR (600 MHz, $CDCl_3$) δ 0.95 (s, 3H), 1.00 (s, 3H), 2.15 (s, 3H), 2.19 (s, 3H), 1.50-1.61 (m, 3H), 1.75-1.89 (m, 2H), 3.86-3.93 (m, 2H), 4.15 (s, 1H),

4.28 (s, 1H), 6.47 (d, *J* = 7.7 Hz, 2H), 6.57-6.59 (m, 2H), 6.65 (d, *J* = 7.4 Hz, 1H), 6.99 (d, *J* = 7.4 Hz, 1H), 7.03 (dd, *J* = 7.4, 8.5 Hz, 2H), 7.19-7.22 (m, 1H), 7.24-7.31 (m, 4H) ppm; ^{13}C NMR (150 MHz, $CDCl_3$) δ 15.8, 21.4, 23.4, 24.1, 24.5, 36.2, 37.2, 65.4, 68.3, 111.9, 113.1, 117.0, 120.6, 123.5, 1126.8, 127.7, 128.7, 129.0, 130.2, 136.5, 140.8, 147.5, 156.9 ppm; HRMS (ESI) *m/z* calcd for $[C_{27}H_{34}NO]^+$ (M + H^+): 3882635; found: 3882635.

N-(((4aS,10aR)-7-isopropyl-1,4a-dimethyl-1,2,3,4,4a,9,10,10a-octahydrophenanthren-1-yl)methyl)-4-methoxyaniline 5av

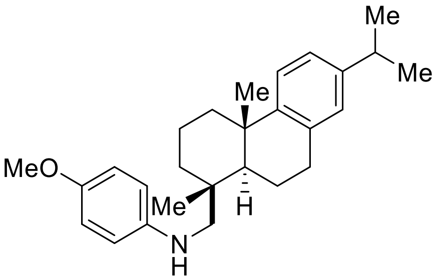

Following the **general procedure A**, 100 mg of **5av** as a white solid was obtained (yield: 51%); ^{1}H NMR (600 MHz, $CDCl_3$) δ 1.00 (s, 3H), 1.22 (d, *J* = 7.0 Hz, 6H), 1.23 (s, 3H), 1.39-1.46 (m, 3H), 1.61-1.83 (m, 5H), 2.29-2.31 (m, 1H), 2.79-2.92 (m, 4H), 2.99 (d, *J* = 12.3 Hz, 1H), 3.33 (br.s, 1H), 3.73 (s, 3H), 6.56-6.57 (m, 2H), 6.74-6.77 (m, 2H), 6.89 (s, 1H), 7.00 (dd, *J* = 1.6, 8.1 Hz, 1H), 7.18 (d, *J* = 8.1 Hz, 1H) ppm; ^{13}C NMR (150 MHz, $CDCl_3$) δ 18.7, 18.8, 19.3, 24.0, 25.3, 30.1, 33.4, 36.3, 37.4, 38.4, 45.3, 55.9, 56.1, 113.8, 114.9, 123.9, 124.2, 126.8, 134.7, 143.4, 145.6, 147.3, 151.7 ppm; HRMS (ESI) *m/z* calcd for $[C_{27}H_{38}NO]^+$ (M + H^+): 392.2948; found: 392.2950.

4-chloro-*N*-(4-((1-((4-methoxyphenyl)amino)-2-methylpropan-2-yl)oxy)phenethyl)benzamide 5aw

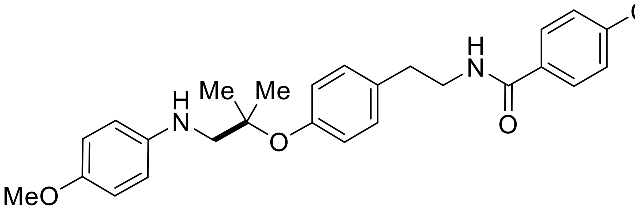

Following the **general procedure A**, 106 mg of **5aw** as a white solid was obtained (yield: 47%); ^{1}H NMR (600 MHz, $CDCl_3$) δ 1.35 (s, 6H), 2.87 (t, J = 7.0 Hz, 2H),

3.17 (s, 2H), 3.65-3.68 (m, 2H), 3.75 (s, 3H), 6.16 (br. s, 1H), 6.63-6.65 (m, 2H), 6.79-6.81 (m, 2H), 6.92-6.94 (m, 2H), 7.10-7.11 (m, 2H), 7.35-7.37 (m, 2H), 7.60-7.61 (m, 2H) ppm; ^{13}C NMR (150 MHz, $CDCl_3$) δ 25.1, 34.9, 41.2, 54.3, 55.8, 80.0, 114.1, 114.9, 124.2, 128.2, 128.8, 129.4, 132.9, 134.1, 137.6, 142.8, 152.0, 153.3, 166.4 ppm; HRMS (ESI) *m/z* calcd for $[C_{26}H_{30}ClN_2O_3]^+$ (M + H^+): 453.1939; found: 453.1938.

_N_-(2-(Benzyloxy)-1-(p-tolyl)ethyl)aniline 6a

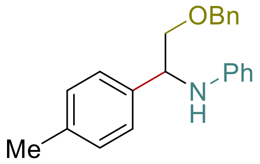

Following the **general procedure B**, 46 mg of **6a** as a colorless oil was obtained (yield: 73%); ^{1}H NMR (600 MHz, $CDCl_3$) δ 2.32 (s, 3H), 3.55-3.58 (m, 1H), 3.70-3.71 (m, 1H), 4.51-4.58 (m, 4H), 6.50-6.51 (m, 2H), 6.64-6.65 (m, 1H), 7.06-7.12 (m, 4H), 7.28-7.34 (m, 7H) ppm; ^{13}C NMR (150 MHz, $CDCl_3$) δ 21.1, 57.8, 72.9, 74.5, 113.9, 117.6, 126.6, 127.7, 127.8, 128.5, 128.9, 129.3, 137.0, 137.6, 137.8, 147.7 ppm; HRMS (ESI) *m/z* calcd for $[C_{22}H_{24}NO]^+$ (M + H^+): 318.1852; found: 318.1853.

_N_-(2-(benzyloxy)-1-(4-methoxyphenyl)ethyl)-4-methylaniline 6b

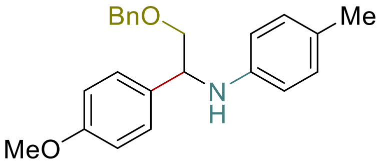

Following the **general procedure B**, 50 mg of **6b** as a colorless oil was obtained (yield: 72%); ^{1}H NMR (600 MHz, $CDCl_3$) δ 2.18 (s, 3H), 3.53-3.56 (m, 1H), 3.68 (dd, *J* = 4.1, 10.0 Hz, 1H), 3.77 (s, 3H), 4.46 (dd, *J* = 4.1, 8.6 Hz, 1H), 4.51 (d, *J* = 12.0 Hz, 1H), 4.56 (d, *J* = 12.0 Hz, 1H), 6.44 (d, *J* = 8.5 Hz, 2H), 6.84 (d, *J* = 8.5 Hz, 2H), 6.88 (d, *J* = 8.2 Hz, 2H), 7.27-7.35 (m, 7H) ppm; ^{13}C NMR (150 MHz, $CDCl_3$) δ 20.3, 55.2, 57.7, 72.8, 74.6, 114.0, 114.1, 126.7, 127.7, 127.8, 128.4, 129.4, 132.8, 137.8, 145.4, 158.8 ppm; HRMS (ESI) *m/z* calcd for $[C_{22}H_{24}NO2]^+$ (M + H^+): 334.1802; found: 334.1801.

_N_-(2-(benzyloxy)-1-(4-chlorophenyl)ethyl)aniline 6c

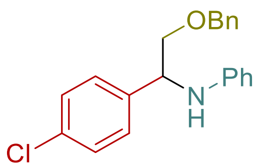

Following the **general procedure B**, 53 mg of **6c** as a colorless oil was obtained (yield: 79%); ^{1}H NMR (600 MHz, $CDCl_3$) δ 3.54 (dd, J = 8.1, 9.8 Hz, 1H), 3.70 (dd, J = 4.0, 9.8 Hz, 1H), 4.49-4.57 (m, 3H), 4.61 (br.s, 1H), 6.47-6.49 (m, 2H), 6.66-6.68 (m, 1H), 7.06-7.09 (m, 2H), 7.27-7.35 (m, 9H) ppm; ^{13}C NMR (150 MHz, $CDCl_3$) δ 57.5, 73.0, 74.1, 113.9, 117.9, 127.7, 127.9, 128.2, 128.5, 128.8, 129.0, 133.0, 137.6, 139.3, 147.2 ppm; HRMS (ESI) m/z calcd for $[C_{21}H_{21}ClNO]^+$ (M + H^+): 338.1306; found: 338.1306.

Methyl 4-(2-(benzyloxy)-1-(phenylamino)ethyl)benzoate 6d

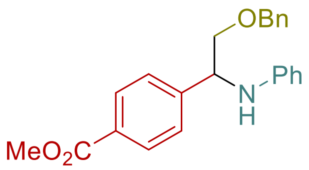

Following the **general procedure B**, 54 mg of **6d**[14] as a colorless oil was obtained (yield: 75%); ^{1}H NMR (600 MHz, $CDCl_3$) δ 3.59 (dd, J = 8.0, 9.8 Hz, 1H), 3.74-3.76 (m, 1H), 3.89 (s, 3H), 4.51 (d, J = 12.0 Hz, 1H), 4.56-4.59 (m, 2H), 4.66 (br.s, 1H), 6.47 (d, J = 7.8 Hz, 2H), 6.66-6.68 (m, 1H), 7.06-7.08 (m, 2H), 7.25-7.35 (m, 5H), 7.47 (d, J = 8.2 Hz, 2H), 7.99 (d, J = 8.3 Hz, 2H) ppm; ^{13}C NMR (150 MHz, $CDCl_3$) δ 52.1, 58.0, 73.0, 73.9, 113.8, 117.9, 126.9, 127.7, 127.9, 128.5, 129.0, 129.4, 129.9, 137.5, 146.2, 147.2, 166.9 ppm.

4-(2-(Benzyloxy)-1-(phenylamino)ethyl)benzonitrile 6e

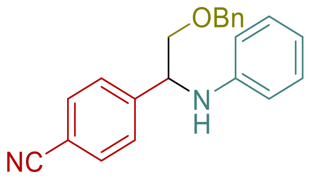

Following the **general procedure B**, 50 mg of **6e** as a colorless oil was obtained (yield: 76%); ^{1}H NMR (600 MHz, $CDCl_3$) δ 3.57 (dd, J = 7.5 Hz, 9.8 Hz, 1H), 3.75 (dd, J = 4.0, 9.8 Hz, 1H), 4.51 (d, J = 12.0 Hz, 1H), 4.55-4.57 (m, 2H), 4.66 (br.s, 1H), 6.44 (d, J = 8.0 Hz, 2H), 6.68-6.71 (m, 1H), 7.07-7.10 (m, 2H), 7.25-7.26 (m, 3H), 7.31-7.36 (m, 3H), 7.51 (d, J = 8.3 Hz, 2H), 7.60 (d, J = 8.3 Hz, 2H) ppm; ^{13}C NMR (150 MHz, $CDCl_3$) δ 57.9, 73.1, 73.6, 111.3, 113.7, 118.2, 118.8, 127.6, 127.7, 128.0,

128.5, 129.1, 132.5, 137.3, 146.5, 146.8 ppm; HRMS (ESI) *m/z* calcd for $[C_{22}H_{21}N_2O]^+$ (M + H^+): 329.1648; found: 329.1654.

1-(4-(2-(benzyloxy)-1-(p-tolylamino)ethyl)phenyl)ethan-1-one 6f

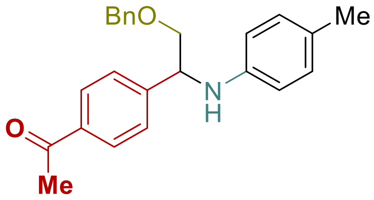

Following the **general procedure B**, 44 mg of **6f** as a colorless oil was obtained (yield: 61%); ^{1}H NMR (600 MHz, $CDCl_3$) δ 2.18 (s, 3H), 2.57 (s, 3H), 3.57-3.60 (m, 1H), 3.75 (dd, J = 4.0, 9.9 Hz, 1H), 4.50-4.58 (m, 4H), 6.40 (d, J = 8.1 Hz, 2H), 6.88 (d, J = 8.1 Hz, 2H), 7.27-7.35 (m, 5H), 7.49 (d, J = 8.0 Hz, 2H), 7.90 (d, J = 8.0 Hz, 2H) ppm; ^{13}C NMR (150 MHz, $CDCl_3$) δ 20.3, 26.6, 58.2, 73.0, 73.9, 113.9, 127.1, 127.2, 127.7, 127.9, 128.5, 128.7, 129.6, 136.4, 137.5, 144.8, 146.6, 197.8 ppm; HRMS (ESI) *m/z* calcd for $[C_{24}H_{26}NO_2]^+$ (M + H^+): 360.1958; found: 360.1965.

***N*-(2-(benzyloxy)-1-(4-(methylsulfonyl)phenyl)ethyl)-4-methylaniline 6g**

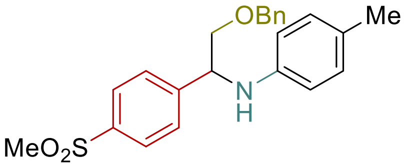

Following the **general procedure B**, 61 mg of **6g** as a colorless oil was obtained (yield: 77%); ^{1}H NMR (600 MHz, $CDCl_3$) δ 2.18 (s, 3H), 3.17 (s, 3H), 3.57 (dd, J = 7.7, 9.9 Hz, 1H), 3.74 (dd, J = 4.1, 9.9 Hz, 1H), 4.50-4.60 (m, 3H), 6.38 (d, J = 8.3 Hz, 2H), 6.89 (d, J = 8.3 Hz, 2H), 7.25-7.27 (m, 2H), 7.29-7.35 (m, 3H), 7.59 (d, J = 8.3 Hz, 2H), 7.87 (d, J = 8.3 Hz, 2H) ppm; ^{13}C NMR (150 MHz, $CDCl_3$) δ 20.3, 44.4, 57.9, 73.1, 73.7, 113.9, 127.4, 127.7, 127.9, 128.0, 128.5, 129.6, 137.3, 139.5, 144.4, 147.7 ppm; HRMS (ESI) *m/z* calcd for $[C_{23}H_{26}NO_3S]^+$ (M + H^+): 396.1628; found: 396.1637.

***N*-(4-(2-(benzyloxy)-1-(p-tolylamino)ethyl)phenyl)acetamide 6h**

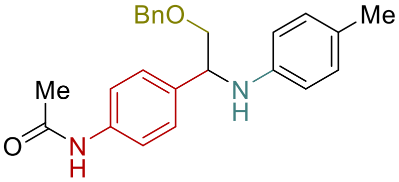

Following the **general procedure B**, 54 mg of **6h** as a colorless oil was obtained (yield: 72%); ^{1}H NMR (600 MHz, $CDCl_3$) δ 2.12 (s, 3H), 2.17 (s, 3H), 3.52-3.55 (m,

1H), 3.68 (dd, J = 4.0, 10.0 Hz, 1H), 4.46-4.56 (m, 4H), 6.42 (d, J = 8.3 Hz, 2H), 6.87 (d, J = 8.3 Hz, 2H), 7.28-7.29 (m, 3H), 7.32-7.35 (m, 4H), 7.38 (s, 1H), 7.42-7.43 (m, 2H) ppm; ^{13}C NMR (150 MHz, $CDCl_3$) δ 20.3, 24.5, 57.8, 729, 74.4, 114.0, 120.2, 126.8, 127.3, 127.7, 127.8, 128.4, 129.4, 136.8, 137.0, 137.7, 145.2, 168.3 ppm; HRMS (ESI) *m/z* calcd for $[C_{24}H_{26}N_2O_2Na]^+$ (M + Na$^+$): 397.1886; found: 397.1890.

***N*-(1-(4-(1H-pyrazol-1-yl)phenyl)-2-(benzyloxy)ethyl)-4-methylaniline 6i**

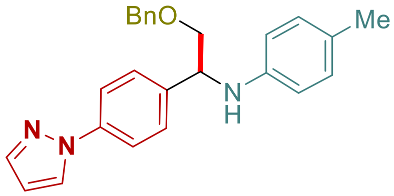

Following the **general procedure B**, 54 mg of **6i** as a colorless oil was obtained (yield: 71%); ^{1}H NMR (600 MHz, $CDCl_3$) δ 2.18 (s, 3H), 3.59 (dd, J = 8.3, 9.9 Hz, 1H), 3.74 (dd, J = 4.1, 9.9 Hz, 1H), 4.52-4.59 (m, 3H), 6.43-6.45 (m, 3H), 6.89 (d, J = 8.2 Hz, 2H), 7.28-7.30 (m, 3H), 7.33-7.35 (m, 2H), 7.47 (d, J = 8.5 Hz, 2H), 7.63 (d, J = 8.5 Hz, 2H), 7.70 (d, J = 1.6 Hz, 1H), 7.87 (d, J = 2.3 Hz, 1H) ppm; ^{13}C NMR (150 MHz, $CDCl_3$) δ 20.3, 57.8, 73.0, 74.3, 107.5, 114.1, 119.5, 126.7, 127.0, 127.7, 127.8, 127.9, 128.5, 129.5, 137.7, 139.2, 139.4, 141.0, 145.0 ppm; HRMS (ESI) *m/z* calcd for $[C_{25}H_{25}N_3ONa]^+$ (M + H$^+$): 406.1890; found: 406.1900.

***N*-(2-(benzyloxy)-1-(4-(4,4,5,5-tetramethyl-1,3,2-dioxaborolan-2-yl)phenyl)ethyl) aniline 6j**

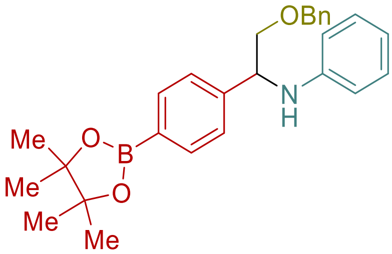

Following the **general procedure B**, 63 mg of **6j** as a colorless oil was obtained (yield: 73%); ^{1}H NMR (600 MHz, $CDCl_3$) δ 1.32 (s, 6H), 1.33 (s, 6H), 3.57 (dd, J = 8.5, 10.0 Hz, 1H), 3.73 (dd, J = 4.1, 10.0 Hz, 1H), 4.50-4.57 (m, 3H), 4.63 (br.s, 1H), 6.49 (d, J = 8.0 Hz, 2H), 6.63-6.66 (m, 1H), 7.04-7.07 (m, 2H), 7.28-7.36(m, 6H), 7.41 (d, J = 7.9 Hz, 2H), 7.77 (d, J = 8.0 Hz, 2H) ppm; ^{13}C NMR (150 MHz, $CDCl_3$) δ 24.8, 24.9, 58.3, 73.0, 74.3, 83.7, 113.9, 117.7, 126.2, 127.7, 127.8, 128.5, 129.0,

135.1, 137.7, 144.0, 147.5 ppm; HRMS (ESI) *m/z* calcd for $[C_{27}H_{33}BNO_3]^+$ (M + H^+): 430.2548; found: 430.2557.

***N*-(1-(4-(allyloxy)phenyl)-2-(benzyloxy)ethyl)-4-methylaniline 6k**

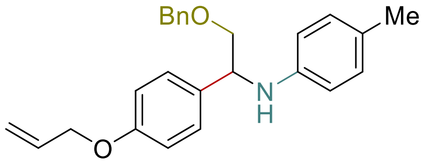

Following the **general procedure B**, 58 mg of **6k** as a colorless oil was obtained (yield: 78%); ^{1}H NMR (600 MHz, $CDCl_3$) δ 2.18 (s, 3H), 3.54 (dd, *J* = 8.8, 9.8 Hz, 1H), 3.68 (dd, *J* = 4.0, 9.8 Hz, 1H), 4.45-4.57 (m, 6H), 5.25-5.247(m, 1H), 5.38-5.41 (m, 1H), 6.00-6.07 (m, 1H), 6.43-6.44 (m, 2H), 6.85-6.88 (m, 4H), 7.28-7.30 (m, 5H), 7.32-7.35 (m, 2H) ppm; ^{13}C NMR (150 MHz, $CDCl_3$) δ 20.3, 57.7, 68.8, 72.8, 74.6, 114.0, 114.8, 117.6, 126.7, 127.7, 127.8, 128.4, 129.4, 132.9, 133.3, 137.8, 145.4, 157.9 ppm; HRMS (ESI) *m/z* calcd for $[C_{25}H_{28}NO_2]^+$ (M + H^+): 374.2115; found: 374.2110.

***N*-(2-(benzyloxy)-1-(2-chlorophenyl)ethyl)aniline 6l**

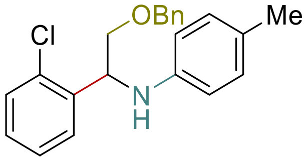

Following the **general procedure B**, 51 mg of **6l** as a colorless oil was obtained (yield: 72%); ^{1}H NMR (600 MHz, $CDCl_3$) δ 2.17 (s, 3H), 3.55 (dd, *J* = 7.9, 10.2 Hz, 1H), 3.83 (dd, *J* = 3.6, 10.2 Hz, 1H), 4.50-4.61 (m, 3H), 5.01 (dd, *J* = 3.6, 7.9 Hz, 1H), 6.38 (d, *J* = 8.5 Hz, 2H), 6.88 (d, *J* = 8.5 Hz, 2H), 7.15-7.18 (m, 2H), 7.27-7.36 (m, 6H), 7.51-7.52 (m, 1H) ppm; ^{13}C NMR (150 MHz, $CDCl_3$) δ 20.3, 54.6, 72.0, 72.7, 113.7, 126.9, 127.2, 127.7, 127.8, 128.4, 128.5, 128.6, 129.6, 132.7, 137.5, 137.8, 144.7 ppm; HRMS (ESI) *m/z* calcd for $[C_{21}H_{21}ClNO]^+$ (M + H^+): 338.1306; found: 338.1312.

2-(2-(benzyloxy)-1-(phenylamino)ethyl)phenol 6m

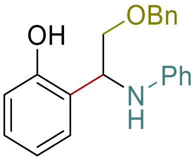

Following the **general procedure B**, 37 mg of **6m** as a colorless oil was obtained (yield: 58%); ^{1}H NMR (600 MHz, $CDCl_3$) δ 3.69 (dd, *J* = 4.3, 10.2 Hz, 1H), 3.79 (br.

t, J = 10.2 Hz, 1H), 4.52 (dd, J = 4.3, 10.2 Hz, 1H), 4.60 (br. s, 2H), 4.73 (br. s, 1H), 6.76-6.80 (m, 3H), 6.85-6.89 (m, 2H), 7.10-7.19 (m, 4H), 7.30-7.38 (m, 5H), 9.89 (br. s, 1H) ppm; ^{13}C NMR (150 MHz, $CDCl_3$) δ 60.8, 71.7, 73.1, 116.9, 117.2, 120.0, 121.3, 122.5, 127.8, 128.1, 128.2, 128.6, 129.1, 129.2, 137.3, 146.7, 157.3 ppm; HRMS (ESI) *m/z* calcd for $[C_{21}H_{22}NO_2]^+$ (M + H^+): 320.1645; found: 320.1643.

3-(2-(Benzyloxy)-1-(p-tolylamino)ethyl)phenol 6n

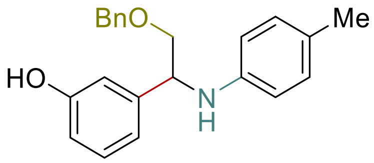

Following the **general procedure B**, 41 mg of **6n** as a colorless oil was obtained (yield: 61%); ^{1}H NMR (600 MHz, $CDCl_3$) δ 2.18 (s, 3H), 3.55 (dd, J = 8.6, 10.0 Hz, 1H), 3.69 (dd, J = 4.0, 10.0 Hz, 1H), 4.44 (dd, J = 4.0, 8.6 Hz, 1H), 4.50 (d, J = 12.0 Hz, 1H), 4.55 (d, J = 12.0 Hz, 1H), 6.43 (d, J = 8.3 Hz, 2H), 6.68-6.70 (m, 1H), 66.84-6.85 (m, 1H), 6.88 (d, J = 8.3 Hz, 2H), 6.93 (d, J = 7.6 Hz, 1H), 7.15-7.18 (m, 2H), 7.28-7.30 (m, 3H), 7.32-7.35 (m, 2H) ppm; ^{13}C NMR (150 MHz, $CDCl_3$) δ 20.3, 58.1, 72.9, 74.3, 113.6, 114.1, 114.4, 119.2, 126.9, 127.7, 127.8, 128.5, 129.5, 129.8, 137.7, 142.9, 145.2, 155.9 ppm; HRMS (ESI) *m/z* calcd for $[C_{22}H_{24}NO_2]^+$ (M + H^+): 334.1802; found: 334.1803.

4-(2-(Benzyloxy)-1-(p-tolylamino)ethyl)phenol 6o

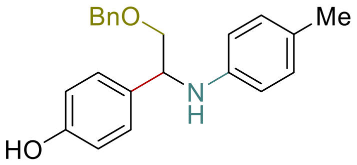

Following the **general procedure B**, 47 mg of **6o** as a colorless oil was obtained (yield: 70%); ^{1}H NMR (600 MHz, $CDCl_3$) δ 2.18 (s, 3H), 3.52-3.55 (m, 1H), 3.66-3.68 (m, 1H), 4.44 (dd, J = 3.9, 8.3 Hz, 1H), 4.51 (d, J = 11.9 Hz, 1H), 4.56 (d, J = 11.9 Hz, 1H), 6.43-6.44 (m, 2H), 6.72-6.74 (m, 2H), 6.87-6.89 (m, 2H), 7.21-7.24 (m, 2H), 7.28-7.35 (m, 5H) ppm; ^{13}C NMR (150 MHz, $CDCl_3$) δ 20.3, 57.7, 72.9, 74.5, 114.1, 115.5, 126.8, 127.7, 127.8, 128.0, 128.5, 129.5, 132.8, 137.7, 145.3, 154.8 ppm; HRMS (ESI) *m/z* calcd for $[C_{22}H_{24}NO_2]^+$ (M + H^+): 334.1802; found: 334.1796.

***N*-(2-(benzyloxy)-1-(3,4-dichlorophenyl)ethyl)-4-methylaniline 6p**

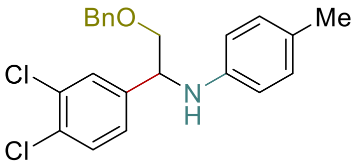

Following the **general procedure B**, 45 mg of **6p** as a colorless oil was obtained (yield: 58%); ^{1}H NMR (600 MHz, $CDCl_3$) δ 2.19 (s, 3H), 3.53 (dd, *J* = 7.9, 9.8 Hz, 1H), 3.68 (dd, *J* = 4.1, 9.8 Hz, 1H), 4.44 (dd, *J* = 4.1, 7.9 Hz, 1H), 4.49-4.56 (m, 3H), 6.39 (d, *J* = 8.3 Hz, 2H), 6.90 (d, *J* = 8.3 Hz, 2H), 7.22-7.23 (m, 1H), 7.26-7.37 (m, 6H), 7.48 (d, *J* = 8.3 Hz, 1H) ppm; ^{13}C NMR (150 MHz, $CDCl_3$) δ 20.3, 57.5, 73.0, 73.8, 113.9, 126.2, 127.3, 127.7, 127.9, 128.5, 128.8, 129.6, 130.6, 131.2, 132.7, 137.4, 141.5, 144.6 ppm; HRMS (ESI) *m/z* calcd for $[C_{21}H_{20}C_{l2}NO]^+$ (M + H^+): 372.0916; found: 372.0924.

_N_-(2-(benzyloxy)-1-(3,4,5-trimethoxyphenyl)ethyl)-4-methylaniline 6q

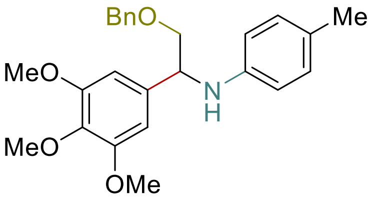

Following the **general procedure B**, 66 mg of **6q** as a pale yellow solid was obtained (yield: 81%); ^{1}H NMR (600 MHz, $CDCl_3$) δ 2.20 (s, 3H), 3.57 (dd, *J* = 8.8, 9.7 Hz, 1H), 3.71 (dd, *J* = 4.1, 9.7 Hz, 1H), 3.81 (s, 6H), 3.82 (s, 3H), 4.40 (dd, *J* = 4.1, 8.8 Hz, 1H), 4.52 (d, *J* = 11.9 Hz, 1H), 4.57 (d, *J* = 11.9 Hz, 1H), 6.46-6.48 (m, 2H), 6.63 (s, 2H), 6.90-6.92 (m, 2H), 7.30-7.31 (m, 3H), 7.33-7.36 (m, 2H) ppm; ^{13}C NMR (150 MHz, $CDCl_3$) δ 20.3, 56.0, 59.0, 60.8, 72.9, 74.5, 103.4, 114.1, 127.0, 127.7, 127.8, 128.4, 129.5, 136.8, 137.0, 137.8, 145.5, 153.4 ppm; HRMS (ESI) *m/z* calcd for $[C_{25}H_{29}NO_4Na]^+$ (M + H^+): 430.1989; found: 430.1994.

_N_-(2-(benzyloxy)-1-(naphthalen-2-yl)ethyl)aniline 6r

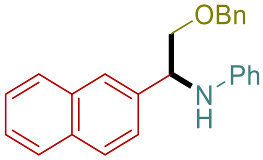

Following the **general procedure B**, 56 mg of **6r** as a colorless oil was obtained (yield: 79%); ^{1}H NMR (600 MHz, $CDCl_3$) δ 3.66 (dd, *J* = 8.6, 9.9 Hz, 1H), 3.81 (dd, *J* = 4.1, 9.9 Hz, 1H), 4.53 (d, *J* = 12.0 Hz, 1H), 4.58 (d, *J* = 12.0 Hz, 1H), 4.70 (dd, *J* = 4.1, 8.4 Hz, 1H), 6.55 (d, *J* = 7.9 Hz, 2H), 6.64-6.66 (m, 1H), 7.04-7.06 (m, 2H), 7.29-7.34 (m, 5H), 7.42-7.46 (m, 2H), 7.51-7.53 (m, 1H), 7.78-7.81 (m, 3H), 7.86 (s,

1H) ppm; ^{13}C NMR (150 MHz, $CDCl_3$) δ 58.4, 73.0, 74.3, 114.0, 117.8, 127.9, 125.6, 125.7, 126.0, 127.6, 127.7, 127.8, 128.4, 128.5, 129.0, 133.0, 133.5, 137.7, 138.2, 147.6 ppm; HRMS (ESI) *m/z* calcd for $[C_{25}H_{24}NO]^+$ (M + H^+): 354.1852; found: 354.1852.

***N*-(2-(benzyloxy)-1-(pyridin-3-yl)ethyl)-4-methylaniline 6s**

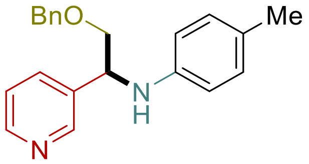

Following the **general procedure B**, 39 mg of **6s** as a colorless oil was obtained (yield: 61%); ^{1}H NMR (600 MHz, $CDCl_3$) δ 2.18 (s, 3H), 3.59 (dd, *J* = 7.7, 9.7 Hz, 1H), 3.75 (dd, *J* = 4.1, 9.7 Hz, 1H), 4.51-4.58 (m, 3H), 6.42 (d, *J* = 8.4 Hz, 2H), 6.89 (d, *J* = 8.2 Hz, 2H), 7.21-7.23 (m, 1H), 7.25-7.35 (m, 5H), 7.71-7.72 (m, 1H), 8.50 (d, *J* = 3.7 Hz, 1H), 8.64 (s, 1H) ppm; ^{13}C NMR (150 MHz, $CDCl_3$) δ 20.3, 56.0, 73.1, 73.8, 114.0, 123.6, 127.3, 127.7, 127.9, 128.5, 129.6, 134.7, 136.5, 137.5, 144.6, 148.7, 148.8 ppm; HRMS (ESI) *m/z* calcd for $[C_{21}H_{23}N_2O]^+$ (M + H^+): 319.1805; found: 319.1810.

***N*-(2-(benzyloxy)-1-(1H-indol-5-yl)ethyl)-4-methylaniline 6t**

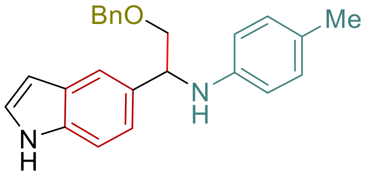

Following the **general procedure B**, 48 mg of **6t** as a colorless oil was obtained (yield: 67%); ^{1}H NMR (600 MHz, $CDCl_3$) δ 2.15 (s, 3H), 3.59-3.63 (m, 1H), 3.74 (dd, *J* = 4.1, 10.1 Hz, 1H), 4.52 (d, *J* = 12.1 Hz, 1H), 4.57 (d, *J* = 12.1 Hz, 1H), 4.61 (dd, *J* = 4.1, 9.0 Hz, 1H), 6.47-6.50 (m, 3H), 6.85 (d, *J* = 8.3 Hz, 2H), 7.12-7.13 (m, 1H), 7.22-7.23 (m, 1H), 7.27-7.34 (m, 6H), 7.66 (s, 1H), 8.04 (s, 1H) ppm; ^{13}C NMR (150 MHz, $CDCl_3$) δ 20.3, 58.7, 72.8, 75.2, 102.5, 111.1, 114.2, 118.7, 120.9, 124.5, 126.6, 127.7, 128.0, 128.4, 129.4, 132.1, 135.3, 137.9, 145.7 ppm; HRMS (ESI) *m/z* calcd for $[C_{24}H_{25}N_2O]^+$ (M + H^+): 357.1962; found: 357.1966.

***N*-(2-(benzyloxy)-1-(9-ethyl-9H-carbazol-3-yl)ethyl)aniline 6u**

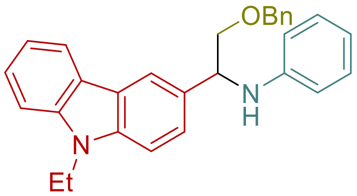

Following the **general procedure B**, 58 mg of **6u** as a colorless oil was obtained (yield: 69%); ^{1}H NMR (600 MHz, $CDCl_3$) δ 1.40 (t, J = 7.2 Hz, 3H), 3.66-3.69 (m, 1H), 3.80 (dd, J = 4.1, 10.2 Hz, 1H), 4.31 (q, J = 7.2 Hz, 2H), 4.55 (d, J = 12.1 Hz, 1H), 4.60 (d, J = 12.1 Hz, 1H), 4.72 (dd, J = 4.1, 8.8 Hz, 1H), 6.60 (d, J = 7.9 Hz, 2H), 6.62-6.65 (m, 1H), 7.03-7.06 (m, 2H), 7.18-7.21 (m, 1H), 7.28-7.37 (m, 7H), 7.42-7.45 (m, 1H), 7.49-7.51 (m, 1H), 8.06 (d, J = 7.7 Hz, 1H), 8.13 (s, 1H) ppm; ^{13}C NMR (150 MHz, $CDCl_3$) δ 13.8, 37.5, 58.6, 72.9, 75.1, 108.4, 108.6, 114.1, 117.6, 118.6, 118.7, 120.5, 122.7, 123.1, 124.4, 125.6, 127.7, 127.8, 128.5, 128.9, 131.0, 137.9, 139.5, 140.2, 148.0 ppm; HRMS (ESI) *m/z* calcd for $[C_{29}H_{28}N_2ONa]^+$ (M + H^+): 443.2094; found: 443.2103.

***N*-(2-(benzyloxy)-1-(dibenzo[b,d]furan-2-yl)ethyl)-4-methylaniline 6v**

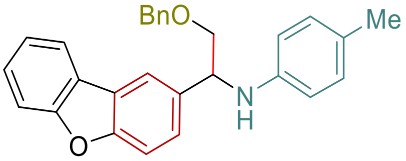

Following the **general procedure B**, 59 mg of **6v** as a colorless oil was obtained (yield: 72%); ^{1}H NMR (600 MHz, $CDCl_3$) δ 2.16 (s, 3H), 3.62-3.65 (m, 1H), 3.78 (dd, J = 4.1, 10.1 Hz, 1H), 4.54 (d, J = 11.9 Hz, 1H), 4.59 (d, J = 11.9 Hz, 1H), 4.66 (dd, J = 4.1, 8.6 Hz, 1H), 6.49 (d, J = 8.5 Hz, 2H), 6.87 (d, J = 8.5 Hz, 2H), 7.28-7.35 (m, 6H), 7.41-7.44 (m, 1H), 7.48-7.51 (m, 2H), 7.53 (d, J = 7.7 Hz, 1H), 7.90 (d, J = 7.7 Hz, 1H), 8.00 (s, 1H) ppm; ^{13}C NMR (150 MHz, $CDCl_3$) δ 30.3, 58.5, 73.0, 74.8, 111.6, 111.7, 114.2, 118.9, 120.8, 122.6, 124.1, 124.5, 125.9, 127.0, 127.1, 127.7, 127.8, 128.5, 129.5, 135.5, 137.7, 145.3, 155.6, 156.5 ppm; HRMS (ESI) *m/z* calcd for $[C_{28}H_{26}NO_2]^+$ (M + H^+): 408.1958; found: 408.1962.

***N*- (1-(benzo[b]thiophen-3-yl)-2-(benzyloxy)ethyl)-4-methylaniline 6w**

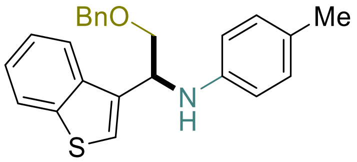

Following the **general procedure B**, 54 mg of **6w** as a colorless oil was obtained (yield: 72%); ^{1}H NMR (600 MHz, $CDCl_3$) δ 2.19 (s, 3H), 3.72 (dd, *J* = 7.8, 10.0 Hz, 1H), 3.92 (dd, *J* = 4.0, 10.0 Hz, 1H), 4.54 (d, *J* = 12.0 Hz, 1H), 4.58 (d, *J* = 12.0 Hz, 1H), 4.95 (dd, *J* = 4.0, 7.8 Hz, 1H), 6.47 (d, *J* = 8.4 Hz, 2H), 6.89 (d, *J* = 8.1 Hz, 2H), 7.28-7.39 (m, 7H), 7.41 (s, 1H), 7.80-7.82 (m, 1H), 7.86-7.87 (m, 1H) ppm; ^{13}C NMR (150 MHz, $CDCl_3$) δ 20.3, 53.5, 72.2, 73.0, 114.0, 121.4, 123.1, 123.9, 124.0, 124.3, 127.1, 127.7, 127.8, 128.5, 129.6, 134.9, 137.4, 137.7, 141.3, 145.1 ppm; HRMS (ESI) *m/z* calcd for $[C_{24}H_{24}NOS]^+$ (M + H^+): 374.1573; found: 374.1574.

N-(1-(benzo[d]thiazol-2-yl)-2-(benzyloxy)ethyl)-4-methylaniline 6x

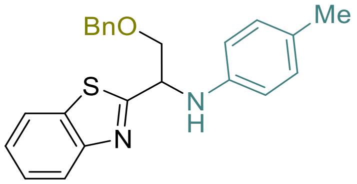

Following the **general procedure B**, 51 mg of **6x** as a colorless oil was obtained (yield: 68%); ^{1}H NMR (600 MHz, $CDCl_3$) δ 2.20 (s, 3H), 3.96 (dd, *J* = 6.7, 9.7 Hz, 1H), 4.03 (dd, *J* = 4.1, 9.7 Hz, 1H), 4.55 (d, *J* = 12.1 Hz, 1H), 4.58 (d, *J* = 12.1 Hz, 1H), 4.79 (br.s, 1H), 4.99 (dd, *J* = 4.1, 6.7 Hz, 1H), 6.58 (d, *J* = 8.3 Hz, 2H), 6.95 (d, *J* = 8.3 Hz, 2H), 7.27-7.36 (m, 6H), 7.45-7.48 (m, 1H), 7.81 (d, *J* = 8.0 Hz, 1H), 7.99 (d, *J* = 8.1 Hz, 1H) ppm; ^{13}C NMR (150 MHz, $CDCl_3$) δ 20.4, 57.5, 72.3, 73.2, 113.9, 121.8, 122.7, 124.8, 125.8, 127.7, 127.9, 128.1, 128.5, 129.7, 135.2, 137.4, 144.2, 153.6, 175.8 ppm; HRMS (ESI) *m/z* calcd for $[C_{23}H_{23}N_2OS]^+$ (M + H^+): 375.1526; found: 375.1526.

(4-(2-(benzyloxy)-1-(p-tolylamino)ethyl)phenyl)(4-(2-chlorodibenzo[b,f][1,4]oxazepin-11-yl)piperazin-1-yl)methanone 6y

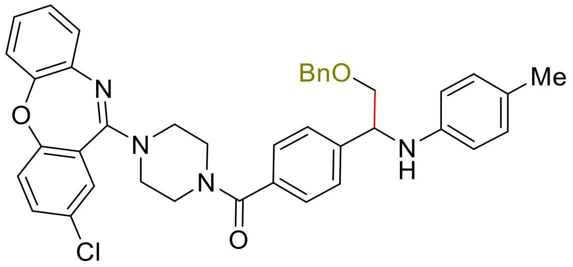

Following the **general procedure B**, 89 mg of **6y** as a white solid was obtained (yield: 68%); ^{1}H NMR (600 MHz, $CDCl_3$) δ 2.19 (s, 3H), 3.53-3.57 (m, 7H), 3.71 (dd, *J* = 3.8, 10.0 Hz, 1H), 3.88 (br. s, 2H), 4.51-4.58 (m, 4H), 6.42 (d, *J* = 8.1 Hz, 2H), 6.88 (d, *J* = 8.1 Hz, 2H), 7.00-7.02 (m, 1H), 7.08-7.14 (m, 3H), 7.18-7.19 (m, 1H),

7.28-7.40 (m, 9H), 7.44-7.45 (m, 2H) ppm; ^{13}C NMR (150 MHz, $CDCl_3$) δ 20.3, 41.8, 47.4, 58.1, 72.9, 74.2, 114.0, 120.1, 122.8, 124.7, 125.0, 125.8, 127.0, 127.1, 127.5, 127.7, 127.9, 128.5, 128.8, 129.5, 130.4, 132.8, 134.4, 137.6, 139.7, 143.1, 145.0, 151.7, 158.7, 159.3, 170.4 ppm; HRMS (ESI) *m/z* calcd for $[C_{40}H_{38}ClN_4O_3]^+$ (M + H^+): 657.2627; found: 657.2626.

3-(2-(benzyloxy)-1-(p-tolylamino)ethyl)phenyl 2-(1-(4-chlorobenzoyl)-5-methoxy-2-methyl-1H-indol-3-yl)acetate 6z

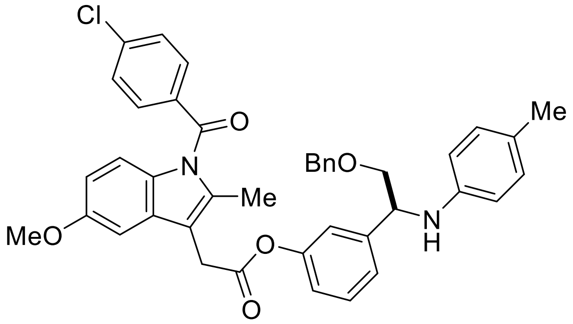

Following the **general procedure B**, 101 mg of **6z** as a white solid was obtained (yield: 75%); ^{1}H NMR (600 MHz, $CDCl_3$) δ 2.18 (s, 3H), 2.43 (s, 3H), 1.78 (s, 3H), 3.53-3.56 (m, 1H), 3.70 (dd, *J* = 4.0, 9.9 Hz, 1H), 3.81 (s, 3H), 3.87 (s, 2H), 4.47-4.55 (m, 3H), 6.41 (d, *J* = 8.3 Hz, 2H), 6.68 (dd, *J* = 2.4, 9.0 Hz, 1H), 6.87-6.90 (m, 3H), 6.96-6.97 (m, 1H), 7.03 (d, *J* = 2.4 Hz, 1H), 7.15 (s, 1H), 7.26-7.36 (m, 7H), 7.46 (d, *J* = 8.5 Hz, 2H), 7.66 (d, *J* = 8.5 Hz, 2H) ppm; ^{13}C NMR (150 MHz, $CDCl_3$) δ 13.4, 20.3, 30.5, 55.7, 58.2, 72.9, 74.2, 101.1, 111.8, 112.0, 114.0, 115.0, 119.6, 120.4, 124.3, 127.0, 127.7, 127.8, 128.4, 128.5, 129.1, 129.5, 130.5, 130.8, 131.2, 133.8, 136.2, 137.6, 139.3, 142.9, 145.1, 151.0, 156.1, 168.3, 169.2 ppm; HRMS (ESI) *m/z* calcd for $[C_{41}H_{38}ClN_2O_5]^+$ (M + H^+): 673.2464; found: 673.2471.

(Z)-3,7-dimethylocta-2,6-dien-1-yl-4-(2-(benzyloxy)-1-(*p*-tolylamino)ethyl)benzoate 6aa

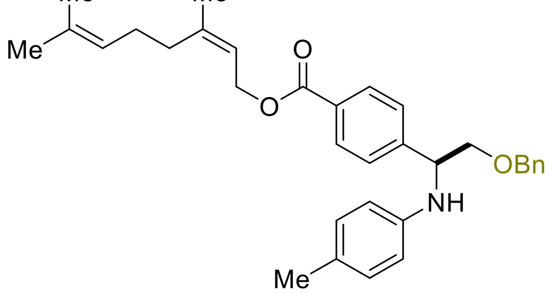

Following the **general procedure B**, 78 mg of **6aa** as a white solid was obtained (yield: 78%); ^{1}H NMR (600 MHz, $CDCl_3$) δ 1.59 (s, 3H), 1.66 (s, 3H), 1.78 (s, 3H),

2.08-2.12 (m, 2H), 2.16-2.18 (m, 5H), 3.56-3.59 (m, 1H), 3.73 (dd, *J* = 4.0, 9.9 Hz, 1H), 4.50-4.57 (m, 4H), 4.78 (d, *J* = 7.1 Hz, 2H), 5.11 (t, *J* = 7.0 Hz, 1H), 5.46 (t, *J* = 7.1 Hz, 1H), 6.39 (d, *J* = 8.3 Hz, 2H), 6.87 (d, *J* = 8.3 Hz, 2H), 7.27-7.35 (m, 5H), 7.45 (d, *J* = 8.2 Hz, 2H), 7.99 (d, *J* = 8.2 Hz, 2H) ppm; ^{13}C NMR (150 MHz, $CDCl_3$) δ 17.7, 20.3, 23.5, 25.7, 26.7, 32.2, 58.2, 61.5, 73.0, 74.0, 114.0, 119.2, 123.6, 126.8, 127.1, 127.7, 127.9, 128.5, 129.5, 129.7, 129.9, 132.2, 137.6, 142.7, 144.9, 146.2, 166.5 ppm; HRMS (ESI) *m/z* calcd for $[C_{33}H_{40}NO_3]^+$ (M + H^+): 498.3003; found: 498.3007.

5-Chloro-2-(2,4-dichlorophenoxy)phenyl 4-(2-(benzyloxy)-1-(*p*-tolylamino)ethyl)benzoate 6ab

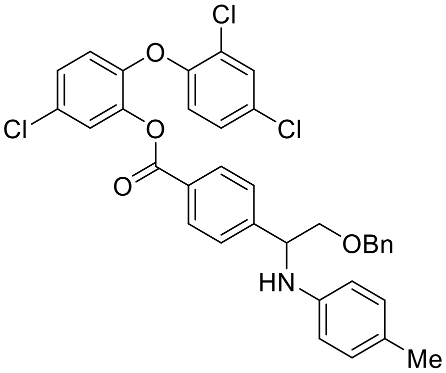

Following the **general procedure B**, 95 mg of **6ab** as a white solid was obtained (yield: 75%); ^{1}H NMR (600 MHz, $CDCl_3$) δ 2.18 (s, 3H), 3.58 (dd, *J* = 7.9, 9.8 Hz, 1H), 3.75 (dd, *J* = 3.9, 9.8 Hz, 1H), 4.51-4.58 (m, 3H), 6.40 (d, *J* = 8.3 Hz, 2H), 6.87-6.91 (m, 4H), 7.11-7.13 (m, 1H), 7.18-7.20 (m, 1H), 7.27-7.35 (m, 7H), 7.48 (d, *J* = 8.3 Hz, 2H), 7.98 (d, *J* = 8.3 Hz, 2H) ppm; ^{13}C NMR (150 MHz, $CDCl_3$) δ 20.3, 58.2, 73.0, 73.9, 114.0, 120.2, 120.5, 124.6, 126.0, 127.0, 127.1, 127.2, 127.5, 127.7, 127.9, 128.0, 128.5, 129.2, 129.4, 129.6, 130.3, 130.6, 137.5, 141.8, 144.7, 146.9, 147.7, 151.0, 163.8 ppm; HRMS (ESI) *m/z* calcd for $[C_{35}H_{29}Cl_3NO_4]^+$ (M + H^+): 632.1157; found: 632.1160.

Ethyl *O*-benzyl-N-(p-tolyl)serinate 6ac

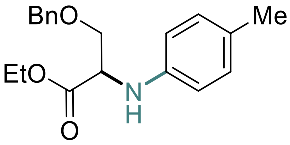

Following the **general procedure C**, 42 mg of **6ac** as a colorless oil was obtained (yield: 67%); ^{1}H NMR (600 MHz, $CDCl_3$) δ 1.22 (t, *J* = 7.2 Hz, 3H), 2.22 (s, 3H), 3.78 (dd, *J* = 4.2, 9.3 Hz, 1H), 3.86 (dd, *J* = 4.0, 9.3 Hz, 1H), 4.16-4.21 (m, 3H), 4.40

(br.s, 1H), 4.52 (d, J = 12.1 Hz, 1H), 4.58 (d, J = 12.1 Hz, 1H), 6.54 (d, J = 8.3 Hz, 2H), 6.97 (d, J = 8.3 Hz, 1H), 7.27-7.34 (m, 5H) ppm; ^{13}C NMR (150 MHz, $CDCl_3$) δ 14.2, 20.4, 57.3, 61.2, 70.1, 73.3, 113.8, 127.6, 127.7, 128.4, 129.7, 137.7, 144.3, 172.1 ppm; HRMS (ESI) *m/z* calcd for $[C_{19}H_{24}NO_3]^+$ (M + H^+): 314.1751; found: 314.1758.

Ethyl *O*-benzyl-*N*-(4-methoxyphenyl)serinate 6ad

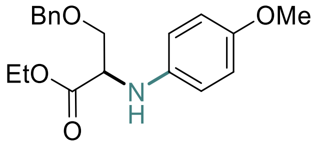

Following the **general procedure C**, 43 mg of **6ad** as a colorless oil was obtained (yield: 65%); ^{1}H NMR (600 MHz, $CDCl_3$) δ 1.22 (t, J = 7.2 Hz, 3H), 3.72 (s, 3H), 3.77 (dd, J = 4.1, 9.3 Hz, 1H), 3.85 (dd, J = 4.3, 9.3 Hz, 1H), 4.14 (t, J = 4.3 Hz, 1H), 4.18 (q, J = 7.2 Hz, 2H), 4.52 (d, J = 12.2 Hz, 1H), 4.58 (d, J = 12.2 Hz, 1H), 6.59-6.61 (m, 2H), 6.75-6.76 (m, 2H), 7.27-7.29 (m, 3H), 7.32-7.34 (m, 2H) ppm; ^{13}C NMR (150 MHz, $CDCl_3$) δ 14.1, 55.6, 58.0, 61.2, 70.2, 73.2, 114.7, 115.2, 127.5, 127.7, 128.3, 137.6, 140.7, 152.7, 172.1 ppm; HRMS (ESI) *m/z* calcd for $[C_{19}H_{24}NO_4]^+$ (M + H^+): 330.1700; found: 330.1707.

Ethyl-3-(2-bromophenyl)-2-(p-tolylamino)propanoate 6ae

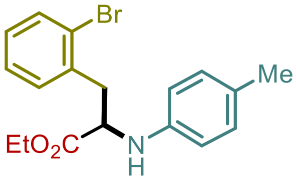

Following the **general procedure C**, 44 mg of **6ae** as a colorless oil was obtained (yield: 61%); ^{1}H NMR (600 MHz, $CDCl_3$) δ 1.10 (t, J = 7.2 Hz, 3H), 2.21 (s, 3H), 3.15 (dd, J = 7.1, 13.7 Hz, 1H), 3.27 (dd, J = 7.4, 13.7 Hz, 1H), 4.02-4.11 (m, 2H), 4.40 (br.t, 7.1 Hz, 1H), 6.54 (d, J = 8.3 Hz, 2H), 6.95 (d, J = 8.3 Hz, 2H), 7.07-7.10 (m, 1H), 7.19-7.22 (m, 2H), 7.54 (d, J = 8.3 Hz, 1H) ppm; ^{13}C NMR (150 MHz, $CDCl_3$) δ 14.0, 20.3, 39.5, 57.1, 61.0, 113.8, 124.8, 127.4, 127.7, 128.5, 129.7, 131.4, 132.9, 136.5, 144.1, 173.4 ppm; HRMS (ESI) *m/z* calcd for $[C_{18}H_{21}BrNO_2]^+$ (M + H^+): 362.0750; found: 362.0757.

***N*-(2-(benzyloxy)ethyl)aniline 6af**

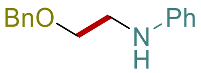

Following the **general procedure A**, 69 mg of **6af** [16] as a yellowish liquid was obtained (yield: 61%); ^{1}H NMR (600 MHz, $CDCl_3$) δ 3.35 (t, J = 5.2 Hz, 2H), 3.72 (t, J = 5.3 Hz, 2H), 4.57 (s, 2H), 4.07 (br. s, 1H), 6.64-6.66 (m, 2H), 6.72-6.75 (m, 1H), 7.18-7.21 (m, 2H), 7.31-7.33 (m, 1H), 7.37-7.38 (m, 4H) ppm; ^{13}C NMR (150 MHz, $CDCl_3$) δ 43.6, 68.6, 73.1, 113.1, 117.6, 127.7, 128.4, 129.2, 138.0, 148.2 ppm

4-methoxy-*N*-((tetrahydrofuran-2-yl)methyl)aniline 6ag

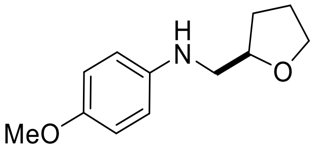

Following the **general procedure A**, 67 mg of **6ag**[17] as a yellowish liquid was obtained (yield: 67%); ^{1}H NMR (600 MHz, $CDCl_3$) δ 1.62-1.68 (m, 1H), 1.88-1.95 (m, 2H), 2.00-2.05 (m, 1H), 3.03 (dd, J = 7.6, 12.2 Hz, 1H), 3.20 (dd, J = 3.7, 12.2 Hz, 1H), 3.74 (s, 3H), 3.76-3.80 (m, 1H), 3.86-3.90 (m, 1H), 4.12 (ddd, J = 3.7, 7.1, 10.9 Hz, 1H), 6.60-6.62 (m, 2H), 6.77-6.78 (m, 2H) ppm; ^{13}C NMR (150 MHz, $CDCl_3$) δ 25.8, 29.1, 49.3, 55.8, 68.0, 77.6, 114.4, 114.8, 142.6, 152.2 ppm.

***N*-(cyclohexylmethyl)aniline 6ah**

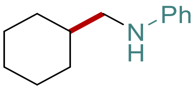

Following the **general procedure A**, 55 mg of **6ah**[18] as a yellowish liquid was obtained (yield: 58%); ^{1}H NMR (600 MHz, $CDCl_3$) δ 0.95-1.01 (m, 2H), 1.15-1.28 (m, 2H), 1.56-1.60 (m, 1H), 1.67-1.69 (m, 1H), 1.73-1.75 (m, 2H), 1.80-1.82 (m, 2H), 2.95 (d, J = 6.7 Hz, 2H), 3.70 (br. s, 1H), 6.58-6.59 (m, 2H), 6.65-6.68 (m, 1H), 7.15-7.17 (m, 2H) ppm; ^{13}C NMR (150 MHz, $CDCl_3$) δ 25.9, 26.6, 31.3, 37.5, 50.6, 112.6, 116.8, 129.2, 148.6 ppm.

***N*-(((3r,5r,7r)-adamantan-1-yl)methyl)-4-methoxyaniline 6ai**

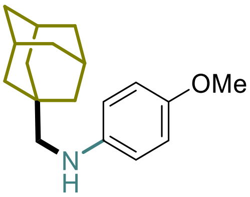

Following the **general procedure A**, 88 mg of **6ai**[19] as a white solid was obtained (yield: 65%); ^{1}H NMR (600 MHz, $CDCl_3$) δ 1.57-1.58 (m, 6H) 1.65-1.67 (m, 3H),

1.73-1.75 (m, 3H), 2.74 (s, 2H), 3.74 (s, 3H), 6.57-6.60 (m, 2H), 6.76-6.77 (m, 2H) ppm; ^{13}C NMR (150 MHz, $CDCl_3$) δ 28.4, 33.7, 37.1, 40.8, 55.9, 57.5, 113.8, 114.9, 143.6, 151.6 ppm.

***N*-(2-(4-chlorophenoxy)-2-methylpropyl)-4-methoxyaniline 6aj**

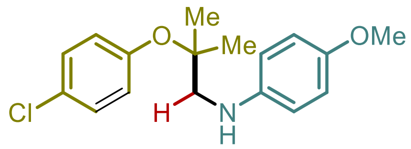

Following the **general procedure A**, 95 mg of **6aj** as a pale yellow oil was obtained (yield: 62%); ^{1}H NMR (600 MHz, $CDCl_3$) δ 1.34 (s, 6H), 3.17 (s, 2H), 3.75 (s, 3H), 3.96 (br. s, 1H), 6.63 (d, *J* = 8.8 Hz, 2H), 6.80 (d, *J* = 8.8 Hz, 2H), 6.90 (d, *J* = 8.8 Hz, 2H), 7.22 (d, *J* = 8.8 Hz, 2H) ppm; ^{13}C NMR (150 MHz, $CDCl_3$) δ 25.0, 54.2, 55.8, 80.5, 114.1, 114.9, 125.3, 129.0, 129.1, 142.6, 152.1, 153.2 ppm; HRMS (ESI) *m/z* calcd for [C17H21ClNO2]$^+$ (M + H$^+$): 306.1255; found: 306.1259.

***N*-(5-(2,5-dimethylphenoxy)-2,2-dimethylpentyl)-4-methoxyaniline 6ak**

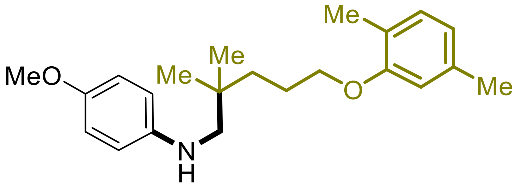

Following the **general procedure A**, 111 mg of **6al** as a white solid was obtained (yield: 65%); ^{1}H NMR (600 MHz, $CDCl_3$) δ 1.00 (s, 6 H), 1.47-1.49 (m, 2H), 1.76-1.78 (m, 2H), 2.18 (s, 3 H), 2.29 (s, 3 H), 2.89 (s, 2 H), 3.74 (s, 3 H), 3.91 (t, *J* = 6.4 Hz, 2H), 6.58-6.60 (m, 2H), 6.61 (s, 1H), 6.65 (d, *J* = 7.5 Hz, 2H), 6.76-6.78 (m, 2H), 7.00 (d, *J* = 7.5 Hz, 2H) ppm; ^{13}C NMR (150 MHz, $CDCl_3$) δ 15.8, 21.4, 24.2, 25.5, 33.9, 36.2, 55.1, 55.8, 68.4, 112.0, 113.9, 114.9, 120.6, 123.5, 130.3, 136.4, 143.4, 151.8, 157.0 ppm; HRMS (ESI) *m/z* calcd for $[C_{22}H_{32}NO2]^+$ (M + H$^+$): 342.2428; found: 342.2436.

***N*-(1-cyclohexyl-2-phenylethyl)-4-methoxyaniline 6al**

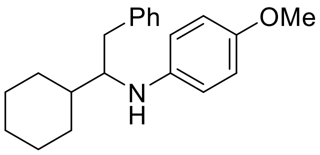

Following the **general procedure B**, except 20 mol% $Sc(OTf)_3$ was added. 33 mg of **6al** as a colorless oil was obtained (yield: 53%); ^{1}H NMR (600 MHz, $CDCl_3$) δ 1.05-1.23 (m, 5H), 1.46-1.49 (m, 1H), 1.64-1.66 (m, 1H), 1.73-1.78 (m, 3H), 1.84-1.86 (m, 1H), 2.73 (dd, J = 6.8, 13.9 Hz, 1H), 2.82 (dd, J = 6.0, 13.9 Hz, 1H), 3.26 (br.s, 1H), 3.35-3.38 (m, 1H), 3.71 (s, 3H), 6.49 (d, J = 8.9 Hz, 2H), 6.73 (d, J = 8.9 Hz, 2H), 7.15-7.18 (m, 3H), 7.23-7.26 (m, 2H) ppm; ^{13}C NMR (150 MHz, $CDCl_3$) δ 26.3, 26.4, 26.6, 28.4, 29.9, 37.3, 40.3, 55.8, 60.2, 114.4, 114.9, 126.0, 128.2, 129.2, 139.5, 142.4, 151.5 ppm; HRMS (ESI) *m/z* calcd for $[C_{21}H_{28}NO]^+$ (M + H^+): 310.2165; found: 310.2173.

***N*-(1-cyclohexyl-2-methylpropyl)-4-methoxyaniline 6am**

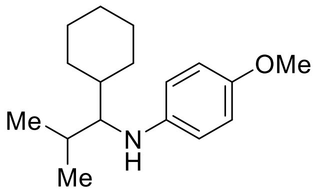

Following the **general procedure B**, except 20 mol% $Sc(OTf)_3$ was added. 19 mg of **6am** as a colorless oil was obtained (yield: 36%); ^{1}H NMR (600 MHz, $CDCl_3$) δ 0.87 (d, J = 6.6 Hz, 3H), 0.92 (d, J = 6.8 Hz, 3H), 1.09-1.26 (m, 5H), 1.43-1.46 (m, 1H), 1.62-1.66 (m, 2H), 1.70-1.72 (m, 2H), 1.79-1.81 (m, 1H), 1.85-1.91 (m, 1H), 2.83-2.85 (m, 1H), 3.12 (br.s, 1H), 3.72 (s, 3H), 6.53 (br.s, 2H), 6.72 (d, J = 8.8 Hz, 2H) ppm; ^{13}C NMR (150 MHz, $CDCl_3$) δ 17.6, 20.8, 26.4, 26.5, 28.7, 30.4, 31.2, 41.6, 55.8, 64.3, 113.2, 114.9, 144.8, 150.8 ppm; HRMS (ESI) *m/z* calcd for $[C_{21}H_{28}NO]^+$ (M + H^+): 262.2165; found: 262.2173.

***N*-(2-(benzyloxy)-1-phenylethyl)-4-bromoaniline 6an**

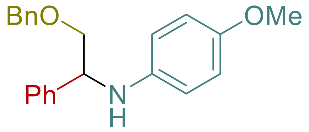

Following the **general procedure B**, 43 mg of **6am** as a colorless oil was obtained (yield: 65%); ^{1}H NMR (600 MHz, $CDCl_3$) δ 3.54-3.57 (m, 1H), 3.67 (s, 3H), 3.70 (dd, J = 4.0, 10.0 Hz, 1H), 4.41 (br.s, 1H), 4.46 (dd, J = 4.0, 8.6 Hz, 1H), 4.52 (d, J = 12.0 Hz, 1H), 4.56 (d, J = 12.0 Hz, 1H), 6.46-6.48 (m, 2H), 6.66-6.67 (m, 2H), 7.23-7.26 (m, 1H), 7.28-7.35 (m, 7H), 7.39-7.40 (m, 2H) ppm; ^{13}C NMR (150 MHz, $CDCl_3$) δ 55.7, 58.9114.6, 115.1, 126.8, 127.4, 127.7, 127.8, 128.5, 128.6, 137.8, 140.9, 141.8,

152.2 ppm; HRMS (ESI) *m/z* calcd for $[C_{22}H_{24}NO_2]^+$ (M + H$^+$): 334.1802; found: 334.1807.

***N*-(2-(benzyloxy)-1-phenylethyl)-4-bromoaniline 6ao**

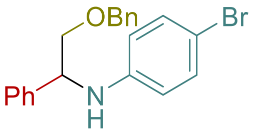

Following the **general procedure B**, 53 mg of **6an** as a colorless oil was obtained (yield: 70%); ^{1}H NMR (600 MHz, $CDCl_3$) δ 3.57 (dd, *J* = 8.6, 9.7 Hz, 1H), 3.72-3.76 (m, 1H), 4.47-4.59 (m, 3H), 4.66 (br.s, 1H), 6.36-6.39 (m, 2H), 7.12-7.16 (m, 2H), 7.25-7.36 (m, 10H) ppm; ^{13}C NMR (150 MHz, $CDCl_3$) δ 58.1, 73.0, 74.3, 109.4, 115.5, 126.7, 127.6, 127.7, 127.9, 128.5, 128.7, 131.7, 137.6, 140.0, 146.5 ppm; HRMS (ESI) *m/z* calcd for $[C_{21}H_{21}BrNO]^+$ (M + H$^+$): 382.0801; found: 382.0803.

***N*- (2-(benzyloxy)-1-phenylethyl)-4-chloroaniline 6ap**

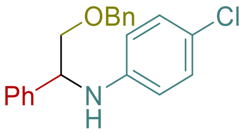

Following the **general procedure B**, 46 mg of **6ao** as a colorless oil was obtained (yield: 68%); ^{1}H NMR (600 MHz, $CDCl_3$) δ 3.56 (dd, *J* = 8.7, 9.8 Hz, 1H), 3.72 (dd, *J* = 4.0, 9.8 Hz, 1H), 4.48 (dd, *J* = 4.0, 8.7 Hz, 1H), 4.51 (d, *J* = 11.9 Hz, 1H), 4.56 (d, *J* = 11.9 Hz, 1H), 4.63 (br.s, 1H), 6.41 (d, *J* = 8.8 Hz, 2H), 6.99 (d, *J* = 8.8 Hz, 2H), 7.24-7.36 (m, 10H) ppm; ^{13}C NMR (150 MHz, $CDCl_3$) δ 58.2, 73.0, 74.3, 115.0, 122.3, 126.7, 127.6, 127.7, 127.9, 128.5, 128.7, 128.8, 137.6, 140.1, 146.1 ppm; HRMS (ESI) *m/z* calcd for $[C_{21}H_{21}ClNO]^+$ (M + H$^+$): 338.1306; found: 338.1313.

***N*-(4-((2-(benzyloxy)-1-phenylethyl)amino)phenyl)acetamide 6aq**

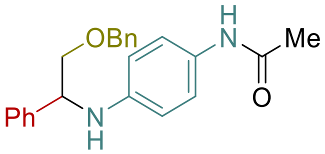

Following the **general procedure B**, 51 mg of **6ap** as a colorless oil was obtained (yield: 71%); ^{1}H NMR (600 MHz, $CDCl_3$) δ 2.05 (s, 3H), 3.55-3.59 (m, 1H), 3.72 (dd, *J* = 4.0, 10.0 Hz, 1H), 4.49-4.57 (m, 4H), 6.44 (dd, *J* = 8.8 Hz, 2H), 7.13 (dd, *J* = 8.8

Hz, 2H), 7.28-7.37 (m, 10H) ppm; ^{13}C NMR (150 MHz, $CDCl_3$) δ 24.1, 58.3, 72.9, 74.4, 114.1, 122.1, 126.7, 127.4, 127.7, 127.8, 128.3, 128.5, 128.6, 137.7, 140.5, 144.8, 168.1 ppm; HRMS (ESI) *m/z* calcd for $[C_{23}H_{25}N_2O_2]^+$ (M + H^+): 361.1911; found: 361.1912.

_N_-(2-(benzyloxy)-1-phenylethyl)-4-(4,4,5,5-tetramethyl-1,3,2-dioxaborolan-2-yl)aniline 6ar

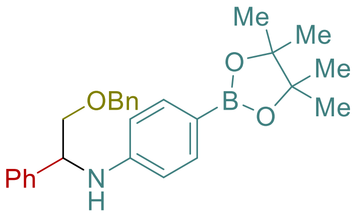

Following the **general procedure B**, 70 mg of **6aq** as a white solid was obtained (yield: 78%); ^{1}H NMR (600 MHz, $CDCl_3$) δ 1.27 (s, 6H), 1.28 (s, 6H), 3.61 (dd, J = 8.0, 10.0 Hz, 1H), 3.75 (dd, J = 4.1, 10.0 Hz, 1H), 4.51 (d, J = 12.0 Hz, 1H), 4.56 (d, J = 12.0 Hz, 1H), 4.59 (dd, J = 4.1, 8.0 Hz, 1H), 4.82 (br.s, 1H), 6.48 (d, J = 8.5 Hz, 2H), 7.22-7.36 (m, 10H), 7.53 (d, J = 8.5 Hz, 2H) ppm; ^{13}C NMR (150 MHz, $CDCl_3$) δ 24.7, 24.8, 57.4, 72.9, 74.2, 83.1, 112.9, 126.7, 127.4, 127.7, 127.8, 128.5, 128.6, 136.1, 137.7, 140.3, 149.9 ppm; HRMS (ESI) *m/z* calcd for $[C_{27}H_{33}BNO_3]^+$ (M + H^+): 430.2548; found: 430.2555.

_N_-(2-(benzyloxy)-1-phenylethyl)-3-methoxyaniline 6as

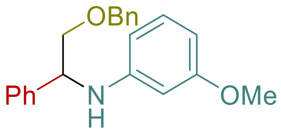

Following the **general procedure B**, 39 mg of **6as** as a colorless oil was obtained (yield: 58%); ^{1}H NMR (600 MHz, $CDCl_3$) δ 3.57 (dd, J = 8.5, 9.9 Hz, 1H), 3.65 (s, 3H), 3.72 (dd, J = 4.1, 9.9 Hz, 1H), 4.50-4.58 (m, 3H), 4.64 (br.s, 1H), 6.06-6.07 (m, 1H), 6.14 (dd, J = 1.8, 8.0 Hz, 1H), 6.22 (dd, J = 2.2, 8.0 Hz, 1H), 6.96-6.99 (m, 1H), 7.23-7.28 (m, 1H), 7.29-7.35 (m, 7H), 7.37-7.39 (m, 2H) ppm; ^{13}C NMR (150 MHz, $CDCl_3$) δ 54.9, 58.1, 72.9, 74.3, 99.9, 102.9, 106.9, 126.7, 127.4, 127.7, 127.8, 128.4, 128.6, 129.7, 137.7, 140.6, 149.0, 160.5 ppm; HRMS (ESI) *m/z* calcd for $[C_{22}H_{24}NO_2]^+$ (M + H^+): 334.1802; found: 334.1807.

Methyl 3-((2-(benzyloxy)-1-phenylethyl)amino)benzoate 6at

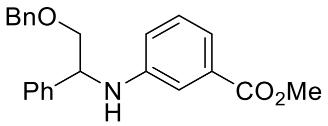

Following the **general procedure B**, 57 mg of **6at** as a colorless oil was obtained (yield: 79%); ^{1}H NMR (600 MHz, $CDCl_3$) δ 3.59 (dd, J = 8.2, 9.9 Hz, 1H), 3.74 (dd, J = 4.0, 9.9 Hz, 1H), 3.83 (s, 3H), 4.52-4.58 (m, 3H), 4.76 (s, 1H), 6.63 (ddd, J = 0.8, 2.5, 3.3 Hz, 1H), 7.08-7.11 (m, 1H), 7.25-7.26 (m, 2H), 7.28-7.35 (m, 8H), 7.37-7.39 (m, 2H) ppm; ^{13}C NMR (150 MHz, $CDCl_3$) δ 51.9, 58.0, 73.0, 74.2, 115.0, 117.8, 118.7, 126.7, 127.6, 127.7, 127.9, 128.5, 128.7, 129.0, 130.7, 137.6, 140.1, 147.5, 167.4 ppm; HRMS (ESI) *m/z* calcd for $[C_{23}H_{24}NO_3]^+$ (M + H$^+$): 362.1751; found: 362.1749.

2-(Benzyloxy)-*N*-(2-(benzyloxy)-1-phenylethyl)aniline 6au

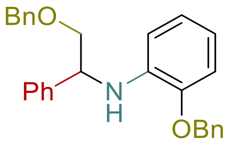

Following the **general procedure B**, 56 mg of **6au** as a colorless oil was obtained (yield: 68%); ^{1}H NMR (600 MHz, $CDCl_3$) δ 3.61 (dd, J = 8.7, 9.8 Hz, 1H), 3.72 (dd, J = 4.2, 9.8 Hz, 1H), 4.50-4.57 (m, 3H), 5.11 (d, J = 11.7 Hz, 1H), 5.14 (d, J = 11.7 Hz, 1H), 5.34 (s, 1H), 6.30 (dd, J = 1.5, 7.9 Hz, 1H), 6.58-6.61 (m, 1H), 6.65-6.68 (m, 1H), 6.83 (dd, J = 1.3, 7.9 Hz, 1H), 7.22-7.25 (m, 6H), 7.29-7.35 (m, 5H), 7.39-7.40 (m, 2H), 7.45-7.47 (m, 2H) ppm; ^{13}C NMR (150 MHz, $CDCl_3$) δ 58.3, 70.5, 72.9, 74.5, 111.2, 111.8, 116.7, 121.4, 126.8, 127.3, 127.4, 127.5, 127.6, 127.8, 128.4, 128.5, 128.6, 137.3, 137.9, 138.0, 140.9, 146.3 ppm; HRMS (ESI) *m/z* calcd for $[C_{28}H_{28}NO_2]^+$ (M + H$^+$): 410.2115; found: 410.2111.

***N*-(2-(benzyloxy)-1-phenylethyl)-2,3-dihydrobenzo[b][1,4]dioxin-6-amine 6av**

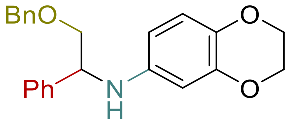

Following the **general procedure B**, 58 mg of **6at** as a white solid was obtained (yield: 80%); ^{1}H NMR (600 MHz, $CDCl_3$) δ 3.61-3.64 (m, 1H), 3.77 (dd, J = 4.0, 10.0 Hz, 1H), 4.17-4.22 (m, 4H), 4.52 (dd, J = 4.0, 8.7 Hz, 1H), 4.58 (d, J = 12.0 Hz,

1H), 4.63 (d, J = 12.0 Hz, 1H), 6.13-6.18 (m, 2H), 6.68 (d, J = 8.6 Hz, 1H), 7.30-7.33 (m, 1H), 7.36-7.42 (m, 7H), 7.45-7.46 (m, 2H) ppm; ^{13}C NMR (150 MHz, $CDCl_3$) δ 58.9, 64.1, 64.6, 72.8, 74.3, 102.8, 107.9, 117.3, 126.8, 127.4, 127.7, 127.8, 128.4, 128.6, 135.9, 137.7, 140.5, 142.3, 143.7 ppm; HRMS (ESI) *m/z* calcd for $[C_{23}H_{24}NO_3]^+$ (M + H^+): 362.1751; found: 362.1752.

_N_-(2-(benzyloxy)-1-phenylethyl)-5-bromo-2-methoxyaniline 6aw

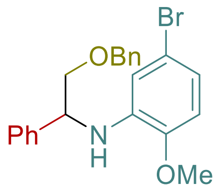

Following the **general procedure B**, 50 mg of **6aw** as a colorless oil was obtained (yield: 61%); ^{1}H NMR (600 MHz, $CDCl_3$) δ 3.62 (dd, J = 8.3, 10.0 Hz, 1H), 3.74 (dd, J = 4.2, 10.0 Hz, 1H), 3.86 (s, 3H), 4.48 (dd, J = 4.2, 8.3 Hz, 1H), 4.53 (d, J = 12.1 Hz, 1H), 4.57 (d, J = 12.1 Hz, 1H), 6.37 (d, J = 2.3 Hz, 1H), 6.58 (d, J = 8.4 Hz, 1H), 6.70 (dd, J = 8.3, 10.0 Hz, 1H), 7.26-7.37 (m, 10H) ppm; ^{13}C NMR (150 MHz, $CDCl_3$) δ 55.6, 57.9, 72.8, 74.2, 110.4, 113.6, 113.9, 119.0, 126.7, 127.5, 127.6, 127.7, 128.4, 128.7, 137.8, 138.8, 140.0, 146.2 ppm; HRMS (ESI) *m/z* calcd for $[C_{22}H_{23}BrNO_2]^+$ (M + H^+): 412.0907; found: 412.0914.

_N_ -(2-(benzyloxy)-1-phenylethyl)-3,5-dimethoxyaniline 6ax

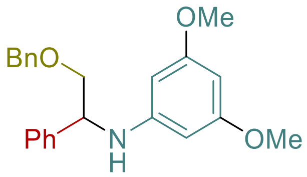

Following the **general procedure B**, 51 mg of **6ax** as a colorless oil was obtained (yield: 70%); ^{1}H NMR (600 MHz, $CDCl_3$) δ 3.56-3.59 (m, 1H), 3.64 (s, 6H), 3.71-3.73 (m, 1H), 4.51-4.58 (m, 3H), 4.64 (br.s, 1H), 5.71 (d, J = 2.0 Hz, 2H), 5.83-5.84 (m, 1H), 7.23-7.25 (m, 1H), 7.28-7.38 (m, 10H) ppm; ^{13}C NMR (150 MHz, $CDCl_3$) δ 55.0, 58.1, 72.9, 74.3, 90.1, 92.8, 126.7, 127.4, 127.7, 127.8, 128.5, 128.6, 137.7, 140.6, 149.5, 161.4 ppm; HRMS (ESI) *m/z* calcd for $[C_{23}H_{26}NO_3]^+$ (M + H^+): 364.1907; found: 364.1910.

_N_-(2-(benzyloxy)-1-phenylethyl)benzofuran-5-amine 6ay

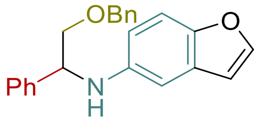

Following the **general procedure B**, 52 mg of **6ay** as a colorless oil was obtained (yield: 76%); ^{1}H NMR (600 MHz, $CDCl_3$) δ 3.61 (dd, J = 8.5, 9.9 Hz, 1H), 3.75 (dd, J = 4.0, 9.9 Hz, 1H), 4.52-4.60 (m, 3H), 6.71 (dd, J = 2.2, 8.6 Hz, 1H), 6.78 (d, J = 2.2 Hz, 1H), 7.01 (d, J = 5.4 Hz, 1H), 7.24-7.35 (m, 9H), 7.41-7.43 (m, 2H), 7.54-7.56 (m, 1H) ppm; ^{13}C NMR (150 MHz, $CDCl_3$) δ 58.5, 72.9, 74.4, 106.4, 114.6, 122.6, 123.4, 126.6, 126.8, 127.4, 127.7, 127.8, 128.5, 128.6, 129.6, 137.7, 140.5, 140.7, 145.0 ppm; HRMS (ESI) *m/z* calcd for $[C_{23}H_{22}NO_2]^+$ (M + H^+): 344.1645; found: 344.1656.

***N*-(4-((2-(benzyloxy)-1-phenylethyl)amino)-2-phenoxyphenyl) methanesulfonamide 6az**

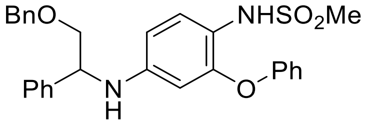

Following the **general procedure B**, 69 mg of **6az** as a white solid was obtained (yield: 71%); ^{1}H NMR (600 MHz, $CDCl_3$) δ 2.85 (s, 3H), 3.51 (dd, J = 8.5, 10.0 Hz, 1H), 3.67 (dd, J = 4.0, 10.0 Hz, 1H), 4.36 (dd, J = 4.0, 8.5 Hz, 1H), 4.49 (d, J = 12.0 Hz, 1H), 4.52 (d, J = 12.0 Hz, 1H), 4.67 (s, 1H), 5.95 (d, J = 2.5 Hz, 1H), 6.22 (s, 1H), 6.28 (dd, J = 2.5, 8.8 Hz, 1H), 6.82-6.84 (m, 2H), 7.11-7.13 (m, 1H), 7.22-7.29 (m, 11H), 7.31-7.33 (m, 2H) ppm; ^{13}C NMR (150 MHz, $CDCl_3$) δ 38.8, 58.2, 73.0, 74.1, 103.1, 109.5, 116.6, 118.7, 124.0, 126.6, 127.1, 127.5, 127.7, 127.9, 128.5, 128.7, 130.0, 137.5, 139.7, 147.2, 150.3, 155.5 ppm; HRMS (ESI) *m/z* calcd for $[C_{28}H_{29}N_2O_4S]^+$ (M + H^+): 489.1843; found: 489.1842.

***N*-(1-phenylethyl-2,2,2-d3)aniline 7**

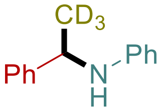

Imine **4a** (18 mg, 0.1 mmol), AcOH-d4 (0.2 mL), $Sc(OTf)_2$ (0.12 mmol, 59 mg, 1.2 eq.), TiO_2 P25 (40 mg) were added to an oven dried 10 mL quartz reaction tube. The reaction tube was then evacuated and backfilled with N_2 three times, and 3 mL of degassed anhydrous MeCN was added. The reaction mixture was irradiated using

Kessil lamp (PR-160, 390 nm) and cooled with a fan for 48 h. The two parallel reactions were performed. After reaction, the two reaction mixtures were combined and filtered through diatomite and a small amount of silica gel. The filtrate was concentrated under reduced pressure. The sat. $NaHCO_3$ (10 mL) was added to residue and the mixture was extracted by CH_2Cl_2 (10 mL ×3). Combined organic layers were dried over Na_2SO_4 and concentrated. The residue was purified by column chromatography on silica gel to furnish the product **5a** (25 mg, 62%), as a pale yellow oil. ^{1}H NMR (600 MHz, $CDCl_3$) δ 4.00 (br. s, 1H), 4.45 (s, 1H), 6.49-6.50 (m, 2H), 6.62-6.64 (m, 1H), 7.06-7.09 (m, 2H), 7.19-7.22 (m, 1H), 7.28-7.36 (m, 4H) ppm; ^{13}C NMR (150 MHz, $CDCl_3$) δ 24.2, 53.2, 113.2, 117.2, 125.8, 126.8, 128.6, 129.0, 145.2, 147.2 ppm; HRMS (ESI) *m/z* calcd for $[C_{14}H_{12}D_3NNa]^+$ (M + Na^+): 223.1285; found: 223.1280.

_N_-(1-phenyl-2-(m-tolyl)ethyl-2,2-d2)aniline 8

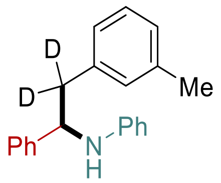

Following the **general procedure A**, except the deuterated carboxylic acid **3q-d2** used, 110 mg of **8** as a colorless oil was obtained (yield: 76%, 98D%); ^{1}H NMR (600 MHz, $CDCl_3$) δ 2.30 (s, 3H), 4.14 (br. s, 1H), 4.54 (s, 1H), 6.43-6.45 (m, 2H), 6.60-6.62 (m, 1H), 6.92-6.94 (m, 2H), 7.02-7.05 (m, 3H), 7.14-7.17 (m, 1H), 7.22-7.24 (m, 1H), 7.29-7.34 (m, 4H) ppm; ^{13}C NMR (150 MHz, $CDCl_3$) δ 21.4, 59.1, 113.7, 117.4, 126.1, 126.4, 127.0, 127.5, 128.4, 128.5, 128.9, 130.0, 137.5, 138.1, 143.6, 147.3 ppm; HRMS (ESI) *m/z* calcd for $[C_{21}H_{20}D_2N]^+$ (M + H^+): 290.1872; found: 290.1870.

4-Methoxy-_N_-(1-phenyl-2-(m-tolyl)ethyl-2,2-d2)aniline 9

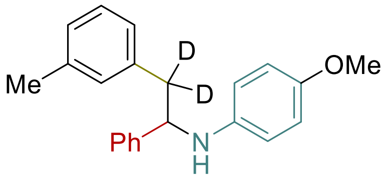

Following the **general procedure B**, except the deuterated carboxylic acid **3q-d2** used, 45 mg of **9** as a colorless oil was obtained (yield: 71%, 98D%); ^{1}H NMR (600

MHz, $CDCl_3$) δ 2.30 (s, 3H), 3.65 (s, 3H), 4.46 (s, 1H), 6.39-6.40 (m, 2H), 6.62-6.64 (m, 2H), 6.92-6.94 (m, 2H), 7.02-7.03 (m, 1H), 7.14-7.16 (m, 1H), 7.23-7.24 (m, 1H), 7.29-7.34 (m, 4H) ppm; ^{13}C NMR (150 MHz, $CDCl_3$) δ 21.4, 55.6, 59.9, 114.6, 114.9, 126.1, 126.4, 126.9, 127.4, 128.4, 128.5, 130.0, 137.6, 138.1, 141.6, 143.8, 152.0 ppm; HRMS (ESI) *m/z* calcd for $[C_{22}H_{22}D_2NO]^+$ (M + H^+): 320.1978; found: 320.1982.

***N*-(2-(2-bromophenyl)-1-phenylethyl-2,2-d2)aniline 10**

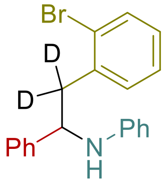

Following the **general procedure A**, except the deuterated carboxylic acid **3p-d2** used, 130 mg of **10** as a white solid was obtained (yield: 74%, 98D%); ^{1}H NMR (600 MHz, $CDCl_3$) δ 4.27 (s, 1H), 4.69 (s, 1H), 6.45-6.47 (m, 2H), 6.59-6.62 (m, 1H), 7.02-7.04 (m, 2H), 7.06-7.09 (m, 2H), 7.16-7.19 (m, 1H), 7.22-7.23 (m, 1H), 7.29-7.32 (m, 2H), 7.37-7.38 (m, 2H), 7.55-7.56 (m, 1H) ppm; ^{13}C NMR (150 MHz, $CDCl_3$) δ 58.0, 113.5, 117.4, 124.9, 126.3, 127.1, 127.4, 128.4, 128.6, 129.0, 131.3, 133.0, 137.5, 143.3, 147.2 ppm; HRMS (ESI) *m/z* calcd for $[C_{20}H_{17}D_2BrN]^+$ (M + H^+): 354.0821, 356.0800; found: 354.0831, 356.0814.

***N*-(2-([1,1'-biphenyl]-4-yl)-1-phenylethyl-2,2-d2)aniline 11**

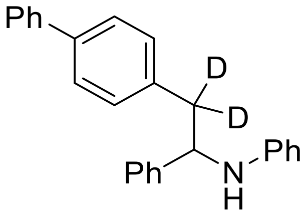

Following the **general procedure A**, except the deuterated carboxylic acid **3s-d2** used, 114 mg of **11** as a white solid was obtained (yield: 65%, 98D%); ^{1}H NMR (600 MHz, $CDCl_3$) δ 4.14 (br. s, 1H), 4.61 (s, 1H), 6.47 (d, J = 7.8 Hz, 2H), 6.61-6.64 (m, 1H), 7.04-7.06 (m, 2H), 7.17-7.18 (m, 2H), 7.22-7.24 (m, 1H), 7.29-7.35 (m, 5H), 7.40-7.43 (m, 2H), 7.49-7.50 (m, 2H), 7.56-7.57 (m, 2H) ppm; ^{13}C NMR (150 MHz, $CDCl_3$) δ 59.0, 113.6, 117.5, 126.4, 127.0, 127.1, 127.2, 128.6, 128.7, 129.0, 129.6, 136.6, 139.6, 140.7, 143.3, 147.2 ppm; HRMS (ESI) *m/z* calcd for $[C_{26}H_{22}D_2N]^+$ (M + H^+): 352.2029; found: 352.2030.

N-(2-(benzyloxy)-1-(4-bromophenyl)ethyl-1-d)-4-methylaniline 12

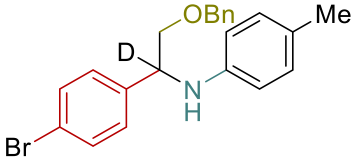

Following the **general procedure B**, except the deuterated aldyhyde **1ai-d1** used, 49 mg of **12** as a colorless oil was obtained (yield: 62%); ^{1}H NMR (600 MHz, $CDCl_3$) δ 2.18 (s, 3H), 3.52 (d, J = 9.9 Hz, 1H), 3.68 (d, 9.9 Hz, 1H), 4.49-4.56 (m, 3H), 6.39-6.40 (m, 2H), 6.88 (d, J = 8.2 Hz, 2H), 7.24-7.30 (m, 5H), 7.32-7.34 (m, 2H), 7.41-7.43 (m, 2H), ppm; ^{13}C NMR (150 MHz, $CDCl_3$) δ 20.3, 57.4 (t, 21.0 Hz), 57.8, 73.0, 74.0, 114.0, 121.0, 127.1, 127.7, 127.8, 128.5, 128.6, 129.5, 131.7, 137.6, 139.9, 144.9 ppm; HRMS (ESI) m/z calcd for $[C_{22}H_{22}DBrNO]^+$ (M + H^+): 397.1020; found: 397.1022.

N-(2-((4-methoxyphenyl)amino)-2-(3,4,5-trimethoxyphenyl)ethyl-2-d)acetamide 13

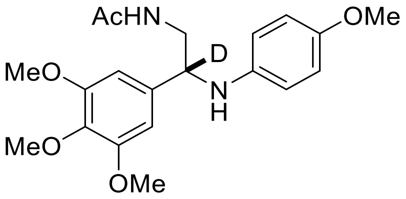

Following the **general procedure B**, except the deuterated aldyhyde **1r-d1** used, 55 mg of **13** as a white solid was obtained (yield: 73%, 98%D); ^{1}H NMR (600 MHz, $CDCl_3$) δ 2.00 (s, 3H), 3.51 (dd, J = 6.2, 14.1 Hz, 1H), 3.58 (dd, J = 6.3, 14.1 Hz, 1H), 3.70 (s, 3H), 3.82 (s, 9H), 5.89 (br. s, 1H), 6.49 (d, J = 8.8 Hz, 2H), 6.59 (s, 2H), 6.70 (d, J = 8.8 Hz, 2H) ppm; ^{13}C NMR (150 MHz, $CDCl_3$) δ 23.2, 46.2, 55.7, 56.1, 60.7, 103.1, 114.5, 114.7, 137.0, 137.1, 141.5, 152.0, 153.5, 171.4 ppm; HRMS (ESI) m/z calcd for $[C_{20}H_{25}DN_2O_5Na]^+$ (M + Na^+): 398.1797; found: 398.1796.

4-Methoxy-N-(2-(m-tolyl)-1-(3,4,5-trimethoxyphenyl)ethyl-1,2,2-d3)aniline 14

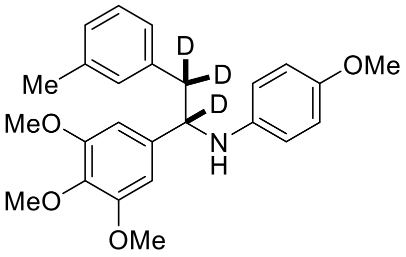

Following the **general procedure B**, except the deuterated aldyhyde **1r-d1** and deuterated carboxylic acid **3q-d2** used, 57 mg of **14** as a white solid was obtained (yield: 69%); ^{1}H NMR (600 MHz, $CDCl_3$) δ 2.30 (s, 3H), 3.68 (s, 3H), 3.79 (s, 6H), 3.83 (s, 3H), 6.42 (d, J = 8.9 Hz, 2H), 6.53 (s, 2H), 6.66 (d, J = 8.9 Hz, 2H), 6.92-6.94 (m, 2H), 7.03-7.04 (m, 1H), 7.16-7.18 (m, 1H) ppm; ^{13}C NMR (150 MHz, $CDCl_3$) δ 21.3, 55.6, 56.0, 60.8, 103.1, 114.5, 115.0, 126.2, 127.4, 128.4, 130.0, 136.6, 137.5, 138.1, 139.6, 141.7, 152.1, 153.3 ppm; HRMS (ESI) *m/z* calcd for $[C_{25}H_{26}D_3NO_4Na]^+$ (M + Na^+): 433.2177; found: 433.2168.

***N*-(2-(benzyloxy)-1-(phenyl-d5)ethyl)-4-methylaniline 15**

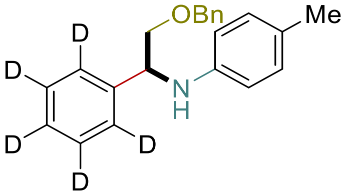

Following the **general procedure B**, except the deuterated aldyhyde **1a-d5** used, 46 mg of **15** as a colorless oil was obtained (yield: 72%); ^{1}H NMR (600 MHz, $CDCl_3$) δ 2.17 (s, 3H), 3.57 (dd, J = 8.6, 9.9 Hz, 1H), 3.72 (dd, J = 4.1, 9.9 Hz, 1H), 4.50-4.52 (m, 2H), 4.57 (d, J = 12.0 Hz, 1H), 6.43 (d, J = 8.4 Hz, 2H), 6.87 (d, J = 8.4 Hz, 2H), 7.27-7.29 (m, 3H), 7.32-7.35 (m, 2H) ppm; ^{13}C NMR (150 MHz, $CDCl_3$) δ 20.3, 58.3, 72.9, 74.5, 114.0, 126.8, 127.7, 127.8, 128.4, 129.5, 137.8, 140.6, 145.3 ppm; HRMS (ESI) *m/z* calcd for $[C_{22}H_{19}D_5NO]^+$ (M + H^+): 323.2166; found: 323.2161.

***N*-(2-(benzyloxy)-1-phenylethyl)benzen-d5-amine 16**

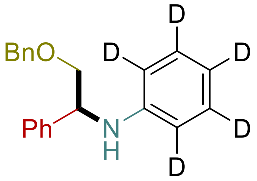

Following the **general procedure B**, except the deuterated amine **2q** used, 42 mg of **16** as a colorless oil was obtained (yield: 68%); ^{1}H NMR (600 MHz, $CDCl_3$) δ 3.57-3.60 (m, 1H), 3.73 (dd, J = 4.1, 9.9 Hz, 1H), 4.51-4.58 (m, 3H), 4.62 (br. s, 1H), 7.22-7.35 (m, 8H), 7.39-7.40 (m, 2H) ppm; ^{13}C NMR (150 MHz, $CDCl_3$) δ 58.1, 72.9, 74.4, 126.8, 127.4, 127.7, 127.8, 128.5, 128.6, 137.7, 140.6, 147.5 ppm; HRMS (ESI) *m/z* calcd for $[C_{21}H_{17}D_5NO]^+$ (M + H^+): 309.2010; found: 309.2018.

***tert*-butyl (R)-(2-((4-methoxyphenyl)amino)-2-(3,4,5-trimethoxyphenyl)ethyl-2-d)carbamate 17**

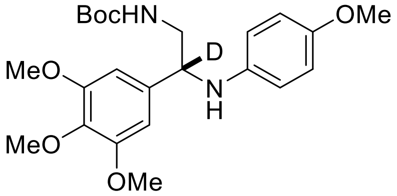

Following the **general procedure B**, except the deuterated aldyhyde **1r-d1** used, 75 mg of **17** as a white solid was obtained (yield: 87%, 98%D); ^{1}H NMR (600 MHz, $CDCl_3$) δ 1.46 (s, 9H), 3.36 (dd, J = 6.1, 14.2 Hz, 1H), 3.58 (dd, J = 6.8, 14.2 Hz, 1H), 3.70 (s, 3H), 3.83 (s, 9H), 4.89 (br. s, 1H), 6.47 (d, J = 8.8 Hz, 2H), 6.60 (s, 2H), 6.70 (d, J = 8.8 Hz, 2H) ppm; ^{13}C NMR (150 MHz, $CDCl_3$) δ 28.3, 47.2, 55.7, 56.0, 60.7, 79.9, 103.1, 114.4, 114.7, 136.9, 137.3, 141.7, 152.0, 153.5, 157.0 ppm; HRMS (ESI) *m/z* calcd for $[C_{23}H_{31}DN_2O_6Na]^+$ (M + Na^+): 456.2215; found: 456.2214.

***N*^{1}-(4-methoxyphenyl)-1-(3,4,5-trimethoxyphenyl)ethane-1-d-1,2-diamine 18**

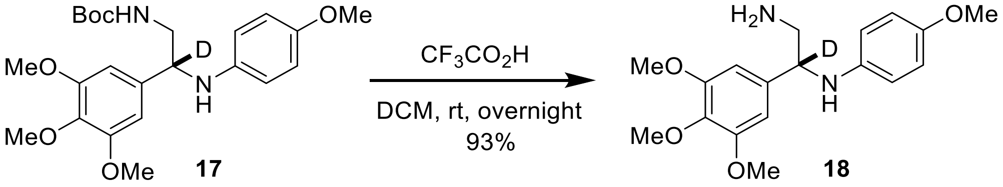

To a solution of deuterated amine **17** (87 mg, 0.2 mmol) in DCM (3 mL) was added CF_3CO_2H (0.5 mL). The mixture was stirred at room temperature for overnight. After reaction, the mixture was concentrated under reduced pressure. The sat. $NaHCO_3$ (10 mL) was added to residue and the mixture was extracted by CH2Cl2 (5 mL×3). Combined organic layers were dried over Na_2SO_4 and concentrated. The residue was purified by column chromatography on silica gel (eluent: ethyl acetate/ hexane =1/ 2) to give the deuterated amine **18** (62 mg, 93%, 98D%) as a colorless oil; ^{1}H NMR (600 MHz, $CDCl_3$) δ 2.60 (br. s, 2H), 2.93 (d, J = 12.6 Hz, 1H), 3.07 (d, J = 12.6 Hz, 1H), 3.69 (s, 3H), 3.82 (s, 9H), 6.54 (d, J = 8.9 Hz, 2H), 6.58 (s, 2H), 6.68 (d, J = 8.9 Hz, 2H) ppm; ^{13}C NMR (150 MHz, $CDCl_3$) δ 48.1, 55.6, 56.1, 60.7, 103.3, 114.7, 115.0, 136.9, 137.5, 141.6, 152.1, 153.4 ppm; HRMS (ESI) *m/z* calcd for $[C_{18}H_{24}DN_2O_4]^+$ (M + H^+): 334.1872; found: 334.1873.

4,4-dimethyl-1,5-diphenylpyrrolidin-2-one 19

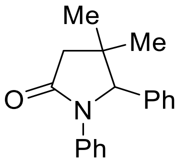

Following the **general procedure A**, 74 mg of **19** as a white solid was obtained (yield: 56%); ^{1}H NMR (600 MHz, $CDCl_3$) δ 0.73 (s, 3H), 1.37 (s, 3H), 2.37 (d, J = 16.7 Hz, 1H), 2.63(d, J = 16.7 Hz, 1H), 4.74 (s, 1H), 7.04-7.06 (m, 1H), 7.15-7.16 (m, 2H), 7.22-7.28 (m, 3H), 7.30-7.33 (m, 2H), 7.43-7.44 (m, 2H) ppm; ^{13}C NMR (150 MHz, $CDCl_3$) δ 24.6, 29.6, 37.4, 45.7, 74.5, 121.9, 124.8, 127.9, 128.6, 137.8, 138.8, 174.1 ppm; HRMS (ESI) *m/z* calcd for $[C_{18}H_{20}NO]^+$ (M + H^+): 266.1539; found: 266.1542.

1,2-Diphenylpiperidine 20

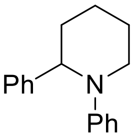

Following the **general procedure A**, 69 mg of known compound **20**[20] as a colorless oil was obtained (yield: 58%); ^{1}H NMR (600 MHz, $CDCl_3$) δ 1.53-1.58 (m, 1H), 1.65-1.70 (m, 1H), 1.74-1.77 (m, 2H), 1.91-2.02 (m, 2H), 3.23-3.27 (m, 1H), 3.38-3.42 (m, 1H), 4.49 (dd, J = 4.5, 6.7 Hz, 1H), 6.73-6.76 (m, 1H), 6.88-6.89 (m, 2H), 7.11-7.14 (m, 3H), 7.20-7.23 (m, 2H), 7.25-7.27 (m, 2H) ppm; ^{13}C NMR (150 MHz, $CDCl_3$) δ 22.0, 25.6, 33.4, 50.5, 61.0, 118.7, 119.5, 126.2, 127.2, 128.2, 128.7, 143.5, 151.8 ppm

4,5-Diphenylpiperazin-2-one 21

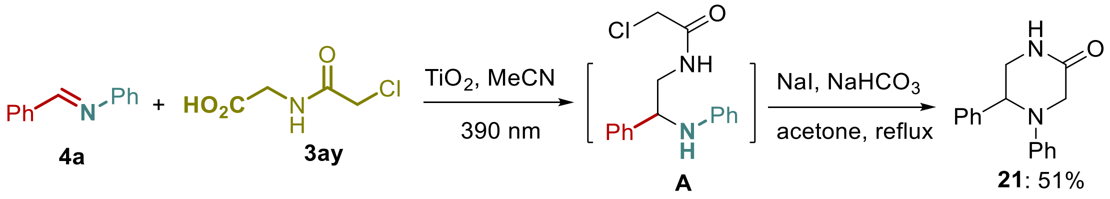

Imine **4a** (91 mg, 0.5 mmol), carboxylic acid **3ay** (151 mg, 1.0 mmol, 2.0 equiv.), TiO_2 P25 (100 mg) were added to an oven dried 10 mL quartz reaction tube. The reaction tube was then evacuated and backfilled with N_2 three times, and 6 mL of degassed anhydrous MeCN was added. The reaction mixture was irradiated using Kessil lamp (PR-160, 390 nm) and cooled with a fan for 48 h. After reaction, the mixture was filtered through diatomite and a small amount of silica gel. The filtrate

was concentrated under reduced pressure. The sat. $NaHCO_3$ (10 mL) was added to residue and the mixture was extracted by CH_2Cl_2 (10 mL×3). Combined organic layers were dried over Na_2SO_4 and concentrated. The residue was used for the next step without further purification.

To a solution of above residue in acetone (4 mL), $NaHCO_3$ (84 mg, 1.0 mmol) and NaI (38 mg, 0.25 mmol) were added and stirred at 70 ℃ for three days. After the reaction was complete, acetone was evaporated in vacuo. The obtained residue was diluted with water, then extracted with CH_2Cl_2 (10 mL×3). Combined organic layers were dried over Na_2SO_4 and concentrated. The resultant crude was purified by column chromatography to give the title compound **21** (64 mg, 51%) as a white solid; ^{1}H NMR (600 MHz, $CDCl_3$) δ 3.61 (ddd, J = 3.3, 5.1, 12.9 Hz, 1H), 3.90 (ddd, J = 1.7, 4.2, 9.7, 12.9 Hz, 1H), 4.03 (d, J = 16.8 Hz, 1H), 4.10 (d, J = 16.8 Hz, 1H), 4.93 (br. t, J = 3.3 Hz, 1H), 6.70-6.71 (m, 2H), 6.78-6.81 (m, 1H), 6.93 (br. s, 1H), 7.20-7.23 (m, 4H), 7.24-7.26 (m, 1H), 7.28-7.31 (m, 2H) ppm; ^{13}C NMR (150 MHz, $CDCl_3$) δ 46.1, 49.8, 56.8, 113.7, 118.6, 126.5, 127.6, 128.8, 129.3, 139.5, 147.4, 170.0 ppm; HRMS (ESI) *m/z* calcd for $[C_{16}H_{16}N_2NaO]^+$ (M + H^+): 275.1155; found: 275.1163.

4,5-Diphenylpiperazin-2-one 22

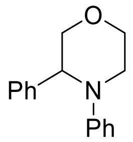

Following the method for synthesizing compound **21**, 63 mg of **22** as a white solid was obtained (yield: 56%);^{1}H NMR (600 MHz, $CDCl_3$) δ 3.11-3.15 (m, 1H), 3.44 (dt, J = 3.4, 12.5 Hz, 1H), 3.67 (dd, J = 8.1, 11.5 Hz, 1H), 3.94-3.99 (m, 3H), 4.34 (d, J = 3.6, 8.1 Hz, 1H), 6.83-6.85 (m, 1H), 6.91-6.93 (m, 2H), 7.12-7.15 (m, 3H), 7.19-7.21 (m, 2H), 7.29-7.30 (m, 2H) ppm; ^{13}C NMR (150 MHz, $CDCl_3$) δ 52.9, 61.6, 67.6, 73.2, 121.1, 121.7, 127.1, 127.8, 128.3, 128.7, 139.3, 151.0 ppm; HRMS (ESI) *m/z* calcd for $[C_{16}H_{18}NO]^+$ (M + H^+):240.1383; found: 240.1391.

2-Methyl-1,5-diphenyl-4,5-dihydro-1H-imidazole 23

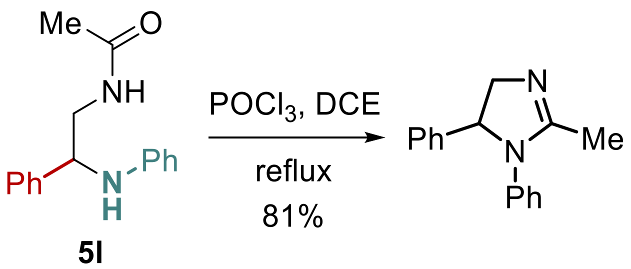

To a solution of deuterated amine **5l** (127 mg, 0.5 mmol) in DCE (5 mL) was added $POCl_3$ (0.5 mmol, 1.0 equiv.). The mixture was refluxed for overnight. Upon cooling, the solution was extracted with sat. $NaHCO_3$, H_2O, and brine. Combined organic layers were dried over Na_2SO_4 and concentrated. The residue was purified by column chromatography on silica gel (eluent: ethyl acetate/ hexane =1/ 10) to give the compound **23** (95.6 mg, 81%) as a colorless oil; ^{1}H NMR (600 MHz, $CDCl_3$) δ 2.16 (s, 3H), 3.83 (dd, J = 9.5, 13.4 Hz, 1H), 4.39-4.43 (m, 1H), 5.23-5.26 (m, 1H), 7.01-7.03 (m, 2H), 7.16-7.18 (m, 1H), 7.26-7.32 (m, 7H) ppm; ^{13}C NMR (150 MHz, $CDCl_3$) δ 14.9, 59.1, 68.6, 125.5, 126.8, 127.1, 128.2, 128.8, 129.4, 138.5, 140.1, 163.9 ppm; HRMS (ESI) *m/z* calcd for $[C_{16}H_{17}N_2]^+$ (M + H^+): 237.1386; found: 237.1387.

2-Phenyl-2-(phenylamino)ethan-1-ol 24

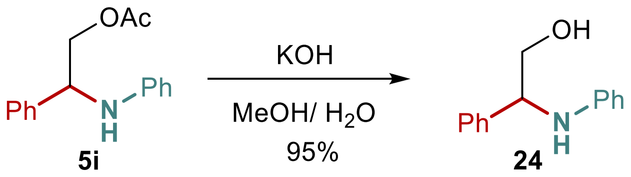

To a solution of deuterated amine **5i** (128 mg, 0.5 mmol) in MeOH (2 mL) was added 1 M KOH solution. The mixture was stirred at room temperature for overnight. The reaction was quenched by the addition of water, and the mixture was extracted with ethyl acetate. Combined organic layers were dried over Na_2SO_4 and concentrated. The residue was purified by column chromatography on silica gel (eluent: ethyl acetate/ hexane =1/ 8) to give the known compound **24**[21] (101 mg, 95%) as a colorless oil; Oil; NMR (600 MHz, $CDCl_3$) δ 3.74 (dd, J = 7.0, 11.2 Hz, 1H), 3.93 (dd, J = 4.2, 11.2 Hz, 1H), 4.50 (dd, J = 4.2, 7.0 Hz, 1H), 6.56 (d, J = 8.3 Hz, 2H), 6.66-6.69 (m, 1H), 7.08-7.11 (m, 2H), 7.24-7.27 (m, 1H), 7.32-7.37 (m, 4H) ppm; ^{13}C NMR (150 MHz, $CDCl_3$) δ 59.8, 67.3, 113.8, 117.9, 126.7, 127.6, 128.8, 129.1, 140.1, 147.2 ppm.

3,4-diphenyloxazolidin-2-one 25

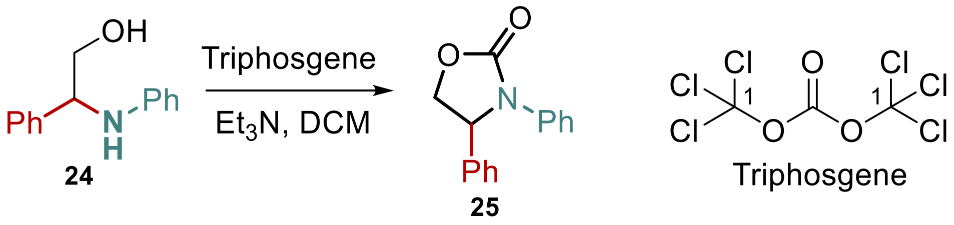

A solution of triphosgene (73.5 mg, 0.25 mmol) in CH_2Cl_2 (3 mL) was added dropwise using syringe pump for 1 h to a solution of aminoethanol **24** (107 mg, 0.5 mmol) and Et_3N (101 mg, 1 mmol) in CH_2Cl_2 (2 mL) at 0 ℃, and the reaction mixture was warmed to room temperature. Stirred for 8 h, the reaction was quenched by H_2O, then extracted with Et_2O (10 mLx3), and washed by brine. Combined organic layers were dried over Na_2SO_4 and concentrated. The residue was purified by column chromatography on silica gel (eluent: ethyl acetate/ hexane =1/15) to give the known compound **25**[22] (93 mg, 78%, 98D%) as a white solid; ^{1}H NMR (600 MHz, $CDCl_3$) δ 4.19 (dd, J = 6.0, 8.6 Hz, 1H), 4.77 (d, J = 8.7 Hz, 1H), 5.39 (dd, J = 6.0, 8.6 Hz, 1H), 7.05-7.07 (m, 1H), 7.24-7.26 (m, 2H), 7.29-7.31 (m, 3H), 7.34-7.36 (m, 2H), 7.38-7.40 (m, 2H) ppm; ^{13}C NMR (150 MHz, $CDCl_3$) δ 60.6, 69.8, 120.8, 124.6, 126.2, 128.7, 128.8, 128.9, 129.3, 129.4, 137.0, 138.2, 155.9 ppm

1,2-Diphenylindoline-3,3-d2 26

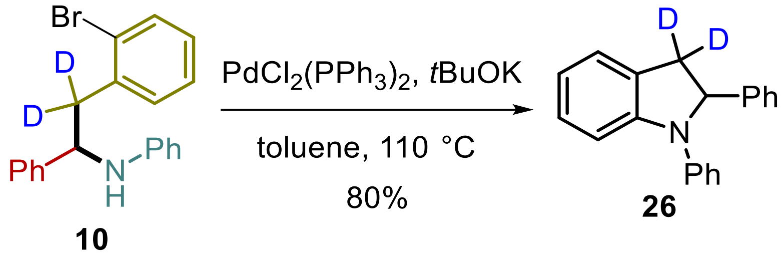

To a dried 25 mL Schlenk flask was charged with compound **4** (0.5 mmol, 1.0 equiv) and toluene (5 mL). The solution was degassed by Ar bubbling for 5 min. t-BuOK (112 mg, 1.0 mmol, 2.0 equiv) and $PdCl_2(PPh_3)_2$ (18 mg, 0.025 mmol, 5 mol %) were successively added in glove box. The flask was sealed (glass stopper) and taken out the glovebox. The reaction was heated at 110 ℃ overnight. Then, the reaction mixture was concentrated and the residue was purified by column chromatography on silica gel (eluent: ethyl acetate/ hexane =1/40) to give the deuterated indoline **26** (109 mg, 80%) as a white solid; ^{1}H NMR (600 MHz, $CDCl_3$) δ 5.23 (s, 1H), 6.76-6.79 (m, 1H), 6.89-6.92 (m, 1H), 7.10-7.15 (m, 5H), 7.19-7.22 (m, 3H), 7.25-7.28 (m, 2H),

7.31-7.32 (m, 2H) ppm; ^{13}C NMR (150 MHz, $CDCl_3$) δ 67.8, 108.8, 119.2, 119.4, 121.7, 125.1, 126.2, 127.2, 127.3, 128.7, 129.0, 143.5, 143.6, 147.9 ppm; HRMS (ESI) *m/z* calcd for $[C_{20}H_{16}D_2N]^+$ (M + H$^+$): 274.1559; found: 274.1552.

3-((benzyloxy)methyl)-2-phenylisoindolin-1-one 27a

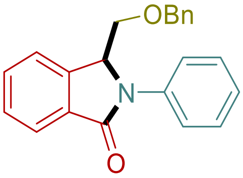

Following the **general procedure B**, 38 mg of **27a** as a white solid was obtained (yield: 58%);^{1}H NMR (600 MHz, $CDCl_3$) δ 3.53 (dd, *J* = 6.8, 9.6 Hz, 1H), 3.87 (dd, *J* = 3.8, 9.6 Hz, 1H), 4.37 (d, *J* = 12.0 Hz, 1H), 4.41 (d, *J* = 12.0 Hz, 1H), 5.27 (dd, *J* = 3.8, 6.8 Hz, 1H), 7.12-7.13 (m, 2H), 7.23-7.29 (m, 4H), 7.42-7.44 (m, 2H), 7.51-7.61 (m, 4H), 7.65 (d, *J* = 7.5 Hz, 1H), 7.93 (d, *J* = 7.5 Hz, 1H) ppm; ^{13}C NMR (150 MHz, $CDCl_3$) δ 60.6, 69.8, 73.4, 123.1, 123.5, 124.0, 125.6, 127.5, 127.7, 128.3, 128.6, 129.1, 132.0, 132.2, 137.2, 137.5, 143.6, 167.4 ppm; HRMS (ESI) *m/z* calcd for $[C_{22}H_{20}NO_2]^+$ (M + H$^+$): 330.1489; found: 330.1491.

3-((benzyloxy)methyl)-2-phenylisoindolin-1-one 27b

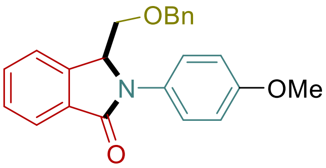

Following the **general procedure B**, 36 mg of **27b** as a white solid was obtained (yield: 50%); ^{1}H NMR (600 MHz, $CDCl_3$) δ 3.53 (dd, *J* = 6.6, 9.6 Hz, 1H), 3.83-3.85 (m, 4H), 4.36 (d, *J* = 12.0 Hz, 1H), 4.41 (d, *J* = 12.0 Hz, 1H), 5.16 (d, *J* = 3.8, 6.6 Hz, 1H), 6.94-6.97 (m, 2H), 7.12-7.13 (m, 2H), 7.24-7.29 (m, 3H), 7.41-7.43 (m, 2H), 7.50-7.53 (m, 1H), 7.57-7.59 (m, 1H), 7.63 (d, *J* = 7.3 Hz, 1H), 7.92 (d, *J* = 7.5 Hz, 1H) ppm; ^{13}C NMR (150 MHz, $CDCl_3$) δ 55.4, 61.2, 69.8, 73.4, 114.4, 123.0, 123.9, 125.7, 127.4, 127.7, 128.3, 128.6, 129.9, 131.7, 132.3, 137.5, 143.5, 157.6, 167.5 ppm; HRMS (ESI) *m/z* calcd for $[C_{23}H_{22}NO_3]^+$ (M + H$^+$): 360.1594; found: 360.1599.

3-(2-(4-chlorophenoxy)propan-2-yl)-2-(p-tolyl)isoindolin-1-one 27c

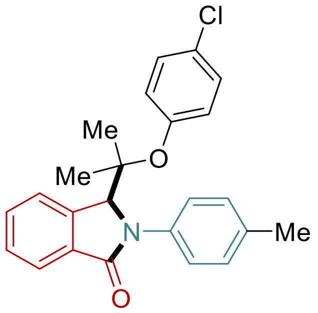

Following the **general procedure B**, 41.6 mg of **27c** as a white solid was obtained (yield: 52%); ^{1}H NMR (600 MHz, $CDCl_3$) δ 0.95 (s, 3H), 0.96 (s, 3H), 2.36 (s, 3H), 5.46 (s, 1H), 6.75-6.77 (m, 2H), 7.19-7.20 (m, 2H), 7.23-7.24 (m, 2H), 7.33-7.34 (m, 2H), 7.53-7.56 (m, 1H), 7.59-7.61(m, 1H), 7.87 (d, J = 7.6 Hz, 1H), 7.94 (d, J = 7.4 Hz, 1H) ppm; ^{13}C NMR (150 MHz, $CDCl_3$) δ 20.8, 21.0, 25.3, 68.8, 83.7, 123.9, 125.4, 125.5, 125.7, 128.7, 129.1, 129.2, 129.6, 131.6, 132.5, 136.0, 136.1, 143.1, 152.3, 168.5 ppm; HRMS (ESI) *m/z* calcd for $[C_{24}H_{23}ClNO_2]^+$ (M + H^+): 392.1412; found: 392.1421.

N-((3-oxo-2-(p-tolyl)isoindolin-1-yl)methyl)acetamide 27d

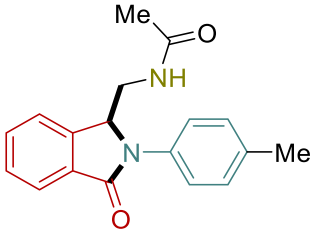

Following the **general procedure B**, 38.8 mg of **27d** as a white solid was obtained (yield: 66%); ^{1}H NMR (600 MHz, $CDCl_3$) δ 1.72 (s, 3H), 2.36 (s, 3H), 3.45-3.49 (m, 1H), 3.93 (ddd, J = 4.5, 6.8, 11.2 Hz, 1H), 5.32-5.33 (m, 1H), 5.71 (br. s, 1H), 7.22 (d, J = 8.1 Hz, 2H), 7.43 (d, J = 8.3 Hz, 2H), 7.48-7.50 (m, 1H), 7.57-7.60 (m, 2H), 7.80 (d, J = 7.6 Hz, 1H) ppm; ^{13}C NMR (150 MHz, $CDCl_3$) δ 20.9, 22.8, 40.4, 60.3, 122.8, 123.8, 124.0, 128.9, 129.9, 132.1, 132.3, 134.1, 136.0, 142.4, 167.5, 170.4 ppm; HRMS (ESI) *m/z* calcd for $[C_{18}H_{19}N_2O_2]^+$ (M + H^+): 295.1441; found: 295.1449.

3-((Benzyloxy)methyl)-5-bromo-2-(p-tolyl)isoindolin-1-one 27e

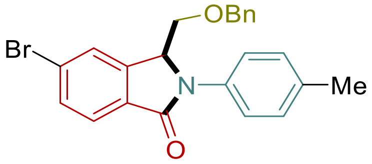

Following the **general procedure B**, 43 mg of **27e** as a white solid was obtained (yield: 51%); ^{1}H NMR (600 MHz, $CDCl_3$) δ 2.37 (s, 3H), 3.44 (dd, J = 7.6, 9.3 Hz, 1H), 3.87 (dd, J = 3.9, 9.3 Hz, 1H), 3.92 (s, 3H), 3.96 (s, 3H), 4.41 (d, J = 12.0 Hz, 1H), 4.43 (d, J = 12.0 Hz, 1H), 5.13 (dd, J = 3.9, 7.6 Hz, 1H), 7.11 (s, 1H), 7.17-7.19 (m, 2H), 7.22-7.24 (m, 2H), 7.27-7.29 (m, 3H), 7.37 (s, 1H), 7.41-7.42 (m, 2H) ppm; ^{13}C NMR (150 MHz, $CDCl_3$) δ 20.9, 56.1, 56.2, 60.0, 70.2, 73.4, 105.3, 105.4, 123.4, 124.4, 127.5, 127.8, 128.3, 129.7, 134.7, 135.2, 137.6, 150.0, 152.7, 167.6 ppm; HRMS (ESI) *m/z* calcd for $[C_{23}H_{20}BrNO_2Na]^+$ (M + H^+): 444.0570; found: 444.0580.

3-(3-methylbenzyl)-2-(p-tolyl)isoindolin-1-one 27f

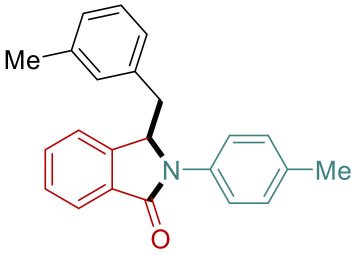

Following the **general procedure B**, 40 mg of **27f** as a colorless oil was obtained (yield: 61%); ^{1}H NMR (600 MHz, $CDCl_3$) δ 2.22 (s, 3H), 2.40 (s, 3H), 2.76 (dd, J = 8.4, 13.8 Hz, 1H), 3.33 (dd, J = 3.5, 13.8 Hz, 1H), 5.38 (dd, J = 3.5, 8.4 Hz, 1H), 6.67-6.68 (m, 2H), 6.98-6.99 (m, 1H), 7.02-7.07 (m, 2H), 7.28-7.30 (m, 2H), 7.43-7.47 (m, 2H), 7.50-7.51 (m, 2H), 7.83-7.84 (m, 1H) ppm; ^{13}C NMR (150 MHz, $CDCl_3$) δ 21.0, 21.2, 38.1, 61.5, 123.0, 1123.5, 123.9, 126.6, 127.6, 128.0, 128.3, 129.7, 130.4, 131.3, 132.3, 134.5, 135.2, 135.3, 137.7, 144.1, 166.9 ppm; HRMS (ESI) *m/z* calcd for $[C_{23}H_{22}NO]^+$ (M + H^+): 328.1696; found: 328.1705.

1-((Benzyloxy)methyl)-2-(p-tolyl)-1,4-dihydroisoquinolin-3(2H)-one 28a

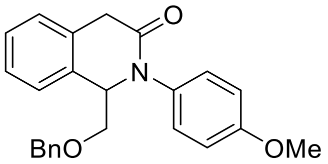

Following the **general procedure B**, 46 mg of **28a** as a colorless oil was obtained (yield: 62%); ^{1}H NMR (600 MHz, $CDCl_3$) δ 3.63 (d, J = 19.2 Hz, 1H), 3.65 (dd, J = 3.5, 9.7 Hz, 1H), 3.76 (dd, J = 4.3, 9.7 Hz, 1H), 3.80 (s, 3H), 4.03 (d, J = 19.2 Hz, 1H), 4.37 (d, J = 12.1 Hz, 1H), 4.40 (d, J = 12.1 Hz, 1H), 4.76 (br. t, J = 3.7 Hz, 1H), 6.89-6.91 (m, 2H), 7.10-7.13 (m, 4H), 7.19-7.20 (m, 2H), 7.25-7.29 (m, 4H),

7.30-7.33 (m, 1H) ppm; ^{13}C NMR (150 MHz, $CDCl_3$) δ 37.9, 55.4, 65.8, 72.0, 73.2, 114.6, 125.7, 126.4, 127.3, 127.4, 127.6, 127.7, 128.3, 128.9, 133.3, 133.5, 134.1, 137.6, 158.5, 170.1 ppm; HRMS (ESI) *m/z* calcd for $[C_{24}H_{24}NO_3]^+$ (M + H^+): 374.1751; found: 374.1746.

1-((benzyloxy)methyl)-6,7-dimethoxy-2-(4-methoxyphenyl)-1,4-dihydroisoquinolin-3(2H)-one 28b

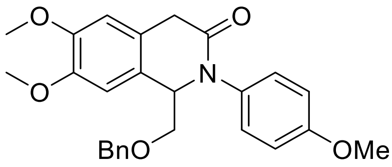

Following the **general procedure B**, 52 mg of **28b** as a colorless oil was obtained (yield: 60%); ^{1}H NMR (600 MHz, $CDCl_3$) δ 3.56 (d, *J* = 19.2 Hz, 1H), 3.65 (dd, *J* = 3.4, 9.7 Hz, 1H), 3.72 (dd, *J* = 4.6, 9.7 Hz, 1H), 3.81 (s, 3H), 3.85 (s, 3H), 3.91 (s, 3H), 3.96 (d, *J* = 19.2 Hz, 1H), 4.38 (d, *J* = 12.1 Hz, 1H), 4.41 (d, *J* = 12.1 Hz, 1H), 4.70 (br. t, *J* = 3.7 Hz, 1H), 6.67 (s, 2H), 6.90-6.91 (m, 2H), 7.13-7.14 (m, 4H), 7.26-7.30 (m, 3H) ppm; ^{13}C NMR (150 MHz, $CDCl_3$) δ 37.4, 55.4, 56.0, 56.1, 65.5, 71.8, 73.2, 109.0, 110.0, 114.6, 125.3, 125.4, 127.5, 127.7, 128.3, 129.0, 134.2, 137.7, 147.6, 148.7, 158.5, 170.0 ppm; HRMS (ESI) *m/z* calcd for $[C_{26}H_{28}NO_5]^+$ (M + H^+): 434.1962; found: 434.1963.

tert-butyl ((2-(4-methoxyphenyl)-3-oxo-1,2,3,4-tetrahydroisoquinolin-1-yl)methyl)carbamate 28c

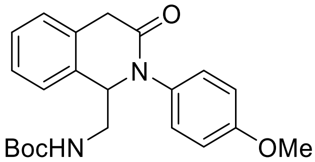

Following the **general procedure B**, 39.7 mg of **28c** as a colorless oil was obtained (yield: 52%); ^{1}H NMR (600 MHz, $CDCl_3$) δ 1.36 (s, 9H), 3.45-3.56 (m, 2H), 3.71 (d, *J* = 19.3 Hz, 1H), 3.81 (s, 3H), 3.91 (d, *J* = 19.3 Hz, 1H), 4.55 (br. s, 1H), 4.85 (br. s, 1H), 6.94-6.95 (m, 2H), 7.20-7.25 (m, 4H), 7.28-7.33 (m, 2H) ppm; ^{13}C NMR (150 MHz, $CDCl_3$) δ 28.2, 37.6, 44.5, 55.4, 65.3, 79.7, 114.7, 126.0, 126.8, 127.7, 128.1,

128.7, 132.5, 132.9, 134.2, 155.4, 158.5, 169.0 ppm; HRMS (ESI) *m/z* calcd for $[C_{22}H_{27}N_2O_4]^+$ (M + H^+): 383.1965; found: 383.1972.

1-(3,4-Dimethoxybenzyl)-6,7-dimethoxy-2-(4-methoxyphenyl)-1,4-dihydroisoquinolin-3(2H)-one 28d

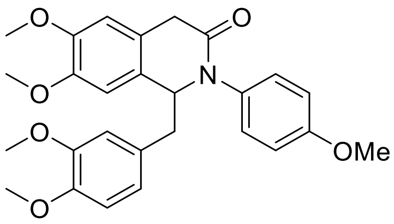

Following the **general procedure B**, 53.7 mg of **28d** as a colorless oil was obtained (yield: 58%); ^{1}H NMR (600 MHz, $CDCl_3$) δ 2.76 (d, *J* = 19.3 Hz, 1H), 2.95-3.01 (m, 2H), 3.29 (d, *J* = 19.3 Hz, 1H), 3.56 (s, 3H), 3.77 (s, 3H), 3.84 (s, 3H), 3.85 (s, 3H), 3.86 (s, 3H), 4.95 (t, *J* = 5.0 Hz, 1H), 6.03 (d, *J* = 1.4 Hz, 1H), 6.42 (s, 1H), 6.52-6.53 (m, 2H), 6.72 (d, *J* = 8.1 Hz, 1H), 7.00 (d, *J* = 8.8 Hz, 2H), 7.32 (d, *J* = 8.8 Hz, 2H) ppm; ^{13}C NMR (150 MHz, $CDCl_3$) δ 36.6, 41.0, 55.4, 55.5, 55.8, 55.9, 56.0, 66.9, 109.1, 109.8, 110.8, 113.1, 114.6, 122.3, 125.3, 125.9, 128.0, 128.8, 134.2, 147.4, 148.0, 148.2, 148.5, 158.4, 169.3 ppm; HRMS (ESI) *m/z* calcd for $[C_{27}H_{30}NO_6]^+$ (M + H^+): 464.2068; found: 464.2069.

***N*-(cyclohexyl(phenyl)methyl)cyclohexanamine 30**

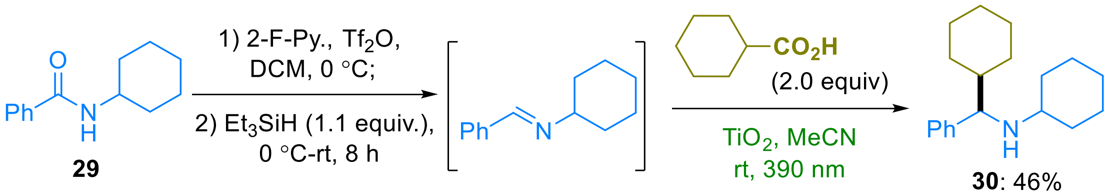

To an oven-dried 10 mL quartz reaction tube was added amide **29** (0.5 mmol, 1.0 equiv). Under a nitrogen atmosphere, anhydrous DCM (2.0 mL) and 2-fluoropyridine (60 mg, 52 μl, 0.6 mmol, 1.2 equiv.) were added. The mixture was cooled to 0 ℃, followed by dropwise addition of Tf_2O (169 mg, 101 μl, 0.6 mmol, 1.2 equiv.) and the reaction was stirred at 0 ℃ for 20 min. Et_3SiH (63.8 mg, 88 μl, 0.55 mmol, 1.1 equiv.) was added dropwise, and the resulting mixture was stirred at 0 °C for an additional 10 minutes, then allowed to warm to room temperature and stirred for 8 h.

After completion of the reaction, the solution was diluted with dry acetonitrile (4 mL), followed by the addition of TiO_2 (100 mg), cyclohexyl formic acid **3ah** (128 mg, 1.0

mmol, 2.0 equiv). The reaction mixture was then irradiated with a Kessil lamp (PR-160, 390 nm) for 48 h. Upon completion, the mixture was filtered through diatomite and a small amount of silica gel. The filtrate was concentrated under reduced pressure. The sat. $NaHCO_3$ (10 mL) was added to residue, and the mixture was extracted by CH_2Cl_2 (10 mL×3). Combined organic layers were dried over Na_2SO_4 and concentrated. The residue was purified by column chromatography on silica gel (dichloromethane / methol = 100:1) to furnish the corresponding known product **30**[23] (62 mg, 46%) as a yellow oil; ^{1}H NMR (600 MHz, $CDCl_3$) δ 0.81-0.90 (m, 1H), 0.94-1.03 (m, 2H), 1.16-1.18 (m, 6H), 1.19-1.25 (m, 1H), 1.35-1.40 (m, 1H), 1.45-1.55 (m, 3H), 1.60-1.68 (m, 5H), 1.72-1.77 (m, 1H), 1.94-1.96 (s, 1H), 1.96-2.00 (m, 1H), 2.14-2.23 (m, 1H), 3.74 (d, *J* =7.2 Hz, 1H), 7.22-7.26 (m, 3H), 7.29-7.34 (m, 2H), ppm; ^{13}C NMR (151 MHz, $CDCl_3$) δ 24.8, 25.2, 26.3, 26.4, 26.0, 26.6, 30.0, 30.5, 32.8, 34.9, 44.5, 53.5, 65.2, 126.5, 127.9, 128.0, 144.0 ppm.

Recirculating-flow packed bed reactor

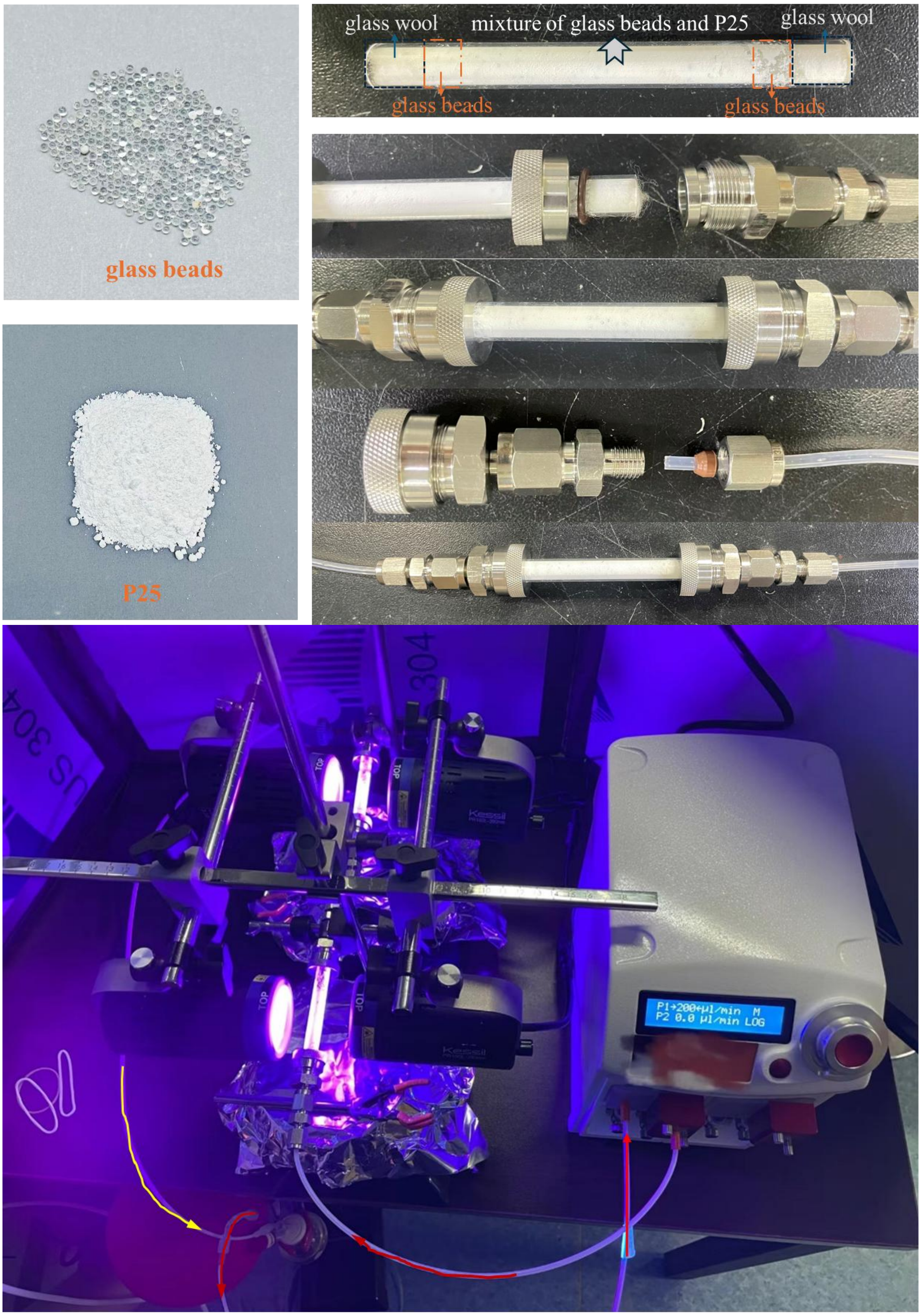

1,2,10,14b-Tetrahydrodibenzo[c,f]pyrazino[1,2-a]azepin-3(4H)-one 32

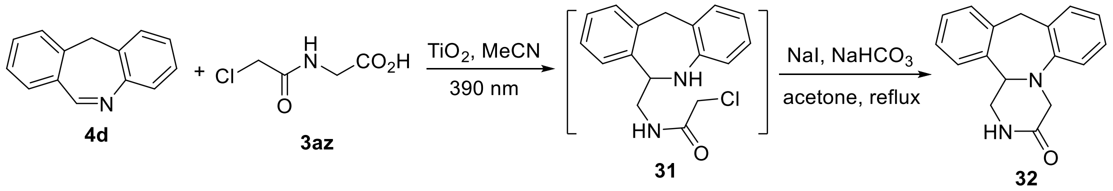

400 mg of P25 and 2000 mg of glass beads (d = ~150 μm) were mixed evenly in a beaker and then the mixture was filled in 2 quartz tubes (internal diameter = 8 mm, wall thickness = 1 mm, length = 100 mm). Both ends of the tube was filled with glass wool and glass beads, which was then connected by PTFE tubing (O.D = 1/8”) to obtain a recirculating-flow set-up. A pump was employed to inject and recirculate the reactant mixture (6 mmol of imine **4d**, 12 mmol of glycine derivative **3az** and 40 mL of CH_3CN) at a flow rate of 200 $\mu L\cdot min^{-1}$. The flow system was irradiated with 4 Kessil lamps (PR-160, 390 nm). The reaction system circulating flow for 48 hours. After completion of the reaction, fresh MeCN (20 mL) was used to rinse the system. The reaction mixtures were combined and concentrated under reduced pressure. The sat. $NaHCO_3$ (10 mL) was added to residue and the mixture was extracted by CH_2Cl_2 (10 mL × 3). Combined organic layers were dried over Na_2SO_4 and concentrated. The residue **31** was used for the next step without further purification.

To a solution of above residue **31** in acetone (50 mL), $NaHCO_3$ (1008 mg, 12 mmol) and NaI (450 mg, 3 mmol) were added and stirred at 70 ℃ for three days. After the reaction was complete, acetone was evaporated in vacuo. The obtained residue was diluted with water, then extracted with CH_2Cl_2 (40 mL × 3). Combined organic layers were dried over Na_2SO_4 and concentrated. The resultant crude was purified by column chromatography to give the compound **32**[24] (839.5 mg, 51%) as a white solid; ^{1}H NMR (600 MHz, $CDCl_3$) δ 3.32 (br. dt, *J* = 3.4, 11.8 Hz, 1H), 3.60 (d, *J* = 13.1 Hz, 1H), 3.67 (t, *J* = 11.4 Hz, 1H), 3.88 (d, *J* = 16.6 Hz, 1H), 4.10 (d, *J* = 16.6 Hz, 1H), 4.40 (dd, *J* = 3.1, 10.9 Hz, 1H), 4.45 (d, *J* = 13.1 Hz, 1H), 6.90-6.95 (m, 2H), 7.00-7.01 (m, 1H), 7.04 (br. s, 1H), 7.10-7.20 (m, 5H) ppm; ^{13}C NMR (150 MHz, $CDCl_3$) δ 38.8, 49.2, 55.1, 63.4, 118.4, 122.7, 126.8, 127.3, 127.4, 128.1, 128.4, 129.3, 134.2, 138.1, 139.4, 145.5, 169.5 ppm

2-Methyl-1,2,3,4,10,14b-hexahydrodibenzo[c,f]pyrazino[1,2-a]azepine 33

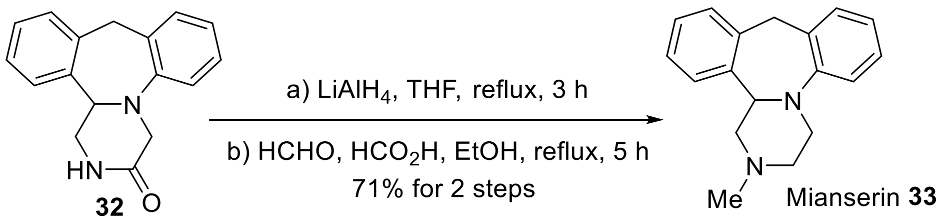

To a suspension of $LiAlH_4$ (57 mg, 1.5 mmol) in dry THF (5 mL), compound **32** (132 mg, 0.5 mmol) in dry THF (5 mL) was added, and then the suspension was stirred for 1 h at room temperature and heated at reflux for 3 h. After cooling to room temperature, the reaction mixture was diluted with EtOAc and quenched with cold water. The precipitate was filtered off and additionally extracted with EtOAc (3 x 10 mL). Combined organic layers were dried over Na_2SO_4 and concentrated. The residue was used for the next step without further purification.

The above residue was dissolved in 10 mL of EtOH and formic acid (391 mg, 8.5 mmol) and formaldehyde (36-38% in H_2O, 0.2 mL, 2.5 mmol) was added to the solution. The solution was refluxed for 5 h, then evaporated the solvent. The residual was added sat. $NaHCO_3$ (10 mL) and extracted by CH_2Cl_2 (10 mL×3). Combined organic layers were dried over Na_2SO_4 and concentrated. The residue was purified by column chromatography on silica gel to furnish the product **33**[24] (93.7 mg, 71% for 2 steps) as a colorless oil. ^{1}H NMR (600 MHz, $CDCl_3$) δ 2.34 (dd, J = 3.0, 11.1 Hz, 1H), 2.38 (s, 3H), 2.44 (t, J = 10.8 Hz, 1H), 2.89 (dt, J = 11.1, 1.9 Hz, 1H), 2.98 (dd, J = 10.8, 1.9 Hz, 1H), 3.26 (dt, J =11.3, 2.7 Hz, 1H), 3.31 (d, J = 12.7 Hz, 1H), 3.36 (td, J = 11.3, 2.7 Hz, 1H), 4.10 (dd, J = 2.1, 10.3 Hz, 1H), 4.82 (d, J = 12.7 Hz, 1H), 6.88 (dt, J = 1.0, 7.5 Hz, 1H), 7.00-7.14 (m, 6H), 7.18 (dt, J = 1.4, 7.7 Hz, 1H) ppm; ^{13}C NMR (150 MHz, $CDCl_3$) δ 38.7, 45.7, 51.2, 55.5, 64.8, 66.4, 119.0, 122.2, 126.4, 126.5, 126.9, 127.2, 128.1, 129.5, 137.3, 139.3, 148.5 ppm.

(6,11-Dihydro-5H-dibenzo[b,e]azepin-6-yl)methanamine 34

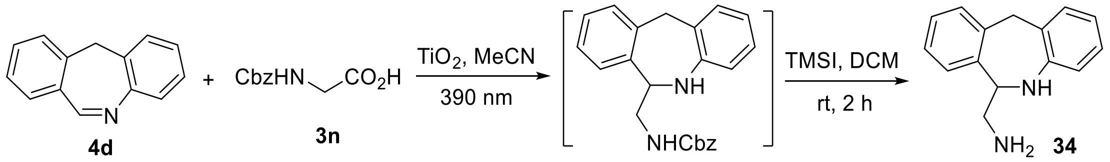

Imine **4d** (96.5 mg, 0.5 mmol), carboxylic acid **3n** (209 mg, 1.0 mmol, 2.0 equiv.), TiO_2 P25 (100 mg) were added to an oven dried 10 mL quartz reaction tube. The reaction tube was then evacuated and backfilled with N_2 three times, and 6 mL of degassed anhydrous MeCN was added. The reaction mixture was irradiated using

Kessil lamp (PR-160, 390 nm) and cooled with a fan for 48 h. After reaction, the mixture was filtered through diatomite and a small amount of silica gel. The filtrate was concentrated under reduced pressure. The sat. $NaHCO_3$ (10 mL) was added to residue and the mixture was extracted by CH_2Cl_2 (10 mL×3). Combined organic layers were dried over Na_2SO_4 and concentrated. The residue was pass through a short silica gel column (hexane/EA/Et_3N=1/1/0.05). The filtrate was concentrated to afford the crude amine, which was used in the next step without further purification.

The above residue was dissolved in 5 mL of dry CH_2Cl_2, TMSI (0.14 mL, 1.0 mmol, 2.0 eq.) was added dropwise and the solution was stirred for 5 h at room temperature. Then MeOH (0.5 mL) was added and the reaction mixture was stirred for another 1 h the solvents were concentrated and purified by column chromatography on silica gel to give the product **34**[25] (79.5 mg, 71%) as a colorless oil; ^1H NMR (600 MHz, $CDCl_3$) δ 3.14 (dd, J = 8.6, 12.3 Hz, 1H), 3.25 (dd, J = 4.2, 12.3 Hz, 1H), 3.95 (d, J = 14.7 Hz, 1H), 4.22 (d, J = 14.7 Hz, 1H), 4.75 (dd, J = 4.2, 8.6 Hz, 1H), 6.67-6.69 (m, 2H), 6.96-6.98 (m, 1H), 7.02-7.05 (m, 2H), 7.16-7.20 (m, 3H) ppm; ^{13}C NMR (150 MHz, $CDCl_3$) δ 39.8, 46.6, 58.3, 118.8, 119.5, 126.2, 126.8, 126.9, 127.5, 127.7, 128.6, 129.6, 137.1, 139.5, 145.4 ppm.

9,13b-dihydro-1H-dibenzo[c,f]imidazo[1,5-a]azepin-3-amine 35

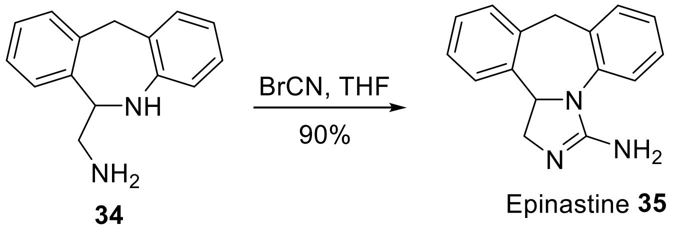

To a solution of amine **34**[24] (67 mg, 0.3 mmol) in dry THF (2 mL), BrCN (38 mg, 0.36 mmol) in dry THF (2 mL) was added. The reaction mixture was stirred for 3 h at room temperature. Et_3N (0.13 mL, 91 mg, 0.9 mmol) was added and the mixture was extracted by EtOAc (10 mL×3). Combined organic layers were dried over Na_2SO_4 and concentrated. The residue was purified by column chromatography on silica gel to furnish the product **35**[25] (67 mg, 90%) as a white solid. ^1H NMR (600 MHz, CD_3OD) δ 3.66 (t, J = 9.8 Hz, 1H), 3.71 (d, J = 14.5 Hz, 1H), 4.40 (t, J = 9.8 Hz, 1H), 4.58 (d, J = 14.5 Hz, 1H), 5.47 (t, J = 9.8 Hz, 1H), 7.14-7.16 (m, 1H), 7.24-7.29 (m, 2H), 7.35-7.37 (m, 1H), 7.41-7.47 (m, 2H), 7.50-7.53 (m, 2H) ppm; ^{13}C NMR (150

MHz, CD_3OD) δ 38.7, 51.4, 64.6, 127.5, 128.6, 129.0, 129.1, 129.6, 129.9, 130.8, 131.4, 134.4, 136.3, 136.4, 142.1, 159.6 ppm.

4-Methoxy-*N*-(1-phenyl-2-(p-tolyl)ethyl)aniline 36

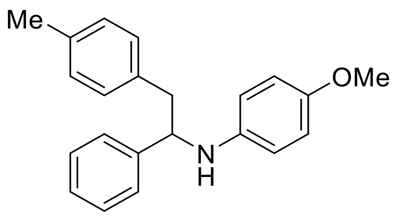

Following the **general procedure B**, 47.5 mg of **36**[26] as a colorless oil was obtained (yield: 75%); ^{1}H NMR (600 MHz, $CDCl_3$) δ 2.33 (s, 3H), 2.95 (dd, *J* = 8.5, 14.1 Hz, 1H), 3.10 (dd, *J* = 5.4, 14.1 Hz, 1H), 3.68 (s, 3H), 4.49 (dd, *J* = 8.5, 14.1 Hz, 1H), 6.41-6.44 (m, 2H), 6.64-6.67 (m, 2H), 7.04 (d, *J* = 8.0 Hz, 2H), 7.10 (d, *J* = 8.0 Hz, 2H), 7.23-7.26 (m, 1H), 7.31-7.36 (m, 4H) ppm; ^{13}C NMR (150 MHz, $CDCl_3$) δ 21.0, 44.8, 55.6, 60.0, 114.6, 114.9, 126.5, 126.9, 128.5, 129.0, 129.2, 134.6, 136.2, 141.5, 143.8, 152.0 ppm.

4-Methoxy-N-(1-phenyl-2-(p-tolyl)ethyl-2,2-d2)aniline 36-d2

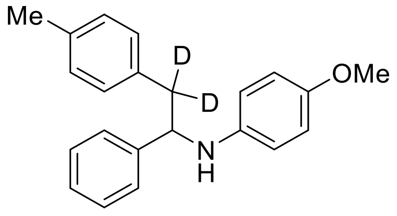

Following the **general procedure B**, except the deuterated carboxylic acid **3aab-d2** used, 46.5 mg of **36-d2** as a colorless oil was obtained (yield: 73%); ^{1}H NMR (600 MHz, $CDCl_3$) δ 2.38 (s, 3H), 3.73 (s, 3H), 4.53 (s, 1H), 6.49-6.51 (m, 2H), 6.70-6.72 (m, 2H), 7.08 (d, *J* = 7.9 Hz, 2H), 7.14 (d, *J* = 7.9 Hz, 2H), 7.28-7.31 (m, 1H), 7.36-7.41 (m, 4H) ppm; ^{13}C NMR (150 MHz, $CDCl_3$) δ 21.0, 55.6, 60.3, 114.6, 115.3, 126.6, 127.0, 128.5, 129.0, 129.2, 134.5, 136.2, 141.0, 143.4, 152.3 ppm; HRMS (ESI) *m/z* calcd for $[C_{22}H_{22}D_2NO]^+$ (M + H^+): 320.1978; found: 320.1982.

1-Phenyl-2-(p-tolyl)ethan-1-amine 37

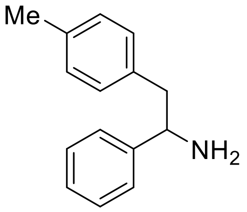

To a stirred solution of **36** (158.5 mg, 0.5 mmol) in CH_3CN/H_2O (5 mL, 1/1), TCCA (58 mg, 0.25 mmol, 0.5 equiv.) and 1M aqueous H_2SO_4 (0.5 mL, 1.0 equiv.) were added. The mixture was stirred at room temperature for 12 h and then quenched by the sat. Na_2SO_3 solution. The resulting mixture was basified to pH 9 by adding 5M aqueous KOH and extracted with EtOAc (10 mL x 3). Combined organic layers were dried over Na_2SO_4 and concentrated. The residue was purified by column chromatography on silica gel to furnish the product **37**[26] (71.7 mg, 68%) as a brown oil; ^{1}H NMR (600 MHz, $CDCl_3$) δ 1.84 (br. s, 2H), 2.38 (s, 3H), 2.84 (dd, J = 9.0, 13.5 Hz, 1H), 3.04 (dd, J = 4.8, 13.5 Hz, 1H), 4.22 (dd, J = 4.8, 9.0 Hz, 1H), 7.12-7.17 (m, 4H), 7.30-7.32 (m, 1H), 7.38-7.43 (m, 4H) ppm; ^{13}C NMR (150 MHz, $CDCl_3$) δ 21.0, 45.9, 57.5, 126.4, 127.0, 128.4, 129.1, 129.2, 135.8, 145.6 ppm.

1-Phenyl-2-(*p*-tolyl)ethan-2,2-d2-1-amine 35-d2

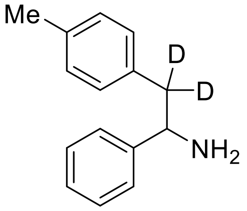

To a stirred solution of **34-d2** (159.5 mg, 0.5 mmol) in CH_3CN/H_2O (5 mL, 1/1), TCCA (58 mg, 0.25 mmol, 0.5 equiv.) and 1 M aqueous H_2SO_4 (0.5 mL, 1.0 equiv.) were added. The mixture was stirred at room temperature for 12 h and then quenched by the sat. Na_2SO_3 solution. The resulting mixture was basified to pH 9 by adding 5 M aqueous KOH and extracted with EtOAc (10 mL x 3). Combined organic layers were dried over Na_2SO_4 and concentrated. The residue was purified by column chromatography on silica gel to furnish the product **35** (67 mg, 63%) as a brown oil; ^{1}H NMR (600 MHz, $CDCl_3$) δ 2.21 (br. s, 2H), 2.37 (s, 3H), 4.20 (s, 1H), 3.04 (dd, J = 4.8, 13.5 Hz, 1H), 4.22 (dd, J = 4.8, 9.0 Hz, 1H), 7.10-7.15 (m, 4H), 7.29-7.31 (m, 1H), 7.36-7.41 (m, 4H) ppm; ^{13}C NMR (150 MHz, $CDCl_3$) δ 21.0, 57.4, 126.4, 127.1, 128.4, 129.1, 129.2, 135.9, 145.0 ppm; HRMS (ESI) *m/z* calcd for $[C_{15}H_{16}D_2N]^+$ (M + H^+): 214.1559; found: 214.1566.

(9Z,12Z)-*N*-(1-phenyl-2-(p-tolyl)ethyl)octadeca-9,12-dienamide 38

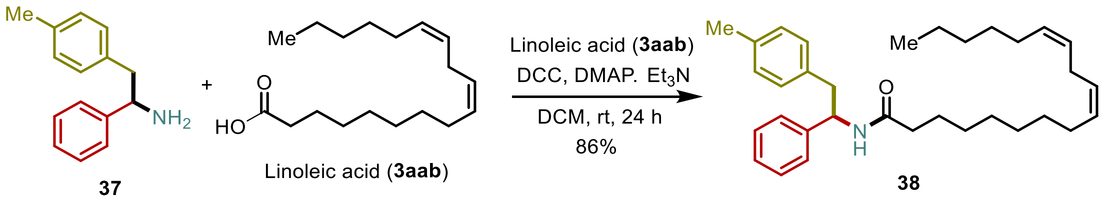

Amine **37** (84 mg, 0.4 mmol), linoleic acid (125 μL, 112 mg, 0.4 mmol, 1.0 equiv.), DMAP (5 mg, 0.04 mol, 0.1 equiv.), DCC (99 mg, 0.48 mmol, 1.2 equiv.) and Et_3N (58 μL, 0.4 mmol, 1.0 equiv.) in dry CH_2Cl_2 (4 mL) were stirred under N_2 atmosphere overnight at room temperature. The reaction mixture was filtered and the solvent was concentrated under reduced pressure. The residue was purified by column chromatography on silica gel to furnish the product **38**[26] (162.7 mg, 86%) as pale yellow solid; ^{1}H NMR (600 MHz, $CDCl_3$) δ 0.95 (t, J = 7.0 Hz, 3H), 1.25-1.44 (m, 14H), 1.56-1.60 (m, 2H), 2.08-2.17 (m, 6H), 2.34 (s, 3H), 2.83 (t, J = 6.7 Hz, 1H), 3.07 (dd, J = 7.8, 14.0 Hz, 1H), 3.12 (dd, J = 6.8, 14.0 Hz, 1H), 5.30-5.47 (m, 5H), 5.99 (d, J = 7.9 Hz, 1H), 7.00 (d, J = 7.8 Hz, 2H), 7.08 (d, J = 7.8 Hz, 2H), 7.27-7.30 (m, 3H), 7.33-7.36 (m, 2H) ppm; ^{13}C NMR (150 MHz, $CDCl_3$) δ 14.0, 21.0, 22.5, 25.5, 25.6, 27.1, 29.0, 29.2, 29.3, 29.5, 31.4, 36.7, 42.0, 54.0, 126.5, 127.1, 127.8, 127.9, 128.4, 128.9, 129.1, 130.0, 130.1, 134.1, 135.9, 141.8, 172.3 ppm.

(9Z,12Z)-*N*-(-1-phenyl-2-(p-tolyl)ethyl-2,2-d2)octadeca-9,12-dienamide 38-d2

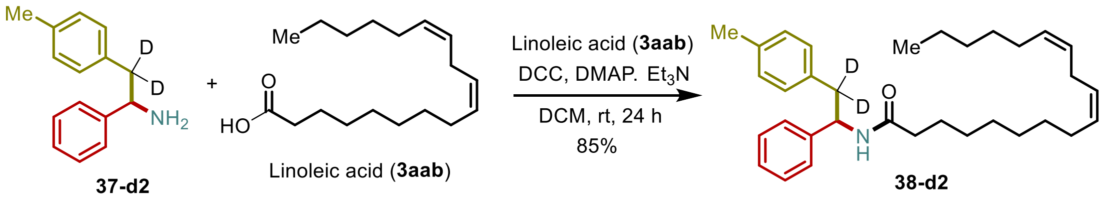

Amine **37-d2** (85 mg, 0.4 mmol), linoleic acid (125 μL, 112 mg, 0.4 mmol, 1.0 equiv.), DMAP (5 mg, 0.04 mol, 0.1 equiv.), DCC (99 mg, 0.48 mmol, 1.2 equiv.) and Et_3N (58 μL, 0.4 mmol, 1.0 equiv.) in dry CH_2Cl_2 (4 mL) were stirred under N_2 atmosphere overnight at room temperature. The reaction mixture was filtered and the solvent was concentrated under reduced pressure. The residue was purified by column chromatography on silica gel to furnish the product **38-d2** (161.5 mg, 85%) as pale yellow solid; ^{1}H NMR (600 MHz, $CDCl_3$) δ 0.92 (t, J = 6.8 Hz, 3H), 1.24-1.41 (m, 14H), 1.53-1.59 (m, 2H), 2.05-2.15 (m, 6H), 2.31 (s, 3H), 2.80 (t, J = 6.6 Hz, 1H),

5.27-5.44 (m, 5H), 5.78 (d, J = 7.9 Hz, 1H), 6.96 (d, J = 7.9 Hz, 2H), 7.05 (d, J = 7.9 Hz, 2H), 7.23-7.28 (m, 3H), 7.31-7.34 (m, 2H) ppm; ^{13}C NMR (150 MHz, $CDCl_3$) δ 14.0, 21.0, 22.5, 25.5, 25.6, 27.2, 29.0, 29.1, 29.2, 29.3, 29.6, 31.5, 36.8, 53.9, 126.5, 127.2, 127.9, 128.0, 128.5, 129.0, 129.1, 130.0, 130.2, 134.0, 136.0, 141.7, 172.3 ppm; HRMS (ESI) *m/z* calcd for $[C_{33}H_{46}D_2NO]^+$ (M + H^+): 476.3856; found: 476.3862.

***N*-(2-(Benzyloxy)-1-(4-methoxyphenyl)propyl)-4-methoxyaniline 39**

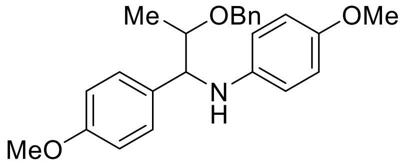

Following the **general procedure A**, 57 mg of **38**, as an inseparable diastereomeric mixture (*dr* = 1:1), a colorless oil was obtained (yield: 76%);^{1}H NMR (600 MHz, $CDCl_3$) δ 1.11 (d, J = 6.4 Hz, 3H), 1.25 (d, J = 6.2 Hz, 3H), 3.71-3.78 (m, 7H), 3.82-3.83 (m, 6H), 3.87-3.93 (m, 1H), 4.13-4.19 (m, 1H), 4.39-4.45 (m, 2H), 4.56-4.66 (m, 3H), 6.47-6.50 (m, 4H), 6.68-6.72 (m, 4H), 6.87-6.90 (m, 4H), 7.28-7.40 (m, 14H) ppm; ^{13}C NMR (150 MHz, $CDCl_3$) δ 15.1, 16.9, 55.2, 55.7, 60.9, 63.4, 70.7, 70.9, 77.9, 79.0, 113.7, 113.8, 114.6, 115.0, 115.1, 127.6, 127.7, 128.4, 128.5, 128.6, 132.1, 138.3, 138.4, 141.9, 152.0, 158.6, 158.7 ppm; HRMS (ESI) *m/z* calcd for $[C_{24}H_{28}NO_3]^+$ (M + H^+): 378.2064; found: 378.2066.

1-(4-Methoxyphenyl)-1-((4-methoxyphenyl)amino)propan-2-ol 40

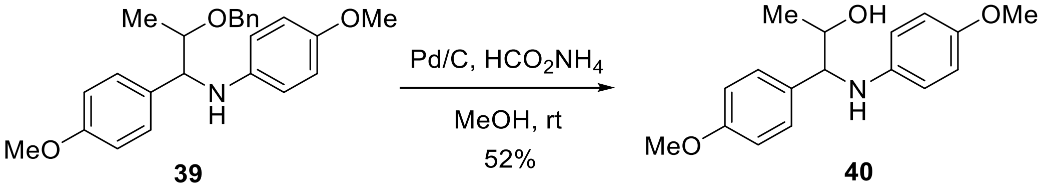

A suspension of protected **39** (188.5 mg, 0.5 mmol), ammonium formate (157.5 mg, 2.5 mmol, 5 eq.) and 10% Pd/C (30 mg) were stirred in methanol (5 mL) for 12 h at room temperature. The catalyst was filtered and washed with methanol twice. The combined washings and filtrate were evaporated in vacuo and the residue taken into DCM, washed with water, and dried over Na_2SO_4. The solvent was removed under reduced pressure and the residue was purified by column chromatography on silica gel to furnish the product **40** (74.6 mg, 52%), as an inseparable diastereomeric mixture (*dr* = 1:1.5, a colorless oil), and recovered the starting material **39** (77 mg).

Note: the reaction time is prolonged, there will still be residual raw materials; ^{1}H NMR (600 MHz, $CDCl_3$) δ 1.11 (d, J = 6.4 Hz, 3H), 1.18 (d, J = 6.2 Hz, 4.5H), 3.67-3.68 (m, 7.5 H), 3.75-3.76 (m, 7.5 H), 3.87-3.92 (m, 1.5 H), 4.02 (d, J = 6.5 Hz, 1.5H), 4.08-4.12 (m, 1H), 4.23 (d, J = 4.0 Hz, 1H), 6.50-6.55 (m, 5H), 6.67-6.69 (m, 5H), 6.83-6.86 (m, 5H), 7.19-7.25 (m, 5H) ppm; ^{13}C NMR (150 MHz, $CDCl_3$) δ 19.2, 19.8, 55.2, 55.6, 63.4, 65.5, 70.6, 71.7, 113.9, 114.1, 114.7, 115.0, 115.7, 128.0, 128.7, 131.0, 133.0, 141.2, 141.4, 152.1, 152.4, 158.9 ppm; HRMS (ESI) m/z calcd for $[C_{17}H_{21}NO_3Na]^+$ ($M + H^+$): 310.1414; found: 310.1422.

Ethyl 2-cyclohexyl-2-((4-methoxyphenyl)amino)acetate 41

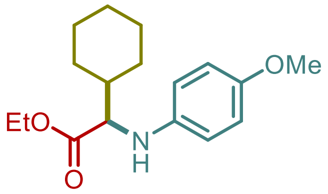

Following the **general procedure C**, 39.5 mg of **39**[27] as a colorless oil was obtained (yield: 68%);^{1}H NMR (600 MHz, $CDCl_3$) δ 1.14-1.32 (m, 8H), 1.69-1.89 (m, 6H), 3.75 (s, 3H), 3.79 (d, J = 6.2 Hz, 1H), 3.91 (br. s, 1H), 4.17 (q, J = 7.1 Hz, 2H), 6.62 (d, J = 8.7 Hz, 1H), 6.78 (d, J = 8.7 Hz, 1H) ppm; ^{13}C NMR (150 MHz, $CDCl_3$) δ 14.2, 25.9, 26.0, 26.1, 29.1, 29.6, 41.2, 55.6, 60.6, 63.3, 114.7, 115.1, 141.6, 152.5, 173.9 ppm;

Ethyl 3-(2',6'-dimethoxy-[1,1'-biphenyl]-4-yl)-2-((4-methoxyphenyl)amino)propanoate 42

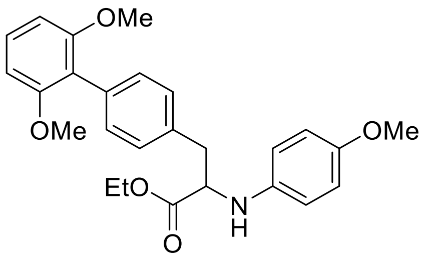

Following the **general procedure C**, 53 mg of **42**[27] as a colorless oil was obtained (yield: 61%); ^{1}H NMR (600 MHz, $CDCl_3$) δ 1.22 (t, J = 7.1 Hz, 3H), 3.17 (dd, J = 6.7, 13.6 Hz, 1H), 3.22 (dd, J = 6.6, 13.6 Hz, 1H), 3.78 (s, 3H), 3.80 (s, 6H), 4.15-4.20 (m, 2H), 4.37 (t, J = 6.6 Hz, 3H), 6.67-6.69 (m, 2H), 6.70-6.72 (m, 2H), 6.82-6.84 (m,

2H), 7.29-7.35 (m, 5H) ppm; ^{13}C NMR (150 MHz, $CDCl_3$) δ 13.9, 38.9, 55.6, 55.7, 58.9, 60.9, 104.1, 114.8, 115.2, 119.0, 128.5, 128.6, 131.0, 132.7, 134.7, 140.6, 152.7, 157.6, 173.8 ppm; HRMS (ESI) *m/z* calcd for $[C_{26}H_{30}NO_5]^+$ (M + H^+): 436.2118; found: 436.2124.

Ethyl 2-((4-methoxyphenyl)amino)-2-(tetrahydrofuran-2-yl)acetate 41

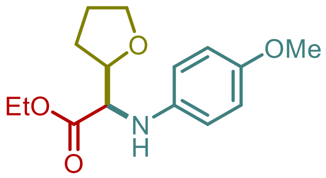

Following the **general procedure C**, 33.5 mg of **43**[28] as a colorless oil was obtained (yield: 60%); *dr* = 1:1.1; ^{1}H NMR (600 MHz, $CDCl_3$) δ 1.23-1.26 (m, 3H), 1.90-2.05 (m, 4H), 3.75 (s, 3H), 3.79-4.02 (m, 3H), 4.18-4.34 (m, 3H), 6.62-6.69 (m, 2H), 6.77-6.79 (m, 2H) ppm; ^{13}C NMR (150 MHz, $CDCl_3$) δ 14.1, 14.2, 25.5, 26.0, 28.0, 28.3, 55.6, 60.9, 61.0, 61.1, 61.8, 68.6, 69.2, 114.7, 114.8, 115.2, 115.4, 141.0, 141.4, 152.7, 152.8, 172.4, 172.7 ppm;

Ethyl (4-methoxyphenyl)phenylalaninate-*d2* 44

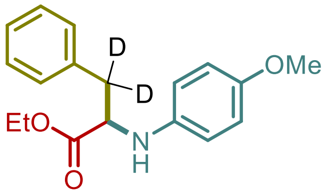

Following the **general procedure C**, except the deuterated carboxylic acid **3aae-d2** used, 39 mg of **44** as a colorless oil was obtained (yield: 65%); ^{1}H NMR (600 MHz, $CDCl_3$) δ 1.18 (t, *J* =7.1 Hz, 3H), 3.76 (s, 3H), 4.13 (q, *J* =7.1 Hz, 2H), 4.28 (s, 1H), 6.61 (d, *J* =8.8 Hz, 2H), 6.79 (d, *J* =8.8 Hz, 2H), 7.21-7.23 (m, 2H), 7.27-7.28 (m, 1H), 7.31-7.34 (m, 2H) ppm; ^{13}C NMR (150 MHz, $CDCl_3$) δ 14.0, 55.6, 55.8, 60.9, 114.8, 115.2, 126.9, 128.4, 129.2, 136.4, 140.5, 152.7, 173.4 ppm. HRMS (ESI) *m/z* calcd for $[C_{18}H_{19}D_2NO_3Na]^+$ (M + H^+): 324.1539; found: 324.1546.

4. Mechanistic studies

4.1 The activity of TiO_2 (P25), anatase, and rutile.

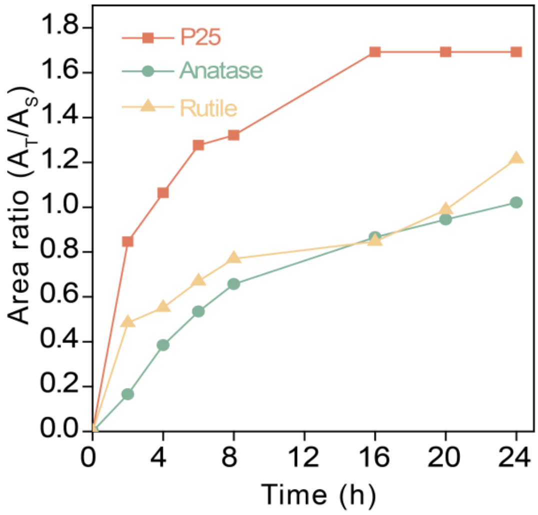

Figure S4. Peak area ratio (determined by GC-MS) of product with time over (a) P25, (b) anatase, and (c) rutile. Reaction conditions: 0.5 mmol of *N*-benzylideneaniline, 1.0 mmol of cyclohexanecarboxylic acid, 100 mg TiO_2, 0.5 mmol anisole as internal standard, 8 mL MeCN, N_2 atmosphere, irradiation under 390 nm wavelength.

4.2 DRIFT analysis

The Diffuse Reflectance Infra-red Fourier Transform (DRIFT) measurements were conducted on a Bruker Tensor II spectrometers. Hexahydrobenzoic acid was adsorbed on the TiO_2 (P25) surface as follows: hexahydrobenzoic acid was added dropwise onto TiO_2 (P25), then the sample was heated to 80 ℃ under vacuum for 2 h.

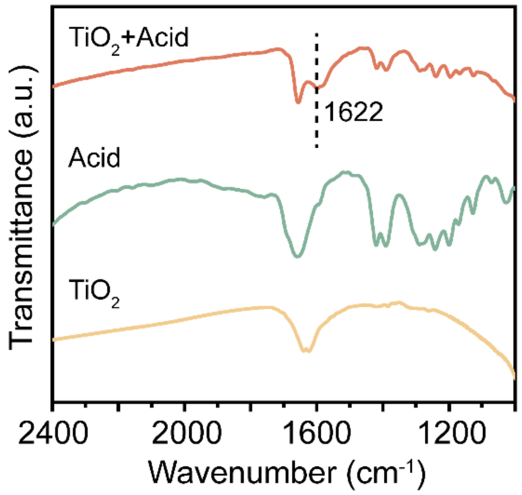

Figure S5. DRIFT spectrum of TiO_2 (P25) after adsorption of hexahydrobenzoic acid and free acid.

The *in-situ* Diffuse Reflectance Infra-red Fourier Transform (DRIFT) under light irradiation measurements were conducted on a Bruker Tensor II spectrometers and a 390 nm Kessil LEDs. Hexahydrobenzoic acid was adsorbed on the TiO_2 (P25) surface as follows: hexahydrobenzoic acid was added dropwise onto TiO_2 (P25), then the sample was heated to 80 ℃ under vacuum for 2 h. Following the pre-treatment, the sample cell was maintained at 30 ℃ and a 390 nm Kessil LEDs was irradiated through the window of the cell directly onto the sample. Spectra were respectively recorded at 0, 20, 40, 60, 80, 100 min.

4.3 Transient absorption decay analysis

Time-dependent photoluminescence spectroscopy was conducted on a FS5 fluorescence spectrometer (Edinburgh Instruments). The comparison of transient absorption decay among P25, anatase, and rutile were recorded at 2,000 cm^{-1} under O_2 and AcOH atmosphere (20 torr).

4.4 Density functional theory (DFT) calculations

The DFT calculations of oxygen vacancy (O_v) formation energy was carried out in the Vienna *ab initio* simulation (VASP6.3.0) code. The exchange-correlation is simulated with PBE functional, and the ion-electron interactions were described by the PAW method. The vdWs interaction was included by using empirical DFT-D3 method. The Monkhorst-Pack-grid-mesh-based Brillouin zone k-points are set as 3×3×1 for all periodic structure with the cutoff energy of 450 eV. The convergence criteria are set as 0.02 eV A^{-1} and 10^{-5} eV in force and energy, respectively.

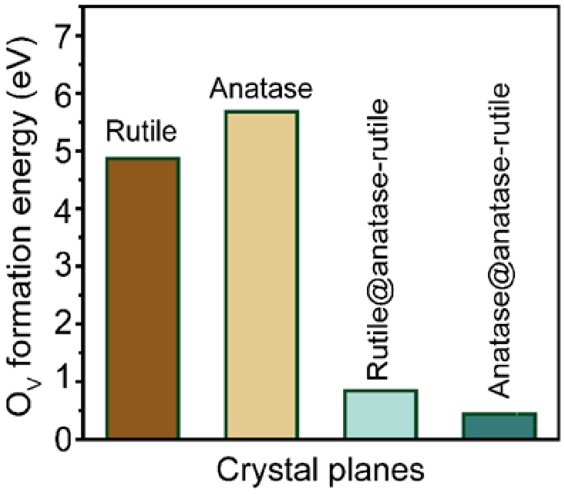

Figure S6. The oxygen vacancy formation energy for different TiO_2.

4.5 The TPD-MS experiments

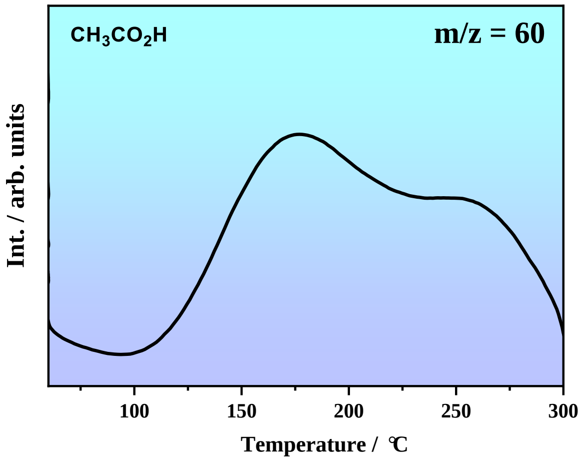

Figure S7. TPD analysis of CH_3CO_2H adsorption on P25.

The CH_3CO_2H-TPD of P25 was accomplished by using a chemisorption analyzer (HIDEN analytical CATALAB) coupled with a MS (HIDEN analytical QGA). The specific steps and details of the $MeCO_2H$-TPD experiments are described as follows: First, the P25 powder samples were soaked with $MeCO_2H$ and vacuum dried overnight in RT. Then a 50 mg of sample was transferred into a glass U-tube. Prior to temperature raising, the sample was purged by He flow for 0.5 h. The TPD profile of water ($m/e^- = 60$) was collected from 60 to 300 °C with a ramping rate of 10 °C min^{-1}. Helium was used as the carrier gas with a flow rate of 20 mL min^{-1} in the whole process.

4.6 Controlled synthesis of TiO_2 with tunable rutile/anatase phase ratios.

P25-*x* was synthesized based on our previously reported method.[29] Briefly, the commercial P25 (100 mg) was dispersed in 50 mL 6 M HCl solution and then transferred to a Teflon-line stainless-steel autoclave (100 mL), which was later heated at 200 °C for 1, 3, 6 h. Accordingly, the samples were designated as P25-*x*, where *x* denotes the hydrothermal treatment duration in hours. After hydrothermal reaction, the resulting precipitates were collected and washed with deionized water and ethanol for three times respectively and dried at 60 °C in a vacuum oven for 24 h.

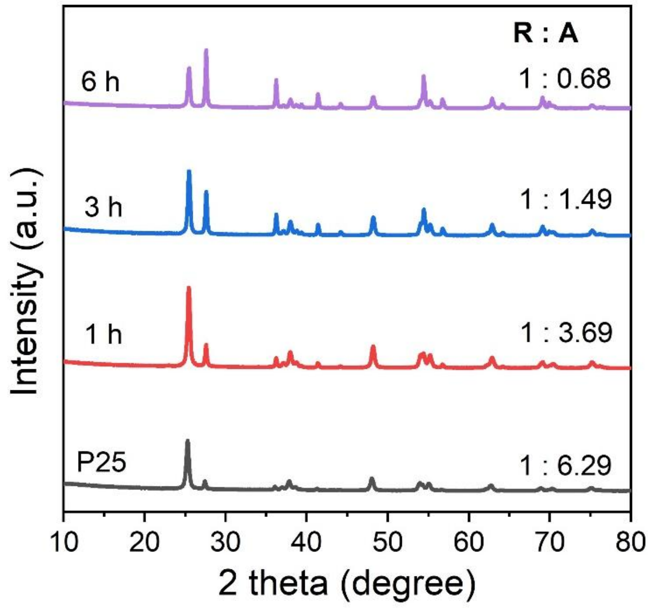

Figure S8. XRD patterns of TiO_2 (P25) after hydrothermal treatment at different time

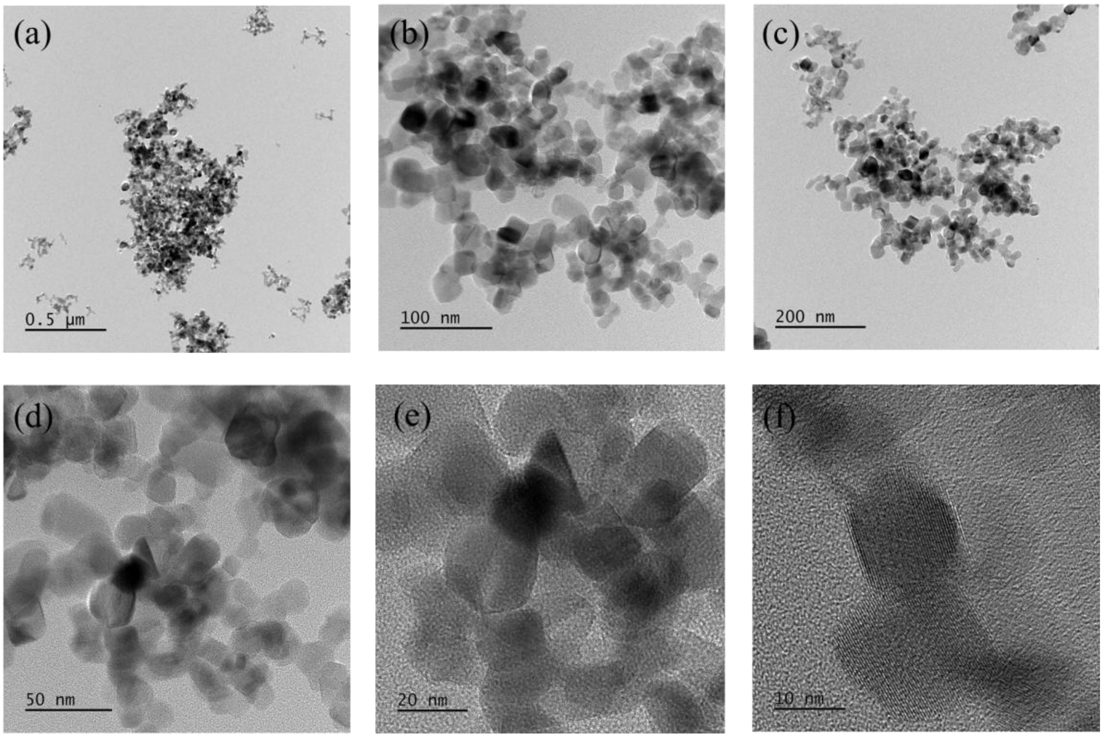

Figure S9. TEM images of P25-3.

The photocatalytic activity test conditions: 0.2 mmol of *N*-benzylideneaniline, 0.4 mmol of cyclohexanecarboxylic acid, 40 mg P25-*x*, 0.2 mmol anisole as internal standard, 3 mL MeCN, N_2 atmosphere, irradiation under 390 nm wavelength.

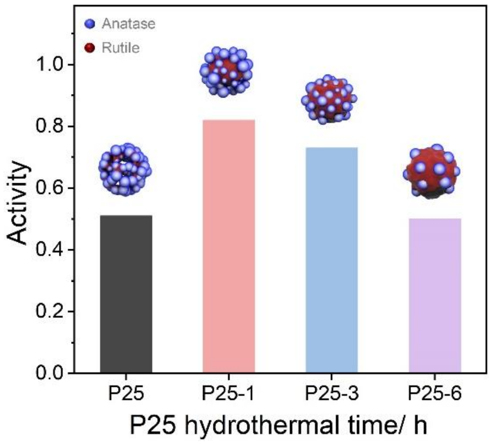

Figure S10. The photocatalytic activity activity of P25, P25-1, P25-3, P25-6.

Note: The vertical axis represents the ratio of product to peak area (determined by GC-MS).

Recycling experiments

Following **General Procedure A**, the reaction mixture was stirred for 24 h. TiO_2 was separated by centrifugation and washed with MeCN and deionized water. The organic phase was purified by column chromatography to afford the target product, which was used to determine the yield. The TiO2 was dried in a vacuum oven at 70 ℃ for 24 h. Subsequently, the dried TiO2 was evaluated for catalytic activity under standard reaction conditions. *Note: In the fifth cycle, the reaction time was extended to 48 h.*

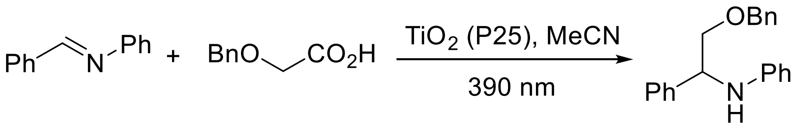

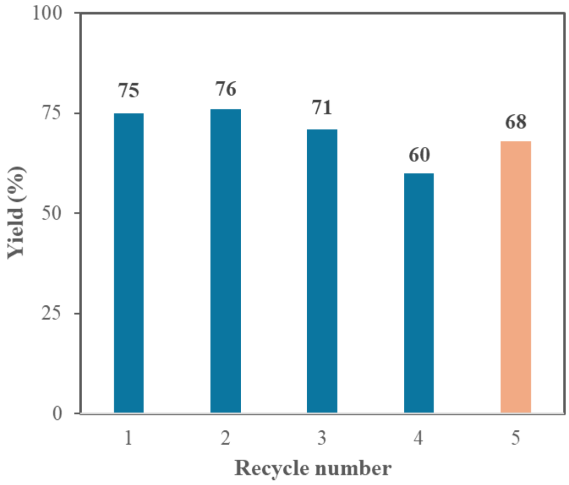

Figure S11. Recycling experiments.

5. References

1. J. M. Farrell, R. T. Posaratnanathana, D, W. Stephan, Chem. Sci., **2015**, *6*, 2010–2015.

2. K. Kutlescha, T. Irrgang, R. Kempe, *Adv. Synth. Catal.* **2010**, *352*, 3126 – 3130.

3. S. N. Gockel, S. Lee, B. L. Gay, K. L. Hull, *ACS Catal.* **2021**, *11*, 5166−5171.

4. Z. Li, L. Zhang, M. Nishiura, Z. Hou, *ACS Catal.* **2019**, *9*, 4388−4393.

5. R. Kancherla, K. Muralirajan, M. Rueping, *Chem. Sci.* **2022**, *13*, 8583–8589.

6. D. P. Plasko, C. J. Jordan, B. E. Ciesa, M. A. Merrill, J. M. Hanna, *Photochem. Photobiol. Sci.* **2018**, *17*, 534–538.

7. F. J. Lakner, M. A. Parker, B. Rogovoy, A. Khvat, A. Ivachtchenko, *Synthesis* **2009**, 1987–1990.

8. Y. Y. Huang, Z.-C. Lv, X. Yang, Z.-L. Wang, X.-X. Zou, Z.-N. Zhao, F. Chen, *Green Chem.* **2017**, *19*, 924–927.

9. Z. Huang, H. Jin, D. Duan, *Synthetic Commun.* **2002**, *32*, 565−570.

10. L. Leng, J. M. Ready, *ACS Catal.* **2020**, *10*, 13196–13201.

11. N. Chen, X. Dai, H. Wang, C.-J. Li, *Angew. Chem. Int. Ed.* **2017**, *56*, 6260−6263.

12. E. L. Gall, C. Haurena, S. Sengmany, T. Martens, M. Troupel, *J. Org. Chem.* **2009**, *74*, 7970–7973.

13. K. Yamada, Y. Yamamoto, K. Tomioka, *Org. Lett.* **2003**, *5*, 1797–1799.

14. J. Yi, S. O. Badir, R. Alam, G. A. Molander, *Org. Lett.* **2019**, *21*, 4853−4858.

15. W. Zhang, S. Ning, Y. Lia, X. Wu, *Chem. Commun.* **2022**, *58*, 12843–12846.

16. S. Xu, H. Guo, Y. Liu, W. Chang, J. Feng, X. He, Z. Zhang, *Org. Lett.* **2022**, *24*, 5546−5551.

17. S. Jiang, E. Muller, F. Jerôme, M. Pera-Titus, K. D. O. Vigier, *Green Chem.* **2020**, *22*, 1832−1836.

18. K. Shimizu, N. Imaiida, K. Kon, S. M. A. H. Siddiki, A. Satsuma, *ACS Catal.* **2013**, *3*, 998−1005.

19. B. Emayavaramban, P. Chakraborty, B. Sundararaju, *ChemSusChem* **2019**, *12*, 3089 –3093.

20. D. Wei, C. Netkaew, C. Darcel, *Adv. Synth. Catal.* **2019**, *361*, 1781−1786.

21. X. Zhu, J. He, Y. Yang, S. Zhou, Y. Wei, S. Wang, *Dalton T.* **2022**, *51*, 13227−13235.

22. M, Sengoden, T. Punniyamurthy, *RSC Adv.* **2012**, *2*, 2736−2738.

23. A. Pulcinella1, S. Bonciolini, R. Stuhr, D. Diprima1, M. T. Tran, M. Johansso, A. J. von Wangelin, T. Noël, *Nat. Commun.* **2025**, *16*, 948
24. P. Roszkowski, J. K. Maurin, Z. Czarnock, *Beilstein J. Org. Chem.* **2015**, *11*, 1509–1513.
25. S. W. Park, H. E.Kang, W. Yun, S. Y. Lee, T. Nam, *Tetrahedron Lett.* **2020**, *61*, 151451.
26. C. Weindl, S. L. Helmbrecht, L. Hintermann, *J. Org. Chem.* **2023**, *11*, 4155–4161.
27. Q. Li, H. Sun, F.Yan, Y. Zhao, Y. Zhang, C. Zhou, M. Han, H. Li, X. Sui, *Green Chem.* **2023**, *25*, 6226-6230.
28. H. Xiang, Y. Ye, *ACS Catal.* **2024**, *14*, 522−532.
29. J. Chen, H. B. Yang, H. B. Tao, L. Zhang, J. Miao, H.-Y. Wang, J. Chen, H. Zhang, B. Liu, *Adv. Funct. Mater.* **2016**, *26*, 456–465.

6. ^{1}H NMR and ^{13}C NMR spectra

^{1}H NMR and ^{13}C NMR spectra of compound **5a**

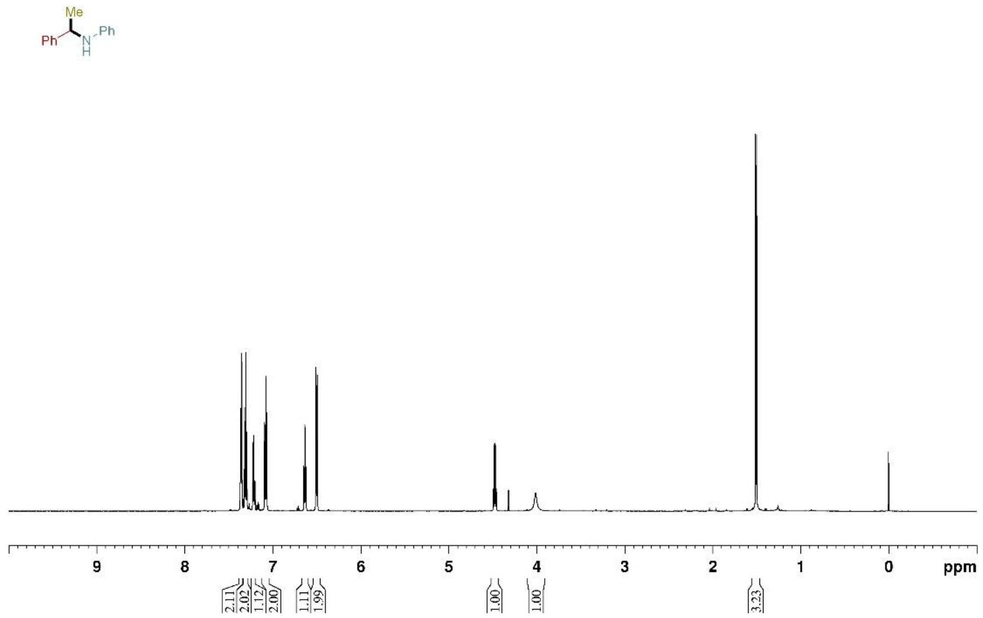

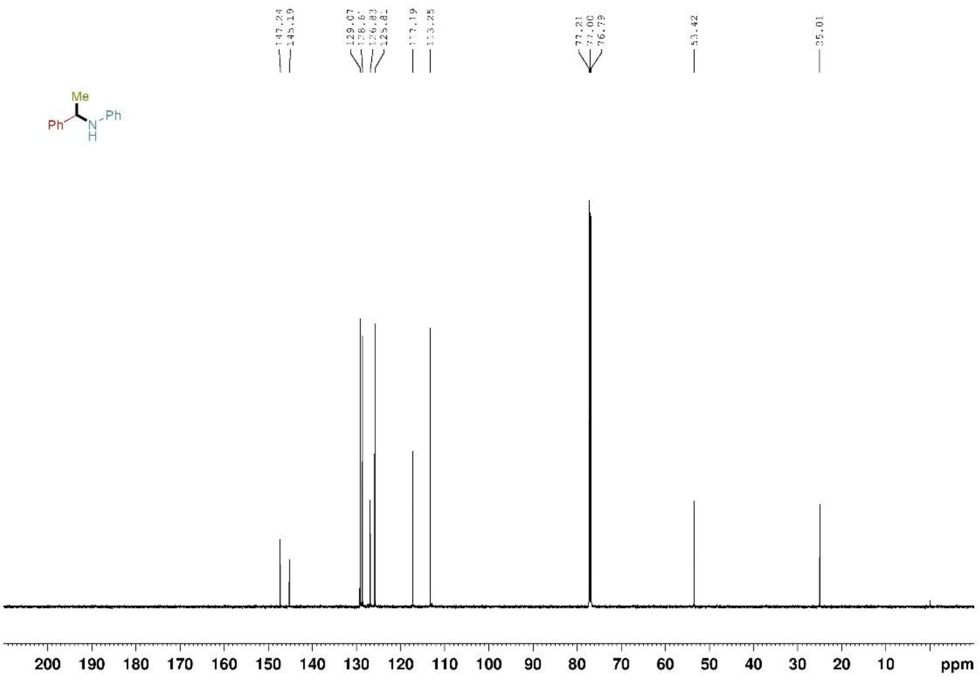

^{1}H NMR and ^{13}C NMR spectra of compound **5b**

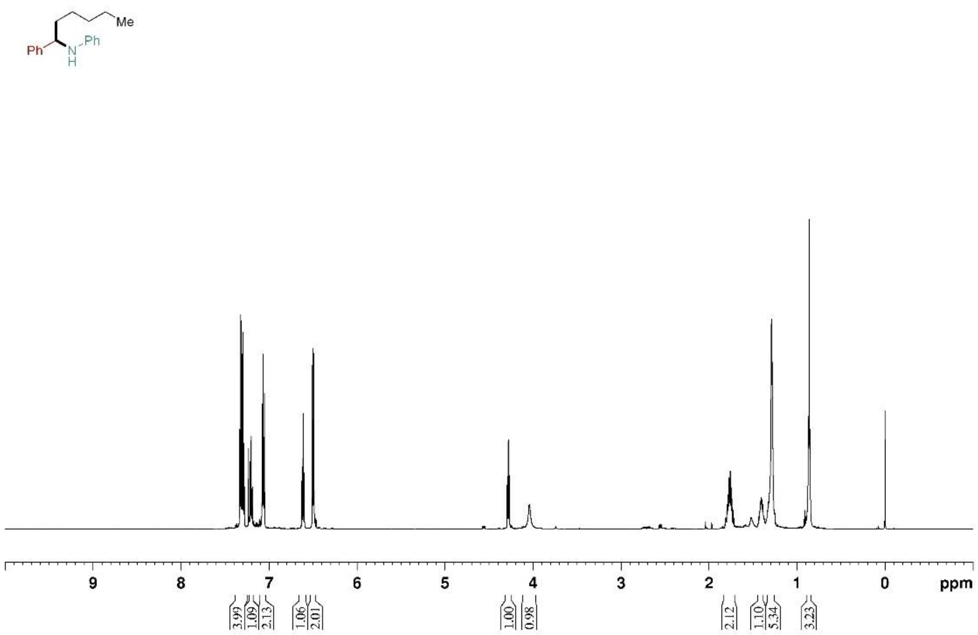

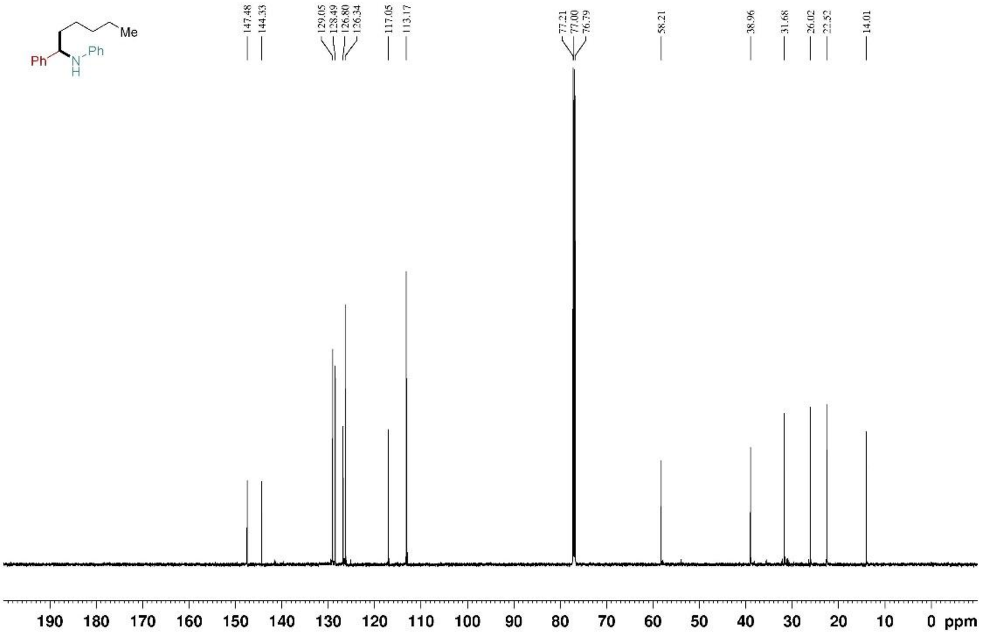

^{1}H NMR and ^{13}C NMR spectra of compound **5c**

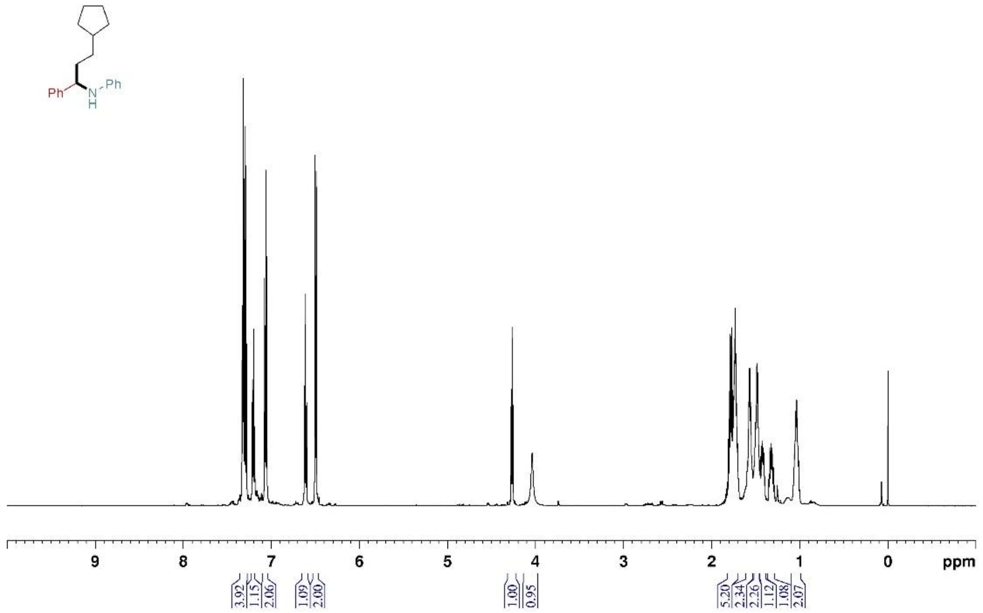

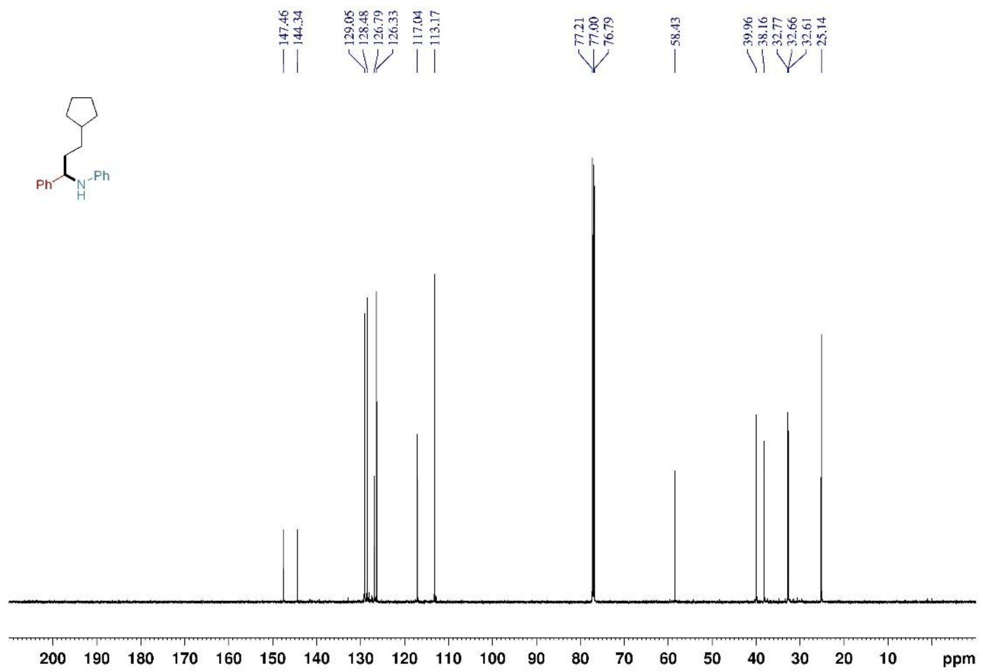

^{1}H NMR and ^{13}C NMR spectra of compound **5d**

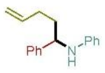

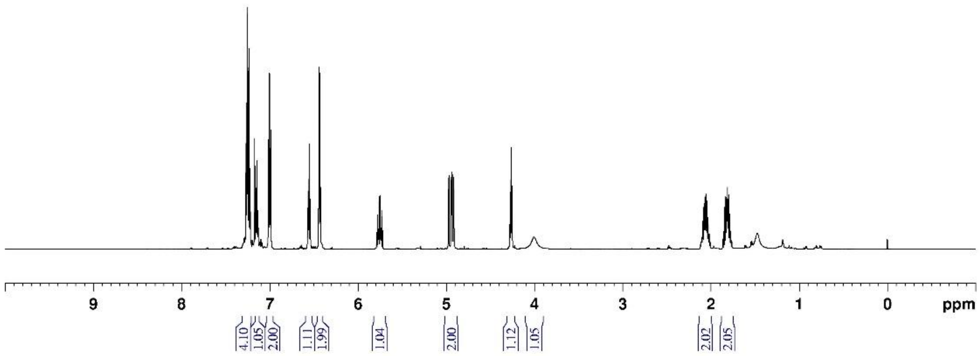

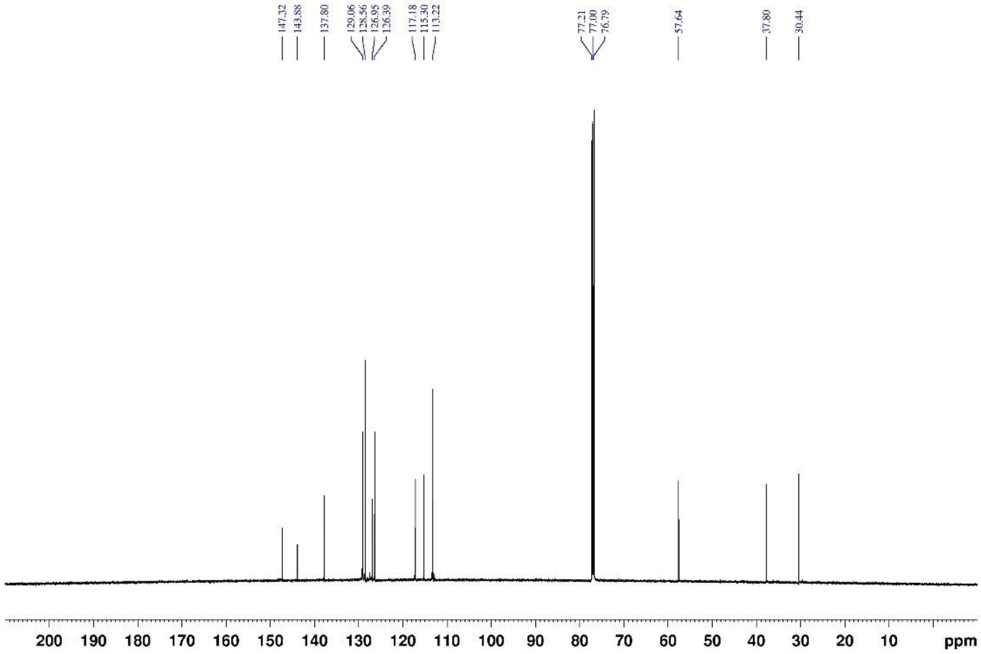

^{1}H NMR and ^{13}C NMR spectra of compound **5e**

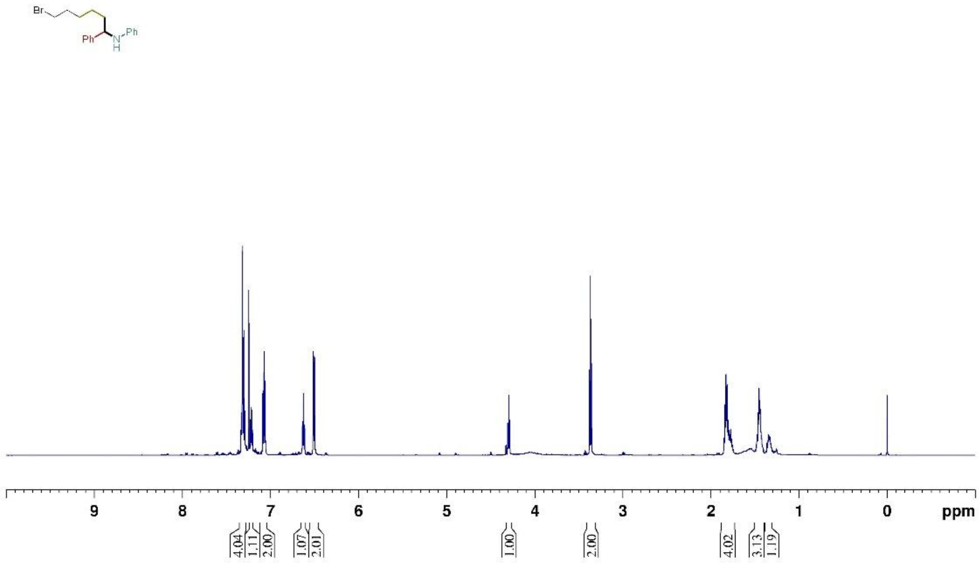

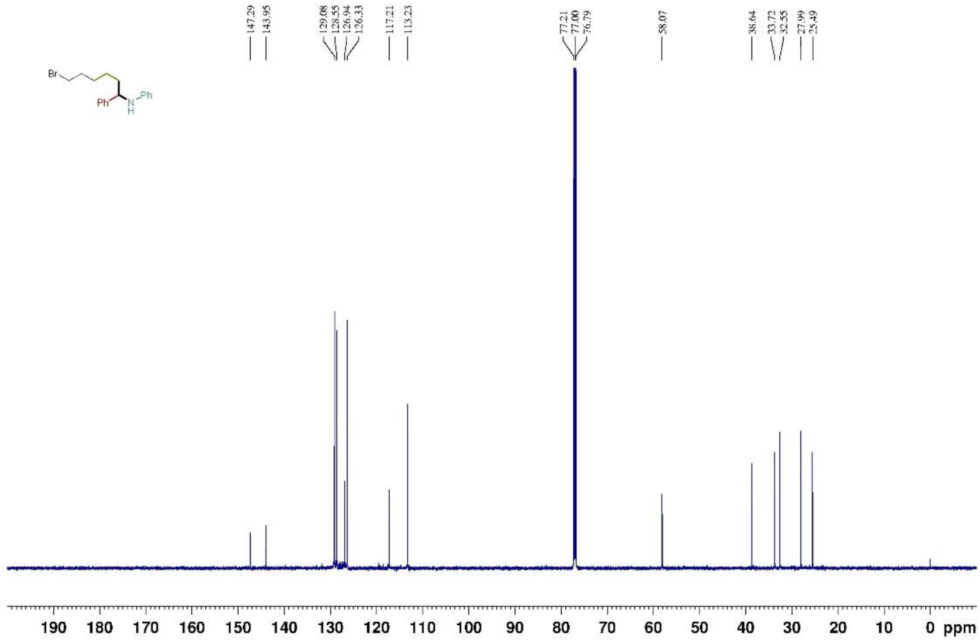

^{1}H NMR and ^{13}C NMR spectra of compound **5f**

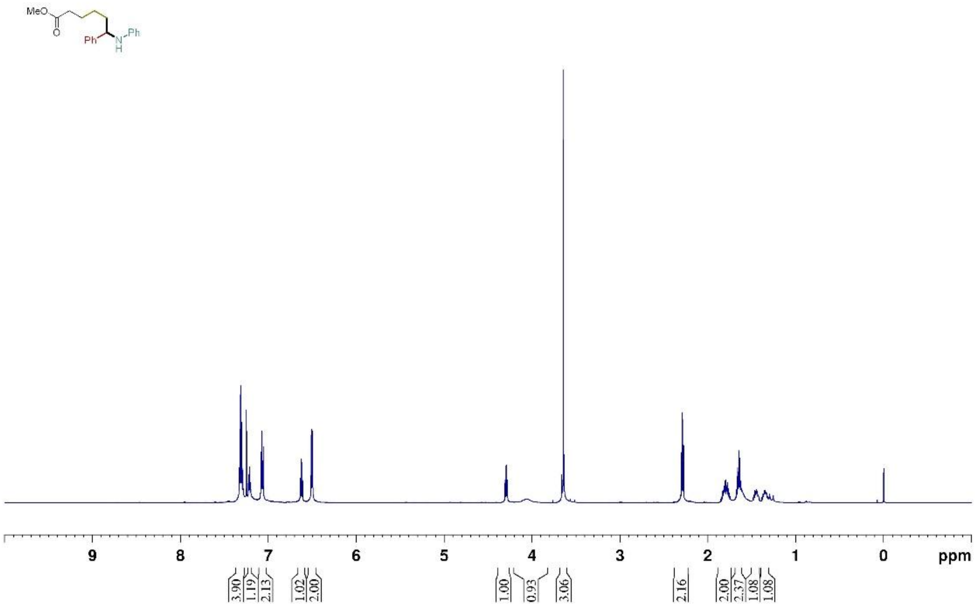

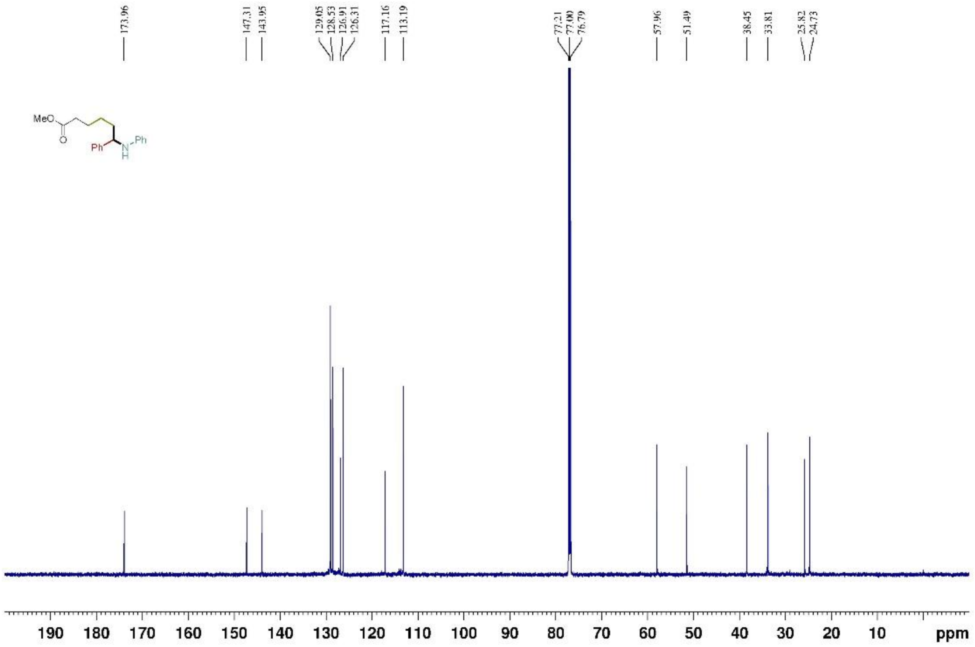

^{1}H NMR and ^{13}C NMR spectra of compound **5g**

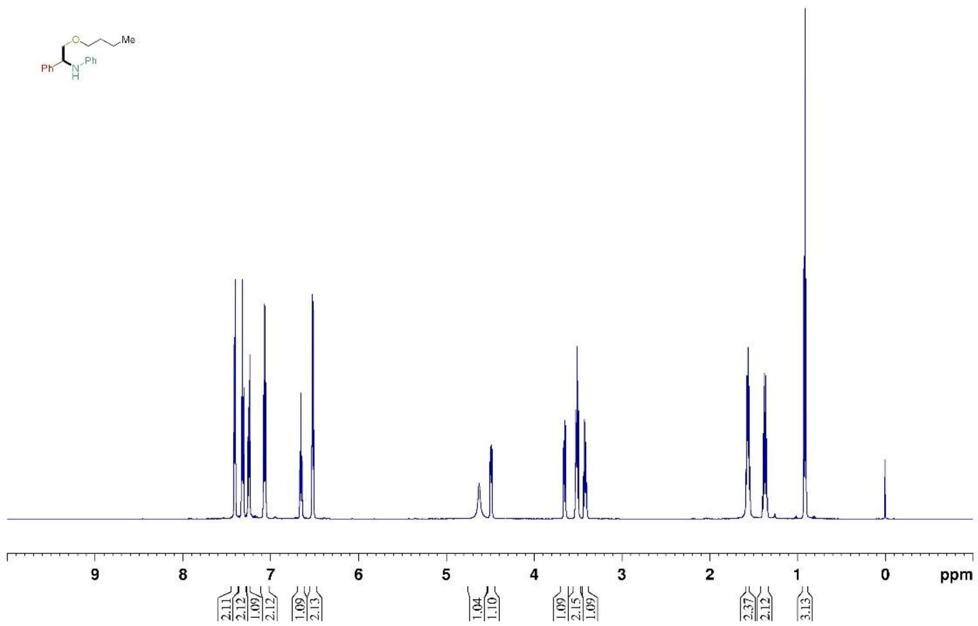

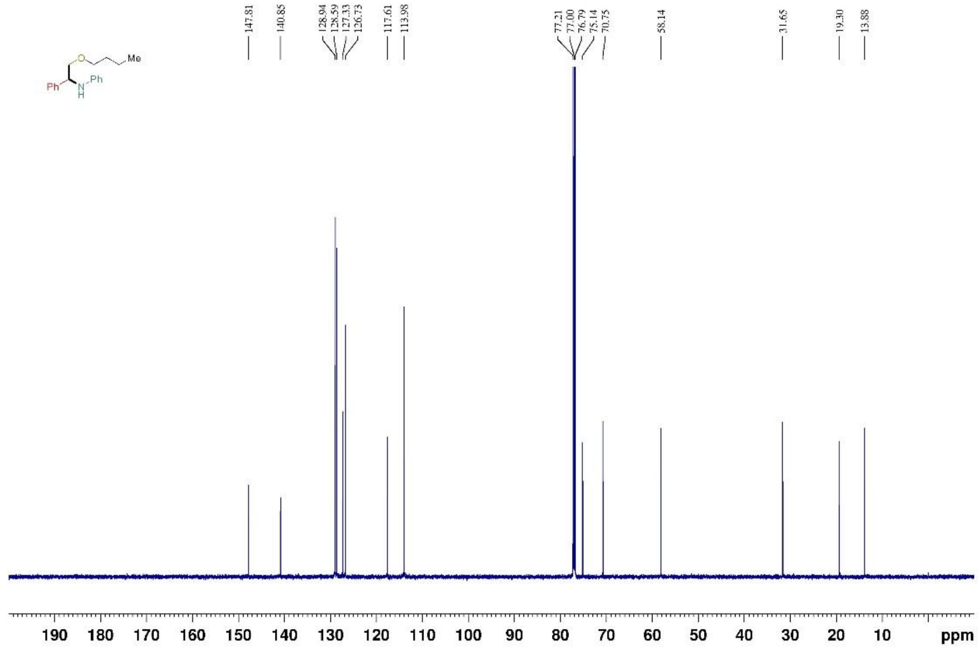

^{1}H NMR and ^{13}C NMR spectra of compound **5h**

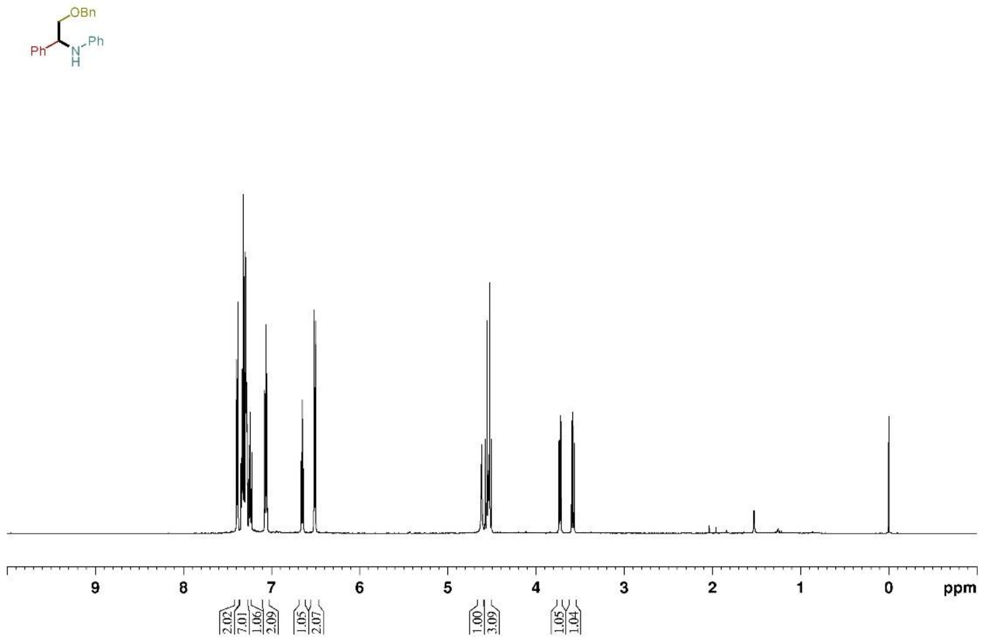

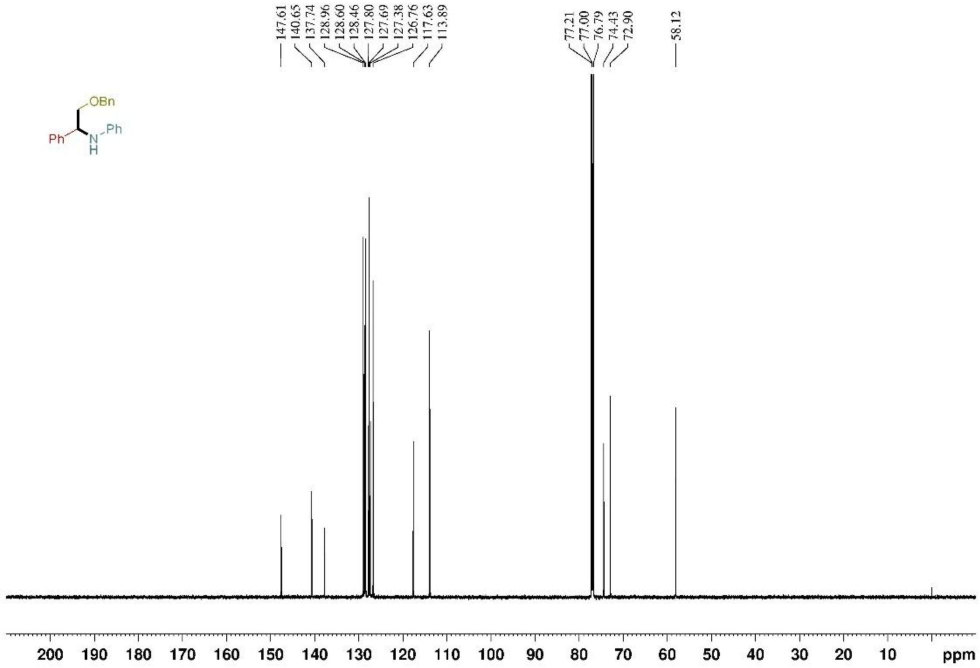

^{1}H NMR and ^{13}C NMR spectra of compound **5i**

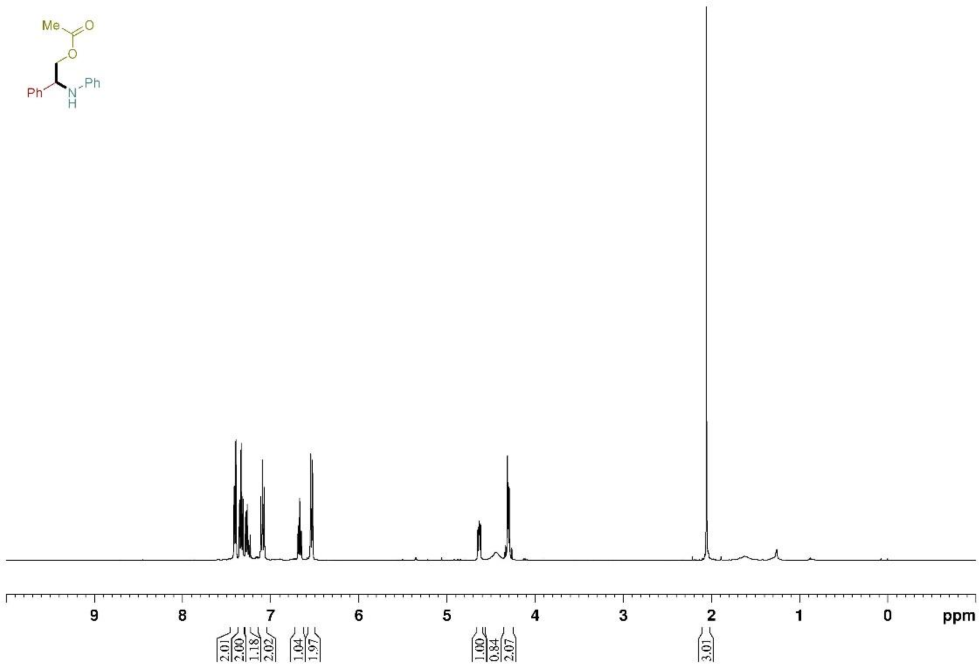

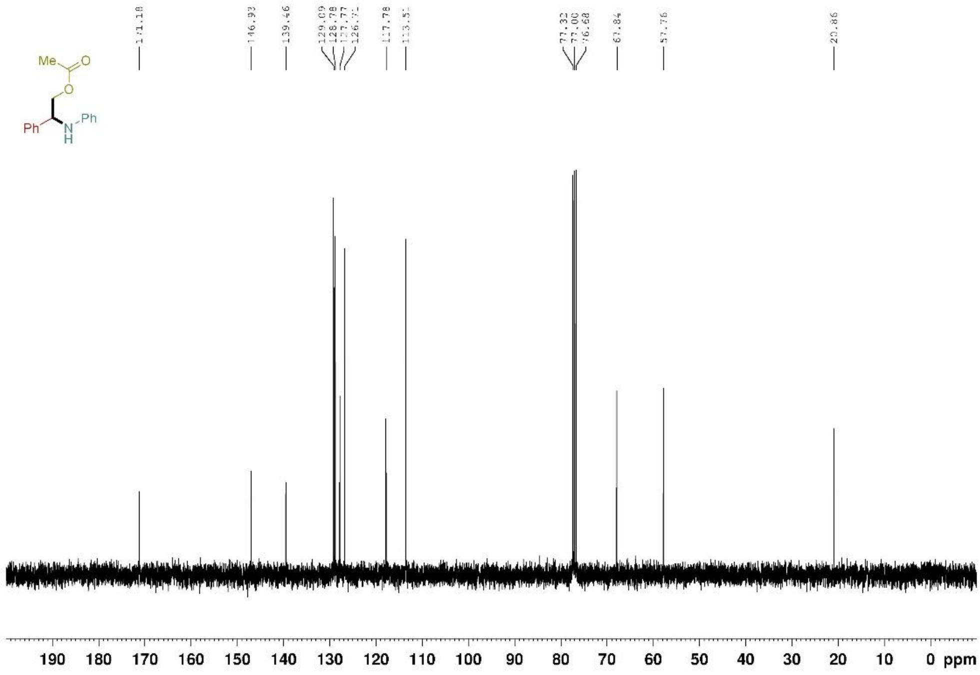

^{1}H NMR and ^{13}C NMR spectra of compound **5j**

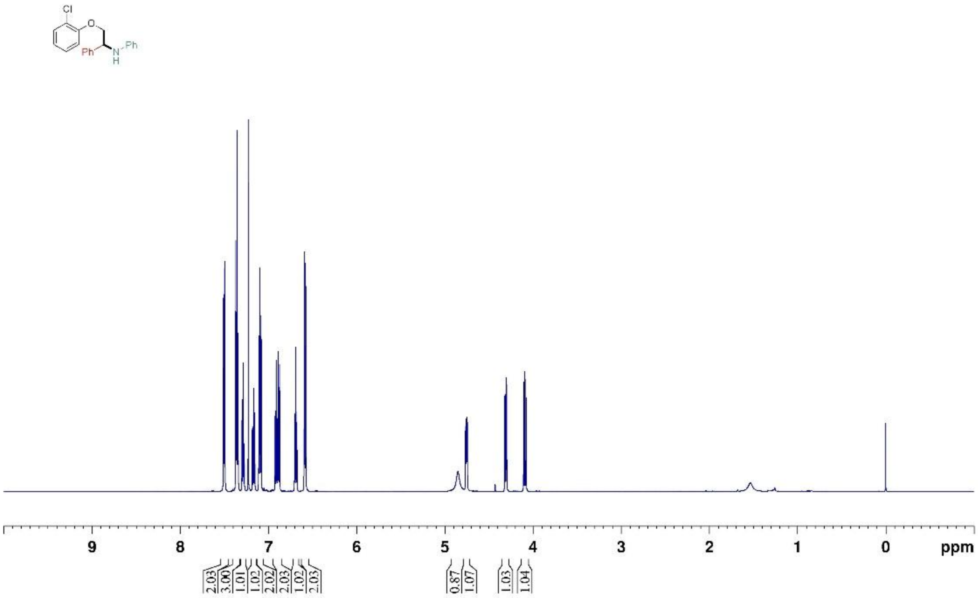

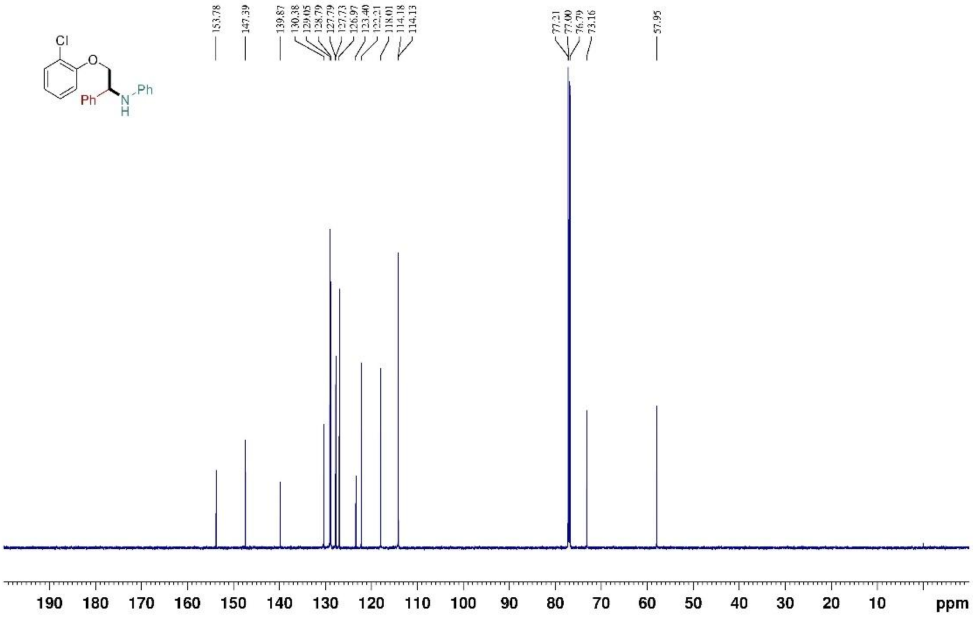

^{1}H NMR and ^{13}C NMR spectra of compound **5k**

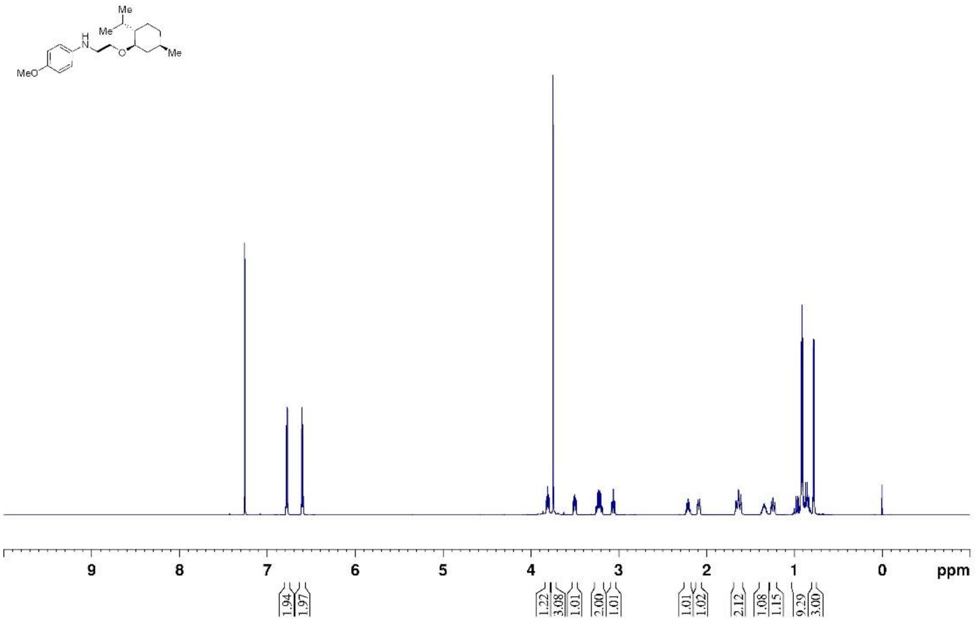

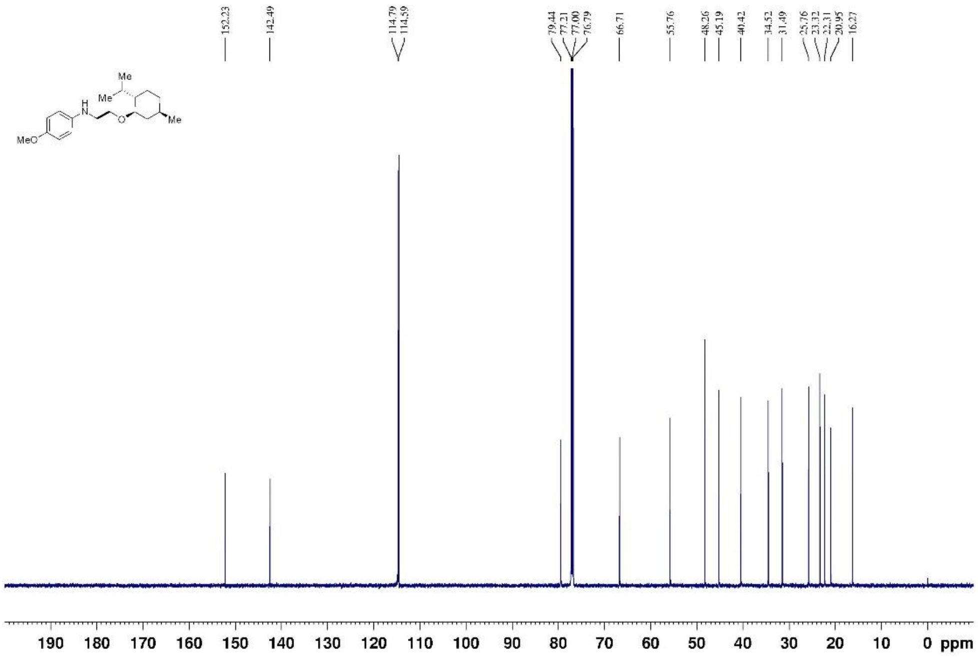

^{1}H NMR and ^{13}C NMR spectra of compound **5l**

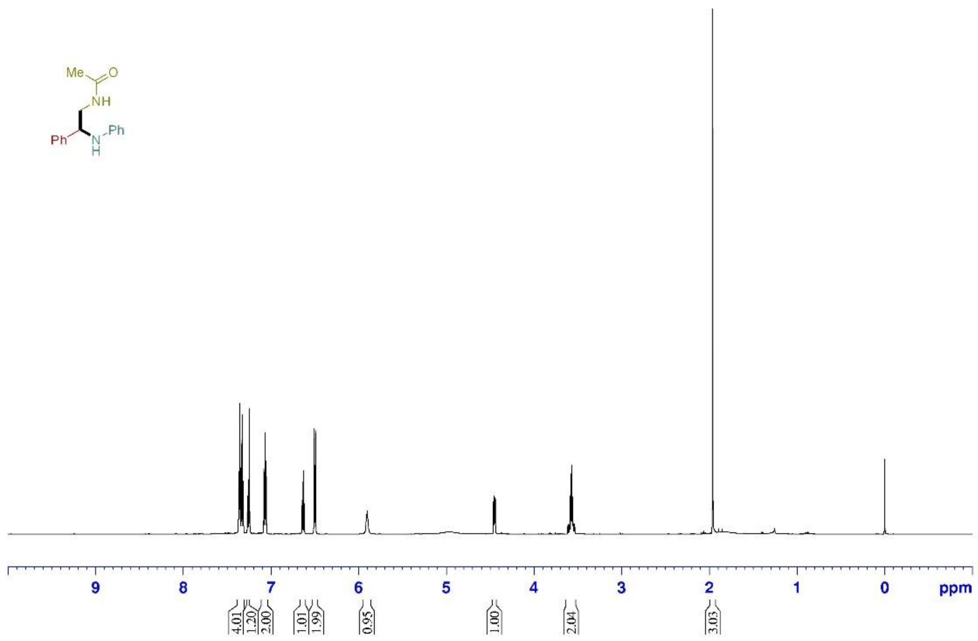

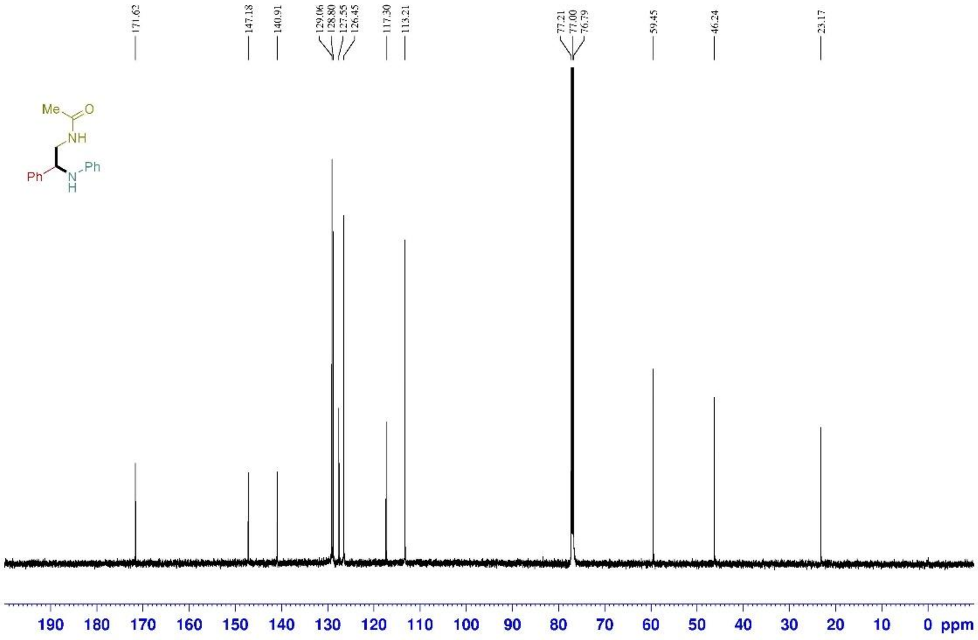

^{1}H NMR and ^{13}C NMR spectra of compound **5m**

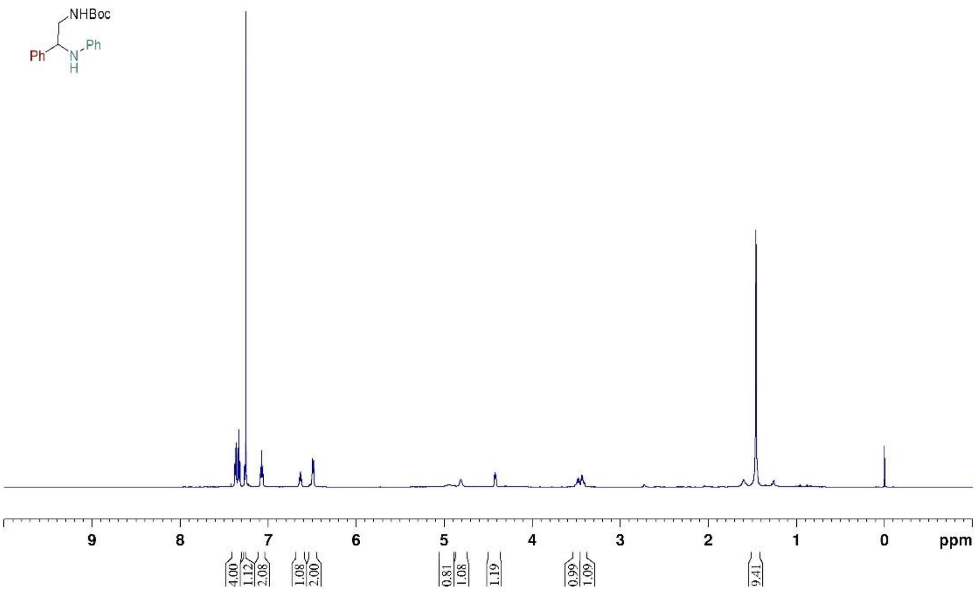

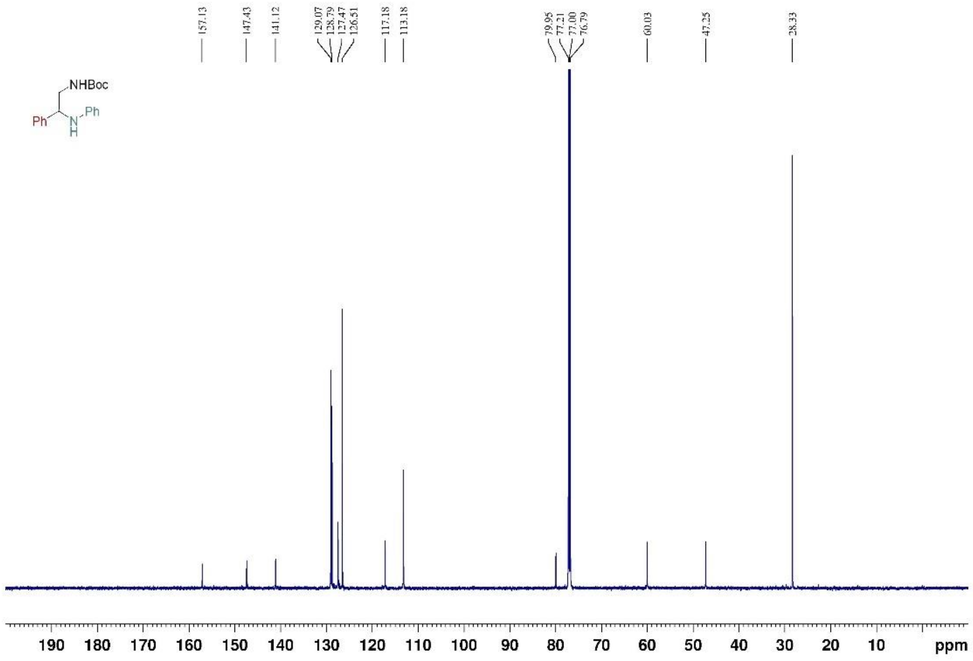

^{1}H NMR and ^{13}C NMR spectra of compound **5n**

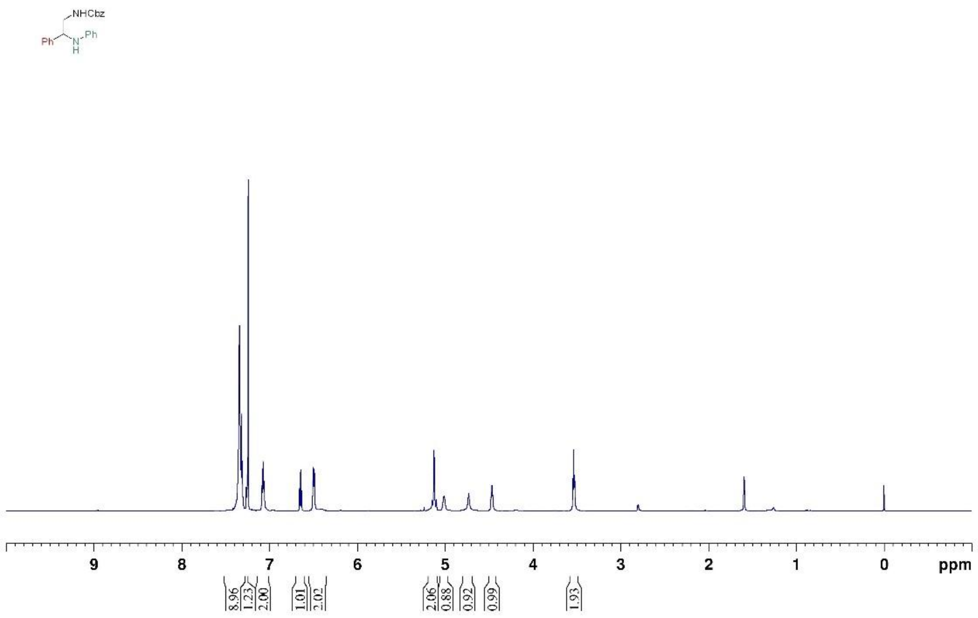

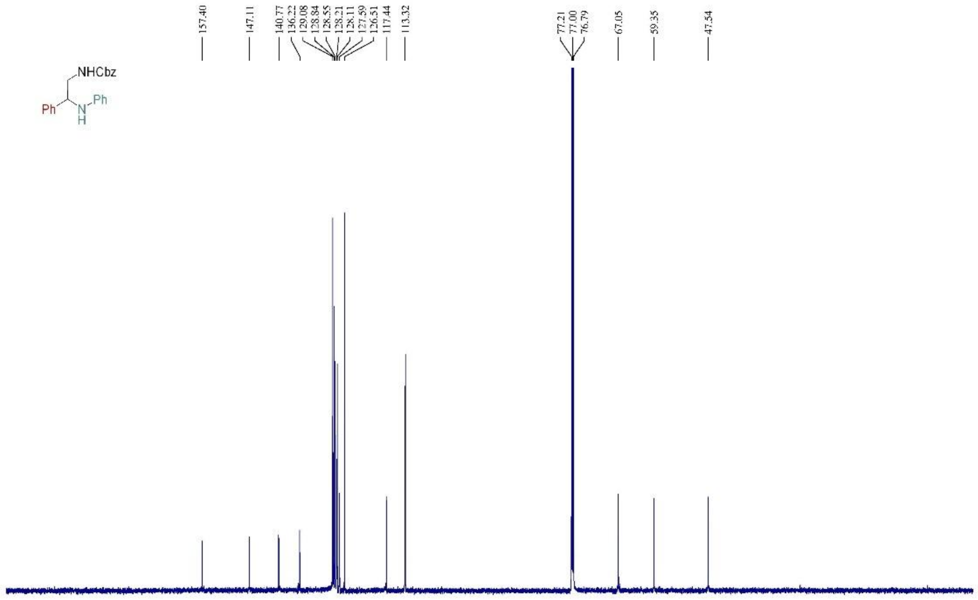

^{1}H NMR and ^{13}C NMR spectra of compound **5o**

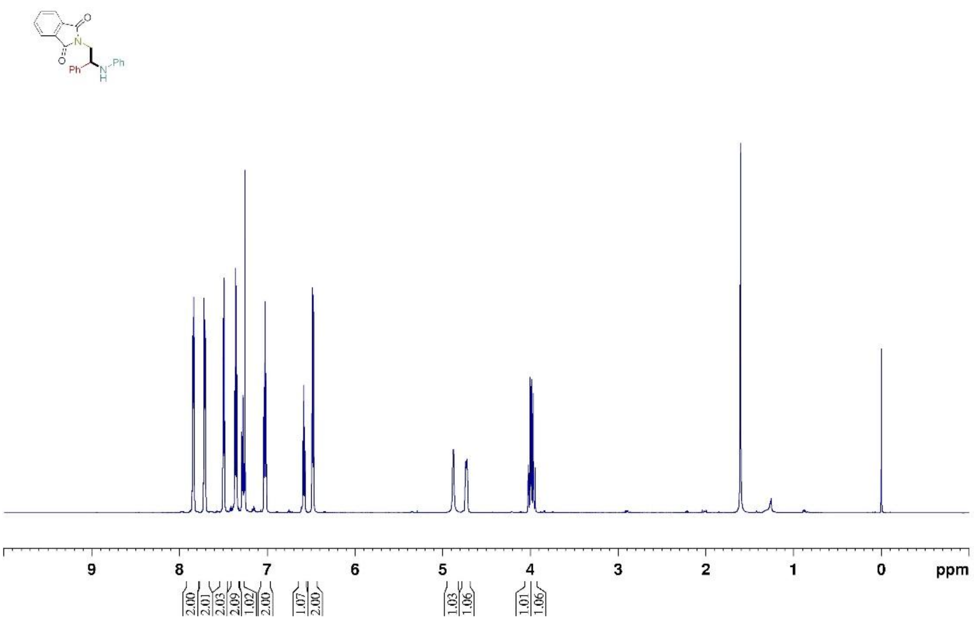

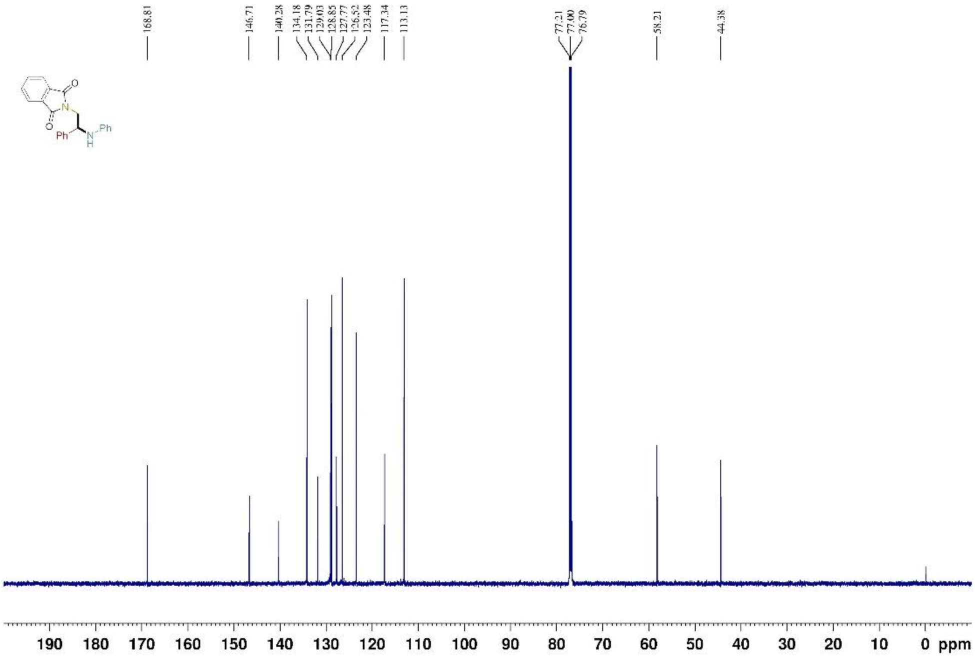

^{1}H NMR and ^{13}C NMR spectra of compound **5p**

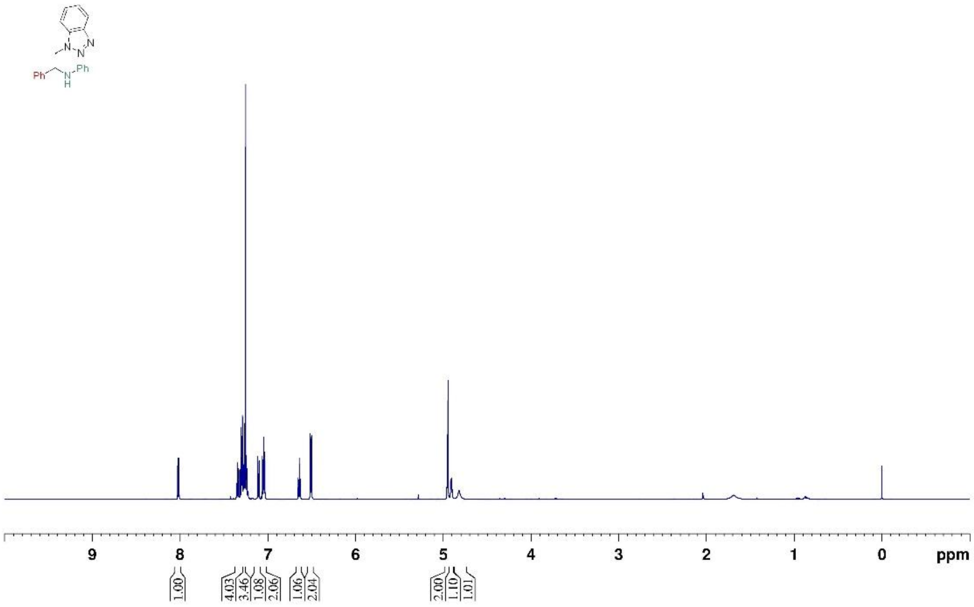

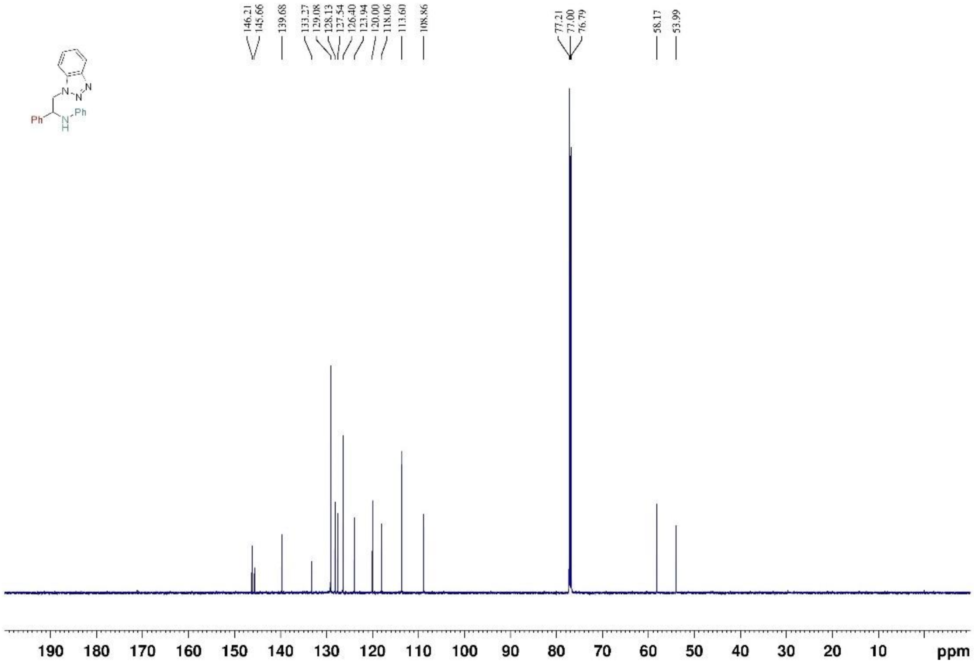

^{1}H NMR and ^{13}C NMR spectra of compound **5q**

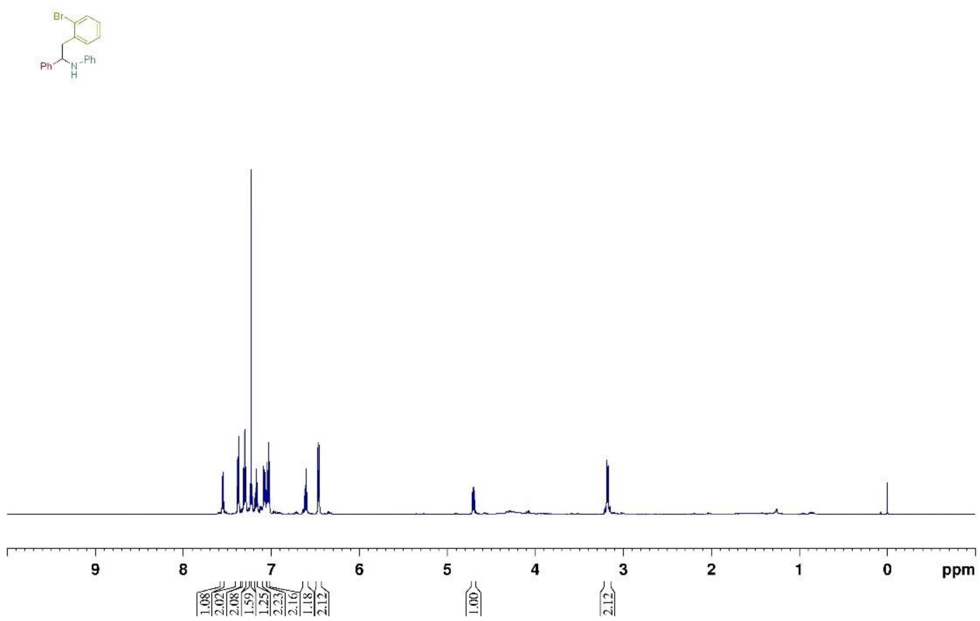

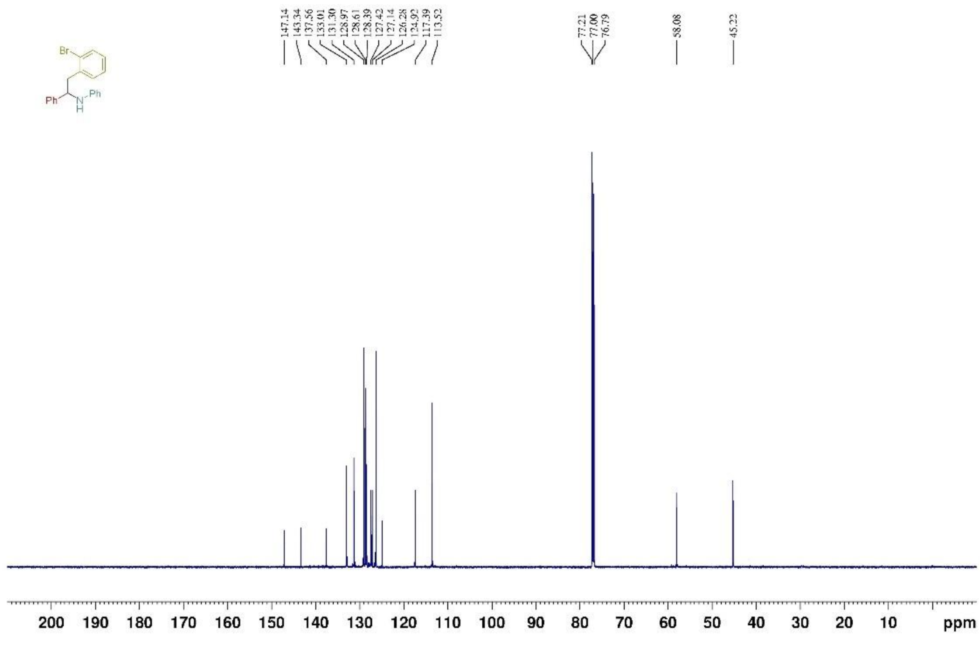

^{1}H NMR and ^{13}C NMR spectra of compound **5r**

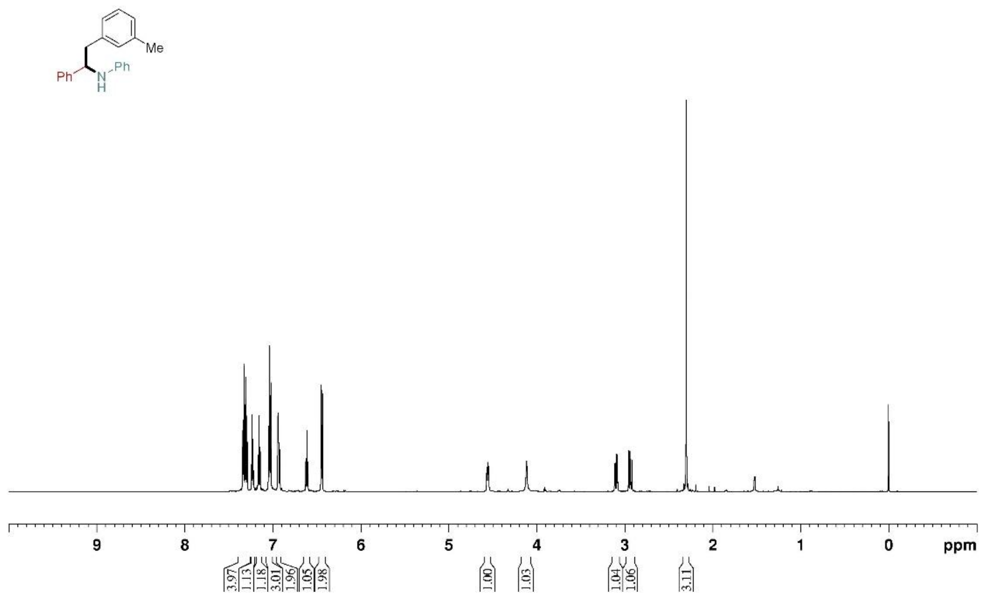

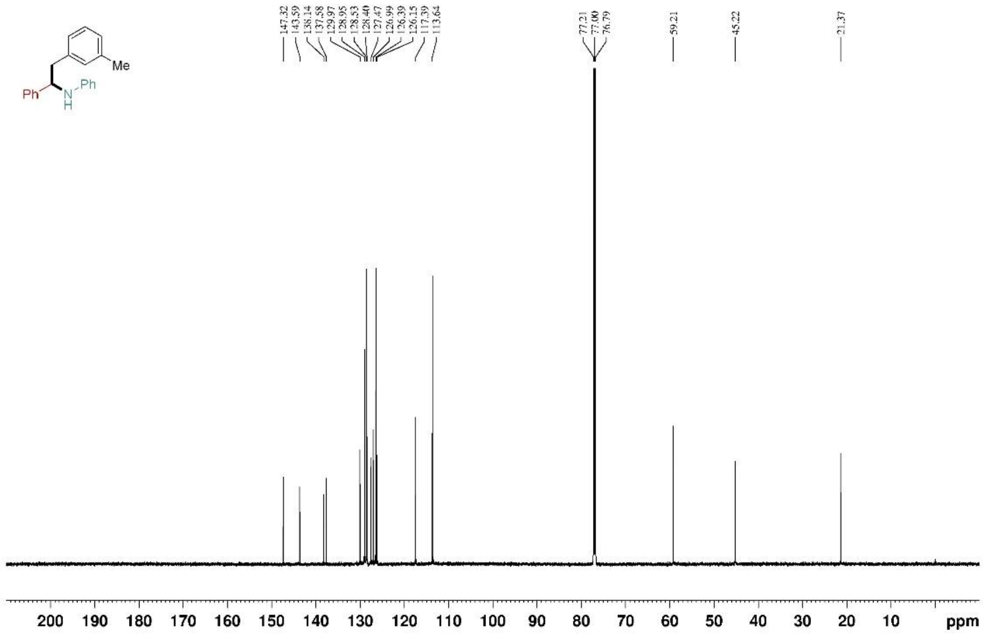

^{1}H NMR and ^{13}C NMR spectra of compound **5s**

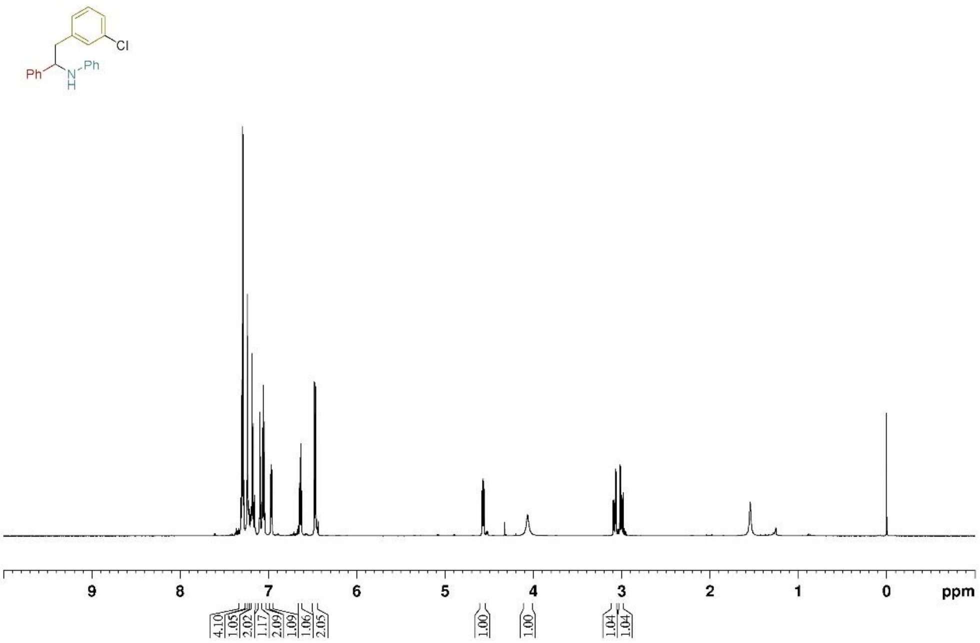

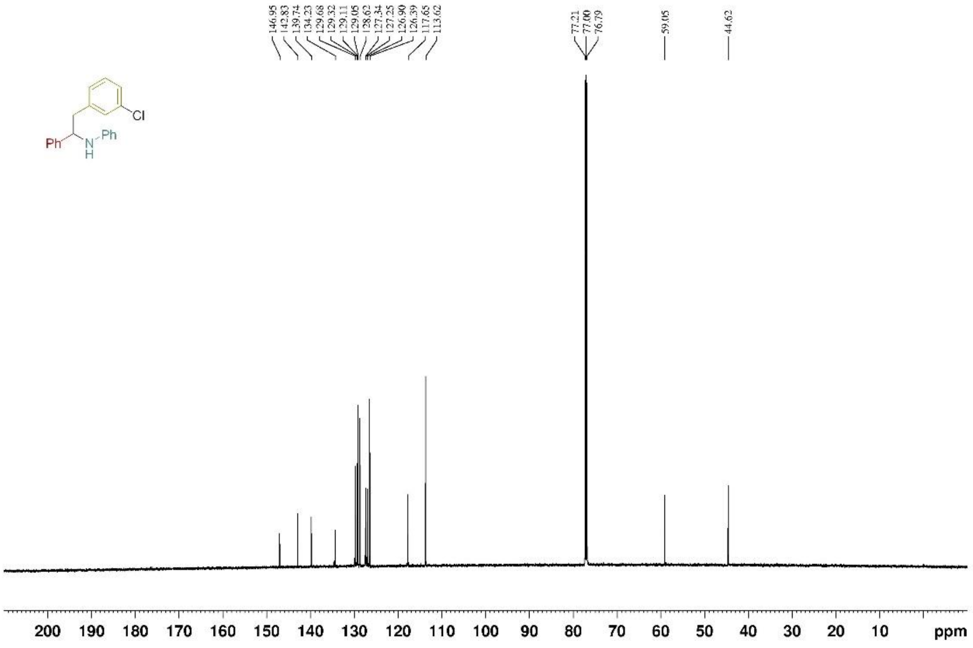

^{1}H NMR and ^{13}C NMR spectra of compound **5t**

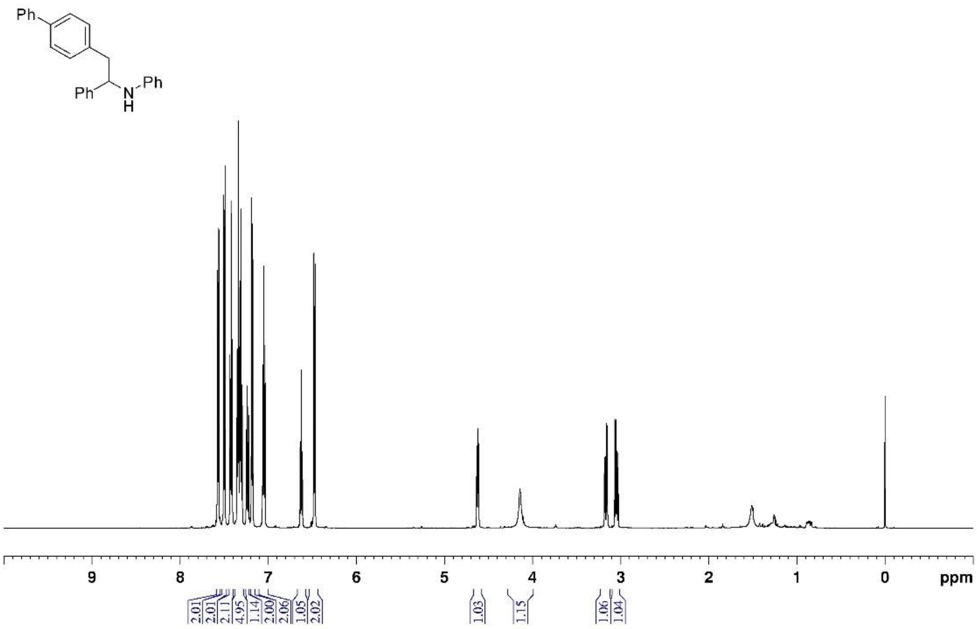

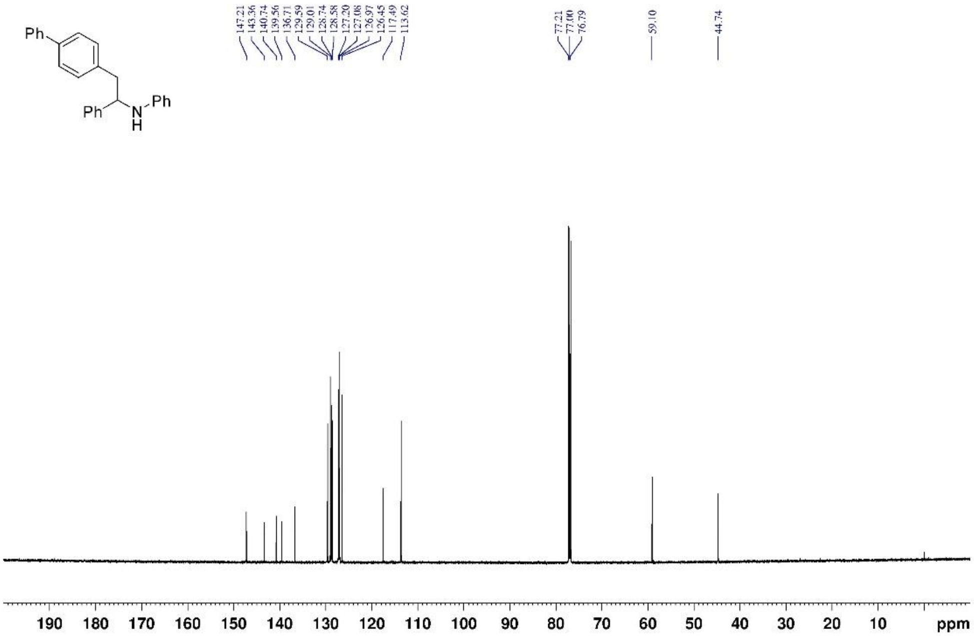

^{1}H NMR and ^{13}C NMR spectra of compound **5u**

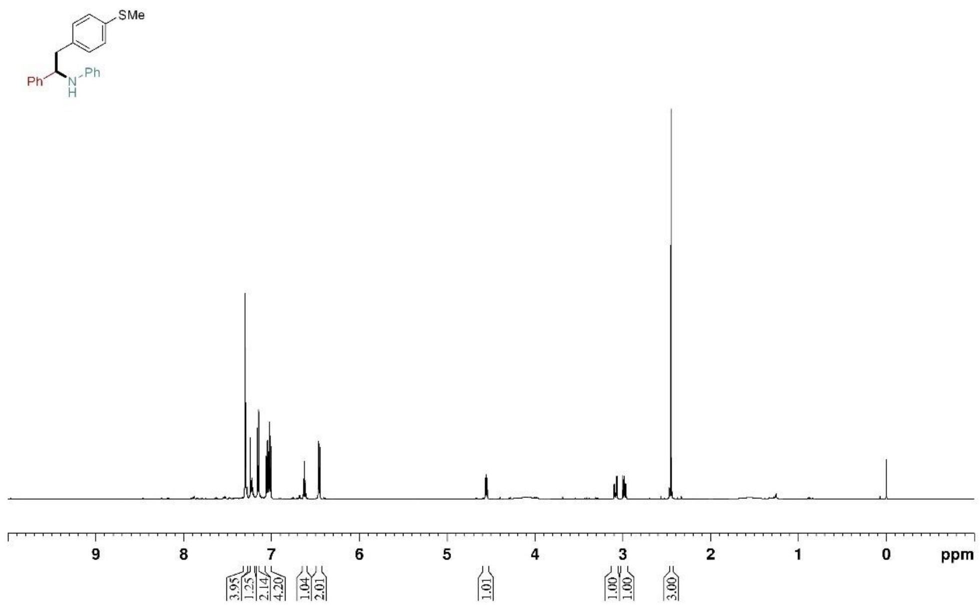

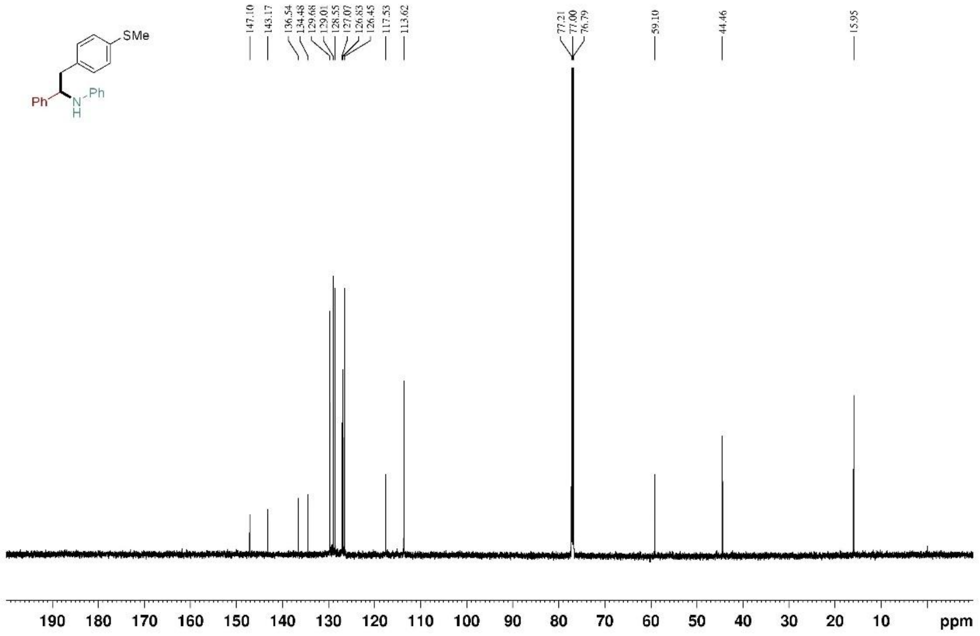

^{1}H NMR and ^{13}C NMR spectra of compound **5v**

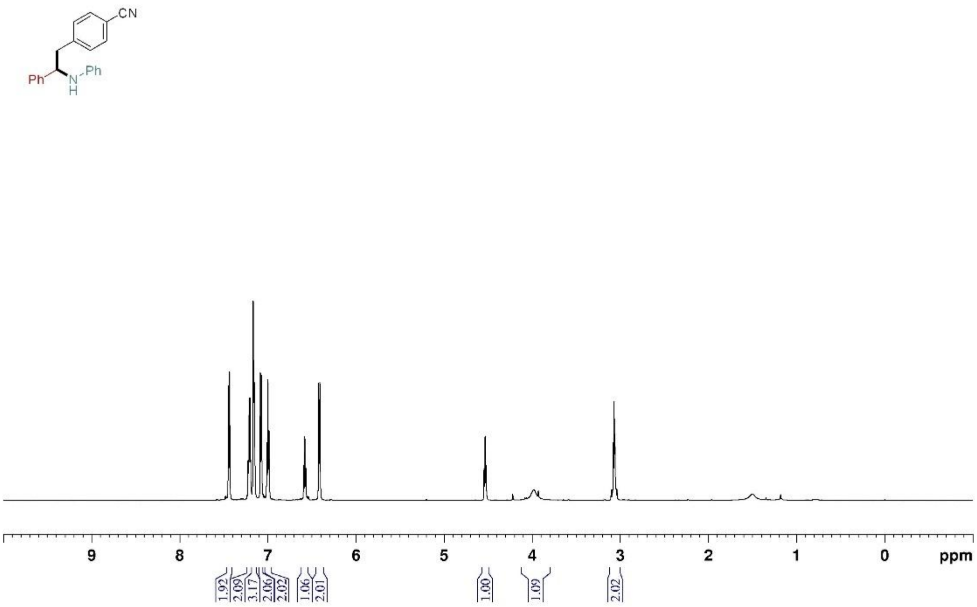

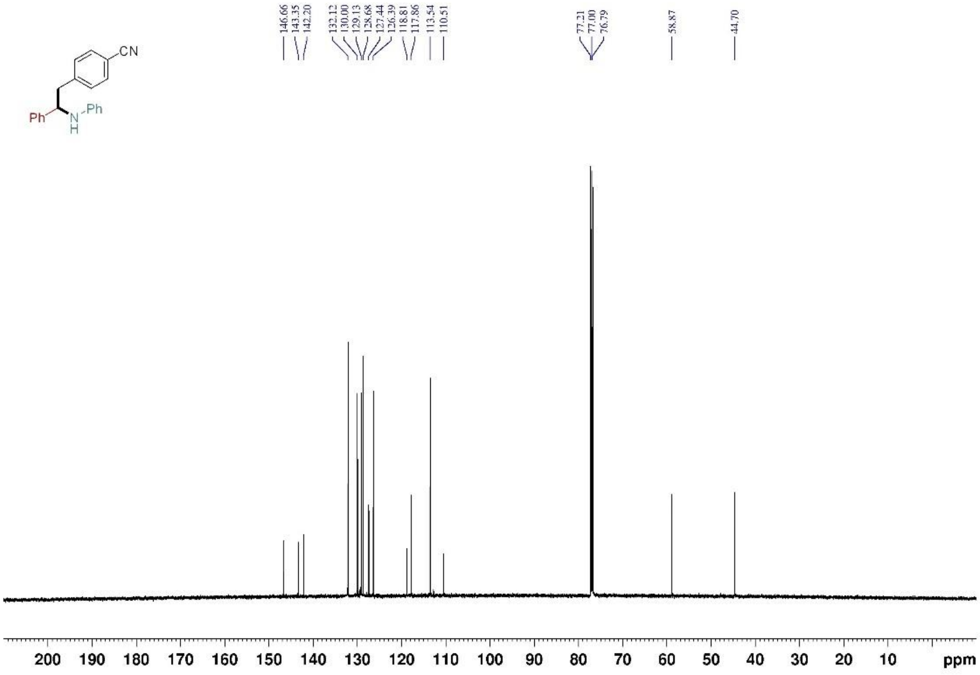

^{1}H NMR and ^{13}C NMR spectra of compound **5w**

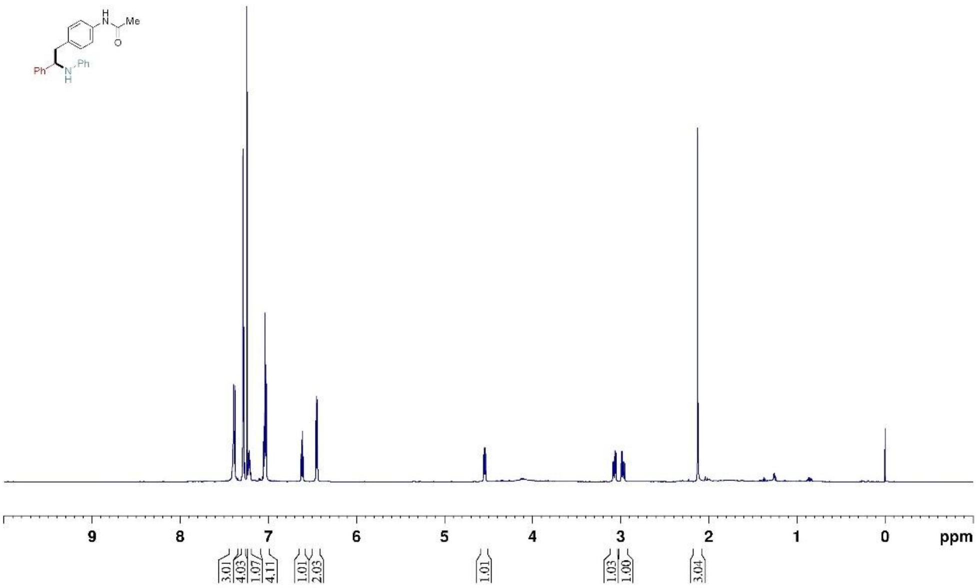

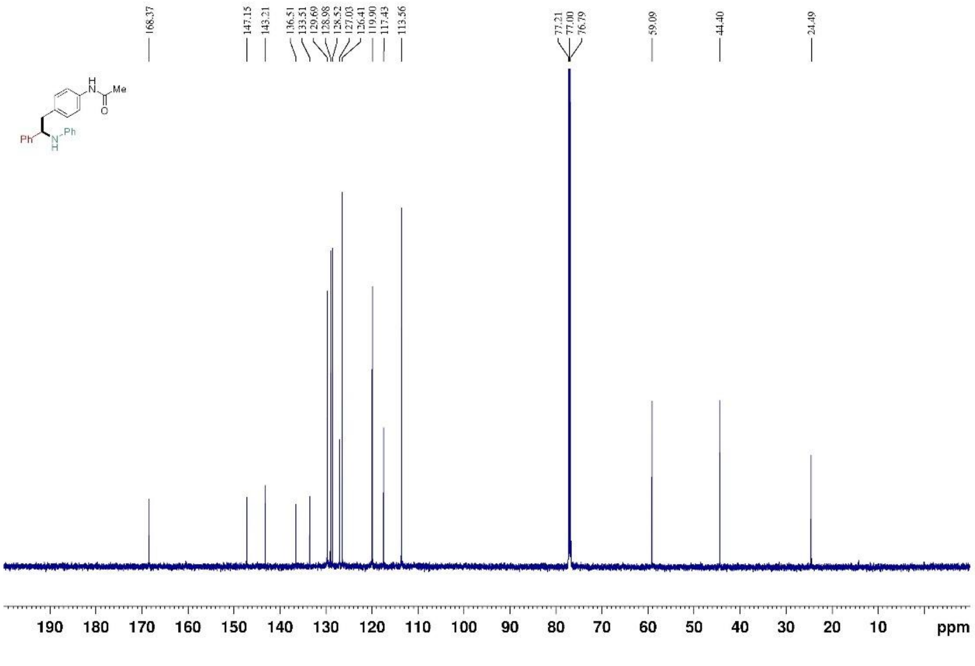

^{1}H NMR and ^{13}C NMR spectra of compound **5x**

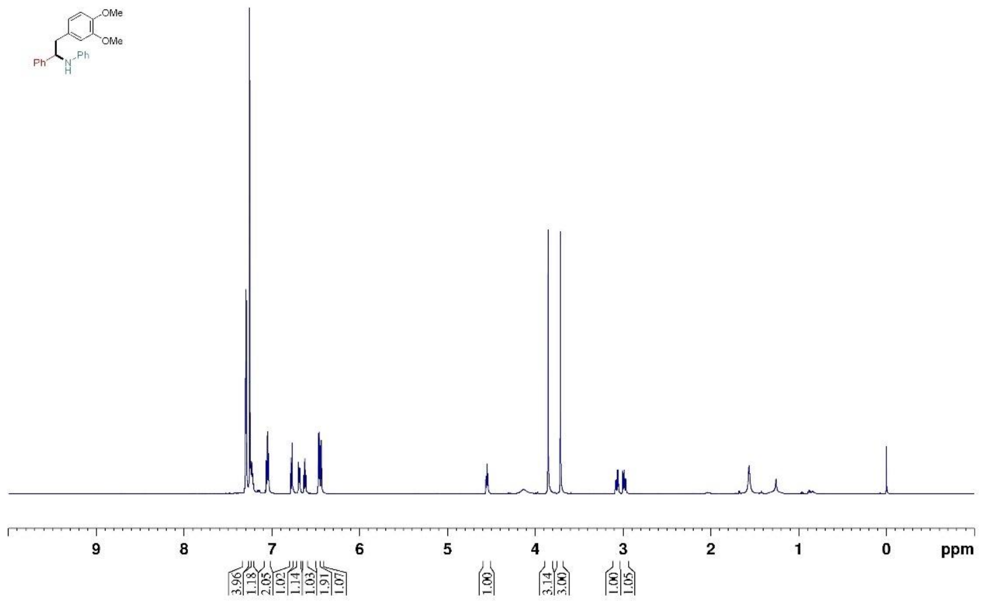

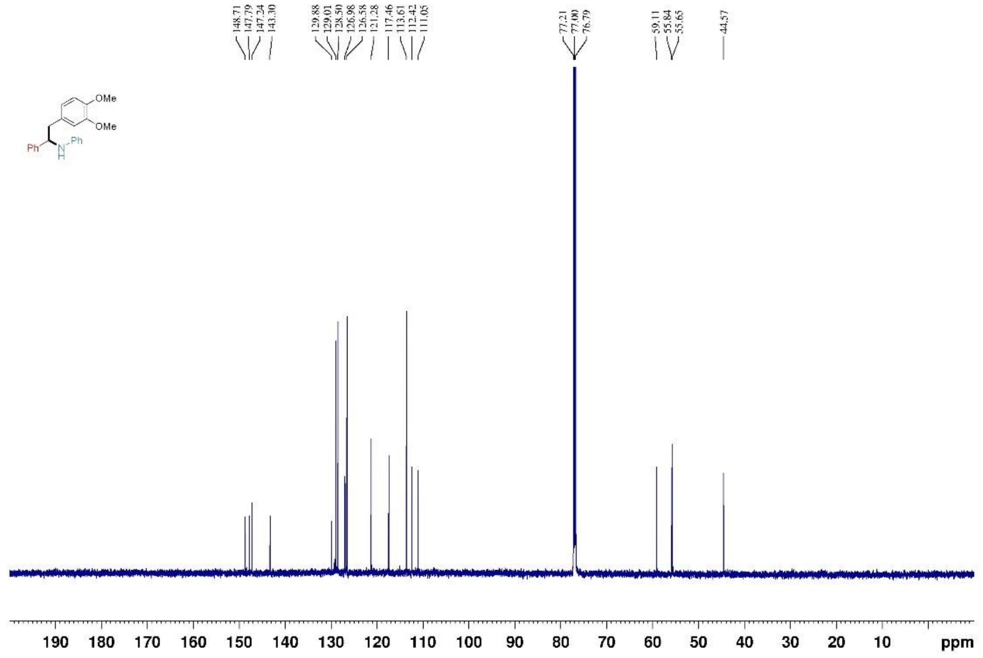

^{1}H NMR and ^{13}C NMR spectra of compound **5y**

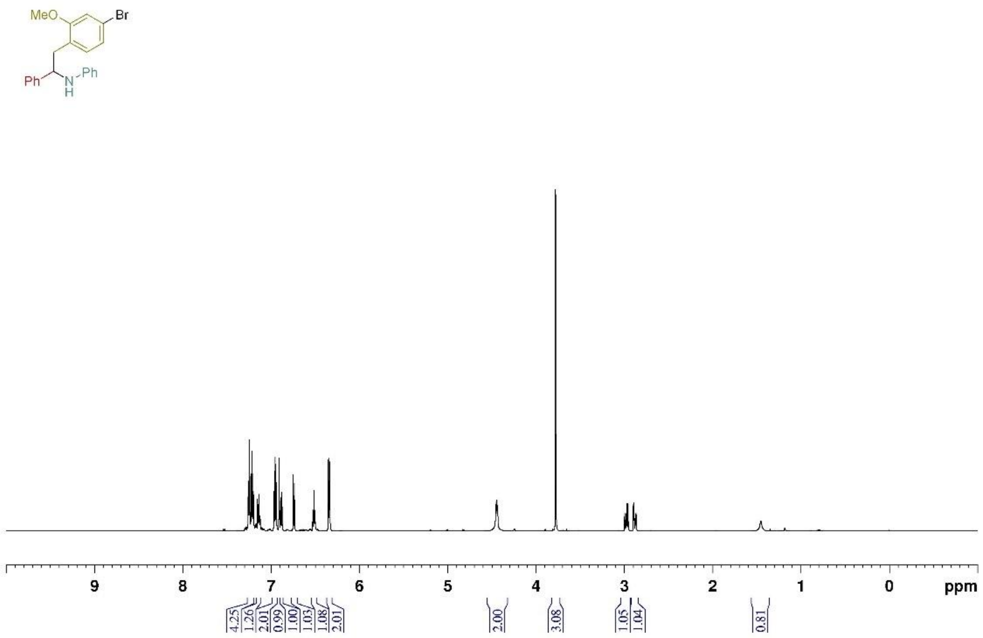

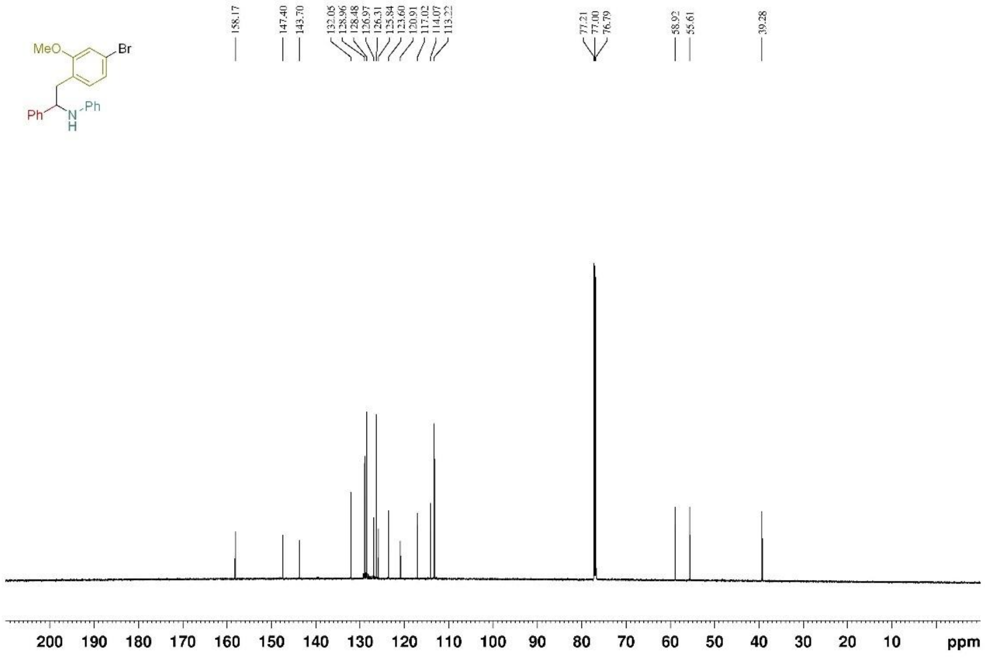

^{1}H NMR and ^{13}C NMR spectra of compound **5z**

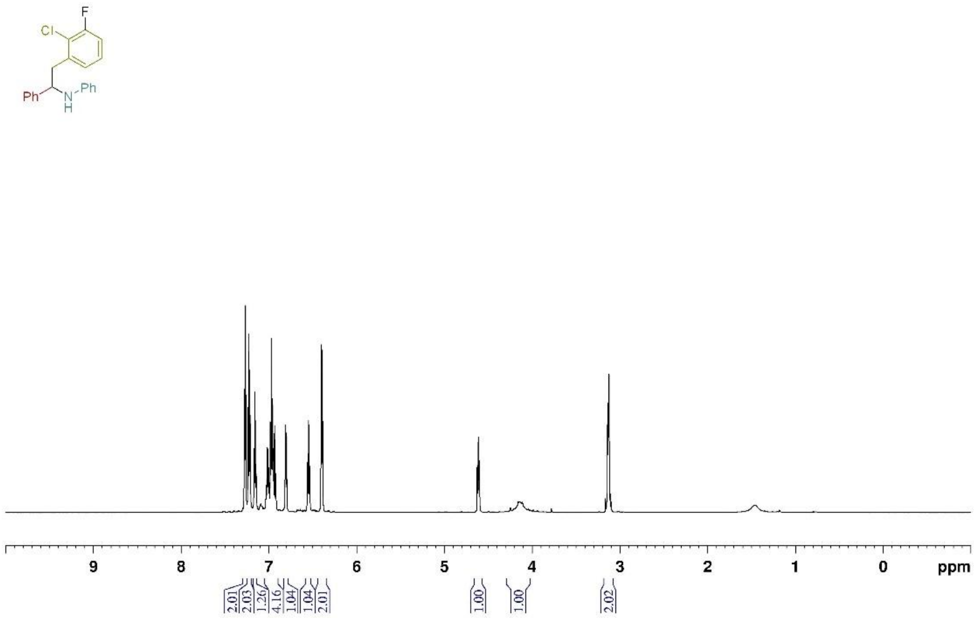

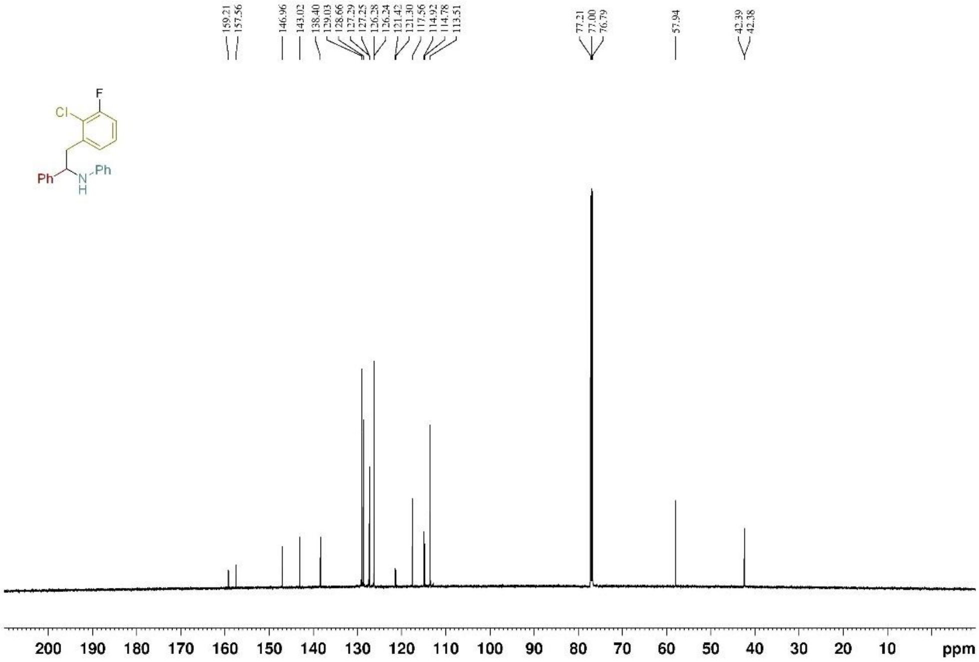

^{1}H NMR and ^{13}C NMR spectra of compound **5aa**

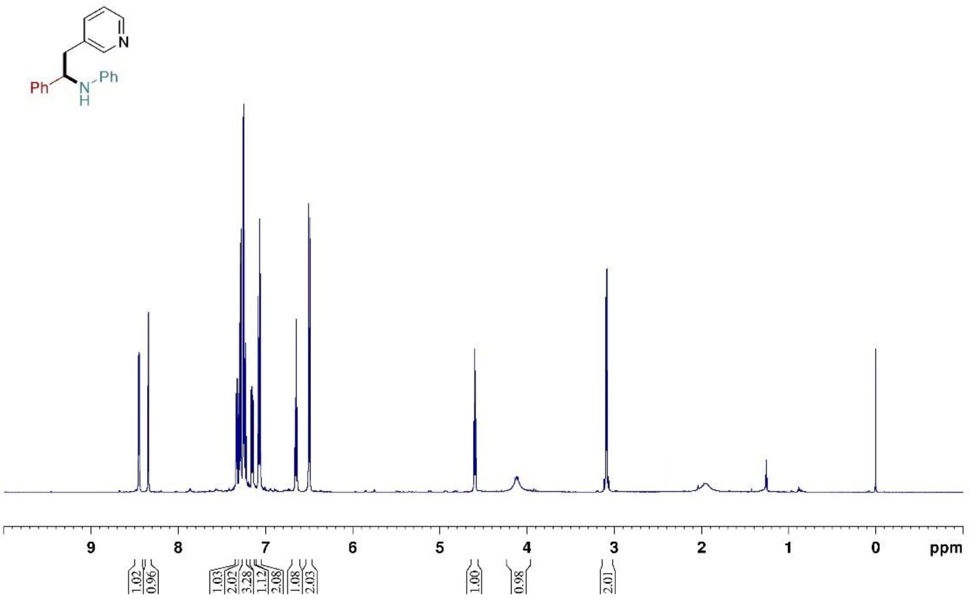

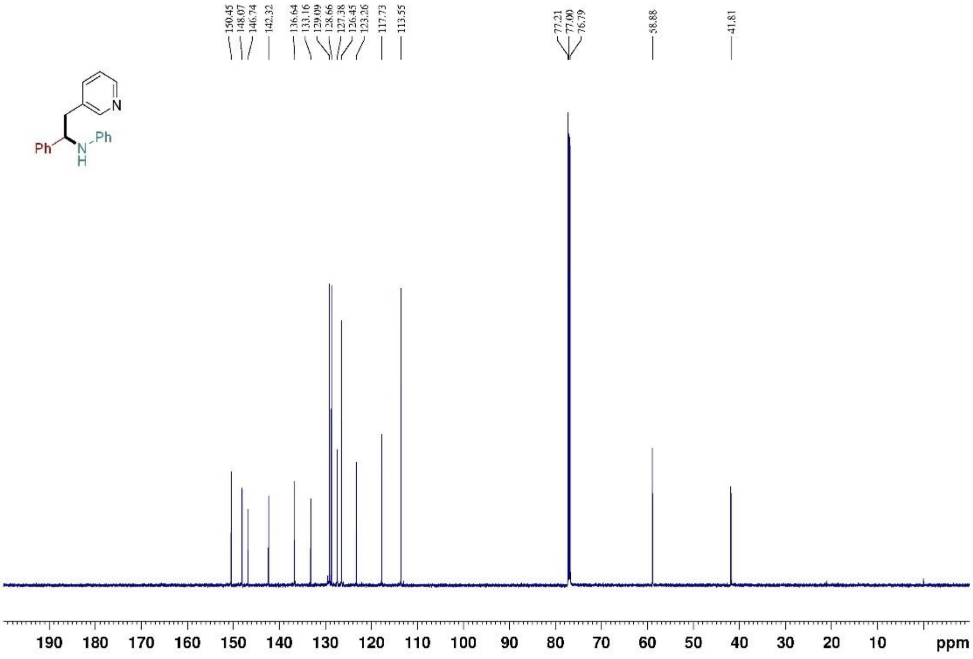

^{1}H NMR and ^{13}C NMR spectra of compound **5ab**

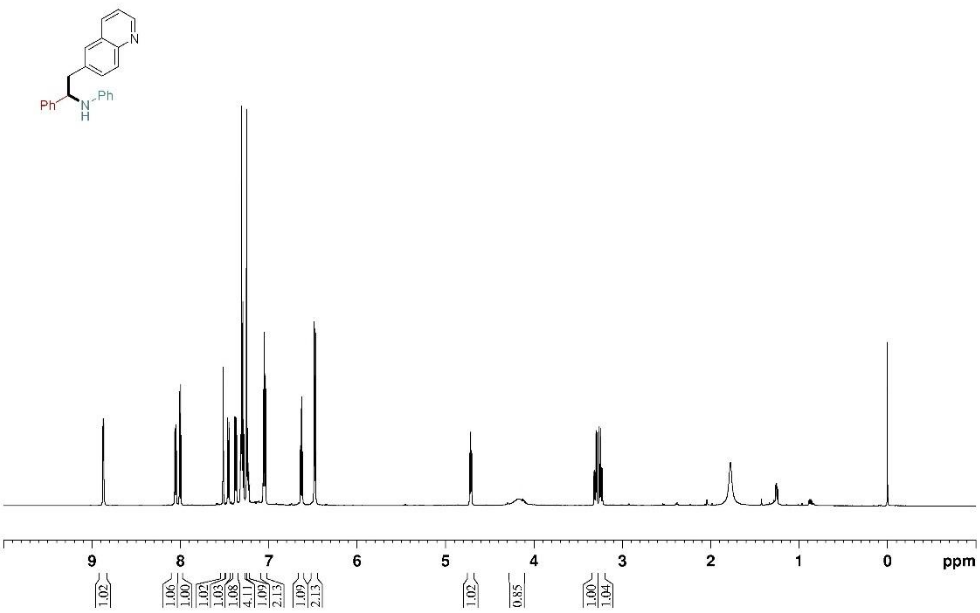

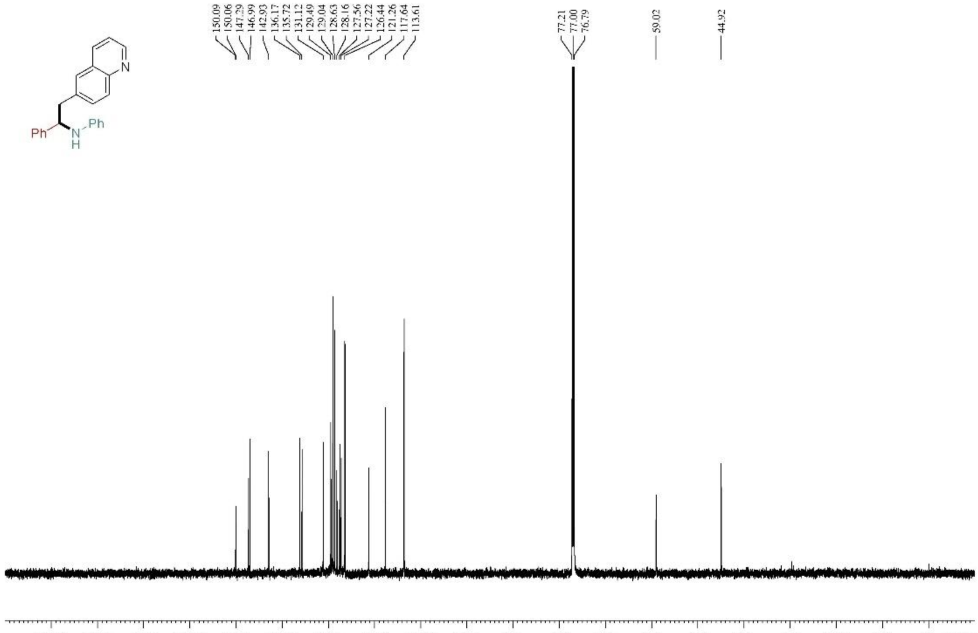

^{1}H NMR and ^{13}C NMR spectra of compound **5ac**

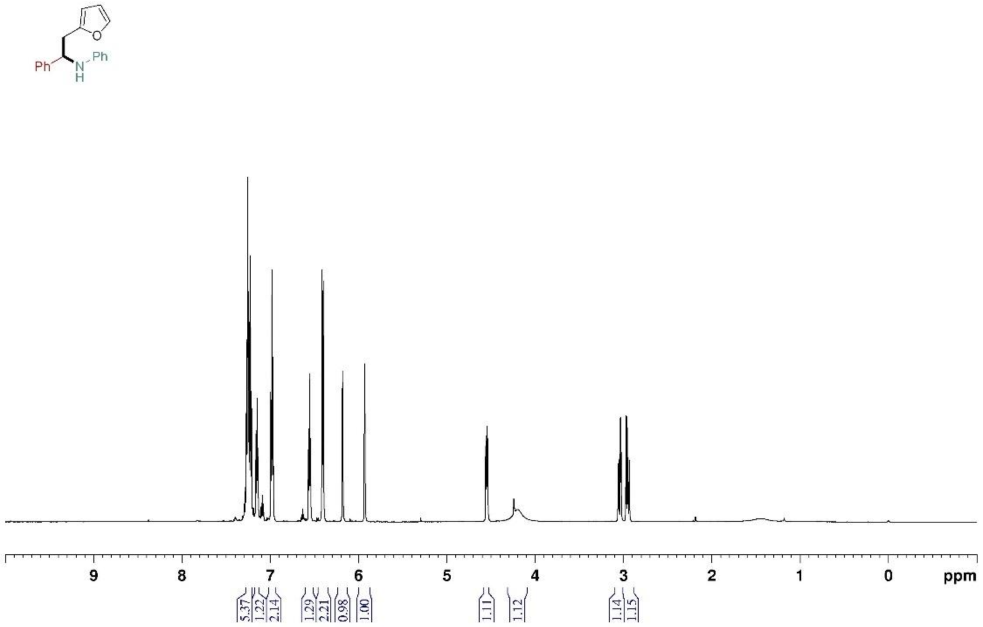

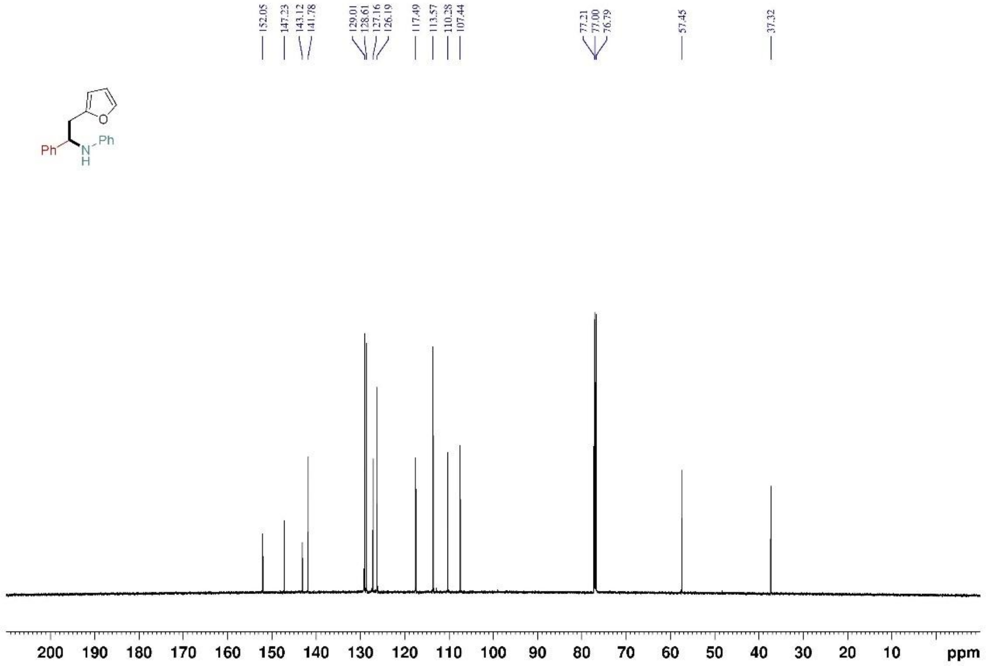

^{1}H NMR and ^{13}C NMR spectra of compound **5ad**

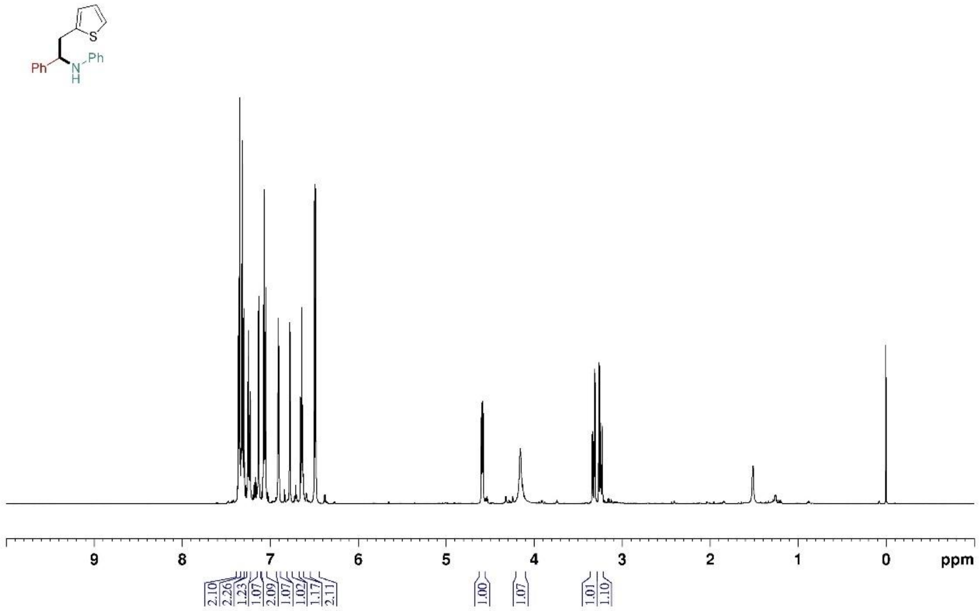

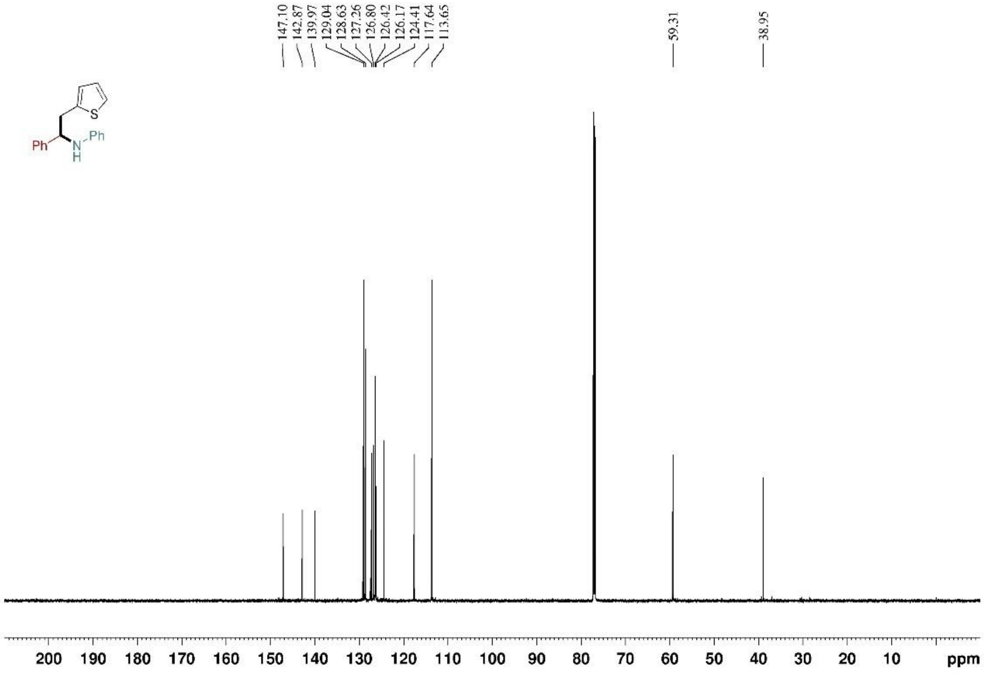

^{1}H NMR and ^{13}C NMR spectra of compound **5ae**

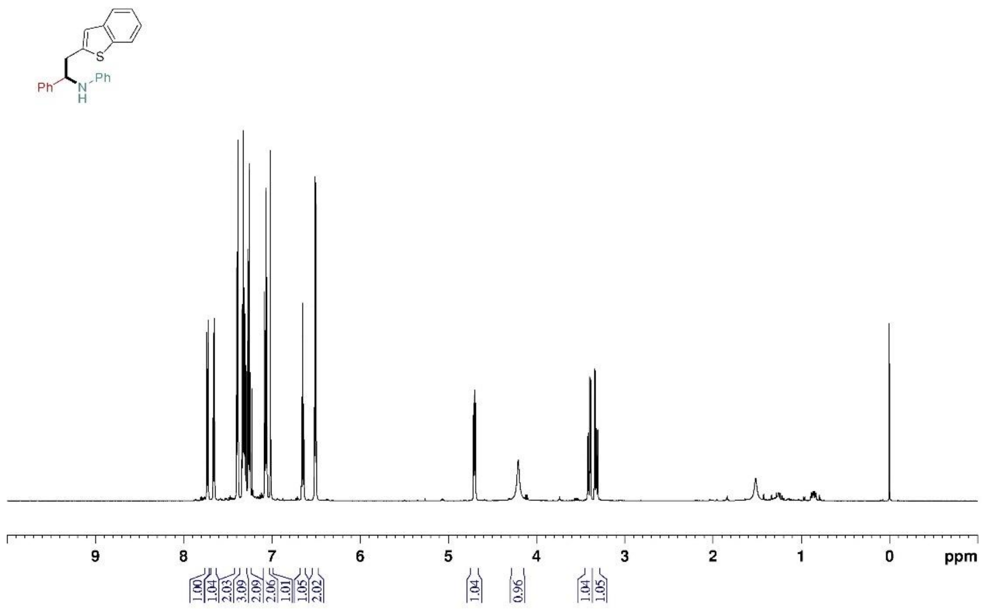

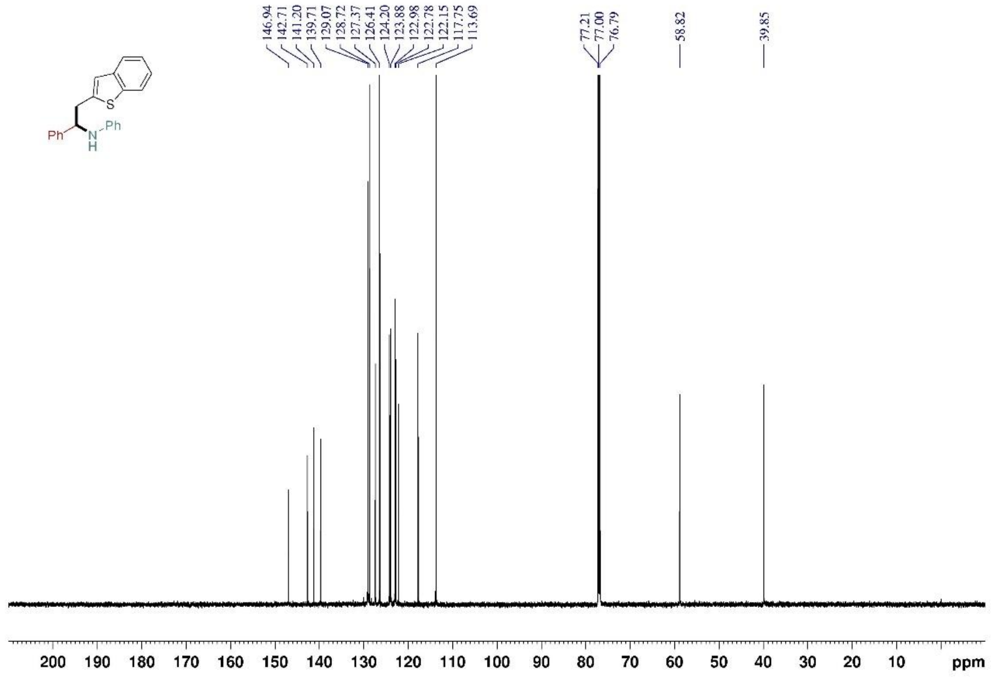

^{1}H NMR and ^{13}C NMR spectra of compound **5af**

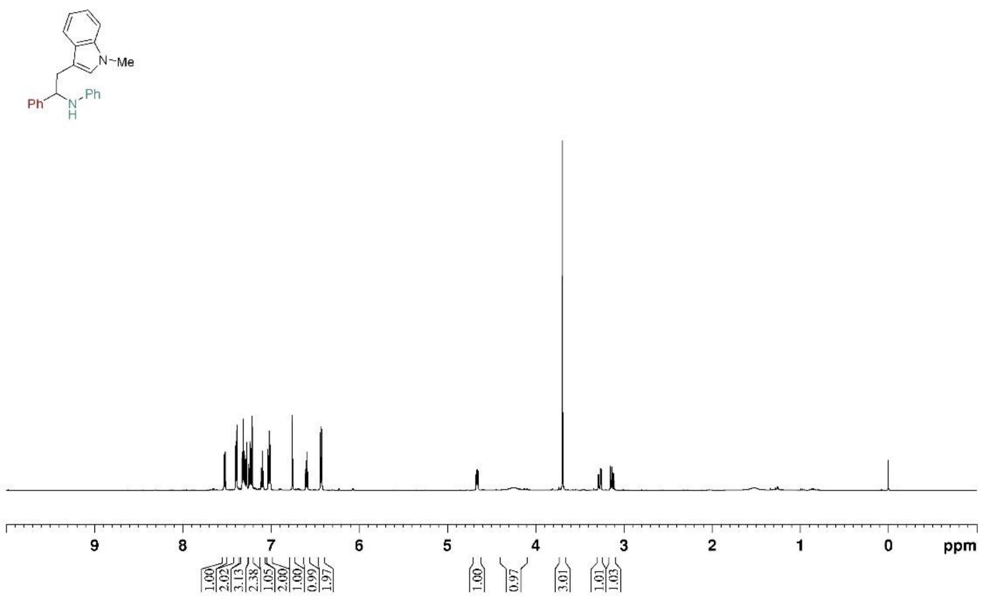

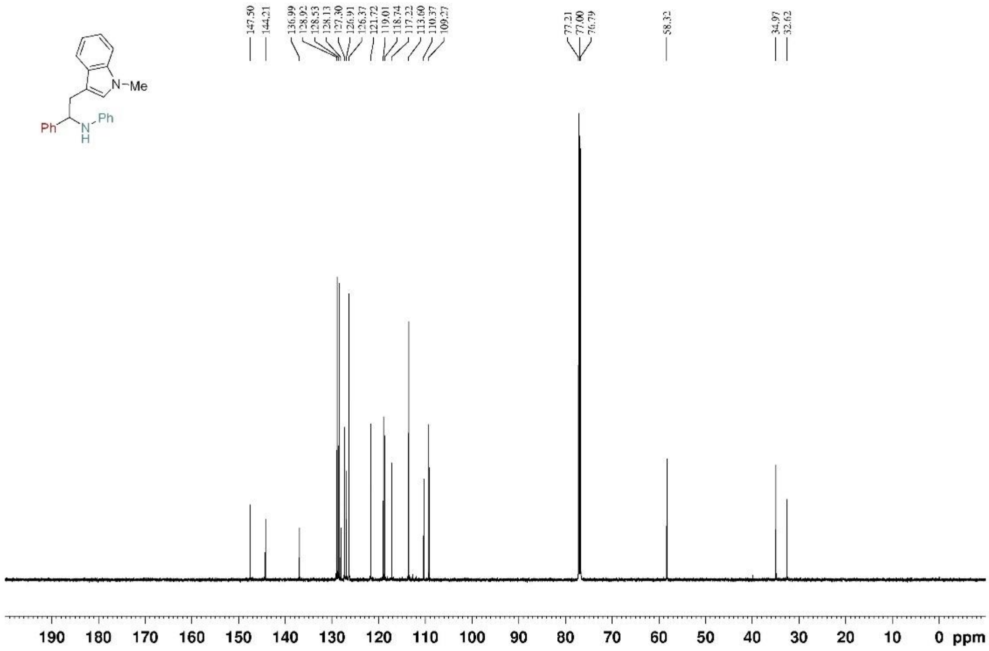

^{1}H NMR and ^{13}C NMR spectra of compound **5ag-A**

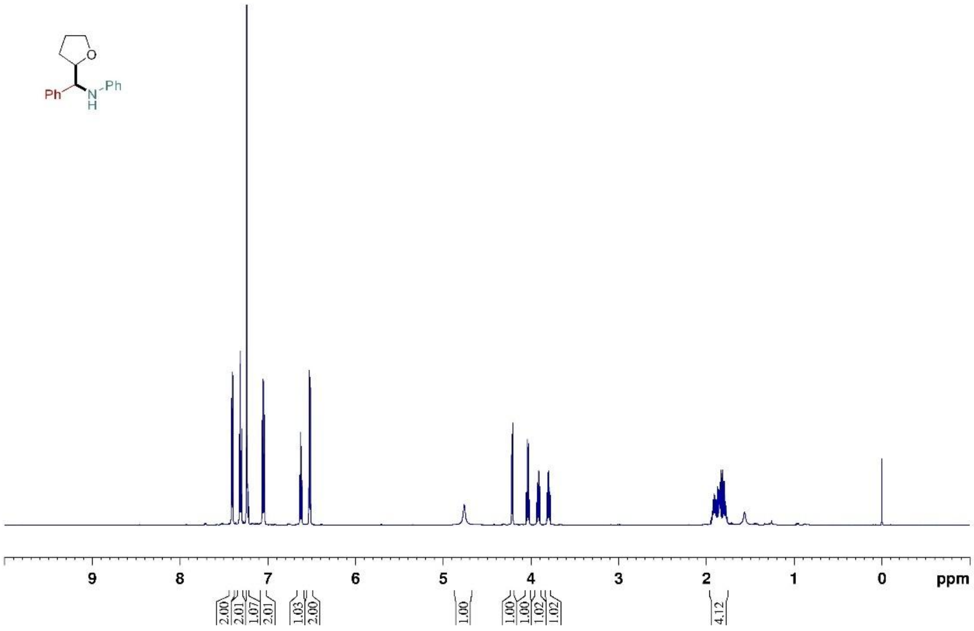

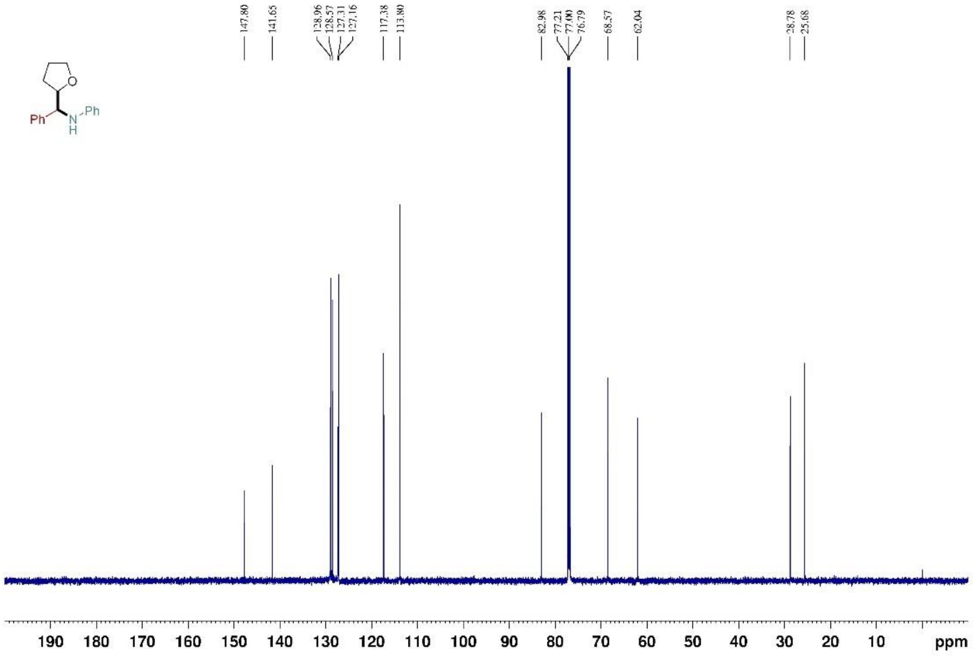

^{1}H NMR and ^{13}C NMR spectra of compound **5ag-B**

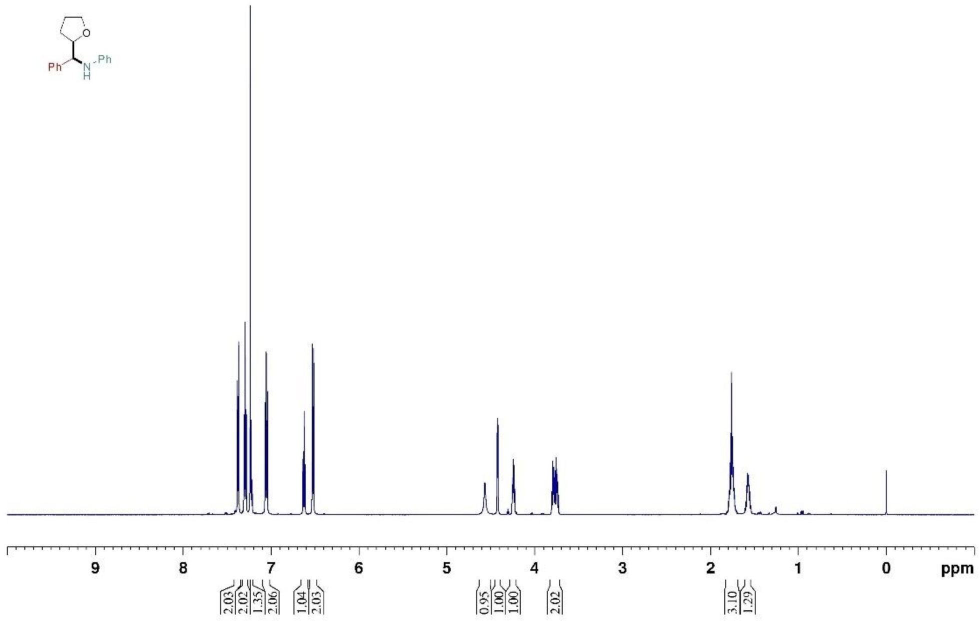

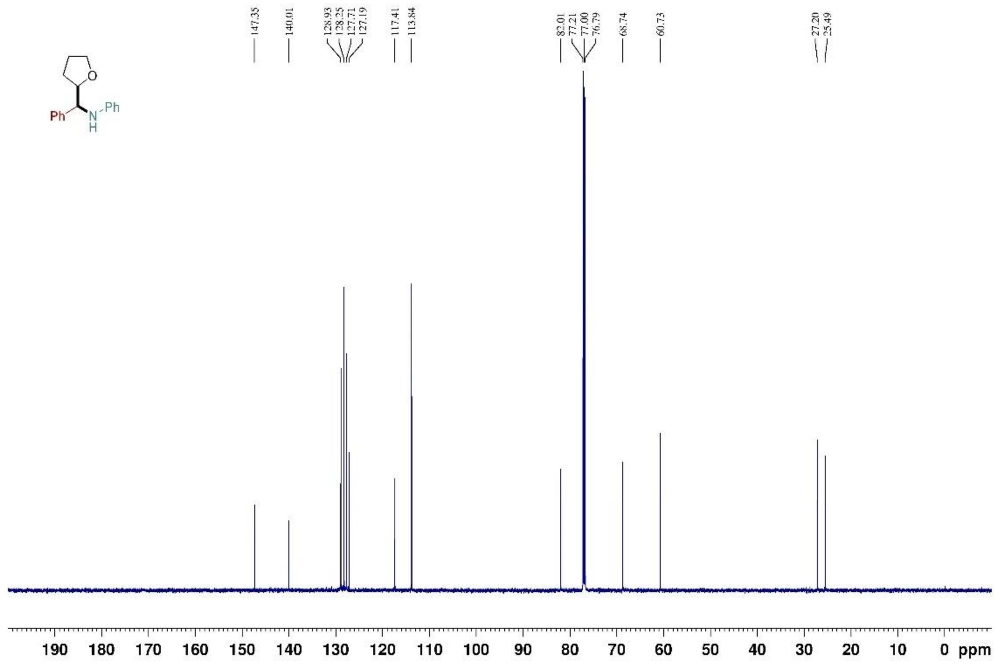

^{1}H NMR and ^{13}C NMR spectra of compound **5ah**

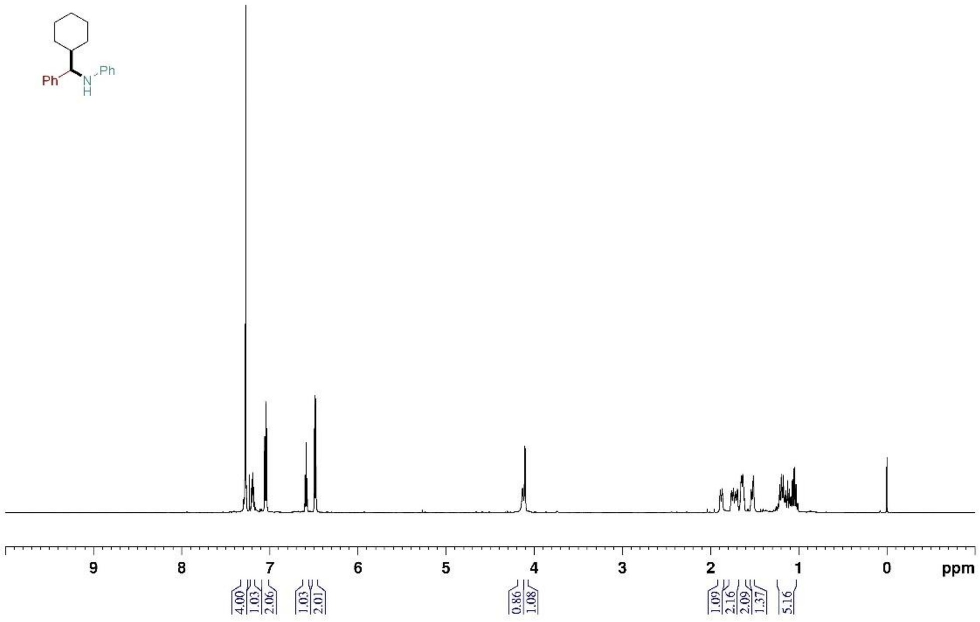

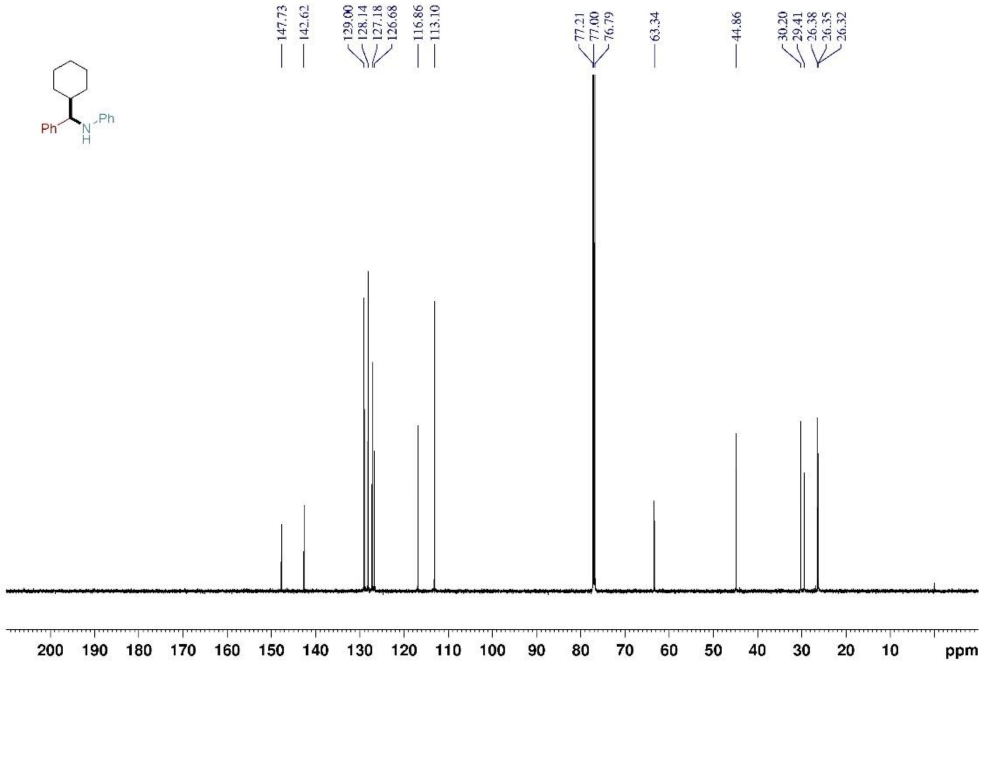

^{1}H NMR and ^{13}C NMR spectra of compound **5ai**

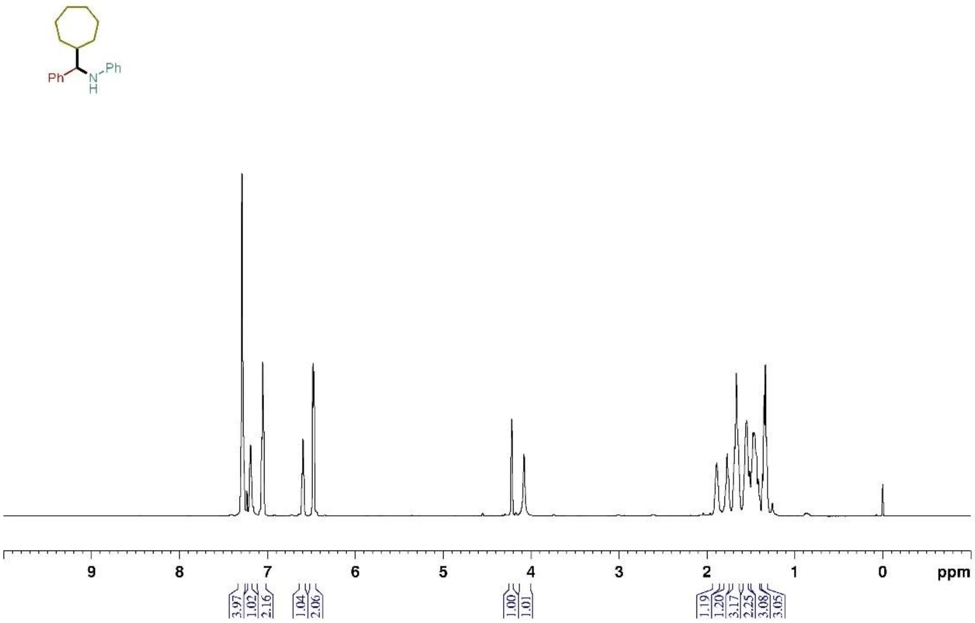

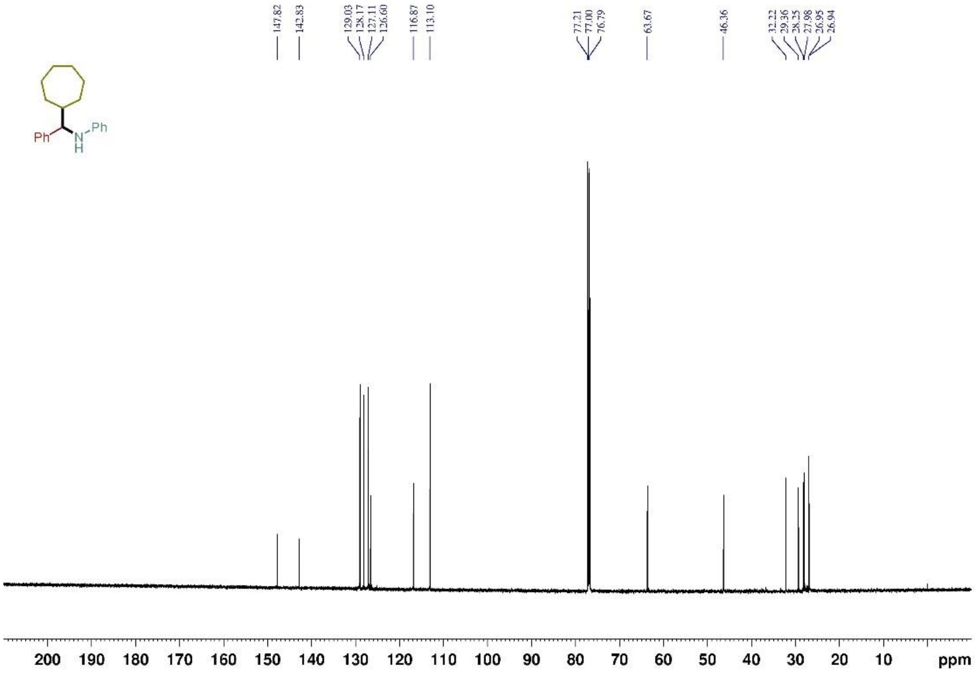

^1H NMR and ^{13}C NMR spectra of compound **5aj**

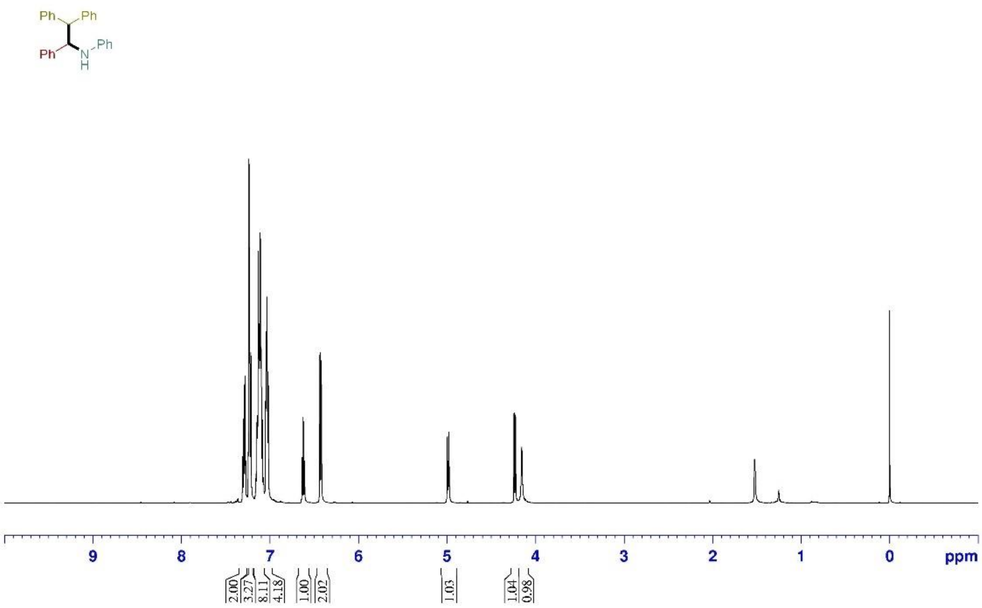

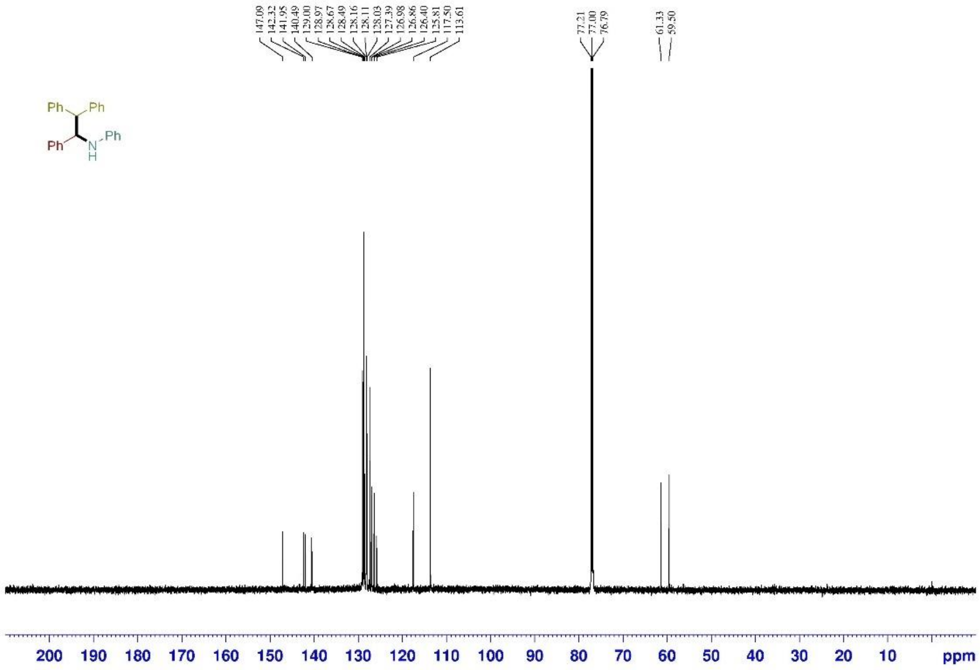

^{1}H NMR and ^{13}C NMR spectra of compound **5ak**

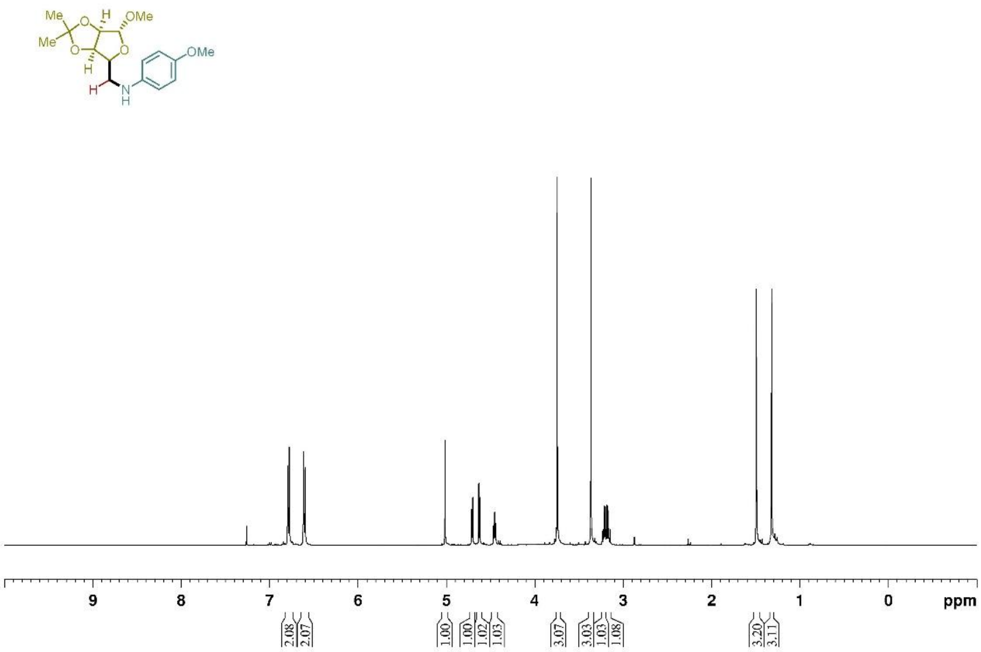

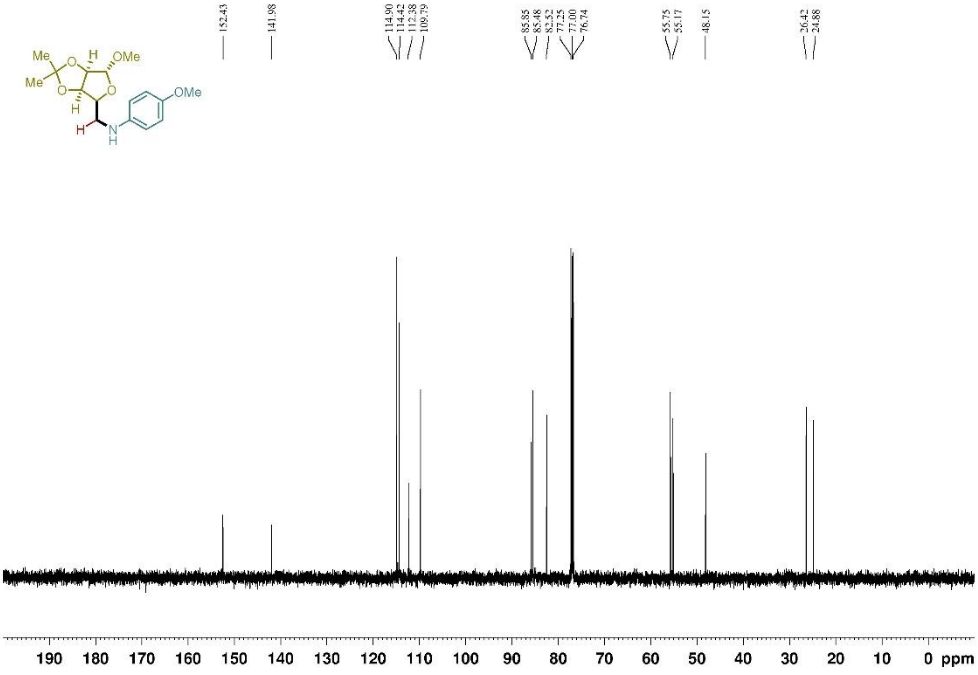

^{1}H NMR and ^{13}C NMR spectra of compound **5al**

^{1}H NMR and ^{13}C NMR spectra of compound **5al**

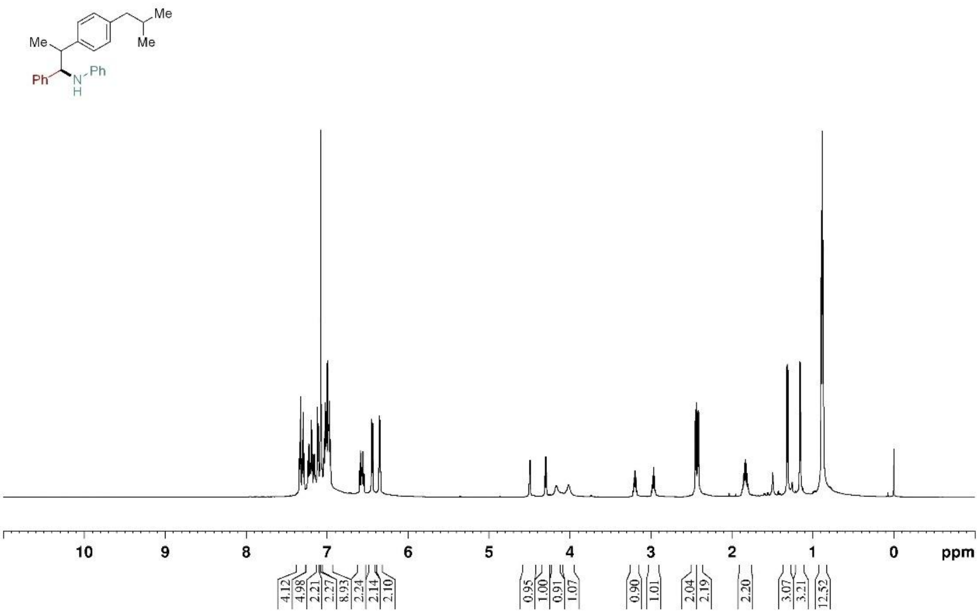

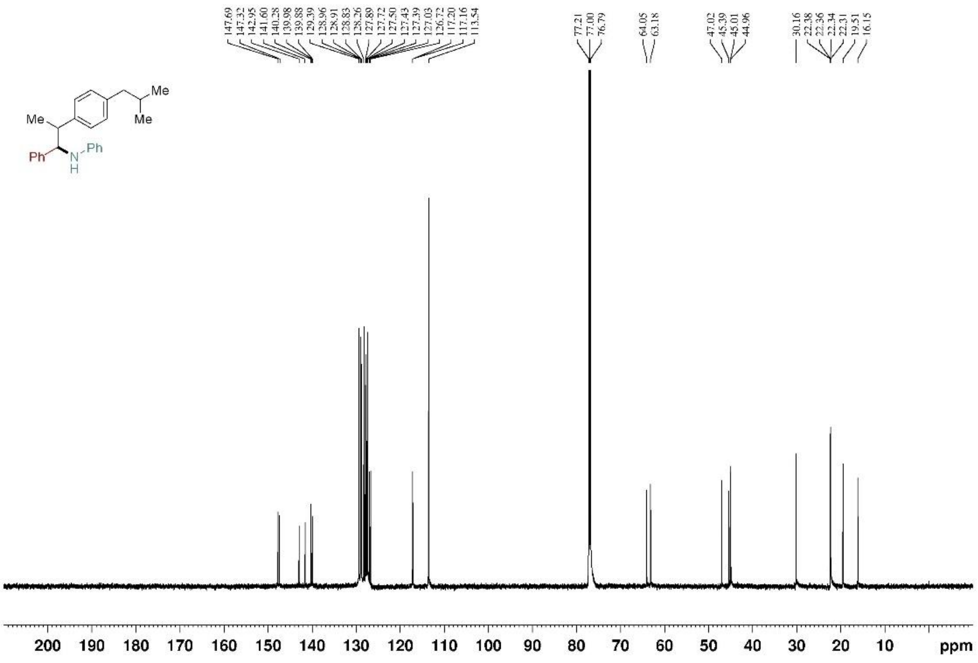

^{1}H NMR and ^{13}C NMR spectra of compound **5am**

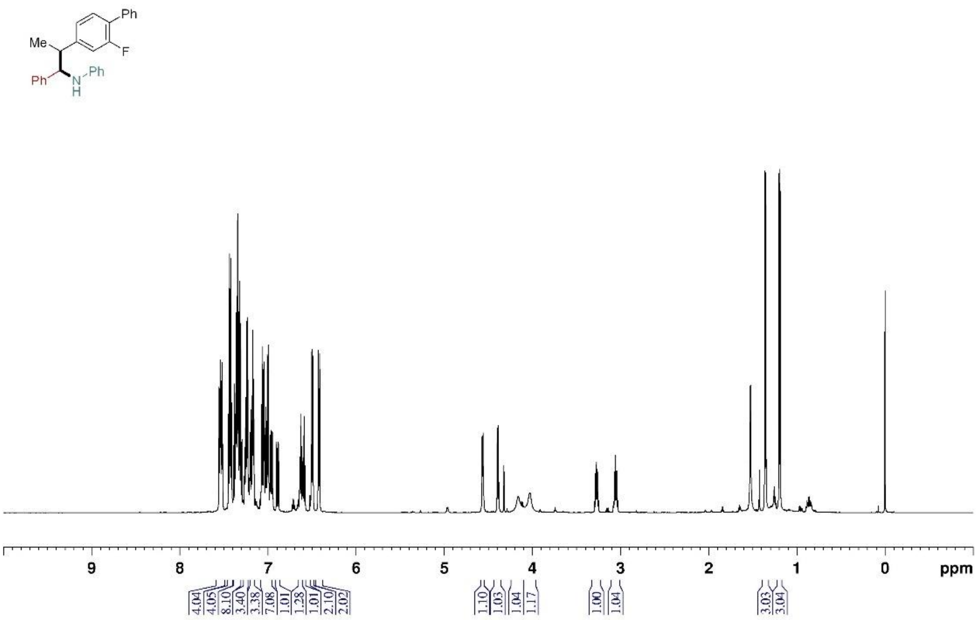

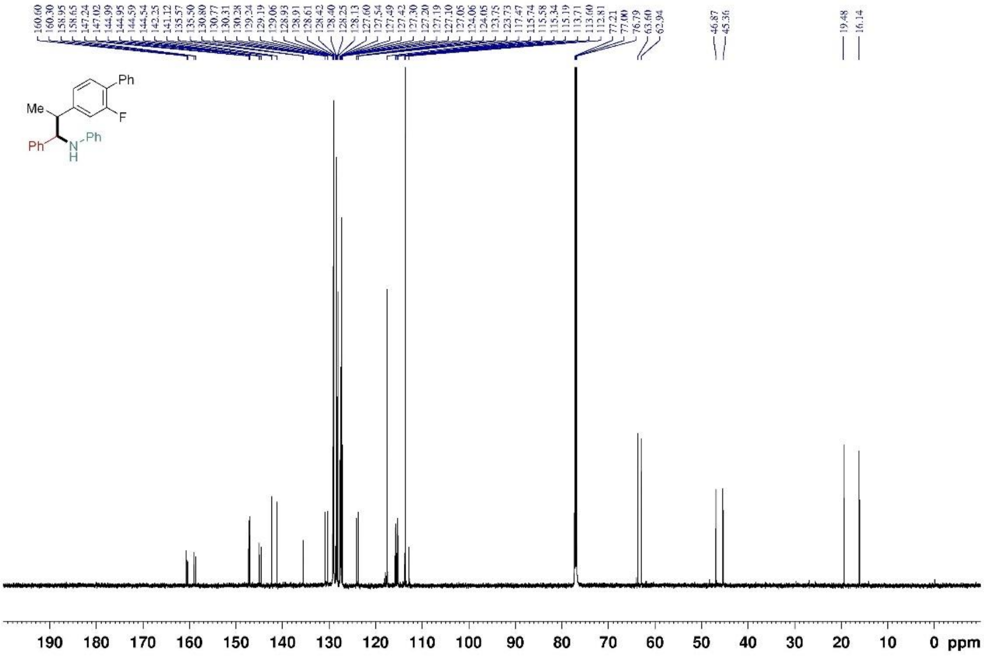

^{1}H NMR and ^{13}C NMR spectra of compound **5an**

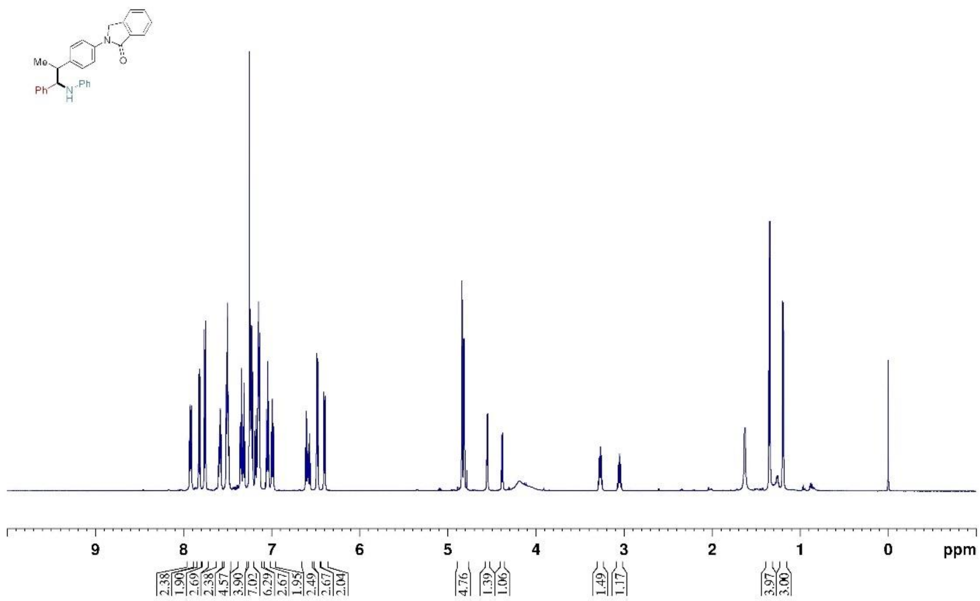

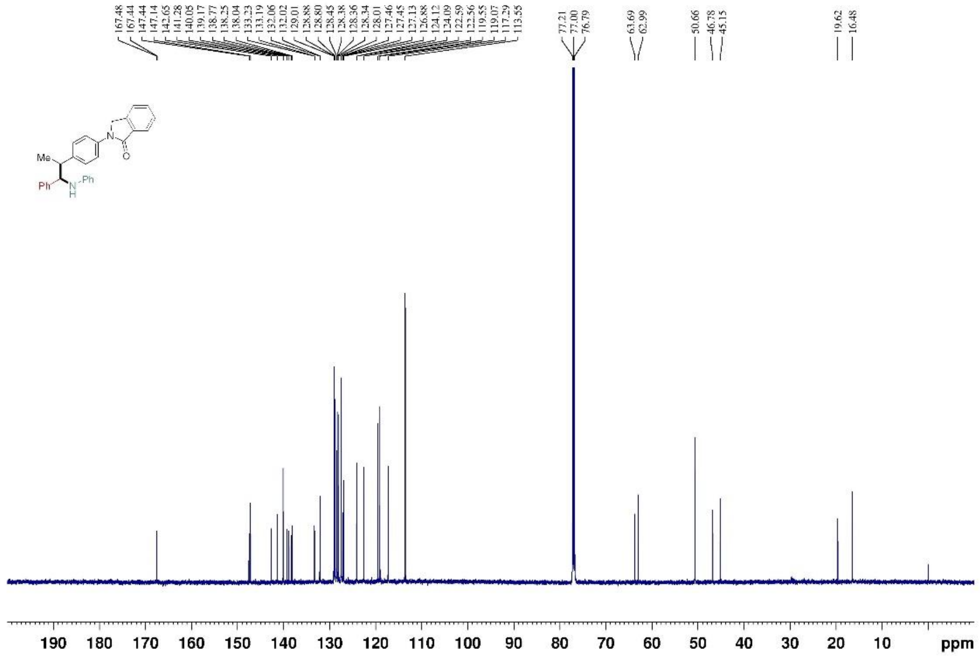

^{1}H NMR and ^{13}C NMR spectra of compound **5ao**

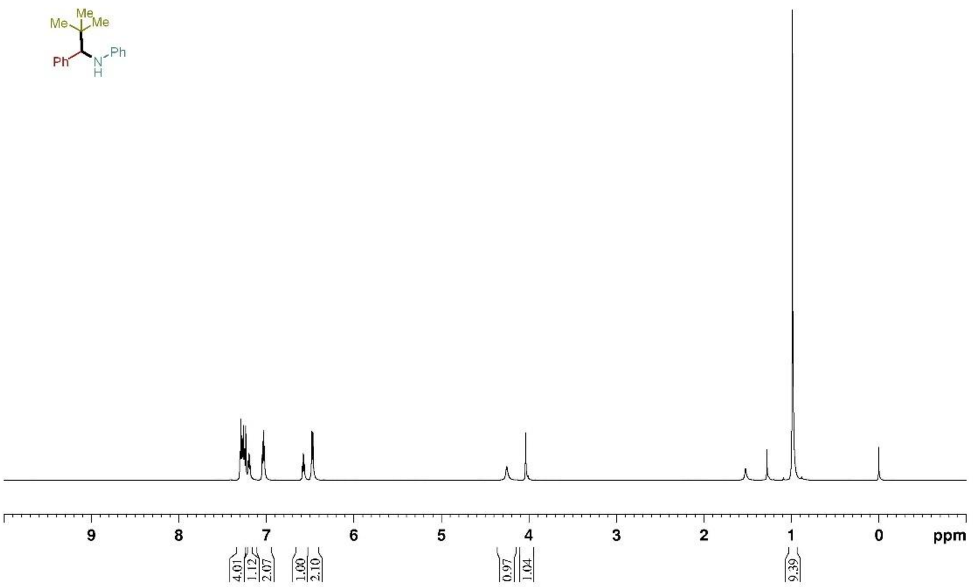

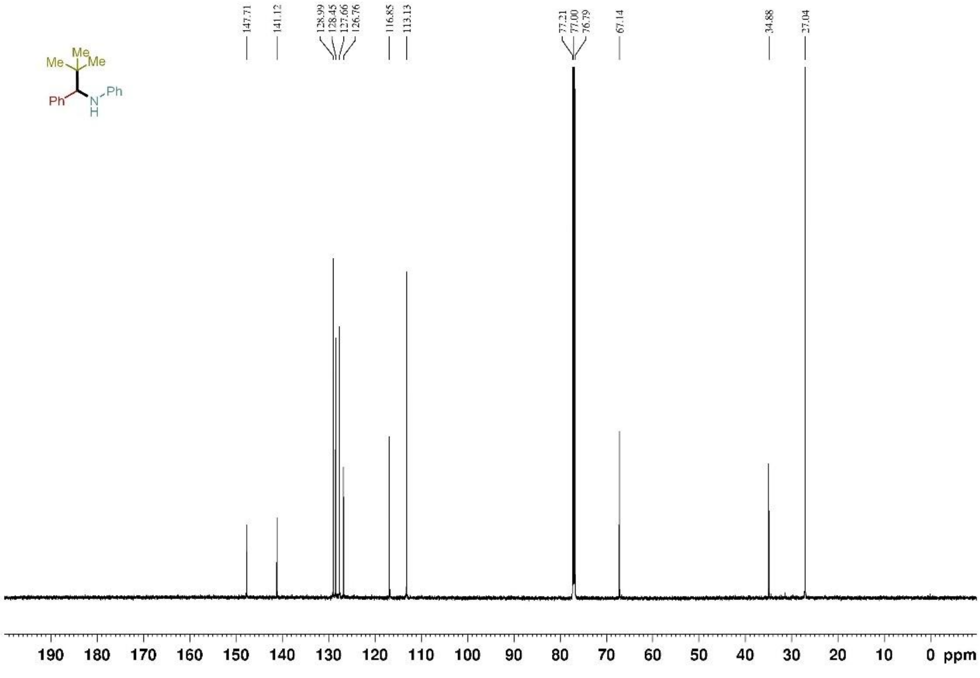

^{1}H NMR and ^{13}C NMR spectra of compound **5ap**

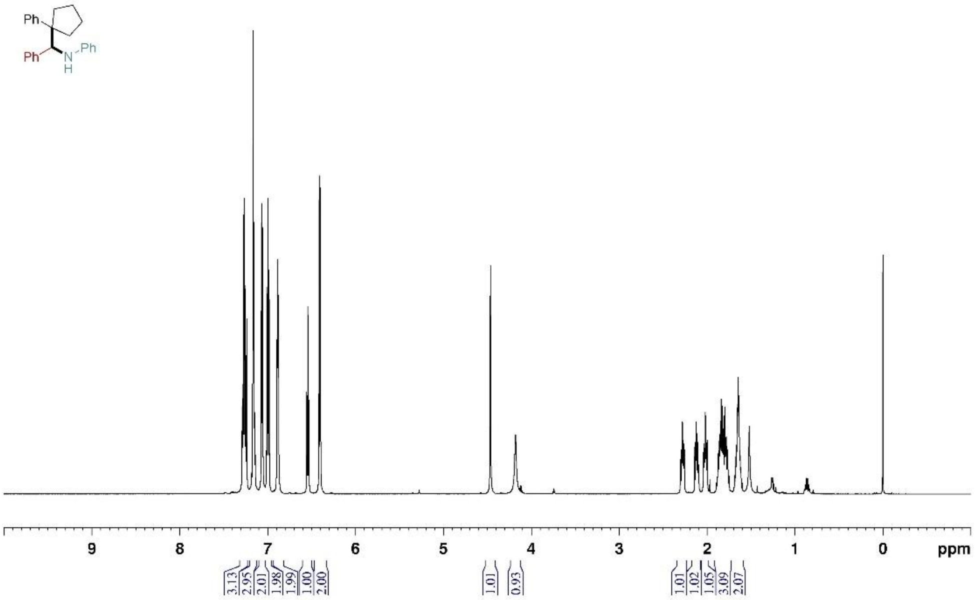

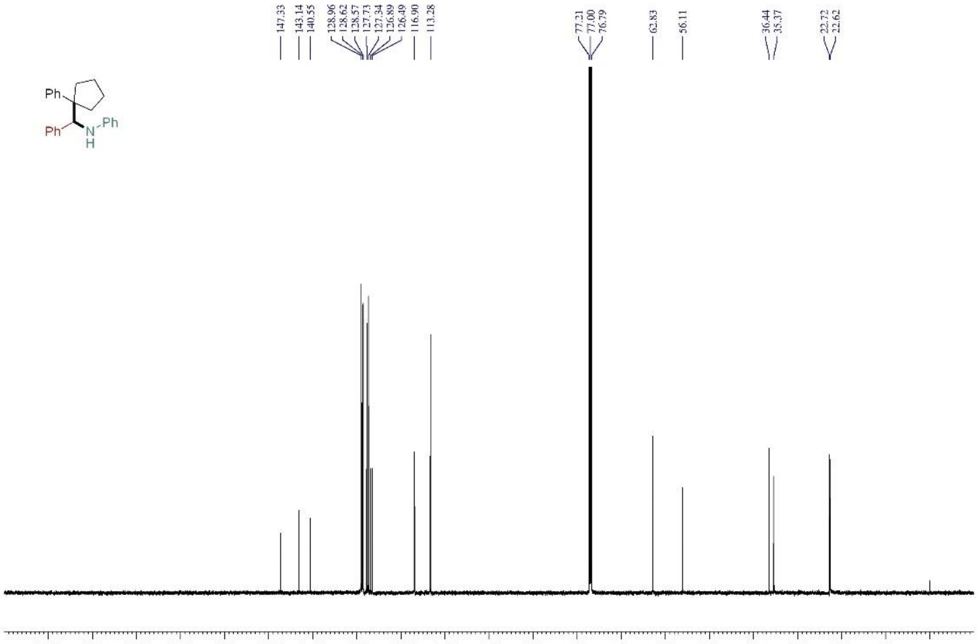

^{1}H NMR and ^{13}C NMR spectra of compound **5aq**

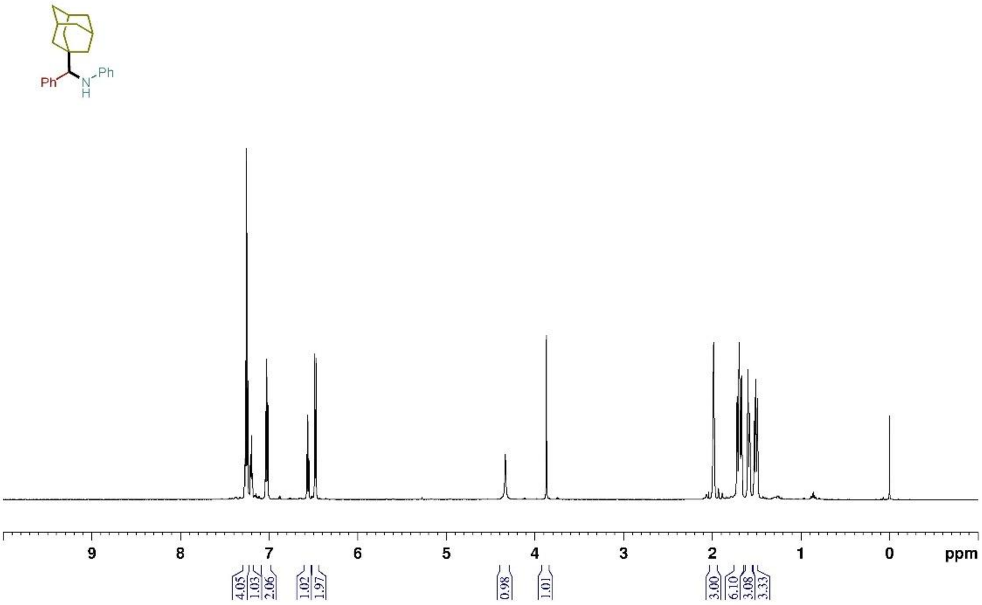

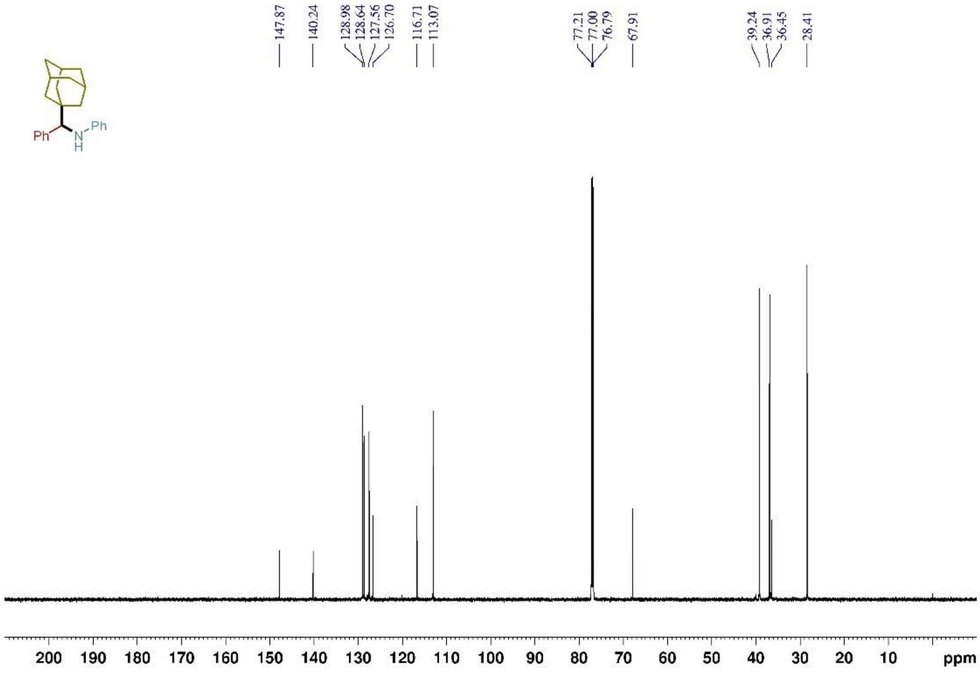

^{1}H NMR and ^{13}C NMR spectra of compound **5ar**

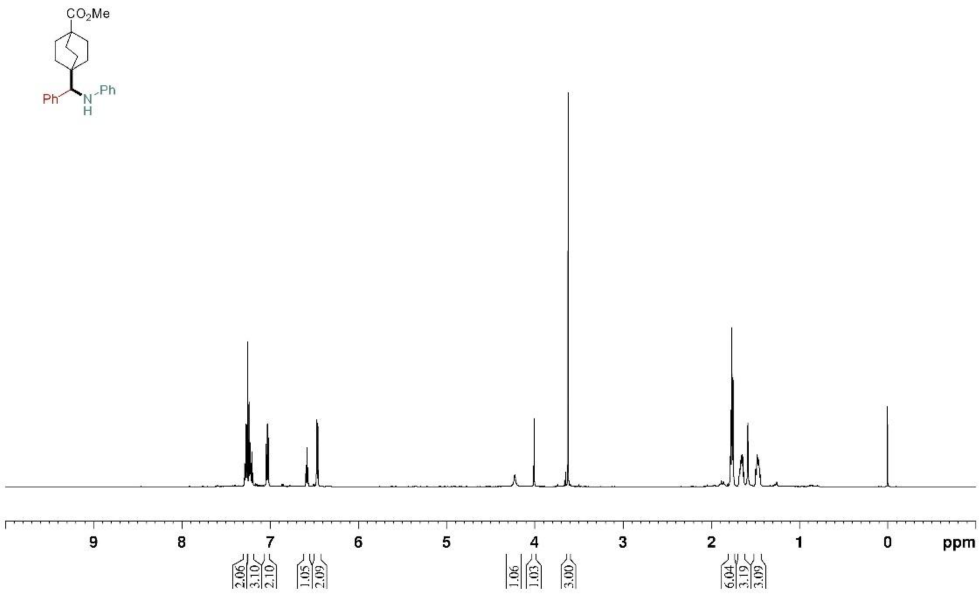

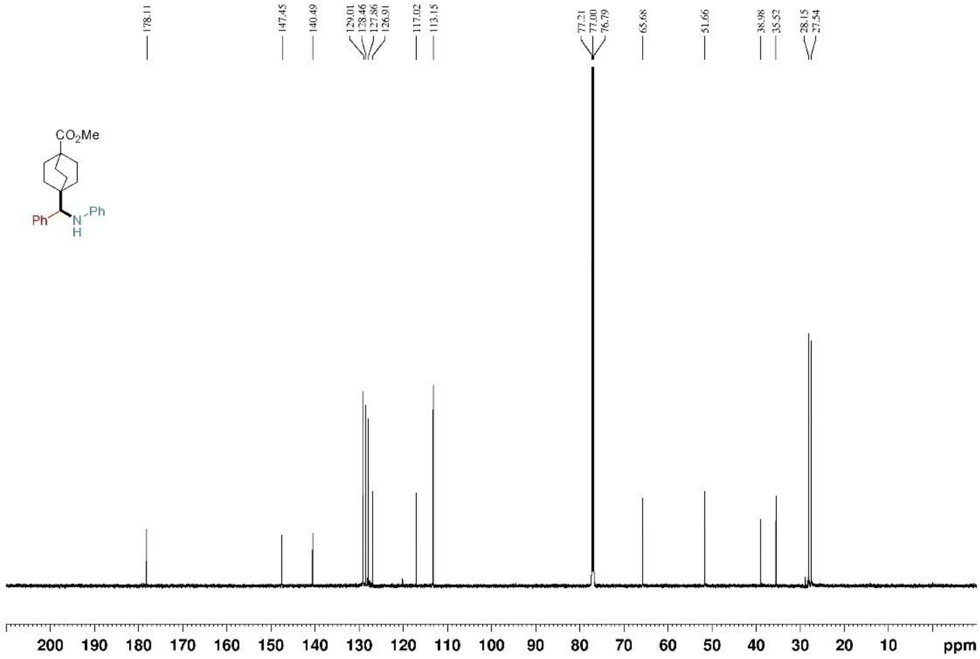

^{1}H NMR and ^{13}C NMR spectra of compound **5as**

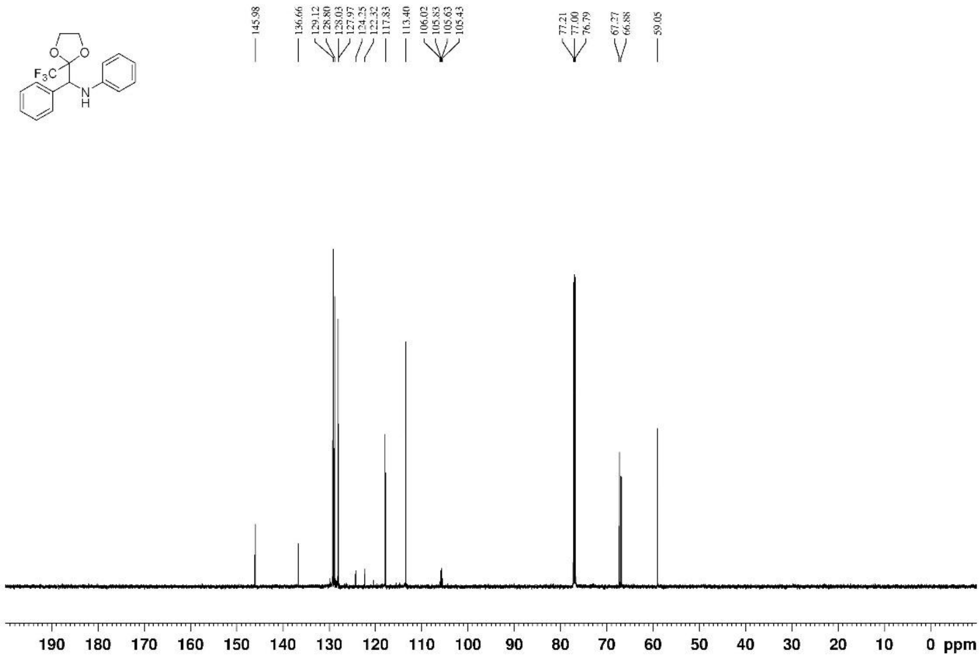

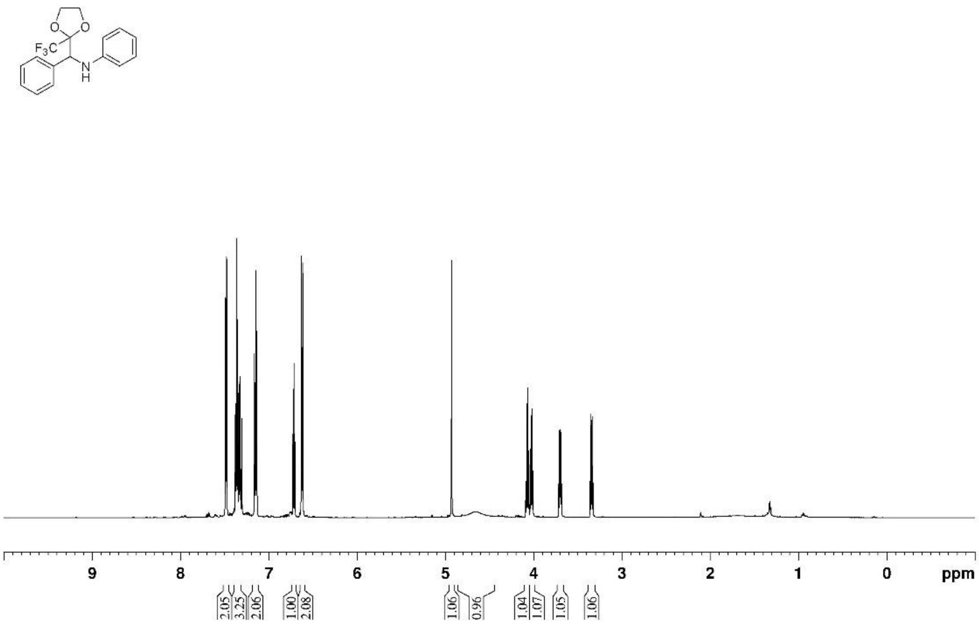

^{1}H NMR and ^{13}C NMR spectra of compound **5at**

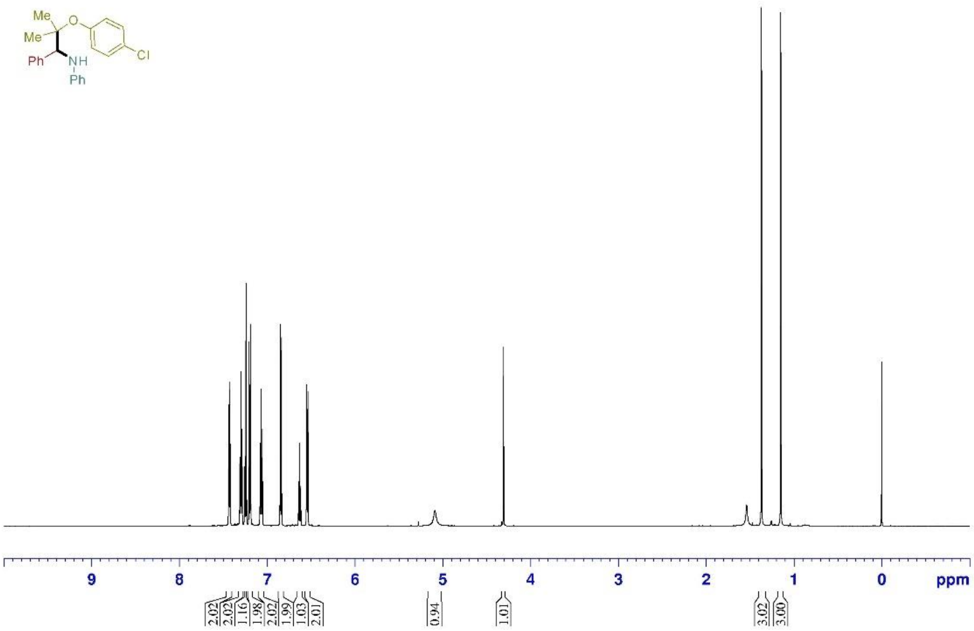

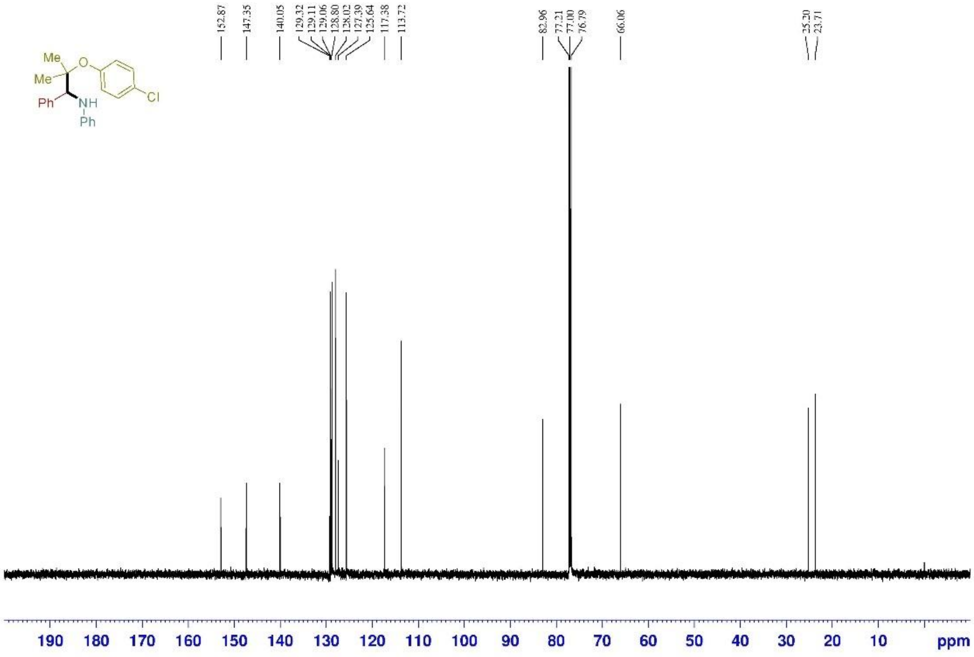

^{1}H NMR and ^{13}C NMR spectra of compound **5au**

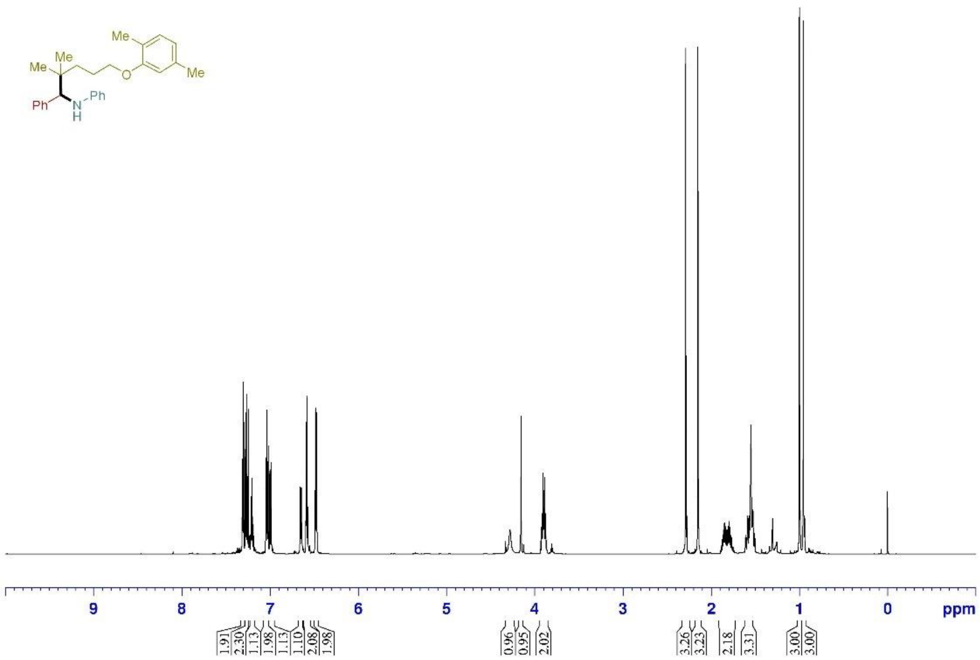

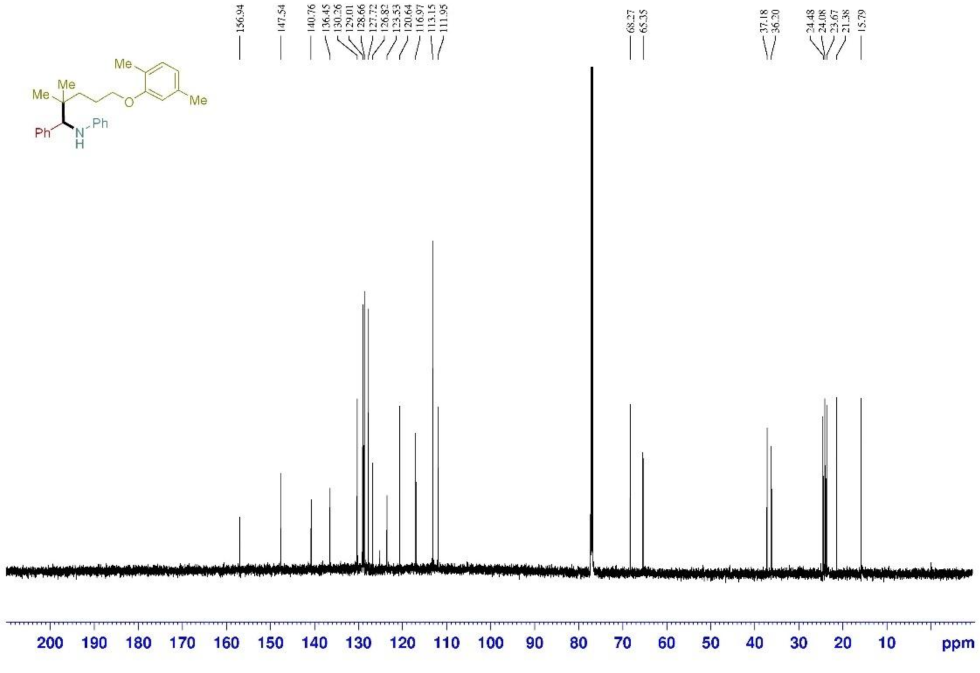

^{1}H NMR and ^{13}C NMR spectra of compound **5av**

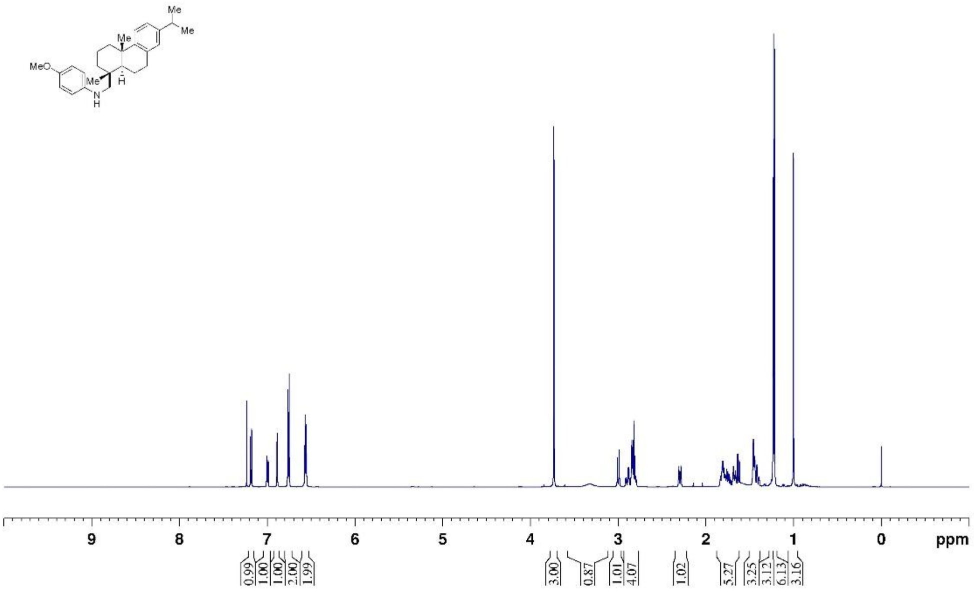

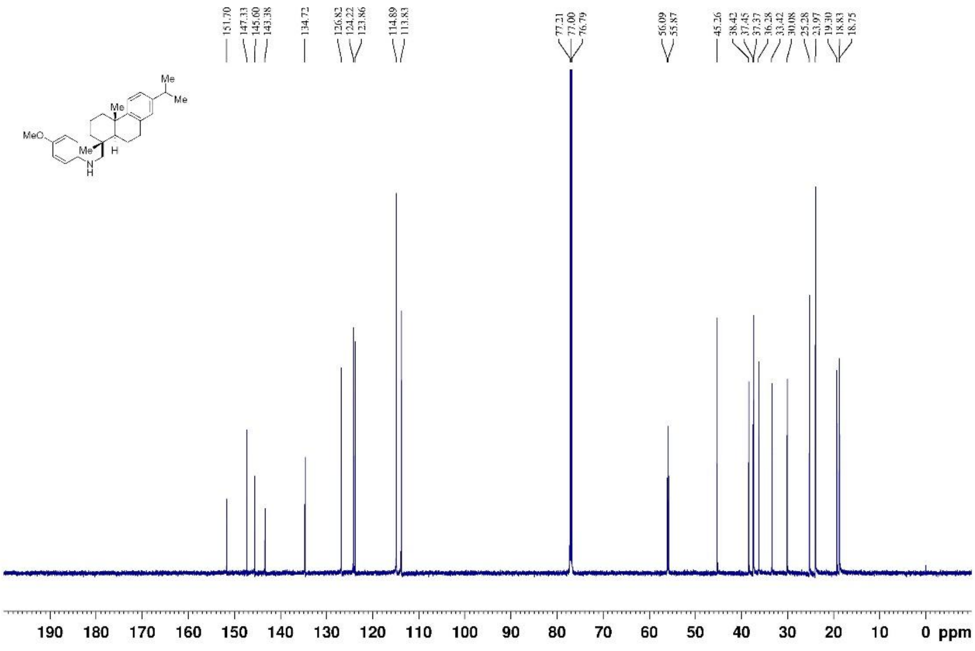

^{1}H NMR and ^{13}C NMR spectra of compound **5aw**

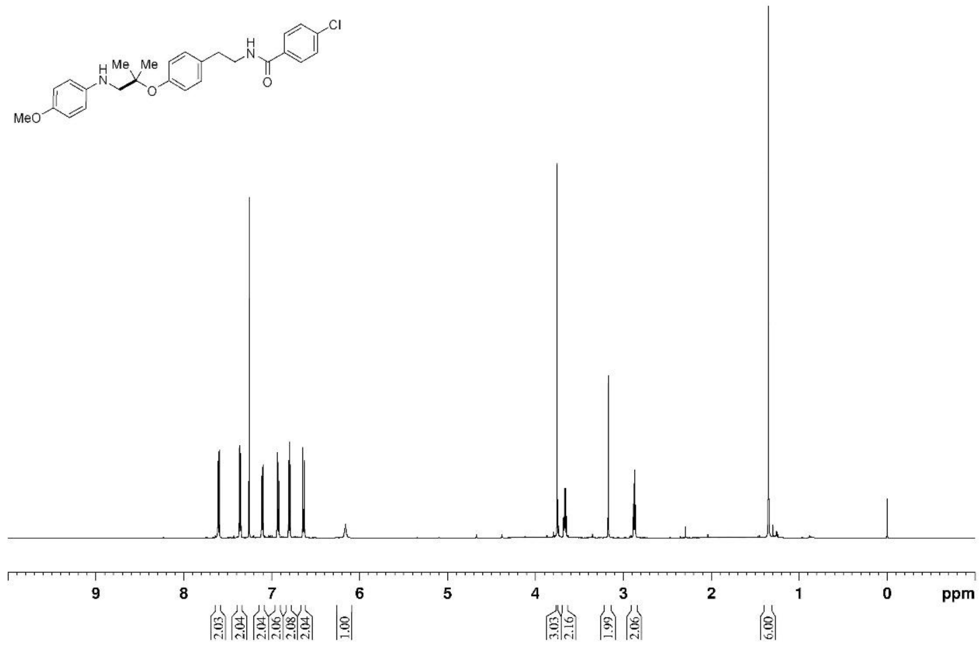

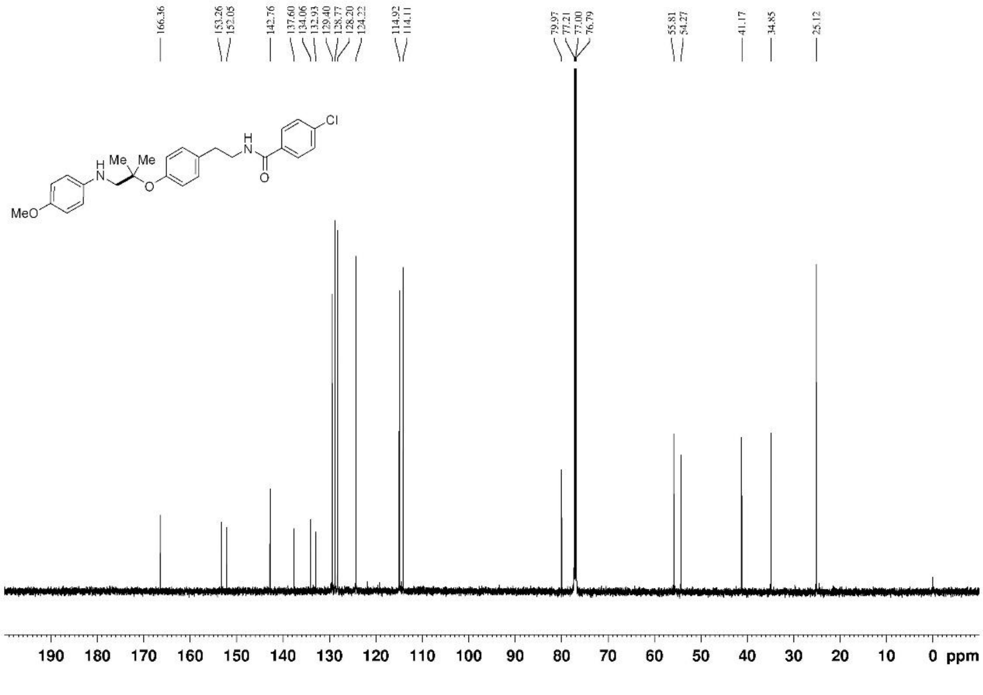

^{1}H NMR and ^{13}C NMR spectra of compound **6a**

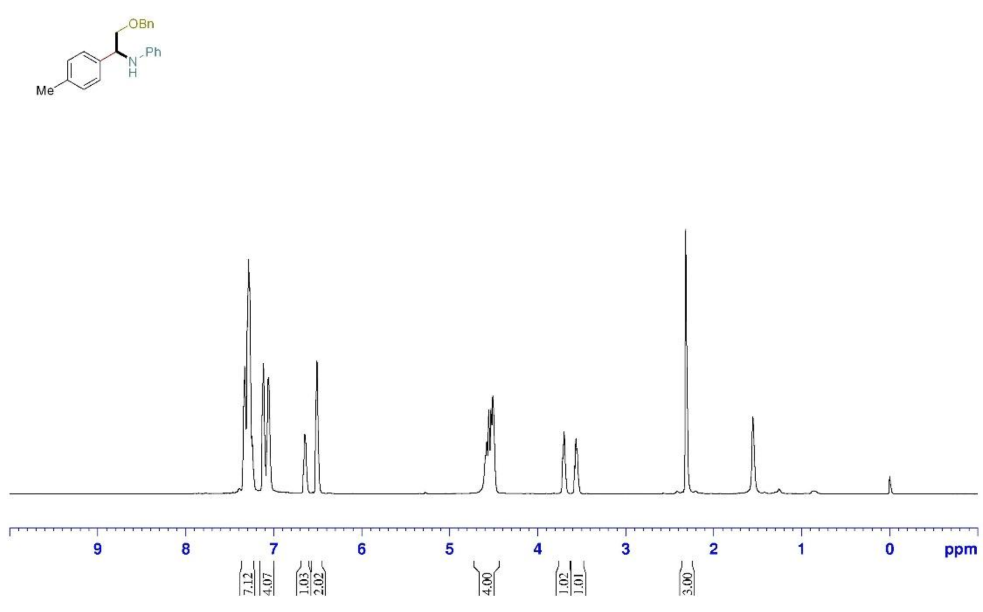

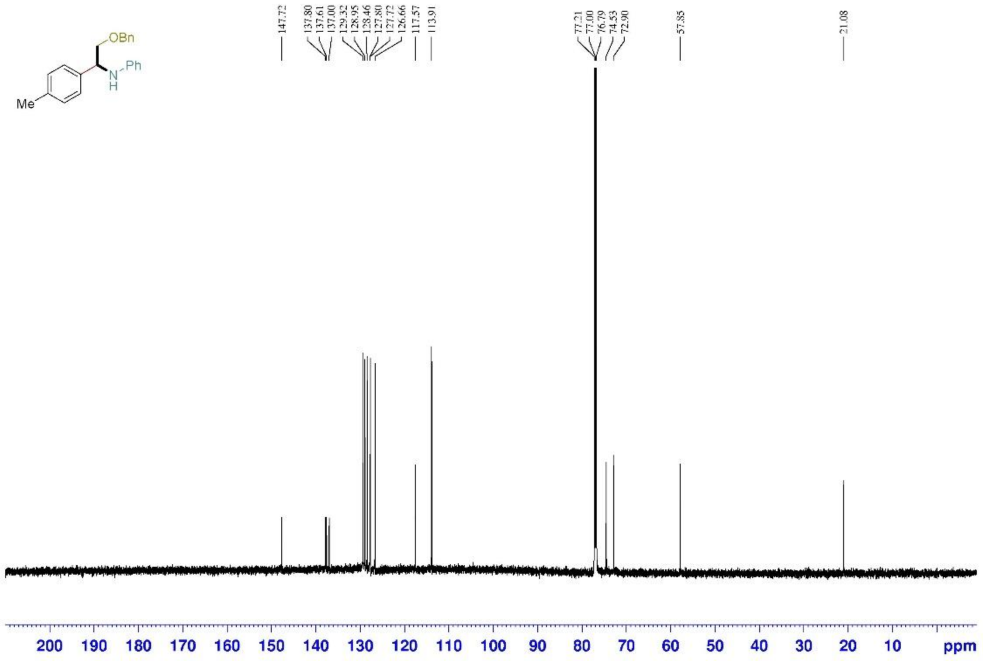

^{1}H NMR and ^{13}C NMR spectra of compound **6b**

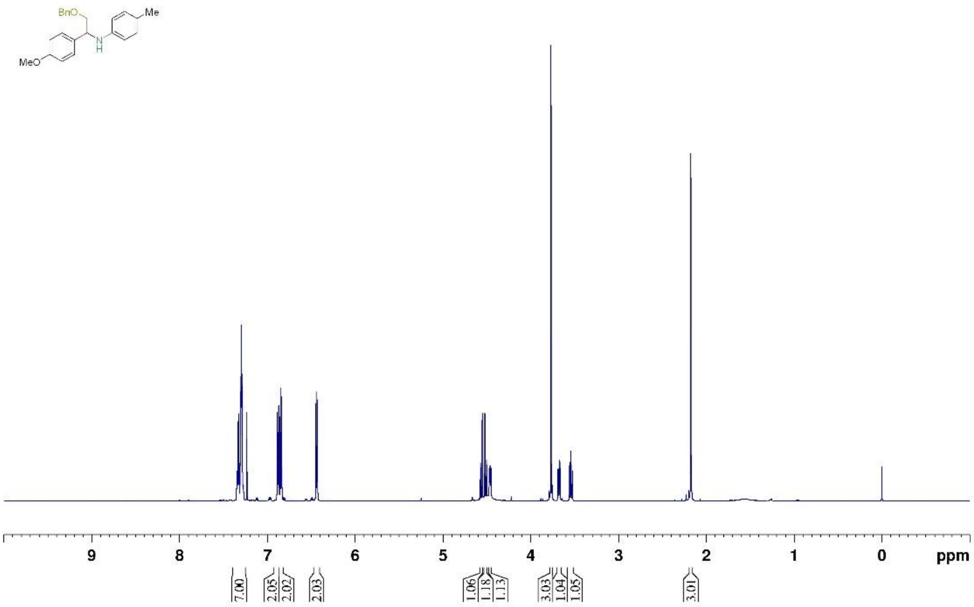

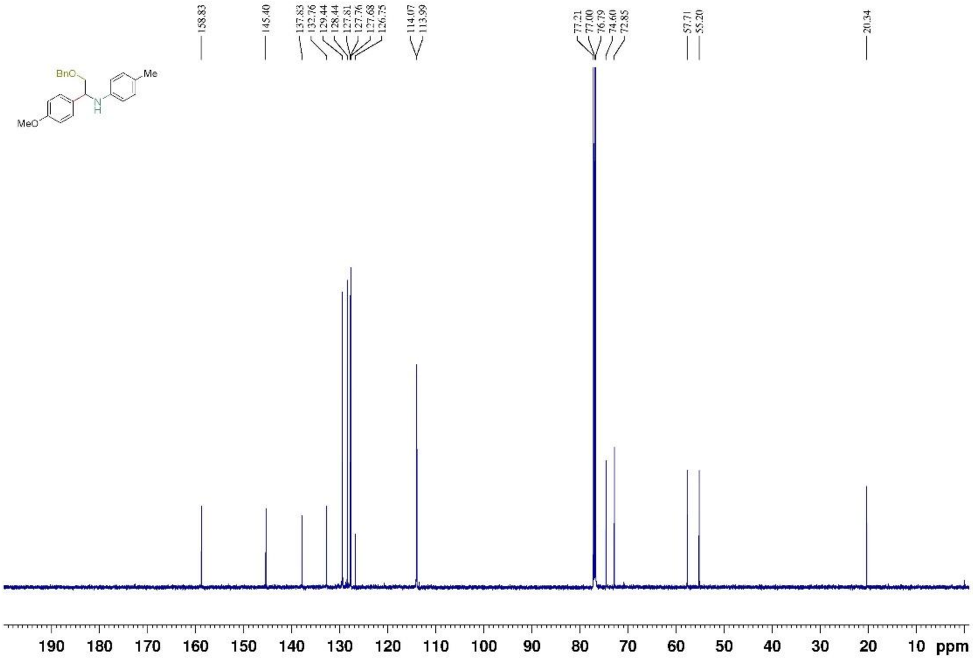

^{1}H NMR and ^{13}C NMR spectra of compound **6c**

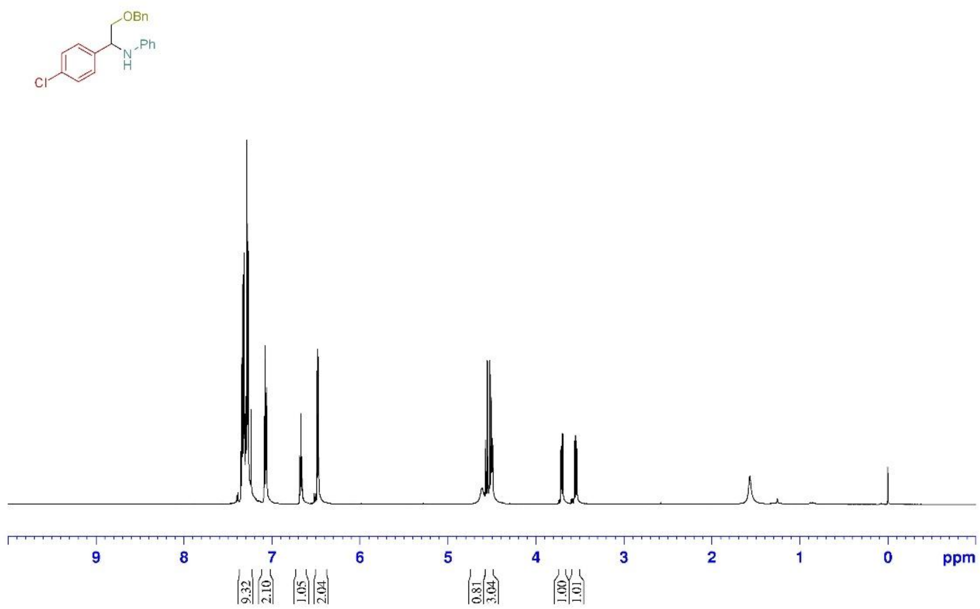

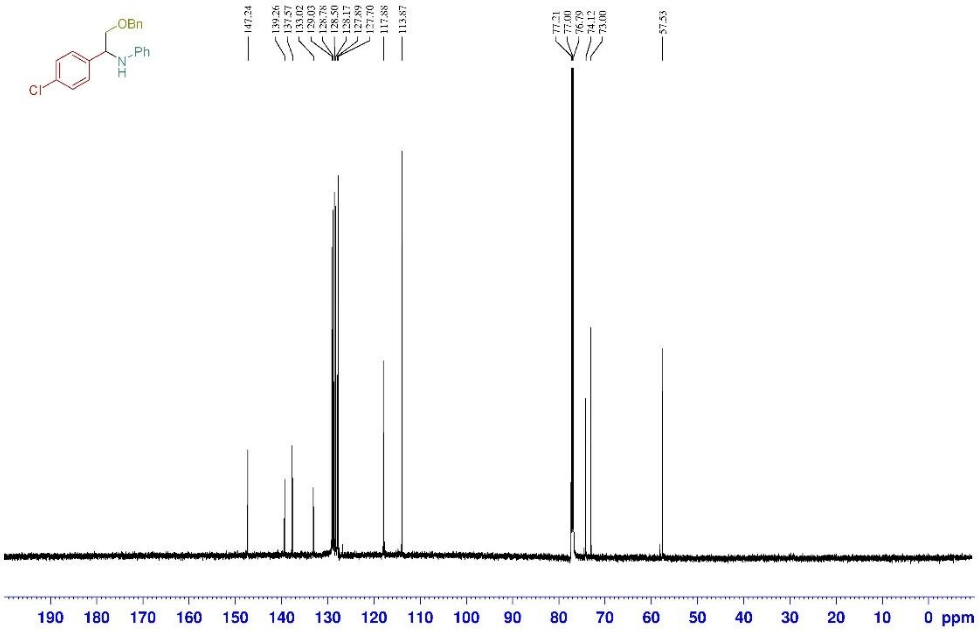

^{1}H NMR and ^{13}C NMR spectra of compound **6d**

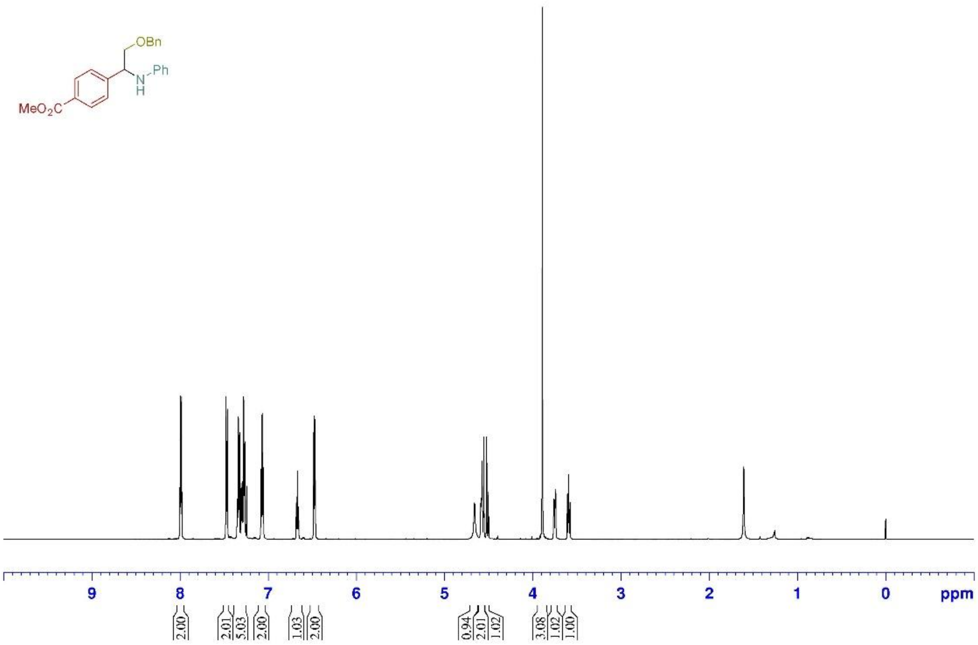

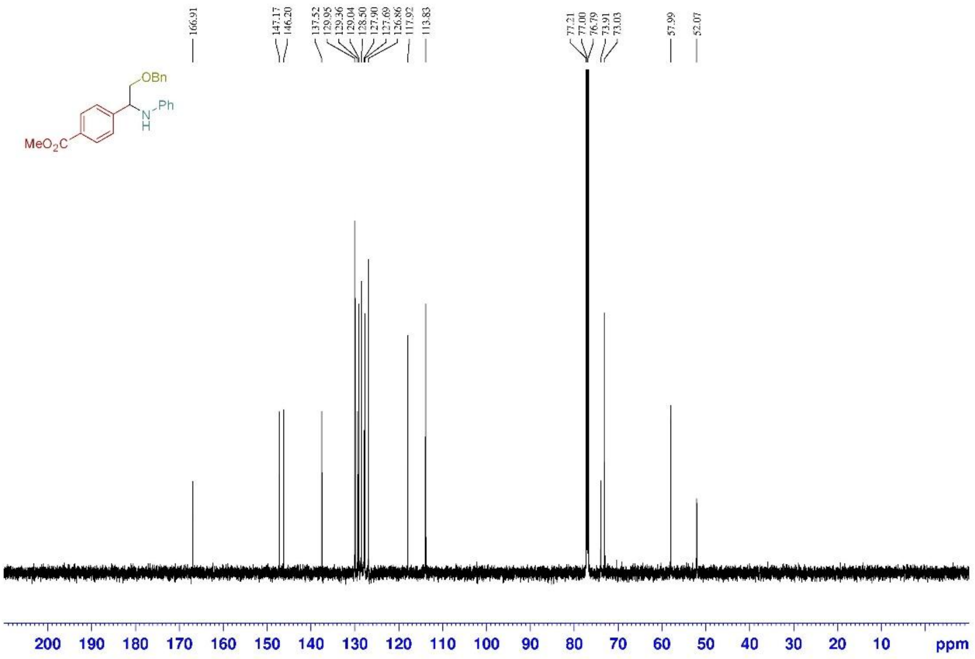

^{1}H NMR and ^{13}C NMR spectra of compound **6e**

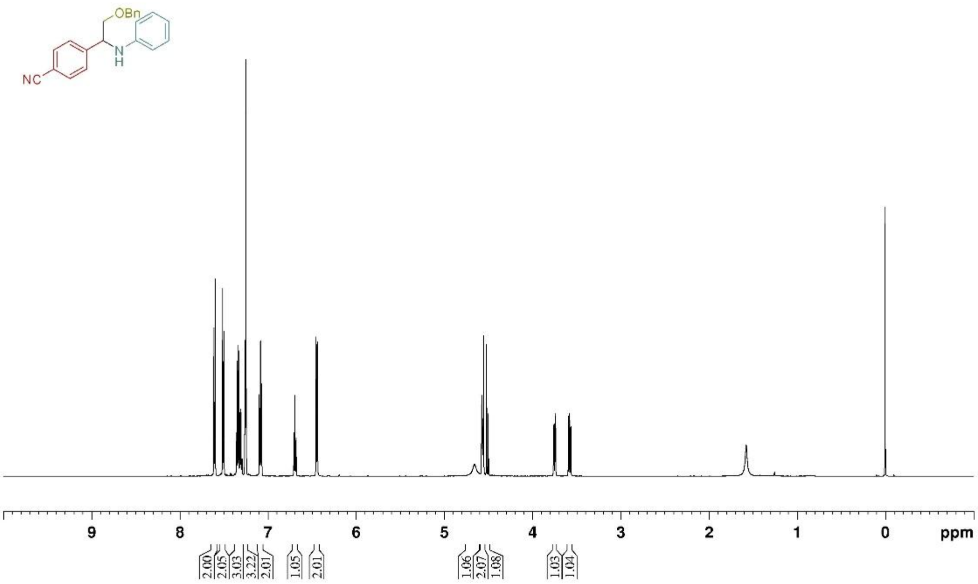

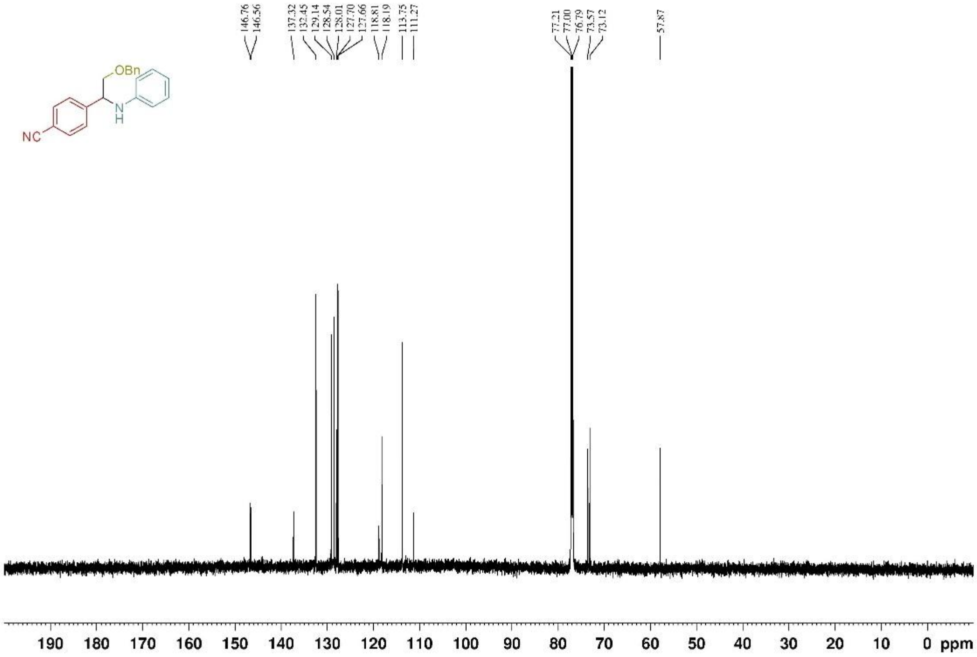

^{1}H NMR and ^{13}C NMR spectra of compound **6f**

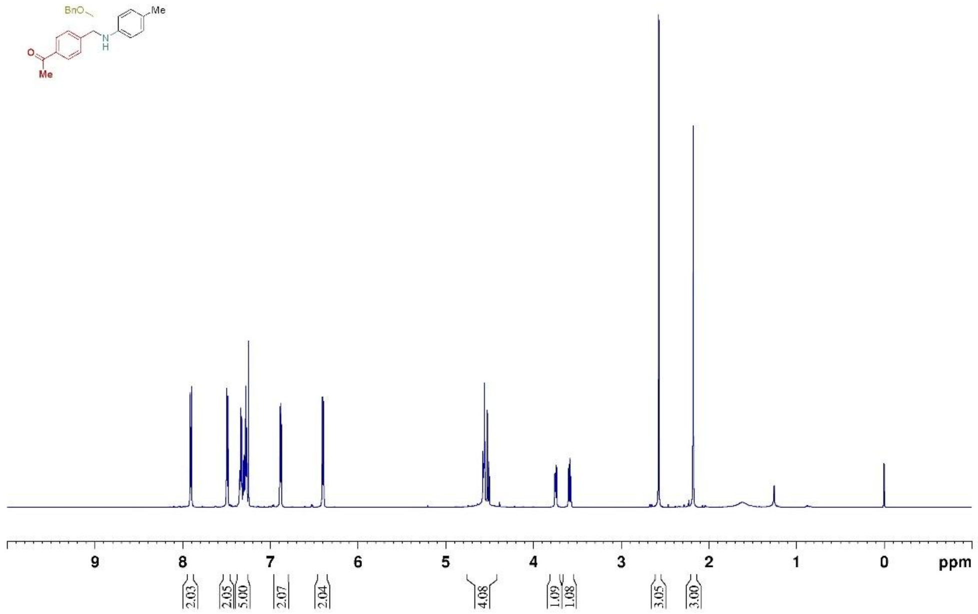

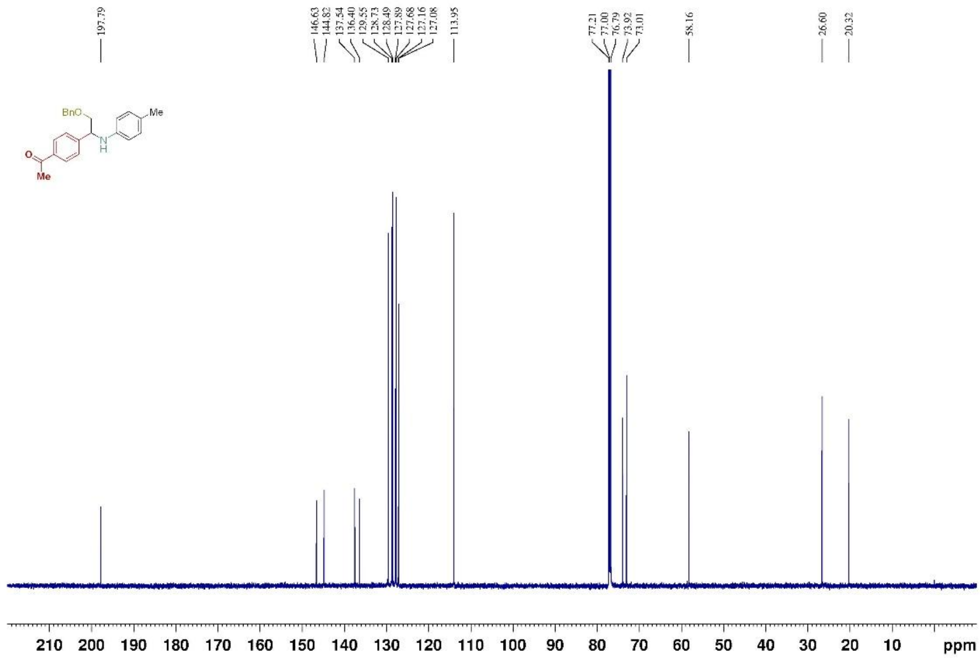

^{1}H NMR and ^{13}C NMR spectra of compound **6g**

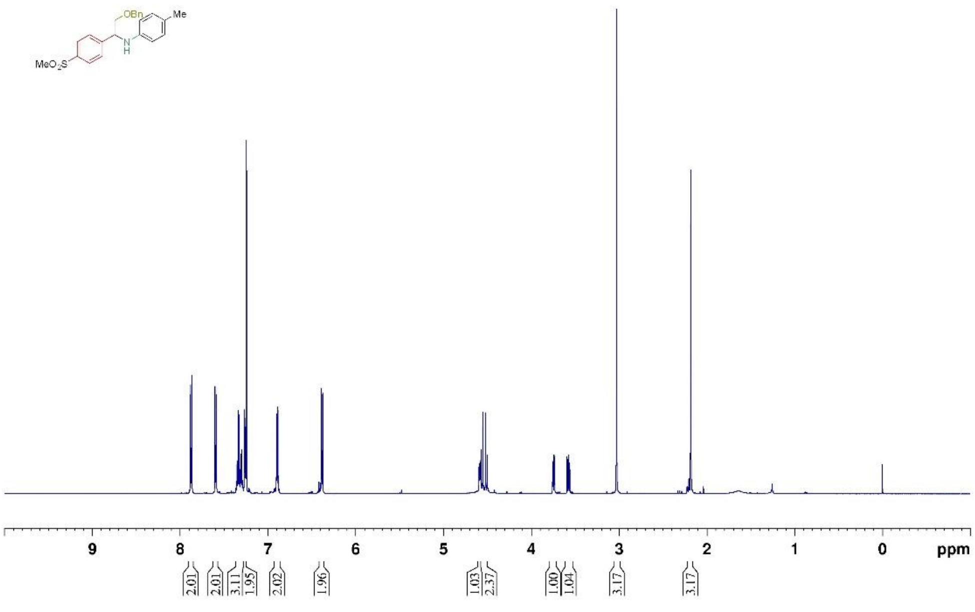

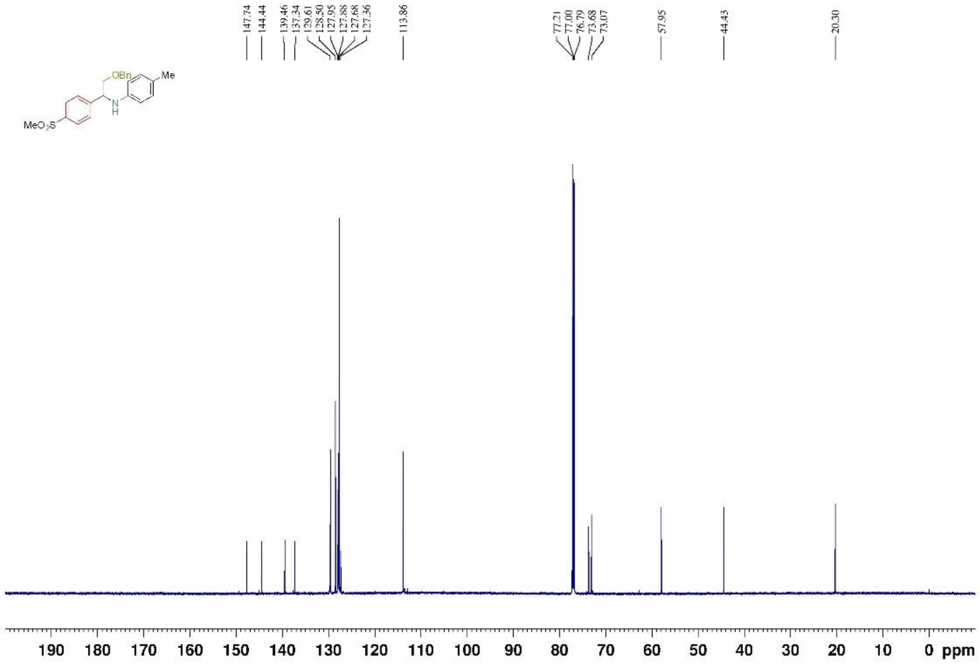

^{1}H NMR and ^{13}C NMR spectra of compound **6h**

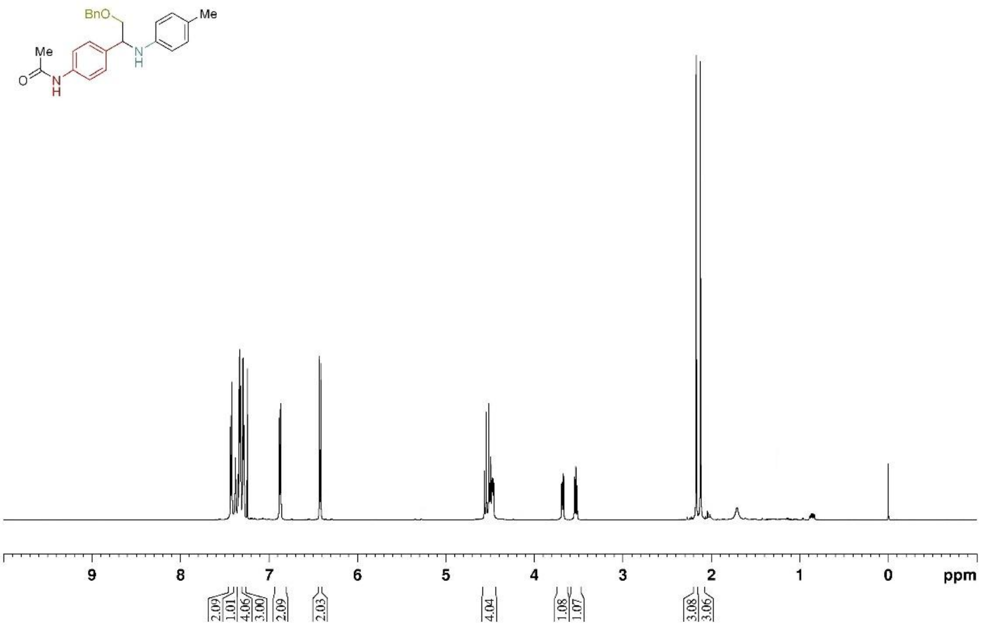

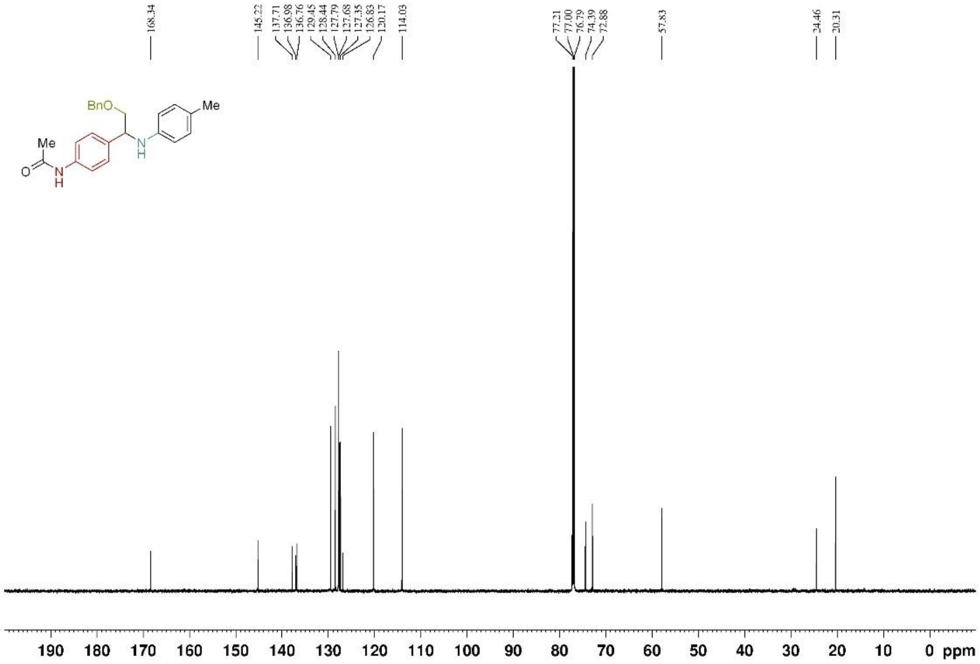

^{1}H NMR and ^{13}C NMR spectra of compound **6i**

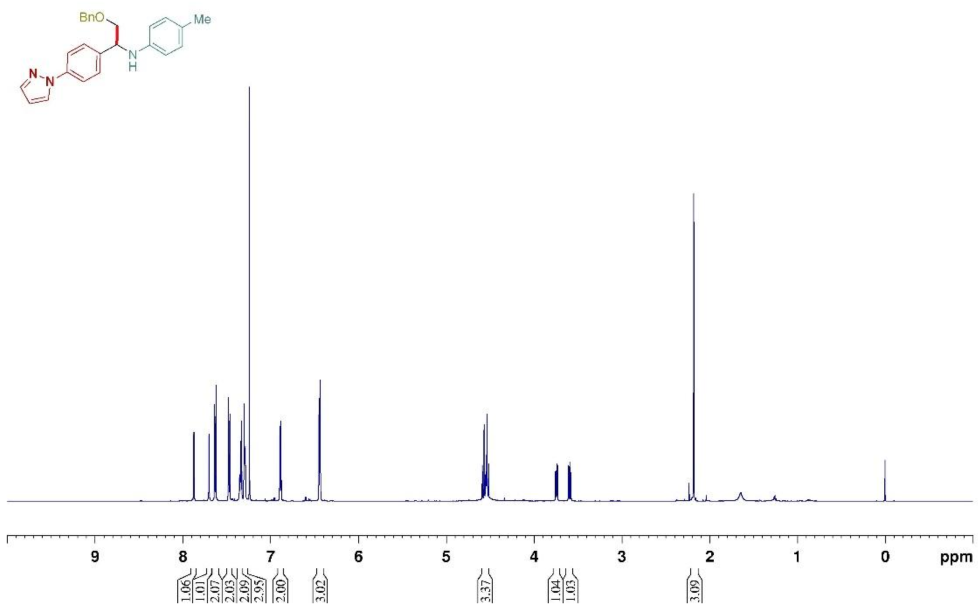

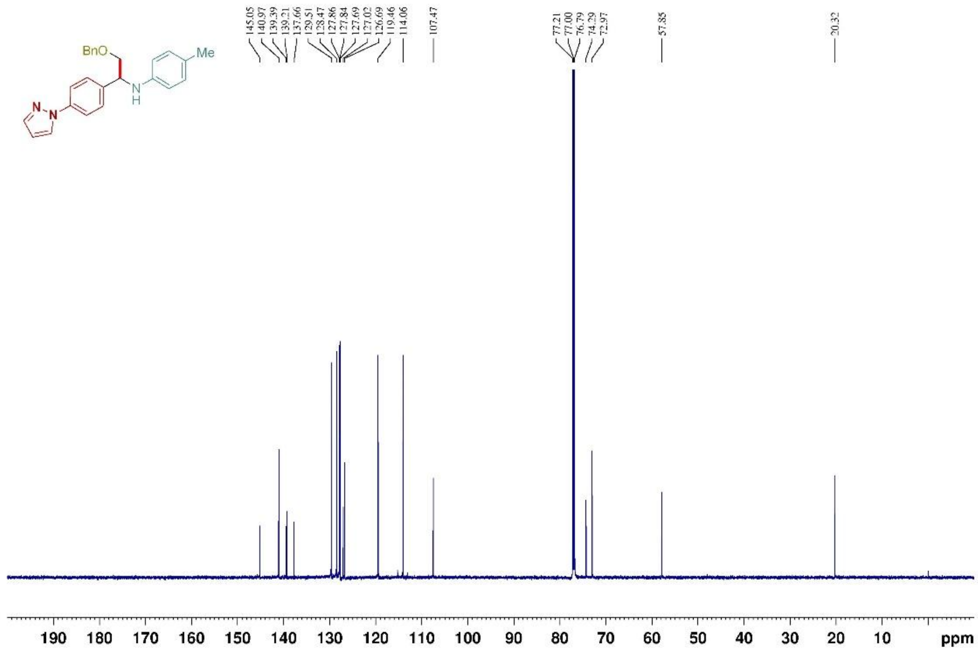

^{1}H NMR and ^{13}C NMR spectra of compound **6j**

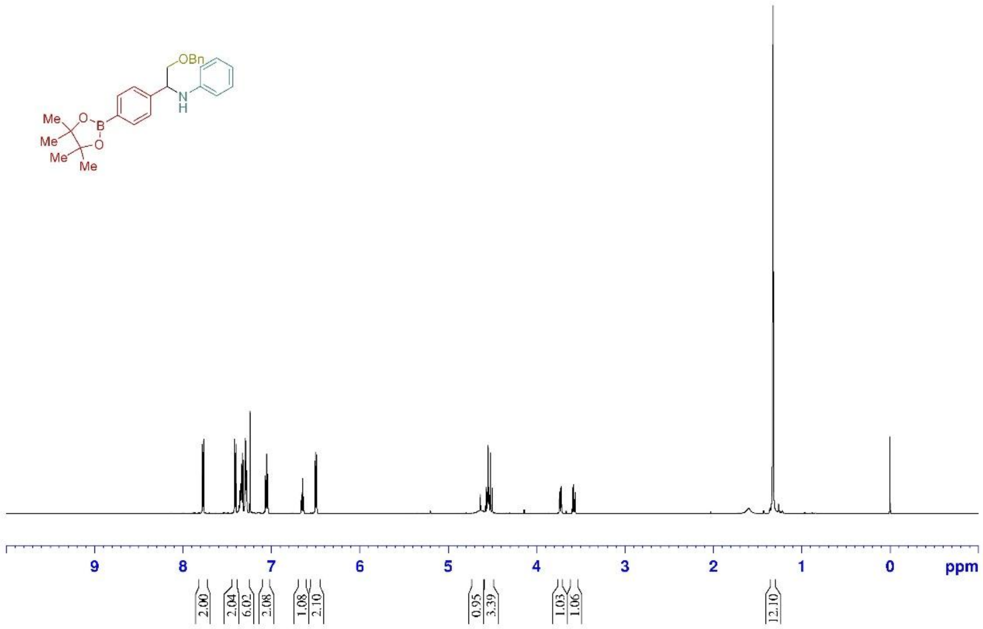

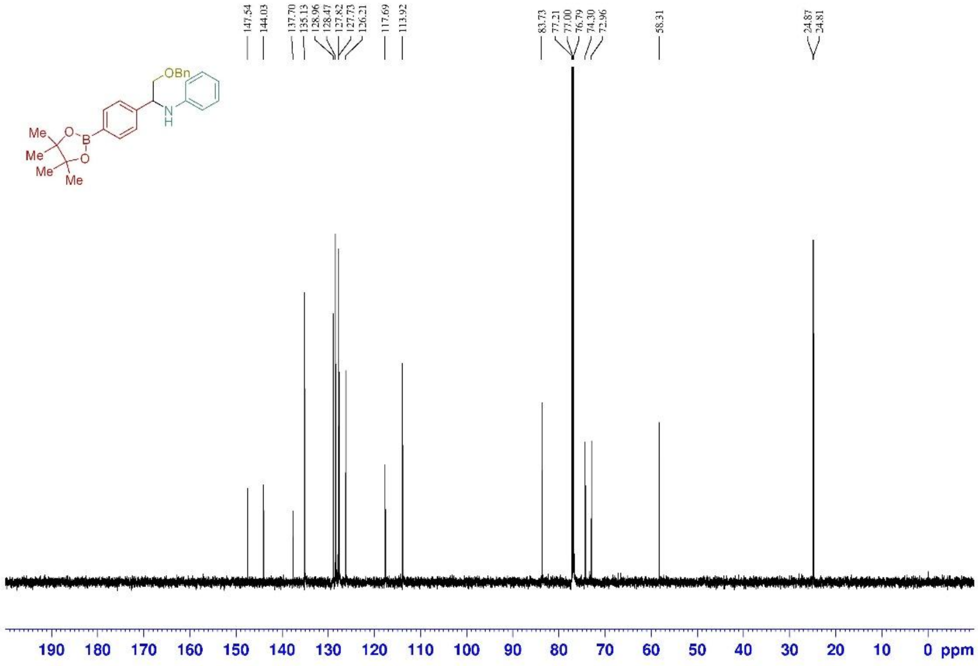

^{1}H NMR and ^{13}C NMR spectra of compound **6k**

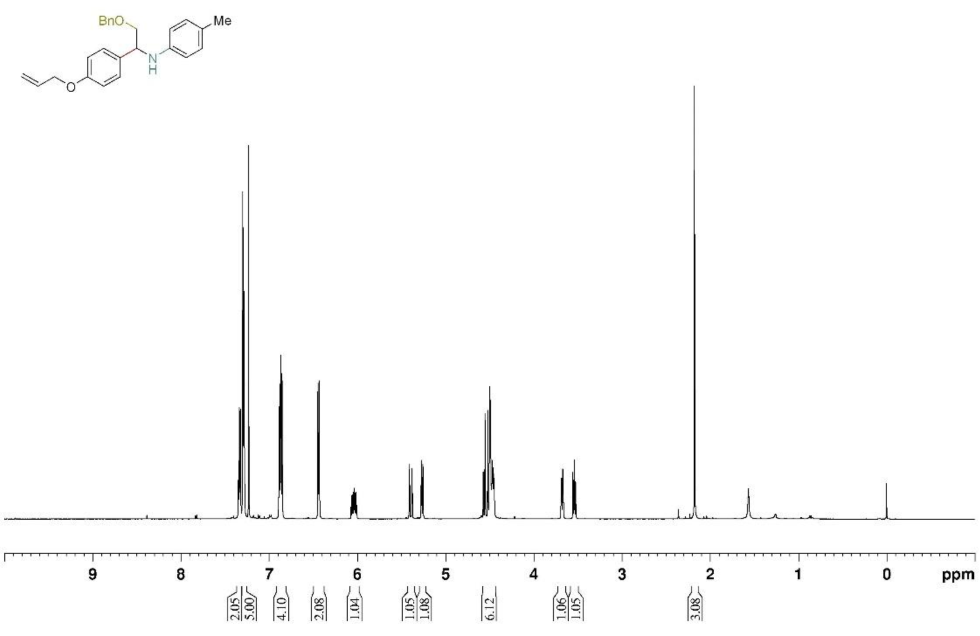

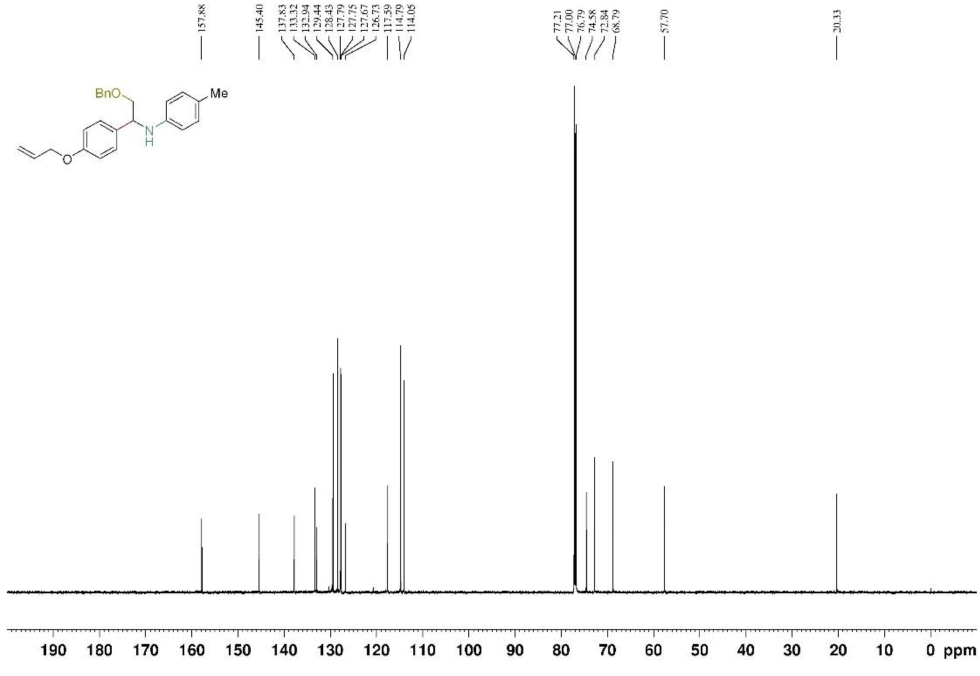

^{1}H NMR and ^{13}C NMR spectra of compound **6l**

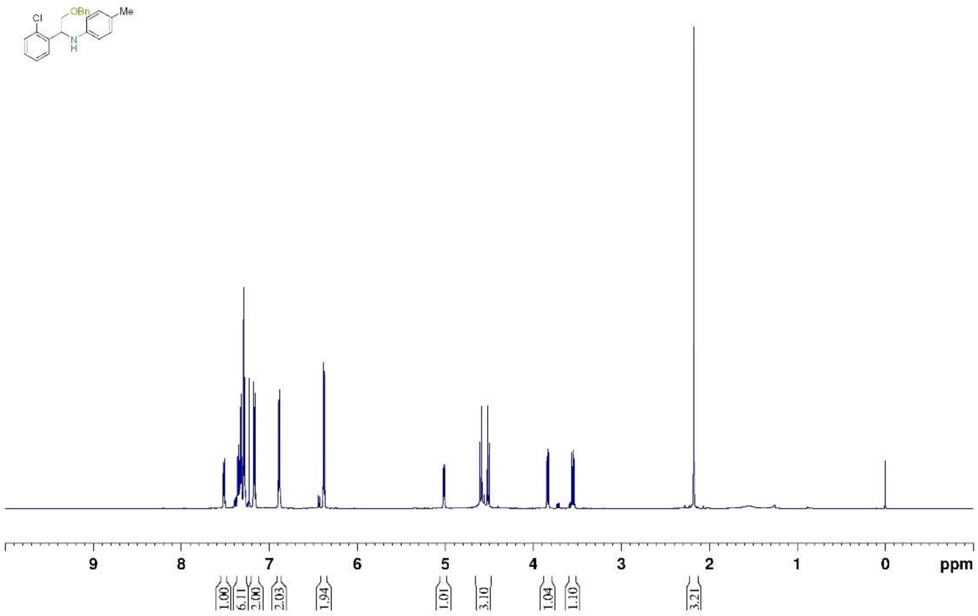

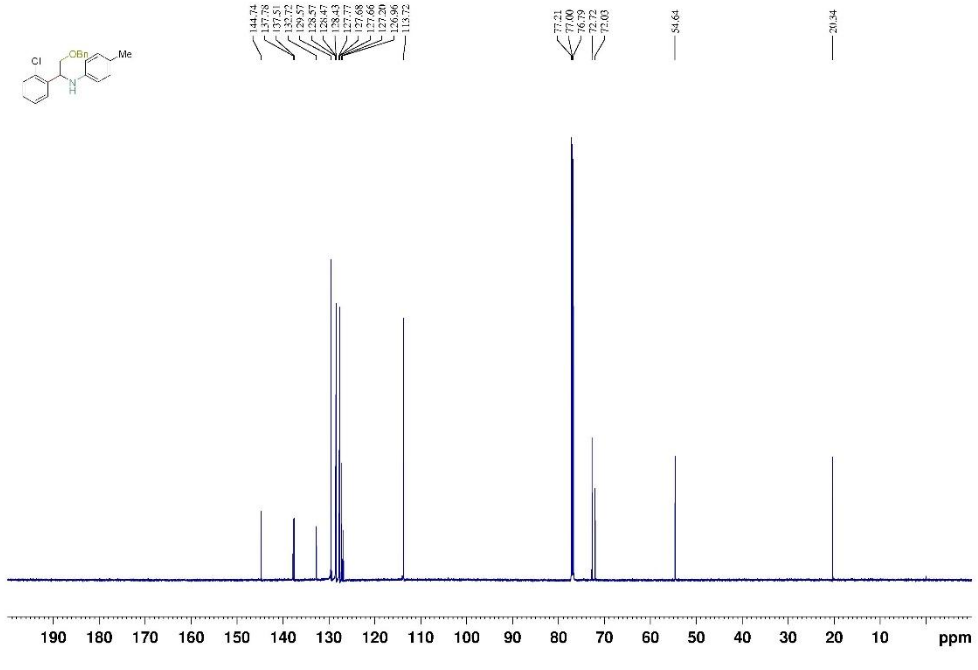

^{1}H NMR and ^{13}C NMR spectra of compound **6m**

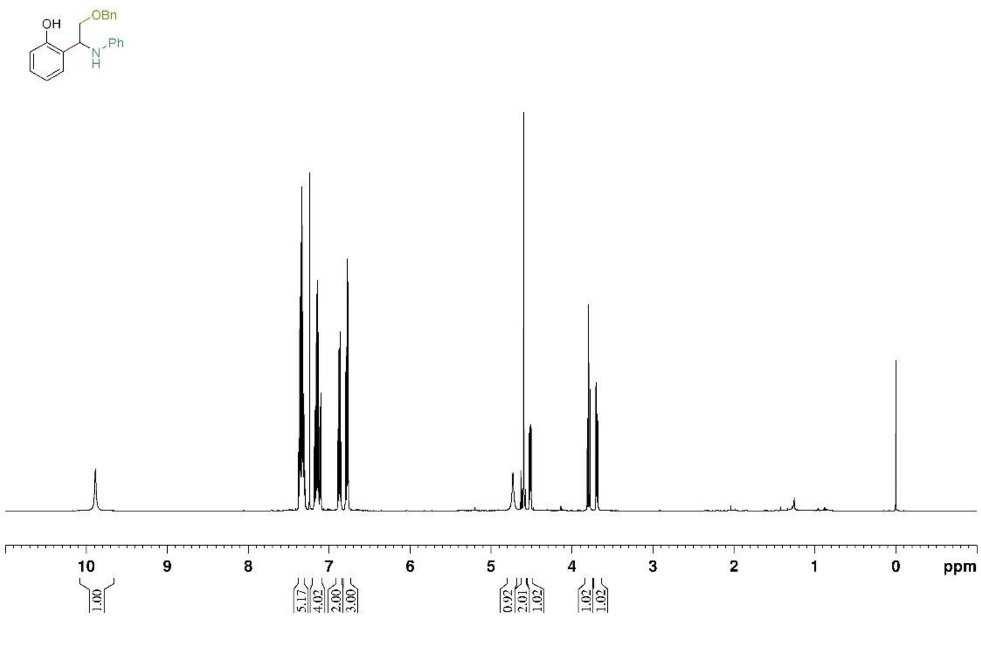

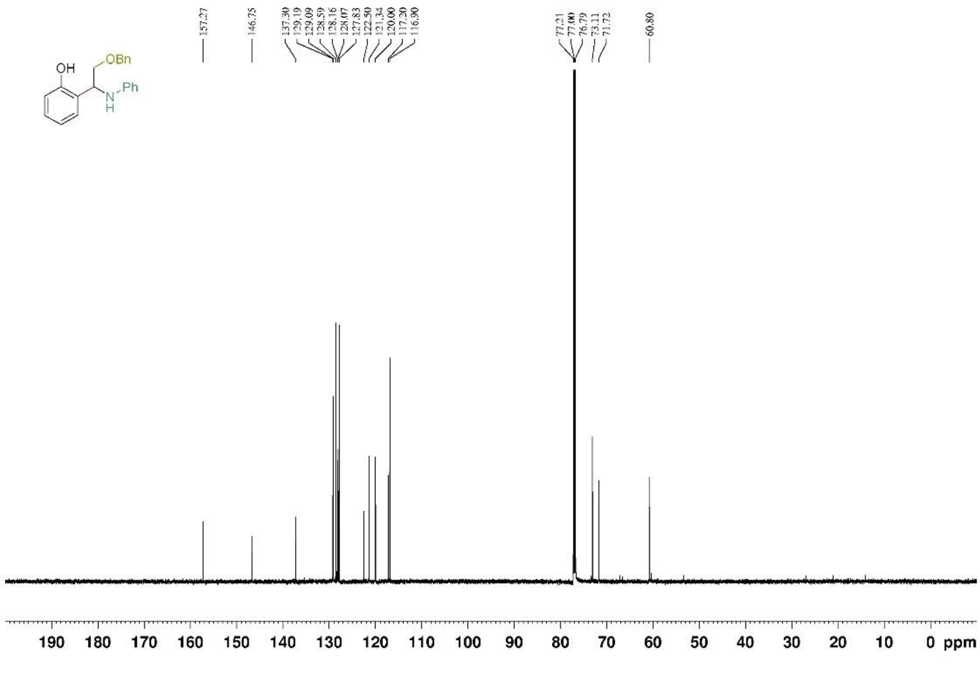

^{1}H NMR and ^{13}C NMR spectra of compound **6n**

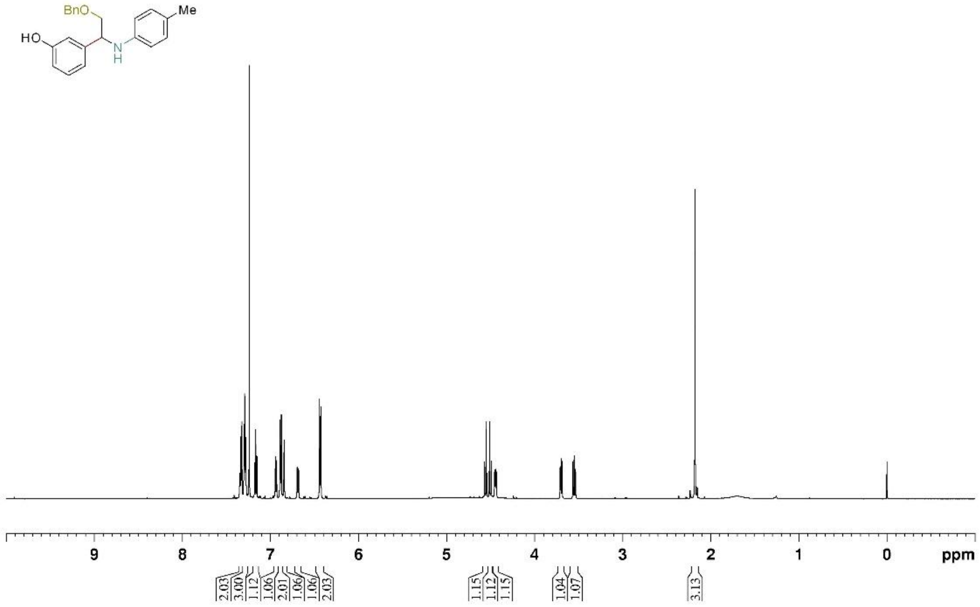

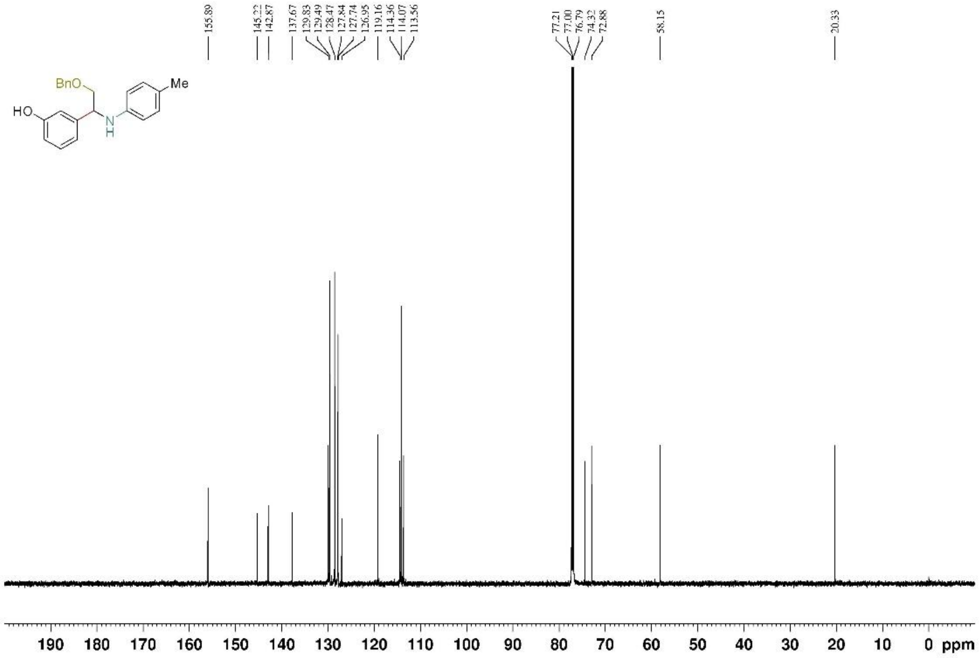

^{1}H NMR and ^{13}C NMR spectra of compound **6o**

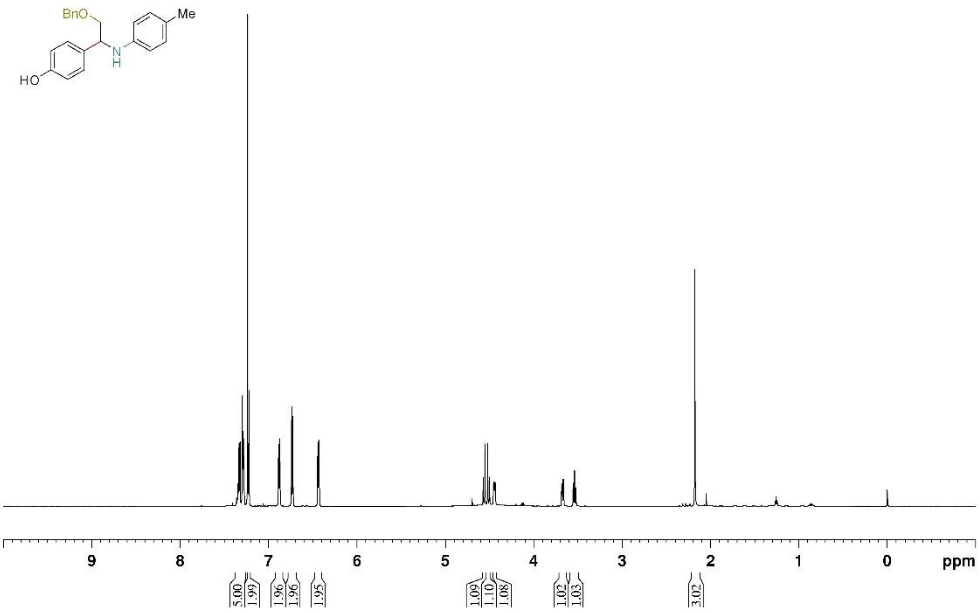

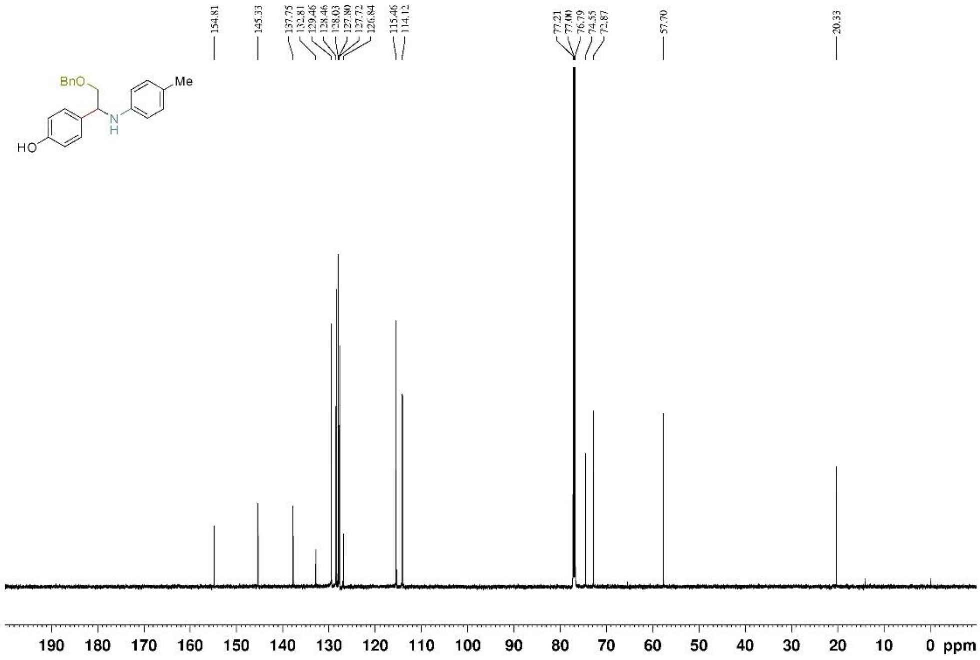

^{1}H NMR and ^{13}C NMR spectra of compound **6p**

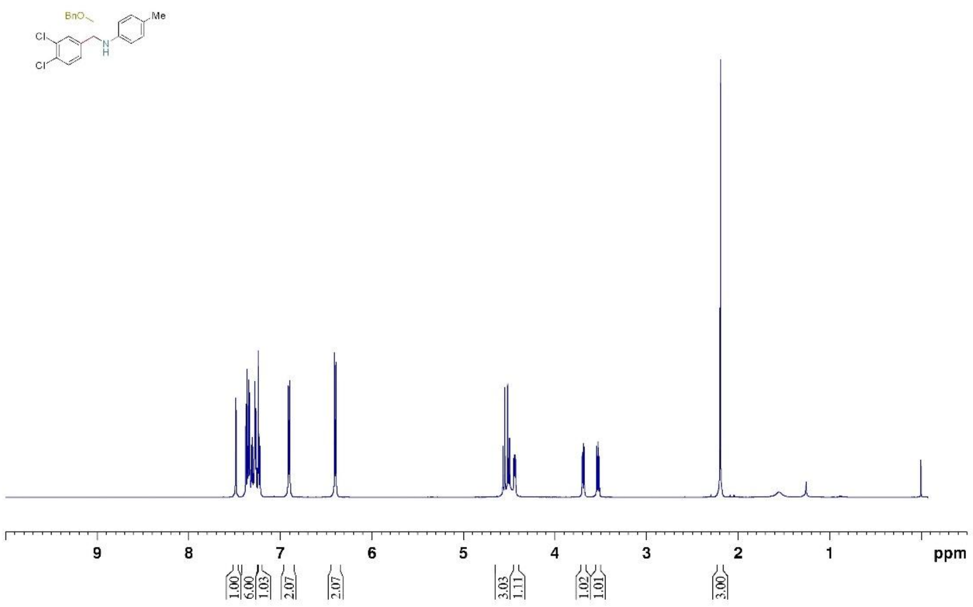

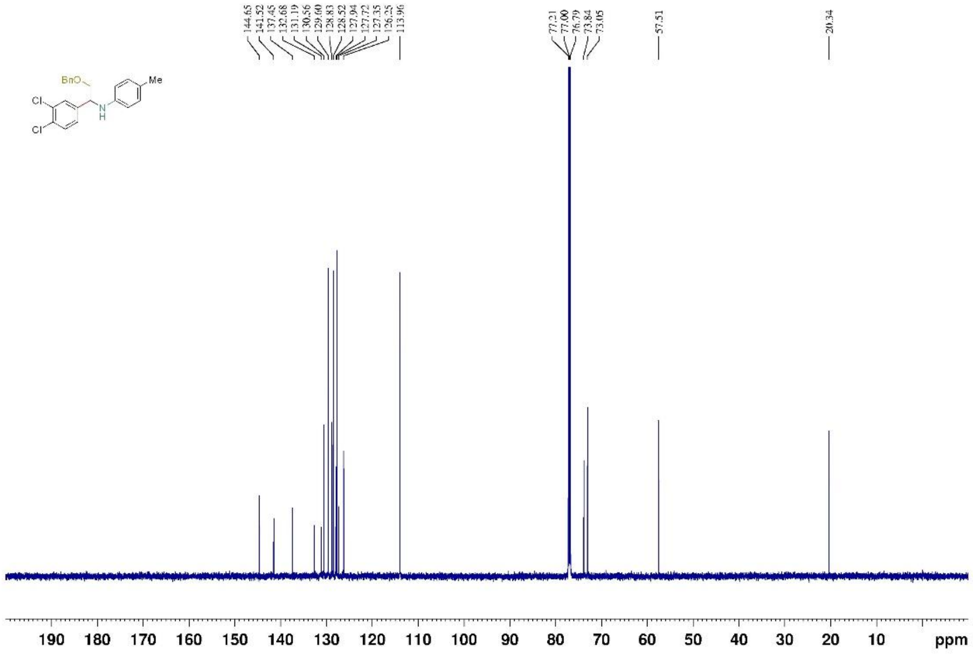

^{1}H NMR and ^{13}C NMR spectra of compound **6q**

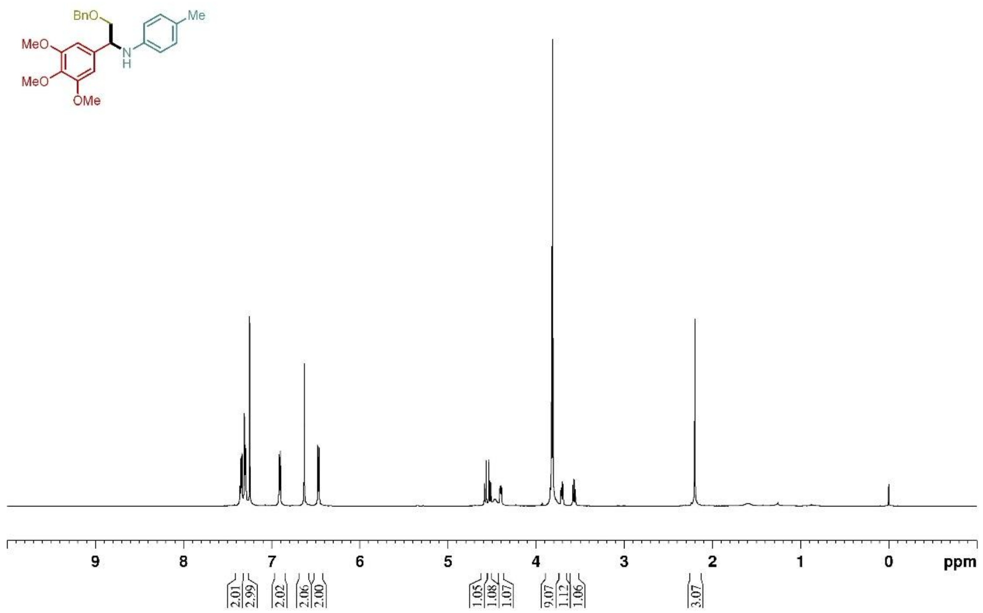

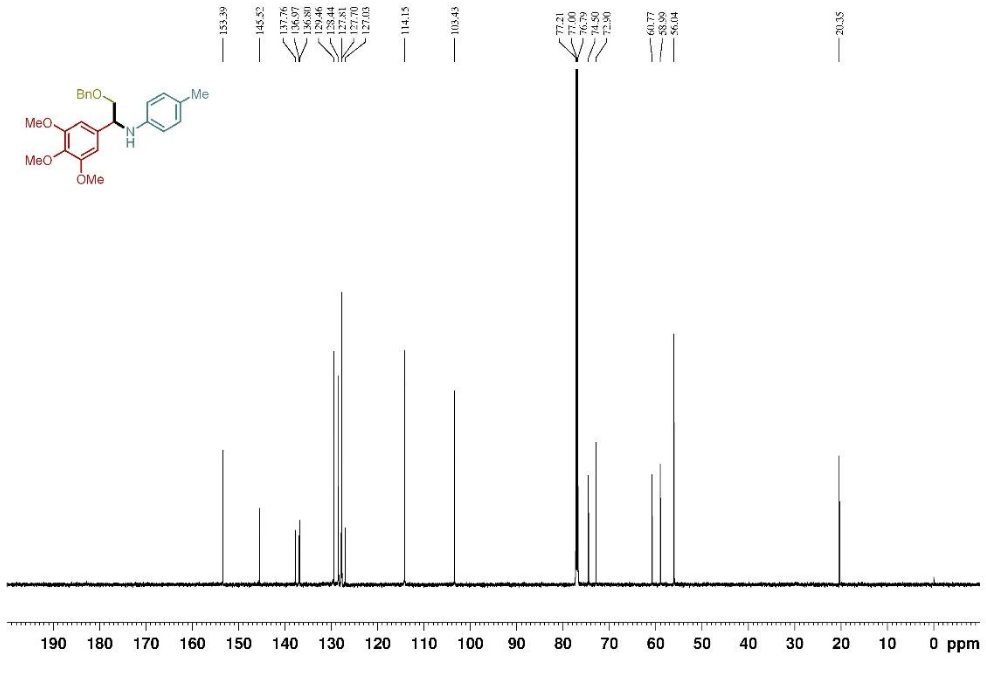

^{1}H NMR and ^{13}C NMR spectra of compound **6r**

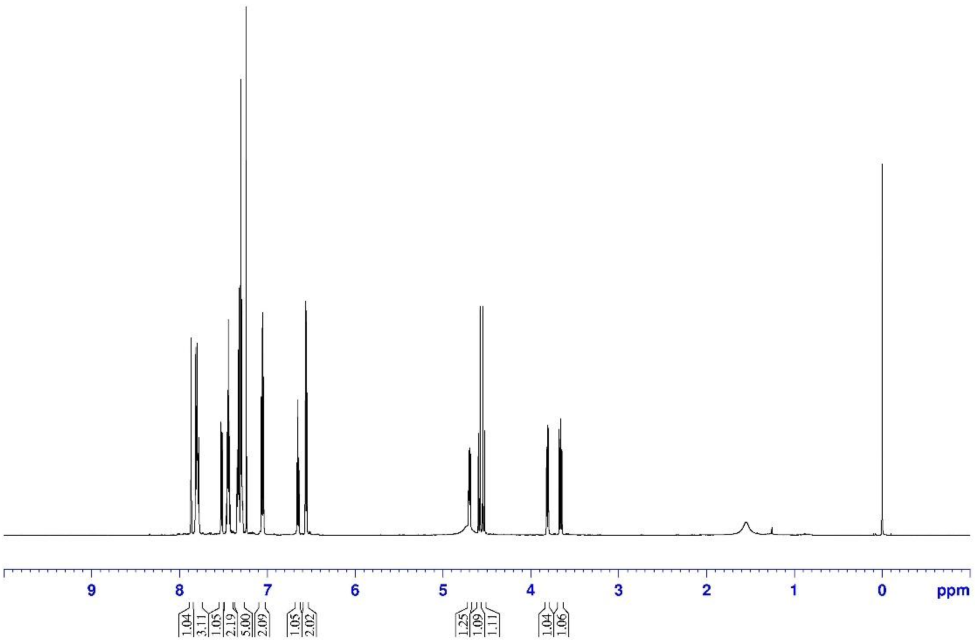

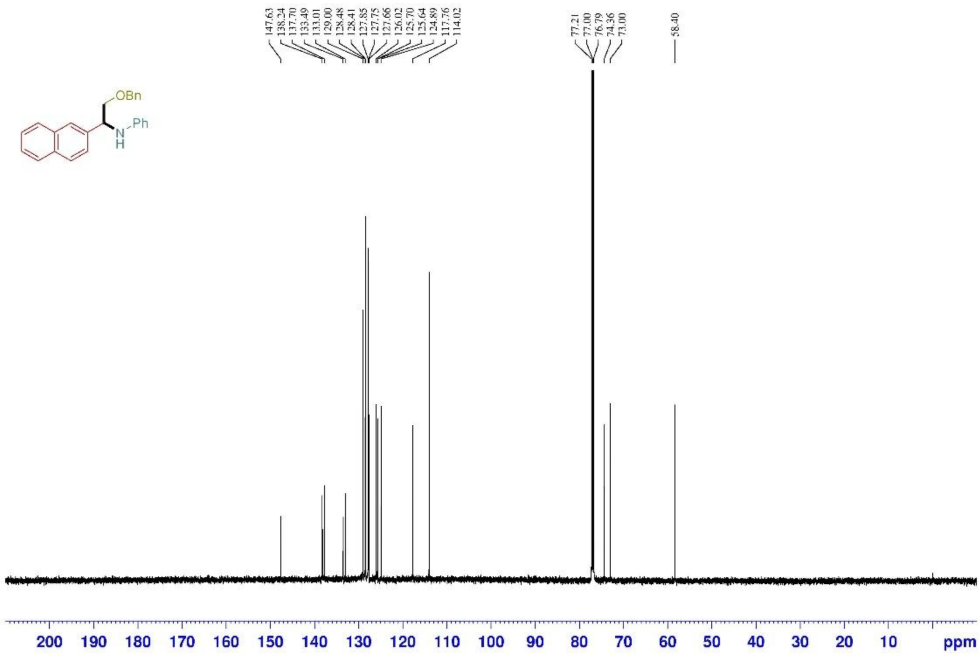

^{1}H NMR and ^{13}C NMR spectra of compound **6s**

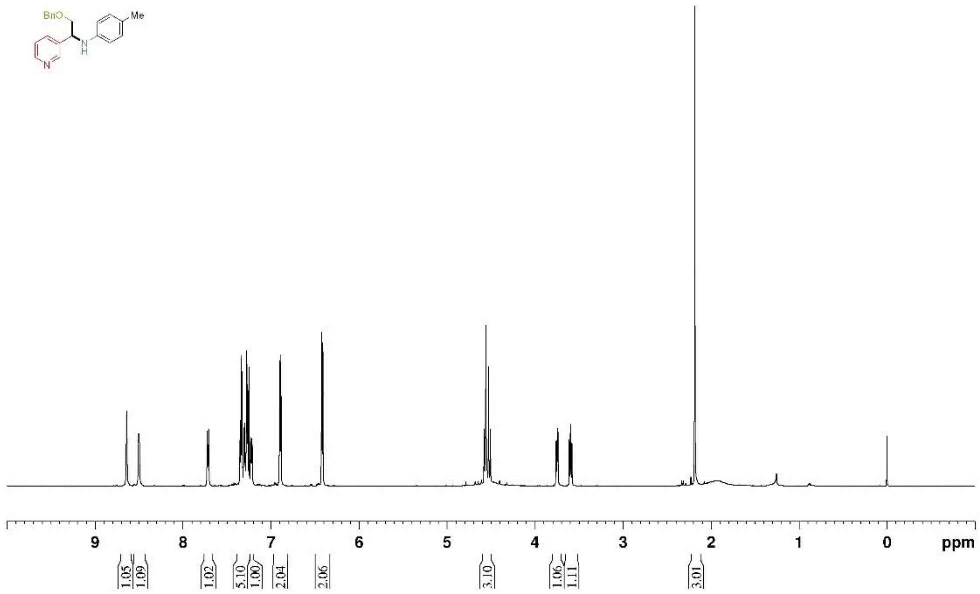

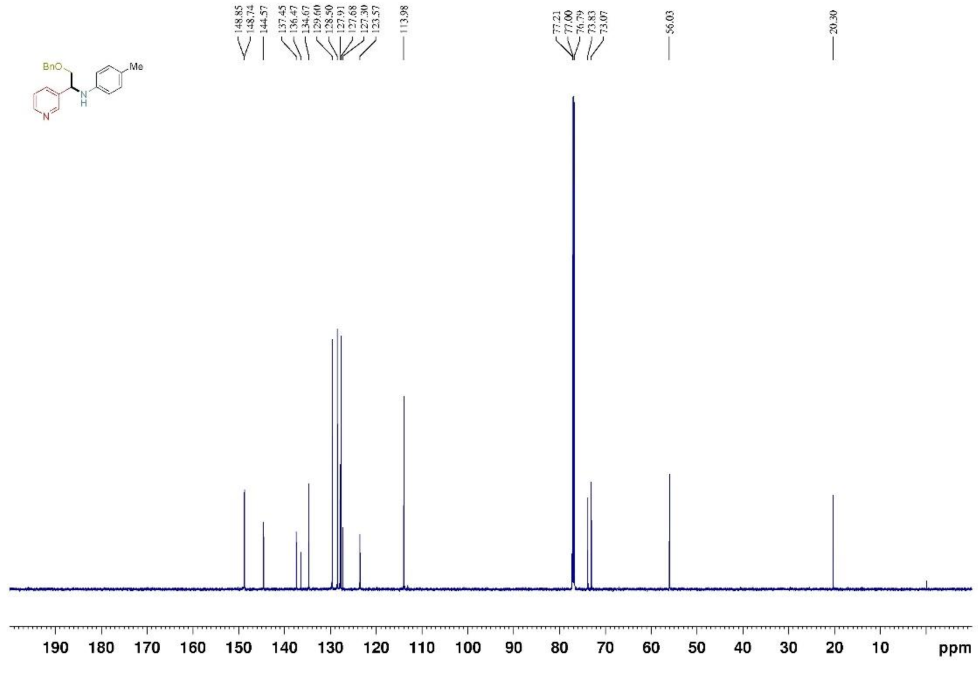

^{1}H NMR and ^{13}C NMR spectra of compound **6t**

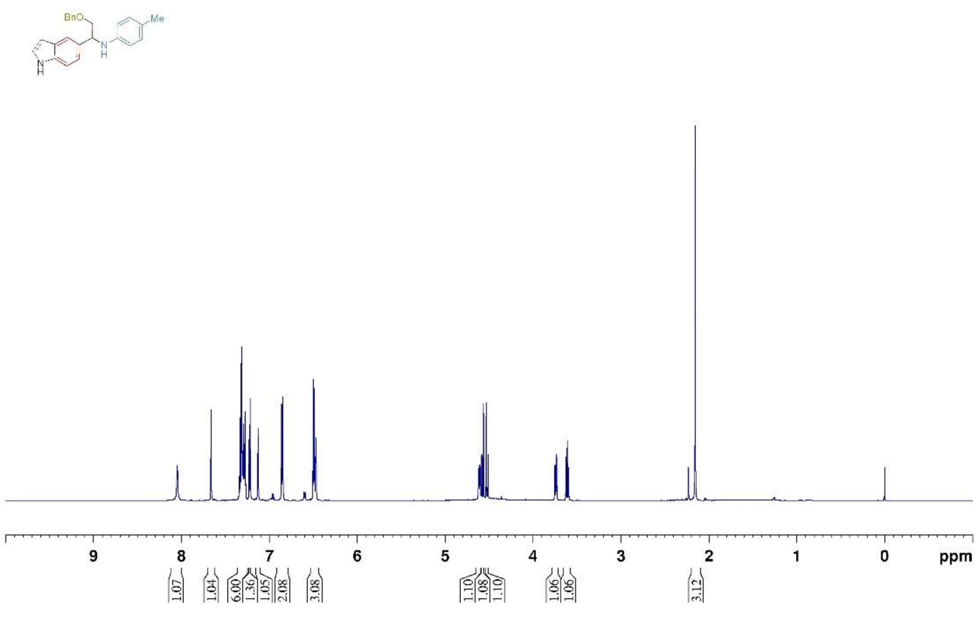

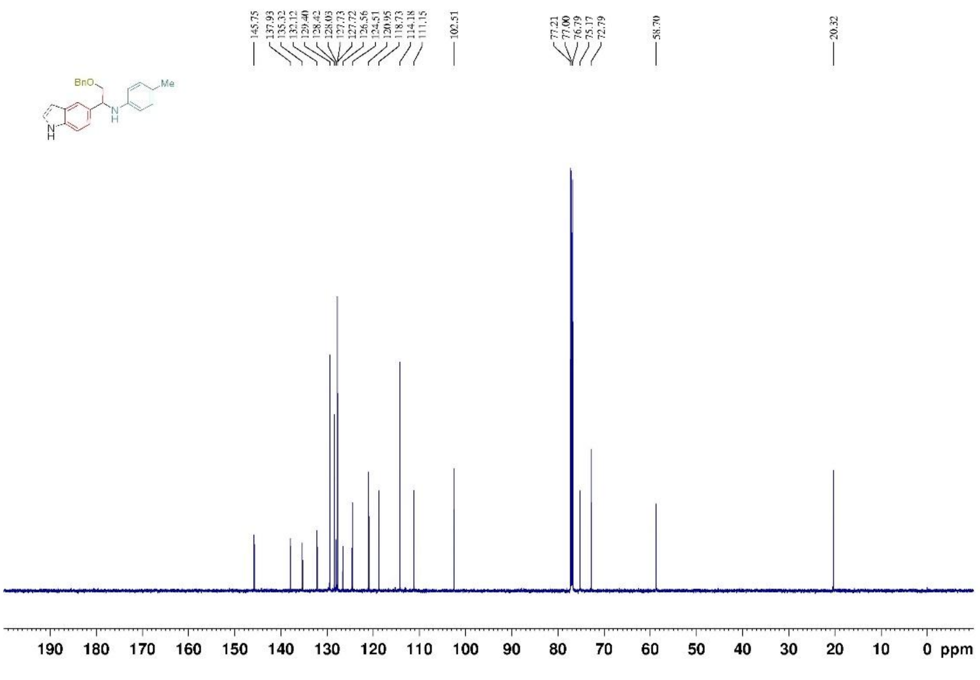

^{1}H NMR and ^{13}C NMR spectra of compound **6u**

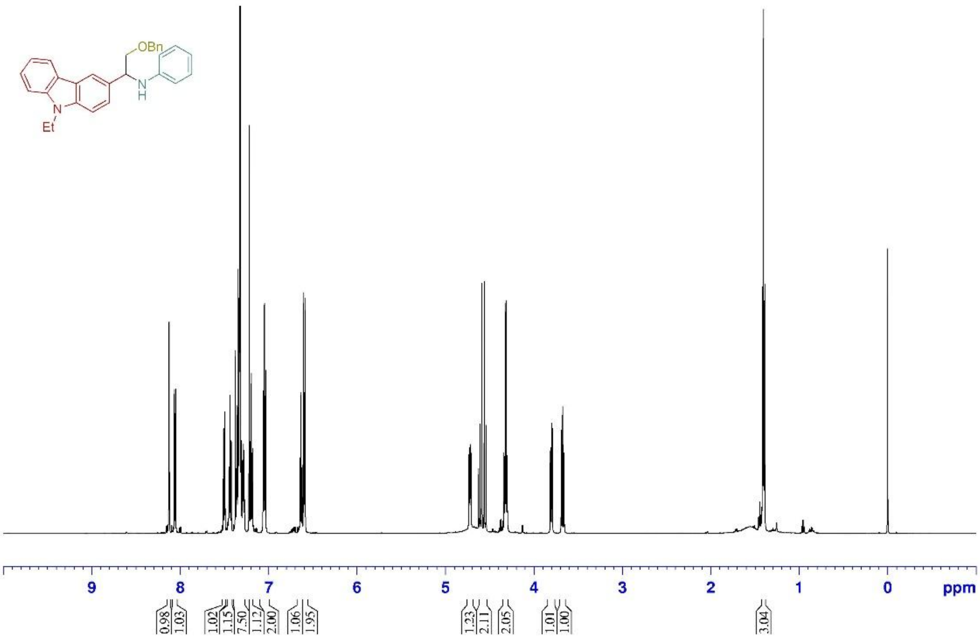

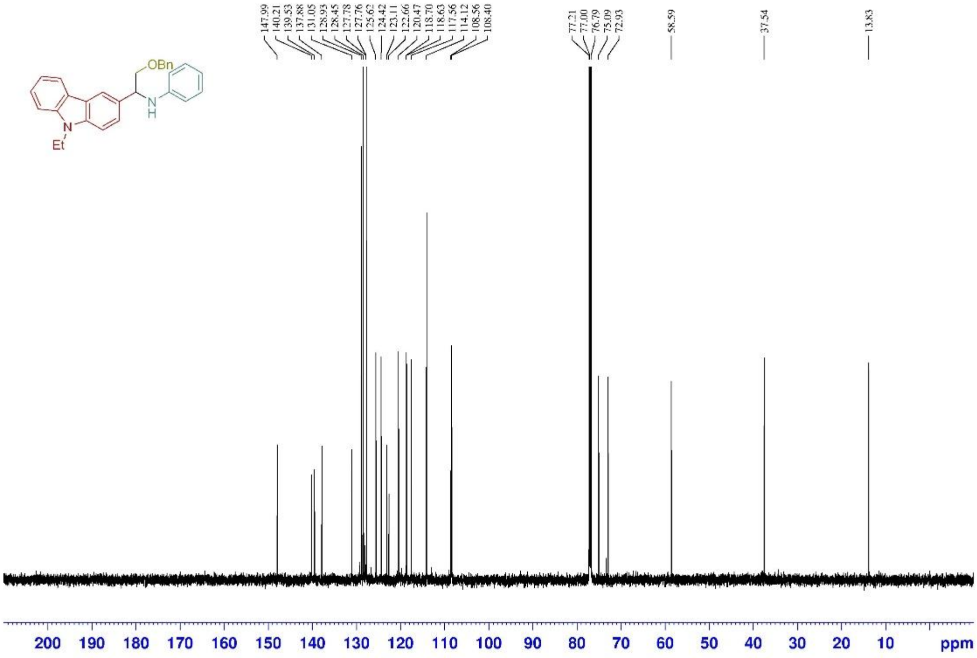

^{1}H NMR and ^{13}C NMR spectra of compound **6v**

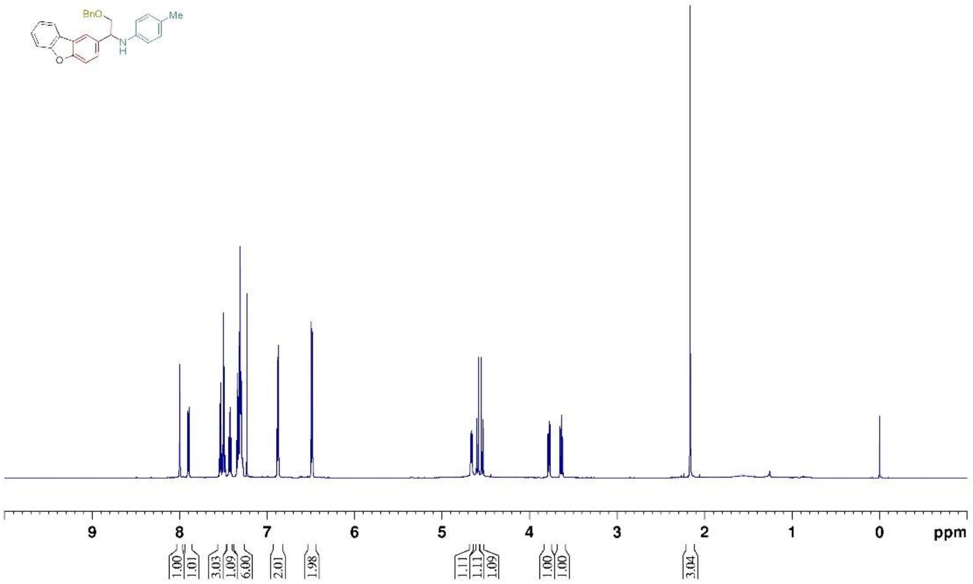

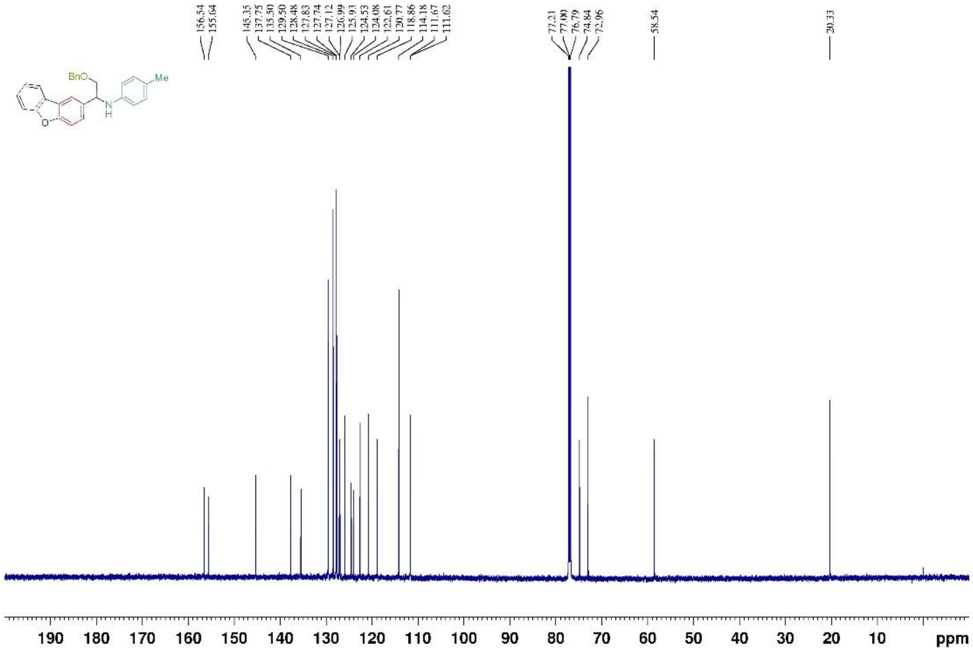

^{1}H NMR and ^{13}C NMR spectra of compound **6w**

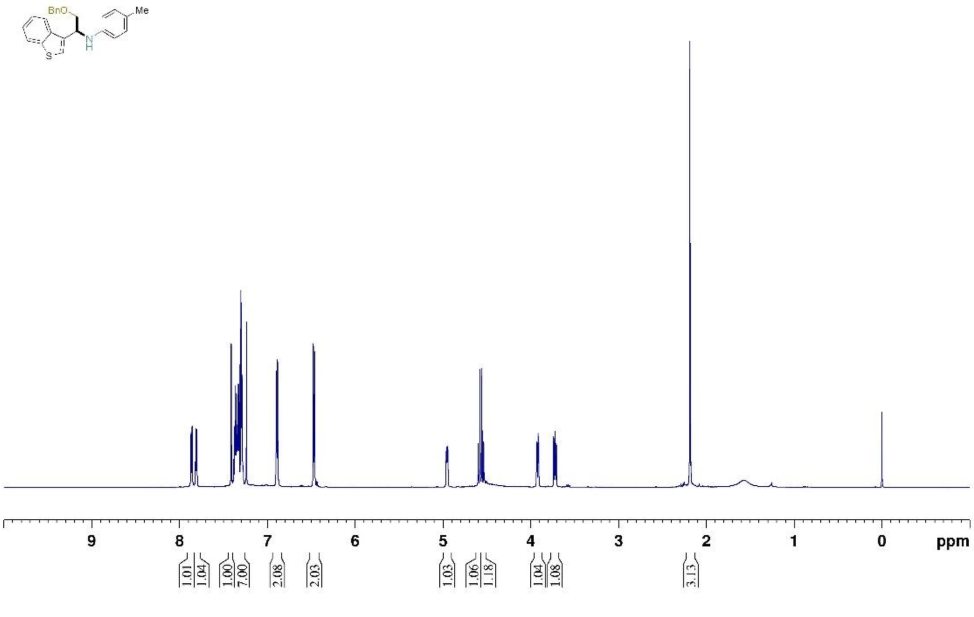

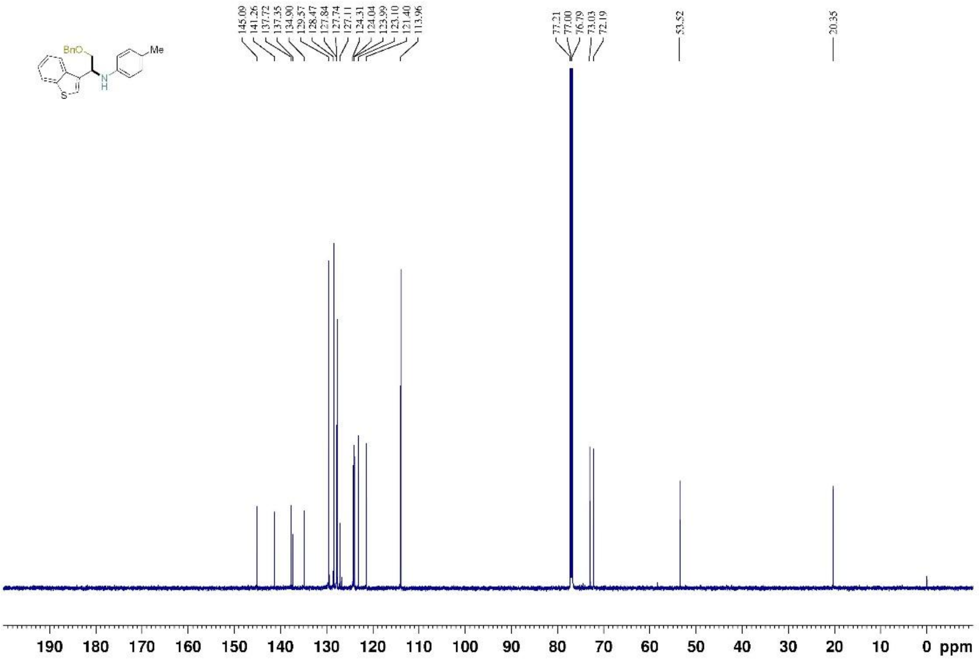

^{1}H NMR and ^{13}C NMR spectra of compound **6x**

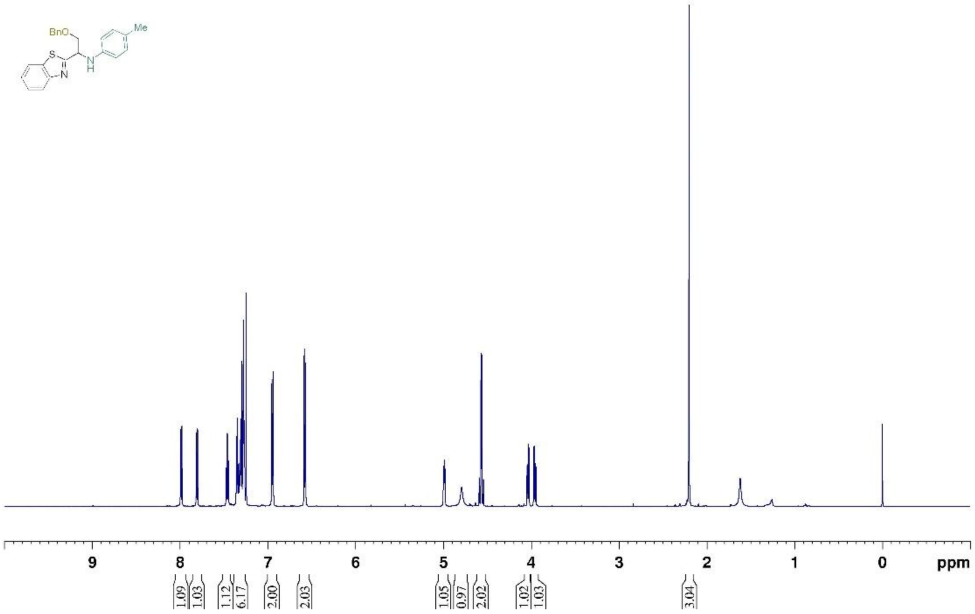

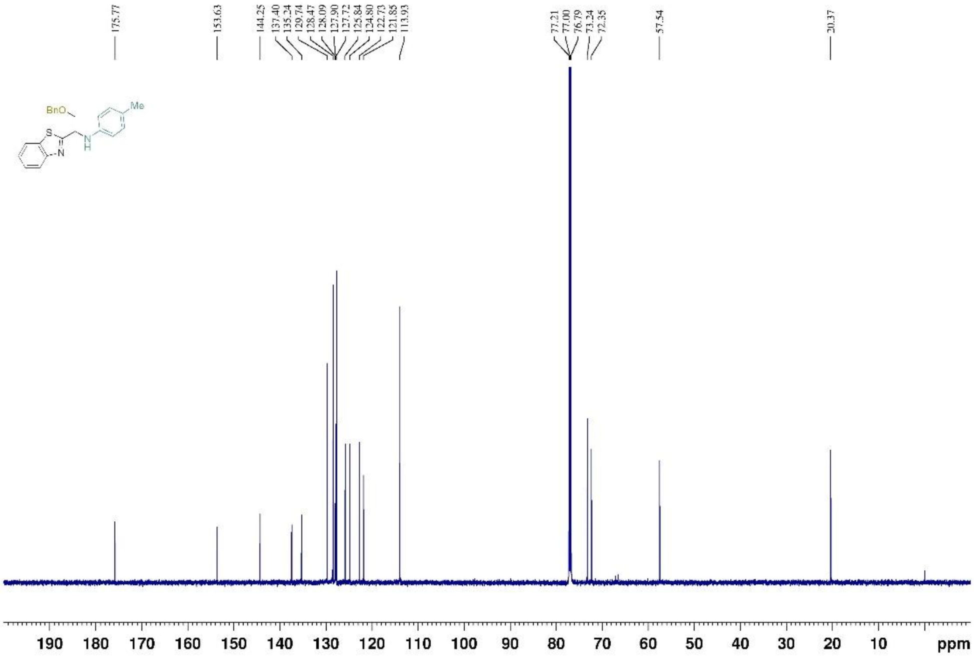

^{1}H NMR and ^{13}C NMR spectra of compound **6y**

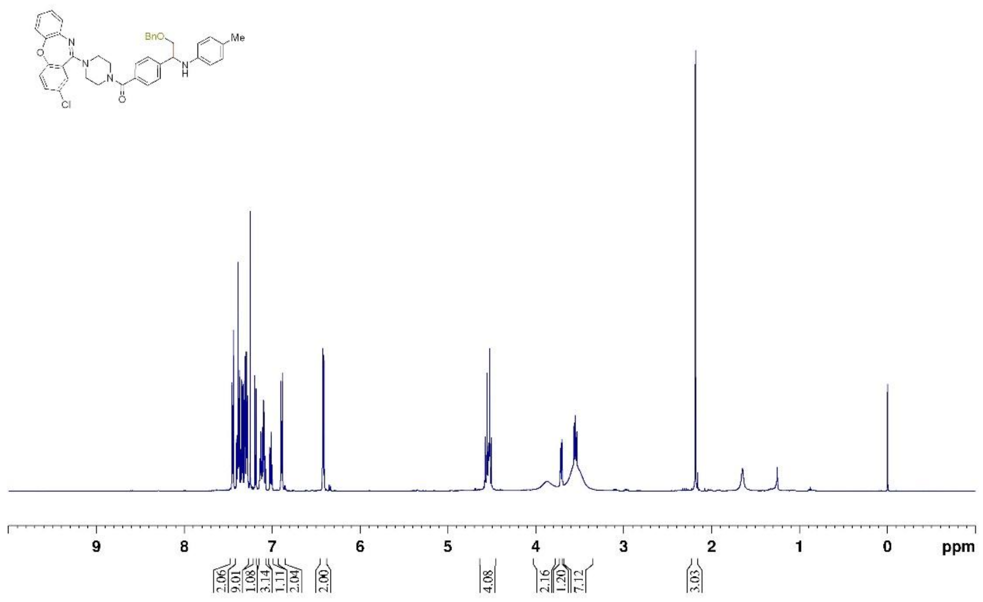

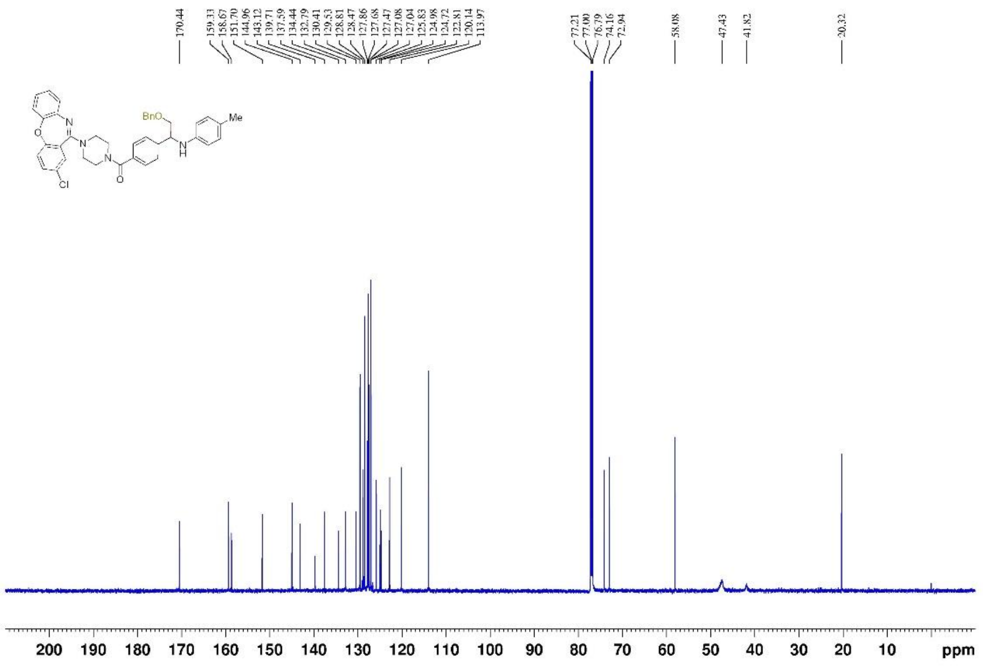

^{1}H NMR and ^{13}C NMR spectra of compound **6z**

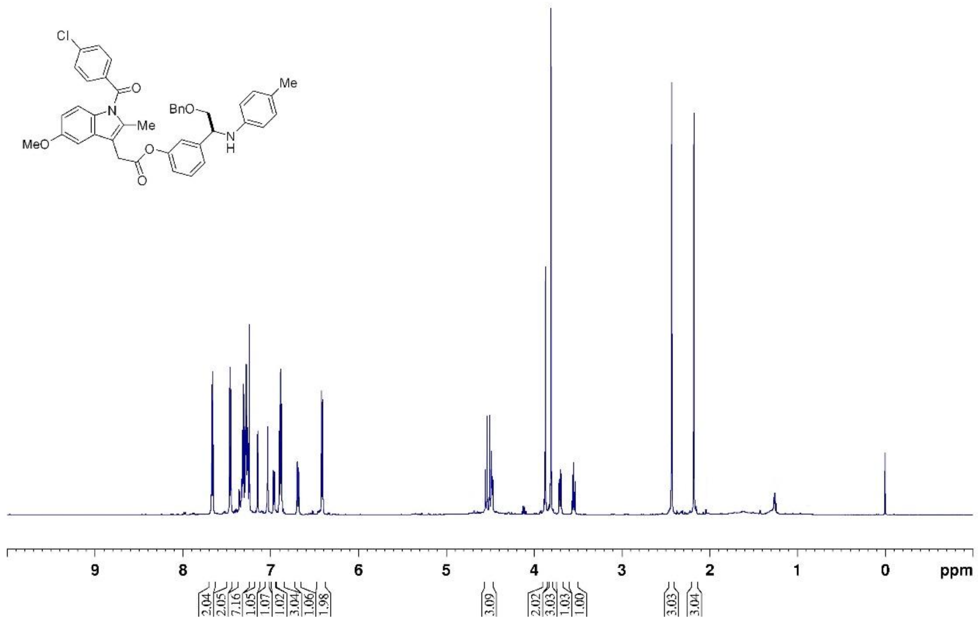

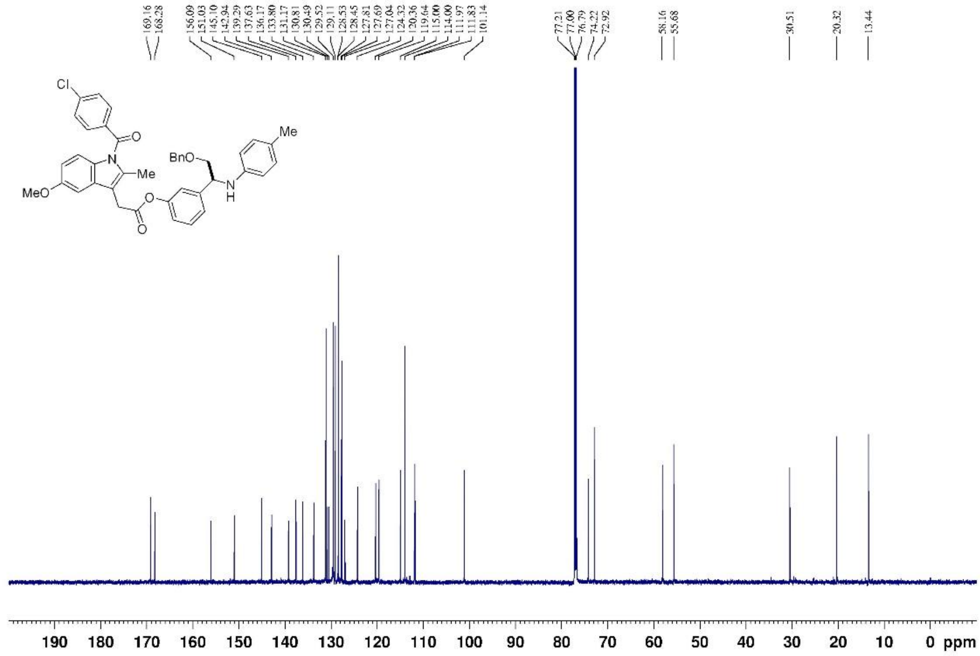

^{1}H NMR and ^{13}C NMR spectra of compound **6aa**

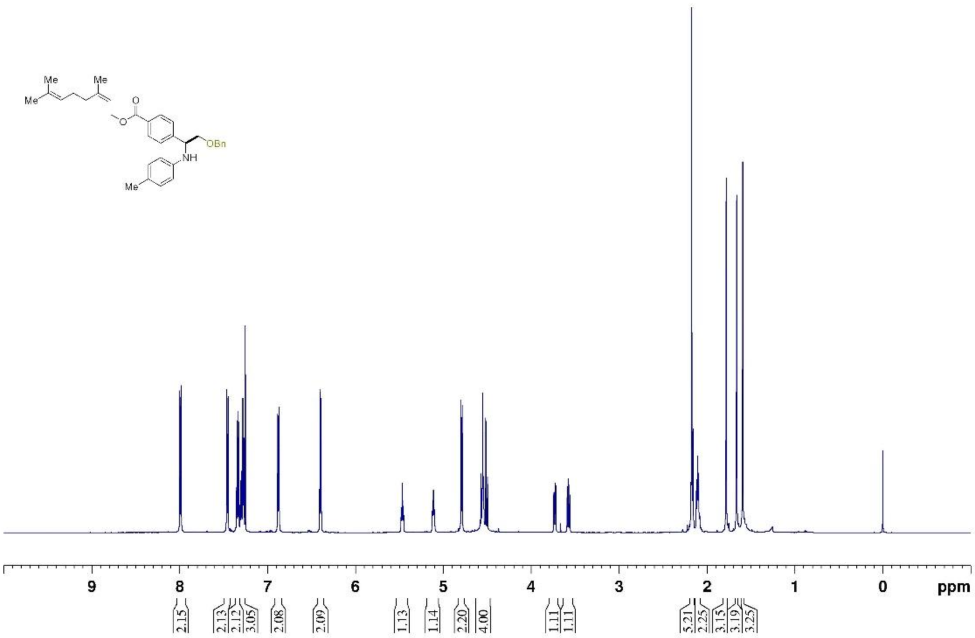

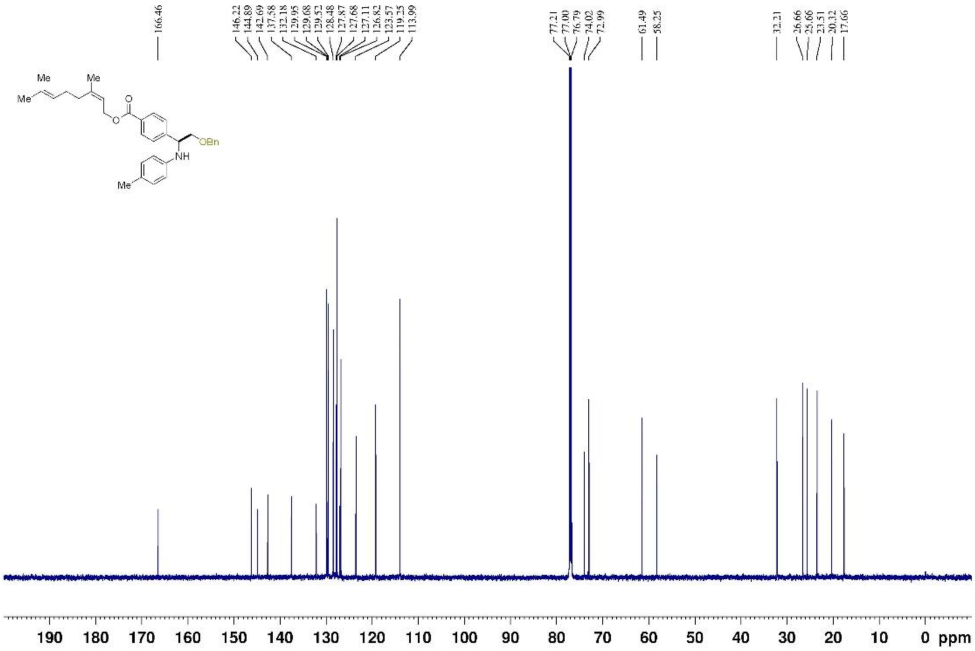

H NMR and ^{13}C NMR spectra of compound **6ab**

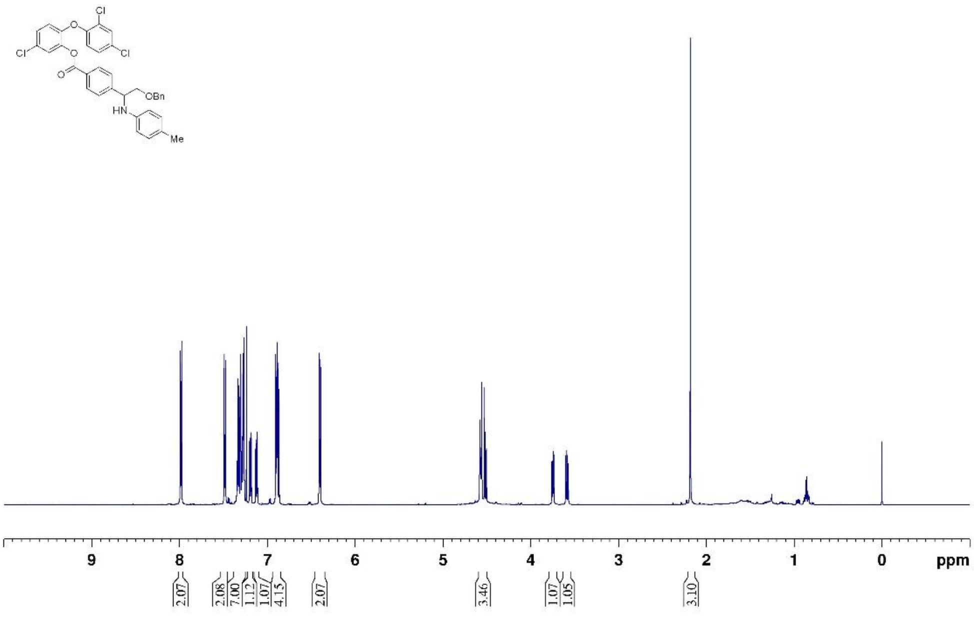

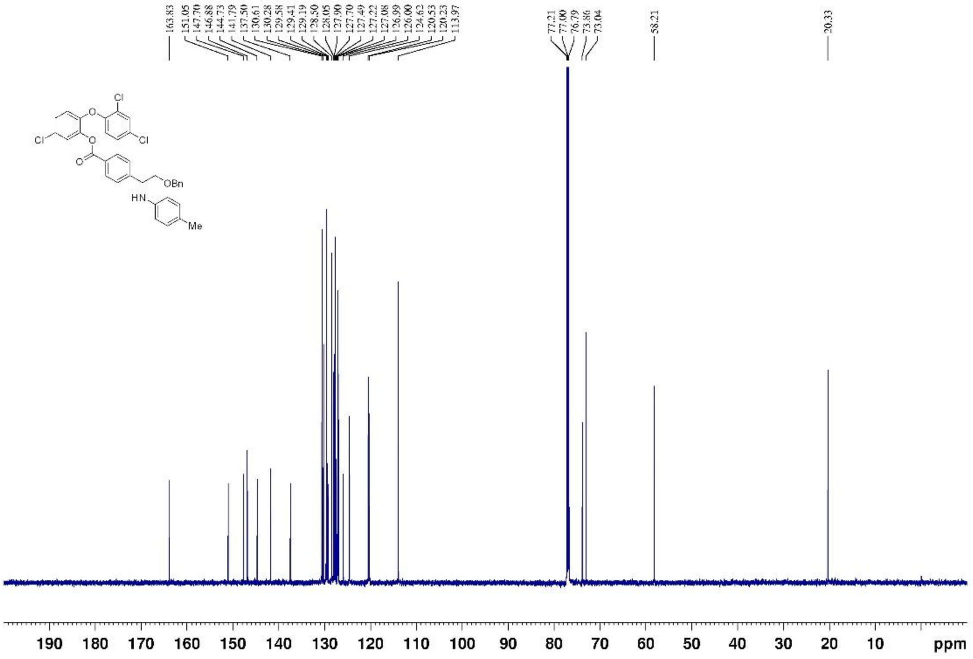

^{1}H NMR and ^{13}C NMR spectra of compound **6ac**

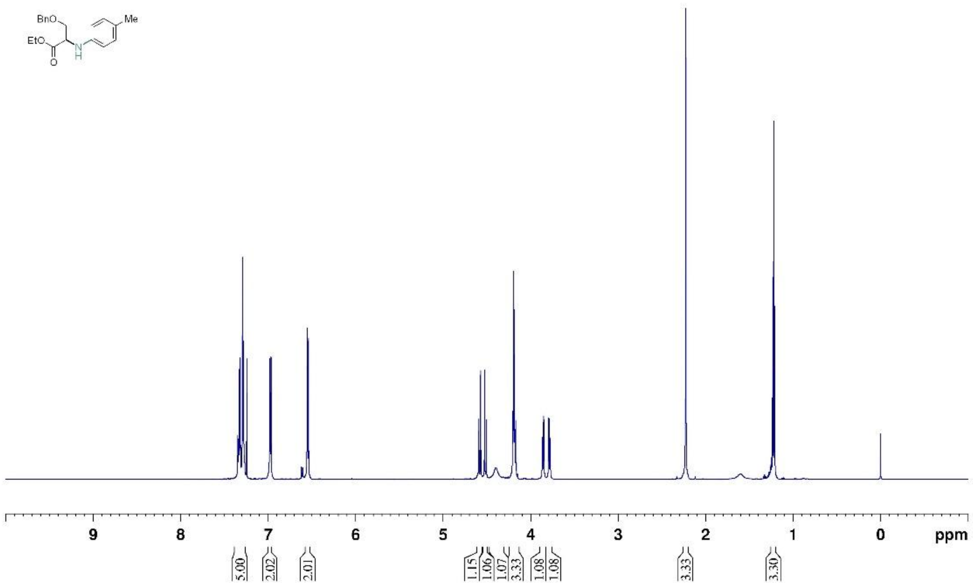

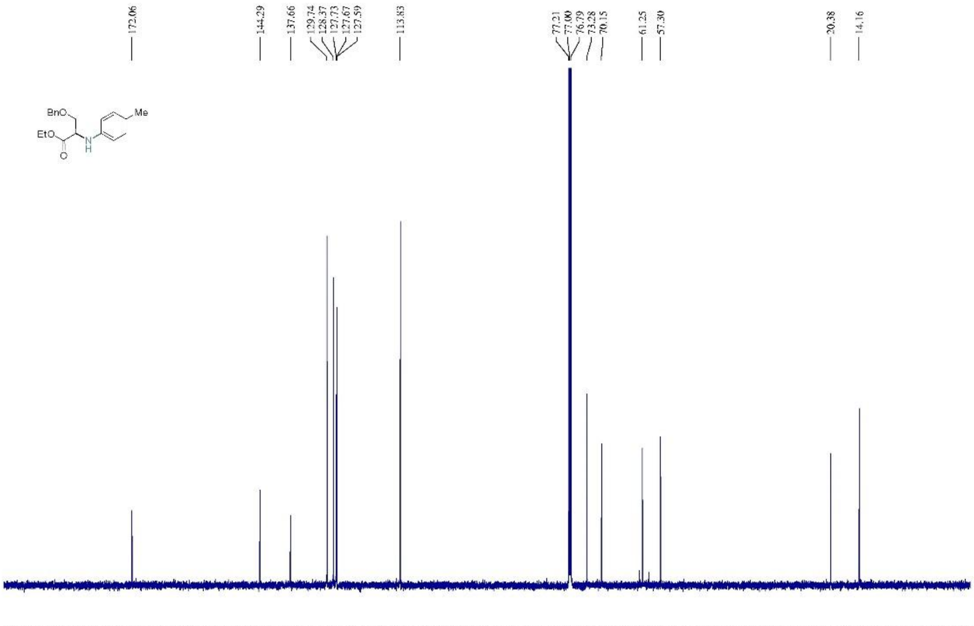

^{1}H NMR and ^{13}C NMR spectra of compound **6ad**

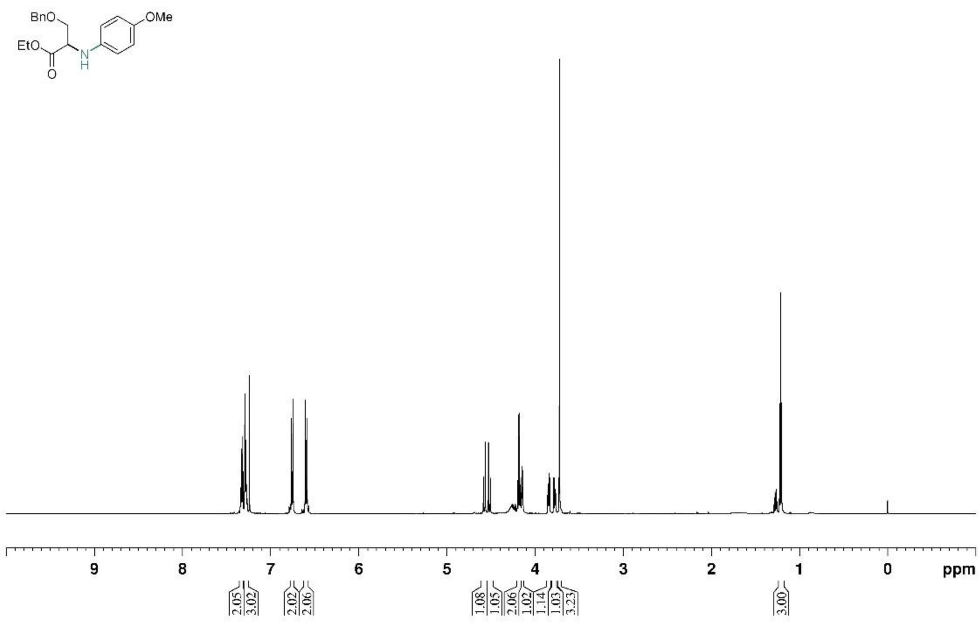

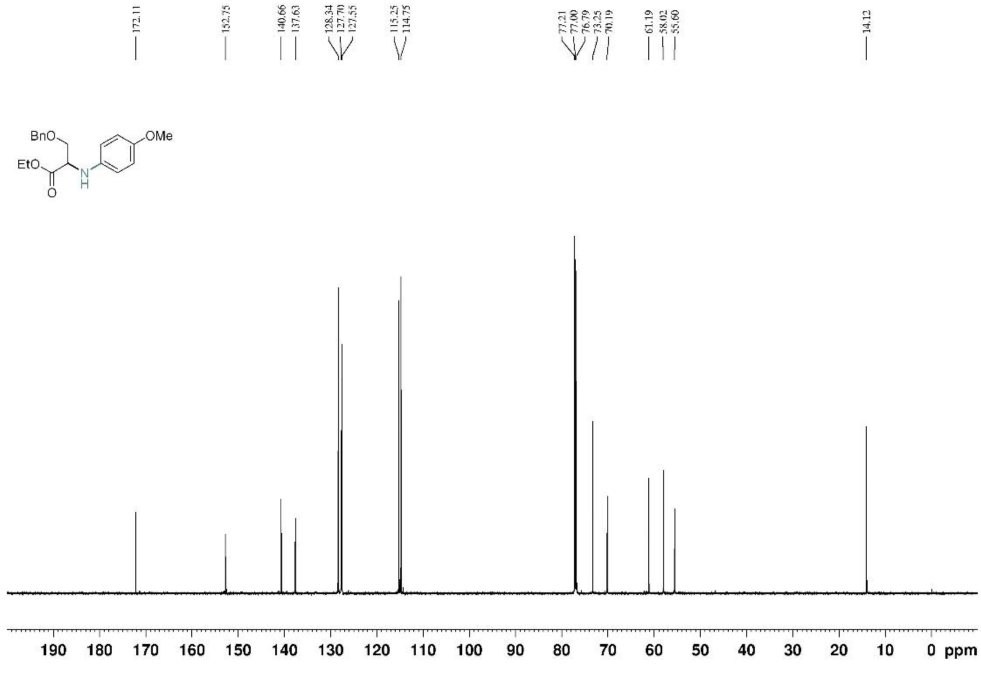

^{1}H NMR and ^{13}C NMR spectra of compound **6ae**

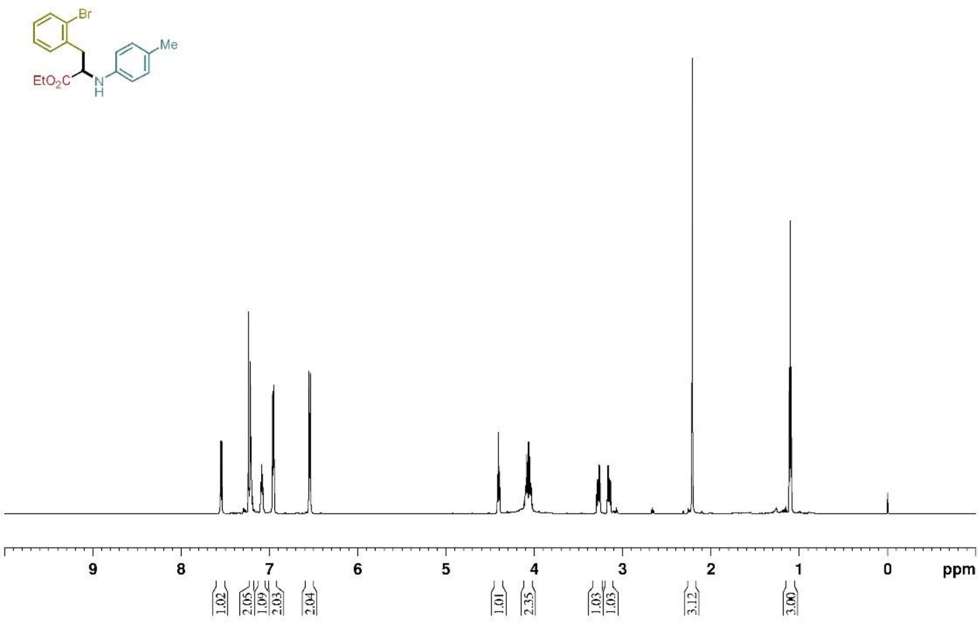

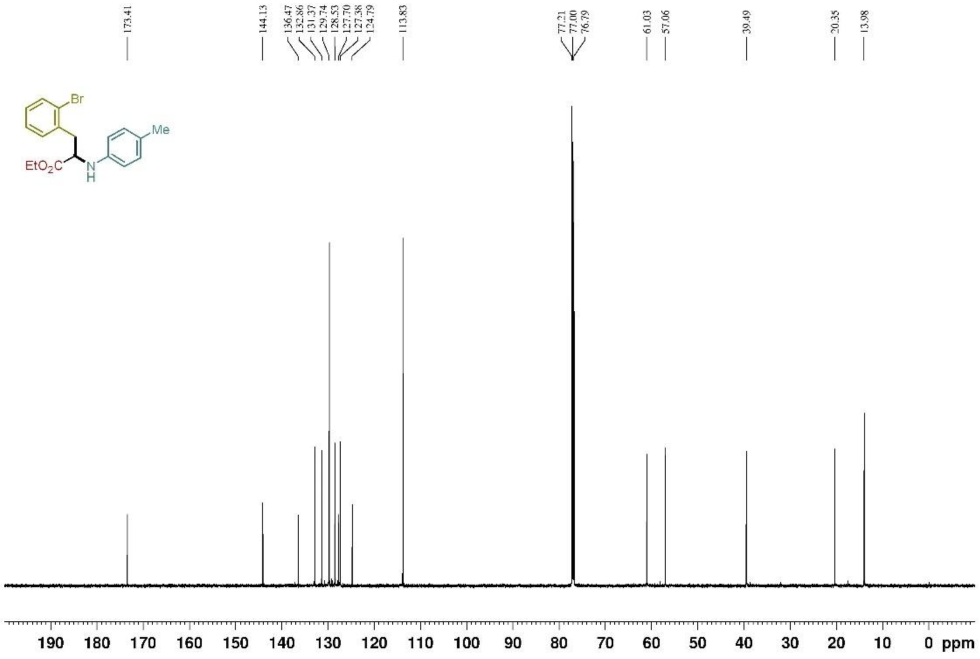

^{1}H NMR and ^{13}C NMR spectra of compound **6af**

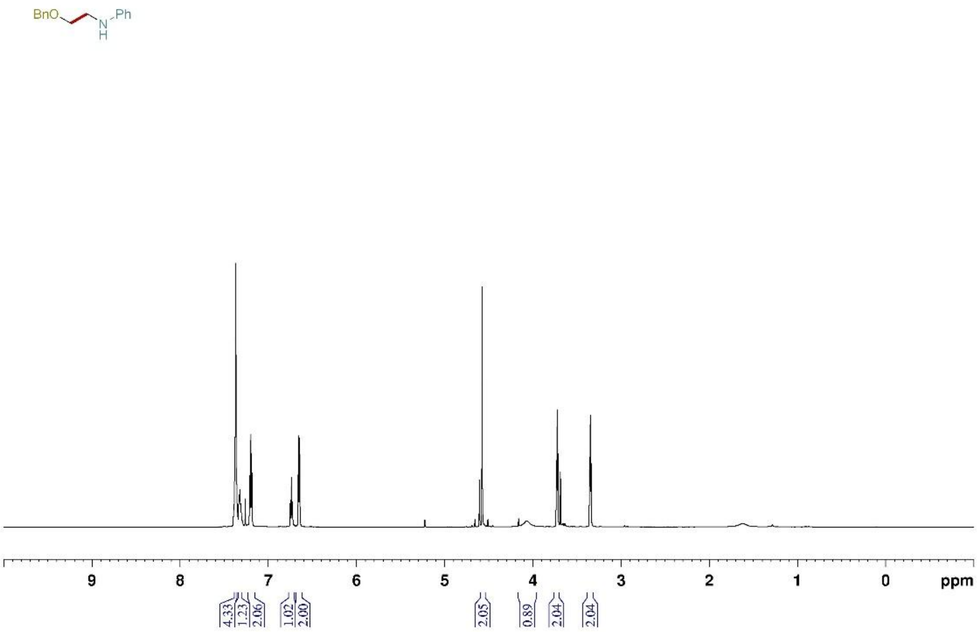

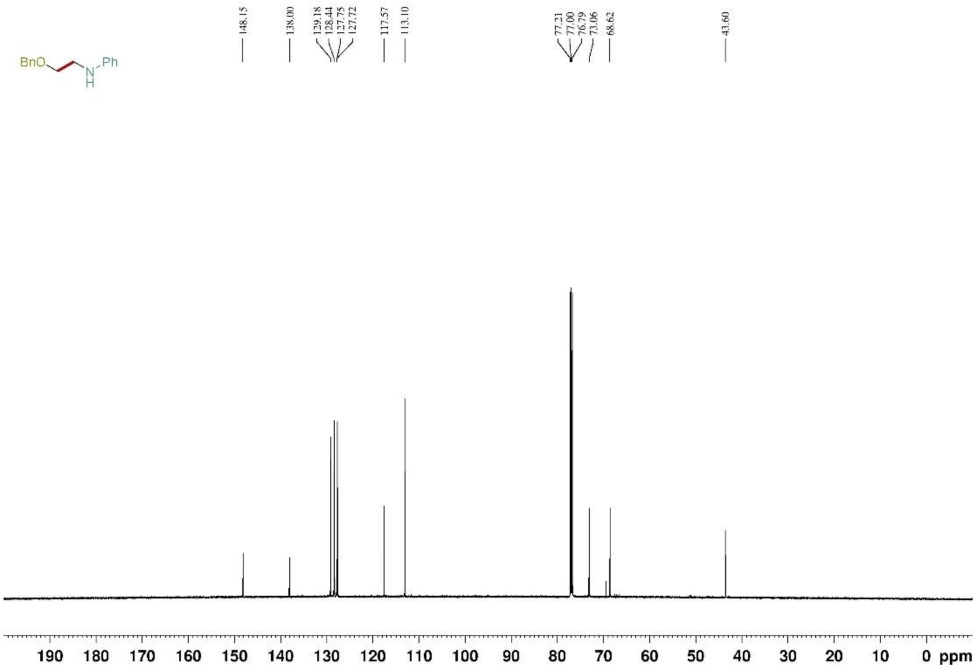

^{1}H NMR and ^{13}C NMR spectra of compound **6ag**

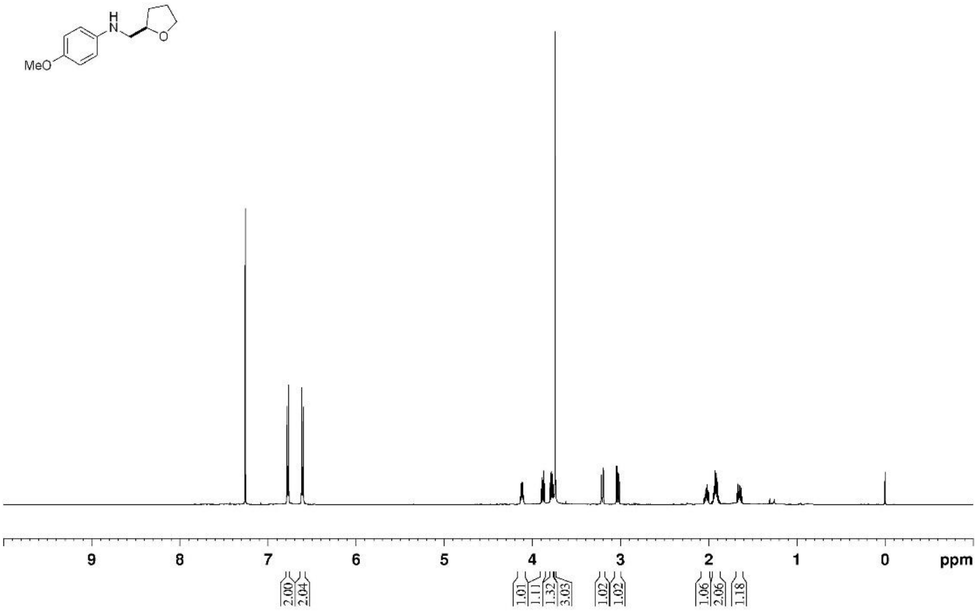

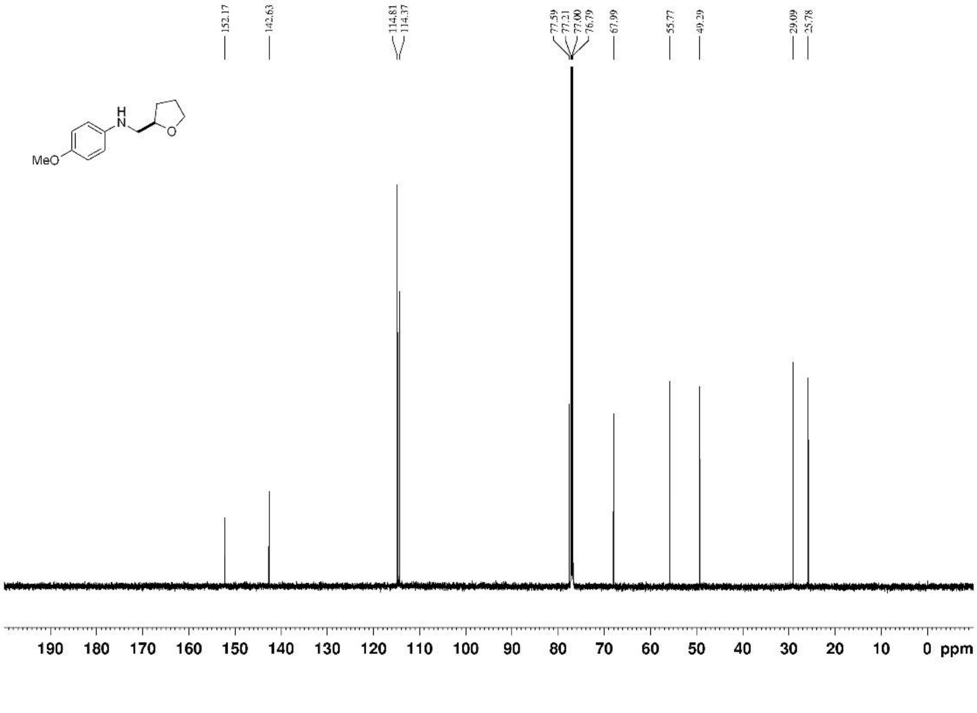

^{1}H NMR and ^{13}C NMR spectra of compound **6ah**

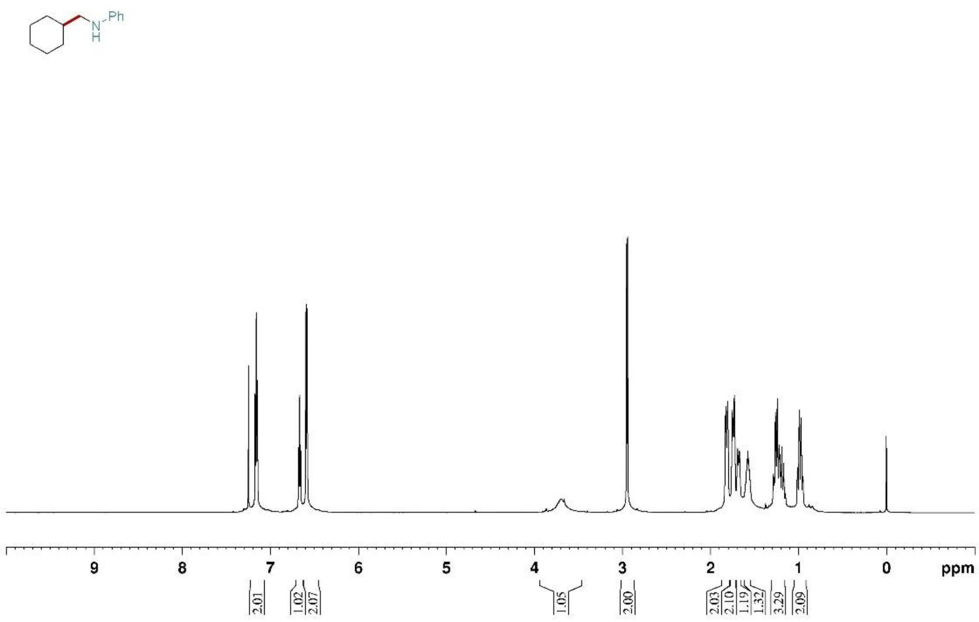

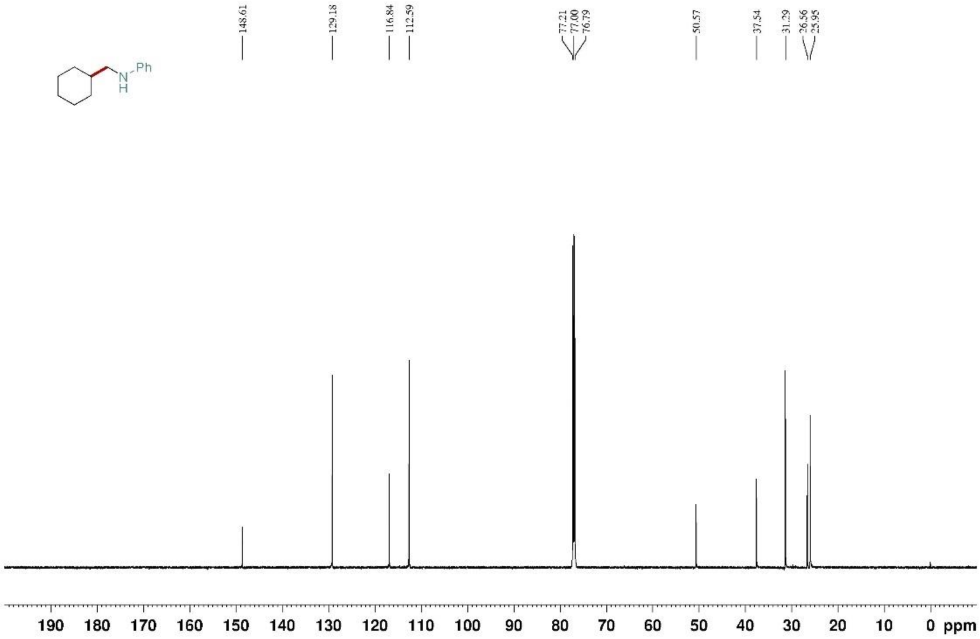

^{1}H NMR and ^{13}C NMR spectra of compound **6ai**

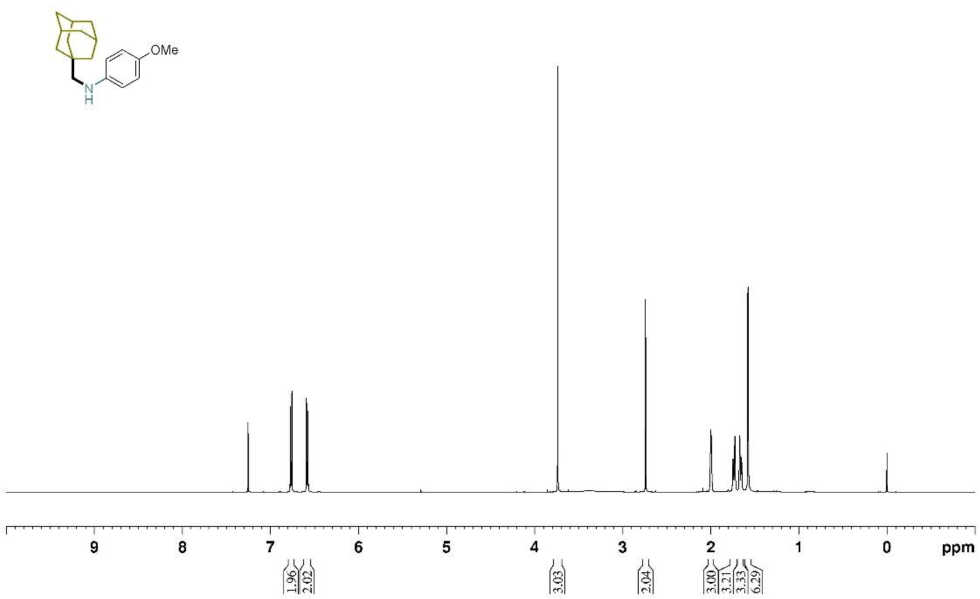

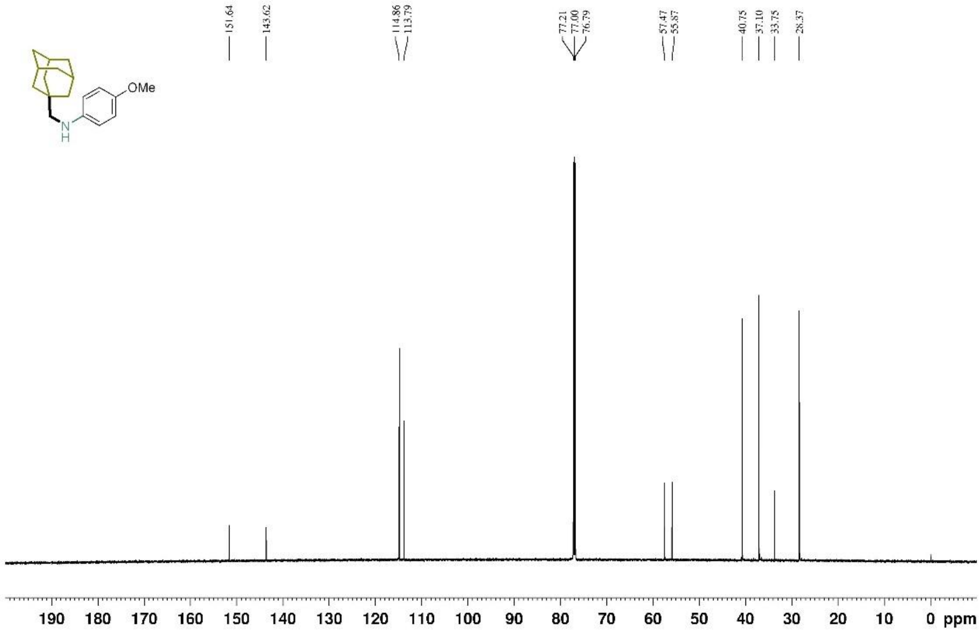

^{1}H NMR and ^{13}C NMR spectra of compound **6aj**

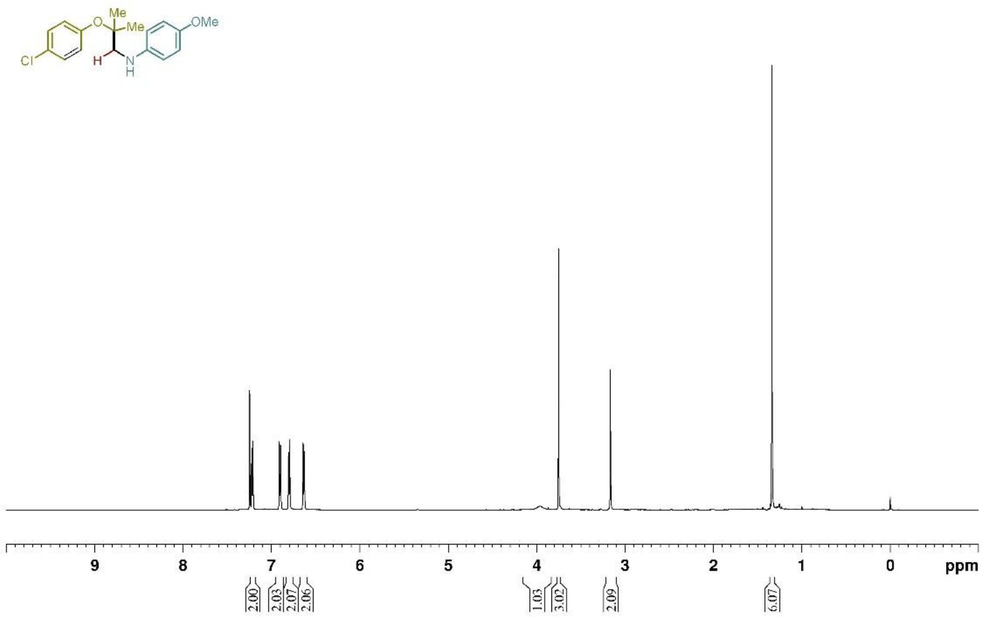

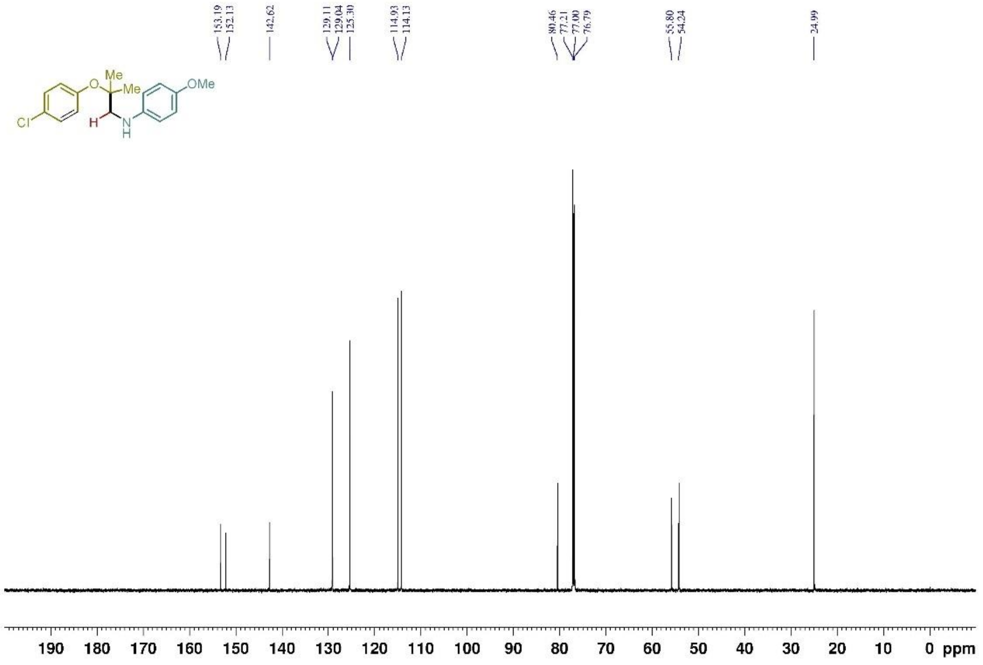

^{1}H NMR and ^{13}C NMR spectra of compound **6ak**

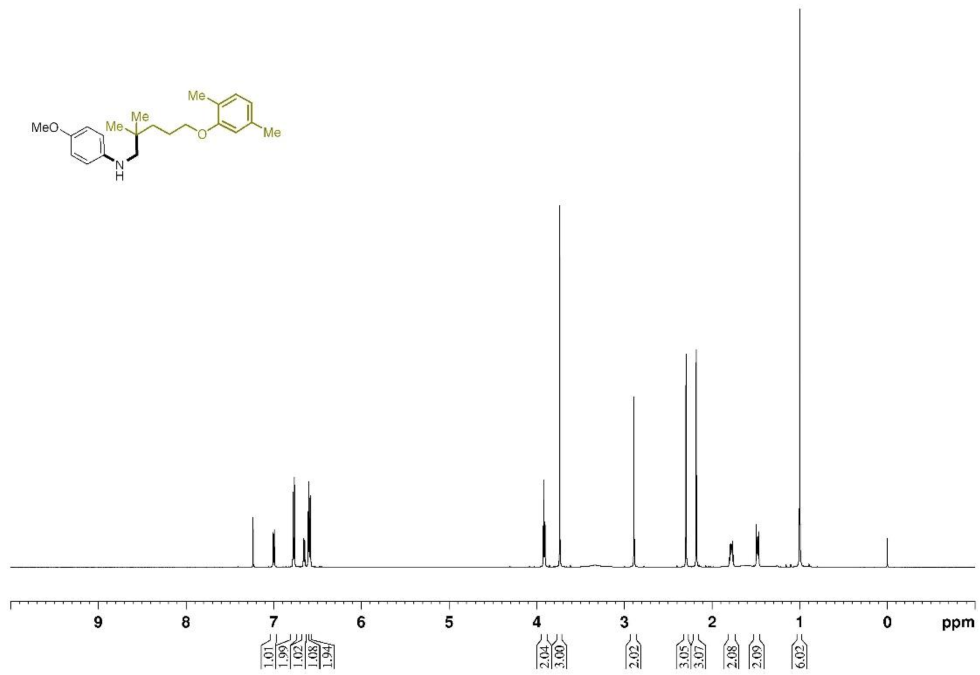

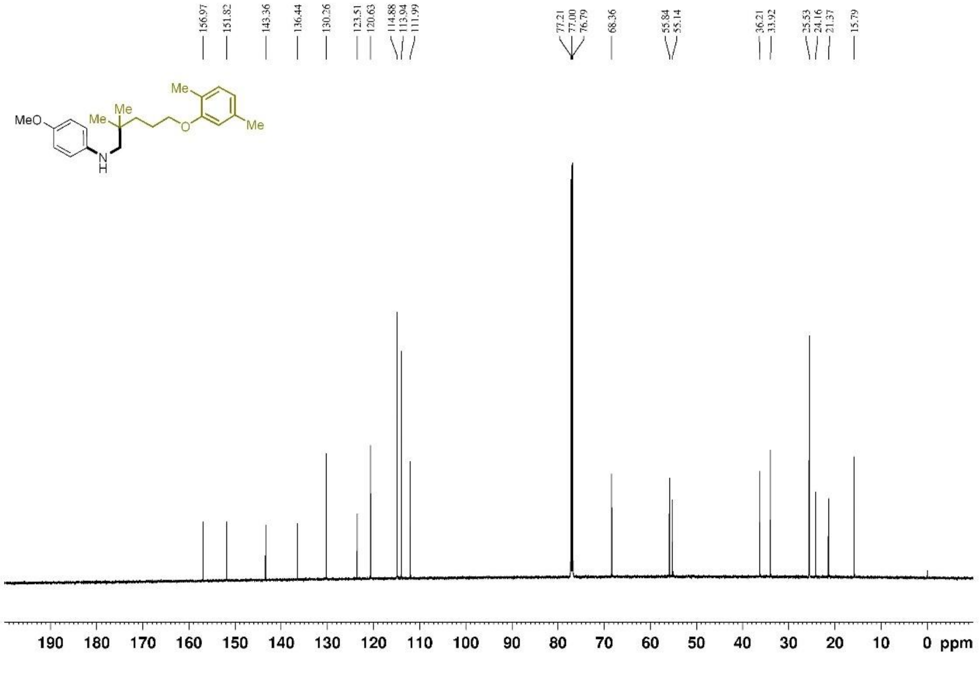

^{1}H NMR and ^{13}C NMR spectra of compound **6al**

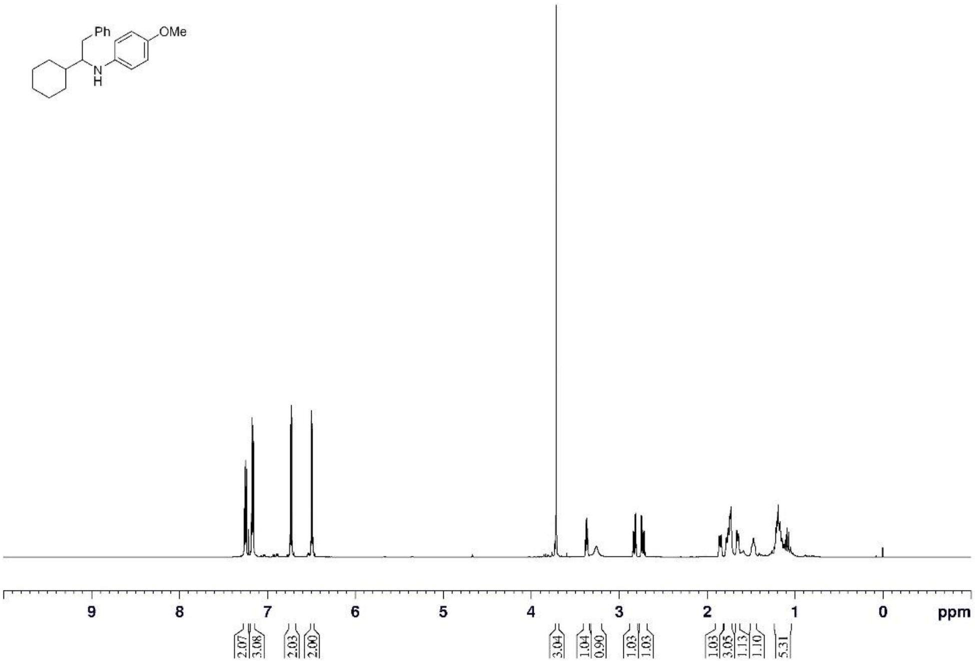

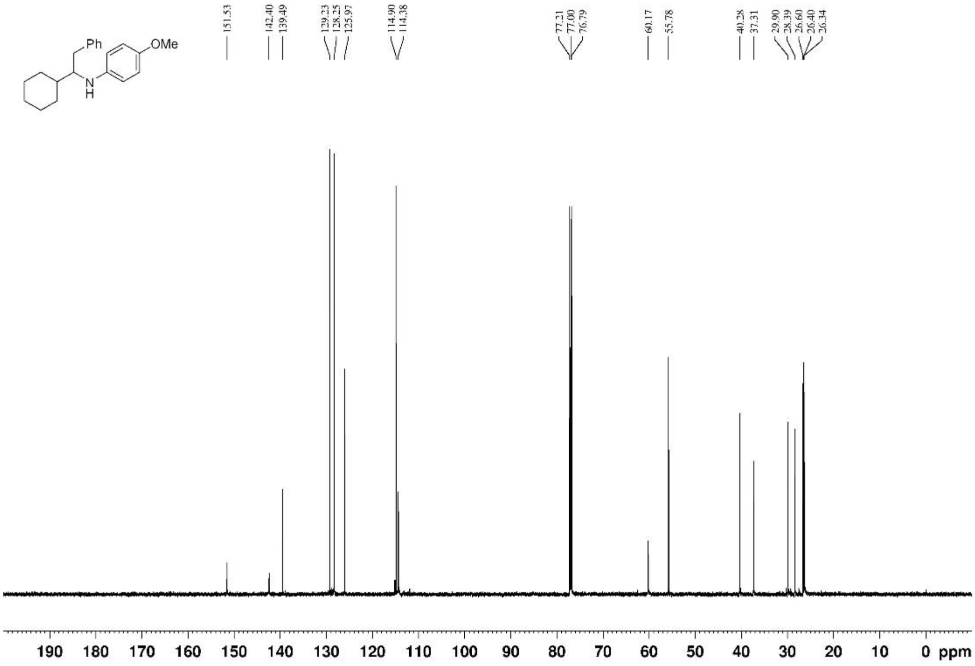

^{1}H NMR and ^{13}C NMR spectra of compound **6am**

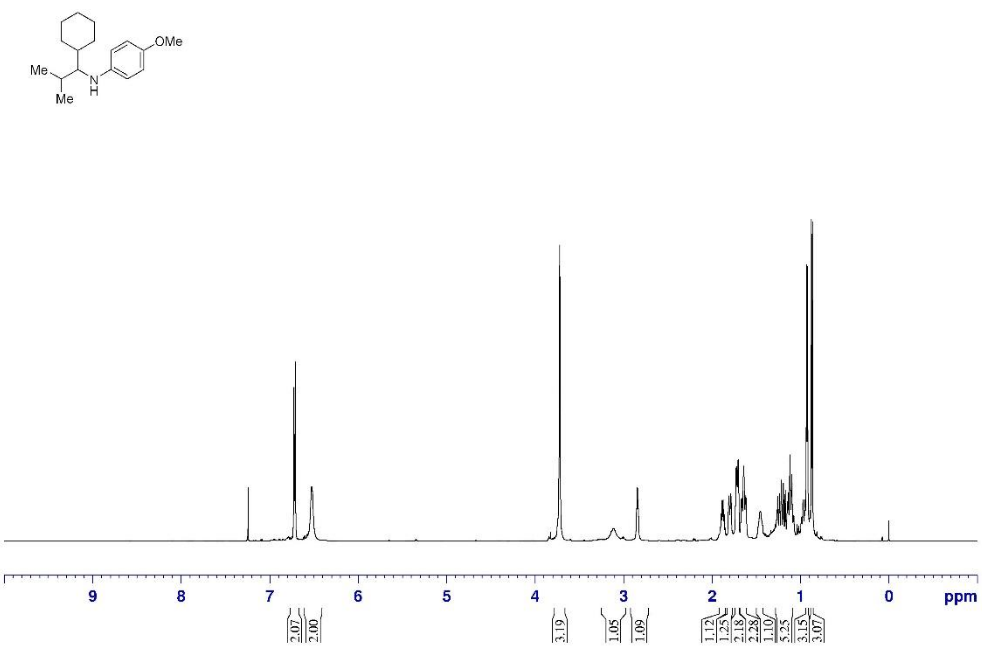

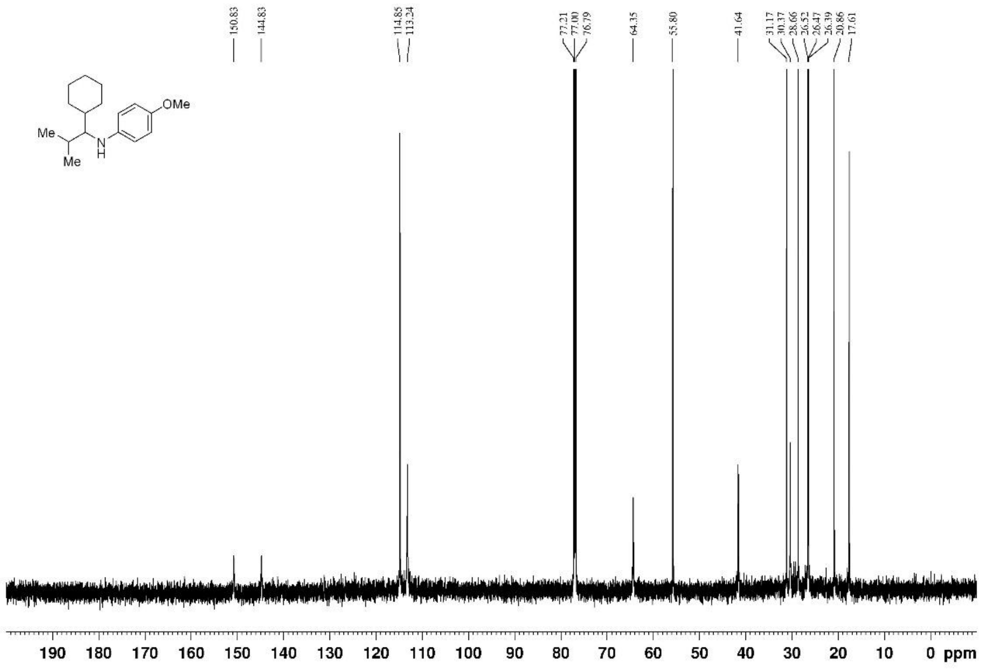

^{1}H NMR and ^{13}C NMR spectra of compound **6an**

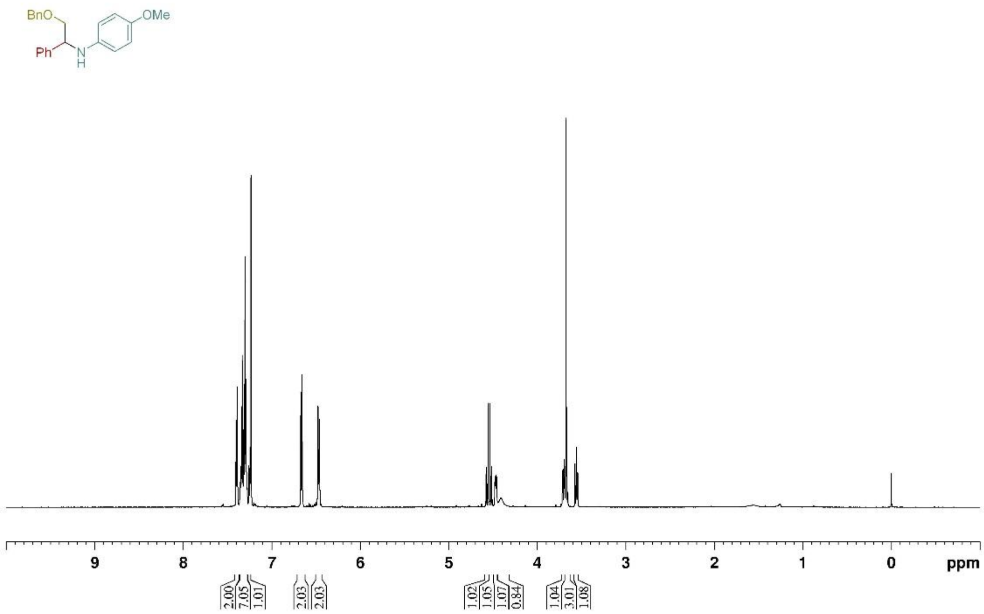

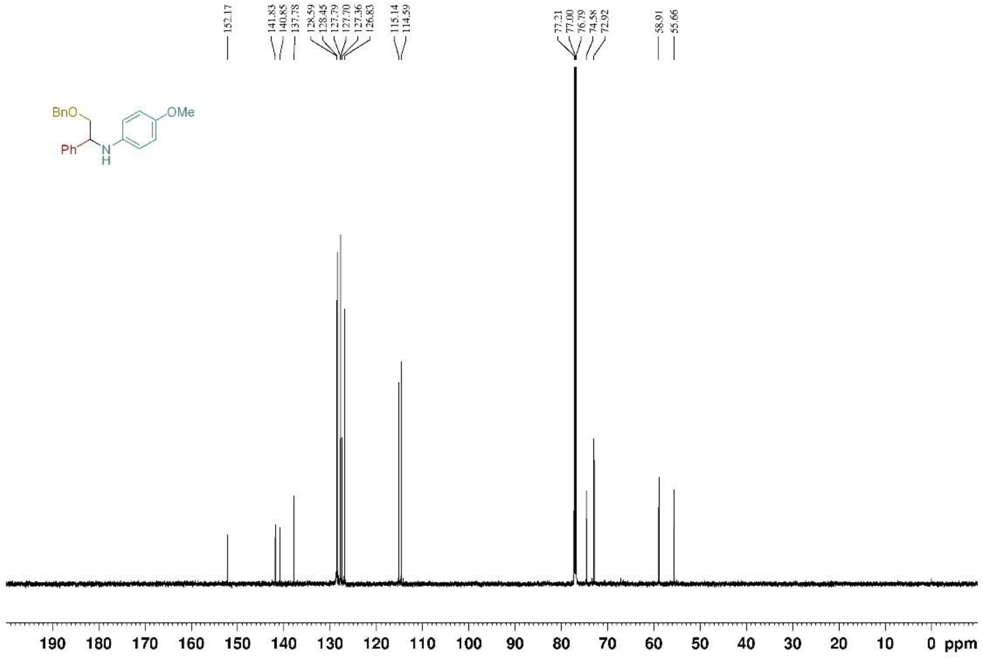

^{1}H NMR and ^{13}C NMR spectra of compound **6ao**

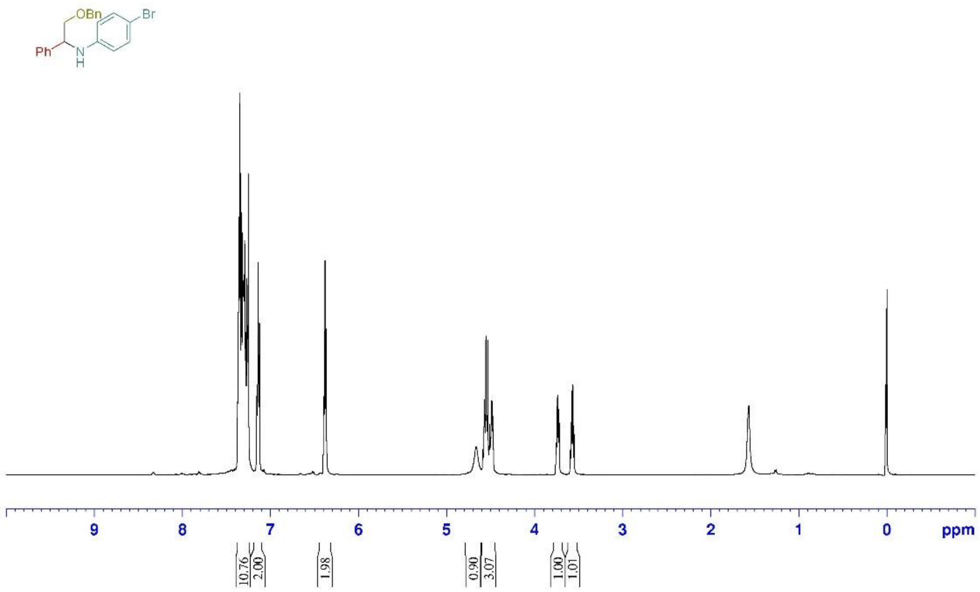

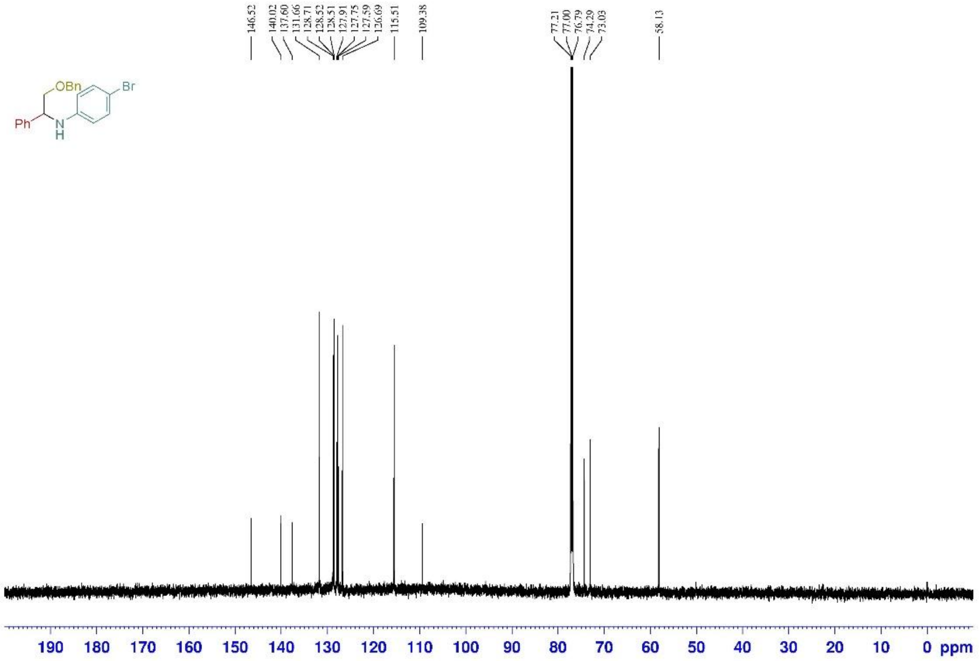

^{1}H NMR and ^{13}C NMR spectra of compound **6ap**

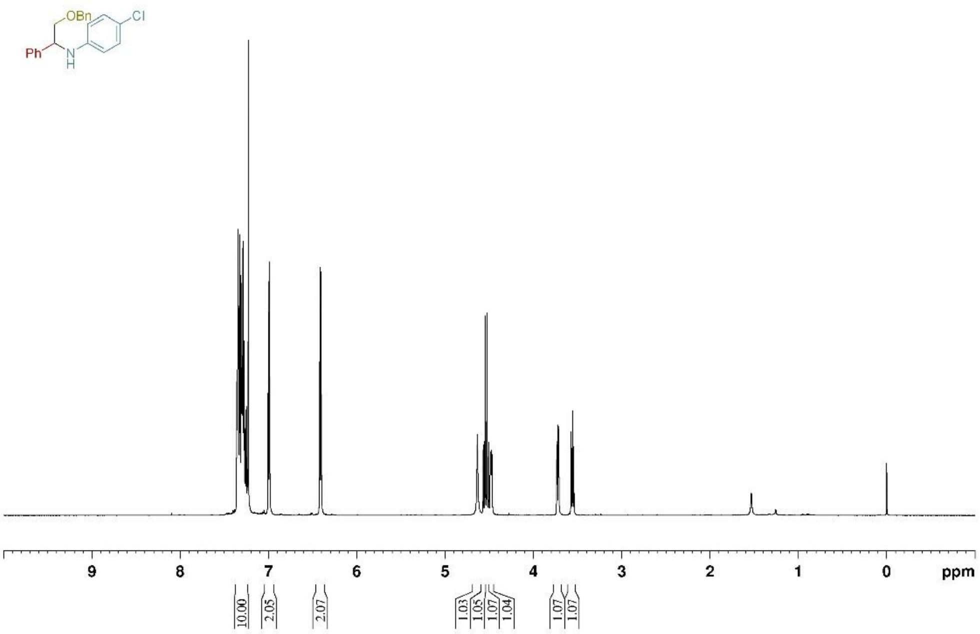

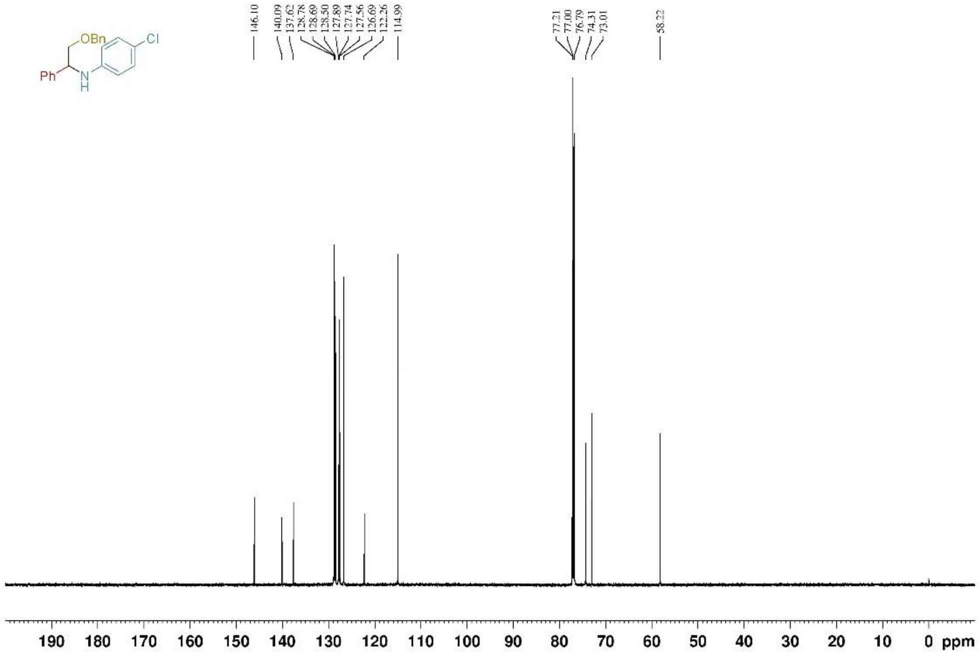

^{1}H NMR and ^{13}C NMR spectra of compound **6aq**

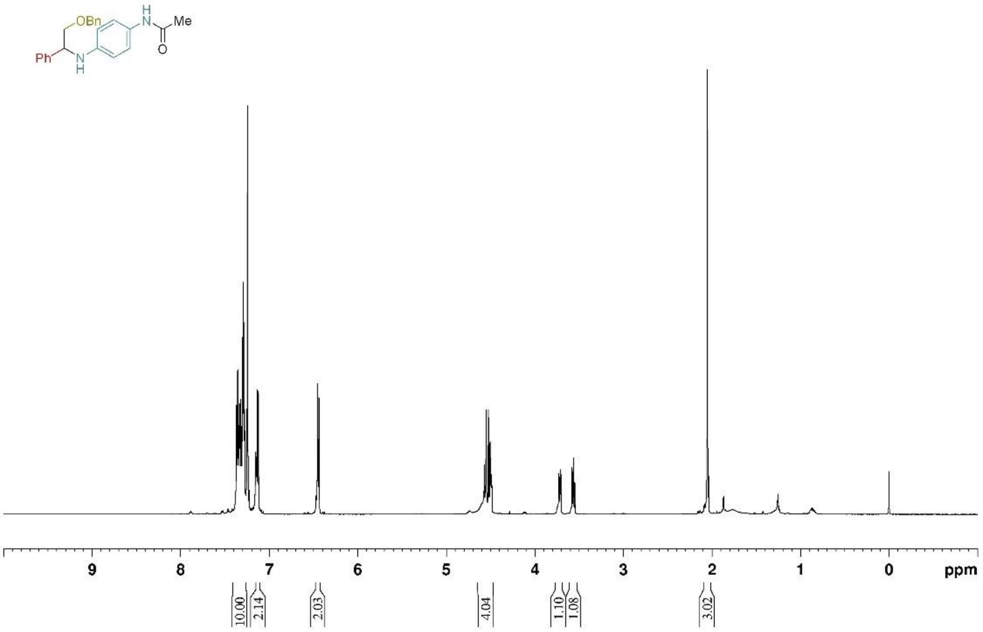

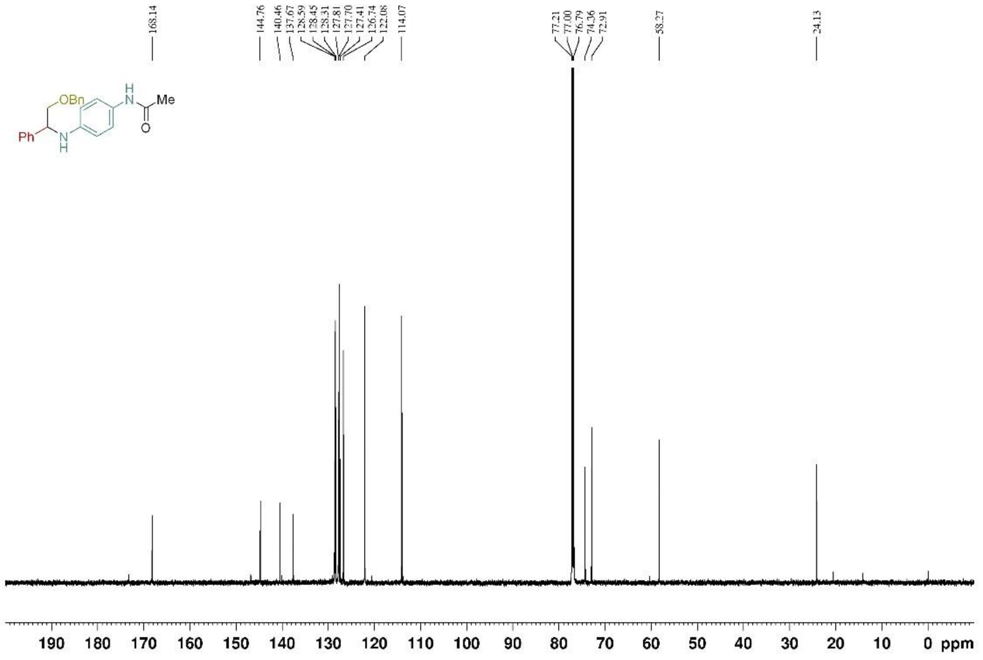

^{1}H NMR and ^{13}C NMR spectra of compound **6ar**

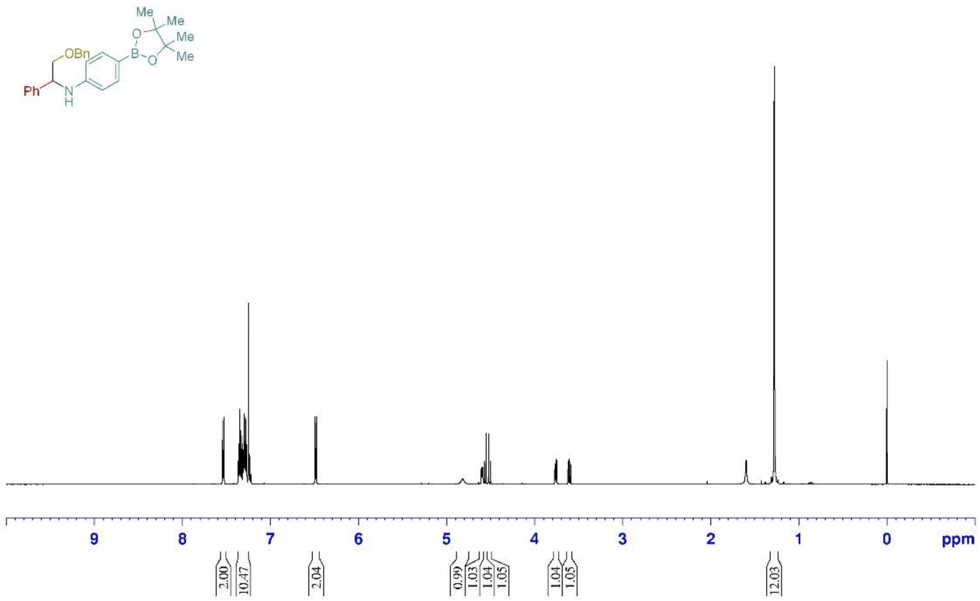

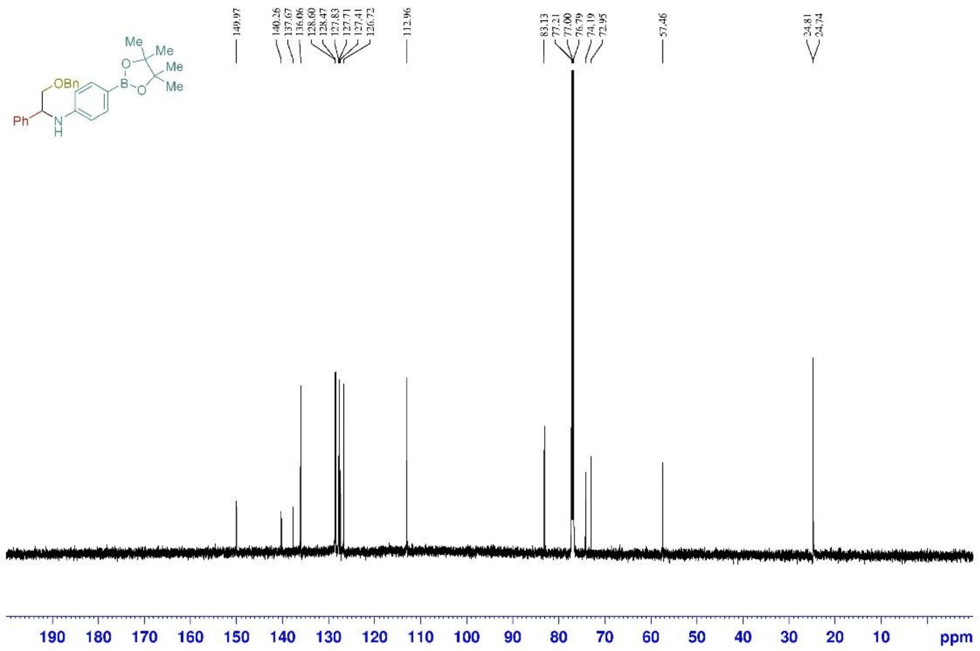

^{1}H NMR and ^{13}C NMR spectra of compound **6as**

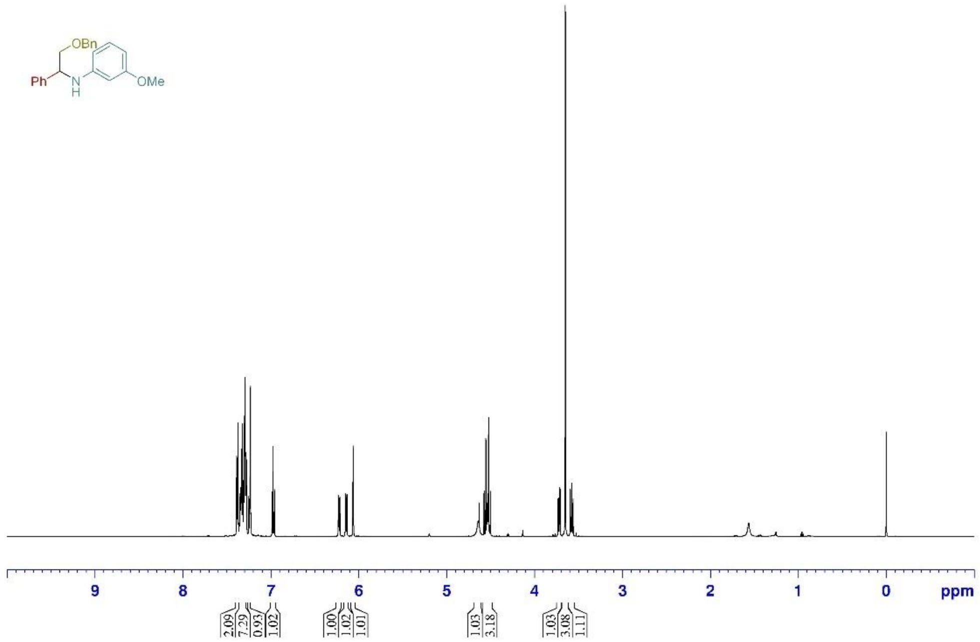

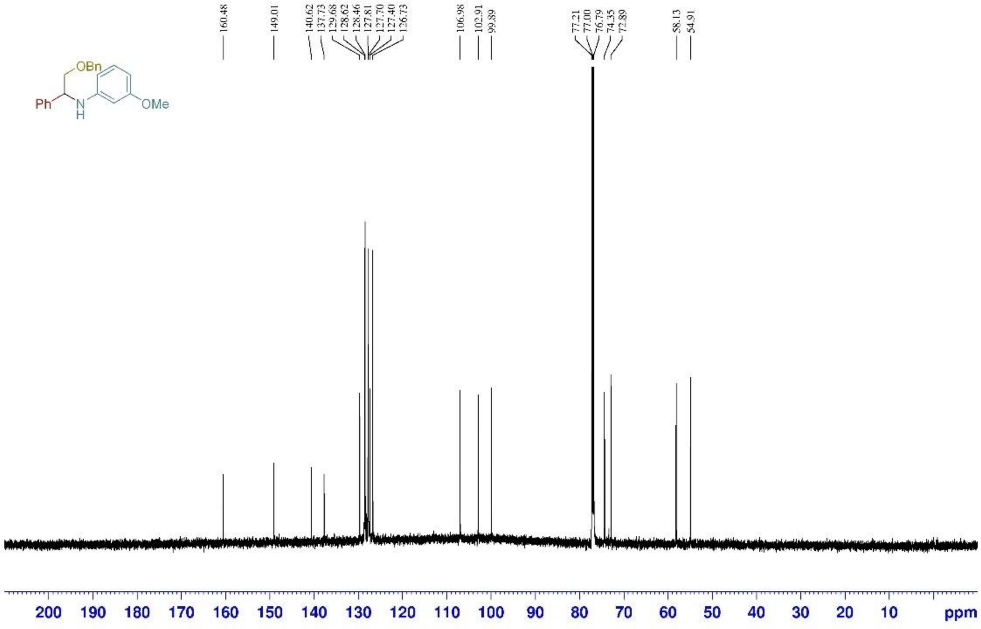

^{1}H NMR and ^{13}C NMR spectra of compound **6at**

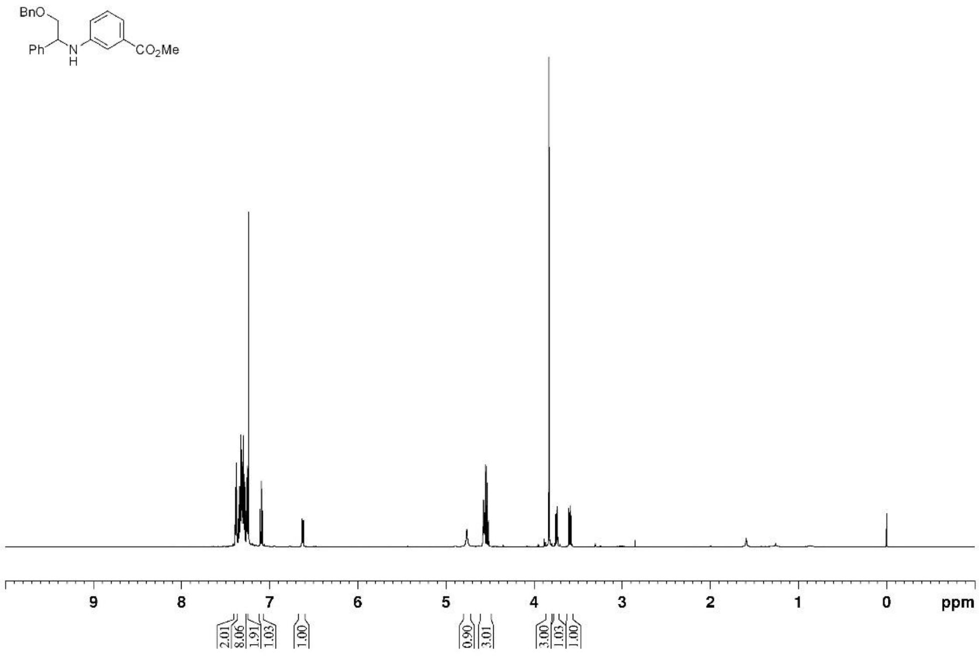

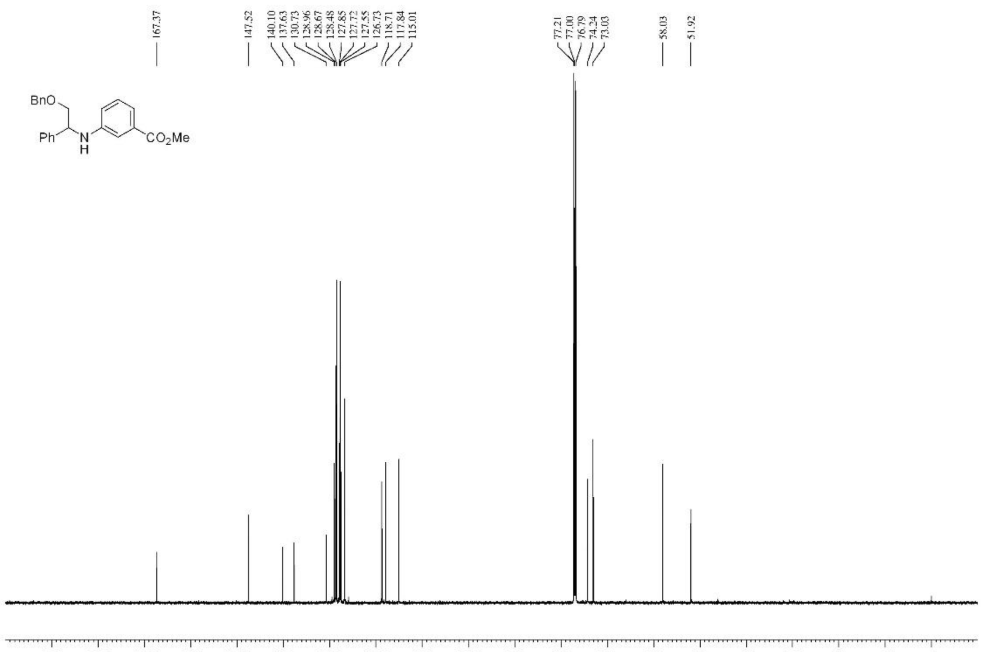

^{1}H NMR and ^{13}C NMR spectra of compound **6au**

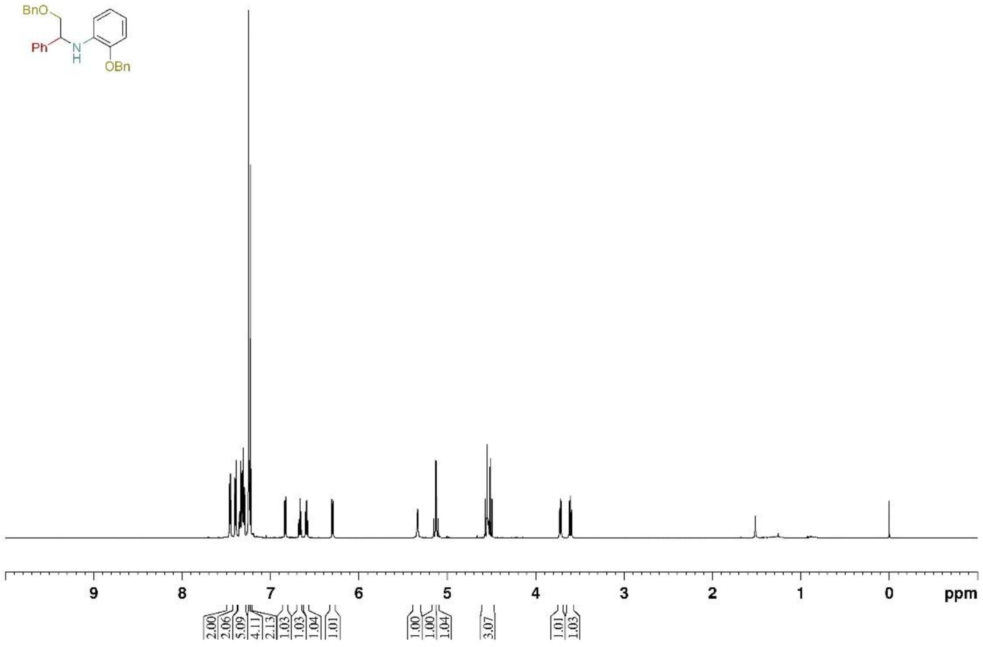

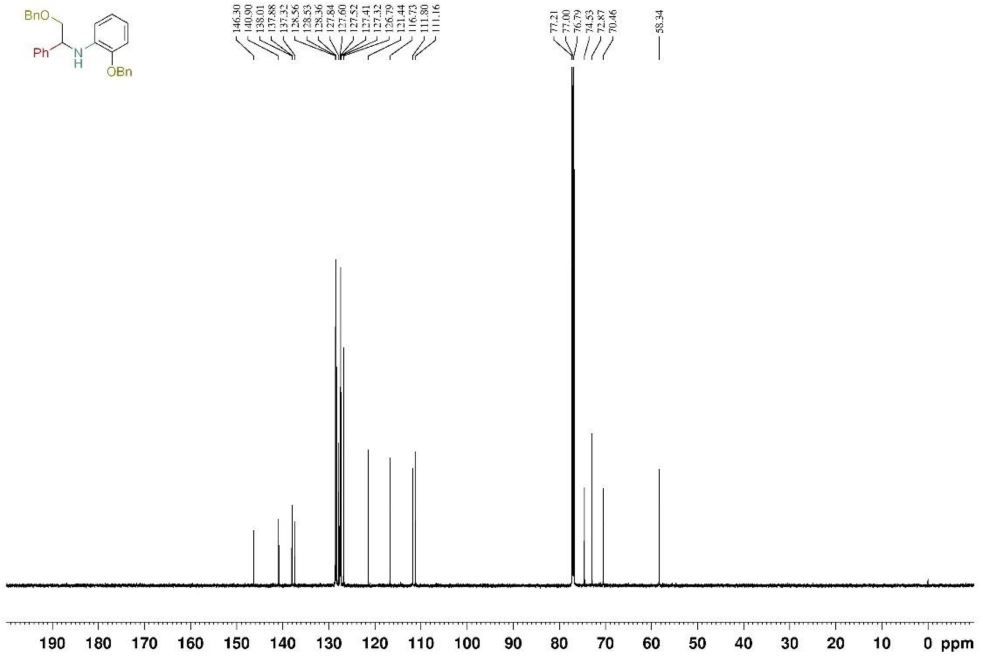

^{1}H NMR and ^{13}C NMR spectra of compound **6av**

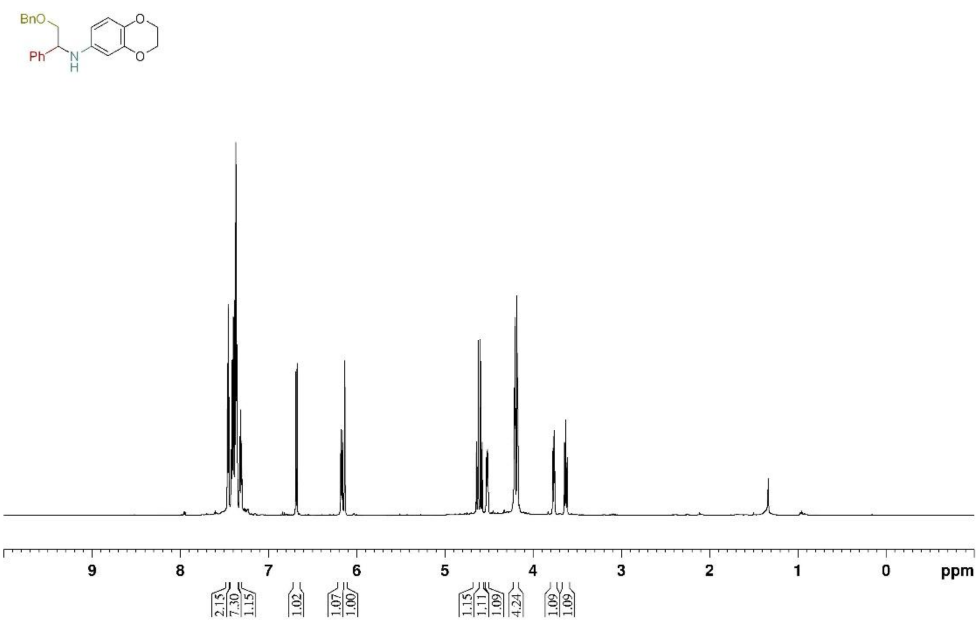

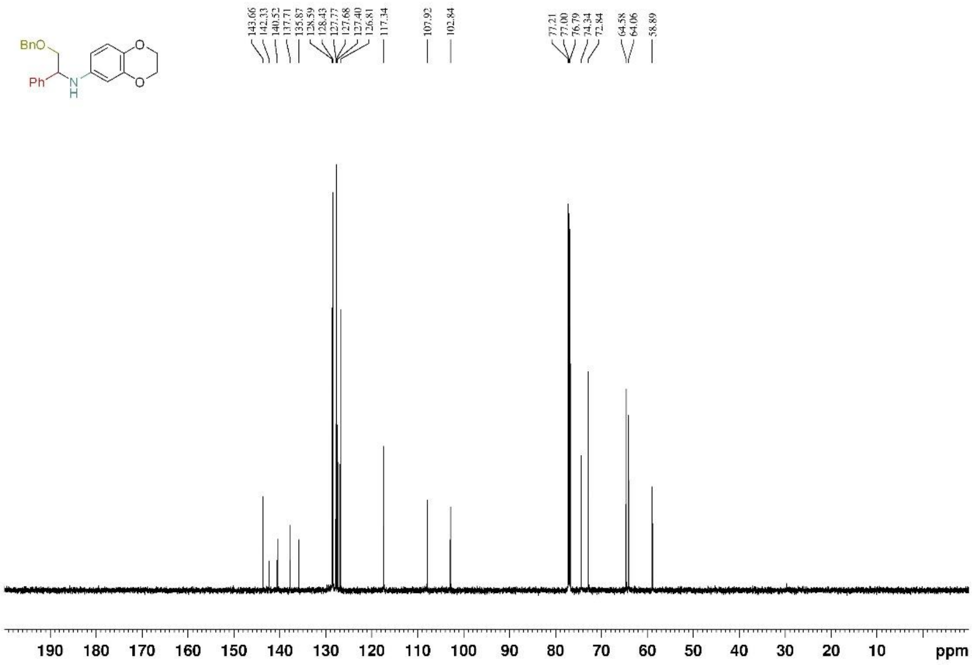

^{1}H NMR and ^{13}C NMR spectra of compound **6aw**

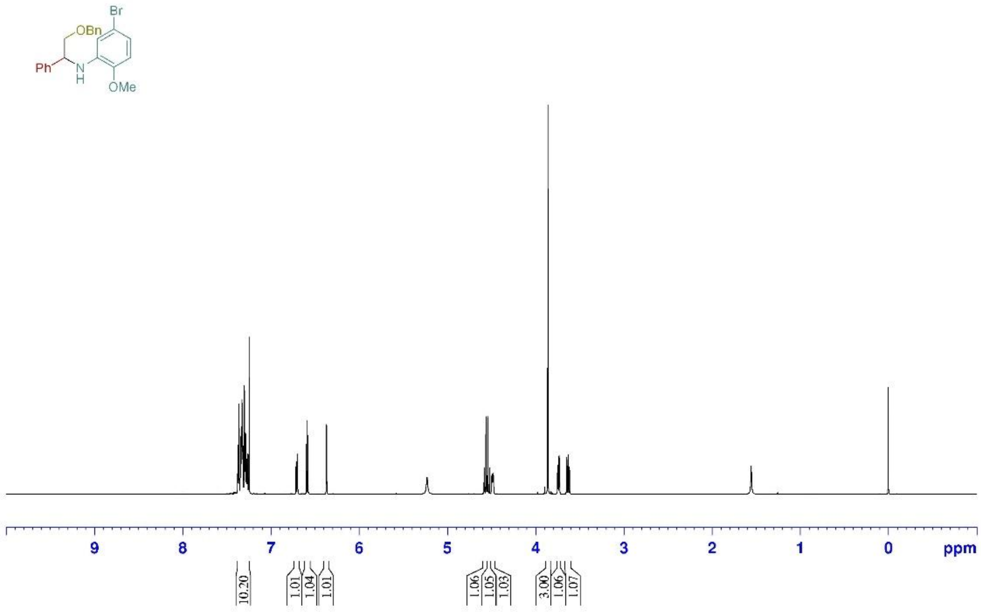

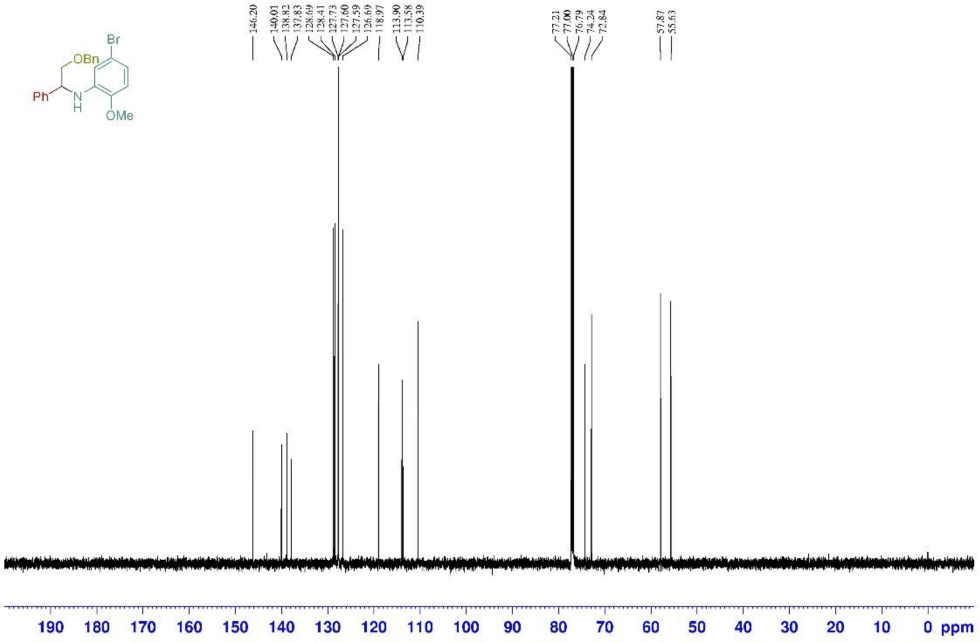

^{1}H NMR and ^{13}C NMR spectra of compound **6ax**

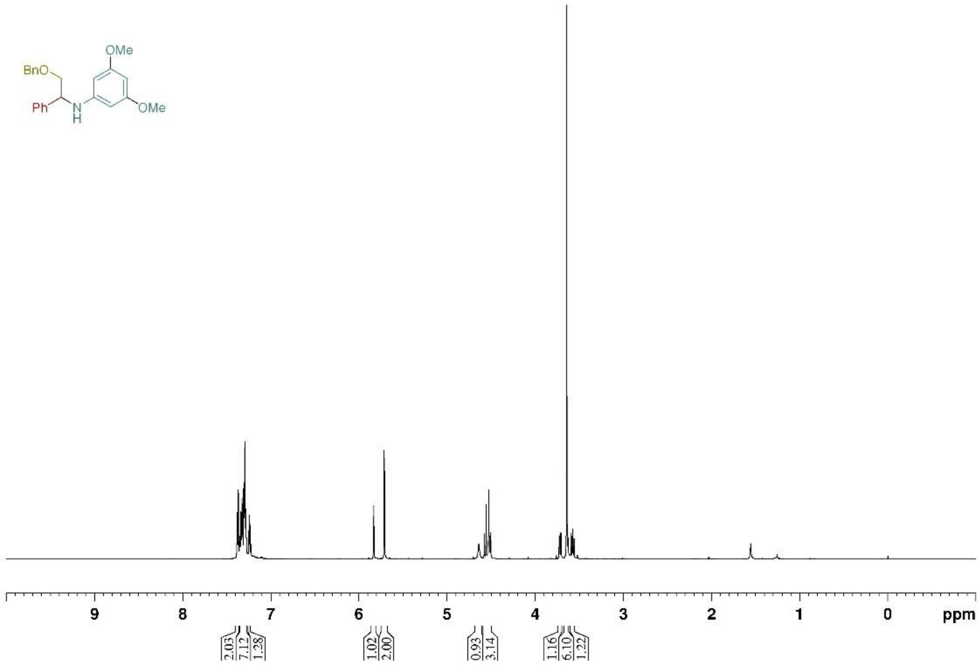

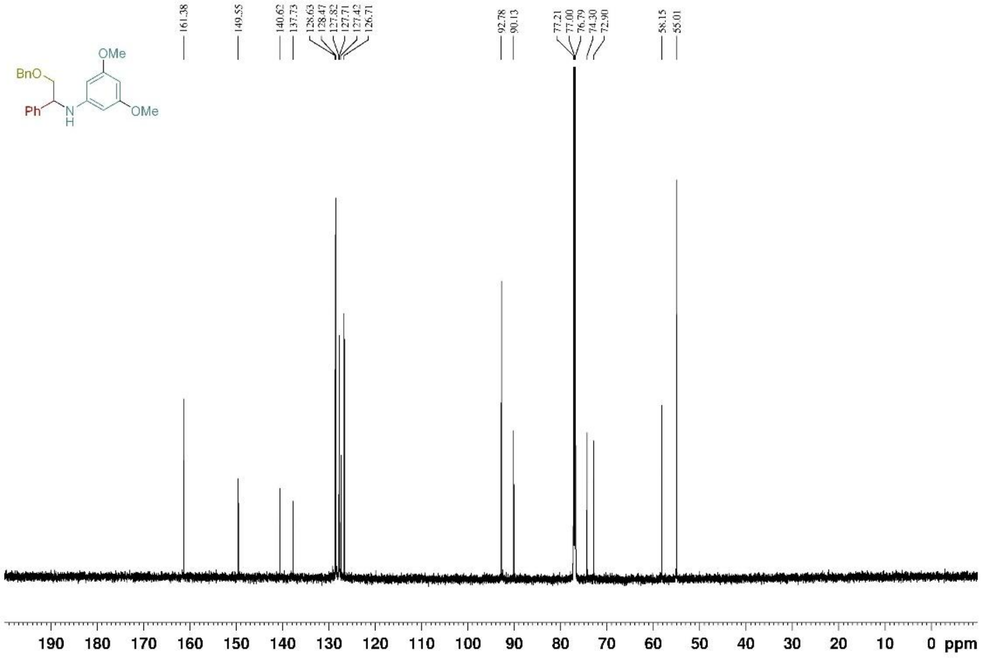

^{1}H NMR and ^{13}C NMR spectra of compound **6ay**

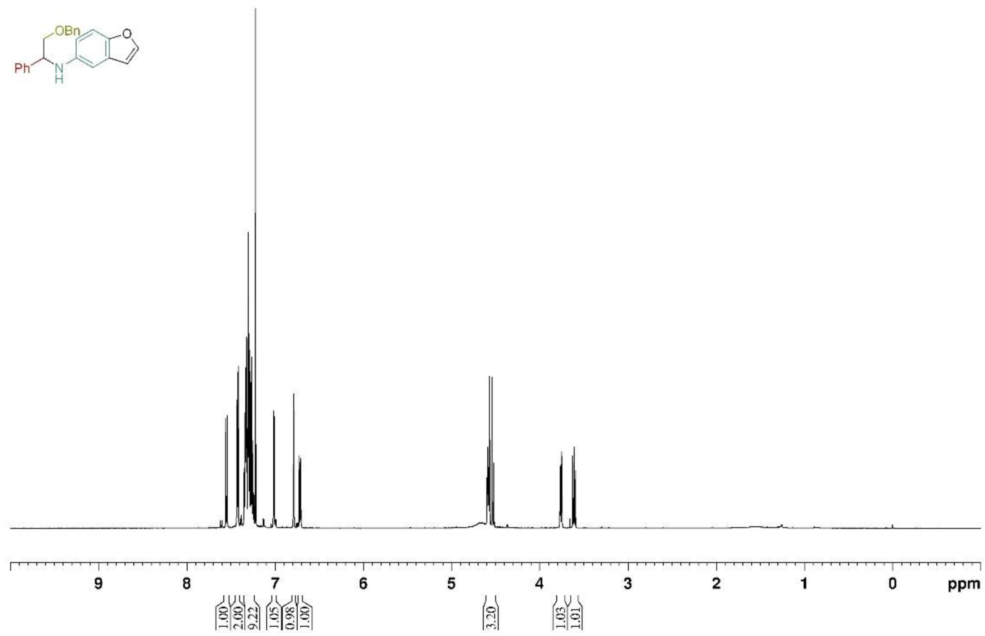

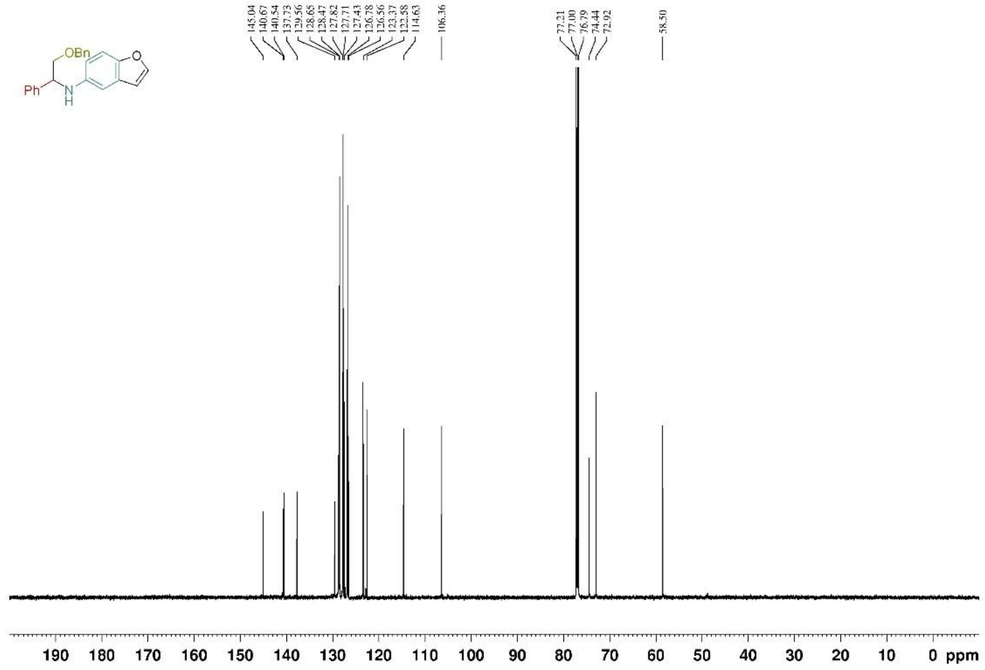

^1H NMR and ^{13}C NMR spectra of compound **6az**

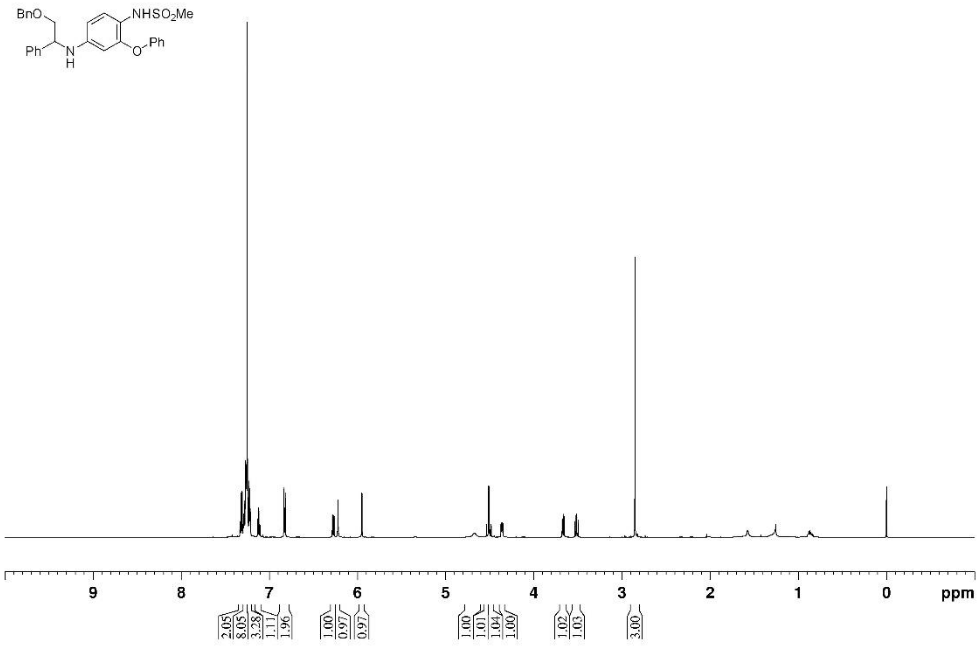

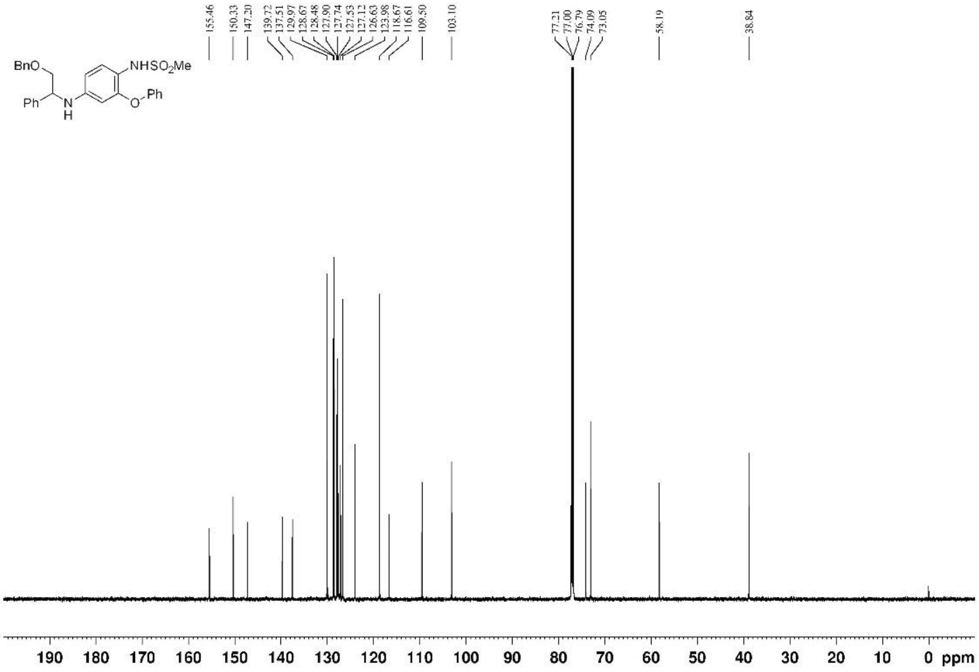

^{1}H NMR and ^{13}C NMR spectra of compound **7**

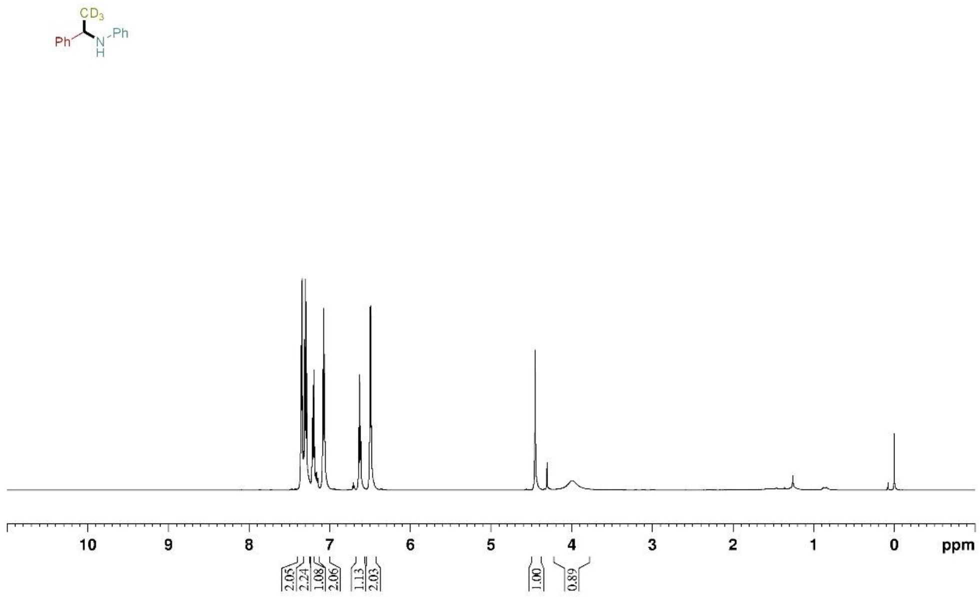

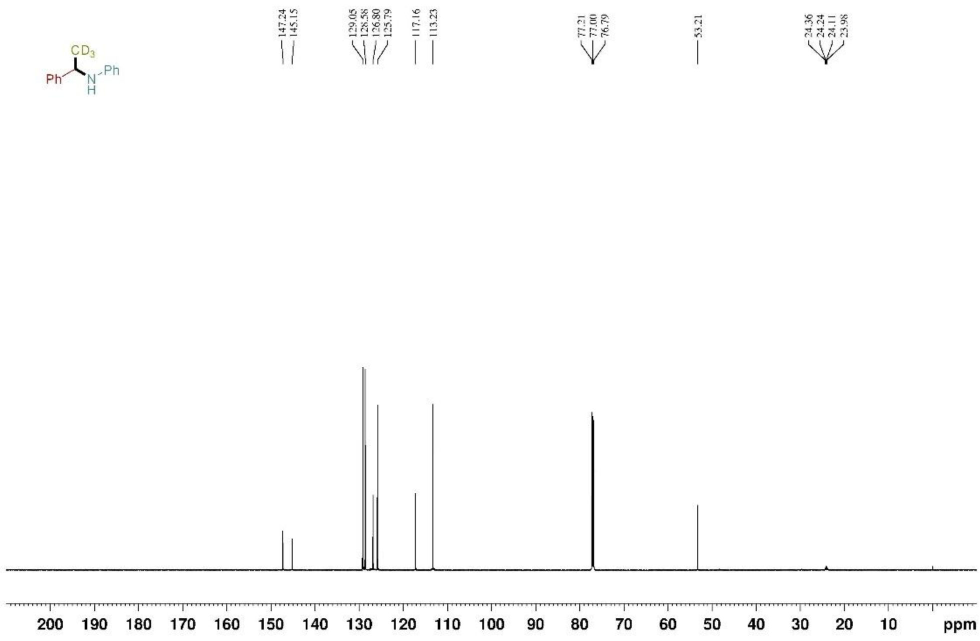

^{1}H NMR and ^{13}C NMR spectra of compound **8**

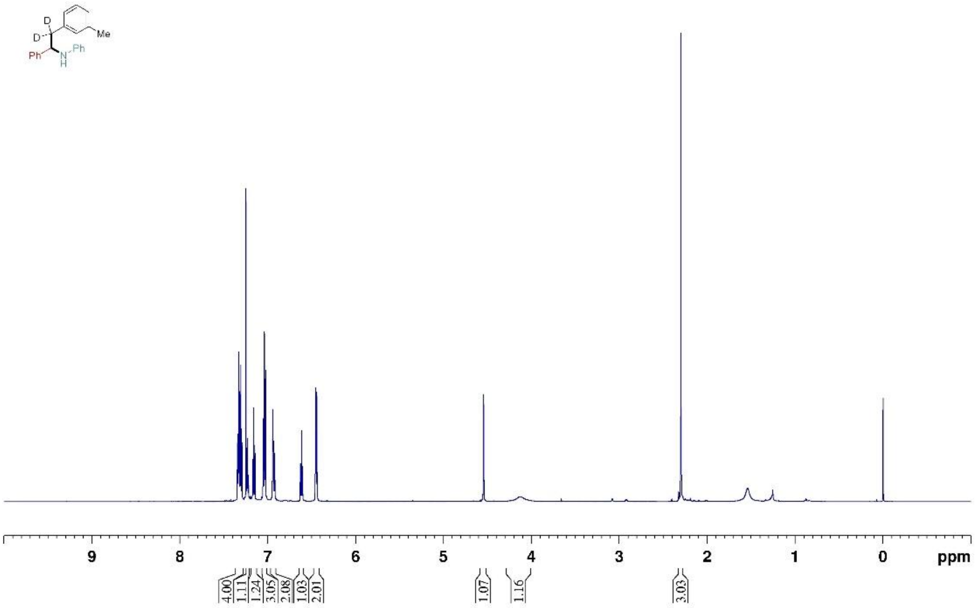

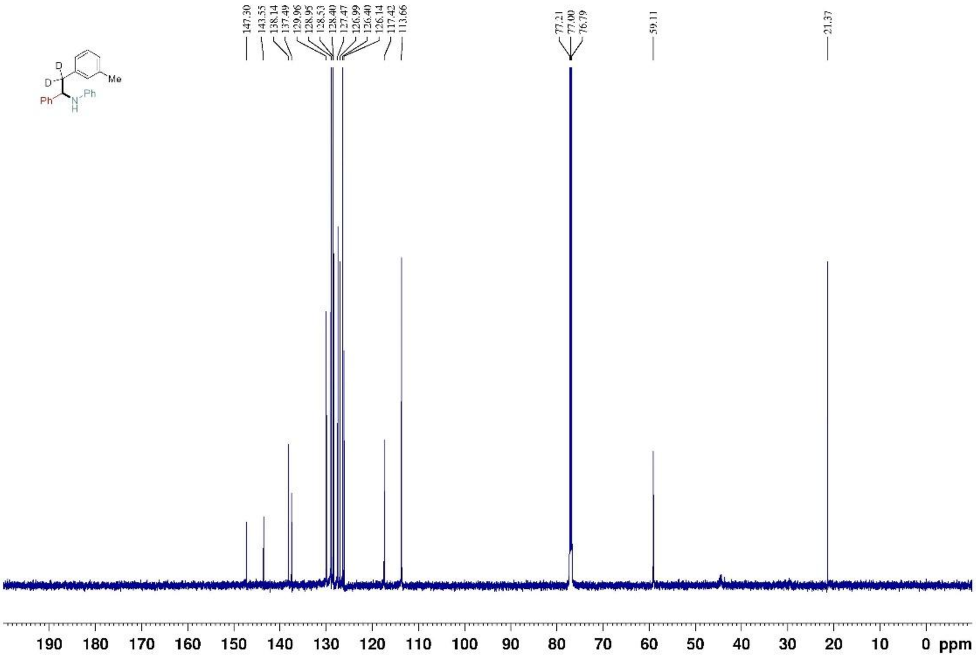

^{1}H NMR and ^{13}C NMR spectra of compound **9**

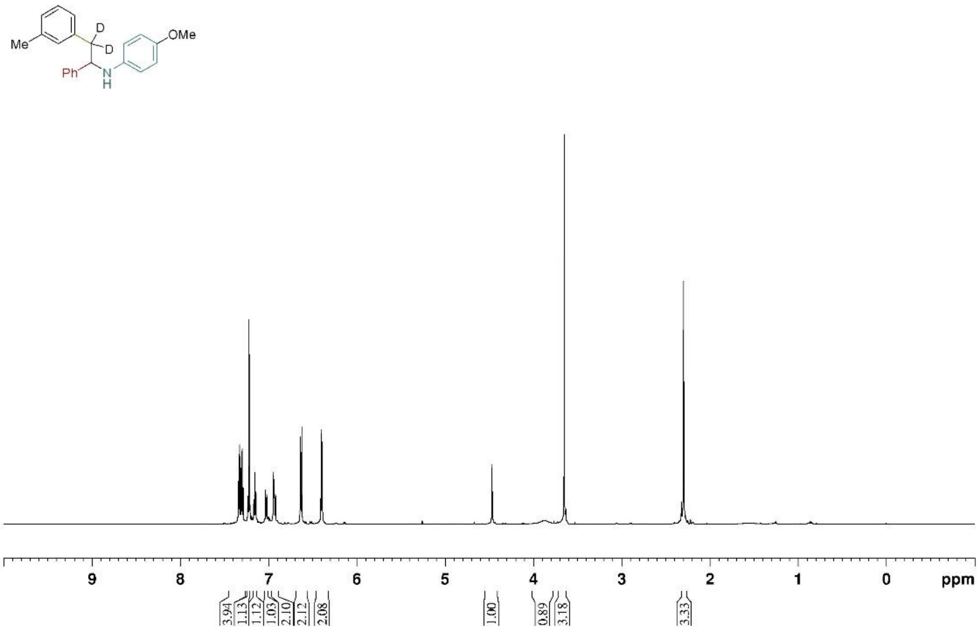

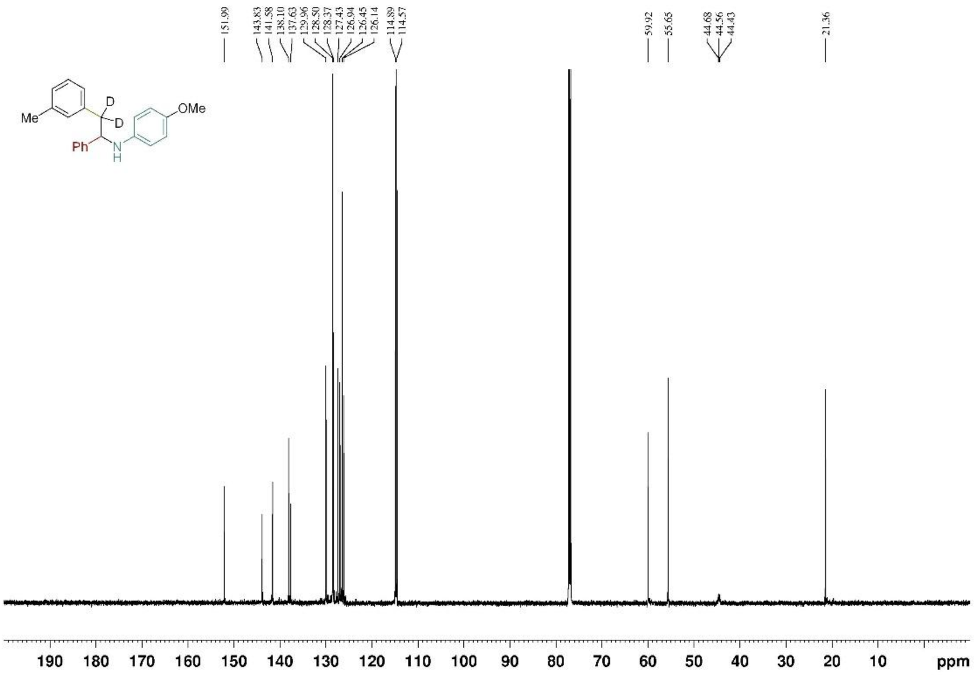

^{1}H NMR and ^{13}C NMR spectra of compound **10**

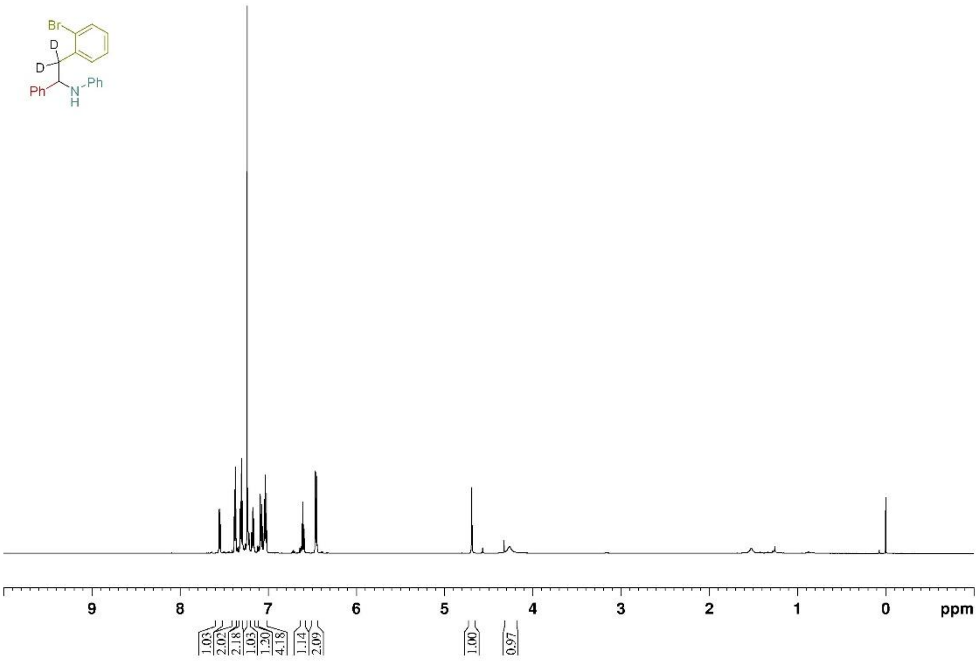

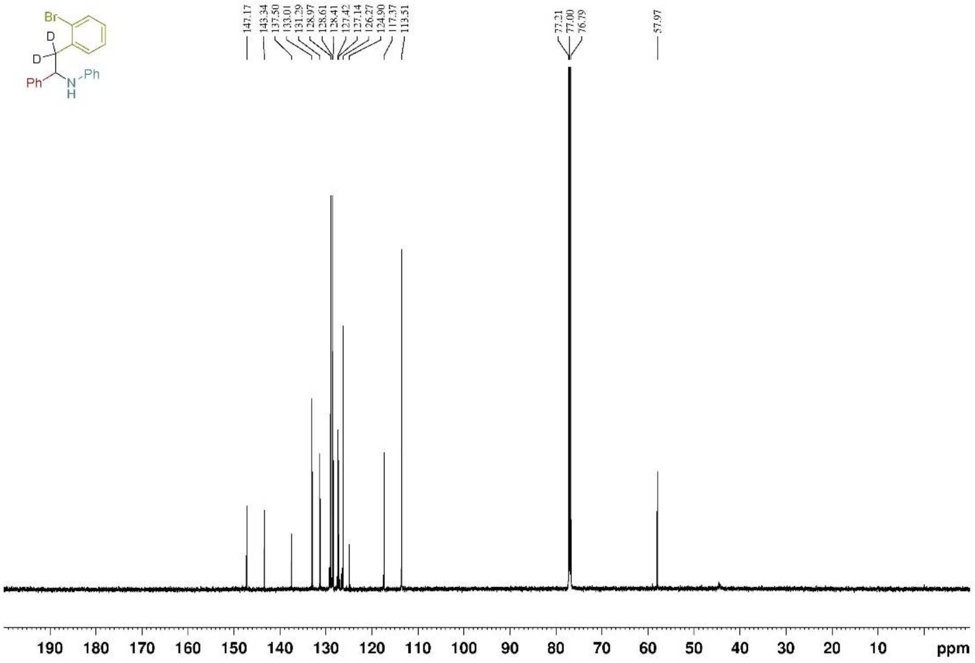

^{1}H NMR and ^{13}C NMR spectra of compound **11**

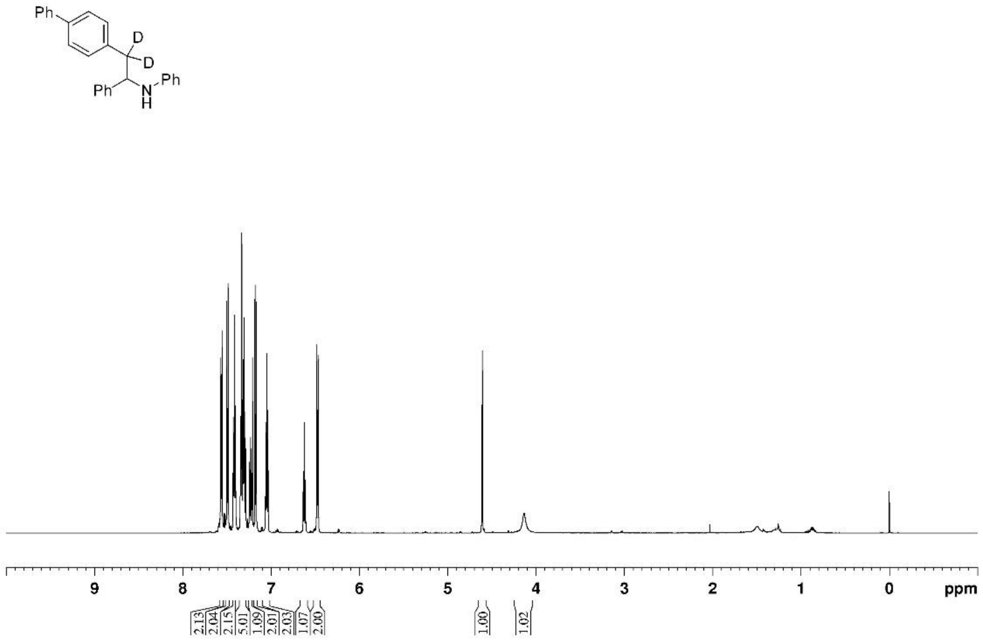

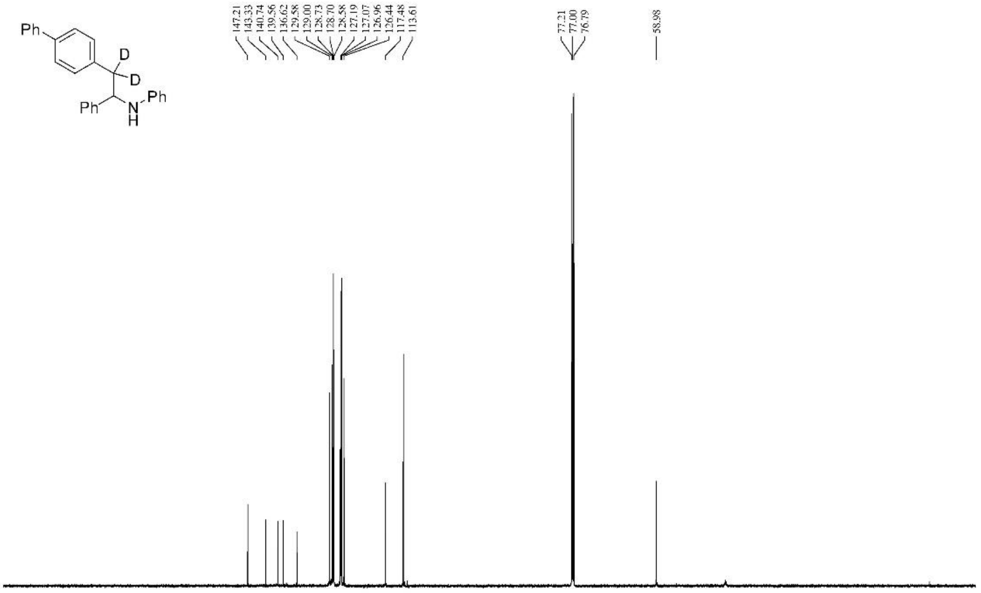

^{1}H NMR and ^{13}C NMR spectra of compound **12**

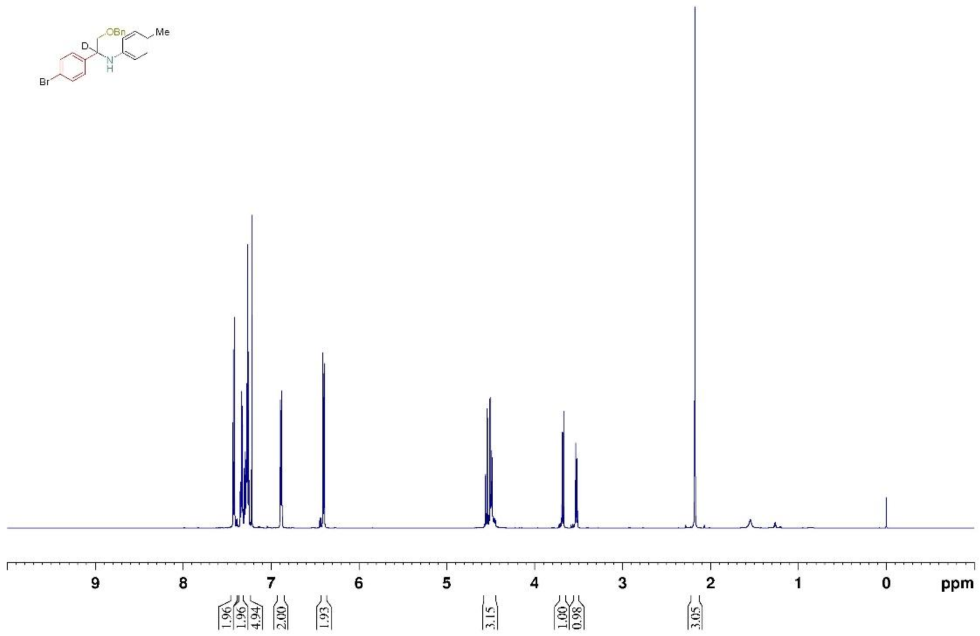

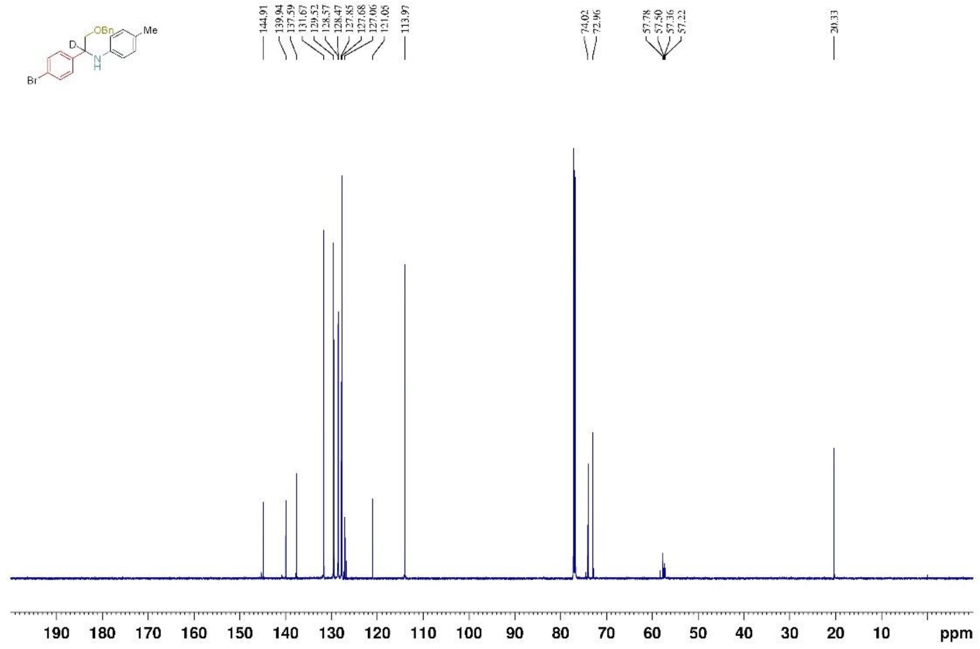

^{1}H NMR and ^{13}C NMR spectra of compound **13**

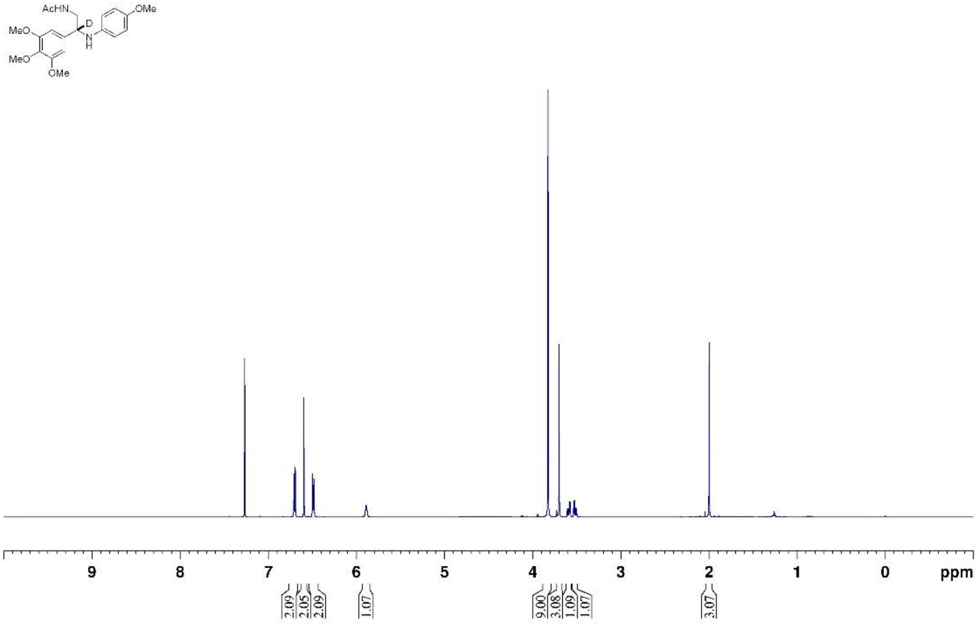

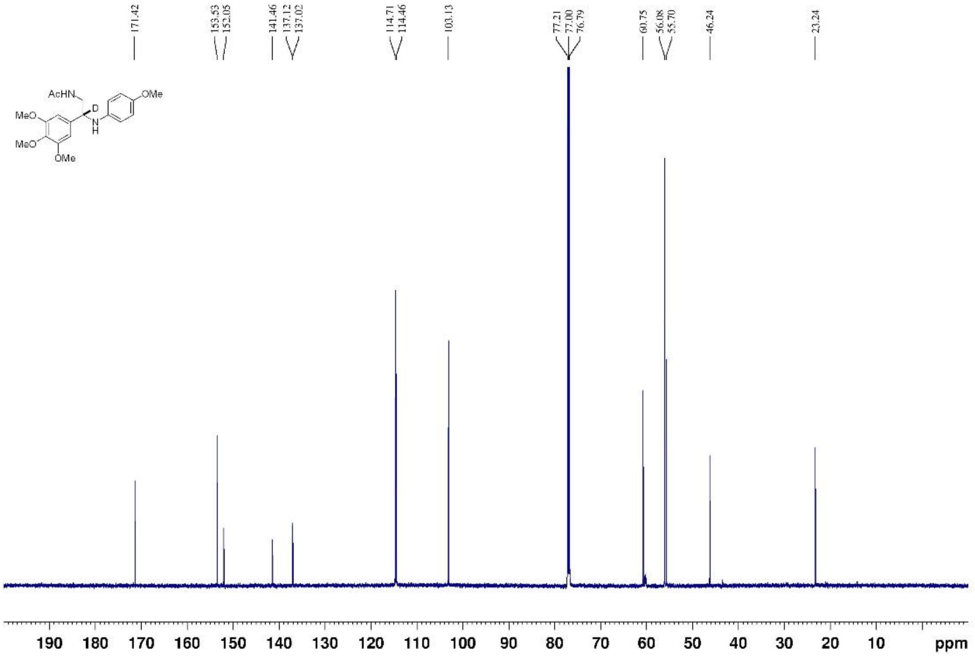

^{1}H NMR and ^{13}C NMR spectra of compound **14**

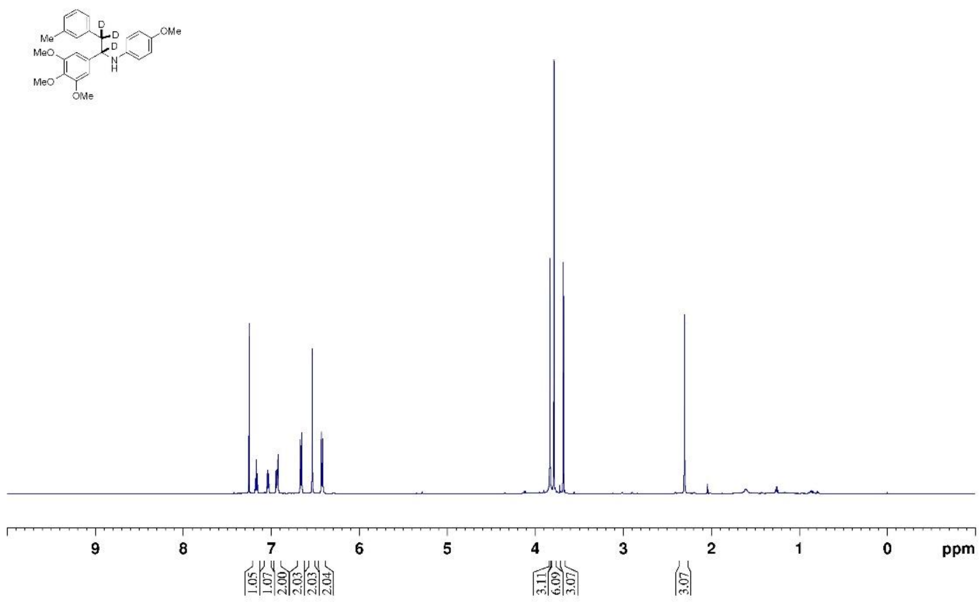

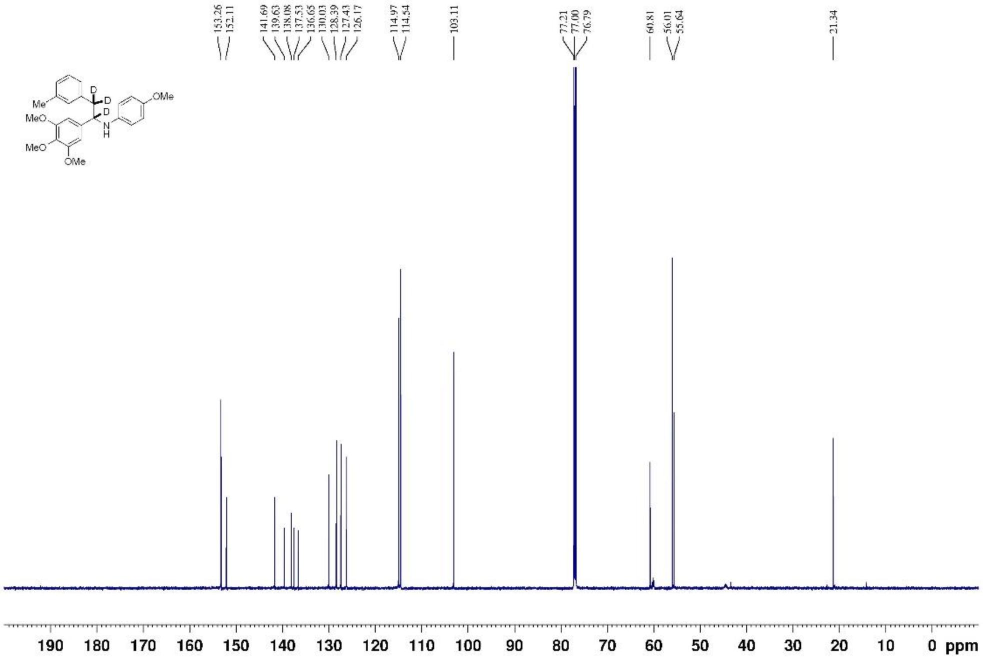

^{1}H NMR and ^{13}C NMR spectra of compound **15**

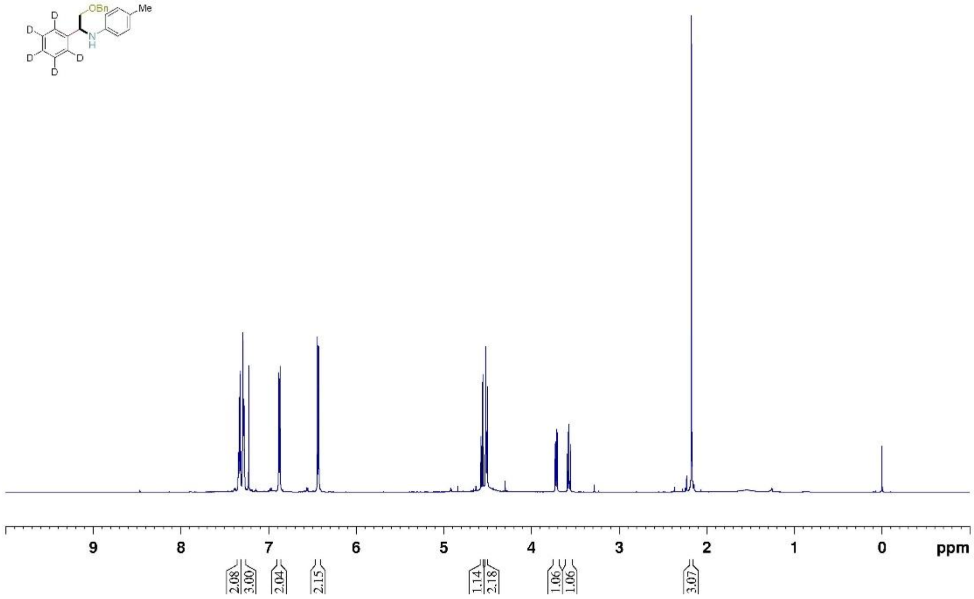

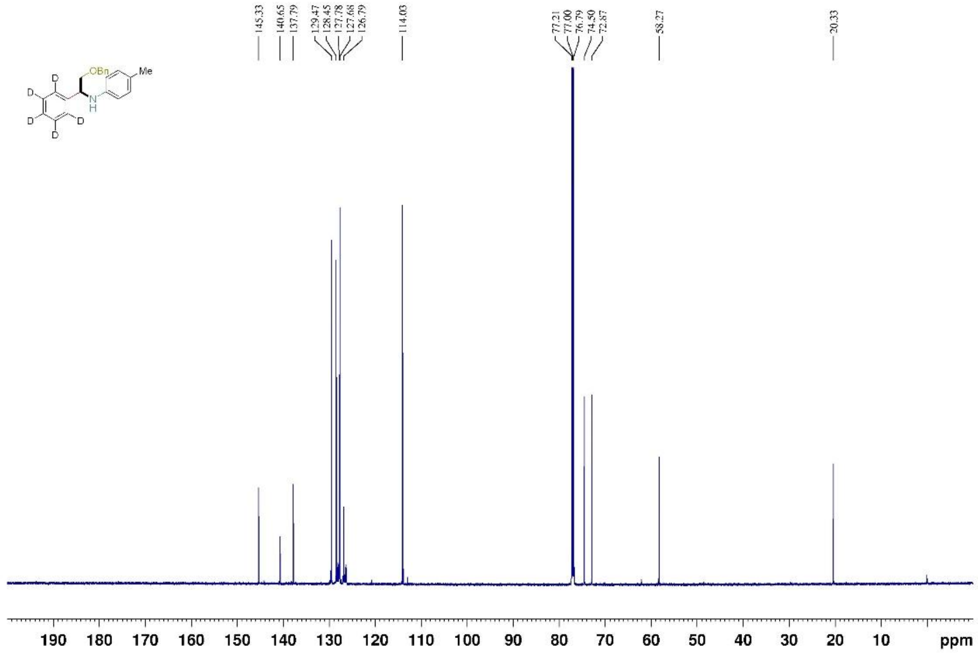

^{1}H NMR and ^{13}C NMR spectra of compound **16**

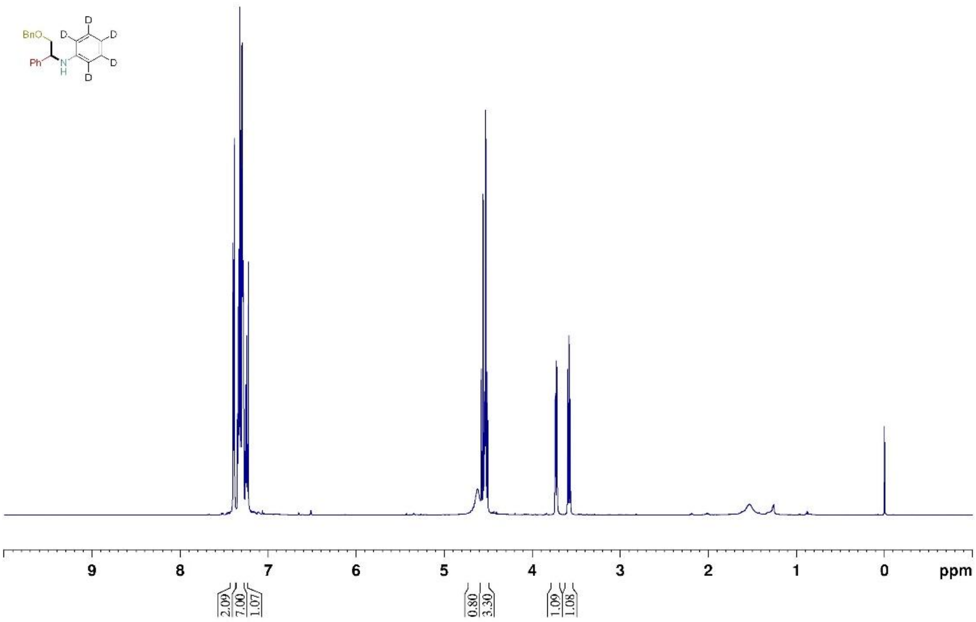

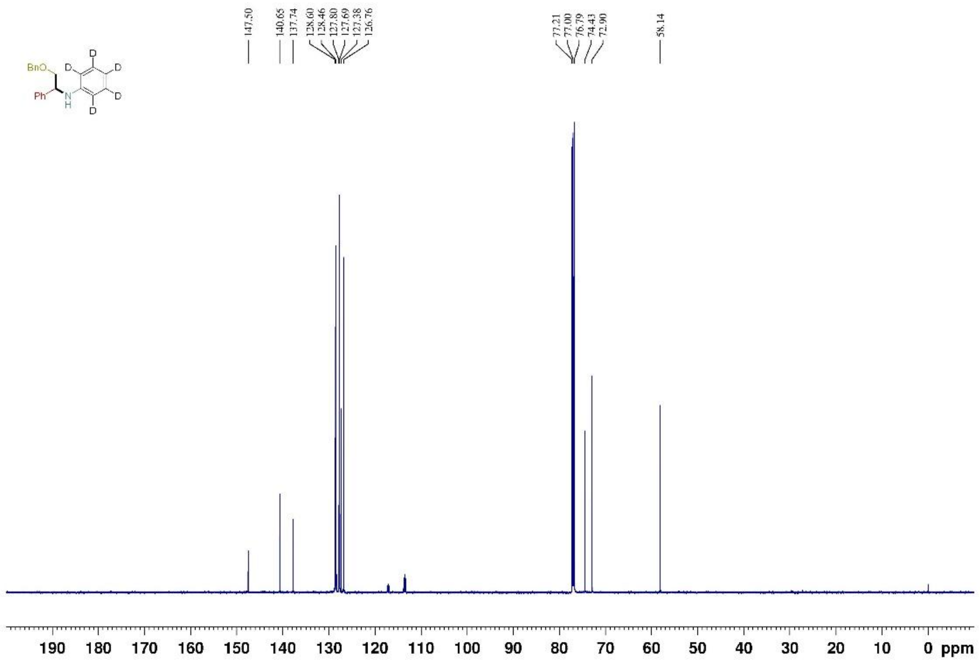

^1H NMR and ^{13}C NMR spectra of compound 1**7**

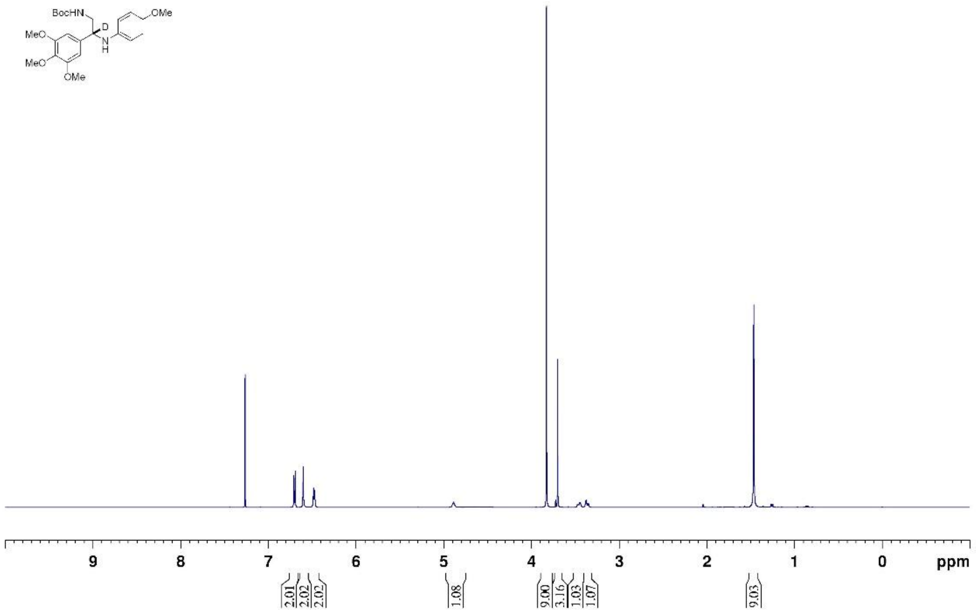

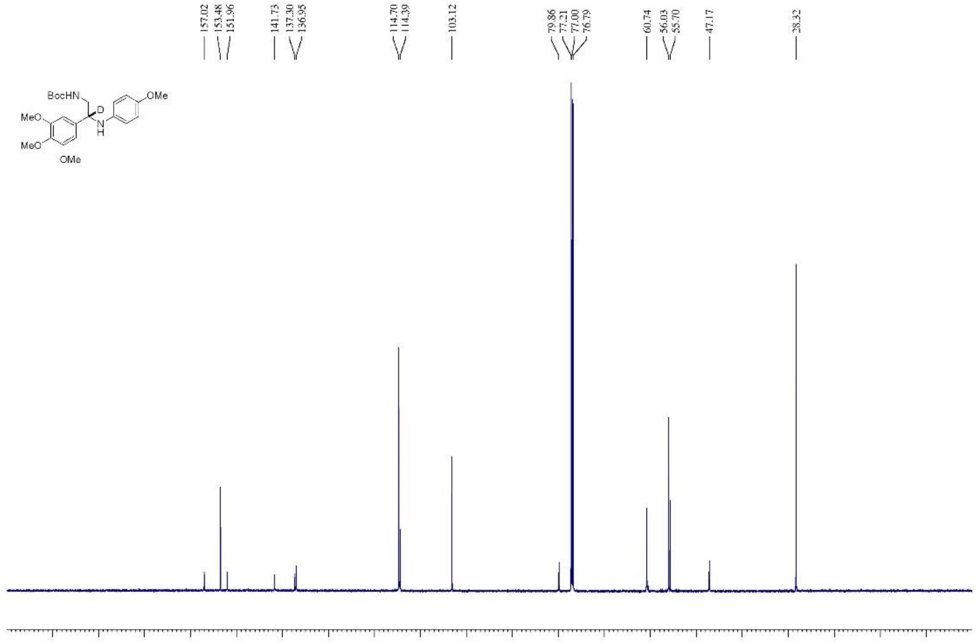

^{1}H NMR and ^{13}C NMR spectra of compound **18**

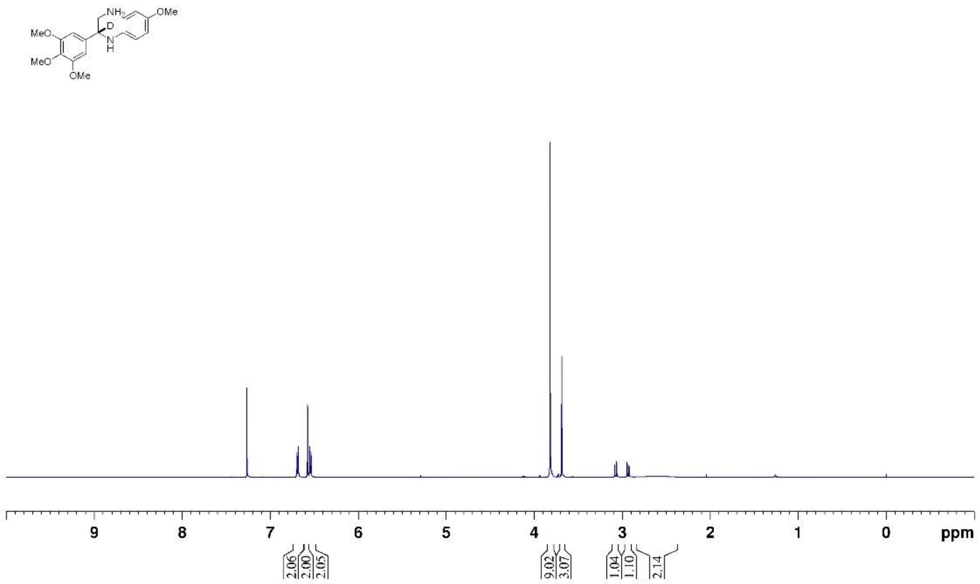

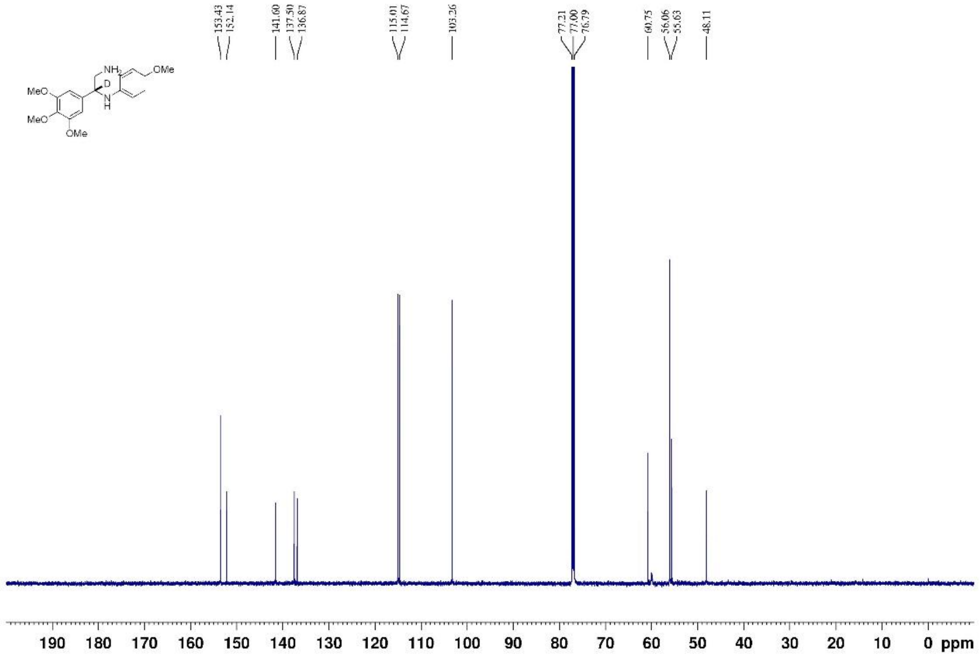

^{1}H NMR and ^{13}C NMR spectra of compound **19**

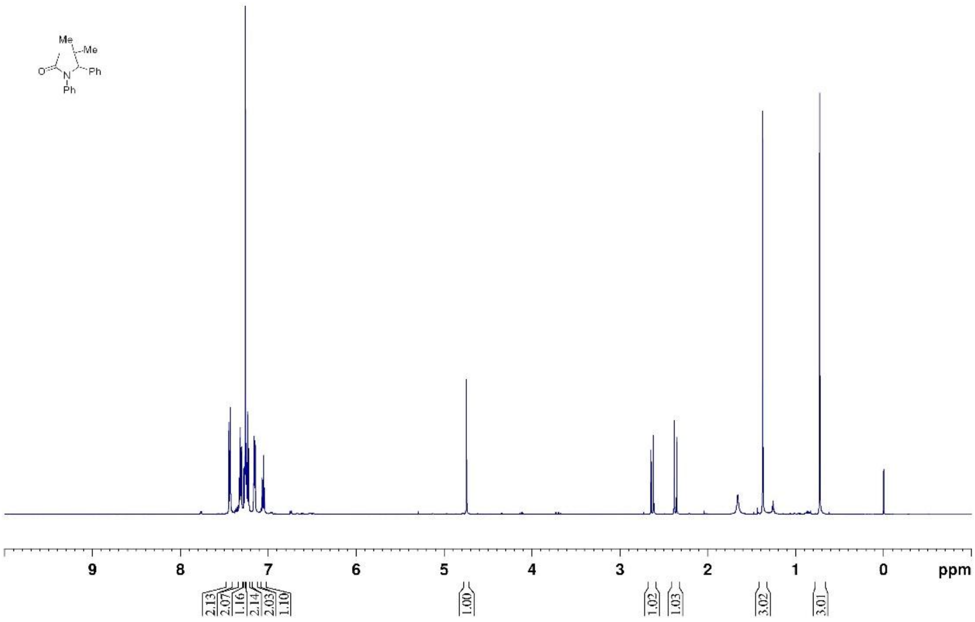

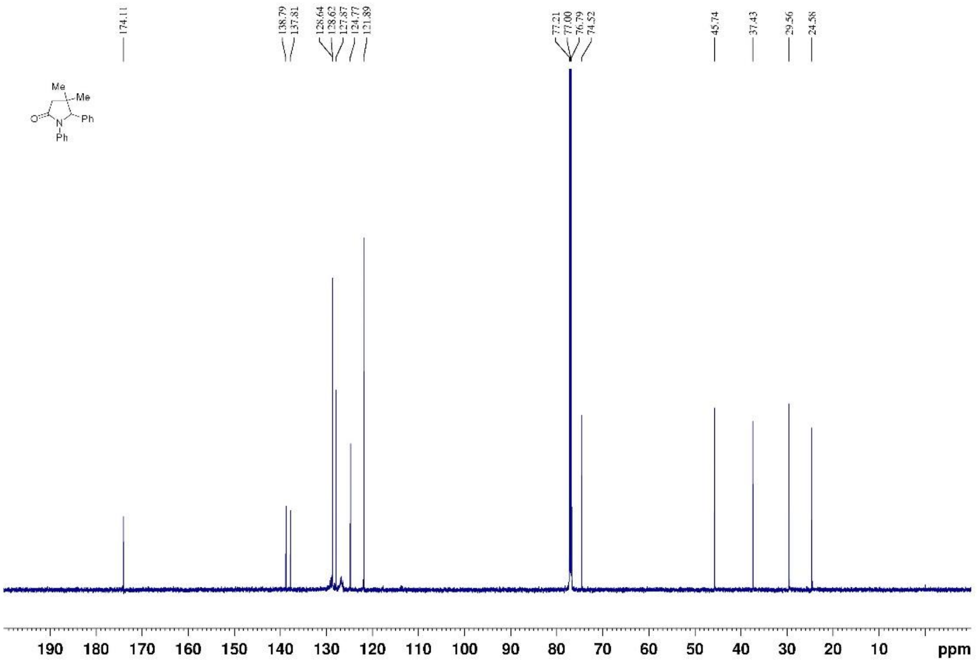

^{1}H NMR and ^{13}C NMR spectra of compound **20**

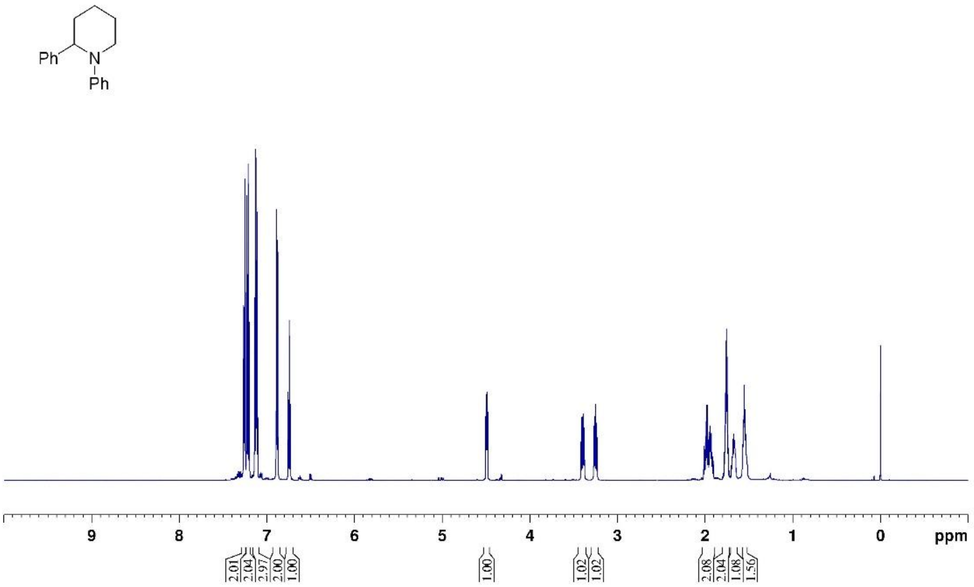

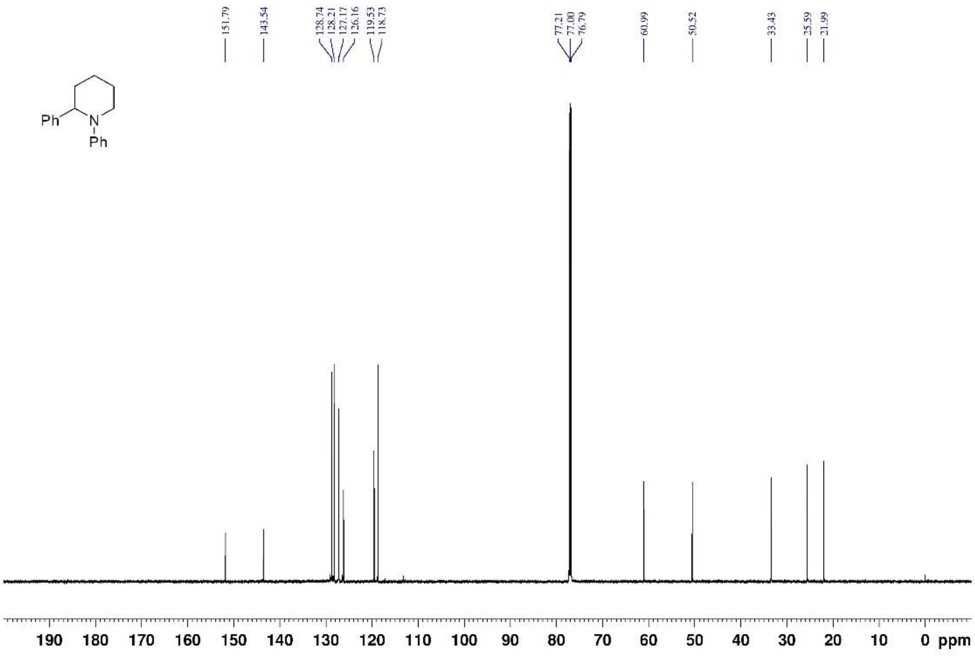

^1H NMR and ^{13}C NMR spectra of compound **21**

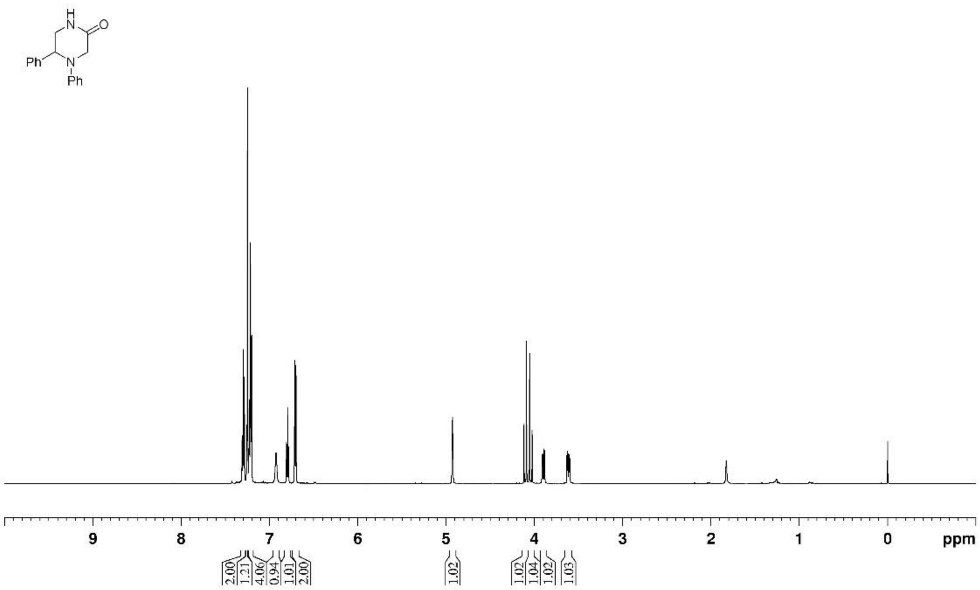

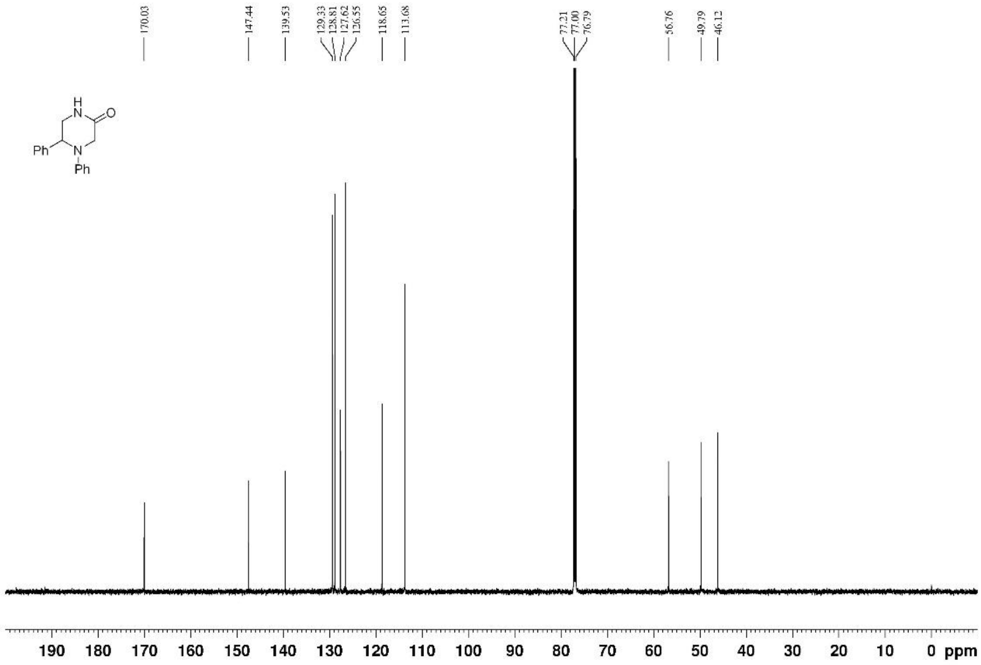

^{1}H NMR and ^{13}C NMR spectra of compound **22**

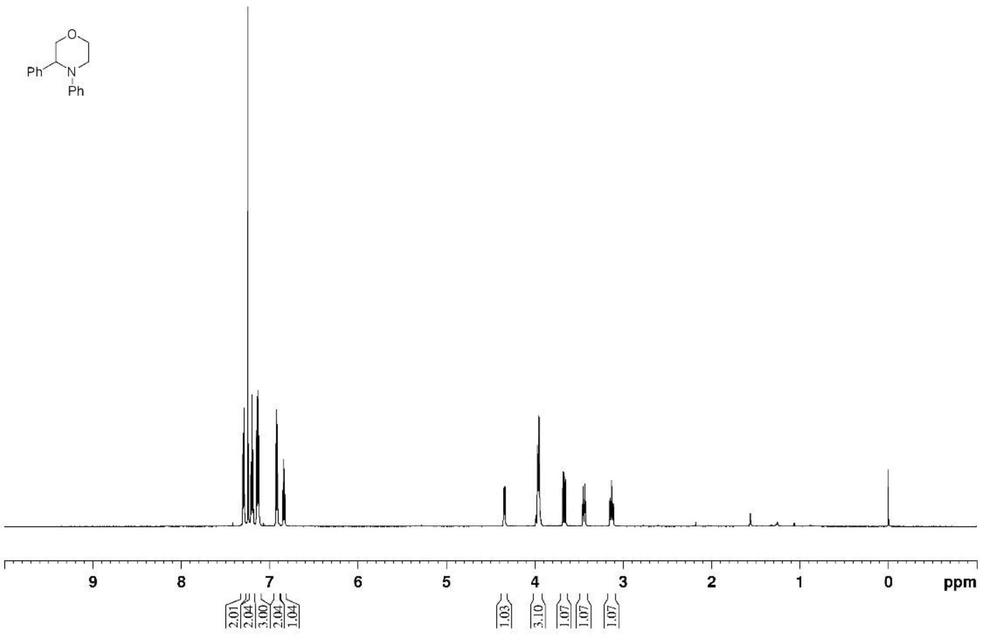

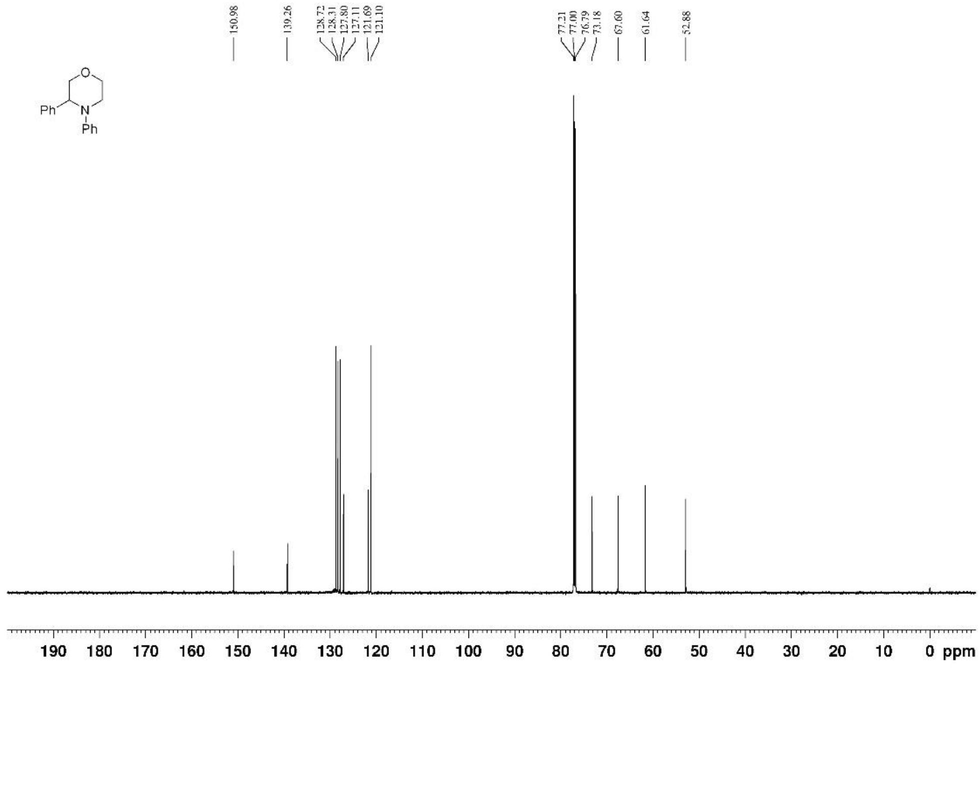

^{1}H NMR and ^{13}C NMR spectra of compound **23**

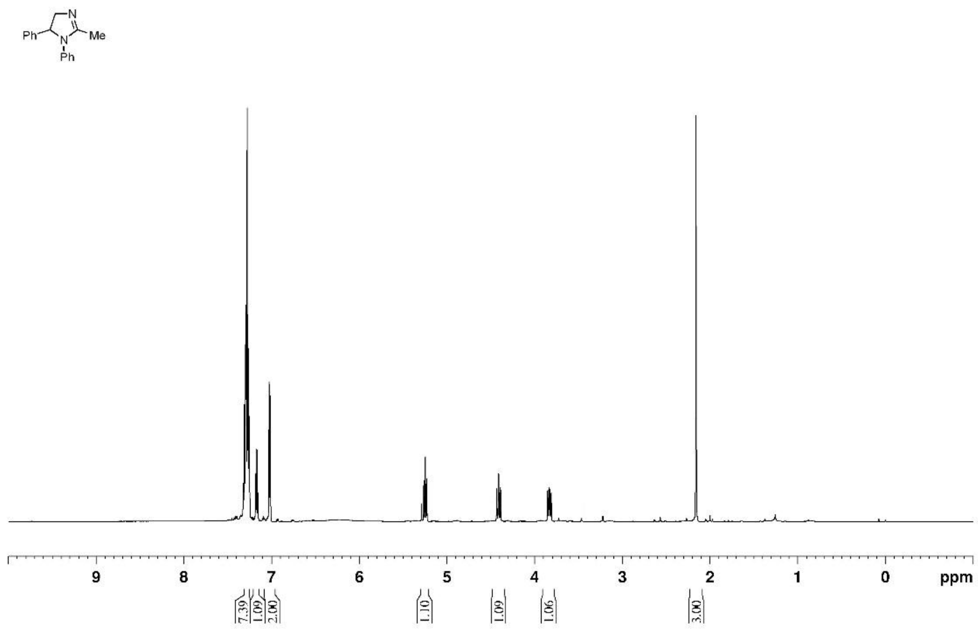

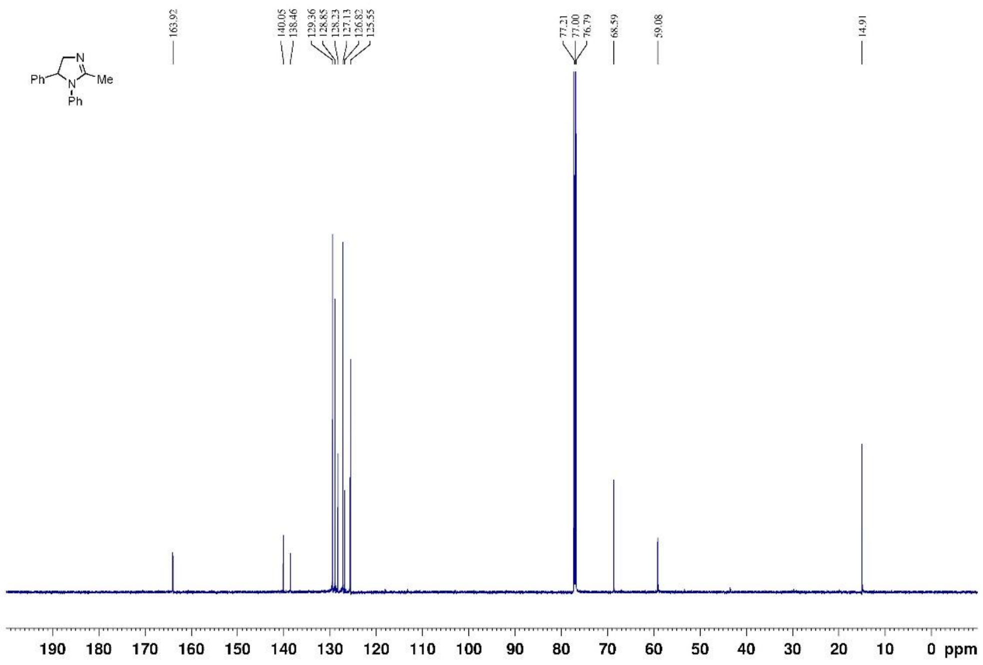

^{1}H NMR and ^{13}C NMR spectra of compound **24**

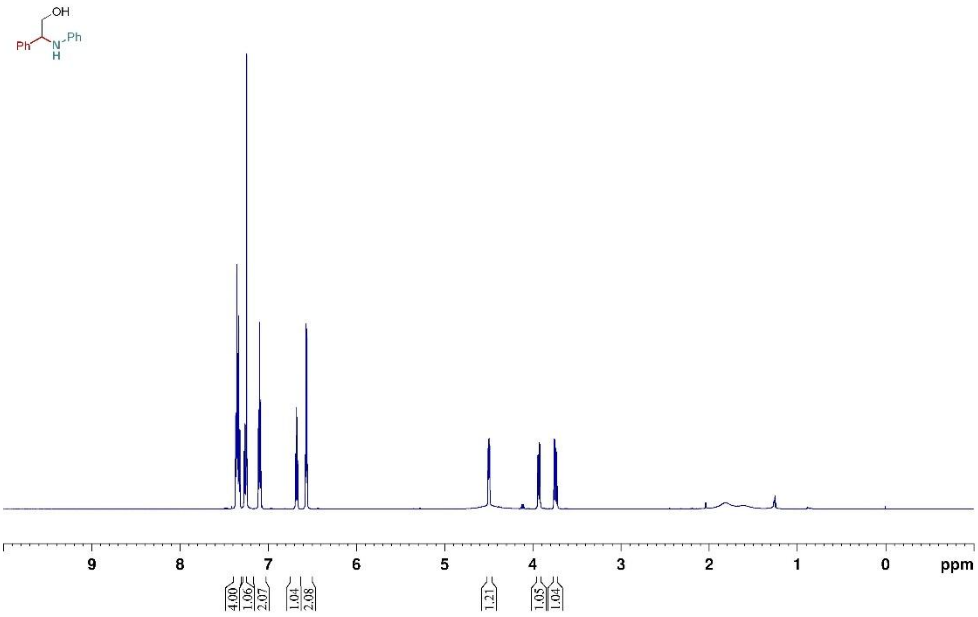

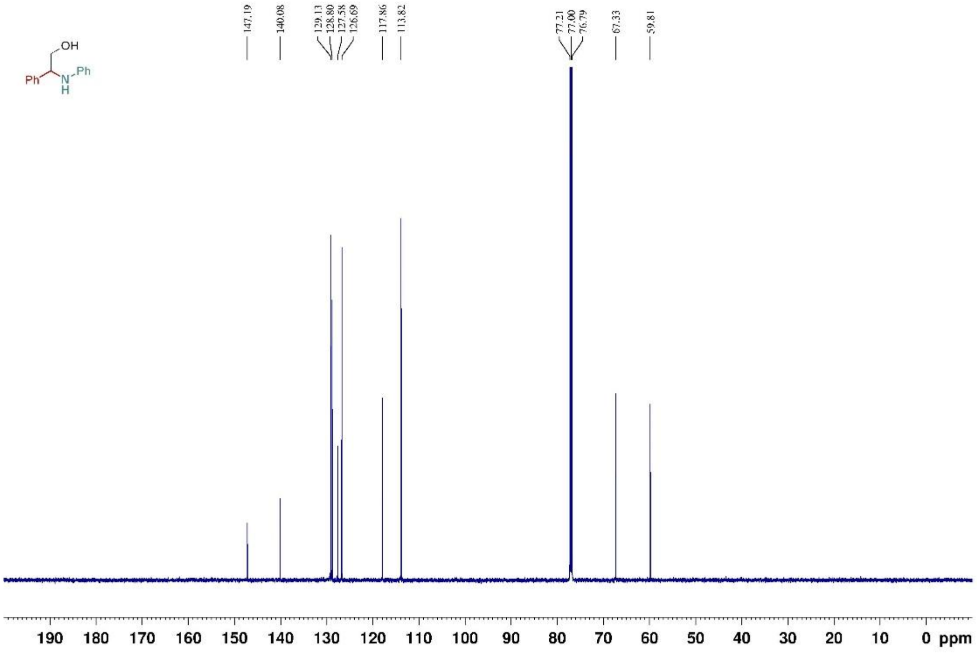

^{1}H NMR and ^{13}C NMR spectra of compound **25**

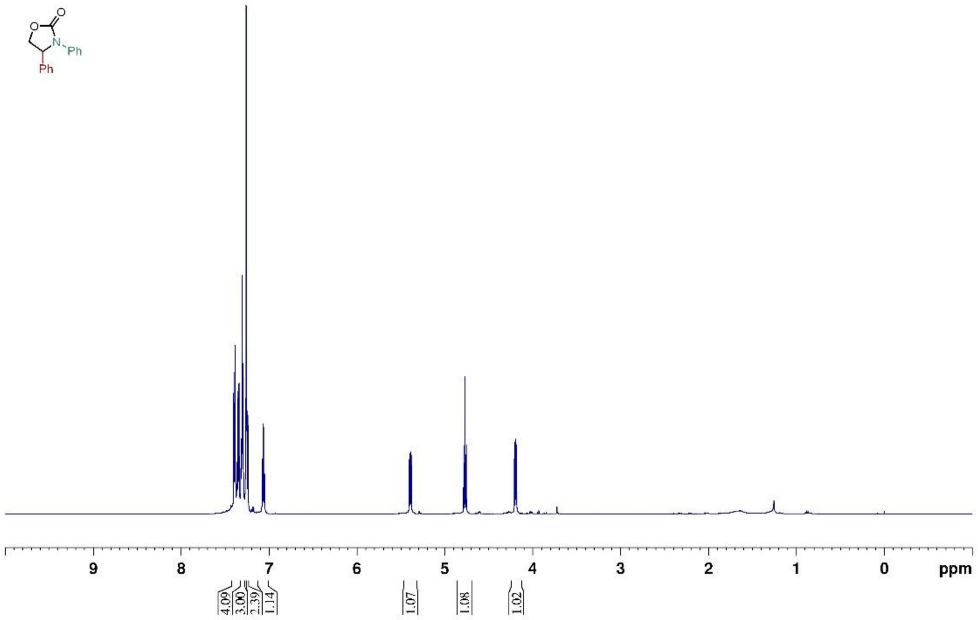

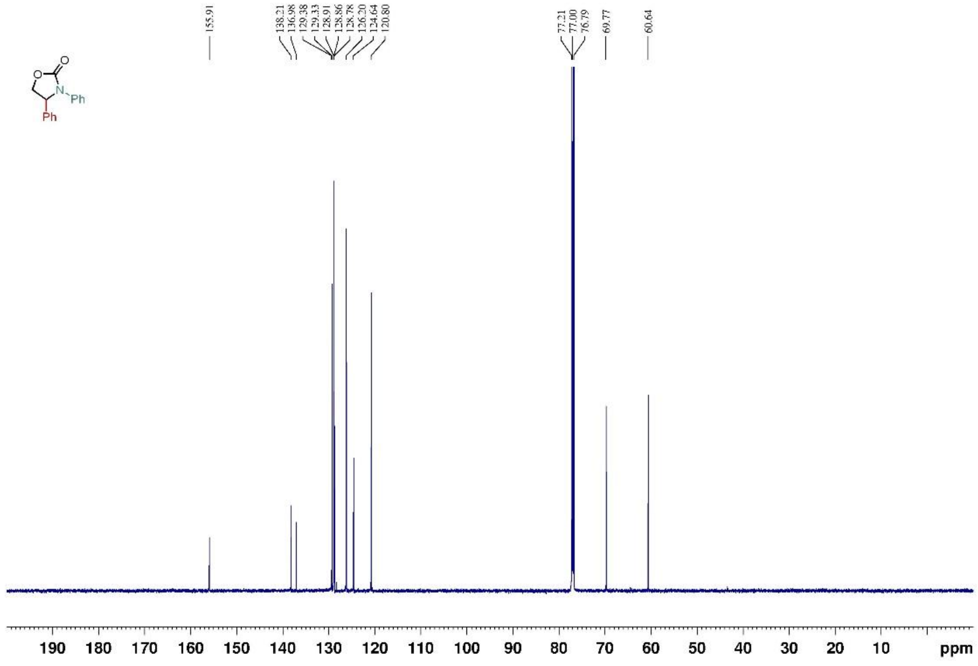

^{1}H NMR and ^{13}C NMR spectra of compound **26**

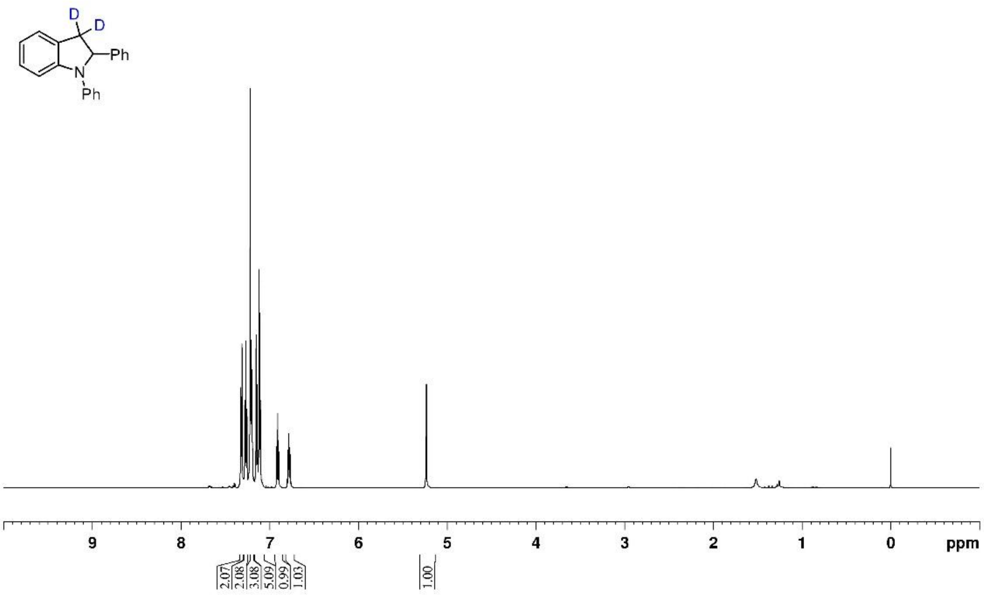

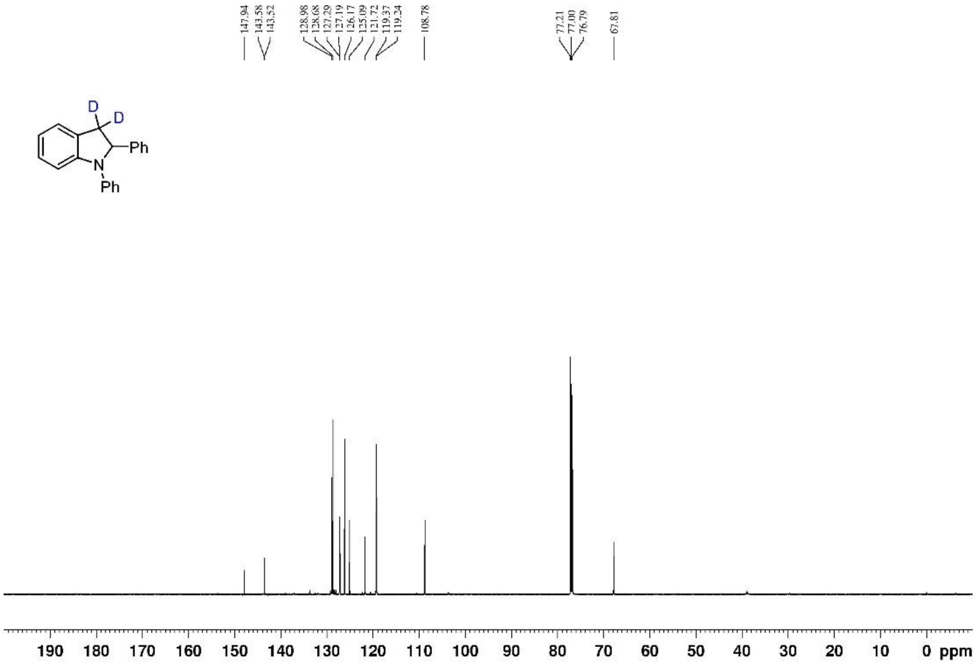

^{1}H NMR and ^{13}C NMR spectra of compound **27a**

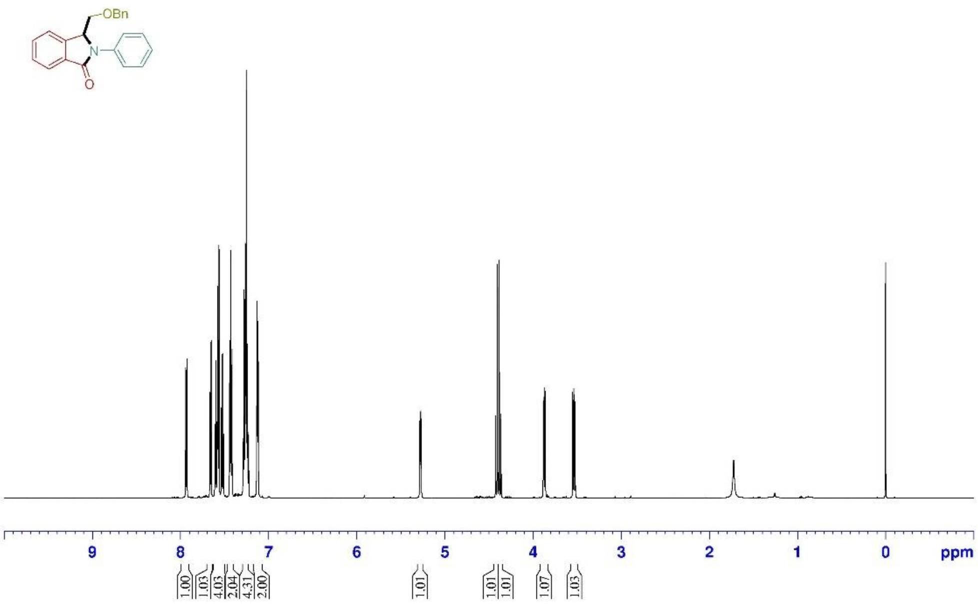

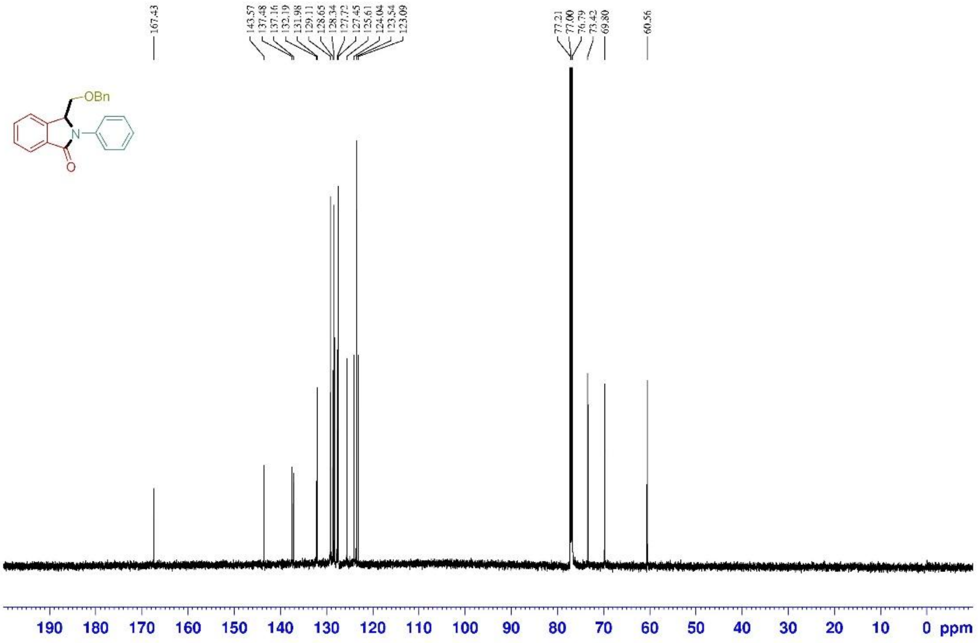

^{1}H NMR and ^{13}C NMR spectra of compound **27b**

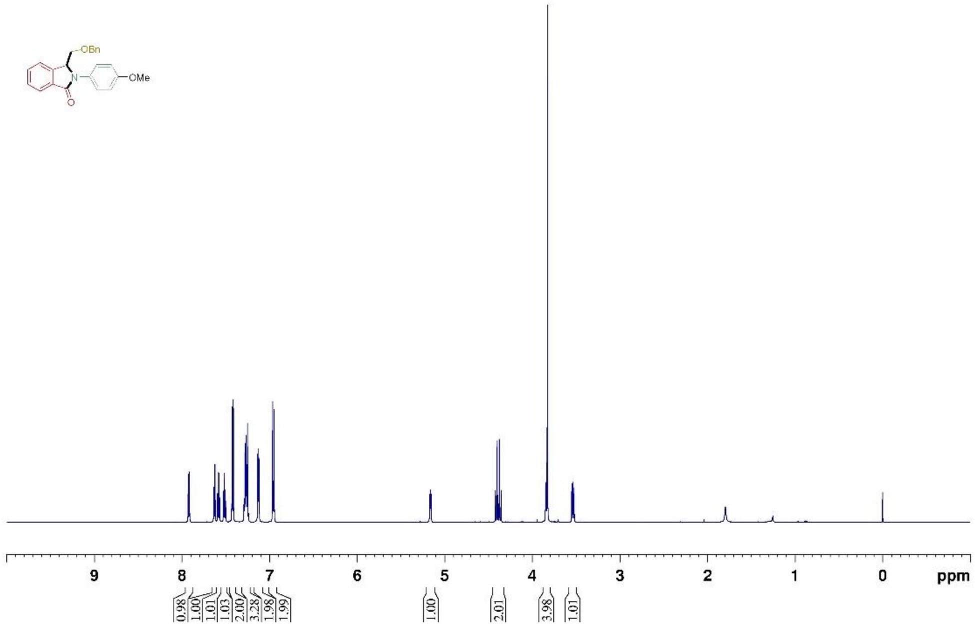

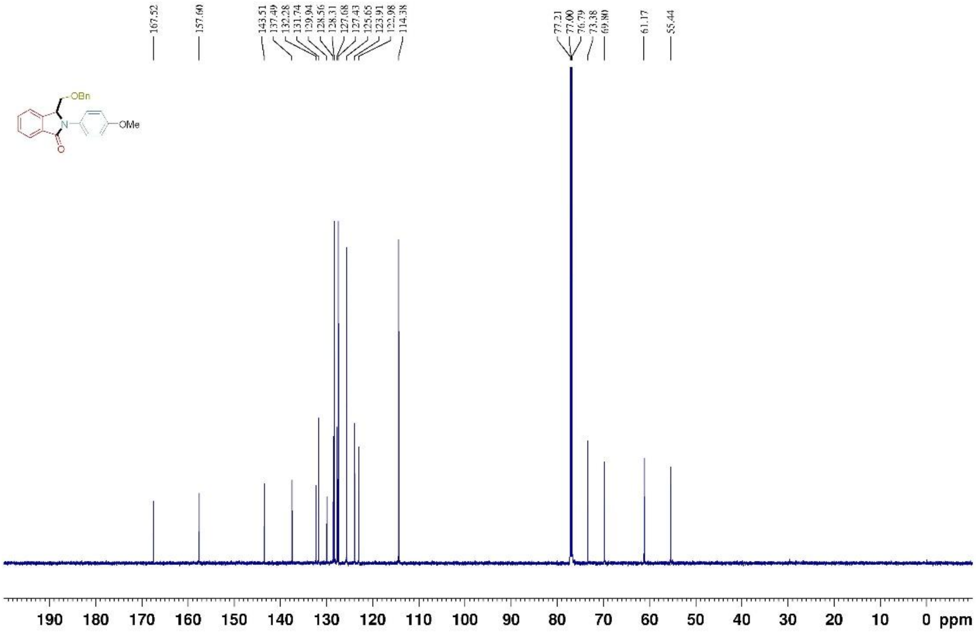

^{1}H NMR and ^{13}C NMR spectra of compound **27c**

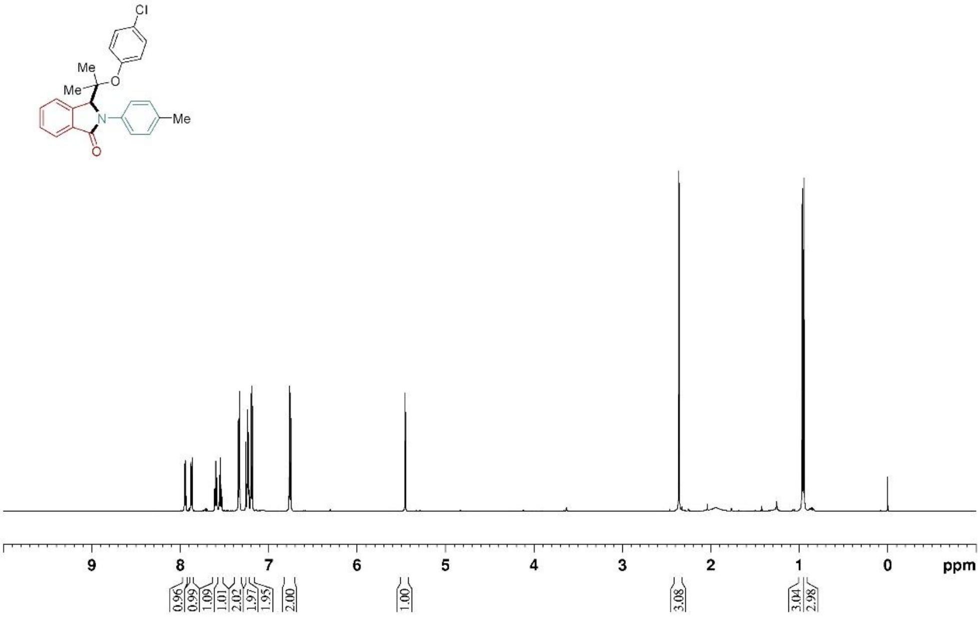

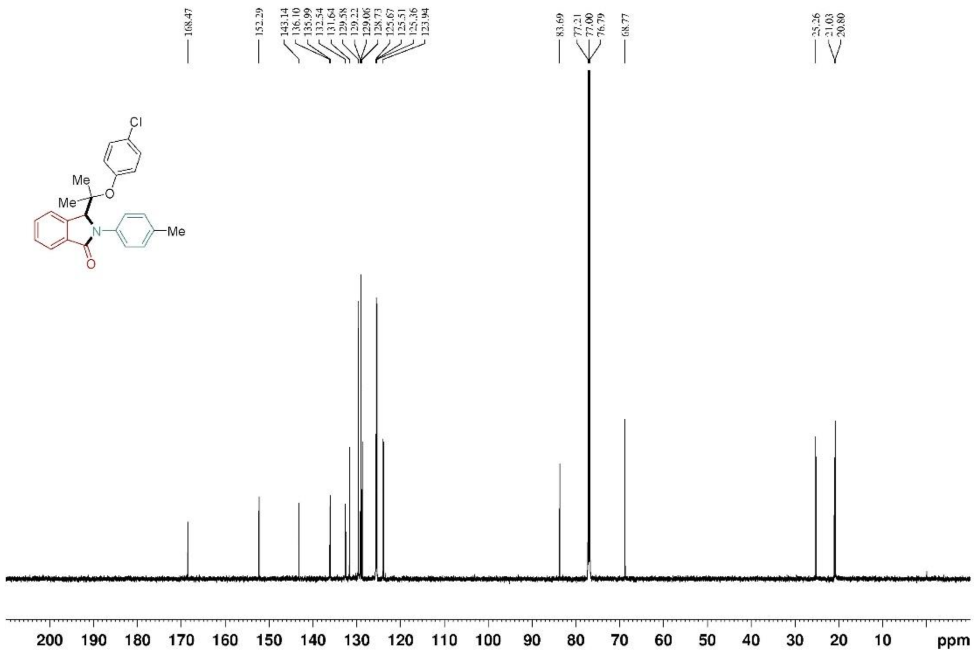

^{1}H NMR and ^{13}C NMR spectra of compound **27d**

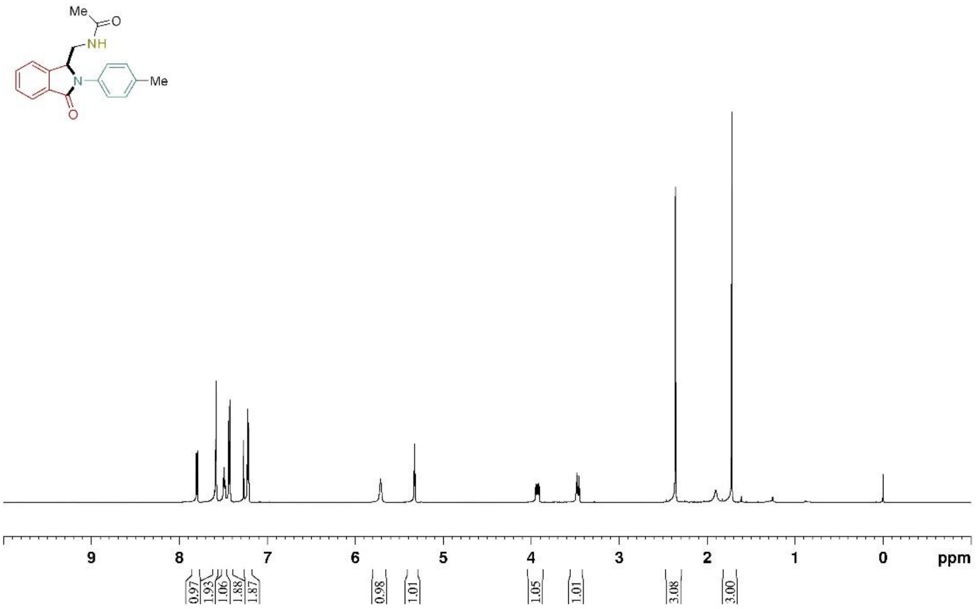

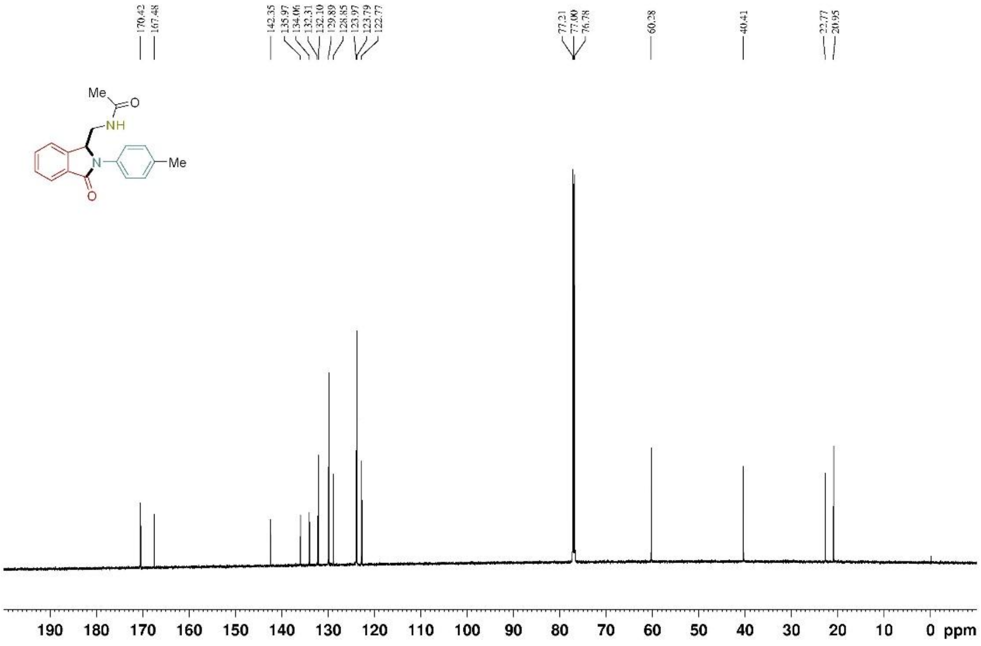

^{1}H NMR and ^{13}C NMR spectra of compound **27e**

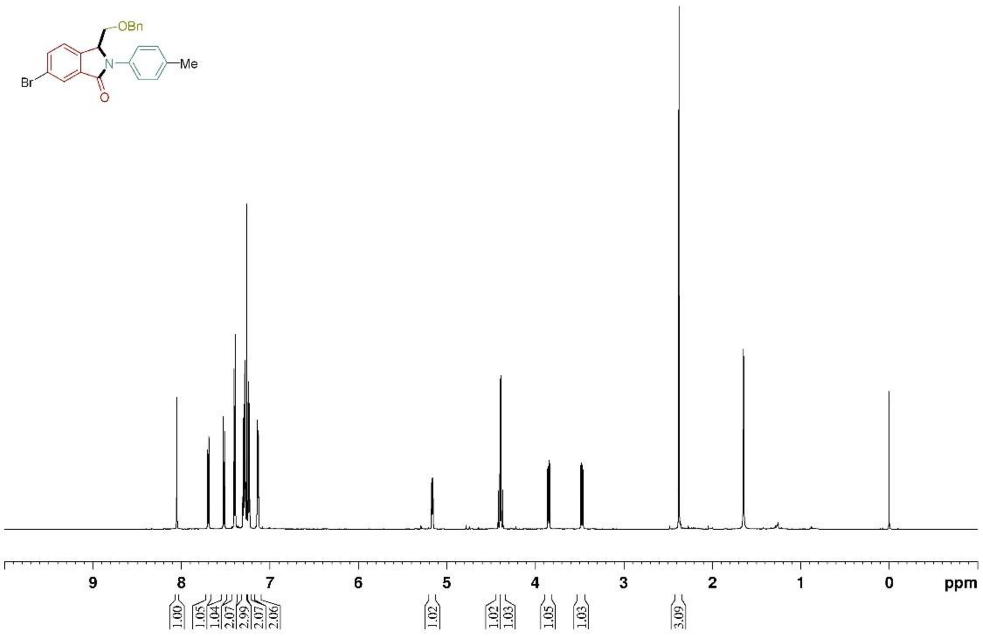

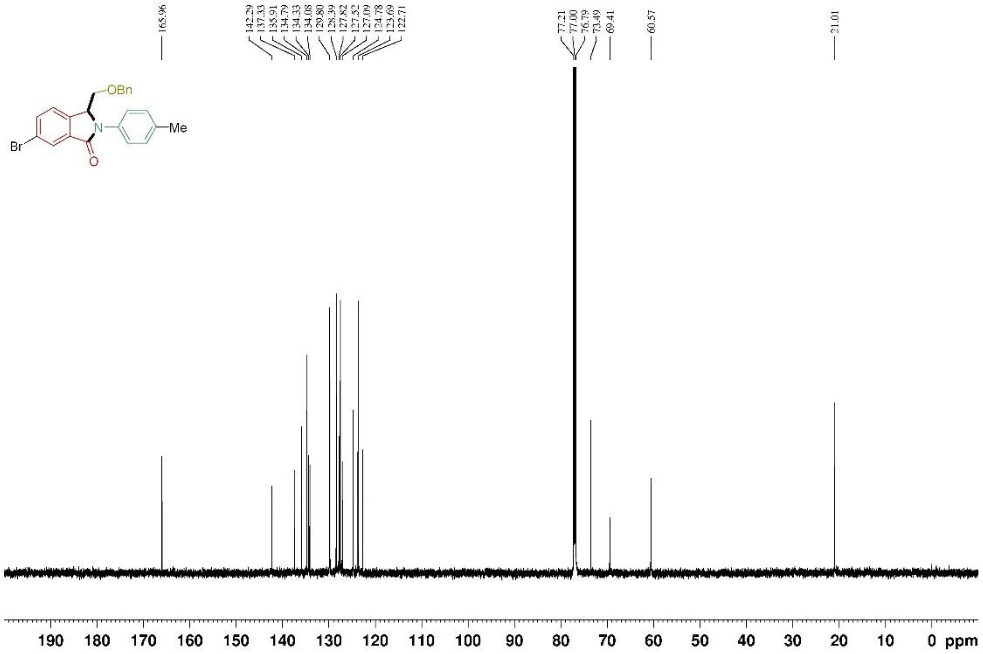

^{1}H NMR and ^{13}C NMR spectra of compound **27f**

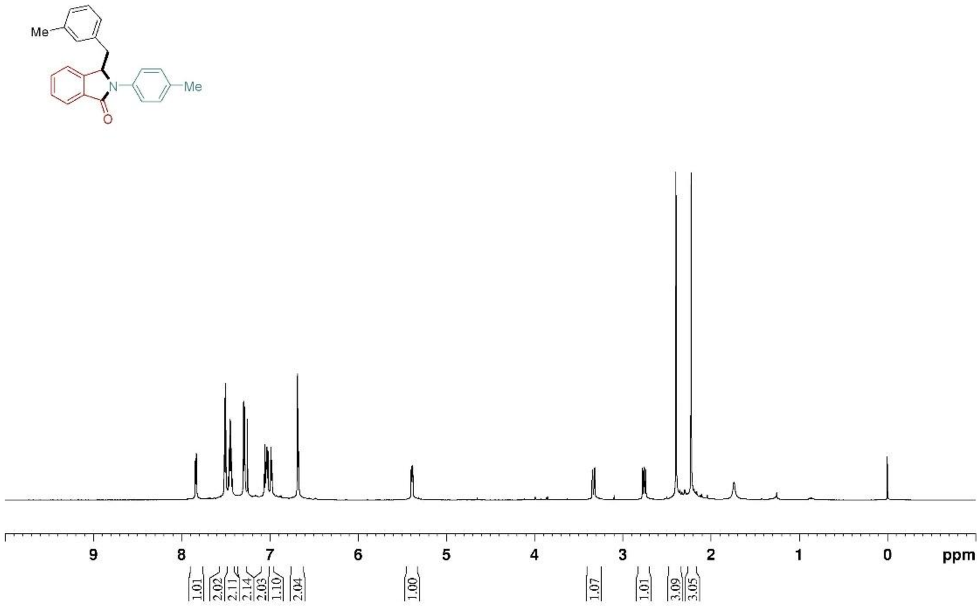

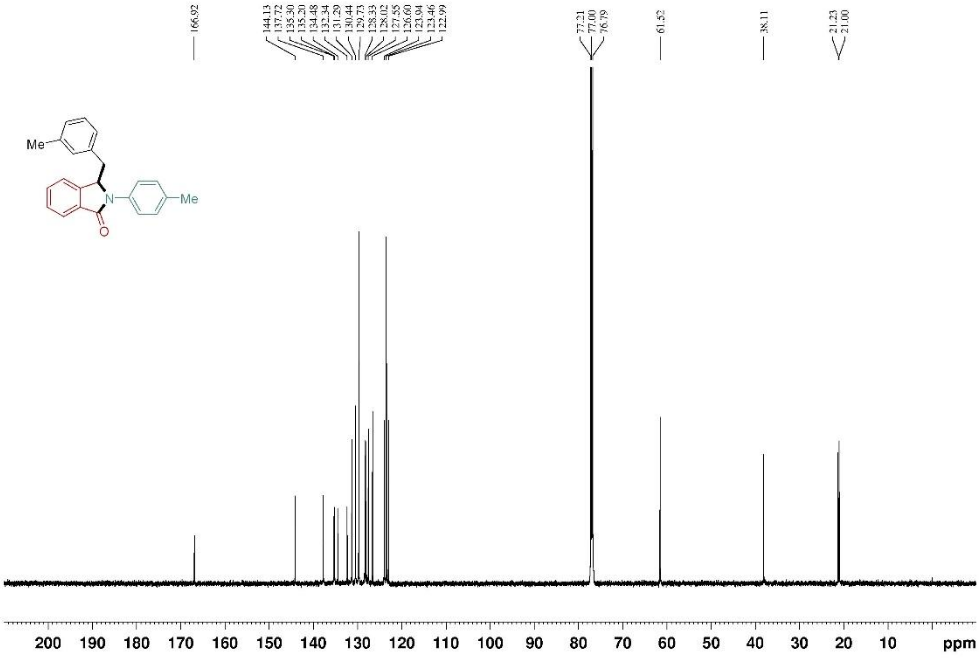

^{1}H NMR and ^{13}C NMR spectra of compound **28a**

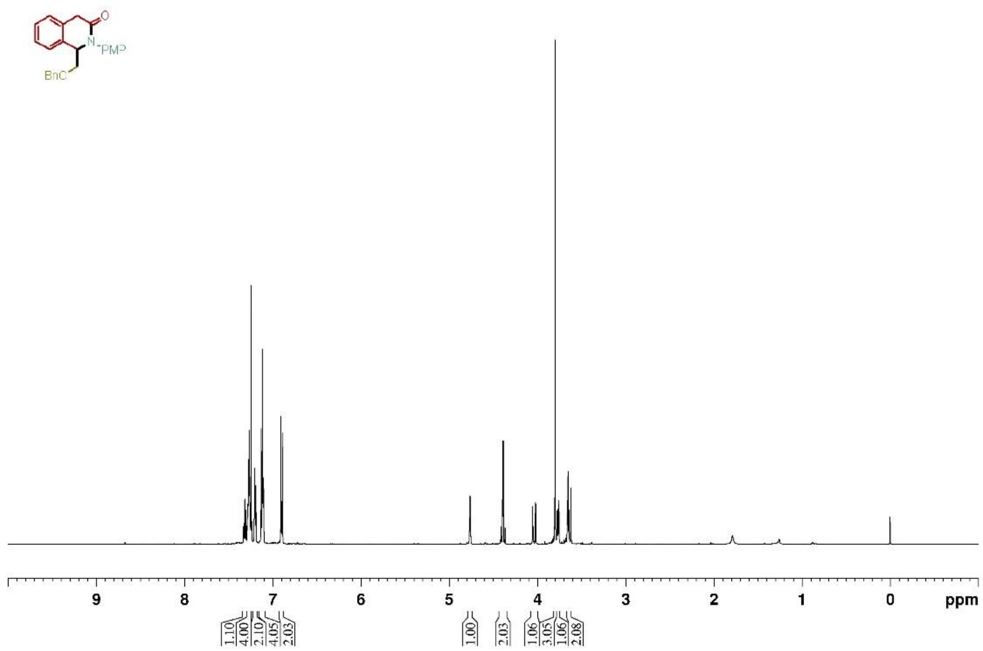

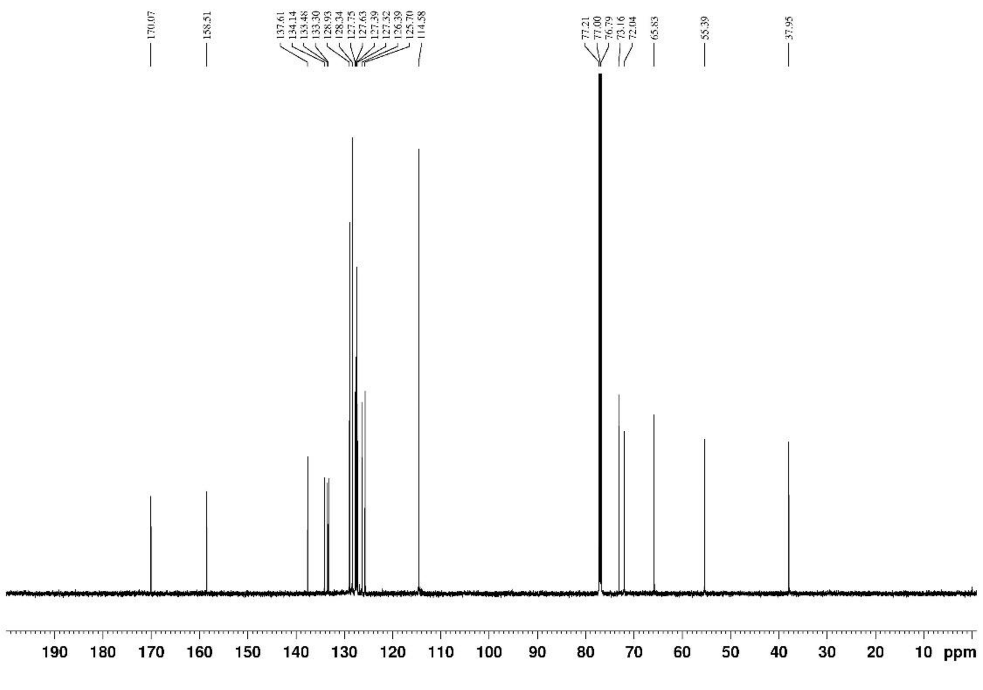

^{1}H NMR and ^{13}C NMR spectra of compound **28b**

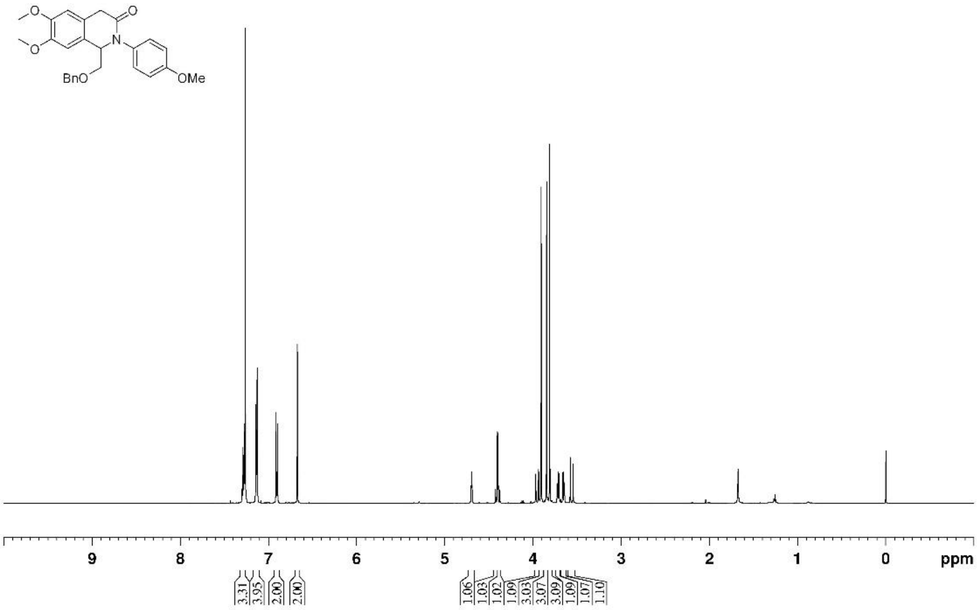

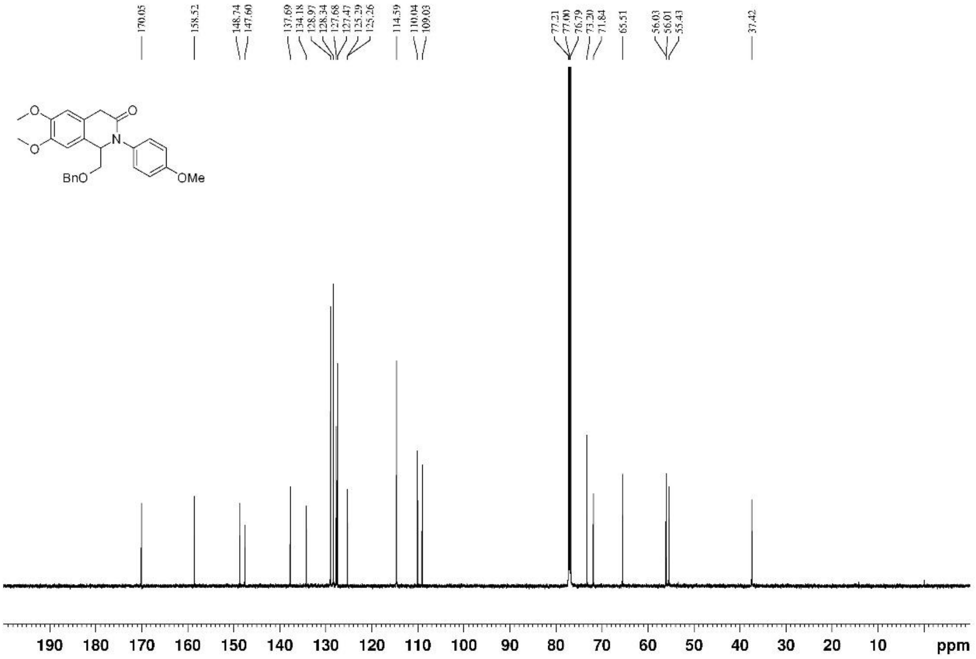

^{1}H NMR and ^{13}C NMR spectra of compound **28c**

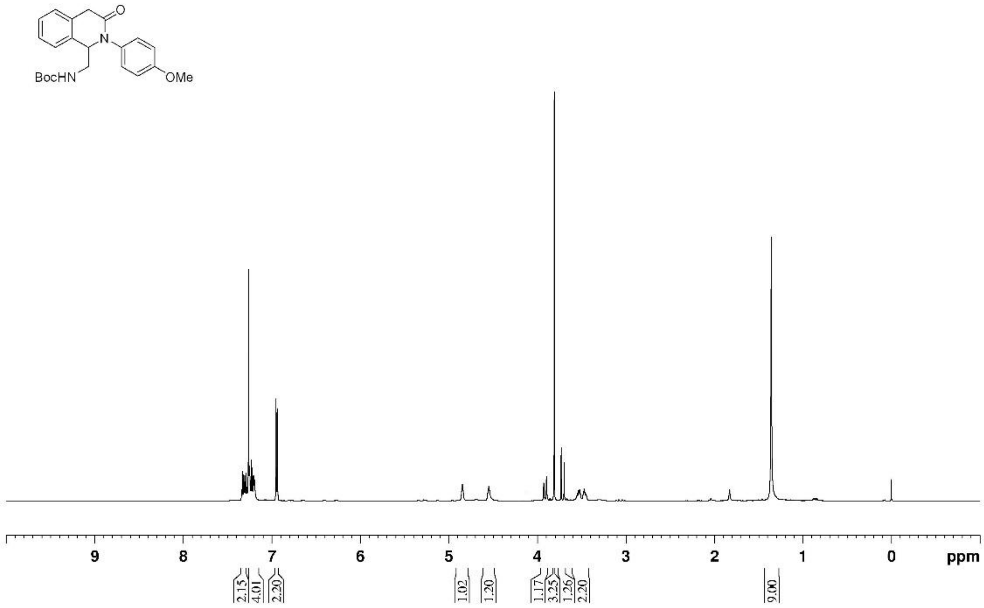

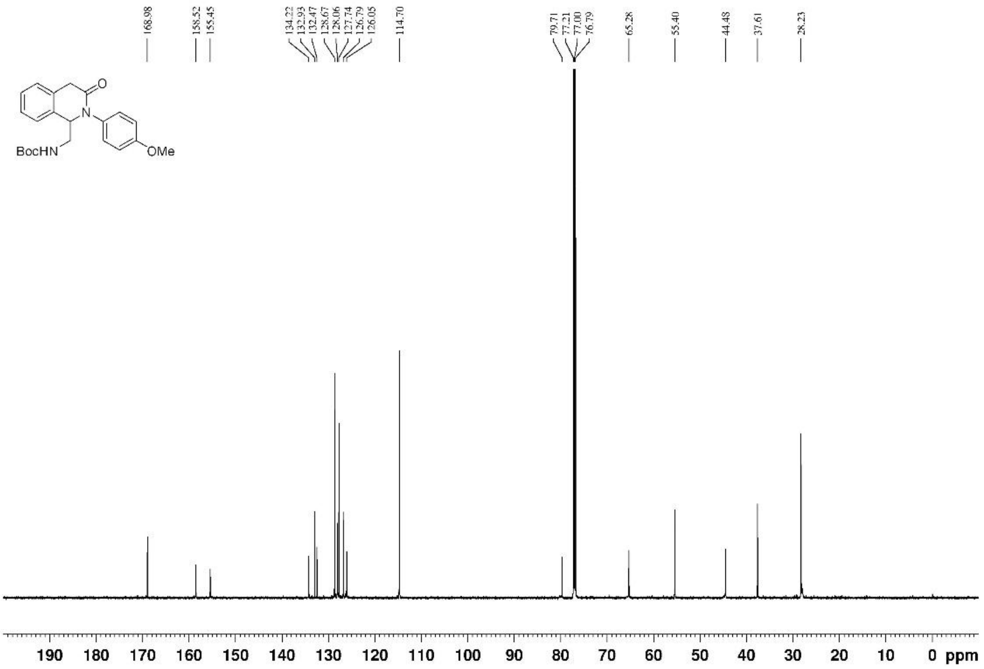

^{1}H NMR and ^{13}C NMR spectra of compound **28d**

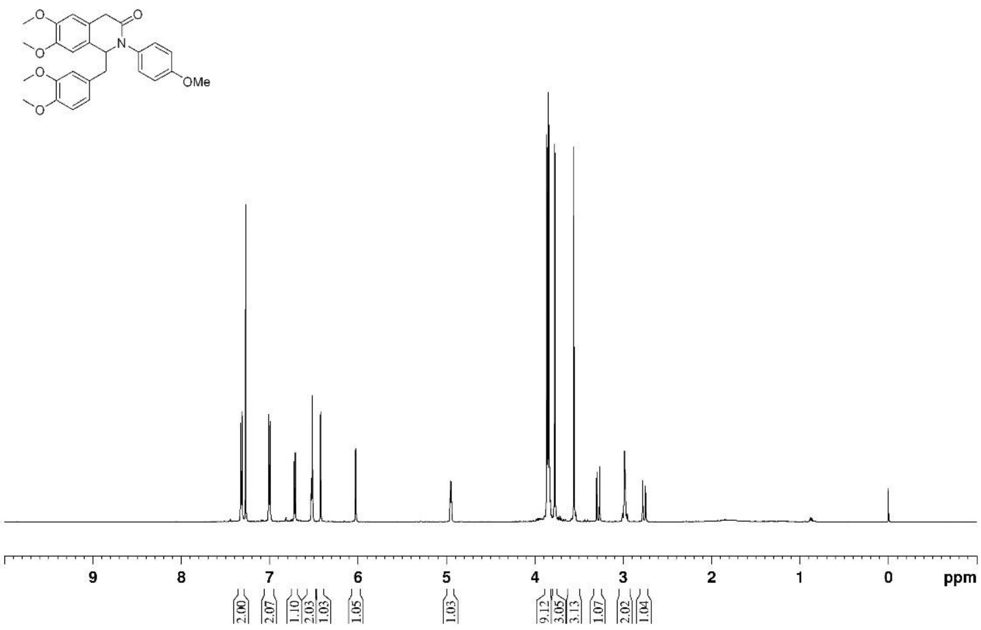

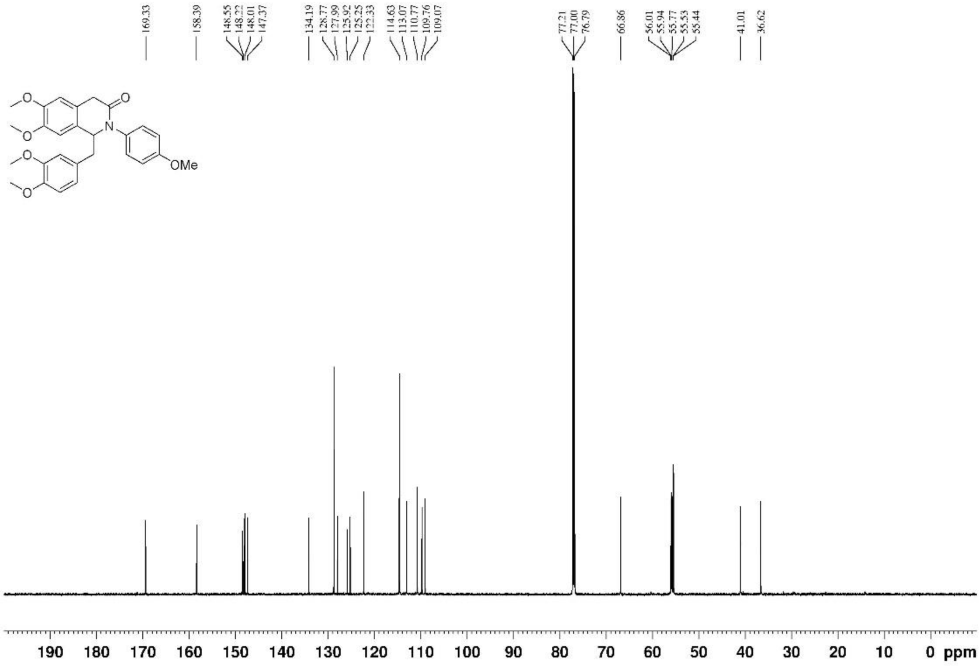

^{1}H NMR and ^{13}C NMR spectra of compound **30**

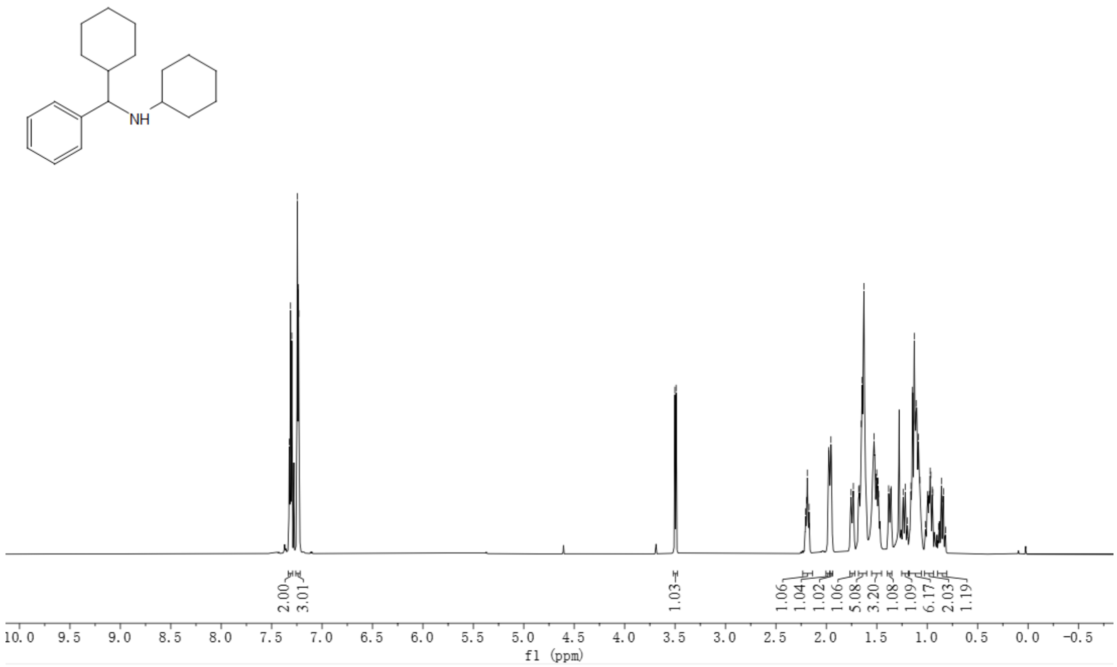

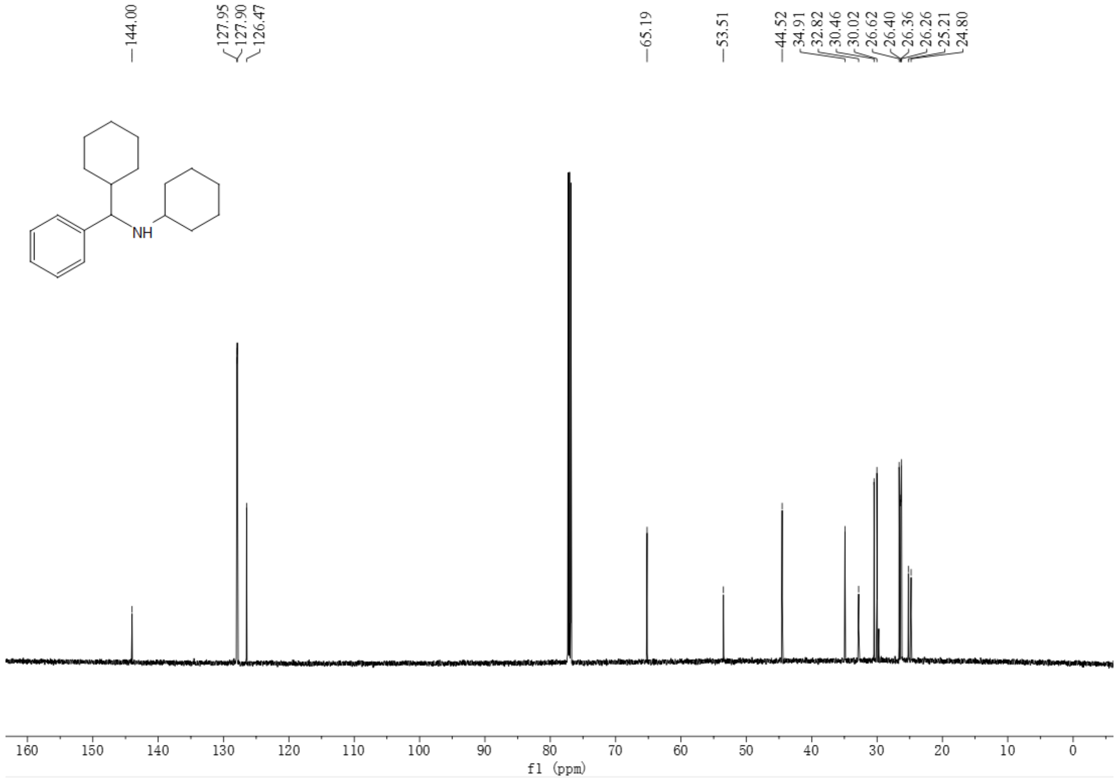

^{1}H NMR and ^{13}C NMR spectra of compound **32**

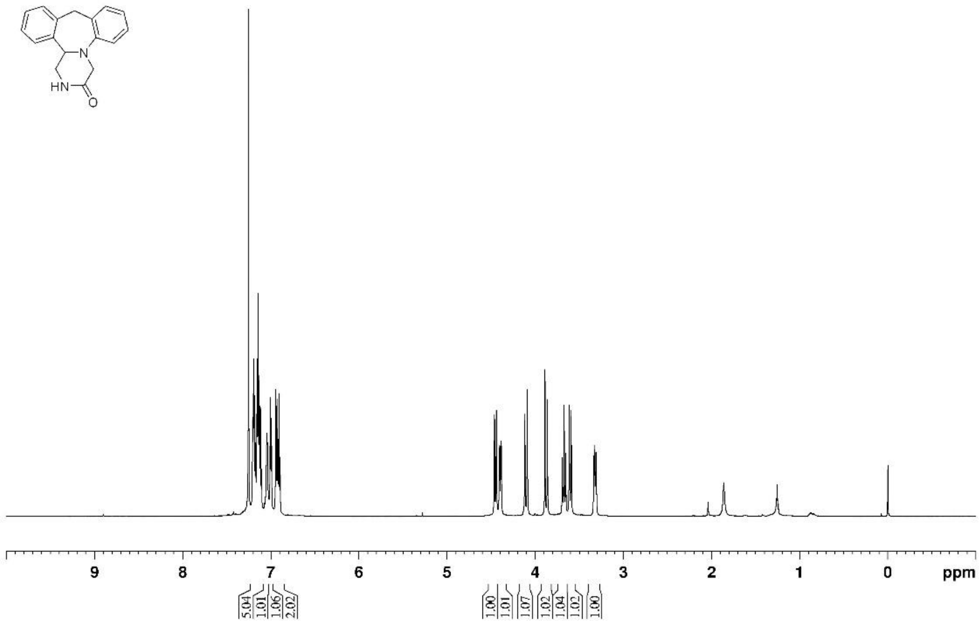

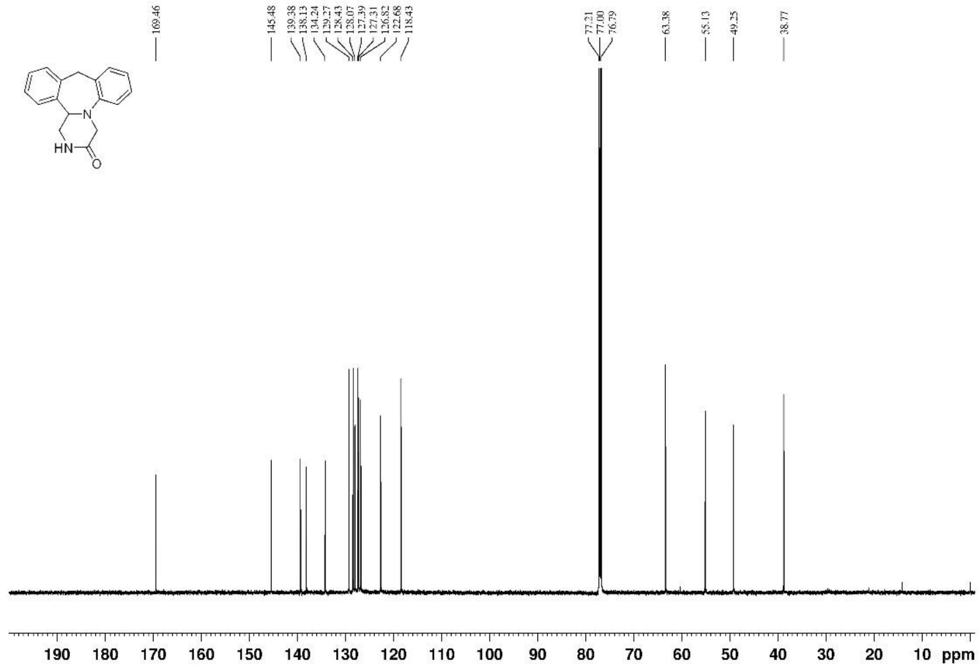

^{1}H NMR and ^{13}C NMR spectra of compound **33**

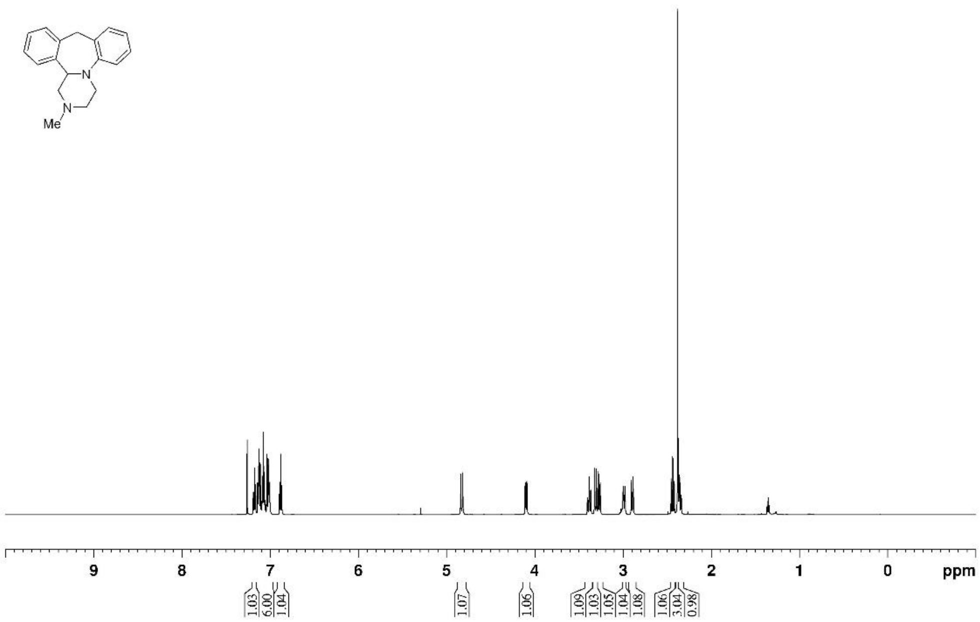

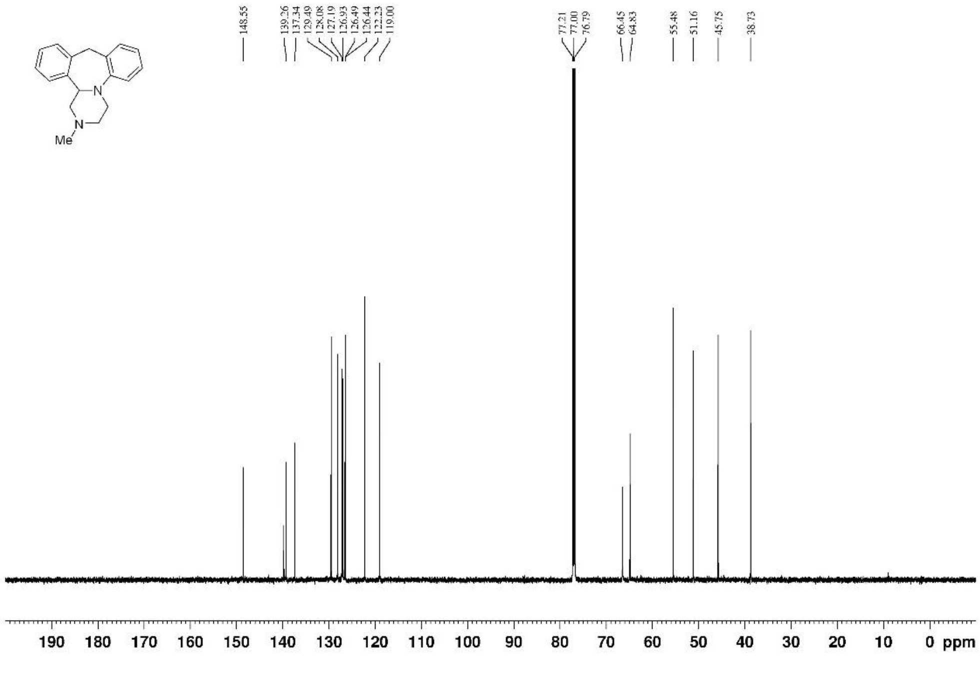

^{1}H NMR and ^{13}C NMR spectra of compound **34**

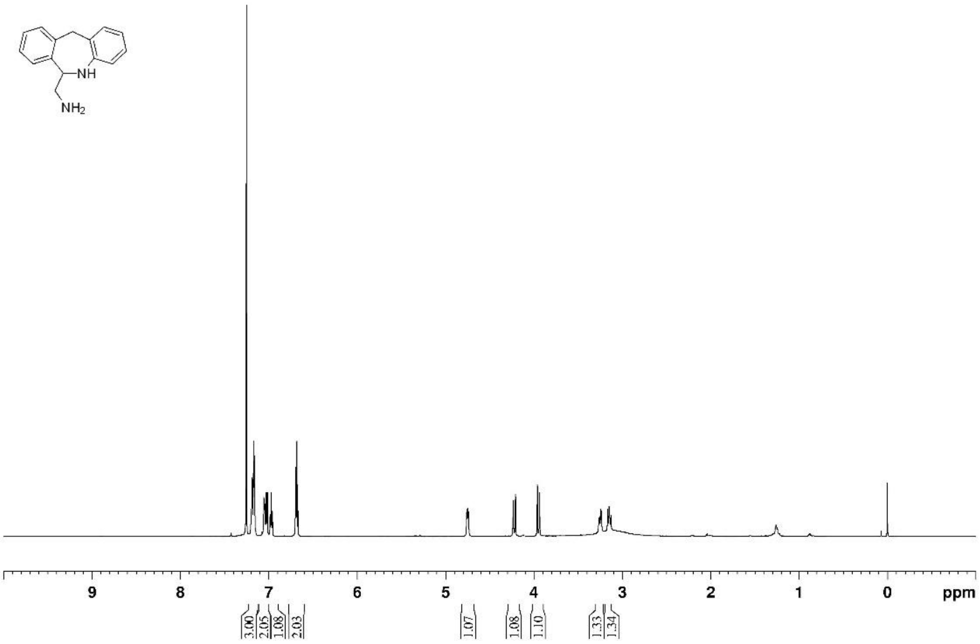

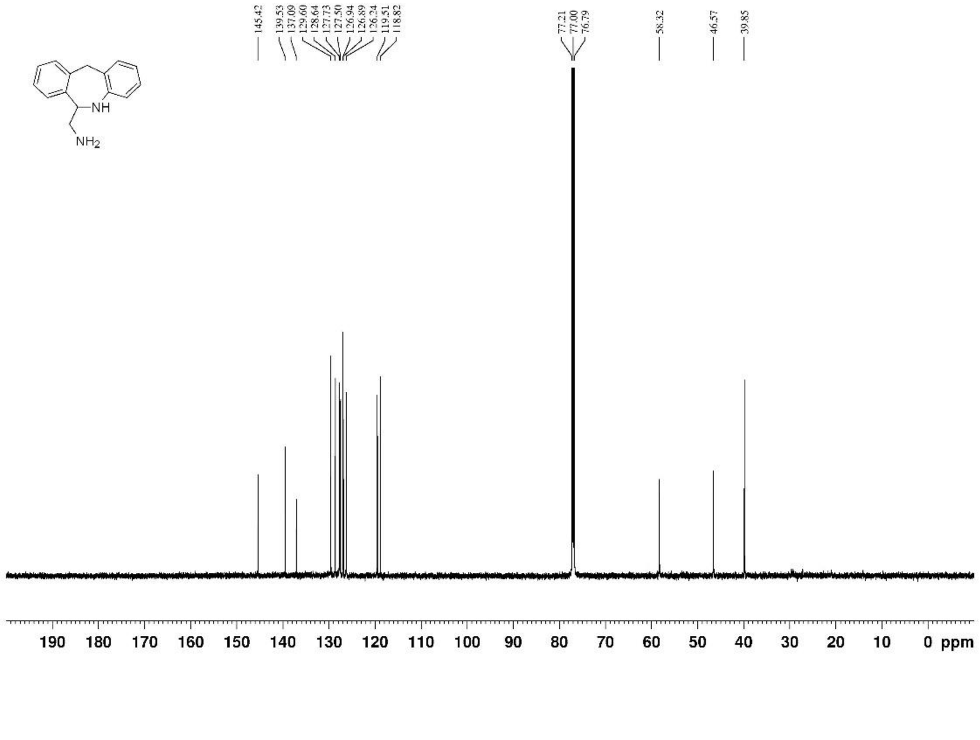

^{1}H NMR and ^{13}C NMR spectra of compound **35**

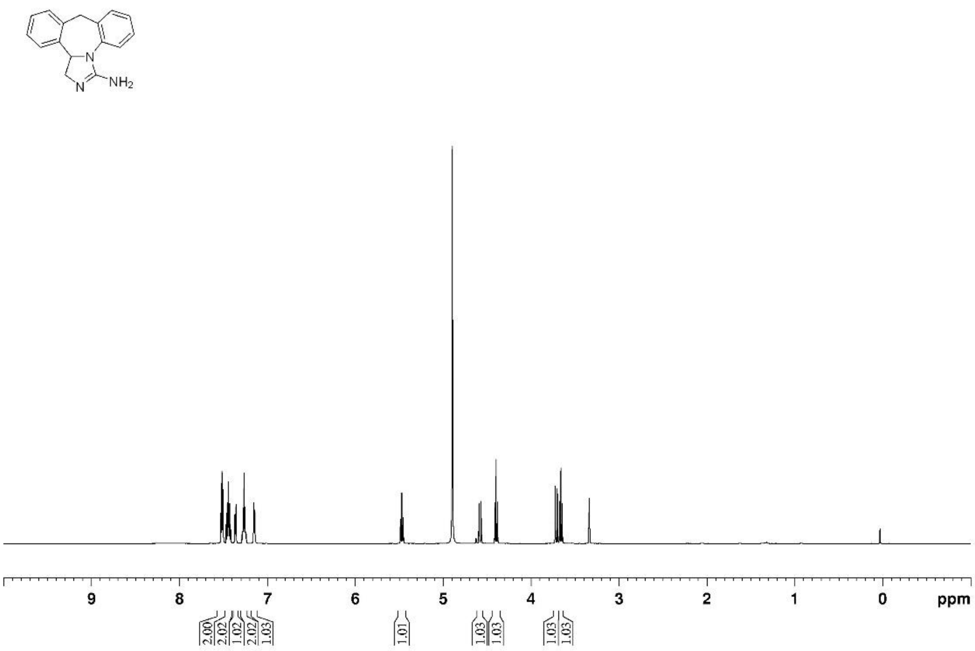
N
N
NH2
9
8
7
6
5
4
3
2
1
0
ppm
2.00
2.02
1.02
2.02
1.03
1.01
1.03
1.03
1.03
1.03

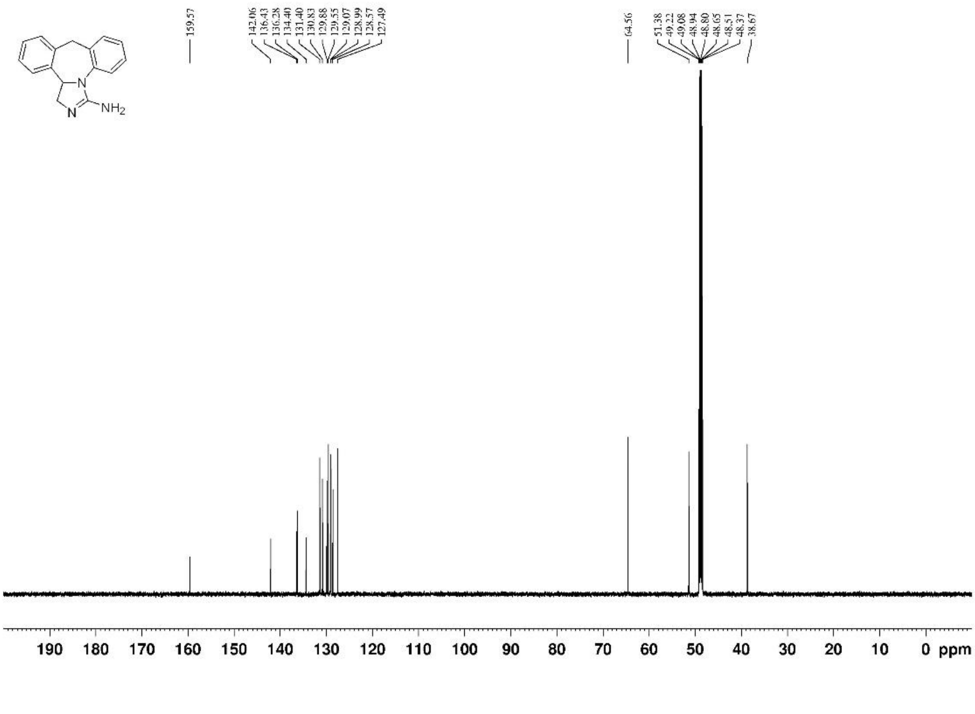
159.57
142.06
136.43
136.28
134.40
131.40
130.83
129.88
129.55
129.07
128.99
128.57
127.49
64.56
51.38
49.22
49.08
48.94
48.80
48.65
48.51
48.37
38.67
N
N
NH2
190
180
170
160
150
140
130
120
110
100
90
80
70
60
50
40
30
20
10
0
ppm

^{1}H NMR and ^{13}C NMR spectra of compound **36**

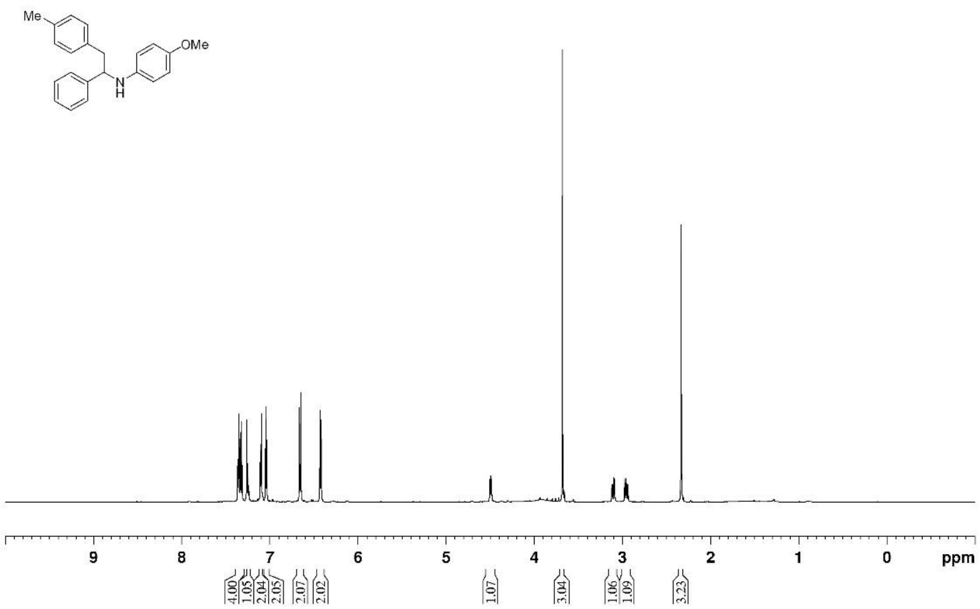

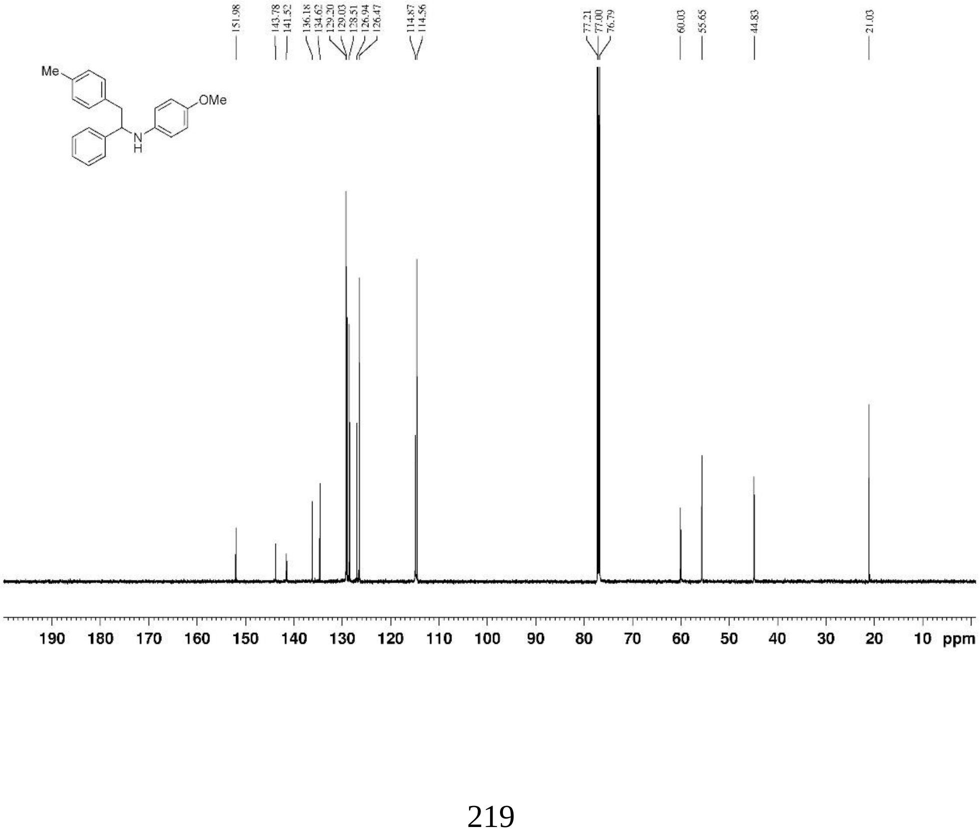

^{1}H NMR and ^{13}C NMR spectra of compound **36-d2**

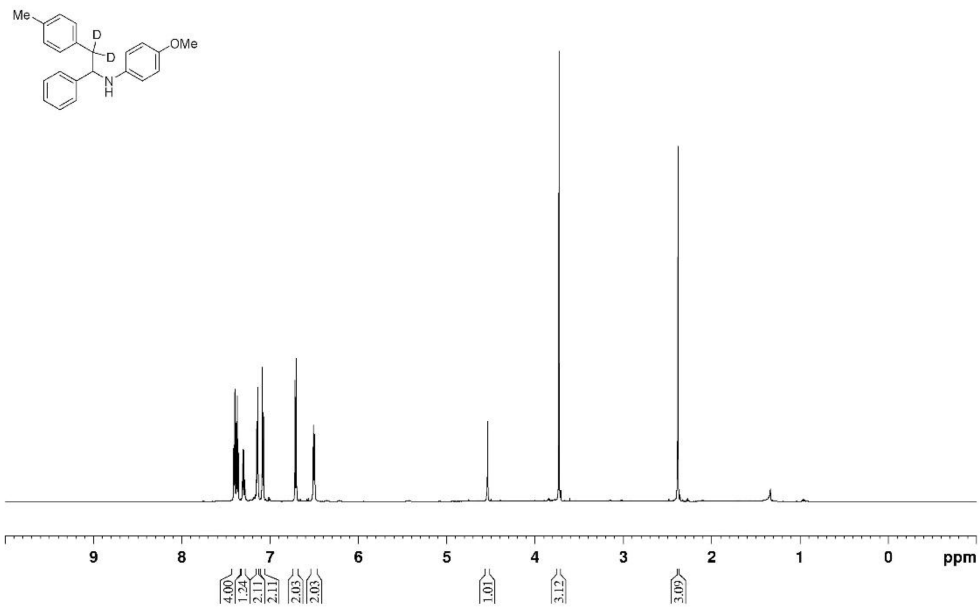

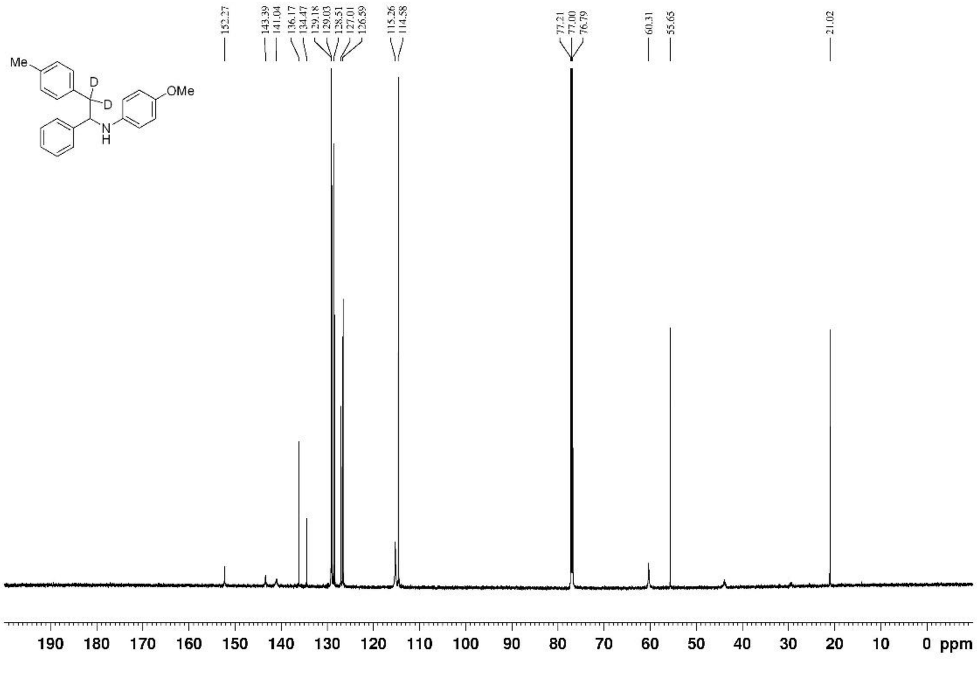

^{1}H NMR and ^{13}C NMR spectra of compound **37**

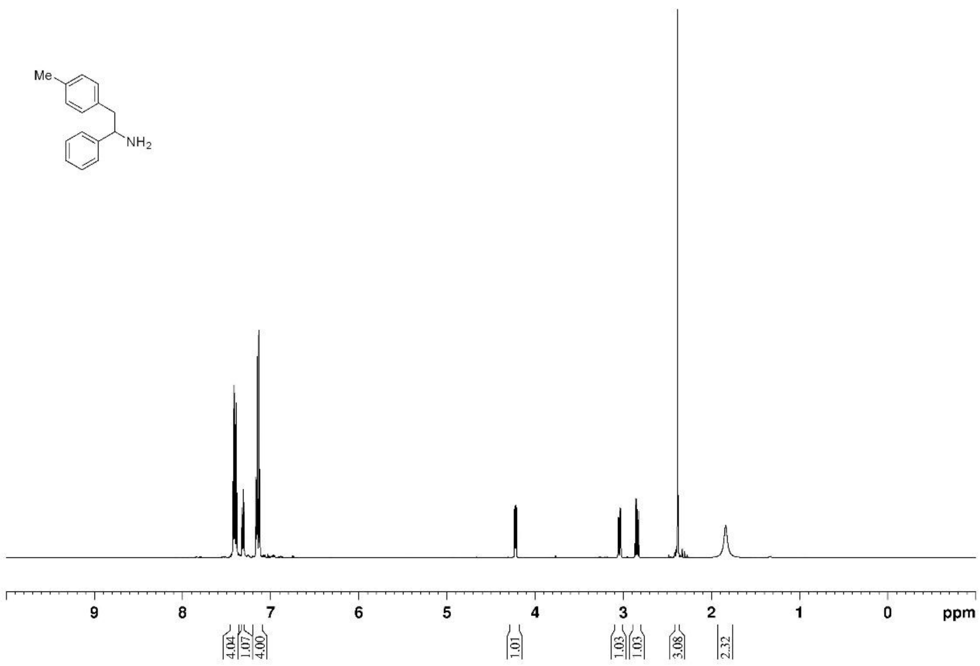

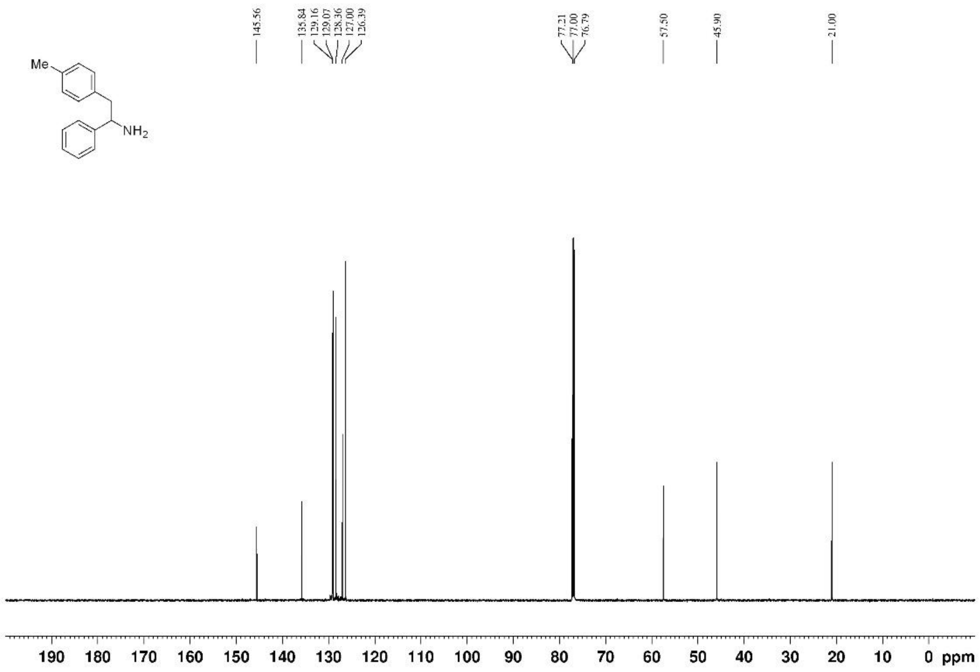

^{1}H NMR and ^{13}C NMR spectra of compound **37-d2**

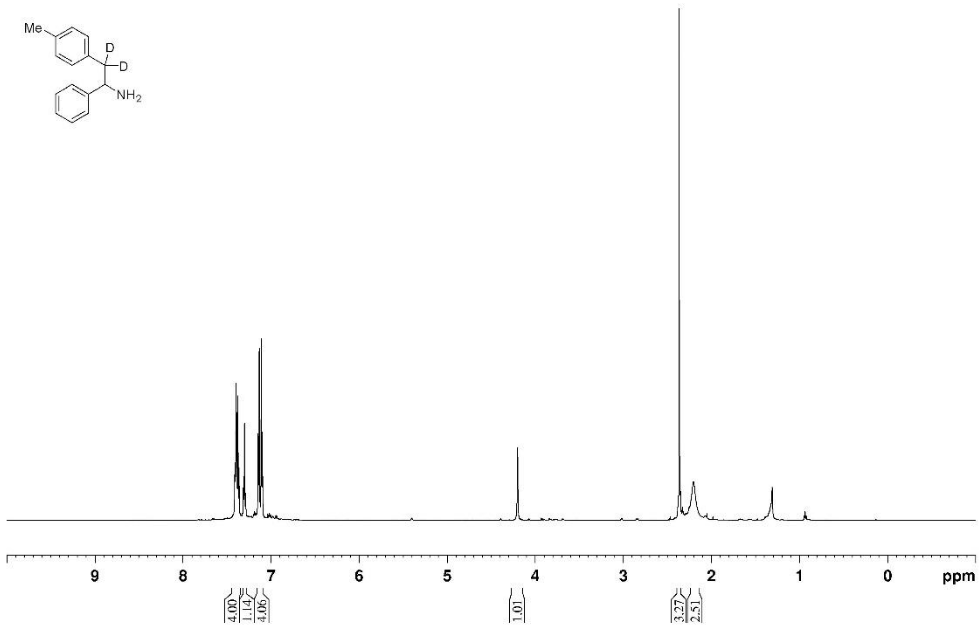

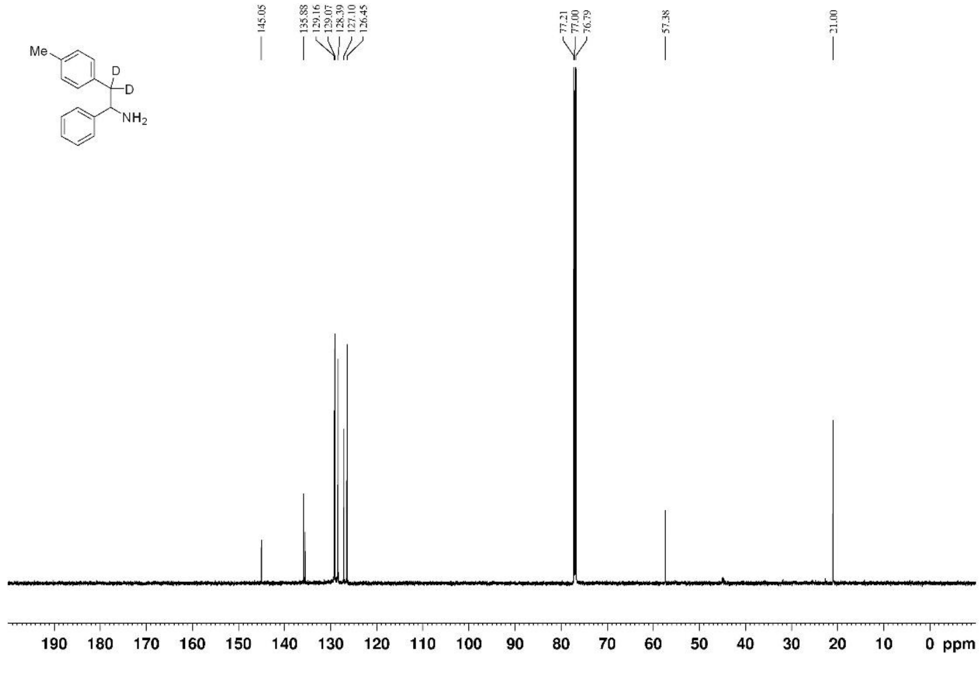

^{1}H NMR and ^{13}C NMR spectra of compound **38**

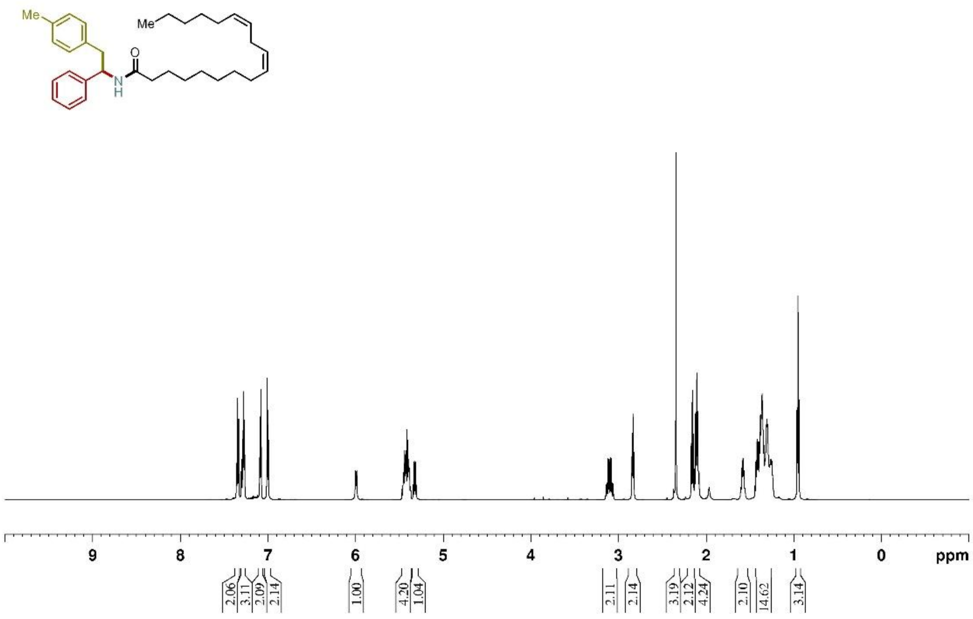

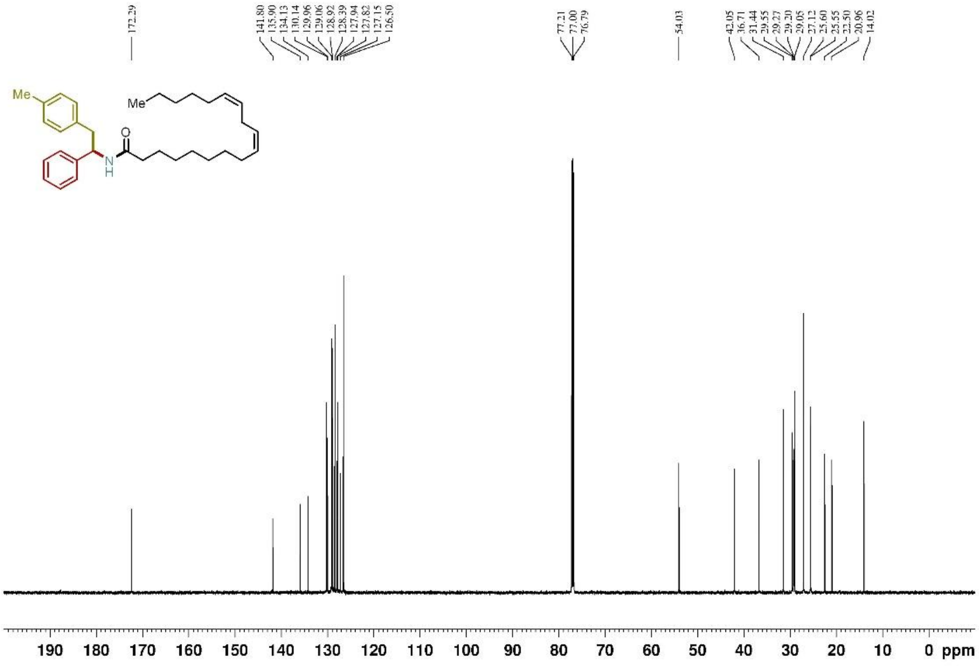

^{1}H NMR and ^{13}C NMR spectra of compound **38-d2**

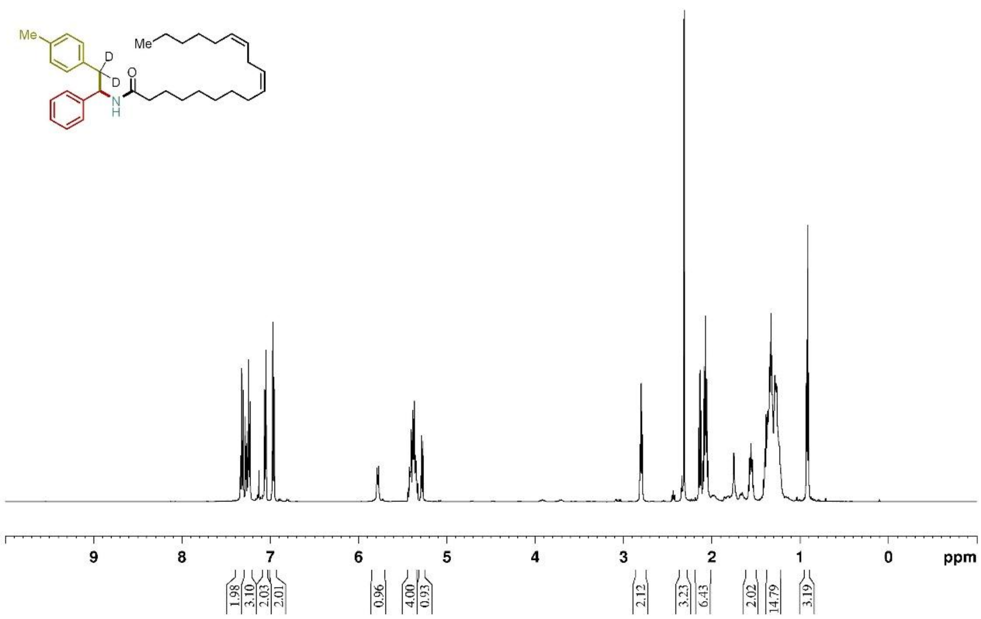

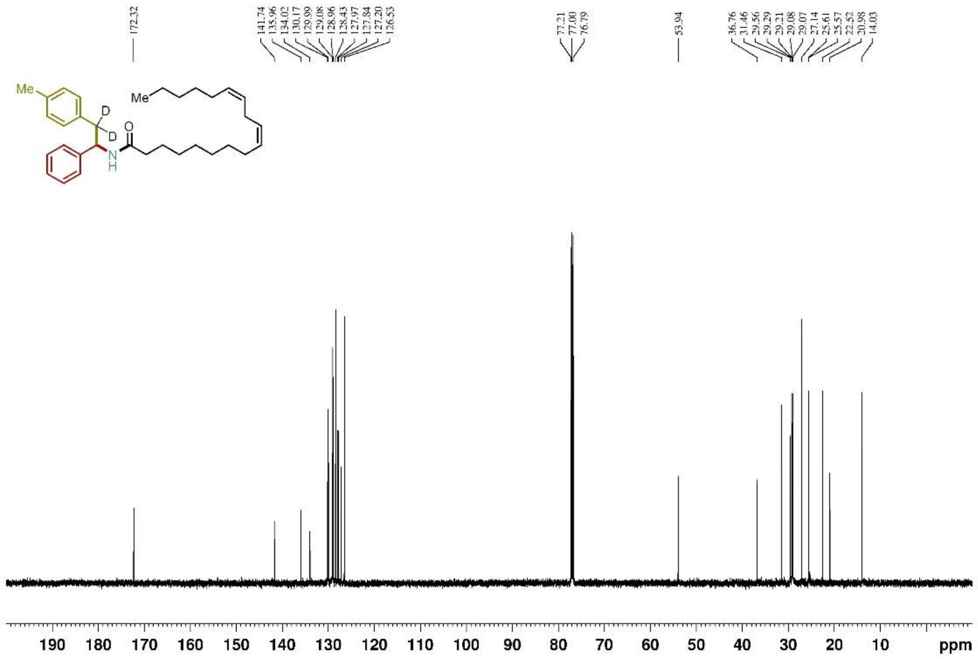

^{1}H NMR and ^{13}C NMR spectra of compound **39**

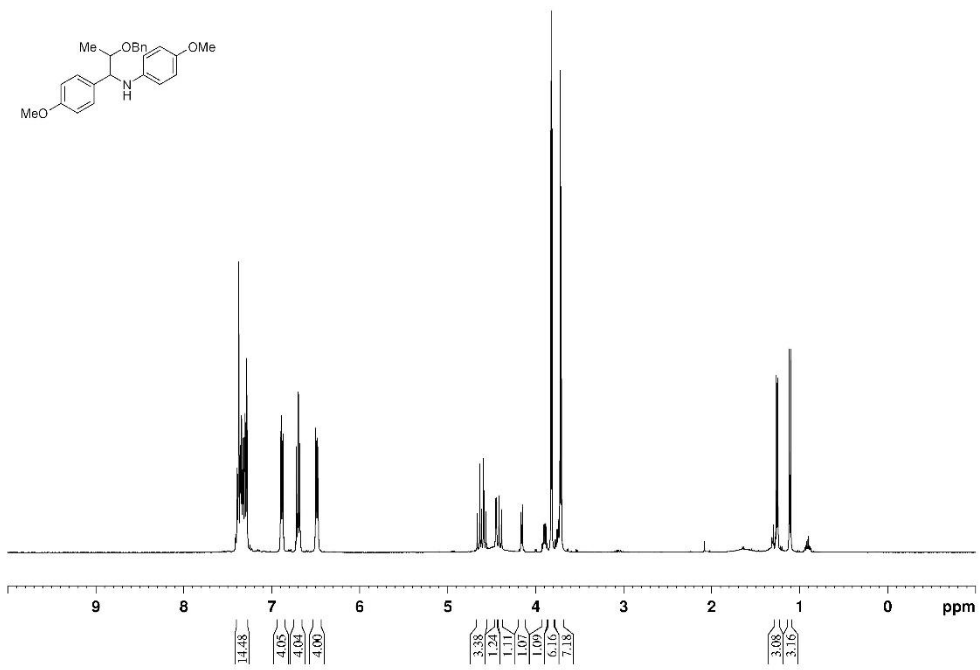

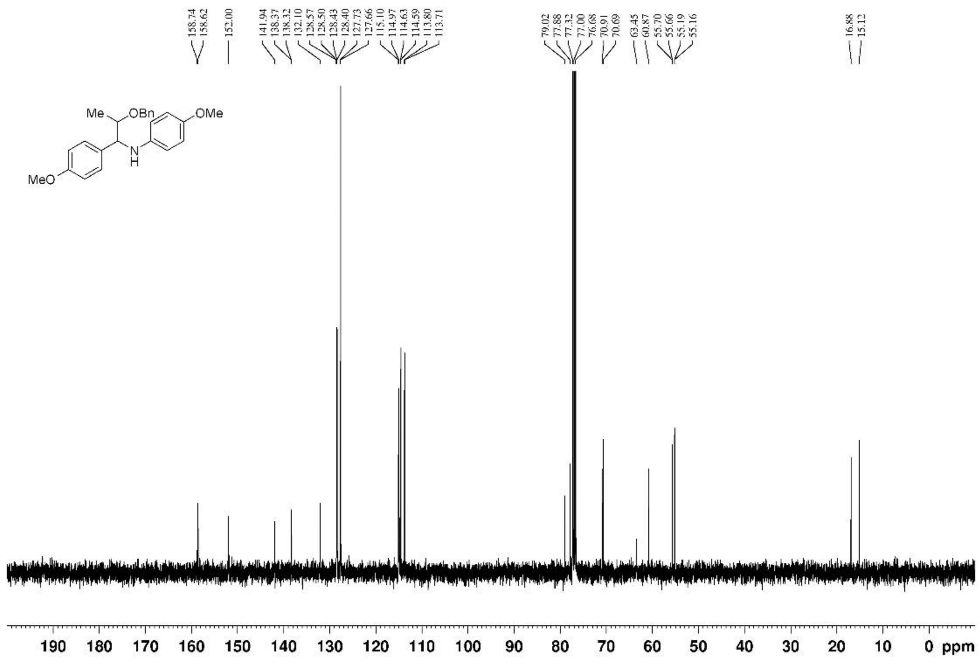

^{1}H NMR and ^{13}C NMR spectra of compound **40**

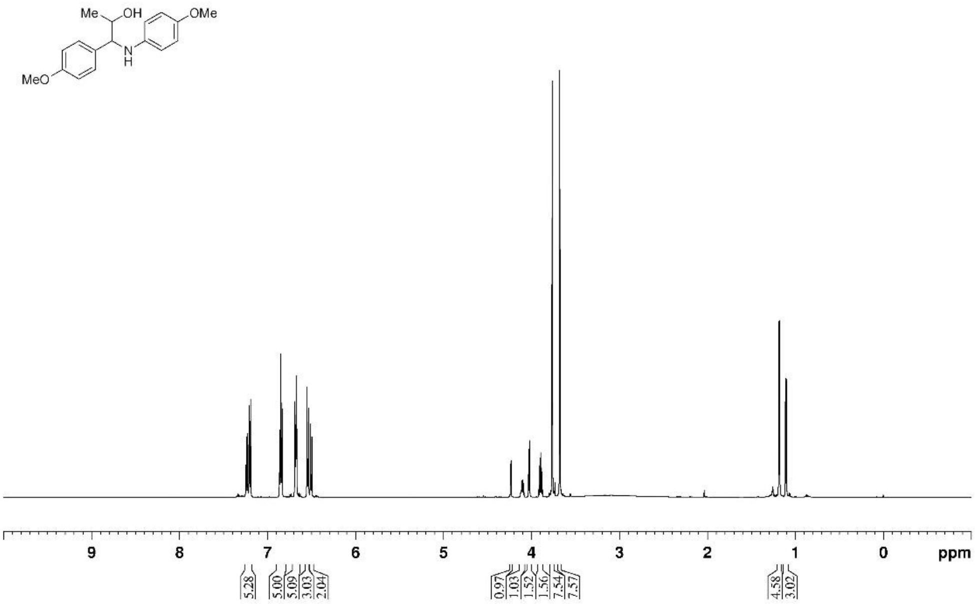

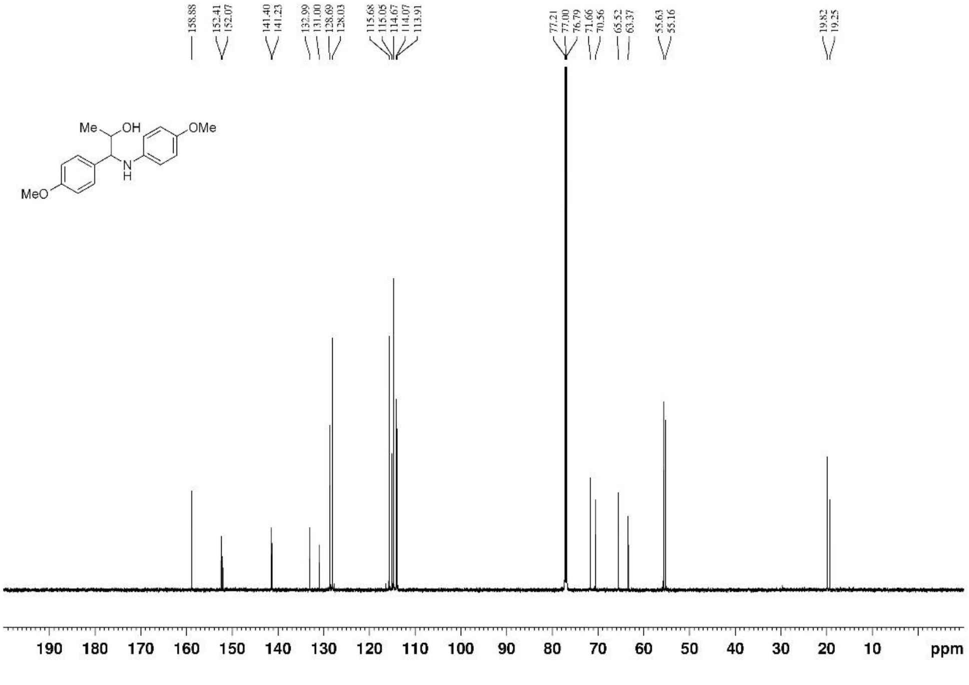

^{1}H NMR and ^{13}C NMR spectra of compound **41**

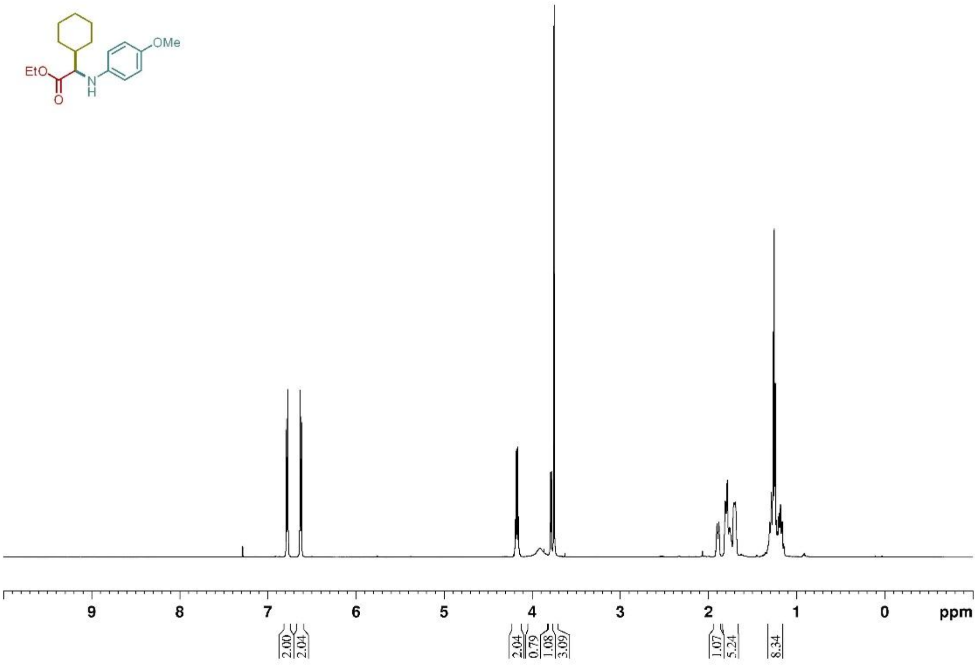

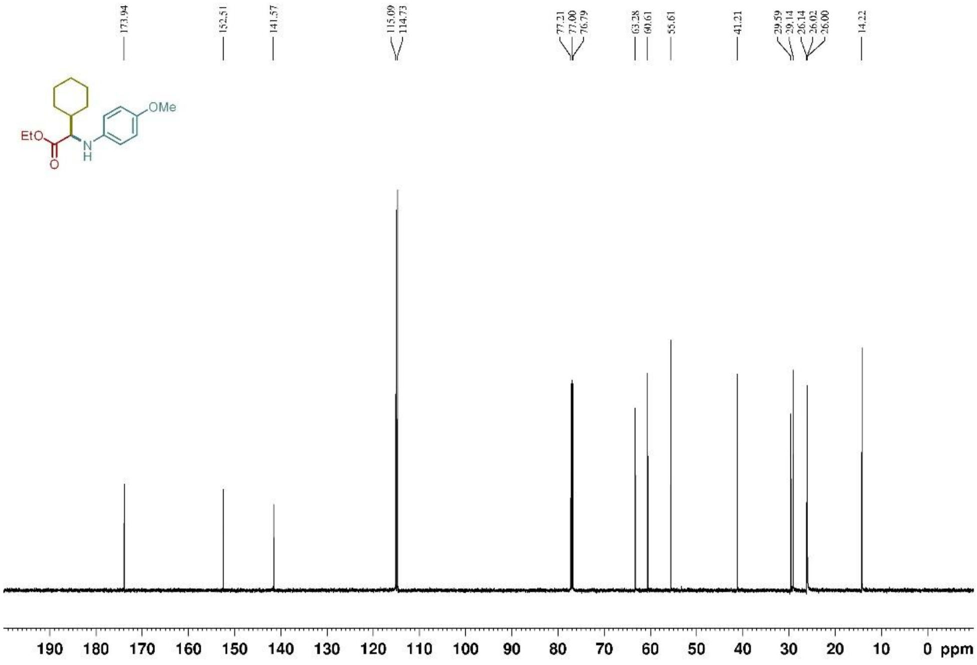

^{1}H NMR and ^{13}C NMR spectra of compound **42**

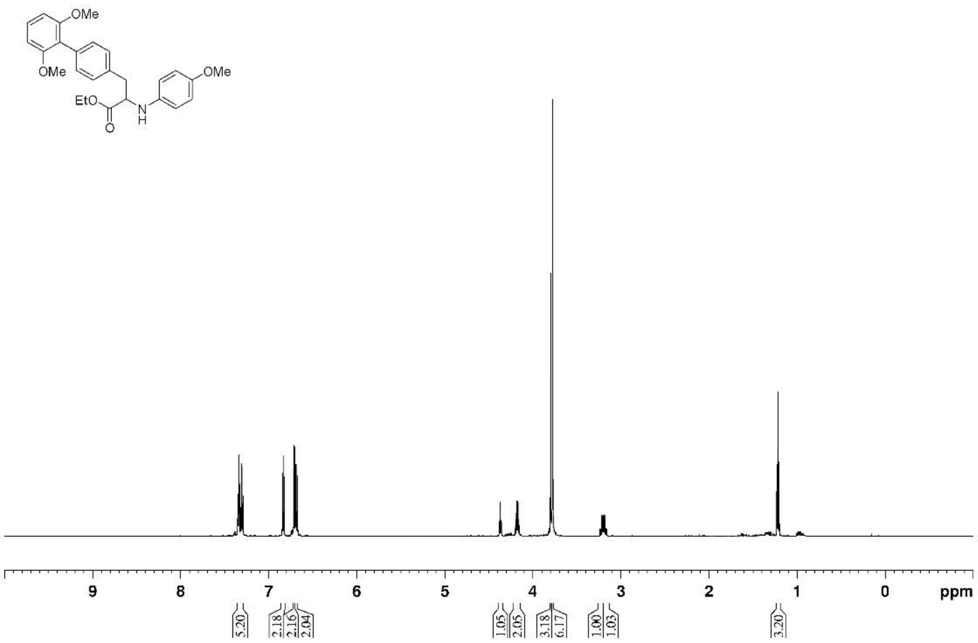

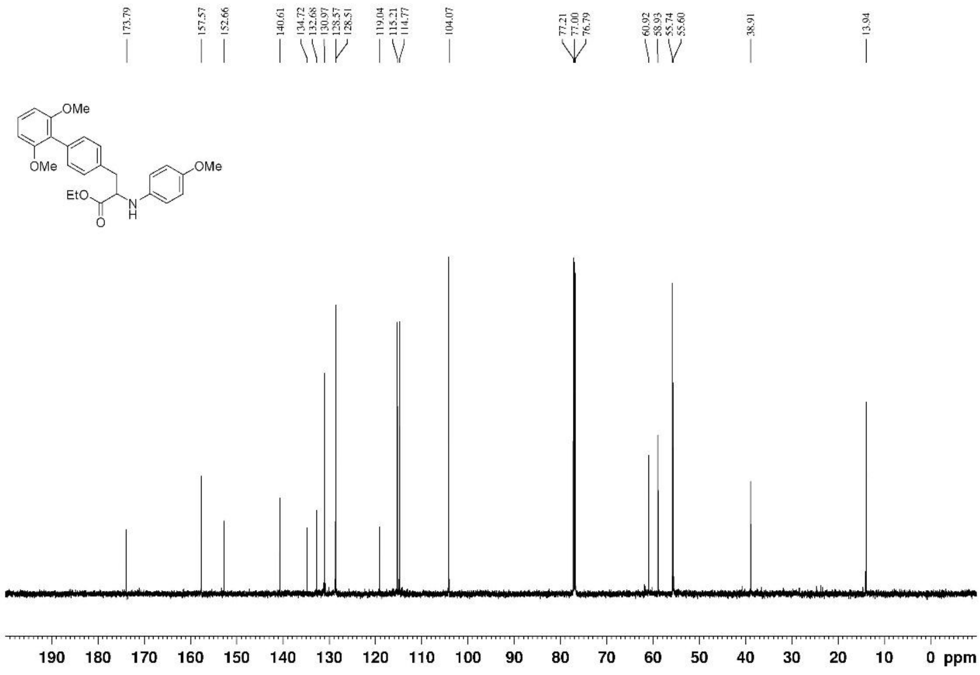

^{1}H NMR and ^{13}C NMR spectra of compound **43**

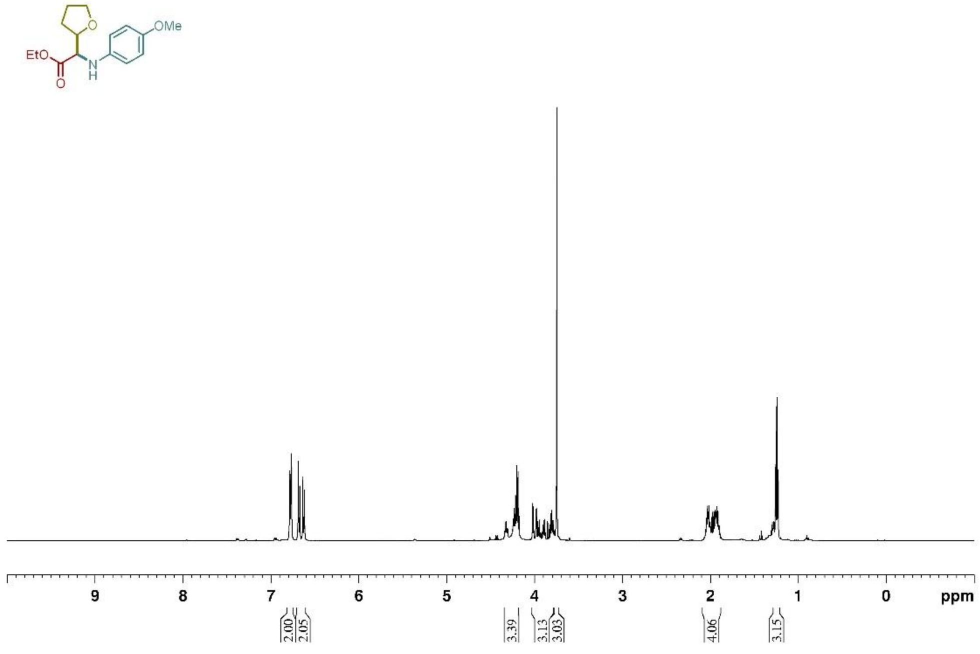

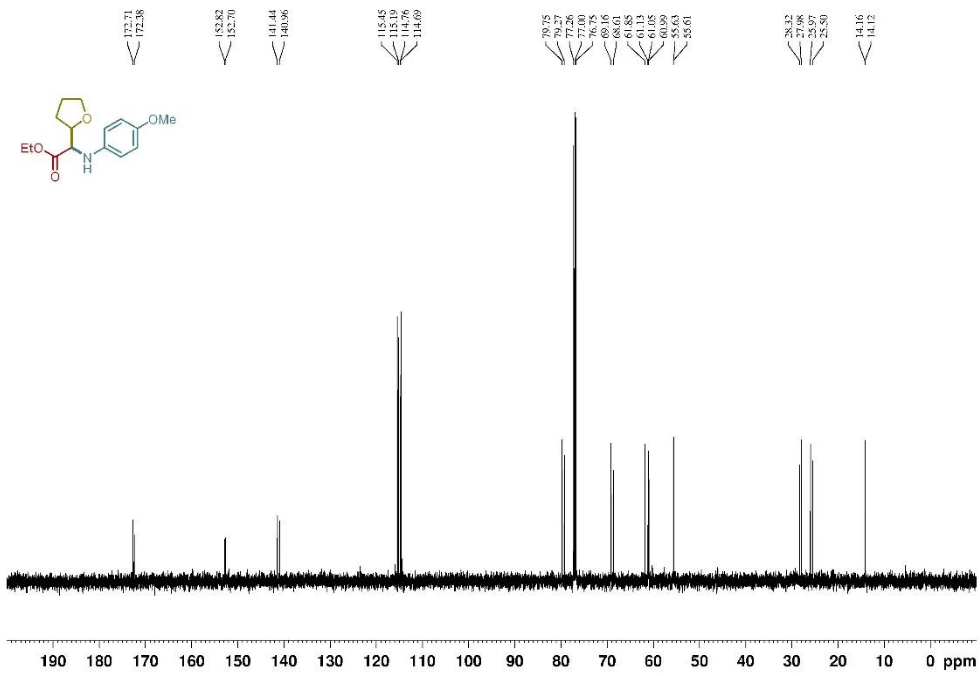

^{1}H NMR and ^{13}C NMR spectra of compound **44**

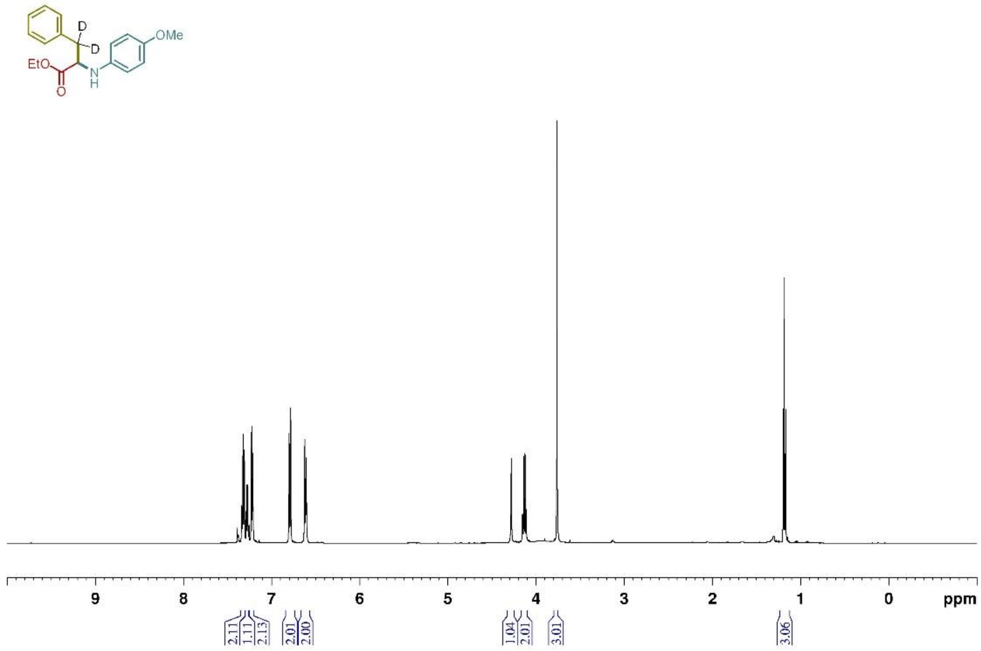

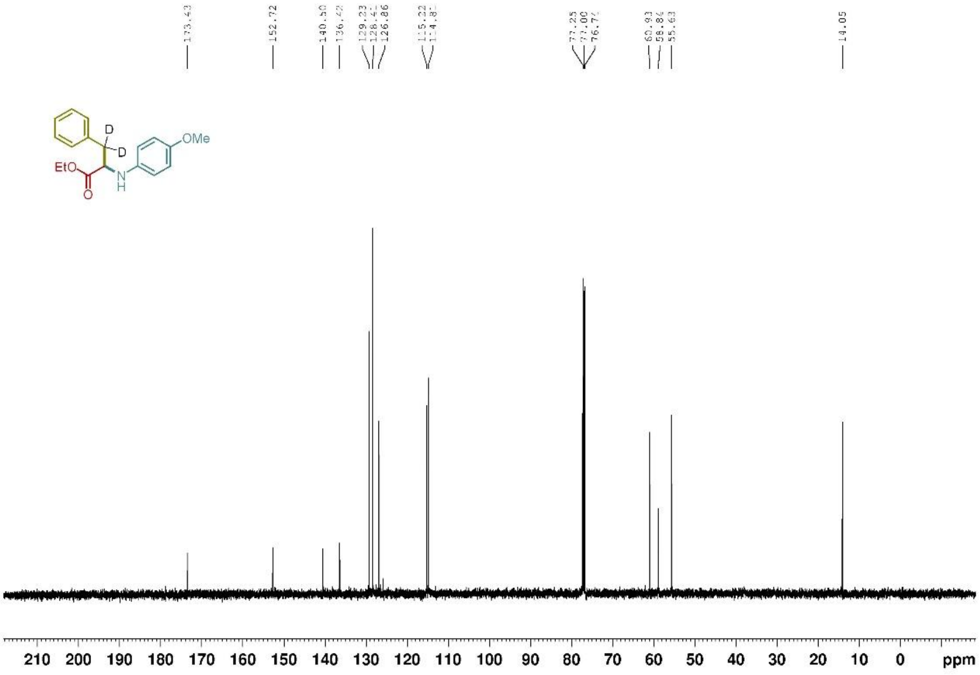

Supplement: nwag020_Supplemental_File [file nwag020_supplemental_file.pdf]
